# Photocatalytic Alkylation of C(sp^3^)−H Bonds Using Sulfonylhydrazones**

**DOI:** 10.1002/anie.202215374

**Published:** 2022-12-13

**Authors:** Antonio Pulcinella, Stefano Bonciolini, Florian Lukas, Andrea Sorato, Timothy Noël

**Affiliations:** ^1^ Flow Chemistry Group Van't Hoff Institute for Molecular Sciences (HIMS) University of Amsterdam Science Park 904 1098 XH Amsterdam The Netherlands

**Keywords:** Alkylation, Hydrazones, Hydrogen Atom Transfer, Photocatalysis, Synthetic Methods

## Abstract

The ability to construct C(sp^3^)−C(sp^3^) bonds from easily accessible reagents is a crucial, yet challenging endeavor for synthetic organic chemists. Herein, we report the realization of such a cross‐coupling reaction, which combines *N‐*sulfonyl hydrazones and C(sp^3^)−H donors through a diarylketone‐enabled photocatalytic hydrogen atom transfer and a subsequent fragmentation of the obtained alkylated hydrazide. This mild and metal‐free protocol was employed to prepare a wide array of alkyl‐alkyl cross‐coupled products and is tolerant of a variety of functional groups. The application of this chemistry further provides a preparatively useful route to various medicinally‐relevant compounds, such as homobenzylic ethers, aryl ethyl amines, β‐amino acids and other moieties which are commonly encountered in approved pharmaceuticals, agrochemicals and natural products.

## Graphical abstract

Herein, we report the realization of an alkyl‐alkyl cross‐coupling reaction, which combines *N‐*sulfonyl hydrazones and C(sp^3^)−H donors through a diarylketone‐enabled photocatalytic hydrogen atom transfer and a subsequent fragmentation of the obtained alkylated hydrazide. This metal‐free protocol provides a preparatively useful route to various medicinally‐relevant compounds, such as homobenzylic ethers, aryl ethyl amines, and β‐amino acids.

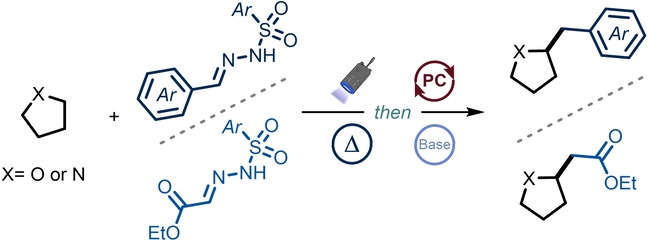

The synthetic routes of many biologically active molecules have been dominated by the use of C(sp^2^)−C(sp^2^) cross‐coupling reactions, resulting in a significant bias towards flat molecules.^[1]^ To improve the probability of finding new potent drugs and agrochemicals, a new trend has emerged to increase the sp^3^‐character of new leads (so‐called Escape‐from‐Flatland strategy).[^1^, ^2^] Consequently, there is a pressing need to develop new synthetic strategies that can realize the creation of C(sp^3^)−C(sp^3^) bonds.^[3]^

Historically, alkyl‐alkyl couplings have been challenging to accomplish, but in recent years significant progress has been achieved using transition‐metal catalysis, particularly by exploiting nickel‐based complexes.[^4^, ^5^, ^6^, ^7^, ^8^, ^9^, ^10^, ^11^, ^12^, ^13^, ^14^, ^15^, ^16^] These methods rely on the availability of prefunctionalized electrophiles (e.g., alkyl halides) and nucleophilic coupling partners (i.e., organometallic reagents), and often require high catalyst loadings, which necessitate energy‐intensive purification strategies to remove any trace metal impurities.^[15]^

Alternatively, an attractive option to enable C(sp^3^)−C(sp^3^) bond formation would be to use easily accessible reagents in the absence of any transition metals, which would also facilitate applications that require higher regulatory scrutiny, such as the late‐stage functionalization of medicinally relevant compounds.[^17^, ^18^, ^19^, ^20^] To realize this synthetic goal, we were drawn to *N‐*sulfonyl hydrazones, which are stable, crystalline compounds and are readily synthesized from the corresponding aldehydes (Figure 1A).[^21^, ^22^, ^23^, ^24^] Notably, these *N‐*sulfonyl hydrazones can be exploited as a suitable electrophilic site for both polar and radical addition to yield, upon dinitrogen and sulfinate extrusion, the targeted C(sp^3^)−C(sp^3^) bonds.[^25^, ^26^, ^27^, ^28^, ^29^, ^30^, ^31^, ^32^]

**Figure 1:**
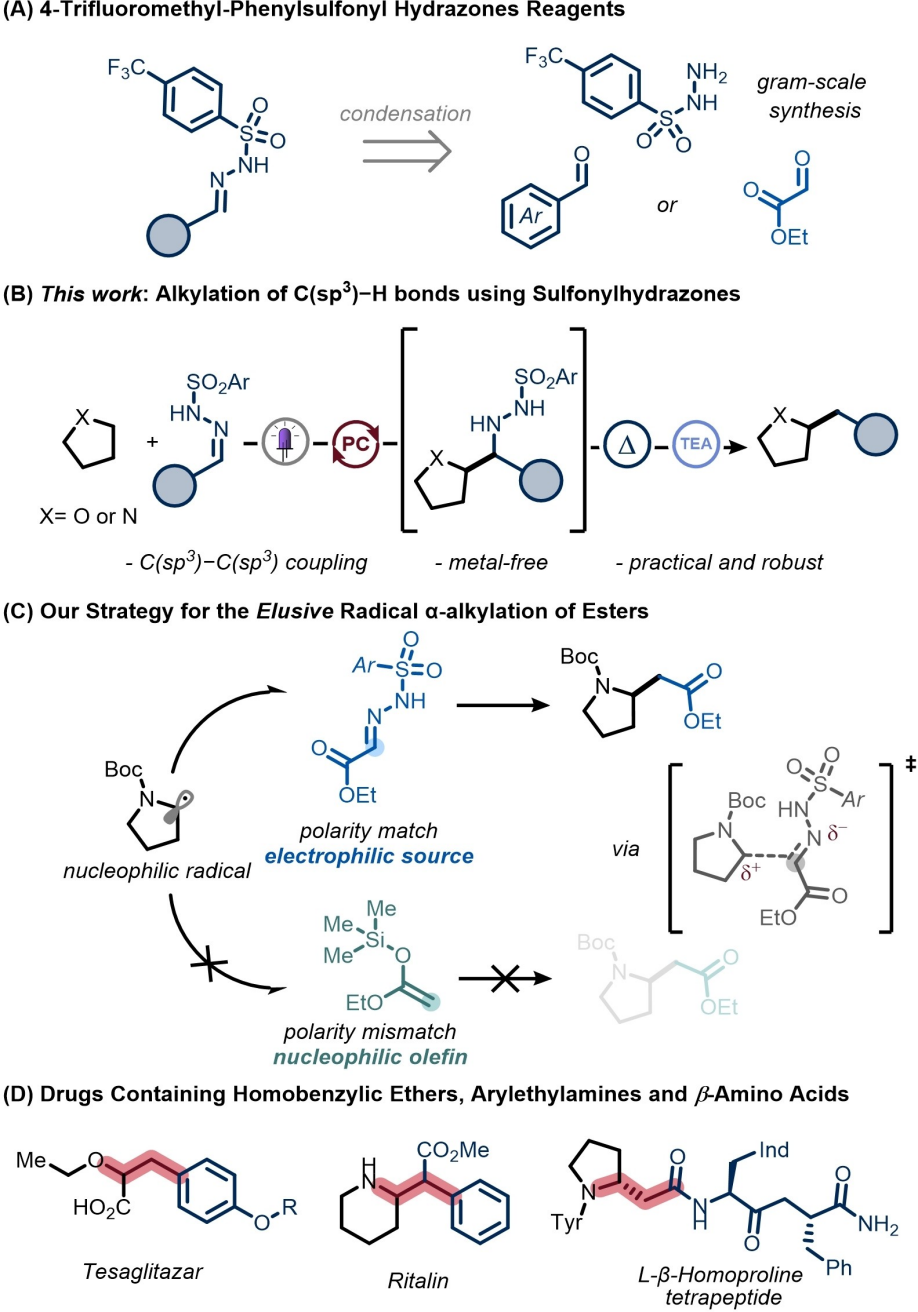
4‐trifluoromethyl‐phenyl sulfonyl hydrazones as practical radical acceptors to promote C(sp^3^)−C(sp^3^) coupling.

To realize a metal‐free protocol, we anticipated that a photocatalytic hydrogen atom transfer (HAT) strategy would be appealing to activate aliphatic C−H bonds,[^33^, ^34^, ^35^, ^36^, ^37^, ^38^, ^39^, ^40^, ^41^, ^42^, ^43^, ^44^, ^45^] thus generating from cheap commodity chemicals the required nucleophilic radicals which can subsequently add to the polarity‐matched *N‐*sulfonylhydrazone. Herein, we describe the realization of such a photocatalytic denitrogenative alkylation of *N*‐sulfonyl hydrazones using diarylketones as the photocatalyst (Figure 1B). Furthermore, the use of glyoxylate‐derived sulfonyl hydrazones enables the formal α‐alkylation of esters using cheap aliphatic scaffolds as radical precursors (Figure 1C). It should be noted that these compounds cannot be prepared from common silyl ketene acetals as radical acceptors due to the polarity mismatch between the electron‐rich alkene and the nucleophilic alkyl radical (Figure 1C).[^46^, ^47^, ^48^, ^49^, ^50^] Finally, we show that the application of this chemistry provides a preparatively useful route to various medicinally‐relevant compounds, such as homobenzylic ethers, aryl ethyl amines, β‐amino acids and other moieties which are commonly encountered in approved pharmaceuticals and natural products (Figure 1D).[^51^, ^52^, ^53^, ^54^, ^55^, ^56^, ^57^]

Our experimental investigation began with the optimization of the photocatalytic HAT step. After extensive screening of all relevant reaction parameters (see Supporting Information, section 5.1), we found that when a solution of 4‐trifluoromethyl‐phenylsulfonyl hydrazone **1 a** (1.0 equiv.) as radical acceptor, tetrahydrofuran (THF) as C−H donor in the presence of 4, 4′‐dichlorobenzophenone (**4‐Cl_2_‐BP**, 20 mol %) as a HAT photo‐organocatalyst in trifluorotoluene (TFT) (0.1 M) was subjected to 40 W Kessil PR160L‐390 nm irradiation, the targeted alkylated hydrazide **2 a** could be isolated in excellent yield (Table 1A, Entry 1, 90 % yield). In the absence of either the photocatalyst or light, no product formation was observed, thus verifying the photocatalytic nature of this transformation (Table 1A, Entries 2, 3). As expected, heating the reaction mixture in the dark also resulted in the quantitative recovery of **1 a** (Table 1A, Entry 4). When dichloromethane (DCM) was evaluated as solvent, reduced yields for **2 a** were observed (Table 1A, Entry 5). A high yield for hydrazide **2 a** was obtained when equal volumes of THF and TFT were used (Table 1A, Entry 6, 91 % yield).

**Table 1:**
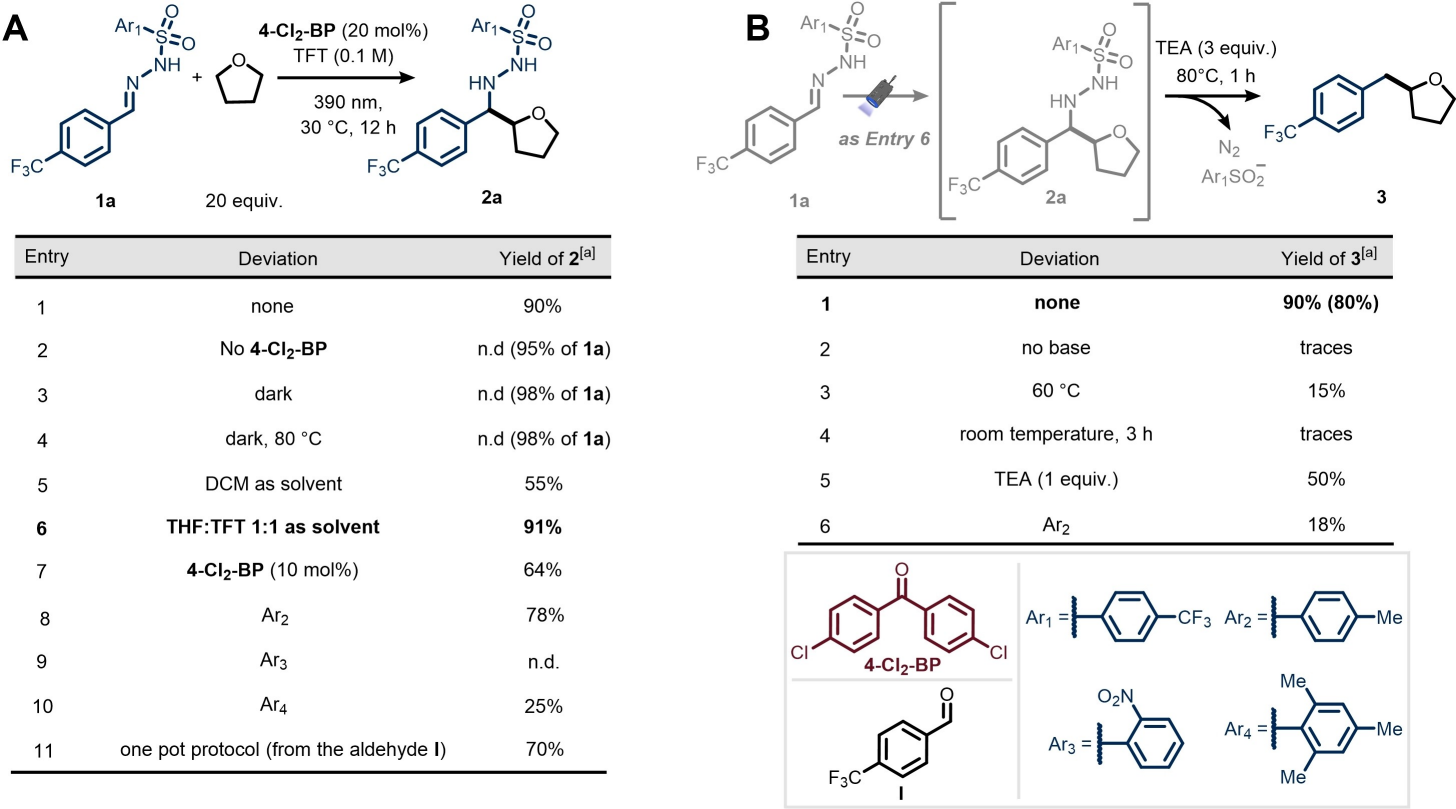
Optimization of reaction conditions: photocatalytic HAT (A) and fragmentation (B) steps.

[a] Yields were determined by ^1^H NMR using trichloroethylene as external standard. 0.2 mmol scale (0.1 M). In parenthesis yields after column chromatography. See Supporting Information for experimental details.

However, lowering the catalyst loading of **4‐Cl_2_‐BP** from 20 to 10 mol % resulted in lower yields (Table 1A, Entry 7). Also the electronic properties of the aryl sulfonyl group of the hydrazone were investigated: while the commonly used tosyl (Ar_2_) and mesityl (Ar_4_) variants resulted in lower yields (Table 1A, Entries 8 and 10),^[58]^ the presence of a more electron‐withdrawing ring completely shuts down the reaction (Table 1A, Entry 9). Of particular note is the practical one‐pot protocol in which the hydrazone is generated in situ from 4‐(trifluoromethyl)benzaldehyde (70 % yield, Table 1A, Entry 11).

Having established suitable conditions for the photocatalytic event, we turned our attention to optimize the ensuing fragmentation of intermediate **2 a**, which would ultimately yield the targeted cross‐coupled product **3**.^[30]^ A one‐pot, two‐step protocol was developed, in which direct addition of 3 equivalents of triethylamine (TEA) and heating the reaction mixture to 80 °C afforded **3** in good isolated yield (Table 1B, Entry 1). Control experiments revealed that both the presence of a base and the supply of thermal energy were crucial (Table 1B, Entries 2–4). Moreover, decreasing the amount of base led to a reduced reaction efficiency (Table 1B, Entry 5). Surprisingly, when using the corresponding tosyl hydrazone, the denitrogenative cleavage proceeded in lower yield, further highlighting the key role of 4‐trifluoromethyl‐phenylsulfonyl group in both reaction steps (Table 1B, Entry 6).

With optimal conditions in hand (see Table 1B, Entry 1), we tested the generality of the one‐pot alkylation protocol (Figure 2). First, we combined THF as C−H donor with a diverse set of aromatic aldehyde‐derived 4‐trifluoromethyl‐phenylsulfonyl hydrazones, as shown in Figure 2. Electron‐poor, ‐neutral and ‐rich aromatic aldehyde‐derived hydrazones successfully served as radical acceptors, affording the corresponding homobenzylic ether products in good isolated yields (**3**–**20**, 43–86 % yield). Notably, our method tolerates well the presence of halogen atoms (**7**–**12**), which are useful synthetic handles for further manipulation with classical transition‐metal‐promoted cross‐coupling reactions.^[15]^

**Figure 2:**
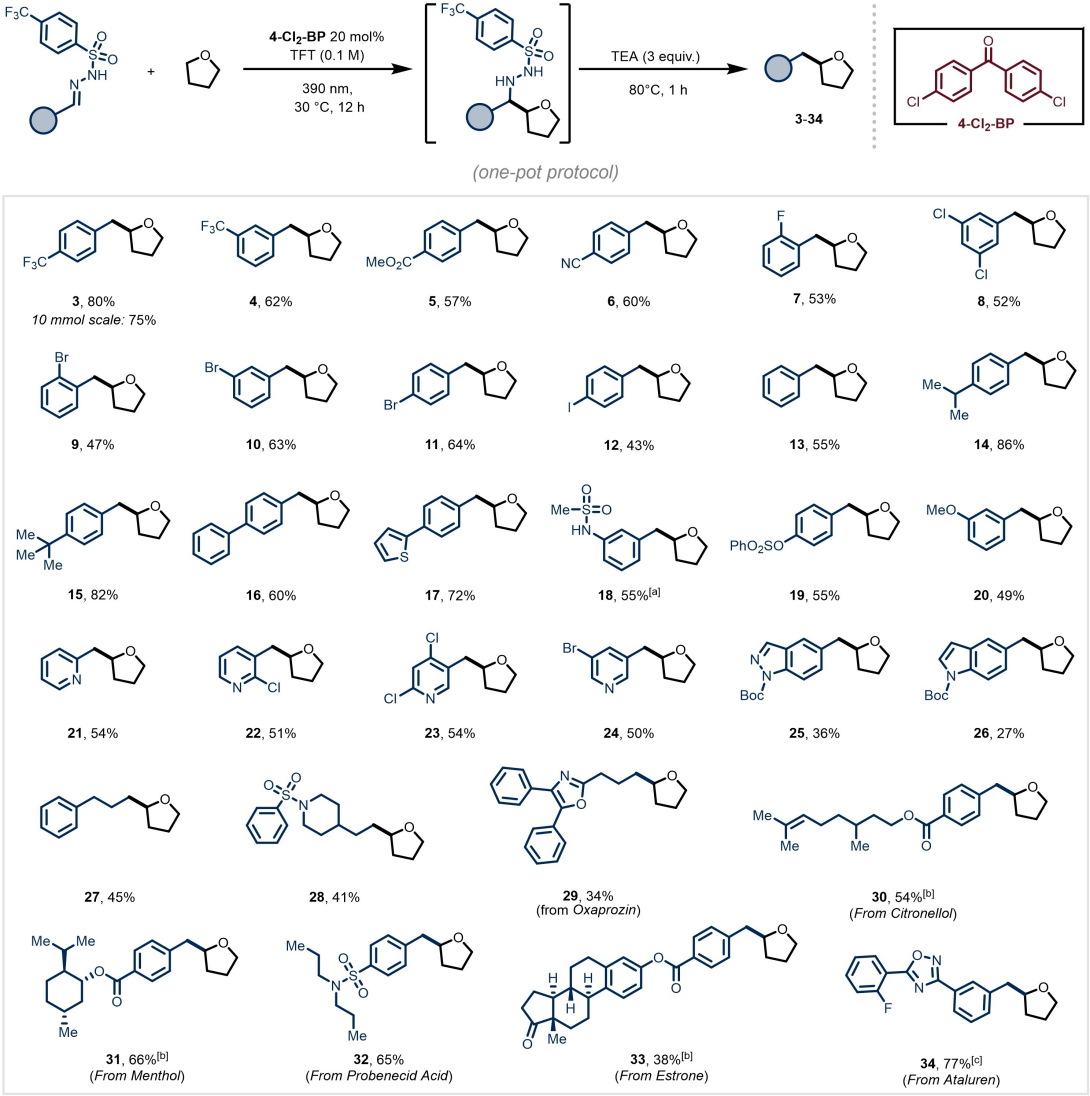
Scope of the alkylation of THF using aromatic and aliphatic aldehyde‐derived sulfonyl hydrazones. For further experimental details see the Supporting Information. Reaction conditions: hydrazone (0.4 mmol, 1 equiv) in 4 mL of a 1 : 1 v : v THF:TFT (0.1 M). [a] Reaction time for the photochemical step: 48 h. [b] 1 : 1 d.r. [c] The photochemical reaction was carried out in THF (4 mL).

Next, we found that a diverse range of heteroaryl aldehydes, such as picolinaldehyde (**21**), nicotinaldehydes derivates (**22**–**24**), indazole (**25**) and indole (**26**) were compatible with our reaction protocol. Also, several aliphatic aldehyde‐derived sulfonyl hydrazones were subjected to our reaction conditions and afforded the targeted C−H alkylated products, albeit with diminished yields (34–45 %, **27**–**29**). To further demonstrate the synthetic value of our method, 4‐trifluoromethyl‐phenylsulfonyl hydrazones of several drugs and natural products, such as probenecid acid (**32**, 65 %), estrone (**33**, 38 %) and ataluren (**34**, 77 %), were successfully engaged as coupling partners.

We subsequently examined the scope of C−H donors in our synthetic process and found that a diverse set of readily available C−H donors were viable for coupling with our model aryl sulfonyl hydrazone **1 a** (Figure 3). Different cyclic and linear ethers were efficiently alkylated to deliver the corresponding homobenzylic ether products in good yields (**35**–**42**, 41–58 %). Interestingly, a masked diol (**39**, 50 %) was successfully alkylated and a formal one‐carbon homologation reaction of 4‐trifluoromethylbenzaldehyde (**40**, 55 %) was accomplished as well.

**Figure 3:**
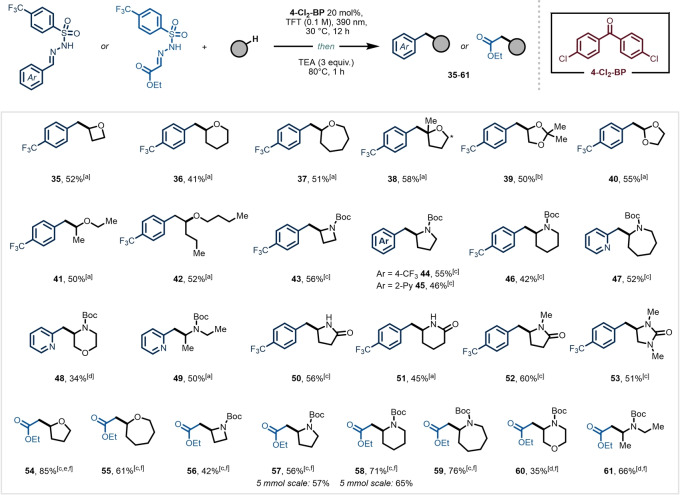
Scope of the alkylation of different C(sp^3^)−H donors using 4‐CF_3_‐benzaldehyde‐, 2‐pyridinecarbaldehyde‐ or ethyl glyoxylate‐derived 4‐trifluoromethyl‐phenyl sulfonyl hydrazones. Reaction conditions: hydrazone (0.4 mmol, 1 equiv) in 4 mL of TFT (0.1 M). For further experimental details see the Supporting Information. [a] 20 equiv of C−H donor used. [b] 1 : 1 *v*/*v* C−H donor:TFT. [c] 5 equiv C−H donor used. [d] 10 equiv of C−H donor used. [e] ^1^H NMR yield using trichloroethylene as external standard due to volatility of 54. [f] Solvent switch to ethanol required for the cleavage step.

Next, a series of cyclic and acyclic protected secondary amines were examined, yielding different medicinally relevant phenyl ethyl amine cores in moderate to good yields (**43**–**49**, 34–56 %). We also found that unprotected and protected cyclic lactams as well as a protected urea were viable substrates under the developed reaction conditions (**50**–**53**, 45–60 %).

After having established this one‐pot C(sp^3^)−H alkylation procedure, we questioned whether the installment of an ester moiety would be feasible through the use of an ethyl glyoxylate‐derived sulfonyl hydrazone **1 aj** (Figure 3, **54**–**61**). For the cleavage step, a solvent switch to ethanol proved to be essential to ensure reproducible results (see Supporting Information, section 5.2). Capitalizing on our two‐step process, we discovered that various aliphatic ethers and amines could be readily coupled, delivering the corresponding products in good yields (**54**–**61**, 35–85 %). Notably, our method provides also immediate access to various protected β‐amino acids, common moieties in many marketed therapeutics (**56**–**61**, 35–76 %).[^54^, ^55^]

Next, we performed some experiments aimed at elucidating the mechanism of this photochemical alkylative process. The involvement of carbon‐centered radicals was confirmed by the presence of adduct **62**, obtained upon addition of 2,2,6,6‐tetramethyl‐1‐piperidinyloxy (TEMPO) as radical scavenger (Figure 4A). The observed kinetic isotope effect (KIE) value of 2.5 indicates that HAT is most likely the rate determining step (Figure 4B).^[59]^ Quantum yield measurements revealed that the reaction is photocatalytic in nature and that a radical chain is either absent or inefficient (*Φ*=0.45, see Supporting Information, section 9.4).^[60]^ From this experimental evidence, a plausible catalytic cycle can be derived as presented in Figure 4C. Upon absorption of a 390 nm photon, the excited state of **4‐Cl_2_‐BP** is responsible for the cleavage of the C(sp^3^)−H bond, thus generating the nucleophilic alkyl radical **63**. This transient species can undergo a polarity‐matched addition to the electrophilic site of **64**, delivering a putative hydrazinyl radical **65**. At this stage, a back‐HAT event from the reduced form of **4‐Cl_2_‐BP** to **65** is envisioned, yielding the Csp^3^−H alkylated product **66** and closing the photocatalytic cycle.

**Figure 4:**
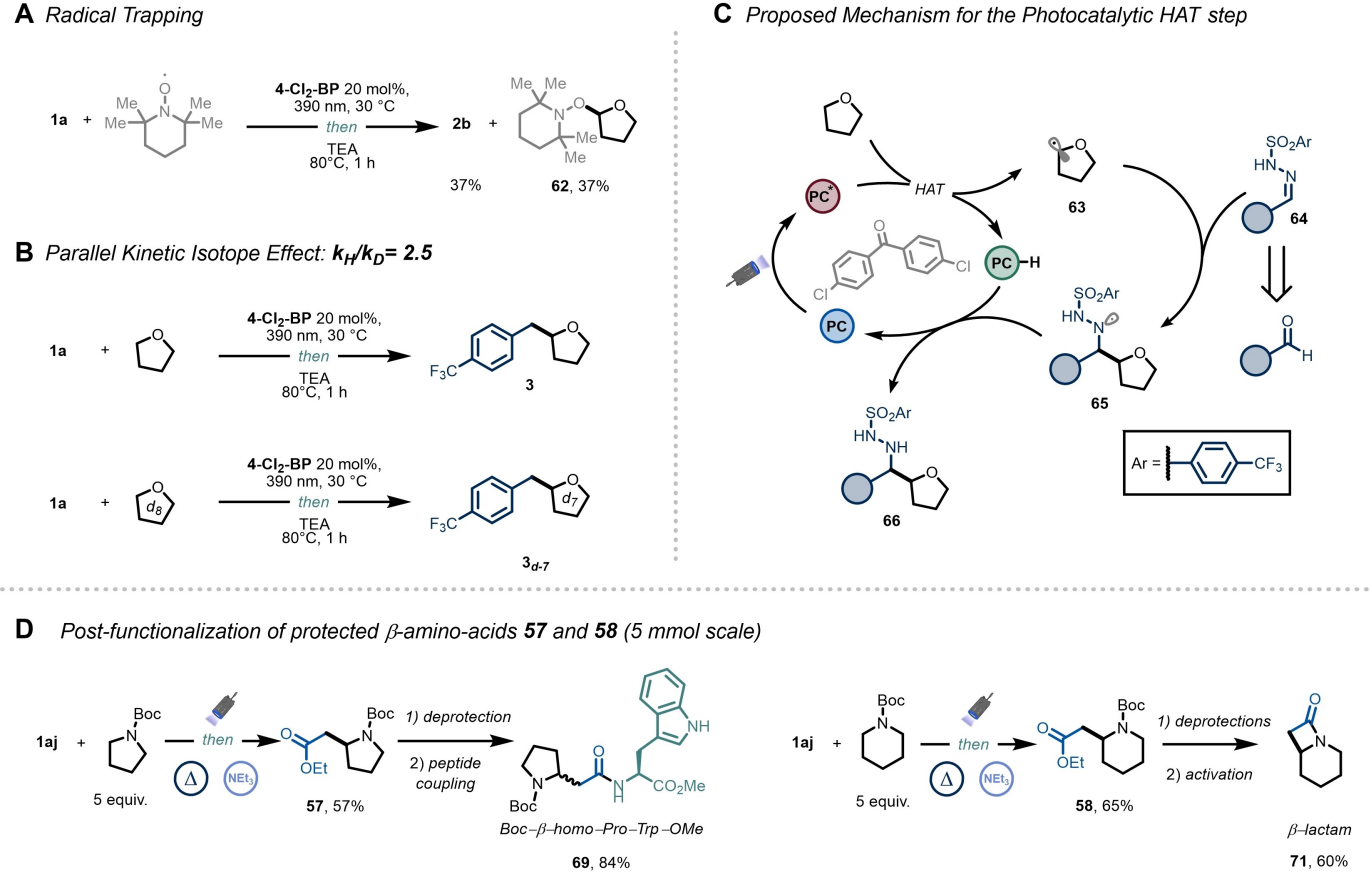
Mechanistic investigation and post‐functionalization (see Supporting Information for experimental details).

Finally, we demonstrated the synthetic potential of ethyl glyoxylate‐derived **1 aj** as a useful denitrogenative ester transfer agent through the post‐functionalization of compounds **57** and **58** on a 5 mmol scale (Figure 4D). Given the high relevance of therapeutic peptides containing β‐amino acids,^[61]^ we showcase the straightforward preparation of dipeptide **69** through a four step sequence, starting from abundant and cheap precursors. Additionally, important motifs in medicinal chemistry, such as β‐lactam **71**,[^62^, ^63^, ^64^] can be prepared through deprotection of **58** and subsequent Mukaiyama reagent‐induced cyclization.^[65]^

In summary, we describe herein an efficient protocol to forge C(sp^3^)−C(sp^3^) bonds from easily accessible *N‐*sulfonyl hydrazones and C(sp^3^)−H donors via a two‐step synthetic strategy, comprising a photocatalytic HAT and a subsequent fragmentation reaction. We demonstrate the utility of this methodology by combining a wide range of hydrazones and C(sp^3^)−H bond‐containing reaction partners, displaying tolerance to many functional groups. The value of this synthetic method was further shown for the preparation of medicinally‐relevant compounds, such as homobenzylic ethers, aryl ethyl amines, and β‐amino acids. Due to its practical and metal‐free nature, we anticipate that this reaction will be of added value to those working in academia and industry. Additional studies to extend the application of this C(sp^3^)−C(sp^3^) bond‐forming strategy to new synthetic contexts are underway and will be reported in due course.

## Conflict of interest

The authors declare no conflict of interest.

## Supporting information

As a service to our authors and readers, this journal provides supporting information supplied by the authors. Such materials are peer reviewed and may be re‐organized for online delivery, but are not copy‐edited or typeset. Technical support issues arising from supporting information (other than missing files) should be addressed to the authors.

Supporting Information

## Data Availability

General information, optimization studies, experimental procedures, mechanistic studies and spectroscopic characterization of the prepared compounds are available in the Supporting Information. In addition, the primary NMR FID files for compounds **3**–**61** and **68**–**71** are available in the FigShare repository at https://www.doi.org/10.6084/m9.figshare.21293955.
