# Supplementary material for: Photocatalytic Alkylation of C(sp3)−H Bonds Using Sulfonylhydrazones
Source: Angew Chem Int Ed Engl. 2022 Dec 13;62(3):e202215374. doi: 10.1002/anie.202215374 (PMC10108173; doi:10.1002/anie.202215374)

Angewandte Chemie
Eine Zeitschrift der Gesellschaft Deutscher Chemiker

Supporting Information

Photocatalytic Alkylation of C(sp^3)–H Bonds Using Sulfonylhydrazones

*A. Pulcinella, S. Bonciolini, F. Lukas, A. Sorato, T. Noël**

Supporting Information

Photocatalytic Alkylation of C(sp^3)−H Bonds Using Sulfonylhydrazones

*Antonio Pulcinella,[1,†] Stefano Bonciolini,[1,†] Florian Lukas,[1] Andrea Sorato,[1] Timothy Noël[1,]**

[1] Flow Chemistry Group, van 't Hoff Institute for Molecular Sciences (HIMS), University of Amsterdam, Science Park 904, 1098 XH Amsterdam, The Netherlands.

* *Email: t.noel@uva.nl*

Table of Contents

1. General information…...S4

2. Chart of sulfonyl hydrazones and synthesized aldehydes… S5

3. Synthesis of Starting Materials….S7

3.1 Synthesis of protected amines…....S7

3.2 Preparation of aldehydes…S7

Synthesis of **S2**….. S7

Synthesis of **S3** and **S4**….. S8

Synthesis of **S7**, **S8** and **S10**….. S8

Synthesis of **S1**…... S10

Synthesis of **S5**…... S10

Synthesis of **S6**, **S9** and **S11**….... S11

3.3 General method for the preparation of arylsulfonyl hydrazines…..S12

3.4 General procedure (GP1) for the preparation of aryl sulfonylhydrazone compounds ***1a-1aj***…..S12

4. Reaction setup… S13

5. Optimization… S14

5.1 Optimization of the first step: radical addition to benzenesulfonyl hydrazones… S14

5.2 Optimization of the second step: fragmentation of the benzenesulfonyl hydrazide…S19

6. General procedure 2 (GP2): photochemical radical addition to benzenesulfonyl hydrazones using THF as the C(sp^3)−H donor and subsequent fragmentation of the benzenesulfonyl hydrazide…..S21

7. General procedure 3 (GP3): photochemical radical addition to benzenesulfonyl hydrazone (**1a**) using C(sp^3)−H donors and subsequent fragmentation of the benzenesulfonyl hydrazide… S21

8. General procedure 4 (GP4): photochemical radical addition to ethyl 2-(2-((4-(trifluoromethyl)phenyl)sulfonyl)hydrazineylidene)acetate (**1aj**) and subsequent fragmentation of the benzenesulfonyl hydrazide….. S22

9. Mechanistic investigation… S23

9.1 UV-Vis characterization…..S23

9.2 Kinetic Isotope Effect (KIE)…S26

Competitive kinetic isotope effect…...S26

Parallel kinetic isotope effect….. S26

9.3 Chemical Quenching…S28

9.4 Quantum yield… S28

10. Scale-up procedure for compounds **3**, **57**, **58**………………………………………….. S31

Scale up for compound **3**……………………………………………………………………S31

Scale up for compounds **57**, **58**…………………………………………………………S32

11. Post-Functionalizations……………………………………………………………………S33

Synthesis of di-peptide **69**……………………………………………………………... S33

Synthesis of β-lactam **71**………………………………………………………………. S33

12. Characterization data of synthesized compound…………………………………………. S34

12.1 Characterization of sulfonyl hydrazones **1a** – **1j**……………………………………S34

12.2 Characterization of hydrazide intermediate **2a**……………………………………S48

12.3 Characterization of compounds **3** – **34**……………………………………………… S49

12.4 Characterization of compounds **35** – **61**…………………………………………….. S60

12.5 Characterization of compounds **68** – **71**…………………………………………….. S70

13. Limitation of the scope…………………………………………………………………S72

14. References………………………………………………………………………………S73

15. NMR spectra of sulfonylhydrazones **1a** – **1aj**………………………………………S76

16. NMR spectra of hydrazide intermediate **2a**…………………………………………….. S148

17. NMR spectra of products **3** – **71**……………………………………………………S149

1. General information

All reagents and solvents were used as received without further purification, unless stated otherwise. Reagents and solvents were bought from Sigma Aldrich, TCI, Fluorochem and Fisher Scientific and, if applicable, kept under argon atmosphere. Technical solvents were bought from VWR International and Biosolve, and were used as received. The catalyst TBADT (tetrabutylammonium decatungstate, (n-$Bu_4N)_4W_{10}O_{32}$) was prepared according to a published procedure.[1] Photocatalyst PC2, PC3, PC4 and PC5 were prepared according to a published procedure.[2] Disposable syringes were purchased from Laboratory Glass Specialist. Product isolation was performed manually, using silica (60, F254, Merck™). TLC analysis was performed using Silica on aluminum foils TLC plates (F254, Supelco Sigma-Aldrich™) with visualization under ultraviolet light (254 nm and 365 nm) or appropriate TLC staining (cerium ammonium molybdate or potassium permanganate). ^{1}H (400 MHz), ^{13}C (101 MHz), ^{19}F NMR (376 MHz) spectra were recorded unless stated otherwise at ambient temperature using a Bruker AV400 or a Bruker AV300. ^{1}H NMR spectra are reported in parts per million (ppm) downfield relative to $CDCl_3$ (7.26 ppm) and all ^{13}C NMR spectra are reported in ppm relative to $CDCl_3$ (77.16 ppm) unless stated otherwise. The following abbreviations have been adopted to describe the multiplicity: bs (broad singlet), s (singlet), d (doublet), t (triplet), q (quartet), p (pentet), h (hextet), hept (heptet), m (multiplet), dd (double doublet), td (triple doublet), tt (triplet of triplets). Coupling constants (J) are reported in hertz (Hz). NMR data were processed using the MestReNova 14.1.0 software package. Known products were characterized through comparison with the corresponding ^{1}H NMR and ^{13}C NMR from literature. High resolution mass spectra (HRMS) were collected on an AccuTOF LC, JMS-T100LP Mass spectrometer (JEOL, Japen. UV-Vis spectra were recorded with a double beam spectrophotometer Shimadzu UV2700 equipped with a deuterium lamp (190-350 nm), a halogen lamp (330-900 nm) and a photomultiplier (Hamamatsu R928). The diastereomeric ratios of compounds **30**, **33** were determined by HPLC analysis on chiral stationary phase. HPLC analysis were performed on a Shimadzu apparatus equipped with a diode array detector (DAD) and column temperature control module. Liquid chromatograph (LC-20AD); Autosampler (SIL-20A); Diode Array detector (SPD-M20A); Column oven (CTO-20AC); Degasser (DGU-$20A_5$). The names of all products were generated using the PerkinElmer ChemBioDraw Ultra v.12.0.2 software package.

For the photochemical batch experiments, a 3D-printed (PLA) reactor internally coated with aluminum foil and equipped with a specific 3D-printed (PLA) lid serving as vials holder and lamp holder was used (see section 4 for details).

2. Chart of sulfonyl hydrazones and synthesized aldehydes

Hydrazones tested in the optimization

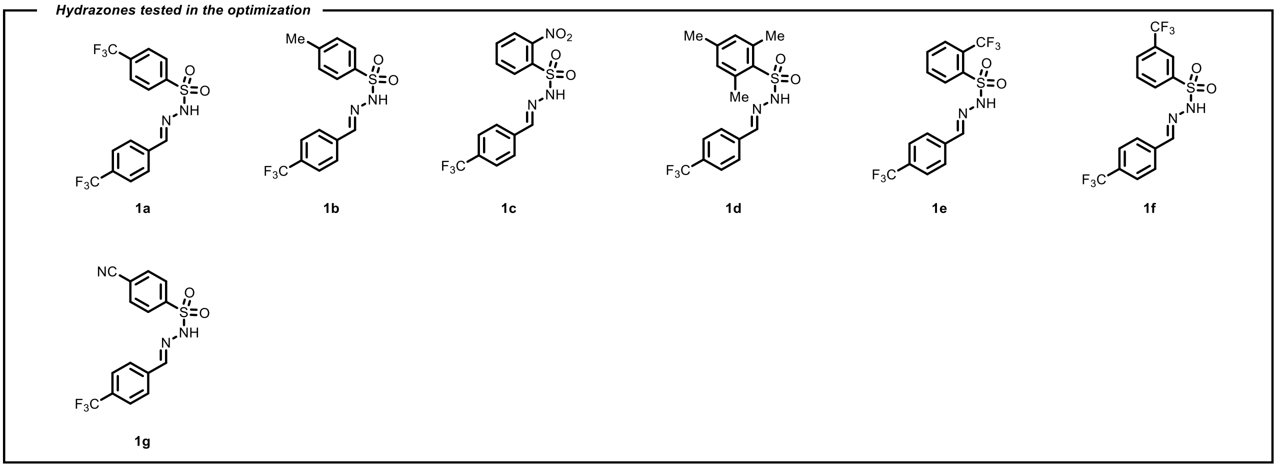

Hydrazones used in the scope

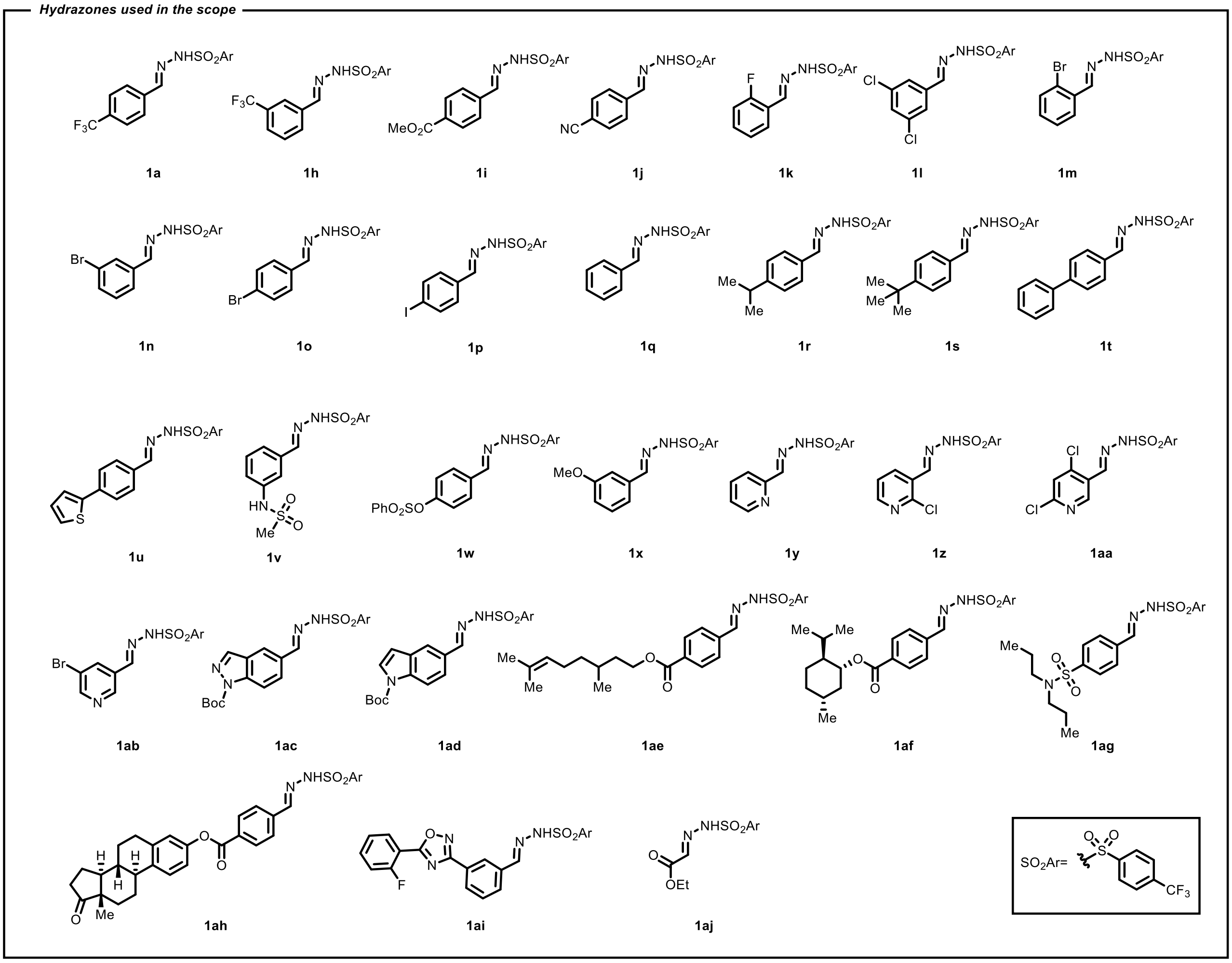

Synthesized Aldehydes

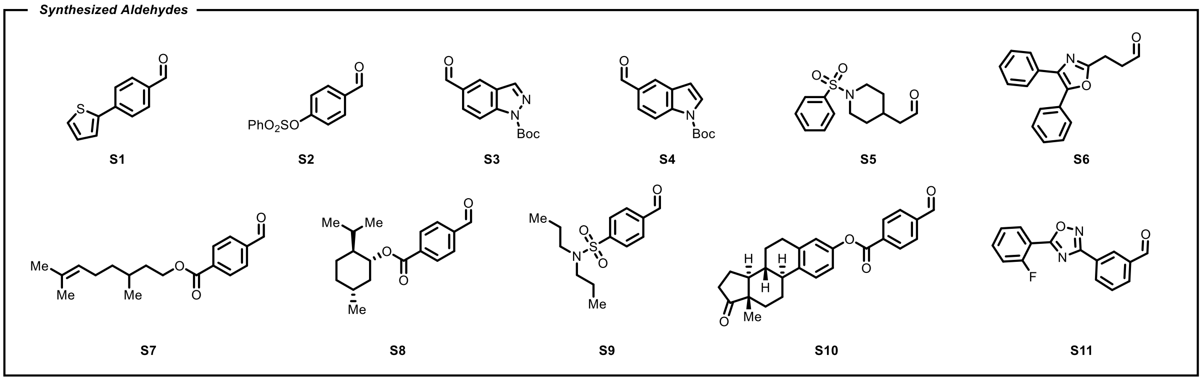

3. Synthesis of Starting Materials

3.1 Synthesis of protected amines.

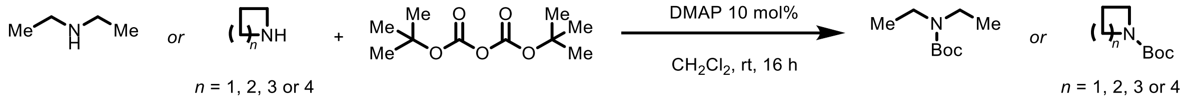

The protected amines shown above were synthesized adapting a procedure reported in the literature.[3]
In particular, Boc_2O (2.4 g, 11 mmol, 1.1 equiv) was dissolved in CH_2Cl_2 in an oven-dried vial under inert atmosphere. In the meantime, in a 100 mL round-bottom flask, 4-DMAP (122 mg, 1 mmol, 10 mol%) together with azetidine (674 µL, 10 mmol, ρ = 0.847 g mL^{-1}), pyrrolidine (821 µL, 10 mmol, ρ = 0.866 g mL^{-1}), piperidine (988 µL, 10 mmol, ρ = 0.862 g mL^{-1}) or diethyl amine (1.02 mL, 10 mmol, ρ = 0.717 g mL^{-1}) were dissolved in 30 mL of CH_2Cl_2. The solution was placed in an ice bath and the Boc_2O solution was added dropwise via a syringe. The resulting solution was stirred at room temperature for 16 hours. Then, was quenched with water and the organic phase was washed with a solution of HCl 0.3 M (2x25 mL) and once with brine (25 mL). The resulting organic phase was then dried over Na_2SO_4 and the solvent was removed under reduced pressure affording the desired protected amines.
The spectroscopic data are in accordance with those reported in the literature.[3]
tert-butyl azetidine-1-carboxylate, colorless liquid, 64%: **^{1}H NMR** (400 MHz, $CDCl_3$): δ 3.95 (t, 4H), 2.18 (p, 2H), 1.45 (s, 9H).[4]
tert-butyl pyrrolidine-1-carboxylate, colorless liquid, 72%: **^{1}H NMR** (400 MHz, CDC13): δ 3.30-3.28 (m, 4H), 1.86-1.74 (m, 4H), 1.45 (s, 9H).[5]
tert-butyl piperidine-1-carboxylate, colorless liquid, 69%: **^{1}H NMR** (400 MHz, $CDCl_3$): δ 3.37 (t, 4H), 1.63-1.49 (m, 6H), 1.47 (s, 9H).[5]
tert-butyl azepane-1-carboxylate, colorless liquid, 75% : **^{1}H NMR** (300 MHz, $CDCl_3$) δ 3.44 – 3.27 (m, 4H), 1.73 – 1.62 (m, 4H), 1.53 (dd, 4H), 1.45 (s, 9H).
tert-butyl diethylcarbamate, colorless liquid, 80%: **^{1}H NMR** (400 MHz, $CDCl_3$) δ 3.19 – 2.99 (m, 4H), 1.37 (s, 9H), 1.01 (t, 6H).

3.2 Preparation of aldehydes

*Synthesis of **S2**.*

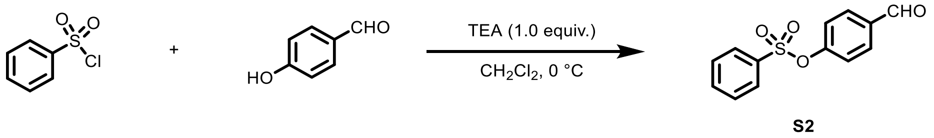

S2

Compound **S2** was synthesized using a procedure reported in the literature.[6]

To a 0 °C solution of 4-hydroxybenzaldehyde (1.83 g, 15.0 mmol) in dichloromethane (28 mL) benzenesulfonyl chloride (2.65 g, 1.91 mL, 15.0 mmol) was added followed by dropwise addition of triethylamine (1.52 g, 2.10 mL, 15.0 mmol). Then, the homogeneous solution was removed from the ice bath and stirred at room temperature for 2 h. The reaction mixture was washed with 1 M HCl (3 x) followed by saturated aqueous $NaHCO_3$ (2 x) and finally with brine. The combined organic layers were dried over MgSO4, filtered, and concentrated under reduced pressure to obtain the product as a white solid (3.54 g, 13.5 mmol, 90%).

Spectroscopic data for compound **S2** are in accordance with those reported in literature.[6]
^{1}H NMR (300 MHz, $CDCl_3$) δ 9.96 (s, 1H), 7.90 – 7.76 (m, 4H), 7.75 – 7.63 (m, 1H), 7.61 – 7.48 (m, 2H), 7.22 – 7.11 (m, 2H).

*Synthesis of **S3** and **S4**.*

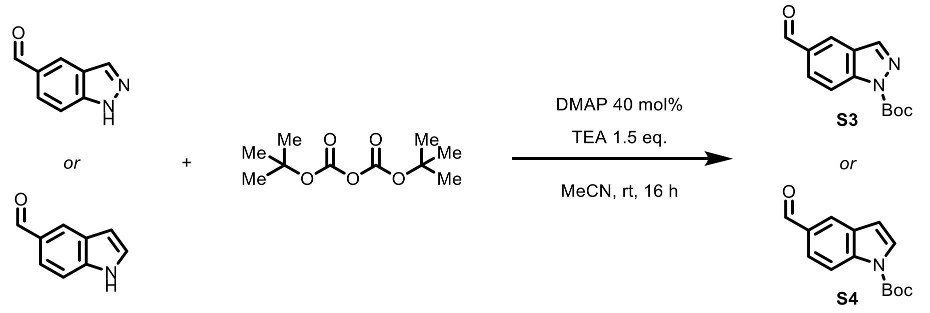

Compounds **S3** and **S4** were synthesized adapting a procedure reported in the literature.[7]
To a solution of starting material (2.0 mmol, 1.0 equiv.) in 15mL MeCN was added under nitrogen Boc-anhydride (458 mg, 2.1 mmol, 1.1 equiv.), DMAP (98 mg, 0.8 mmol, 0.4 equiv.), and triethylamine (304 mg, 418 µL, 3.0 mmol, 1.5 equiv.). After stirring overnight, the solvent was evaporated. The residue was dissolved in CH_2Cl_2 and washed with saturated sodium bicarbonate solution (aq.) and brine. The organic layer was dried over sodium sulfate and the solvent evaporated under reduced pressure. The crude product was purified via flash chromatography, eluting with 5:1 Hexane:Ethyl Acetate, to give a white solid. Yields: **S3** (365 mg, 1.48 mmol, 74%), **S4** (402 mg, 1.64 mmol, 82%).

Spectroscopic data for compounds **S3** and **S4** are in accordance with those reported in literature.
S3: ^{1}H NMR (400 MHz, CDC13) δ = 10.15 (s, 1H), 8.71 (d, J = 1 Hz, 1H), 8.25 (d, J = 1 Hz, 1H), 7.86 (d, J = 1 Hz, 2H), 1.75 (s, 9H).[8]
^{13}C NMR (101 MHz, $CDCl_3$) δ = 192.1, 149.0, 139.7, 139.4, 136.6, 129.4, 123.0, 122.0, 118.6, 86.0, 28.2.
S4: ^{1}H NMR (400 MHz, $CDCl_3$) δ = 10.05 (s, 1H), 8.28 (d, J = 9 Hz, 1H), 8.08 (d, J = 2 Hz, 1H), 7.85 (dd, J = 9, 2 Hz, 1H), 7.68 (d, J = 4 Hz, 1H), 6.68 (d, J = 4 Hz, 1H), 1.69 (s, 9H).[9]
^{13}C NMR (101 MHz, $CDCl_3$) δ = 192.3, 149.4, 138.9, 131.8, 130.8, 127.8, 125.3, 124.4, 115.7, 108.0, 84.8, 28.3.

*Synthesis of **S7**, **S8** and **S10**.*

Compounds **S7** and **S8** were synthesized adapting a procedure reported in the literature.[10]

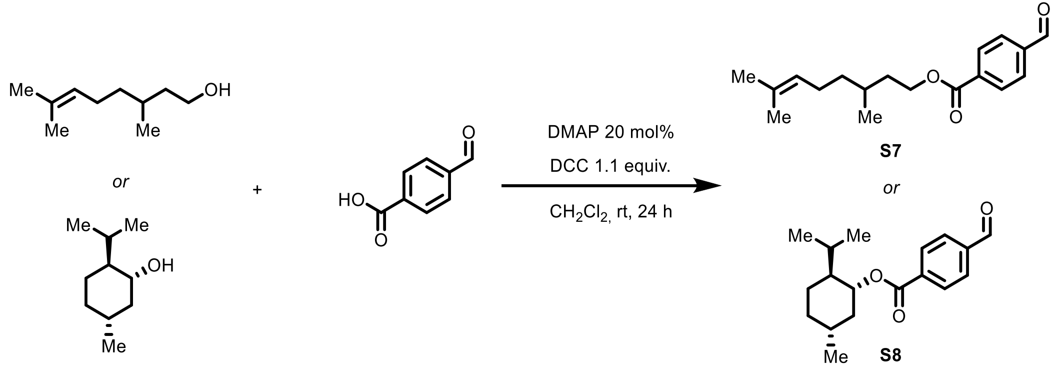

To a suspension of 4-formylbenzoic acid (450 mg, 3.0 mmol, 1.0 equiv.) in dichloromethane (10 mL) were added N,N-dimethyl-4-aminopyridine (73 mg, 0.60 mmol, 0.20 equiv.), the corresponding alcohol (3.9 mmol, 1.3 equiv.) and N,N'-dicyclohexylcarbodiimide (681 mg, 3.3 mmol, 1.1 equiv.). The mixture was stirred at room temperature for 24 h then diethyl ether (15 mL) was added. The precipitate was filtered through celite and the filtrate was successively washed with a 1 M aqueous solution of HCl (2 × 15 mL), saturated sodium bicarbonate solution (aq.), (2 × 15 mL) and brine (15 mL). The organic phase was dried over sodium sulfate, filtered and concentrated under reduced pressure. The crude product was purified by flash chromatography on silica gel, eluting with Diethyl Ether:Pentane 1:10 to afford the product as a colorless oil. Yields: **S7** (580 mg, 2.0 mmol, 67%), **S8** (707 mg, 2.45 mmol, 82%).

Spectroscopic data for compounds **S7** and **S8** are in accordance with those reported in literature.[11]
S7: **^{1}H NMR** (300 MHz, $CDCl_3$) δ 10.11 (s, 1H), 8.23 – 8.16 (m, 2H), 7.98 – 7.92 (m, 2H), 5.09 (tdd, *J* = 6, 3, 1 Hz, 1H), 4.40 (td, *J* = 6, 3 Hz, 2H), 2.02 (p, *J* = 7 Hz, 2H), 1.89 – 1.77 (m, 1H), 1.67 (q, *J* = 1 Hz, 3H), 1.60 (d, *J* = 1 Hz, 3H), 1.55 (s, 2H), 1.48 – 1.34 (m, 1H), 1.32 – 1.18 (m, 1H), 0.98 (d, *J* = 6 Hz, 3H).
S8: **^{1}H NMR** (300 MHz, $CDCl_3$) δ 10.10 (s, 1H), 8.22 – 8.15 (m, 2H), 7.99 – 7.91 (m, 2H), 4.97 (td, *J*=11, 4 Hz, 1H), 2.13 (dd, *J* = 12, 5 Hz, 1H), 1.94 (pd, *J* = 7, 3 Hz, 1H), 1.81 – 1.68 (m, 2H), 1.66 – 1.50 (m, 2H), 1.30 – 1.04 (m, 2H), 1.00 (s, 1H), 0.93 (dd, *J* = 7, 4 Hz, 6H), 0.80 (d, *J* = 7 Hz, 3H).

Compound **S10** was synthesized using a procedure reported in the literature.[12]

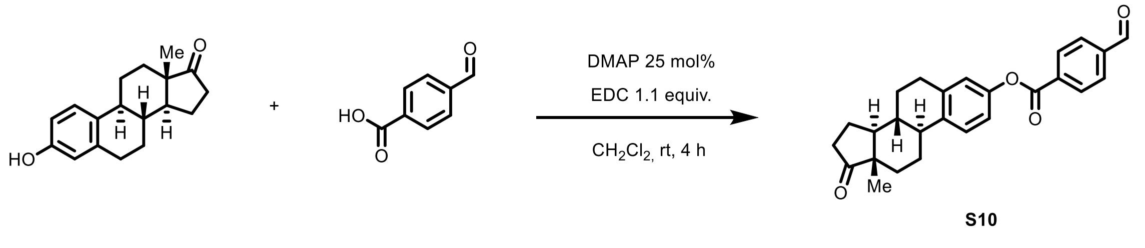

To a round-bottom flask equipped with formyl-benzoic acid (450 mg, 3.0 mmol, 1.0 equiv.) was added estrone (811 mg, 3.0 mmol, 1.0 equiv.), 1-(3-dimethylaminopropyl)-3-ethylcarbodiimide hydrochloride (633 mg, 3.3 mmol, 1.1 equiv.), N, N-dimethyl-4-aminopyridine (92 mg, 0.75 mmol, 0.25 equiv.), and CH_2Cl_2 (10 mL). After stirring at room temperature for 4 h, the reaction was quenched with 10 mL of saturated sodium bicarbonate solution (aq.) and extracted with CH_2Cl_2 (3 × 5 mL). The combined organic layers were dried over sodium sulfate, filtrated, and concentrated under reduced pressure. The crude product was purified by flash chromatography on silica gel (from Pentane:Ethyl Acetate 5:1 to 1:1) to afford product **S10** as a white solid (1.0 g, 2.5 mmol, 83%).

Spectroscopic data for compound **S10** is in accordance with those reported in literature.[13]
S10 : **^{1}H NMR** (300 MHz, $CDCl_3$) δ = 10.15 (s, 1H), 8.41 – 8.31 (m, 2H), 8.06 – 7.94 (m, 2H), 7.36 (d, *J* = 8 Hz, 1H), 7.03 – 6.93 (m, 2H), 2.95 (dd, *J* = 8, 4 Hz, 2H), 2.58 – 1.92 (m, 7H), 1.75 – 1.41 (m, 6H), 0.93 (s, 3H).

*Synthesis of **S1**.*

S1 was synthesized adapting a procedure reported in the literature.[14]

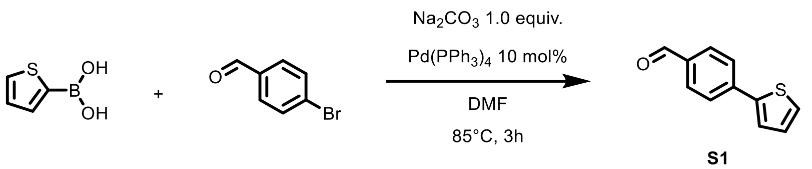

4-bromobenzaldehyde (277 mg, 1.5 mmol, 1.0 equiv.) and thiophene boronic acid (211 mg, 1.65 mmol, 1.1 equiv.) were suspended in a solution of DMF (30 mL) and sodium carbonate (159 mg, 1.5 mmol, 1.0 equiv.). The atmosphere was exchanged to nitrogen three times using Schlenk techniques. To this mixture $Pd(PPh_3)_4$ (173 mg, 0.15 mmol, 0.10 equiv.) was added under nitrogen and the mixture was degassed for 2 minutes. The mixture was then heated at 85 °C for 3 hours. The reaction mixture was combined with HCl (100 mL of a 1M aqueous solution) and ethyl acetate (200 mL). The organic layer was separated, washed sequentially with HCl (50 mL of a 1M aqueous solution) and brine (50 mL), and dried over sodium sulfate. After filtration the solvent was removed under reduced pressure. The desired product was obtained by column chromatography on silica gel (263 mg, 1.4 mmol, 91%).

Spectroscopic data are in accordance with those reported in the literature.[15]
S1: **^{1}H NMR** (300 MHz, $CDCl_3$) δ 9.93 (s, 1H), 7.84 – 7.76 (m, 2H), 7.71 – 7.61 (m, 2H), 7.38 (dd, *J* = 4, 1, 1H), 7.33 (dd, *J* = 5, 1, 1H), 7.07 (dd, *J* = 5, 4, 1H).

*Synthesis of **S5**.*

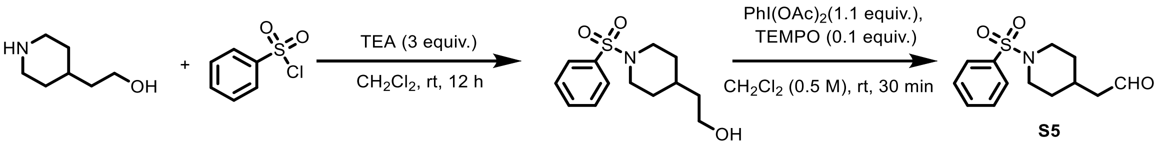

S5 was synthesized adapting two procedures reported in the literature.
A solution of sulfonyl chloride (3.18 g, 18.0 mmol) in CH_2Cl_2 (20 mL) was added dropwise to a stirred solution of diethanolamine (2.56 g, 19.8 mmol) in triethylamine (5.46 g, 7.53 mL, 54.0 mmol) at room temperature and the resulting mixture was stirred at room temperature for 12 h. After, water (10 mL) was added and the aqueous phase was extracted with CH_2Cl_2. The combined organic layer was dried over Na_2SO_4, filtered and the solvent was removed under reduced pressure to afford the protected aminoalchol that was used in the next step without any further purification.[16]

Next, in a 100 mL round bottom flask, the crude product was dissolved in 35 mL of CH_2Cl_2 (0.5 M). Subsequently, iodobenzene diacetate (6.37g, 19.8 mmol, 1.05 equiv.) and TEMPO (283 mg, 1.80 mmol, 0.1 equiv). were added and the mixture was stirred at room temperature for 12 h. After, the reaction was quenched by the addition of a 1.0 M aqueous solution of $Na_2S_2O_3$ and the aqueous phase was extracted with

CH_2Cl_2. The combined organic layer was dried over Na_2SO_4, filtered and the solvent was removed under reduced pressure. The crude was purified via flash column chromatography (SiO_2, Hexane: Ethyl Acetate 2:1) affording aldehyde **S5** as a transparent wax (3.9 g, 14.6 mmol, 81% yield).[17]

^{1}H NMR (400 MHz, $CDCl_3$) δ 9.70 (s, 1H), 7.76 – 7.72 (m, 2H), 7.61 – 7.56 (m, 1H), 7.55 – 7.50 (m, 2H), 3.76 (d, *J* = 12 Hz, 2H), 2.36 (d, *J* = 7 Hz, 2H), 2.28 (td, *J* = 12, 2 Hz, 2H), 1.88 – 1.67 (m, 3H), 1.35 (qd, *J* = 12, 4 Hz, 2H).
^{13}C NMR (101 MHz, $CDCl_3$) δ 201.0, 136.1, 132.8, 129.1, 127.7, 49.9, 46.3, 31.3, 29.7.
HRMS (ESI+) (m/z): $[M+H]^+]^+$ calcd. for $C_{13}H_{17}NO_3S$, 268.1006; found: 268.1007.

Synthesis of **S6**, **S9** *and* **S11**.

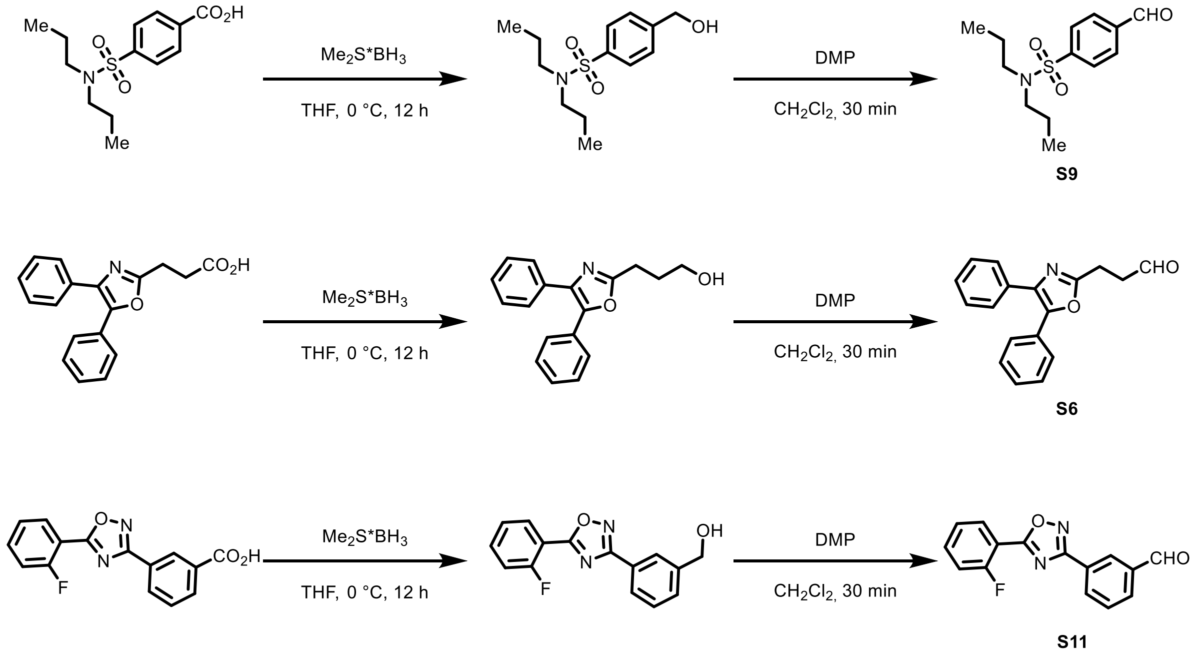

S6, S9 and **S11** were synthesized adapting a procedure reported in literature.[18]
A round bottom flask was charged with the carboxylic acid (5.0 mmol, 1.0 equiv.), dry THF and BH_3•Me_2S (12.5 mmol, 2.5 equiv.) was added dropwise at 0 °C under nitrogen atmosphere. The mixture was stirred at room temperature for 12 hours and then quenched with MeOH, washed with saturated NH_4Cl and extracted with ethyl acetate, dried with Na_2SO_4 and filtered. The filtrate was concentrated under reduced pressure to give the corresponding alcohol. The crude product was used without purification in the next step. The alcohol (5.0 mmol, 1.0 equiv.) was dissolved in CH_2Cl_2 and Dess-Martin periodinane (15 mmol, 3.0 equiv.) was added portionwise. The mixture was stirred at room temperature for about 1 hour. Then the mixture was washed with saturated $NaHSO_3$ and extracted with ethyl acetate, dried with Na_2SO_4 and filtered. The filtrate was concentrated under reduced pressure. The residue was purified by flash column chromatography on silica gel to afford the corresponding aldehyde. Yield over 2 steps: **S9** 60% **S6** 72% **S11** 33%.

Spectroscopic data for compounds **S6** and **S9** are in accordance with those reported in the literature.[18]
S9: ^{1}H NMR (300 MHz, $CDCl_3$) δ 10.09 (s, 1H), 8.03 – 7.94 (m, 4H), 3.17 – 3.05 (m, 4H), 1.54 (dq, *J* = 15, 7 Hz, 4H), 0.86 (t, *J* = 7 Hz, 6H).

S6: ^{1}**H NMR** (400 MHz, $CDCl_3$) δ 9.71 (t, *J* = 2 Hz, 1H), 7.73 – 7.61 (m, 2H), 7.53 – 7.41 (m, 2H), 6.91 – 6.81 (m, 2H), 6.69 (dd, *J* = 9, 3 Hz, 1H), 3.83 (s, 3H), 3.73 (d, *J* = 2 Hz, 2H), 2.39 (s, 3H).[19]
S11: ^{1}**H NMR** (400 MHz, $CDCl_3$) δ 10.13 (s, 1H), 8.68 (d, *J* = 2 Hz, 1H), 8.45 (dt, *J* = 7, 1 Hz, 1H), 8.24 (td, *J* = 7, 2 Hz, 1H), 8.06 (dt, *J* = 8, 1 Hz, 1H), 7.70 (t, *J* = 8 Hz, 1H), 7.66 – 7.59 (m, 1H), 7.36 (t, *J* = 8 Hz, 1H), 7.33 – 7.28 (m, 1H).
^{13}**C NMR** (101 MHz, $CDCl_3$) δ 191.7, 173.3 (d, *J* = 4 Hz), 167.9, 161.0 (d, *J* = 261 Hz), 137.1, 135.0 (d, *J* = 9 Hz), 133.2, 131.6, 129.8, 129.5, 128.1, 124.9 (d, *J* = 4 Hz), 117.4 (d, *J* = 21 Hz), 112.7 (d, *J* = 11 Hz).
^{19}**F NMR** (282 MHz, $CDCl_3$) δ -108.10.
HRMS (EI+) m/z $[M]^+$ calcd. for $C_{15}H_9F_1N_2O_2$ 268.0648, found 268.0645.

3.3 General method for the preparation of arylsulfonyl hydrazines

Aryl sulfonylhydrazines were synthesized using a procedure reported in the literature.[20]

Hydrazine hydrate (3 equiv.) was added dropwise to a solution of sulfonyl chloride (6 mmol) in 30 mL of THF at 0°C and stirred for 30 minutes at the same temperature. The reaction mixture was diluted with ethyl acetate and washed five times with brine. The organic layer was dried over Na_2SO_4, filtered and the solvent was removed under reduced pressure to afford the desired benzenesulfonyl hydrazine that was used without any further purification for the next step.

3.4 General procedure (GP1) for the preparation of aryl sulfonylhydrazone compounds *1a-1aj*

Aryl sulfonylhydrazones ***1a-1aj*** *were synthesized adapting a procedure reported in the literature.*[21]

To a suspension of aryl sulfonylhydrazide (1.0 equiv.) in ethanol (0.8 M), the corresponding aldehyde (1.0 equiv.) was added portionwise and the reaction mixture was stirred at room temperature for 3-16 hours. In case a solid crashed out of the solution, it was filtered, washed with n-hexane and dried to afford the desired aryl sulfonylhydrazone. For those compounds that did not precipitate, the solvent was removed under reduced pressure and the obtained solid was washed with n-hexane, filtered and dried to afford the desired aryl sulfonylhydrazone.

N.B. Aryl Sulfonylhydrazones prepared from the corresponding aliphatic aldehydes were used without any further purification.

4. Reaction setup

4 vials photoreactor (UFO reactor)

Four reaction were irradiating simultaneously using the photoreactor described below. A 40W Kessil PR160L-390 nm was used as LED lamp, while the temperature was maintained below 30 °C via a fan positioned under the reactor. The assembled set-up was placed behind UV-light shielding amber acrylic for all duration of the reaction.

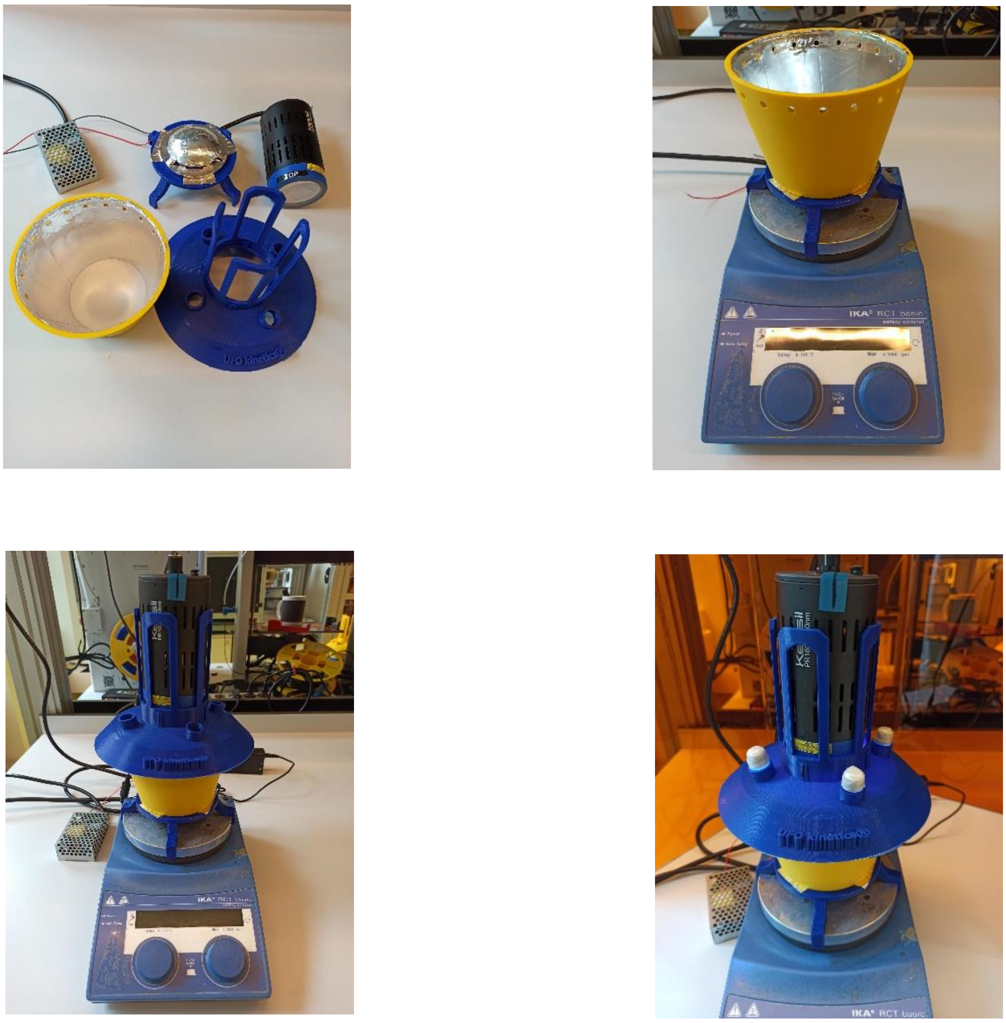

Figure S1. Photoreactor set-up used for optimization and scope

5. Optimization

5.1 Optimization of the first step: radical addition to benzenesulfonyl hydrazones

Table S1. Photocatalysts screening.

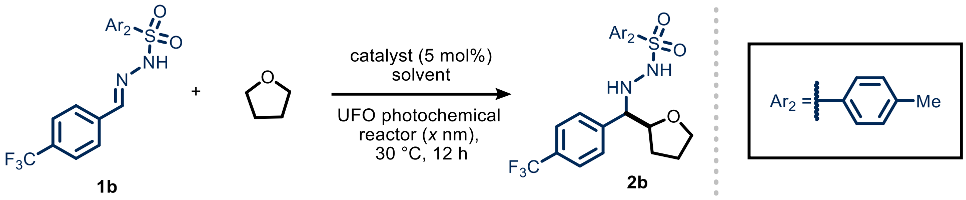

solvent	catalyst	wavelenght	conversion	Yield of 2a[a]
MeCN	PC1[b]	390 nm	100%	35%
THF[c]	PC1[b]	390 nm	100%	82%
MeCN	AQ	390 nm	100%	15%
MeCN	PT	390 nm	100%	10%
DCM	PT	390 nm	100%	n.d.
MeCN	PT	456 nm	100%	n.d.
DCM	PT	456 nm	100%	n.d.
MeCN	TBADT	390 nm	100%	n.d

Reaction conditions: **1b** (0.2 mmols, 1 equiv.), tetrahydrofuran (20 equiv.) in 2 mL of the indicated solvent (0.1 M). [a]Determined via ^{1}H NMR using tricloroethylene as external standard. [b]30 mol% of catalyst used. [c]THF used as solvent.

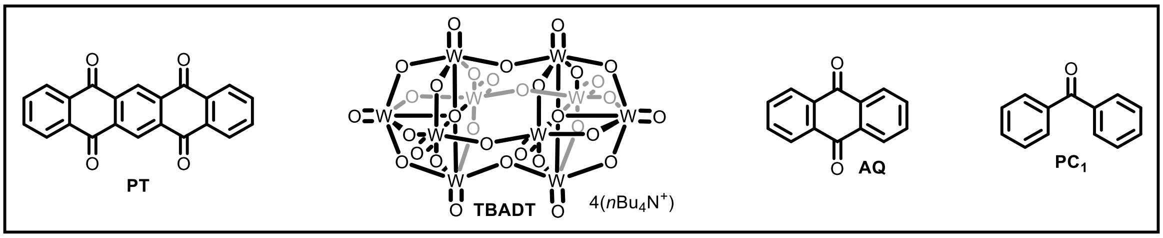

Table S2. Photocatalysts loading.

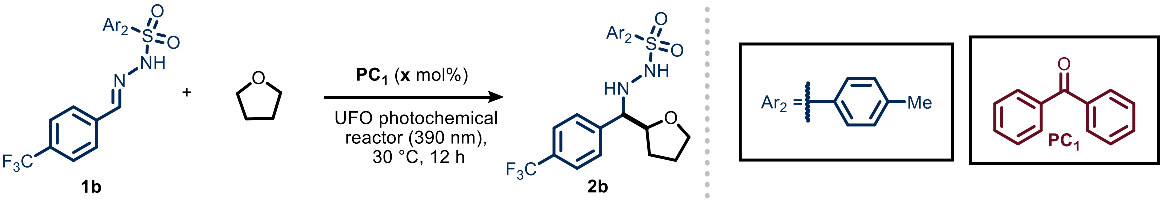

solvent	(**x** mol%)	conversion	Yield of **2b**[a]
THF	10 mol%	100%	55%
THF	20 mol%	100%	75%
THF	30 mol%	100%	82%

Reaction conditions: **1b** (0.2 mmols, 1 equiv.) in 2 mL of dry THF (0.1 M).
[a]Determined via ^{1}H NMR using tricloroethylene as external standard.

Table S3. Co-Solvent screening.

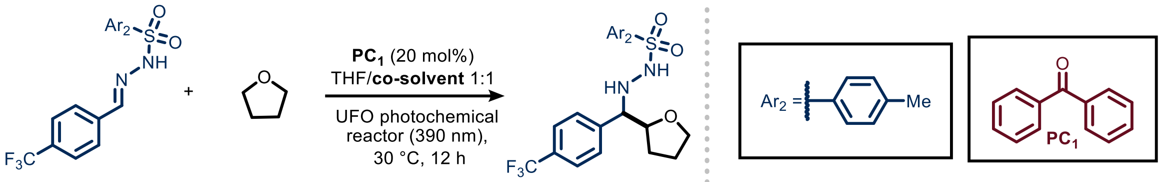

1b **2b**

co-solvent	conversion	Yield of **2b**[a]
Benzene	100%	75%
TFT	100%	27%[b]
TFT	100%	75%[c]
TFT	100%	80%
DCM	100%	69%

Reaction conditions: **1b** (0.2 mmols, 1 equiv.) in 2 mL of a 1:1 *v:v* THF:cosolvent solution (0.1 M). [a]Determined via ^{1}H NMR using tricloroethylene as external standard. [b]10 equiv. of THF used in TFT (0.1 M). [c]20 equiv. of THF used in TFT (0.1 M).

Table S4. Solvent screening (20 equiv. THF).

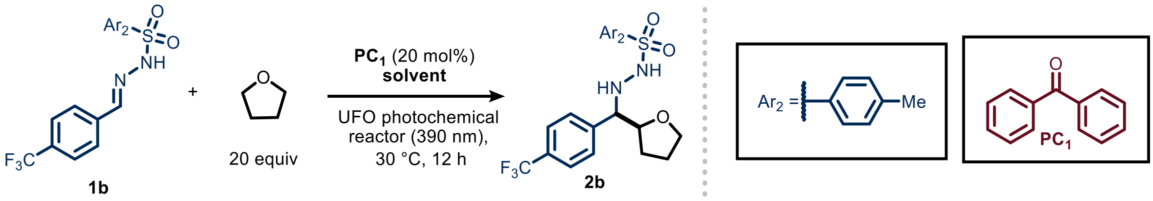

1b **2b**

solvent	conversion	Yield of **2b**[a]
Benzene	100%	55%
TFT	100%	75%
DCM	100%	30%
t-BuOH	100%	45%
HFIP	100%	n.d
Acetone	100%	33%

Reaction conditions:1**b** (0.2 mmols, 1 equiv.), tetrahydrofuran (20 equiv.) in 2 mL of the indicated solvent (0.1 M).
[a]Determined via ^{1}H NMR using tricloroethylene as external standard

Table S5. Second round of photocatalysts screening

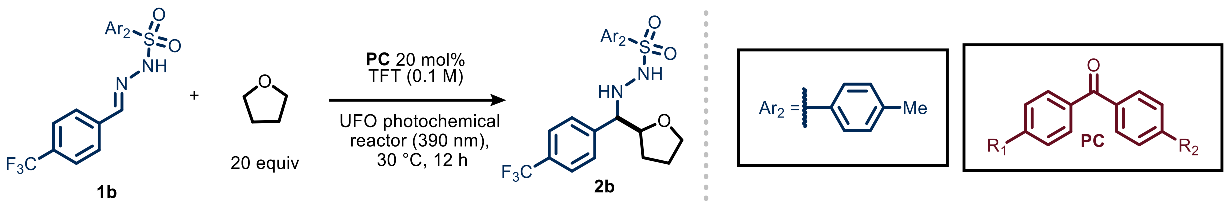

R_1	R_2	PC	conversion	Yield of **2b**[a]
H	H	PC_1	100%	75%
OMe	OMe	PC_2	100%	n.d
OMe	CF_3	PC_3	100%	45%
CF_3	CF_3	PC_4	100%	24%
Cl	Cl	PC_5	100%	78%

Reaction conditions:1**b** (0.2 mmols, 1 equiv.), tetrahydrofuran (20 equiv.) in 2 mL of TFT (0.1 M).
[a]Determined via ^{1}H NMR using tricloroethylene as external standard

Table S6. Electronic properties of the benzenesulfonyl group

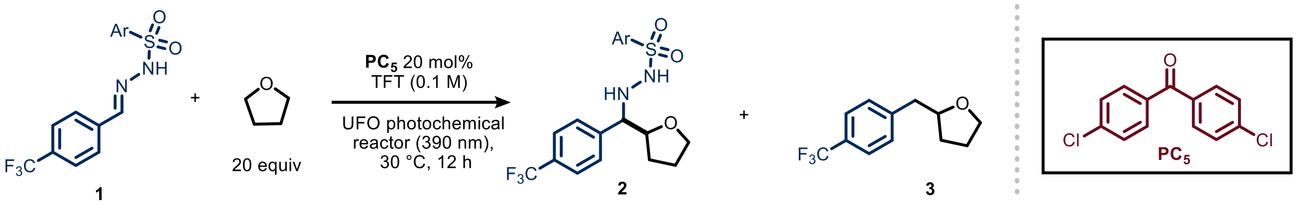

Ar	conversion	Yield of **2**[a]	Yield of **3**[a]
Ar_2	100%	78%	n.d
Ar_4	100%	25%	n.d
Ar_1	100%	90%	n.d
Ar_6	100%	75%	n.d
Ar_5	70% (100%)	n.d	35% (50%, 48h)
Ar_3	100%	n.d	n.d
Ar_7	100%	n.d	n.d

Reaction conditions:1**b** (0.2 mmols, 1 equiv.), tetrahydrofuran (20 equiv.) in 2 mL of TFT (0.1 M).
[a]Determined via ^{1}H NMR using tricloroethylene as external standard

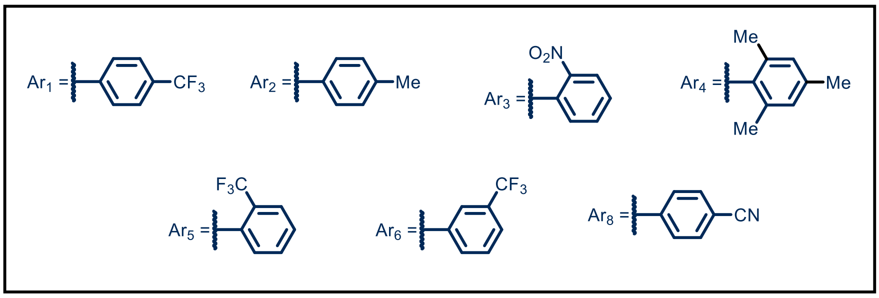

Table S7. Control experiments

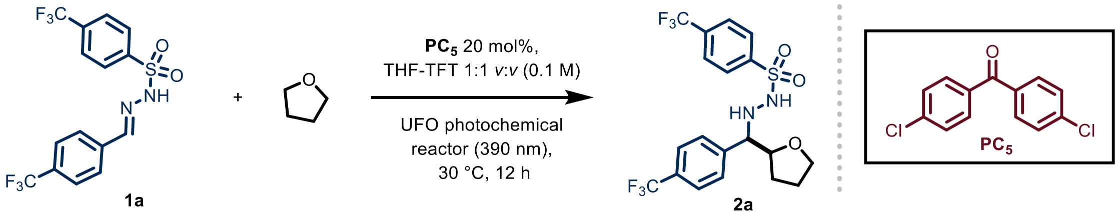

Deviation	Recovery of **1a**[a]	Yield of **2b**[a]
none	-	91%
one pot protocol[b]	-	70%
dark	98%	-
dark, 80 °C	98%	-
no catalyst	95%	-
technical grade THF	-	69%
no degassing	-	64%
10 equiv. H_2O	-	65%

Reaction conditions:**1b** (0.2 mmols, 1 equiv.) in 2 mL of a 1:1 *v:v* THF:cosolvent solution (0.1 M).
[a]Determined via ^{1}H NMR using tricloroethylene as external standard.
[b]4-Trifluoromethylbenzaldehyde (1.0 equiv.) and sulfonyl hydrazine (1.0 equiv.) were used.

5.2 Optimization of the second step: fragmentation of the benzenesulfonyl hydrazide

Table S8. Fragmentation of benzenesulfonyl group Ar_1

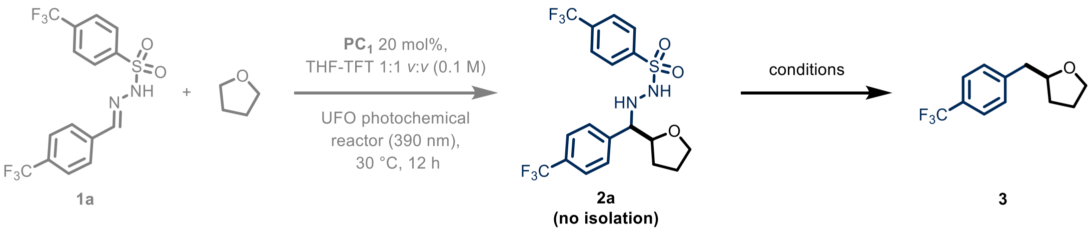

Conditions	conversion of 1a	Yield of **3**[a]
no base, 80°C, 3h	100%	traces
1 equiv. TEA, 80°C, 3h	100%	70%
1 equiv. TEA, 60°C, 3h	100%	30%
1 equiv. TEA, 50°C, 3h	10%	10%
3 equiv. TEA, rt, 3h	100%	traces
3 equiv.TEA, 60°C, 3h	50%	30%
3 equiv.TEA, 80°C, 3h	100%	90%
3 equiv. TEA, 80°C, 1h	100%	90%
3 equiv. TEA, 80°C, 1h[b]	100%	90%

Reaction conditions for the photochemical step:**1b** (0.2 mmols, 1 equiv.) in 2 mL of a 1:1 *v:v* THF:cosolvent solution (0.1 M). [a] Yield over two steps was determined via ^{1}H NMR using tricloroethylene as external standard;
[b] The photocatalyzed reaction was carried out in THF:TFT 1:1 v:v

Scheme S1. Fragmentation of benzenesulfonyl group Ar_2 (solvent switch required)

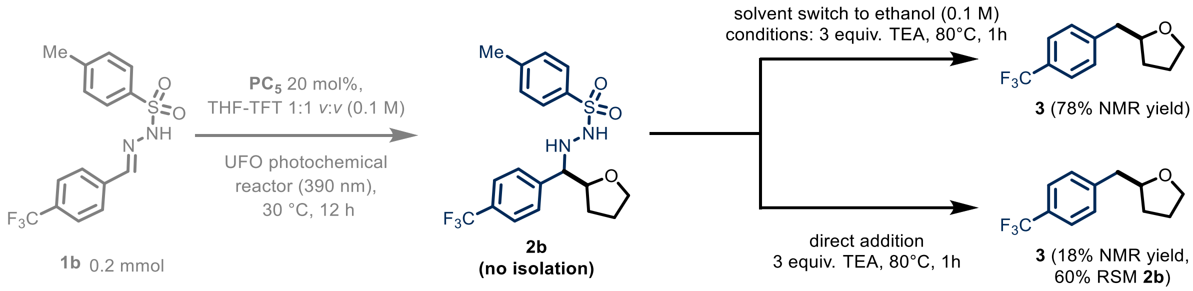

Scheme S2. Fragmentation of ethyl (2-tetrahydrofuran-2-yl)-2-(2-((4-(trifluoromethyl)phenyl)sulfonyl)hydrazineyl)acetate

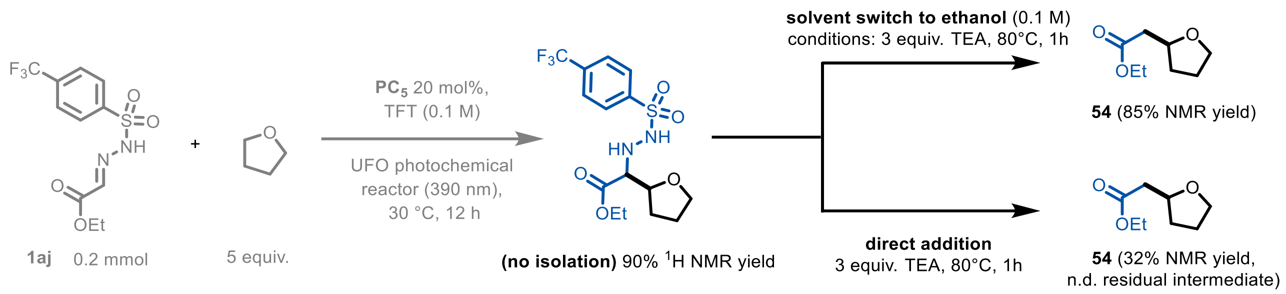

Scheme S3. Fragmentation of ethyl 2-(tetrahydrofuran-2-yl)-2-(2-tosylhydrazineyl)acetate

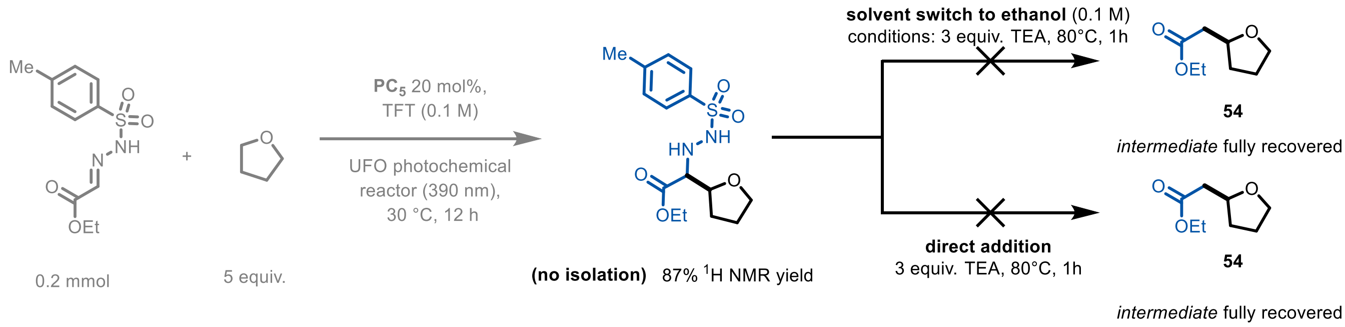

6. General procedure 2 (GP2): photochemical radical addition to benzenesulfonyl hydrazones using THF as the C(sp^3)−H donor and subsequent fragmentation of the benzenesulfonyl hydrazide

In a typical experiment, to an oven-dried 7 mL vial equipped with a stirring bar were added the sulfonyl hydrazone (0.400 mmol, 1 equiv.) and **PC$_5$** (20.1 mg, 20 mol%).and the vial was sealed with a rubber septum. Subsequently, trifluorotoluene (2 mL) and dry THF (2 mL) were added under N_2 atmosphere (0.1 M). The solution was then sparged with N_2 (1 min). The vial was stirred and irradiated in the UFO photochemical reactor (**See section 4**) for 12 h. The temperature was maintained at 30 °C during the course of the reaction. Then, the vial was removed from the photochemical reactor, TEA was added (3.0 equiv., 1.2 mmol, 167 μL) and the vial was placed in an oil bath at 80°C for 1h. Later, the reaction mixture was cooled to rt, diluted with EtOAc and transferred to a separatory funnel. The organic layer was washed with a 0.1 M HCl solution. The aqueous layer was then extracted with EtOAc and the combined organic layers were dried over Na_2SO_4. The solvent was removed under reduced pressure and the crude reaction mixture was purified by flash column chromatography on silica gel.

7. General procedure 3 (GP3): photochemical radical addition to benzenesulfonyl hydrazone (1a) using C(sp^3)−H donors and subsequent fragmentation of the benzenesulfonyl hydrazide

In a typical experiment, to an oven-dried 7 mL vial equipped with a stirring bar were added **1a** (0.4 mmol, 159 mg, 1 equiv.), **PC$_5$** (20.1 mg, 20 mol%) and the C−H donor (5, 10 or 20 equiv. as specified in the characterization section, if solid).The vial was sealed with a rubber septum and the solids were dissolved in trifluorotoluene (0.1 M). If liquid, the C−H donor was then added (5 to 20 equiv. or 2 mL, 0.1 M final concentration). The solution was then sparged with N_2 (1 min). In the case of volatile compounds, these were added after degassing via syringe through the septum. The vial was then stirred and irradiated in the UFO photochemical reactor (**See section 4**) for 12 h. The temperature was maintained at 30 °C during the course of the reaction. Then, the vial was removed from the photochemical reactor, TEA was added (3.0 equiv., 1.2 mmol, 167 μL) and it was placed in an oil bath at 80°C for 1h. Later, the reaction mixture was cooled to rt, diluted with EtOAc and transferred to a separatory funnel. The organic layer was washed with a 0.1 M HCl solution. The aqueous layer was then extracted with EtOAc and the combined organic layers were dried over Na_2SO_4. The solvent was removed under reduced pressure and the crude reaction mixture was purified by flash column chromatography on silica gel.

8. General procedure 4 (GP4): photochemical radical addition to ethyl 2-(2-((4-(trifluoromethyl)phenyl)sulfonyl)hydrazineylidene)acetate (1aj) and subsequent fragmentation of the benzenesulfonyl hydrazide

In a typical experiment, to an oven-dried 7 mL vial equipped with a stirring bar were added **1aj** (0.4 mmol, 130 mg, 1 equiv.), **PC$_5$** (20.1 mg, 20 mol%) and the C−H donor (5, 10 or 20 equiv. as specified in the characterization section, if solid).The vial was sealed with a rubber septum and the solids were dissolved in trifluorotoluene (0.1 M). If liquid, the C−H donor was then added (5, 10 or 20 equiv., 0.1 M final concentration). The solution was then sparged with N_2 (1 min). In the case of volatile compounds, these were added after degassing via syringe through the septum. The vial was then stirred and irradiated in the UFO photochemical reactor (**See section 4**) for 12 h. The temperature was maintained at 30 °C during the course of the reaction. Then, the vial was removed from the photochemical reactor and the solvent was removed under reduced pressure. The obtained crude mixture was then dissolved in 4 mL of dry ethanol (0.1 M), TEA was added (3.0 equiv., 1.2 mmol, 167 μL) and the vial was placed in an oil bath at 80°C for 2 h. The reaction mixture was cooled to rt and the solvent was removed under reduced pressure. The crude reaction mixture was then purified by flash column chromatography on silica gel.

9. Mechanistic investigation

9.1 UV-Vis characterization

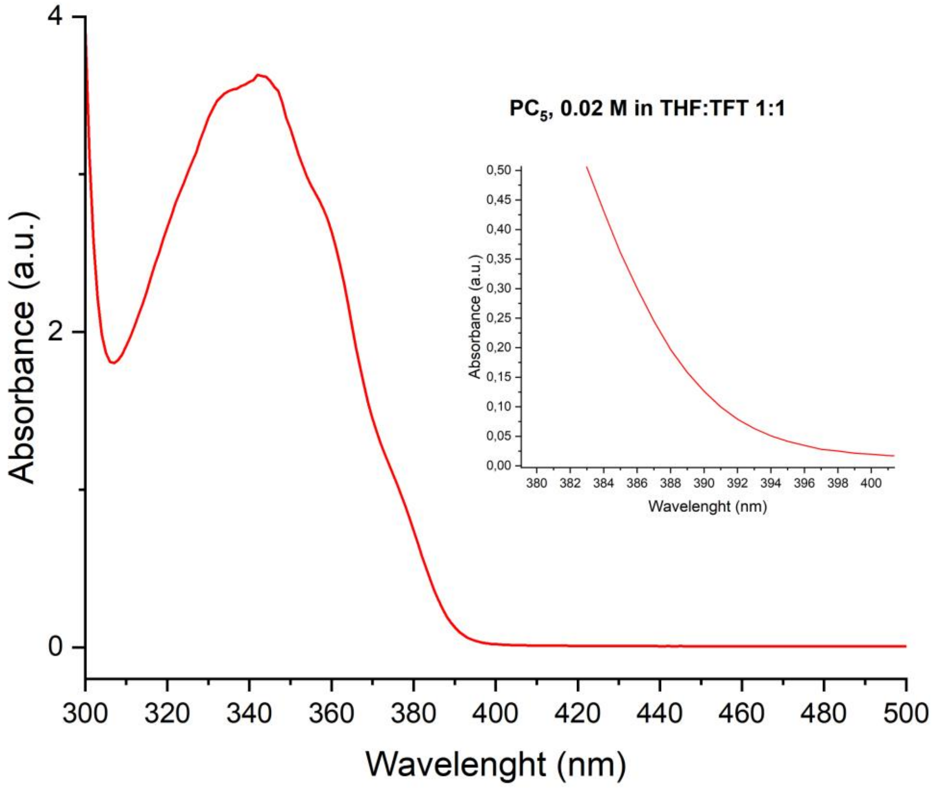

Figure S2: Absorption spectra of **PC$_5$**. The spectrum was recorded 0.02 M in THF:TFT 1:1 in quartz cuvettes (optical path: 1 cm) with a bandwidth of 5 nm and a data pitch of 1 nm. Scan rate: medium.

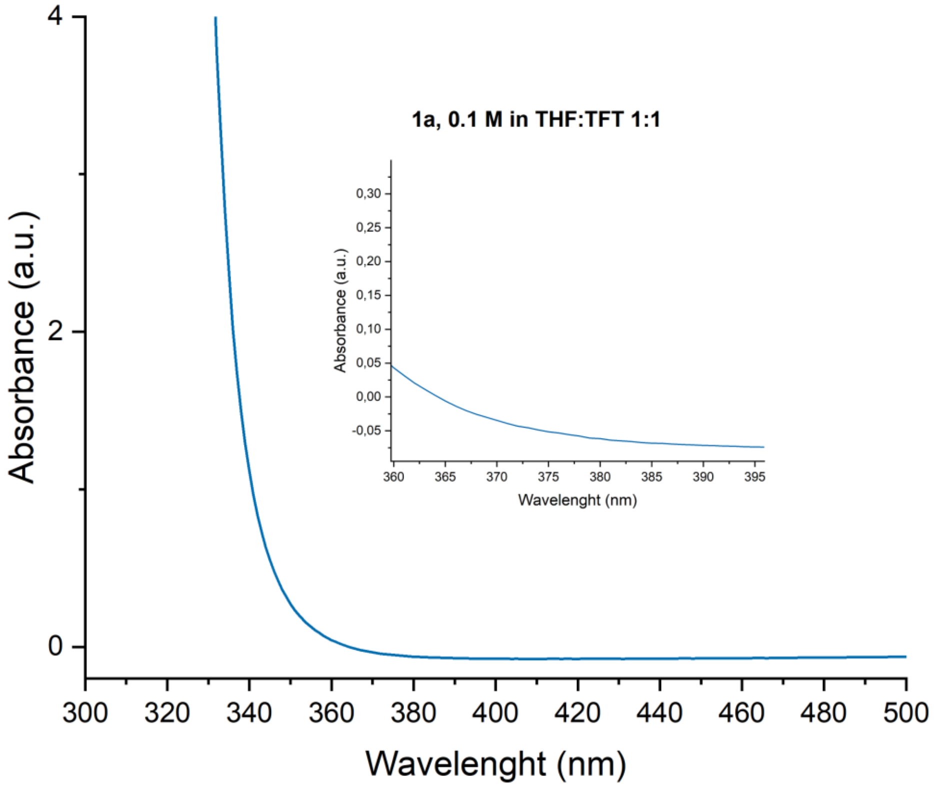

Figure S3: Absorption spectra of sulfonyl hydrazone **1a**. The spectrum was recorded 0.1 M in THF:TFT 1:1 in quartz cuvettes (optical path: 1 cm) with a bandwidth of 5 nm and a data pitch of 1 nm. Scan rate: medium.

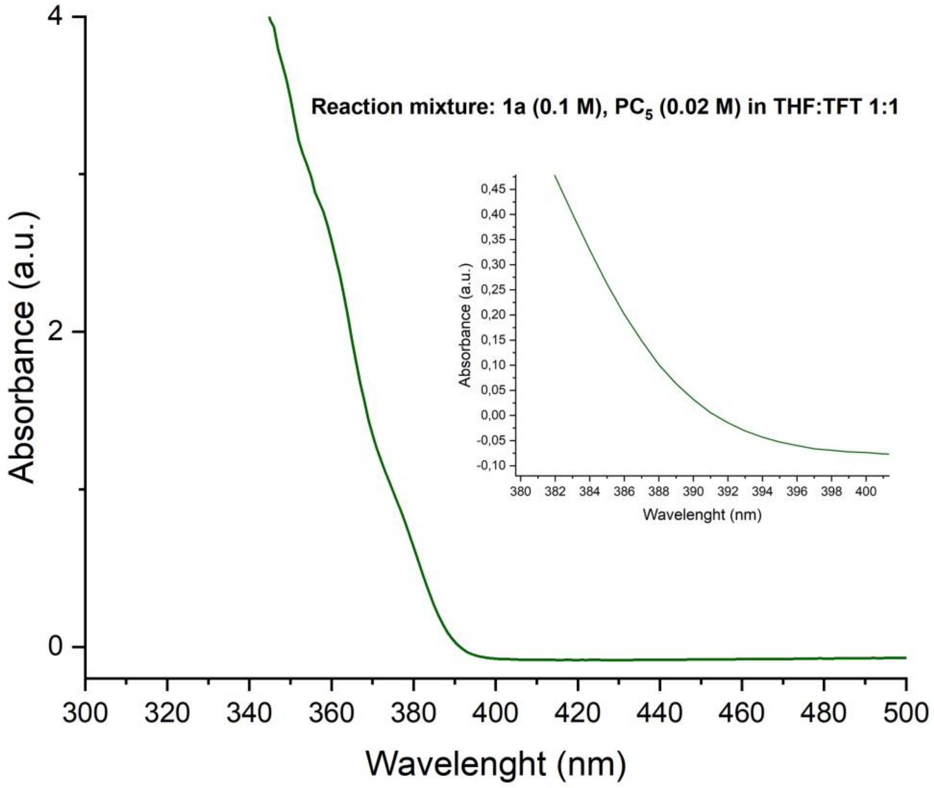

Figure S3: Absorption spectra of reaction mixture. The spectrum was recorded 0.1 M in THF:TFT 1:1 in quartz cuvettes (optical path: 1 cm) with a bandwidth of 5 nm and a data pitch of 1 nm. Scan rate: medium.

9.2 Kinetic Isotope Effect (KIE)

Competitive kinetic isotope effect

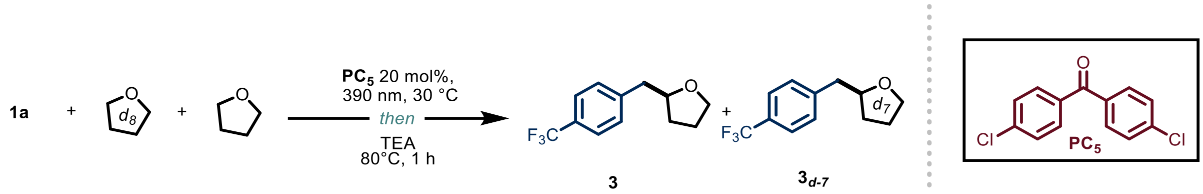

Parallel kinetic isotope effect

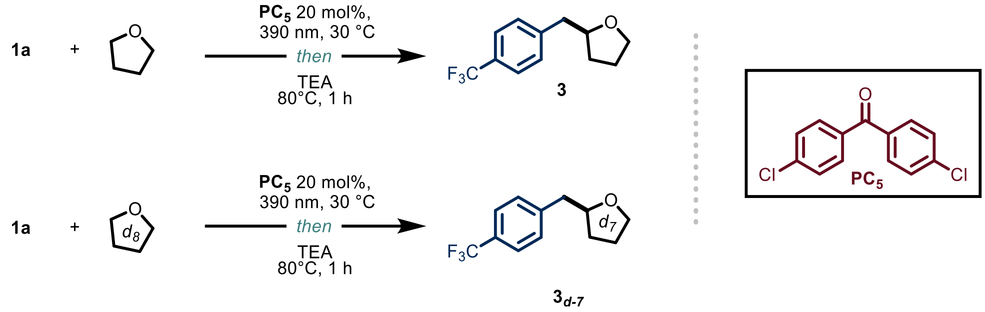

In an oven-dried 7 mL vial equipped with a stirring bar were added the sulfonyl hydrazone **1a** (0.2 mmol, 1 equiv.) and **PC5** (10 mg, 20 mol%).and the vial was sealed with a rubber septum. Subsequently, trifluorotoluene (1 mL) and dry THF or THF-d_8 (1 mL) were added under N_2 atmosphere (0.1 M). The solution was then sparged with N_2 (1 min). The vial was stirred and irradiated in the UFO photochemical reactor for the required reaction time (τ_r = 2, 5, 10 min). The temperature was maintained at 30 °C during the course of the reaction. Then, the vial was removed from the photochemical reactor, TEA was added (3.0 equiv., 0.6 mmol) and the vial was placed in an oil bath at 80°C for 1h. Later, the reaction mixture was cooled to rt, diluted with EtOAc and transferred to a separatory funnel. The organic layer was washed with a 0.1 M HCl solution. The aqueous layer was then extracted with EtOAc and the combined organic layers were dried over Na_2SO_4. The solvent was removed under reduced pressure. Then trichloroethylene was added as external standard and ^{1}H-NMR was used to evaluate the yield.

Reaction time (min)	Yield (%) with THF	Yield (%) with THF-d_8
0	0	0
2	6	2
5	13	5
10	25	11

Yield for THF) and THF-d_8) was plotted vs reaction time (**Figure S2**) to give a linear correlation and the KIE was calculated as the ratio between the slopes of the two curves to be **2.5**.

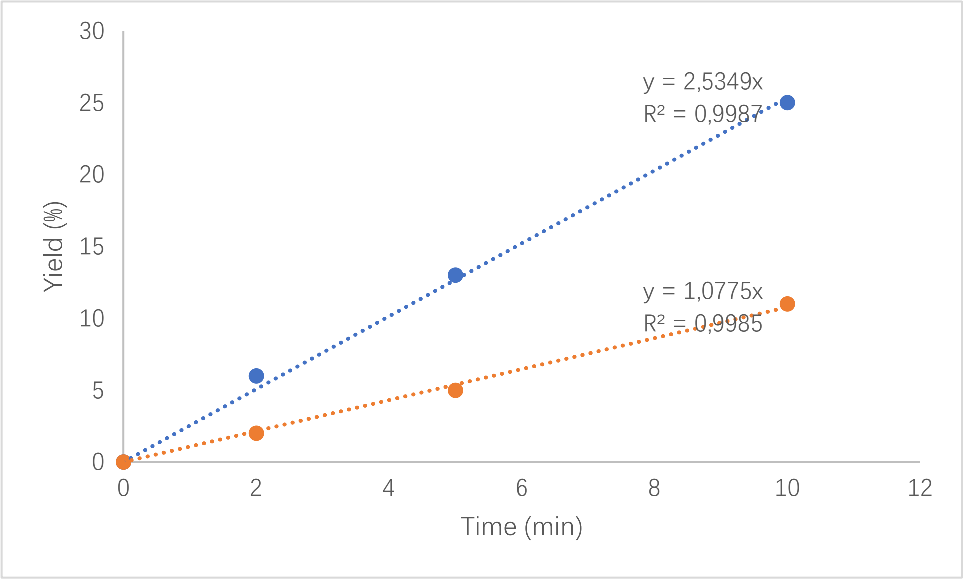

Figure S4. Yield vs reaction time for the evaluation of the KIE (THF blue line and THF-d_8 orange line).

9.3 Chemical Quenching

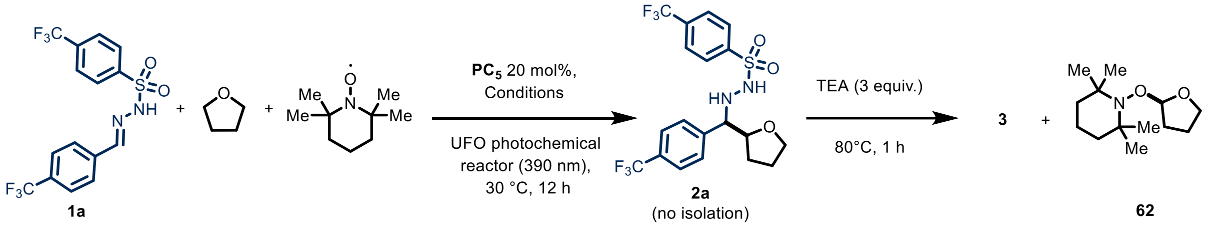

Conditions	equiv. of **TEMPO**	Conversion (**1a**)	Yield of **3**[a]	Yield of **62**
1:1, V:V, THF:TFT (0.1 M)	1	100%	37%	37%
20 equiv. THF (0.1 M in TFT)	1	100%	12%	23%

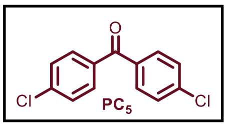

In a typical experiment, to an oven-dried 7 mL vial equipped with a stirring bar were added **1a** (0.4 mmol, 1 equiv.), TEMPO (1.0 equiv.) and **PC**$_5$ (20.1 mg, 20 mol%).and the vial was sealed with a rubber septum. Subsequently, trifluorotoluene and dry THF were added under N_2 atmosphere (0.1 M concentration). The solution was then sparged with N_2 (1 min). The vial was stirred and irradiated in the UFO photochemical reactor (**See section 4**) for 16 h. The temperature was maintained at 30 °C during the course of the reaction. Then, the vial was removed from the photochemical reactor, TEA was added (3.0 equiv., 1.2 mmol, 167 µL) and it was placed in an oil bath at 80°C for 1h. The reaction mixture was cooled to rt, diluted with EtOAc and transferred to a separatory funnel. The organic layer was then washed with a 0.1 M HCl solution and water. The acqueous layer was then extracted with EtOAc and the combined organic layers were dried over Na_2SO_4. The solvent was removed under reduced pressure and the crude reaction mixture was analyzed via ^{1}H-NMR using trichloroethylene as external standard.

9.4 Quantum yield

The quantum yield was determined via ferrioxalate actinometry according to a procedure reported in literature.[22]

To measure the moles of photons hitting the solution, a 0.15 M solution of ferrioxalate was prepared by dissolving 1.84 g (3.75 mmol) of $K_3[Fe(C_2O_4)_3]*3H_2O$ in 25 mL of 0.05 M H_2SO_4. Five 7-mL vials were charged with 4 mL of ferrioxalate solution and irradiated in the UFO reactor setup (**see Fig. S1, Section 4**). The irradiation times are reported below (**Table S9**). 20 µL of the irradiated solutions and the non-irradiated solution were added to a 2 mL phenanthroline solution (5.5 mM), prepared by dissolving 50 mg (0.28 mmol) of phenanthroline and 11.25 g of NaOAc in 50 mL of 0.5 M H_2SO_4. The resulting mixture was left equilibrating in the dark for one hour, before being further diluted by adding 50 µL of the mixture to 3 mL of distilled water. The final solution (dilution 1:6161) was analyzed by UV-VIS spectroscopy. The measured absorbances and corresponding moles of Fe^{2+} formed can be found in table **Table S9**. To calculate the moles of Fe^{2+} formed **Formula S1** was used.

$$mol(Fe^{2+}) = \frac{V * \Delta A}{d * \varepsilon}$$

Formula S1. Calculation of moles of Fe^{2+} formed: V is total volume after dilution (24.644 L), ΔA is the difference in absorption to the non-irradiated sample, d is the path length (1.00 cm) and ε the molar absorptivity at 510 nm (11.100 L mol^{-1} cm^{-1}).

	Irradiation time [s]	$A_{510\ nm}$	mol (Fe^{2+})
blank	0	0	0
1	2	0.00246	$5.46164 \bullet 10^{-6}$
2	4	0.00355	$7.88164 \bullet 10^{-6}$
3	6	0.00431	$9.56898 \bullet 10^{-6}$
4	8	0.00608	$1.34987 \bullet 10^{-5}$

Table S9. Irradiation times, absorbance and moles of Fe^{2+} formed.

Plotting the moles of Fe^{2+} formed versus the irradiation time gives a slope indicating the moles of Fe^{2+} formed per second, shown in **Figure S5:**

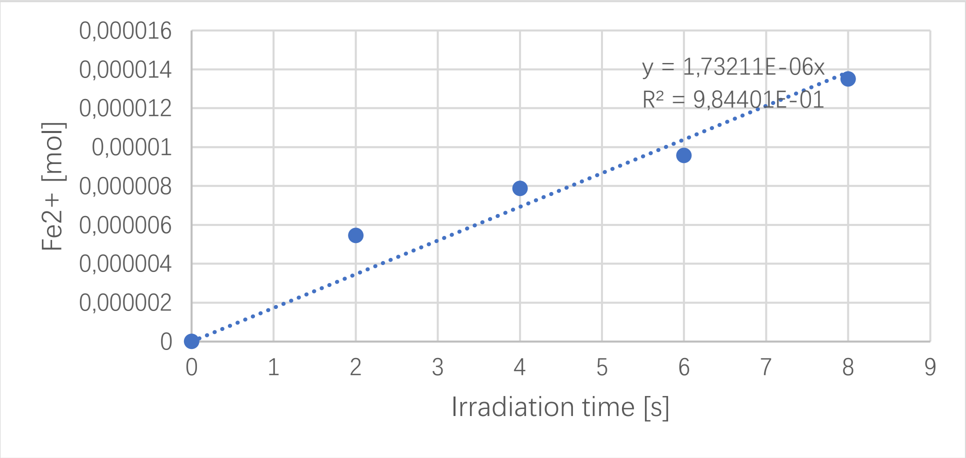

Figure S5. Moles of Fe^{2+} formed versus irradiation time.

By using the slope shown in **Figure S5** (1.73•10^{-6} mol/s) in **Formula S2** the photon flux of the setup can be calculated:

$$\frac{\frac{mol(Fe^{2+})}{s}}{f_1 * \varphi} = 1.44 * 10^{-6}\frac{E}{s}$$

Formula S2. *Calculation of photon flux. f_1 is the fraction of light absorbed by ferrioxolate at 510 nm (~1), φ is the quantum yield of the transformation of ferrioxalate (1.2).*

To calculate quantum yield **Formula S3** was used, dividing the moles of **3** formed in 10 minutes (25%, 0.1 mmol) by the moles of photons hitting the solution and the fraction of photons absorbed by the reaction solution.

$$\frac{mol(\mathbf{3})}{\frac{E}{s} * t * f_2} = 0.45$$

Formula S3. Calculation of the quantum yield: t is the irradiation time (600 s), f_2 is the fraction of light absorbed by **PC$_5$** at 390 nm (0.25).

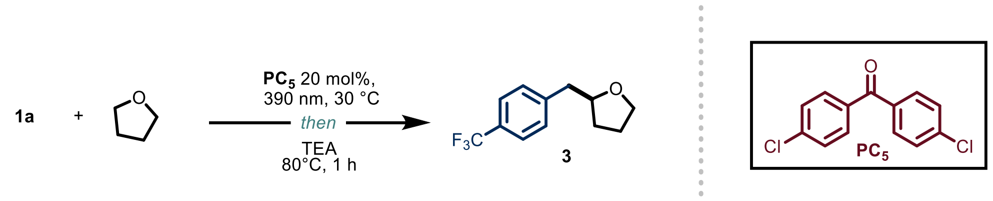

The quantum yield for the reaction is calculated to be **0.45**.

10. Scale-up procedure for compounds 3, 57, 58

Scale up for compound 3

For the scale up procedure of **3** a further short optimization of the photocatalyzed HAT step was performed. Instead of the UFO kinetic reactor, the reaction mixture was irradiated with two 40W Kessil PR160L-390 nm lamps from ca. 6 cm away. Cooling was applied via a strong compressed air flow to keep the temperature around 30 °C (see photos below).

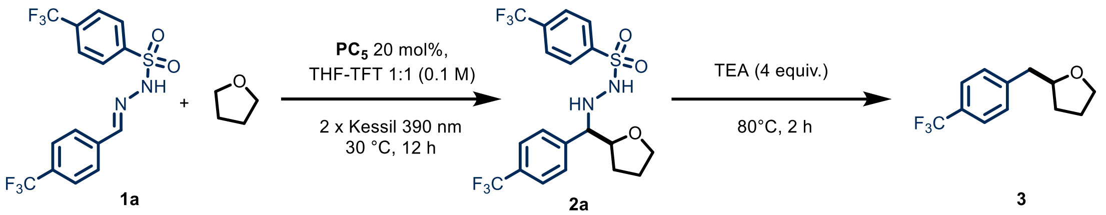

scale	*Light intensity*	*NMR Yield (**3**)*
0.2 mmol	100%	60%
0.2 mmol	25%	80%
10 mmol	100%	55%
10 mmol	25%	75%

Scale-up (10 mmol)

An oven dried 250 mL Schlenk flask, equipped with a magnetic stirring bar, was charged with **1a** (3.96 g, 10.0 mmol, 1 equiv.) and **PC$_5$** (502 mg, 0.20 equiv.) and sealed with a rubber septum. Subsequently, trifluorotoluene (50 mL) and dry THF (50 mL) were added under nitrogen atmosphere to prepare a 0.1 M solution (both the solvents were sparged with nitrogen for 20 min before the addition). The Schlenk tube was stirred and irradiated using the two Kessil lamps manifold showed below (390 nm, 25% light intensity, 6 cm away from the flask) for 12 h. (**Figure S6**). Compressed air was used to maintain the temperature at 30 °C during all the course of the reaction. Then, the flask was removed from the photochemical reactor, TEA was added (4.0 equiv., 40.0 mmol, 5.58 mL) and equipped with a reflux condenser (connected to the schlenk line). The vessel was placed under stirring in an oil bath at 80°C for 2 h (**Figure S7**). Subsequently, the reaction mixture was cooled to rt, diluted with EtOAc and transferred to a separatory funnel. The organic layer was then washed with a 0.1 M HCl solution. The aqueous layer was then extracted once with EtOAc and the combined organic layers were dried over Na_2SO_4. The solvent was then removed under reduced pressure and the crude reaction mixture was analyzed via ^{1}H NMR using trichloroethylene as external standard.

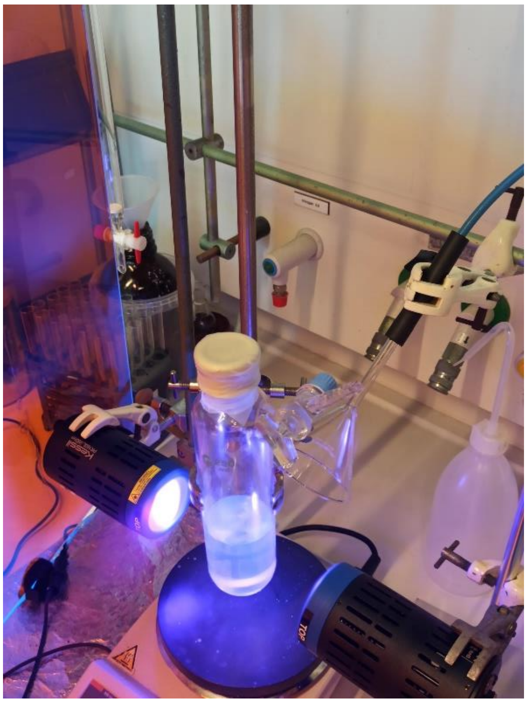

Figure S6. Photocatalyzed HAT step (390 nm)

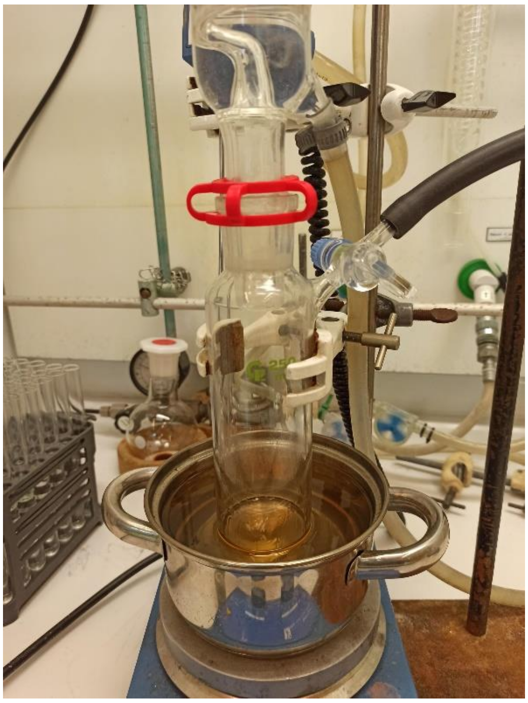

Figure S7. Cleavage step (80 °C)

*Scale up for compounds **57**, **58***

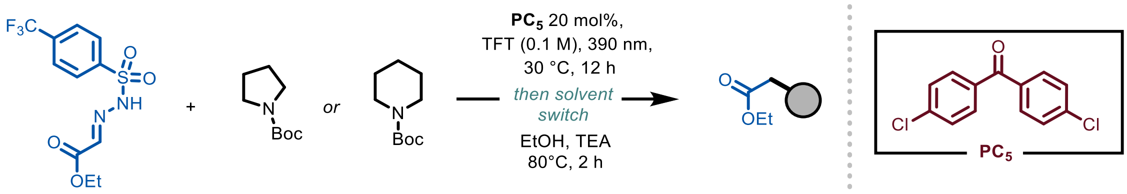

1aj **57** or **58**

An oven dried 250 mL Schlenk flask, equipped with a magnetic stirring bar, was charged with **1aj** (1.62 g, 5.0 mmol, 1 equiv.), **PC$_5$** (251 mg, 0.20 equiv.) and the corresponding Boc-protected secondary amine (25 mmol, 5 equiv.) and sealed with a rubber septum. Subsequently, trifluorotoluene (50 mL) was added under nitrogen atmosphere to prepare a 0.1 M solution (the solvent was sparged with nitrogen for 20 min before the addition). The Schlenk tube was stirred and irradiated using two Kessil lamps (390 nm, 100% light intensity, 6 cm away from the flask) for 12 h. Compressed air was used to maintain the temperature at 30 °C during all the course of the reaction. Subsequently, the solvent was removed under reduced pressure. The obtained crude mixture was then dissolved in 50 mL of dry ethanol (0.1 M), TEA was added (3.0 equiv., 15 mmol, 2.09 mL) and the mixture was placed in an oil bath at 80°C for 2 h. The reaction mixture was cooled to rt and the solvent was removed under reduced pressure. The crude reaction mixture was then purified by flash column chromatography on silica gel.

57, colorless liquid, 57%:

58, colorless liquid, 65%:

11. Post-Functionalizations

*Synthesis of di-peptide **69***

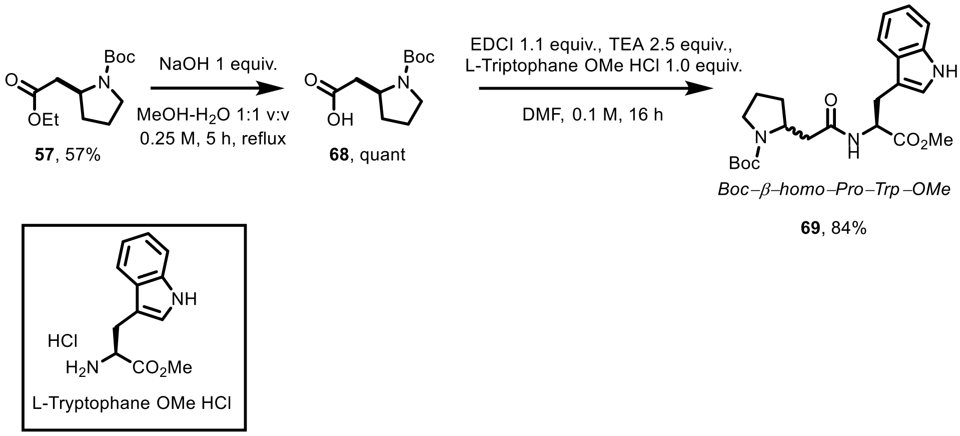

For experimental details and analytics of synthesized compounds **68** and **69** see **Section 12.5**.

*Synthesis of β-lactam **71***

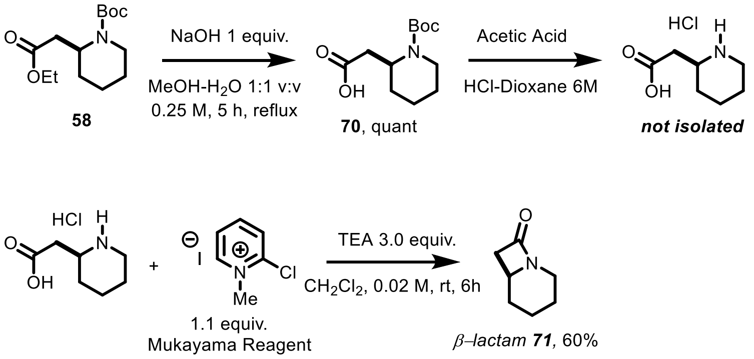

For experimental details and analytics of synthesized compounds **70** and **71** see **Section 12.5**.

12. Characterization data of synthesized compound

12.1 Characterization of sulfonyl hydrazones 1a – 1j

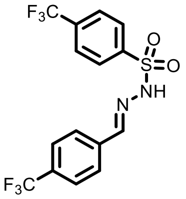

4-(trifluoromethyl)-N'-(4-(trifluoromethyl)benzylidene)benzenesulfonohydrazidebenzenesulfonohydrazide (**1a**). Prepared according to GP1 using 4-(trifluoromethyl)benzenesulfonohydrazide (4.80 g, 20 mmol, 1.0 equiv.) and 4-(trifluoromethyl)benzaldehyde (3.48 g, 2.73 mL, 20 mmol, 1.0 equiv.). Isolated as a white solid after filtration (7.57 g, 96% yield).

^{1}H NMR (400 MHz, $CDCl_3$) δ 8.40 (d, J = 2.9 Hz, 1H), 8.14 (d, J = 8 Hz, 2H), 7.84 – 7.78 (m, 3H), 7.69 (d, J = 8 Hz, 2H), 7.62 (d, J = 8 Hz, 2H).
^{13}C NMR (101 MHz, $CDCl_3$) δ 146.4, 141.5 (q, J = 1 Hz), 136.0 (q, J = 1 Hz), 135.2 (q, J = 33 Hz), 132.3 (d, J = 33 Hz), 128.5, 127.6, 126.4 (q, J = 4 Hz), 125.8 (d, J = 4 Hz) 123.7 (q, J = 272 Hz), 123.1 (q, J = 272 Hz).
^{19}F NMR (282 MHz, $CDCl_3$) δ -62.96, -63.25.
HRMS (ESI+) (m/z): $[M+H]^+$ calcd. for $C_{15}H_{10}F_6N_2O_2S$, 397.0445; found: 397.0457.

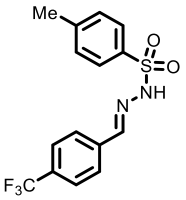

4-methyl-N'-(4-(trifluoromethyl)benzylidene)benzenesulfonohydrazide (**1b**). Prepared according to GP1 using 4-methylbenzenesulfonohydrazide (279 mg, 1.5 mmol, 1.0 equiv.) and 4-(trifluoromethyl)benzaldehyde (261 mg, 205 μL, 1.5 mmol, 1.0 equiv.). Isolated as a white solid after filtration (421 mg, 82% yield).

Characterization data are in accordance with literature.[23]
^{1}H NMR (400 MHz, $CDCl_3$) δ 8.58 (s, 1H), 7.89 (d, J = 8 Hz, 2H), 7.81 (s, 1H), 7.66 (d, J = 8 Hz, 2H), 7.58 (d, J = 8 Hz, 2H), 7.32 (d, J = 8 Hz, 2H), 2.41 (s, 3H).
^{13}C NMR (101 MHz, $CDCl_3$) δ 145.8, 144.8, 136.6 (q, J = 1 Hz), 135.2, 132.0 (J = 32 Hz), 130.0, 128.1, 127.6, 125.7 (q, J = 4 Hz), 123.9 (q, J = 272 Hz), 21.7.
^{19}F NMR (282 MHz, $CDCl_3$) δ -62.89.

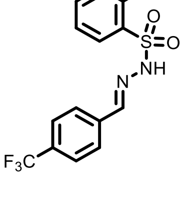

***2-nitro-N'-(4-(trifluoromethyl)benzylidene)benzenesulfonohydrazide* (1c).** Prepared according to GP1 using 2-nitrobenzenesulfonohydrazide (326 mg, 1.5 mmol, 1.0 equiv.) and 4-(trifluoromethyl)benzaldehyde (261 mg, 205μL, 1.5 mmol, 1.0 equiv.). Isolated as a white solid after filtration (476 mg, 85% yield).
Characterization data are in accordance with literature.[24]

^{1}H NMR (400 MHz, DMSO) δ 12.46 (s, 1H), 8.25 – 8.08 (m, 1H), 8.05 – 7.95 (m, 2H), 7.94 – 7.57 (m, 6H).
^{13}C NMR (101 MHz, DMSO) δ 147.9, 146.0, 137.3, 134.9, 132.7, 131.0, 130.6, 130.1 (q, *J* = 32 Hz), 127.5, 125.7 (q, *J* = 4 Hz), 124.6, 124.2 (q, *J* = 274 Hz).
^{19}F NMR (282 MHz, DMSO) δ -61.47.

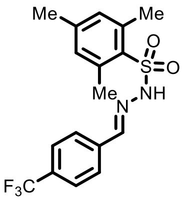

***2,4,6-trimethyl-N'-(4-(trifluoromethyl)benzylidene)benzenesulfonohydrazide* (1d).** Prepared according to GP1 using 2,4,6-trimethylbenzenesulfonohydrazide (321 mg, 1.5 mmol, 1.0 equiv.) and 4-(trifluoromethyl)benzaldehyde (261 mg, 205μL, 1.5 mmol, 1.0 equiv.). Isolated as a white solid after filtration (517 mg, 93% yield).
^{1}H NMR (400 MHz, DMSO) δ 11.91 (s, 1H), 7.99 (s, 1H), 7.71 – 7.66 (m, 4H), 7.00 (s, 2H), 2.65 (s, 6H), 2.19 (s, 3H).
^{13}C NMR (101 MHz, DMSO) δ 143.4, 142.4, 139.2, 137.8, 133.3, 131.6, 129.5 (q, *J* = 32 Hz), 127.0, 125.6 (q, *J* = 4 Hz), 124.0 (q, *J* = 272 Hz), 22.7, 20.3.
^{19}F NMR (282 MHz, DMSO) δ -61.37.
HRMS (ESI+) (m/z): $[M+H]^+$ calcd. for $C_{17}H_{17}F_3N_2O_2S$ 371.1041, found 371.1046.

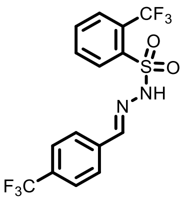

***2-(trifluoromethyl)-N'-(4-(trifluoromethyl)benzylidene)benzenesulfonohydrazide* (1e).** Prepared according to GP1 using 2-(trifluoromethyl)benzenesulfonohydrazide (360 mg, 1.5 mmol, 1.0 equiv.) and 4-(trifluoromethyl)benzaldehyde (261 mg, 205μL, 1.5 mmol, 1.0 equiv.). Isolated as a white solid after filtration (552 mg, 93% yield)
^{1}H NMR (400 MHz, DMSO) δ 12.39 (s, 1H), 8.18 (d, *J* = 8 Hz, 1H), 8.14 (s, 1H), 7.98 (d, *J* = 8 Hz, 1H), 7.93 – 7.88 (m, 1H), 7.87 – 7.80 (m, 1H), 7.74 (d, *J* = 8 Hz, 2H), 7.68 (d, *J* = 8 Hz, 2H).
^{13}C NMR (101 MHz, DMSO) δ 145.2, 138.0, 137.5, 133.6, 133.3, 131.4, 129.9 (q, *J* = 32 Hz), 128.4 (q, *J* = 6 Hz), 127.4, 126.5 (q, *J* = 33 Hz), 125.6 (q, *J* = 4 Hz), 124.0 (q, *J* = 272 Hz), 122.8 (q, *J* = 274 Hz).
^{19}F NMR (282 MHz, DMSO) δ -56.39, -61.54.
HRMS (ESI+) (m/z): $[M+H]^+$ calcd. for $C_{15}H_{10}F_6N_2O_2S$, 397.0445; found: 397.0448.

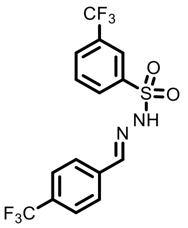

***3-(trifluoromethyl)-N'-(4-(trifluoromethyl)benzylidene)benzenesulfonohydrazide* (1f).** Prepared according to GP1 using 3-(trifluoromethyl)benzenesulfonohydrazide (360 mg, 1.5 mmol, 1.0 equiv.) and 4-(trifluoromethyl)benzaldehyde (261 mg, 205µL, 1.5 mmol, 1.0 equiv.). Isolated as a white solid after filtration (541 mg, 91% yield)

^{1}H NMR (400 MHz, DMSO) δ 12.01 (s, 1H), 8.24 (d, *J* = 8 Hz, 1H), 8.17 (s, 1H), 8.05 (s, 1H), 7.98 (d, *J* = 8 Hz, 1H), 7.86 (t, *J* = 8 Hz, 1H), 7.76 (d, *J* = 8 Hz, 2H), 7.65 (d, *J* = 8 Hz, 2H).

^{13}C NMR (101 MHz, DMSO) δ 146.5, 140.1, 137.3, 131.3, 130.9, 130.1 (q, *J* = 32 Hz), 130.0 (q, *J* = 33 Hz), 129.8 (q, *J* = 3 Hz), 127.4, 125.6 (q, *J* = 4 Hz), 123.9 (q, *J* = 272 Hz), 123.8 (q, *J* = 4 Hz), 123.3 (q, *J* = 273 Hz).

^{19}F NMR (282 MHz, $CDCl_3$) δ -62.88, -62.96.

HRMS (ESI+) (m/z): $[M+H]^+$ calcd. for $C_{15}H_{10}F_6N_2O_2S$, 397.0445; found: 397.0448.

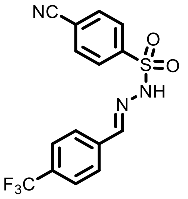

***4-cyano-N'-(4-(trifluoromethyl)benzylidene)benzenesulfonohydrazide* (1g).** Prepared according to GP1 using 4-cyanobenzenesulfonohydrazide (296 mg, 1.5 mmol, 1.0 equiv.) and 4-(trifluoromethyl)benzaldehyde (261 mg, 205 µL, 1.5 mmol, 1.0 equiv.). Isolated as a white solid after filtration (514 mg, 97% yield).

^{1}H NMR (400 MHz, DMSO) δ 12.11 (s, 1H), 8.17 – 8.01 (m, 5H), 7.79 (d, *J* = 8 Hz, 2H), 7.73 (d, *J* = 8 Hz, 2H).

^{13}C NMR (101 MHz, DMSO) δ 146.4, 142.8, 137.3 (q, *J* = 1 Hz),133.5, 130.0 (*J* = 32 Hz), 127.9, 127.5, 125.7 (q, *J* = 4 Hz), 124.0 (q, *J* = 272 Hz), 117.6, 115.7.

^{19}F NMR (282 MHz, DMSO) δ -61.38.

HRMS (ESI+) (m/z): $[M+H]^+$ calcd. for $C_{15}H_{10}F_3N_3O_2S$, 354.0524; found: 354.0524.

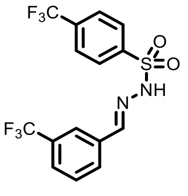

***4-(trifluoromethyl)-N'-(3-(trifluoromethyl)benzylidene)benzenesulfonohydrazide* (1h).** Prepared according to GP1 from 3-(trifluoromethyl)benzaldehyde (261 mg, 1.5 mmol, 1.0 equiv.) and 4-(trifluoromethyl)benzenesulfonohydrazide (360 mg, 1.5 mmol, 1.0 equiv.). Isolated as a white solid after removal of solvent and washing with hexane (570 mg, 96% yield).

^{1}H NMR (400 MHz, CDC13) δ 8.34 (s, 1H), 8.15 (d, *J* = 8 Hz, 2H), 7.86 – 7.75 (m, 5H), 7.64 (d, *J* = 8 Hz, 1H), 7.51 (t, *J* = 8 Hz, 1H).

^{13}C NMR (101 MHz, CDCl3) δ 146.5, 141.5, 135.2 (q, *J* = 33 Hz), 133.6, 131.4 (q, *J* = 33 Hz), 130.3, 129.4, 128.5, 127.3 (q, *J* = 4 Hz), 126.4 (q, *J* = 4 Hz), 124.2 (q, *J* = 4 Hz), 123.6 (q, *J* = 274 Hz), 123.0 (q, *J* = 274 Hz).
^{19}F NMR (282 MHz, CDCl3) δ -62.94, -63.24.
HRMS (ESI+) m/z $[M+H]^+$ calcd. for $C_{15}H_{10}F_6N_2O_2S_1$ 397.0445, found 397.0447.

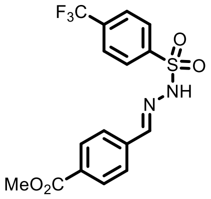

methyl 4-((2-((4-(trifluoromethyl)phenyl)sulfonyl)hydrazineylidene)methyl)benzoate (**1i**) Prepared according to GP1 using 4-(trifluoromethyl)benzenesulfonohydrazide (360 mg, 1.5 mmol, 1.0 equiv.) and methyl 4-formylbenzoate (246 mg, 1.5 mmol, 1.0 equiv.). Isolated as a white solid after filtration (567 mg, 98% yield).

^{1}H NMR (400 MHz, DMSO) δ 12.05 (s, 1H), 8.11 (d, *J* = 8 Hz, 2H), 8.05 – 7.98 (m, 3H), 7.95 (d, *J* = 8 Hz, 2H), 7.71 (d, *J* = 8 Hz, 2H), 3.84 (s, 3H).
^{13}C NMR (101 MHz, DMSO) δ 165.7, 146.6, 142.8, 137.7, 132.8 (d, *J* = 32 Hz), 130.7, 129.6, 128.2, 127.1, 126.6 (q, *J* = 4 Hz), 123.4 (q, *J* = 272 Hz), 52.3.
^{19}F NMR (282 MHz, DMSO) δ -61.76
HRMS (ESI+) (m/z): $[M+H]^+$ calcd. for $C_{16}H_{13}F_3N_2O_4S$, 387.0626; found: 387.0629.

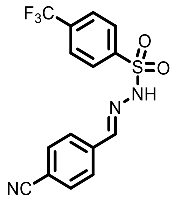

N'-(4-cyanobenzylidene)-4-(trifluoromethyl)benzenesulfonohydrazide (**1j**). Prepared according to GP1 using 4-(trifluoromethyl)benzenesulfonohydrazide (360 mg, 1.5 mmol, 1.0 equiv.) and 4-formylbenzonitrile (197 mg, 1.5 mmol, 1.0 equiv.). Isolated as a white solid after filtration (434 mg, 82% yield).

^{1}H NMR (400 MHz, DMSO) δ 12.14 (s, 1H), 8.11 (d, *J* = 8 Hz, 2H), 8.04 – 7.97 (m, 3H), 7.85 (d, *J* = 8 Hz, 2H), 7.76 (d, *J* = 8 Hz, 2H).
^{13}C NMR (101 MHz, DMSO) δ 146.0, 142.7, 137.8, 132.8 (*J* = 32 Hz), 132.7, 128.2, 127.5, 126.6 (q, *J* = 4 Hz), 123.4 (q, *J* = 272 Hz), 118.5, 112.2.
^{19}F NMR (282 MHz, DMSO) δ -61.74.
HRMS (ESI+) (m/z): $[M]^+$ calcd. for $C_{15}H_{10}F_3N_3O_2S$, 354.0524; found: 354.0533.

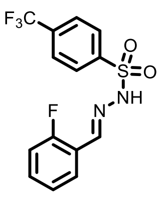

N'-(2-fluorobenzylidene)-4-(trifluoromethyl)benzenesulfonohydrazide (**1k**) Prepared according to GP1 using 4-(trifluoromethyl)benzenesulfonohydrazide (360 mg, 1.5 mmol, 1.0 equiv.) and 2-fluorobenzaldehyde (186 mg, 158 µL, 1.5 mmol, 1.0 equiv.). Isolated as a white solid after filtration (454 mg, 87% yield).

^{1}H NMR (300 MHz, $CDCl_3$) δ 8.55 (s, 1H), 8.15 (d, J = 8 Hz, 2H), 8.06 (s, 1H), 7.86 – 7.74 (m, 3H), 7.42 – 7.25 (m, 1H), 7.18 – 7.08 (m, 1H), 7.02 (ddd, J = 10, 8, 1 Hz, 1H).
^{13}C NMR (75 MHz, $CDCl_3$) δ 161.4 (d, J = 253 Hz), 141.9 (d, J = 5 Hz), 141.8, 135.2 (q, J = 33 Hz), 132.5 (d, J = 8 Hz), 128.6, 127.0 (d, J = 2 Hz), 126.4 (q, J = 4 Hz), 124.63 (d, J = 4 Hz), 123.2 (q, J = 273 Hz), 120.8 (d, J = 10 Hz), 115.9 (d, J = 21 Hz).
^{19}F NMR (282 MHz, $CDCl_3$) δ -63.22, -120.44.
HRMS (ESI+) (m/z): $[M+H]^+$ calcd. for $C_{14}H_{10}F_4N_2O_2S$, 347.0477; found: 347.0475.

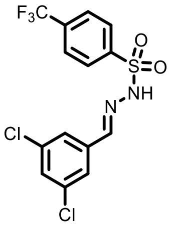

N'-(3,5-dichlorobenzylidene)-4-(trifluoromethyl)benzenesulfonohydrazide (**1l**) Prepared according to GP1 using 4-(trifluoromethyl)benzenesulfonohydrazide (360 mg, 1.5 mmol, 1.0 equiv.) and 3,5-dichlorobenzaldehyde (263 mg, 1.5 mmol, 1.0 equiv.). Isolated as a white solid after filtration (520 mg, 82% yield).

^{1}H NMR (400 MHz, DMSO) δ 12.16 (s, 1H), 8.23 (s, 1H), 8.10 (d, J = 8 Hz, 2H), 8.00 (d, J = 8 Hz, 2H), 7.76 (d, J = 9 Hz, 1H), 7.63 (d, J = 2 Hz, 1H), 7.42 (dd, J = 8, 2 Hz, 1H).
^{13}C NMR (101 MHz, DMSO) δ 142.7, 142.6, 135.4, 133.7, 132.8 (q, J = 32 Hz) 129.7, 129.4, 128.2, 128.0, 127.9, 126.6 (q, J = 4 Hz), 123.3 (q, J = 273 Hz)
^{19}F NMR (282 MHz, DMSO) δ -61.76.
HRMS (ESI+) (m/z): $[M+H]^+$ calcd. for $C_{14}H_9Cl_2F_3N_2O_2S$, 396.9792; found: 396.9794.

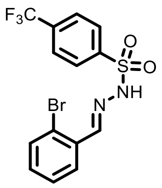

N'-(2-bromobenzylidene)-4-(trifluoromethyl)benzenesulfonohydrazide (**1m**) Prepared according to GP1 using 4-(trifluoromethyl)benzenesulfonohydrazide (360 mg, 1.5 mmol, 1.0 equiv.) and 2-bromobenzaldehyde (278 mg, 175 µL, 1.5 mmol, 1.0 equiv.). Isolated as a white solid after filtration (567 mg, 93% yield).

^{1}H NMR (400 MHz, DMSO) δ 12.10 (s, 1H), 8.26 (s, 1H), 8.11 (d, J = 8 Hz, 2H), 8.01 (d, J = 8 Hz, 2H), 7.75 (dd, J = 8, 2 Hz, 1H), 7.62 (dt, J = 8, 1 Hz, 1H), 7.43 – 7.36 (m, 1H), 7.35 – 7.27 (m, 1H).
^{13}C NMR (101 MHz, DMSO) δ 146.0, 142.7, 133.1, 132.8 (q, J = 32 Hz), 132.1, 132.0, 128.2, 128.1, 127.0, 126.6 (q, J = 4 Hz), 123.4 (q, J = 272 Hz), 123.3.
^{19}F NMR (282 MHz, DMSO) δ -61.77
HRMS (ESI+) (m/z): $[M+H]^+$ calcd. for $C_{14}H_{10}BrF_3N_2O_2S$, 406.9677; found: 406.9690.

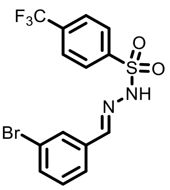

N'-(3-bromobenzylidene)-4-(trifluoromethyl)benzenesulfonohydrazide **(1n).** Prepared according to GP1 using 4-(trifluoromethyl)benzenesulfonohydrazide (360 mg, 1.5 mmol, 1.0 equiv.) and 3-bromobenzaldehyde (277 mg, 175µL, 1.5 mmol, 1.0 equiv.). Isolated as a white solid after filtration (544 mg, 89% yield)

^{1}H NMR (400 MHz, DMSO) δ 11.98 (s, 1H), 8.11 (d, *J* = 8 Hz, 2H), 7.99 (d, *J* = 8 Hz, 2H), 7.94 (s, 1H), 7.75 – 7.72 (m, 1H), 7.59 – 7.53 (m, 2H), 7.33 (t, *J* = 8 Hz, 1H).
^{13}C NMR (101 MHz, DMSO) δ 146.3, 142.8, 135.8, 133.0, 132.8 (q, *J* = 32 Hz), 130.9, 129.2, 128.2, 126.6 (q, *J* = 4 Hz), 125.8. 123.4 (q, *J* = 273 Hz), 122.1.
^{19}F NMR (282 MHz, DMSO) δ -61.80.
HRMS (ESI+) (m/z): $[M+H]^+$ calcd. for $C_{14}H_{10}BrF_3N_2O_2S$, 408.9657; found: 408.9753.

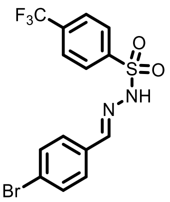

N'-(4-bromobenzylidene)-4-(trifluoromethyl)benzenesulfonohydrazide **(1o).** Prepared according to GP1 using 4-(trifluoromethyl)benzenesulfonohydrazide (360 mg, 1.5 mmol, 1.0 equiv.) and 4-bromobenzaldehyde (277 mg, 149 µL, 1.5 mmol, 1.0 equiv.). Isolated as a white solid after filtration (580 mg, 95% yield)

^{1}H NMR (400 MHz, DMSO) δ 11.90 (s, 1H), 8.11 (d, *J* = 8 Hz, 2H), 7.99 (d, *J* = 8 Hz, 2H), 7.95 (s, 1H), 7.57 (d, *J* = 9 Hz, 2H), 7.52 (d, *J* = 9 Hz, 2H).
^{13}C NMR (101 MHz, DMSO) δ 146.9, 142.8, 132.8 (q, *J* = 32 Hz), 132.7, 131.8, 128.7, 128.2, 126.5 (q, *J* = 4 Hz), 123.6, 123.4 (q, *J* = 273 Hz).
^{19}F NMR (282 MHz, DMSO) δ -61.69.
HRMS (ESI+) (m/z): $[M+H]^+$ calcd. for $C_{14}H_{10}BrF_3N_2O_2S$, 408.9657; found: 408.9626.

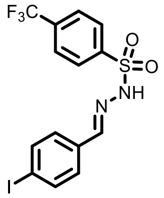

N'-(4-iodobenzylidene)-4-(trifluoromethyl)benzenesulfonohydrazide (**1p**) Prepared according to GP1 using 4-(trifluoromethyl)benzenesulfonohydrazide (360 mg, 1.5 mmol, 1.0 equiv.) and 4-iodobenzaldehyde (348 mg, 1.5 mmol, 1.0 equiv.). Isolated as a white solid after filtration (550 mg, 81% yield).

^{1}H NMR (400 MHz, $CDCl_3$) δ 8.12 (d, *J* = 8 Hz, 2H), 8.06 (s, 1H), 7.80 (d, *J* = 8 Hz, 2H), 7.75 – 7.67 (m, 3H), 7.34 – 7.28 (m, 2H).

^{13}C NMR (101 MHz, $CDCl_3$) δ 147.5, 141.7, 135.3 (q, J = 32 Hz), 138.2, 132.3, 128.9, 128.6, 126.45 (q, J = 4 Hz), 123.2 (q, J = 272 Hz), 97.5.
^{19}F NMR (282 MHz, $CDCl_3$) δ -63.20
HRMS (ESI+) (m/z): $[M+H]^+$ calcd. for $C_{14}H_{10}F_3IN_2O_2S$, 454.9538; found: 454.9533.

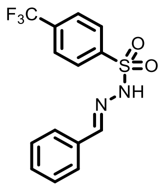

***N'-benzylidene-4-(trifluoromethyl)benzenesulfonohydrazide* (1q).** Prepared according to GP1 from benzaldehyde (159 mg, 1.5 mmol, 1.0 equiv.) and 4-(trifluoromethyl)benzenesulfonohydrazide (360 mg, 1.5 mmol, 1.0 equiv.). Isolated as a white solid after removal of solvent and washing with hexane (309 mg, 63% yield).

^{1}H NMR (400 MHz, DMSO) δ 11.79 (s, 1H), 8.12 (d, J = 8 Hz, 2H), 8.00 (d, J = 8 Hz, 2H), 7.97 (s, 1H), 7.62 – 7.53 (m, 2H), 7.42 – 7.34 (m, 3H).
^{13}C NMR (101 MHz, DMSO) δ 148.1, 142.8, 133.4, 132.7 (q, J = 32 Hz), 130.3, 128.8, 128.2, 126.9, 126.5 (q, J = 4 Hz), 123.4. (q, J = 273 Hz).
^{19}F NMR (282 MHz, CDCl3) δ -63.20.
HRMS (ESI+) m/z $[M+H]^+$ calcd. for $C_{14}H_{11}F_3N_2O_2S_1$ 329.0572, found 329.0572.

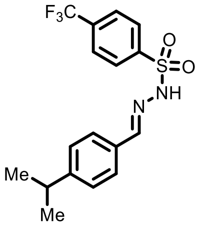

N'-(4-isopropylbenzylidene)-4-(trifluoromethyl)benzenesulfonohydrazide (**1r**). Prepared according to GP1 using 4-(trifluoromethyl)benzenesulfonohydrazide (360 mg, 1.5 mmol, 1.0 equiv.) and 4-isopropylbenzaldehyde (222 mg, 228 µL, 1.5 mmol, 1.0 equiv.). Isolated as a white solid after filtration (501 mg, 90% yield).

^{1}H NMR (400 MHz, DMSO) δ 11.71 (s, 1H), 8.12 (d, J = 8 Hz, 2H), 7.99 – 7.95 (m, 3H), 7.49 (d, J = 8 Hz, 2H), 7.21 (d, J = 8 Hz, 2H), 2.81 (h, J = 7 Hz, 1H), 1.11 (d, J = 7 Hz, 6H).
^{13}C NMR (101 MHz, DMSO) δ 150.9, 148.1, 142.9, 132.7 (q, J = 32 Hz), 131.2, 128.2, 127.0, 126.7, 126.4 (q, J = 4 Hz), 123.4 (q, J = 273 Hz), 33.4, 23.5.
^{19}F NMR (282 MHz, DMSO) δ -61.81.
HRMS (ESI+) (m/z): $[M+H]^+$ calcd. for $C_{17}H_{17}F_3N_2O_2S$, 371.1041; found: 371.1043.

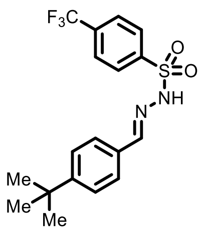

N'-(4-(tert-butyl)benzylidene)-4-(trifluoromethyl)benzenesulfonohydrazide **(1s).** Prepared according to GP1 from 4-(tert-butyl)benz aldehyde (243 mg, 1.5 mmol, 1.0 equiv.) and 4-(trifluoromethyl)benzenesulfonohydrazide (360 g, 1.5 mmol, 1.0 equiv.). Isolated as a white solid after removal of solvent and washing with hexane (456 mg, 79% yield).

^{1}H NMR (400 MHz, DMSO) δ 11.68 (s, 1H), 8.09 (d, *J* = 8 Hz, 2H), 8.01 (d, *J* = 8 Hz, 2H), 7.93 (s, 1H), 7.50 (d, *J* = 8 Hz, 2H), 7.40 (d, *J* = 8 Hz, 2H), 1.24 (s, 9H).
^{13}C NMR (101 MHz, DMSO) δ 153.2, 148.0, 142.8, 132.7 (q, *J* = 32 Hz), 130.7, 128.2, 126.7, 126.5 (q, *J* = 4 Hz), 125.6, 123.4 (q, *J* = 273 Hz), 34.6, 30.9.
^{19}F NMR (282 MHz, CDCl3) δ -56.94.
HRMS (ESI+) m/z [M+H]$^{+}$ calcd. for $C_{18}H_{19}F_3N_2O_2S_1$ 385.1198, found 385.1195.

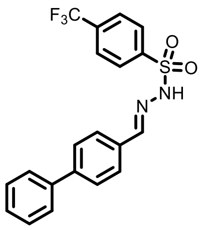

N'-([1,1'-biphenyl]-4-ylmethylene)-4-(trifluoromethyl)benzenesulfonohydrazide **(1t).** Prepared according to GP1 using 4-(trifluoromethyl)benzenesulfonohydrazide (360 mg, 1.5 mmol, 1.0 equiv.) and [1,1'-biphenyl]-4-carbaldehyde (273 mg, 1.5 mmol, 1.0 equiv.). Isolated as a white solid after filtration (528 mg, 87% yield)

^{1}H NMR (400 MHz, DMSO) δ 11.83 (s, 1H), 8.13 (d, *J* = 8 Hz, 2H), 8.05 – 7.97 (m, 3H), 7.73 – 7.62 (m, 6H), 7.49 – 7.43 (m, 2H), 7.37 (t, *J* = 7 Hz, 1H).
^{13}C NMR (101 MHz, DMSO) δ 147.7, 142.8, 141.8, 139.2, 132.7 (q, *J* = 32 Hz), 132.5, 129.0, 128.2, 127.9, 127.5, 127.0, 126.7, 126.5 (q, *J* = 4 Hz), 123.4 (q, *J* = 273 Hz).
^{19}F NMR (282 MHz, DMSO) δ -56.95.
HRMS (ESI+) (m/z): [M+H]$^{+}$ calcd. for $C_{20}H_{15}F_3N_2O_2S$, 405.0885; found: 405.0882

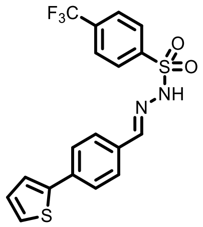

N'-(4-(thiophen-2-yl)benzylidene)-4-(trifluoromethyl)benzenesulfonohydrazide **(1u).** Prepared according to GP1 from 4-(thiophen-2-yl)benzaldehyde (282 mg, 1.5 mmol, 1.0 equiv.) and 4-

(trifluoromethyl)benzenesulfonohydrazide (360 mg, 1.5 mmol, 1.0 equiv.). Isolated as a white solid after filtration (458 mg, 74% yield).

^{1}H NMR (400 MHz, DMSO) δ 11.83 (s, 1H), 8.21 – 7.93 (m, 5H), 7.76 – 7.53 (m, 6H), 7.21 – 7.12 (m, 1H).
^{13}C NMR (101 MHz, DMSO) δ 147.5, 142.8, 142.5, 135.4, 132.7 (q, *J* = 32 Hz), 132.4, 128.7, 128.2, 127.7, 126.6, 126.5, 125.6, 124.6, 123.4 (q, *J* = 272 Hz).
^{19}F NMR (282 MHz, DMSO) δ -61.67.
HRMS (ESI+) m/z [M+H]$^+$ calcd. for $C_{18}H_{13}F_3N_2O_2S_2$ 411.0449, found 411.0445.

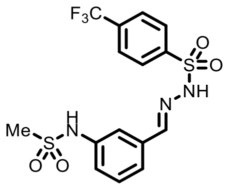

N-(3-((2-((4-(trifluoromethyl)phenyl)sulfonyl)hydrazineylidene)methyl)phenyl)methanesulfonamide **(1v).** Prepared according to GP1 from N-(3-formylpahenyl)methanesulfonamide (299 mg, 1.5 mmol, 1.0 equiv.) and 4-(trifluoromethyl)benzenesulfonohydrazide (360 mg, 1.5 mmol, 1.0 equiv.). Isolated as a white solid after filtration (550 mg, 87% yield).

^{1}H NMR (400 MHz, DMSO) δ 11.83 (s, 1H), 9.87 (s, 1H), 8.10 (d, *J* = 8 Hz, 2H), 8.01 (d, *J* = 8 Hz, 2H), 7.93 (s, 1H), 7.45 (s, 1H), 7.36 (t, *J* = 8 Hz, 1H), 7.29 (d, *J* = 8 Hz, 1H), 7.24 (d, *J* = 8 Hz, 1H), 2.99 (s, 3H).
^{13}C NMR (101 MHz, DMSO) δ 147.57, 142.78, 138.95, 134.54, 132.79 (q, *J* = 32 Hz), 129.87, 128.24, 126.55 (q, *J* = 4 Hz), 123.4 (q, *J* = 273 Hz), 122.68, 121.29, 117.18, 39.4.
^{19}F NMR (282 MHz, DMSO) δ -61.70.
HRMS (ESI+) m/z [M+H]$^+$ calcd. for $C_{15}H_{14}F_3N_3O_4S_2$ 422.0456, found 422.0462.

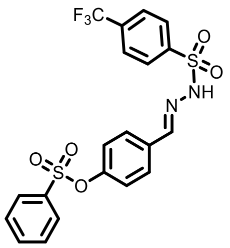

4-((2-((4-(trifluoromethyl)phenyl)sulfonyl)hydrazineylidene)methyl)phenyl benzenesulfonate (**1w**) Prepared according to GP1 using 4-(trifluoromethyl)benzenesulfonohydrazide (360 mg, 1.5 mmol, 1.0 equiv.) and 4-formylphenyl benzenesulfonate (393 mg, 1.5 mmol, 1.0 equiv.). Isolated as a pale yellow solid after filtration (700 mg, 96% yield).

^{1}H NMR (400 MHz, $CDCl_3$) δ 8.34 (s, 1H), 8.10 (d, *J* = 8 Hz, 2H), 7.87 – 7.76 (m, 4H), 7.73 (s, 1H), 7.71 – 7.65 (m, 1H), 7.57 – 7.45 (m, 4H), 7.02 – 6.94 (m, 2H).
^{13}C NMR (101 MHz, $CDCl_3$) δ 151.1, 146.8, 141.8, 135.2, 135.1 (q, *J* = 32 Hz), 134.6, 131.9, 129.4, 128.8, 128.6, 128.6, 126.4 (q, *J* = 4 Hz), 123.2 (q, *J* = 272 Hz), 122.9.
^{19}F NMR (282 MHz, $CDCl_3$) δ -63.19.
HRMS (ESI+) (m/z): [M+H]$^+$ calcd. for $C_{20}H_{15}F_3N_2O_5S_2$, 485.0453; found: 485.0451.

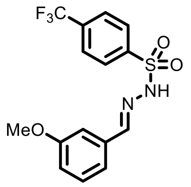

***N'-(3-methoxybenzylidene)-4-(trifluoromethyl)benzenesulfonohydrazide* (1x).** Prepared according to GP1 from 3-methoxybenzaldehyde (204 mg, 1.5 mmol, 1.0 equiv.) and 4-(trifluoromethyl)benzenesulfonohydrazide (360 mg, 1.5 mmol, 1.0 equiv.). Isolated as a yellow oil (359 mg, 67% yield).

^{1}H NMR 1H NMR (400 MHz, CDCl3) δ 9.11 (s, 1H), 8.16 (d, *J* = 8 Hz, 2H), 7.86 (s, 1H), 7.73 (d, *J* = 8 Hz, 2H), 7.22 (t, *J* = 8 Hz, 1H), 7.16 – 7.05 (m, 2H), 6.89 (ddd, *J* = 8, 3, 1 Hz, 1H), 3.77 (s, 3H).
^{13}C NMR (101 MHz, CDCl3) δ 159.8, 149.1, 141.7 (d, *J* = 2 Hz), 134.9 (q, *J* = 33 Hz), 134.3, 129.8, 128.5, 126.3 (q, *J* = 4 Hz), 123.2 (q, *J* = 271 Hz), 120.5, 116.8, 111.8, 55.3.
^{19}F NMR (282 MHz, CDCl3) δ -63.20.
HRMS (ESI+) m/z [M+H]$^+$ calcd. for $C_{15}H_{13}F_3N_2O_3S_1$ 359.0677, found 359.0673.

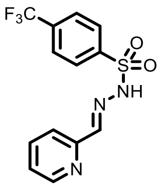

***N'-(pyridin-2-ylmethylene)-4-(trifluoromethyl)* (1y).** Prepared according to GP1 from picolinaldehyde (535 mg, 5.0 mmol, 1.0 equiv.) and 4-(trifluoromethyl)benzenesulfonohydrazide (1.201 g, 5.0 mmol, 1.0 equiv.). Isolated as a white solid after filtration (1.471 g, 89% yield, 90% purity, see NMR spectra).

^{1}H NMR (400 MHz, DMSO) δ 12.12 (s, 1H), 8.58 – 8.52 (m, 1H), 8.12 (d, *J* = 8 Hz, 2H), 8.00 (d, *J* = 8 Hz, 2H), 7.97 (s, 1H), 7.84 – 7.74 (m, 2H), 7.42 – 7.32 (m, 1H).
^{13}C NMR (101 MHz, DMSO) δ 152.1, 149.5, 148.0, 142.7, 137.0, 133.3, 133.0, 132.7, 132.3, 128.2, 127.4, 126.7, 126.6, 126.6, 126.5, 124.7, 122.0, 119.8, 119.3.
^{19}F NMR (282 MHz, DMSO) δ -61.71.
HRMS (ESI+) m/z [M+H]$^+$ calcd. for $C_{13}H_{10}F_3N_3O_2S_1$ 330.0524, found 330.0532.

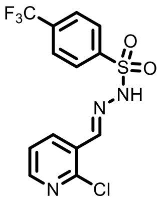

N'-((2-chloropyridin-3-yl)methylene)-4-(trifluoromethyl)benzenesulfonohydrazide (**1z**) Prepared according to GP1 using 4-(trifluoromethyl)benzenesulfonohydrazide (360 mg, 1.5 mmol, 1.0 equiv.) and 2-chloronicotinaldehyde (212 mg, 1.5 mmol, 1.0 equiv.). Isolated as a white solid after filtration (450 mg, 83% yield).

^{1}H NMR (400 MHz, $CDCl_3$) δ 9.43 (s, 1H), 8.41 (dd, *J* = 5, 2 Hz, 1H), 8.22 (dd, *J* = 8, 2 Hz, 1H), 8.18 (s, 1H), 8.12 (d, *J* = 8 Hz, 2H), 7.79 (d, *J* = 8 Hz, 2H), 7.30 (dd, *J* = 8, 5 Hz, 1H).

^{13}C NMR (101 MHz, $CDCl_3$) δ 151.0, 150.2, 142.7, 141.8, 136.3, 135.3 (q, *J* = 32 Hz), 128.6, 127.9, 126.5 (q, *J* = 4 Hz) 123.3, 123.2 (q, *J* = 272 Hz).
^{19}F NMR (282 MHz, $CDCl_3$) δ -63.20.
HRMS (ESI+) (m/z): $[M+H]^+$ calcd. for $C_{13}H_9ClF_3N_3O_2S$, 364.0134; found: 364.0133.

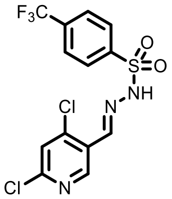

N'-((4,6-dichloropyridin-3-yl)methylene)-4-(trifluoromethyl) **(1aa).** Prepared according to GP1 from 4,6-dichloronicotinaldehyde (352 mg, 2.0 mmol, 1.0 equiv.) and 4-(trifluoromethyl)benzenesulfonohydrazide (480 g, 2.0 mmol, 1.0 equiv.). Isolated as a yellow solid after filtration (595 mg, 90% yield).

^{1}H NMR (400 MHz, DMSO) δ 12.30 (s, 1H), 8.66 (s, 1H), 8.16 (s, 1H), 8.13 (d, *J* = 8 Hz, 2H), 8.01 (d, *J* = 8 Hz, 2H), 7.84 (s, 1H).
^{13}C NMR (101 MHz, DMSO) δ 151.3, 147.7, 143.9, 142.5 (2C), 140.6, 132.9 (q, *J* = 33 Hz), 128.3, 126.7 (q, *J* = 4 Hz), 124.8, 123,4 (q, *J* = 273 Hz).
^{19}F NMR (282 MHz, DMSO) δ -61.75.
HRMS (ESI+) m/z $[M+H]^+$ calcd. for $C_{13}H_8Cl_2F_3N_3O_2S_1$ 397.9745, found 397.9744.

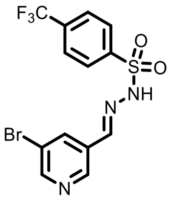

N'-((5-bromopyridin-3-yl)methylene)-4-(trifluoromethyl)benzenesulfonohydrazide (**1ab**). Prepared according to GP1 using 4-(trifluoromethyl)benzenesulfonohydrazide (360 mg, 1.5 mmol, 1.0 equiv.) and 5-bromonicotinaldehyde (279 mg, 1.5 mmol, 1.0 equiv.). Isolated as a white solid after filtration (563 mg, 92% yield).

^{1}H NMR (400 MHz, DMSO) δ 12.18 (s, 1H), 8.77 – 8.71 (m, 1H), 8.69 – 8.63 (m, 1H), 8.15 (d, *J* = 4 Hz, 2H), 8.12 (s, 1H), 8.01 – 7.96 (m, 3H).
^{13}C NMR (101 MHz, DMSO) δ 151.2, 146.6, 143.6, 142.6, 135.6, 132.8 (q, *J* = 32 Hz), 131.2, 128.2, 126.6 (q, *J* = 4 Hz), 123.3 (q, *J* = 276 Hz), 120.4.
^{19}F NMR (282 MHz, DMSO) δ -61.82.
HRMS (ESI+) (m/z): $[M+H]^+$ calcd. for $C_{13}H_9BrF_3N_3O_2S$, 409.9609; found: 409.9596.

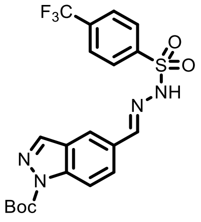

tert-butyl-6-((2-((4-(trifluoromethyl)phenyl)sulfonyl)hydrazineylidene)methyl)-1H-indazole-1-carboxylate **(1ac).** Prepared according to GP1 from tert-butyl 6-formyl-1H-indazole-1-carboxylate (246 mg, 1.0 mmol, 1.0 equiv.) and 4-(trifluoromethyl)benzenesulfonohydrazide (240 mg, 1.0 mmol, 1.0 equiv.). Isolated as a white solid after filtration (460 mg, 98% yield).

^{1}H NMR (400 MHz, $CDCl_3$) δ 9.6 (s, 1H), 8.3 (s, 1H), 8.2 – 8.1 (m, 4H), 7.7 (d, *J* = 8 Hz, 2H), 7.7 (s, 2H), 1.7 (s, 9H).
^{13}C NMR (101 MHz, $CDCl_3$) δ 149.1, 148.3, 142.0 (d, *J* = 2 Hz), 139.7, 139.5, 134.8 (q, *J* = 33 Hz), 134.3, 128.5, 126.9, 126.2 (q, *J* = 4 Hz), 123.2 (q, *J* = 273 Hz), 122.1, 121.6, 114.6, 85.8, 28.1.
^{19}F NMR (282 MHz, CDCl3) δ -63.22.
HRMS (ESI+) m/z $[M+H]^+$ calcd. for $C_{20}H_{19}F_3N_4O_4S_1$ 469.1157, found 469.1155.

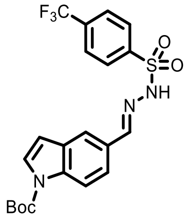

tert-butyl-5-((2-((4-(trifluoromethyl)phenyl)sulfonyl)hydrazineylidene)methyl)-1H-indole-1-carboxylate **(1ad).** Prepared according to GP1 from tert-butyl 5-formyl-1H-indole-1-carboxylate (367 mg, 1.5 mmol, 1.0 equiv.) and 4-(trifluoromethyl)benzenesulfonohydrazide (360 mg, 1.5 mmol, 1.0 equiv.). Isolated as a yellow solid after filtration (670 mg, 96% yield).

^{1}H NMR (400 MHz, DMSO) δ 11.78 (s, 1H), 8.13 (d, *J* = 8 Hz, 2H), 8.09 – 7.98 (m, 4H), 7.81 (d, *J* = 2 Hz, 1H), 7.69 (d, *J* = 4 Hz, 1H), 7.58 (dd, *J* = 9, 2 Hz, 1H), 6.74 (d, *J* = 4 Hz, 1H), 1.61 (s, 9H).
^{13}C NMR (101 MHz, DMSO) δ 148.8, 148.7, 143.0, 135.5, 132.6 (q, *J* = 32 Hz), 130.4, 128.3, 128.2, 127.2, 126.5 (q, *J* = 4 Hz), 123.4 (q, *J* = 273 Hz), 122.6, 120.7, 115.0, 107.7, 84.2, 27.6.
^{19}F NMR (282 MHz, DMSO) δ -61.67.
HRMS (ESI+) m/z $[M+H]^+$ calcd. for $C_{21}H_{20}F_3N_3O_4S_1$ 468.1205, found 468.1203.

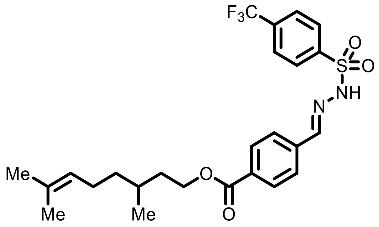

3,7-dimethyloct-6-en-1-yl-4-((2-((4-(trifluoromethyl)phenyl)sulfonyl)hydrazineylidene)methyl)benzoate **(1ae).** Prepared according to GP1 from 3,7-dimethyloct-6-en-1-yl 4-formylbenzoate (432 mg, 1.5 mmol, 1.0 equiv.) and 4-(trifluoromethyl)benzenesulfonohydrazide (360 mg, 1.5 mmol, 1.0 equiv.). Isolated as a white solid after filtration (450 mg, 59% yield).

^{1}H NMR (400 MHz, CDCl3) δ 8.42 (s, 1H), 8.14 (d, *J* = 8 Hz, 2H), 8.02 (d, *J* = 8 Hz, 2H), 7.83 – 7.76 (m, 3H), 7.64 (d, *J* = 8 Hz, 2H), 5.09 (tt, *J* = 7, 1 Hz, 1H), 4.42 – 4.29 (m, 2H), 2.10 – 1.90 (m, 2H), 1.87 – 1.74 (m, 1H), 1.71 – 1.52 (m, 8H), 1.46 – 1.31 (m, 1H), 1.31 – 1.16 (m, 1H), 0.96 (d, *J* = 6 Hz, 3H).

^{13}C NMR (101 MHz, CDCl3) δ 166.1, 147.0, 141.7, 136.9, 135.3 (q, *J* = 33 Hz), 132.3, 131.6, 130.1, 128.6, 127.4, 126.5 (q, *J* = 4 Hz), 124.6, 123.3 (q, *J* = 273 Hz), 64.0, 37.1, 35.6, 29.7, 25.8, 25.5, 19.6, 17.8.
^{19}F NMR (282 MHz, CDCl3) δ -63.22.
HRMS (ESI+) m/z $[M+H]^+$ calcd. for $C_{25}H_{29}F_3N_2O_4S_1$ 511.1878, found 511.1881.

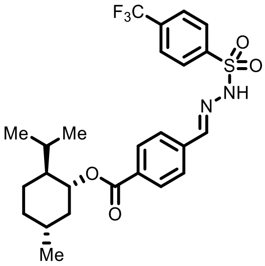

***(1R,2S,5R)-2-isopropyl-5-methylcyclohexyl 4-((2-((4-(trifluoromethyl)phenyl)sulfonyl)hydrazineylidene)methyl) benzoate* (1af).** Prepared according to GP1 from (1R,2S,5R)-2-isopropyl-5-methylcyclohexyl 4-formylbenzoate (432 mg, 1.5 mmol, 1.0 equiv.) and 4-(trifluoromethyl)benzenesulfonohydrazide (360 mg, 1.5 mmol, 1.0 equiv.). Isolated as a white solid after filtration and flash-chromatography over silica, eluting with hexane:ethyl acetate (3:1)(489 mg, 64% yield).

^{1}H NMR (400 MHz, CDCl3) δ 8.65 (s, 1H), 8.13 (d, *J* = 8 Hz, 2H), 8.02 (d, *J* = 8 Hz, 2H), 7.84 (s, 1H), 7.79 (d, *J* = 8 Hz, 2H), 7.63 (d, *J* = 8 Hz, 2H), 4.92 (td, *J* = 11, 4 Hz, 1H), 2.13 – 2.06 (m, 1H), 1.91 (hd, *J* = 7, 3 Hz, 1H), 1.78 – 1.68 (m, 2H), 1.61 – 1.48 (m, 2H), 1.20 – 1.03 (m, 2H), 0.91 (dd, *J* = 7, 4 Hz, 6H), 0.78 (d, *J* = 7 Hz, 3H).
^{13}C NMR (101 MHz, CDCl3) δ 165.6, 147.2, 141.7, 136.8, 135.2 (q, *J* = 33 Hz), 132.6, 130.1, 128.6, 127.3, 126.4 (q, *J* = 4 Hz), 123.2 (q, *J* = 273 Hz), 75.5, 47.3, 41.0, 34.4, 31.6, 26.7, 23.7, 22.1, 20.9, 16.6.
^{19}F NMR 19F NMR (282 MHz, CDCl3) δ = -63.22.
HRMS (ESI+) m/z $[M+H]^+$ calcd. for $C_{25}H_{29}F_3N_2O_4S_1$ 511.1878, found 511.1881.

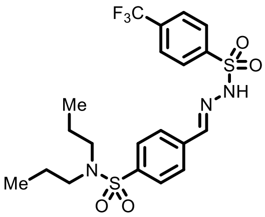

***N,N-dipropyl-4-((2-((4-(trifluoromethyl)phenyl)sulfonyl)hydrazineylidene)methyl)benzenesulfonamide* (1ag).** Prepared according to GP1 from 4-formyl-N,N-dipropylbenzenesulfonamide (404 mg, 1.5 mmol, 1.0 equiv.) and 4-(trifluoromethyl)benzenesulfonohydrazide (360 mg, 1.5 mmol, 1.0 equiv.). Isolated as a white solid after filtration (713 mg, 97% yield).

^{1}H NMR (400 MHz, CDCl3) δ 8.94 (s, 1H), 8.13 (d, *J* = 8 Hz, 2H), 7.82 (s, 1H), 7.80 – 7.77 (m, 2H), 7.75 (d, *J* = 8 Hz, 2H), 7.65 (d, *J* = 8 Hz, 2H), 3.10 – 3.02 (m, 4H), 1.52 (h, *J* = 7 Hz, 4H), 0.84 (t, *J* = 7 Hz, 6H).
^{13}C NMR (101 MHz, CDCl3) δ 146.2, 141.8 (q, *J* = 2 Hz), 141.6, 136.8, 135.2 (q, *J* = 33 Hz), 128.6, 127.9, 127.5, 126.4 (q, *J* = 4 Hz), 123.3 (q, *J* = 273 Hz), 50.0, 22.0, 11.2.
^{19}F NMR (282 MHz, CDCl3) δ -63.22.
HRMS (ESI+) m/z $[M+H]^+$ calcd. for $C_{20}H_{24}F_3N_3O_4S_2$ 492.1239, found 492.1230.

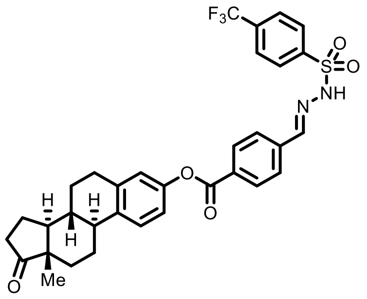

13-methyl-17-oxo-7,8,9,11,12,13,14,15,16,17-decahydro-6H-cyclopenta[a]phenanthren-3-yl-4-((2-((4-(trifluoromethyl)phenyl)sulfonyl)hydrazineylidene)methyl)benzoate **(1ah).** Prepared according to GP1 from 13-methyl-17-oxo-7,8,9,11,12,13,14,15,16,17-decahydro-6H-cyclopenta[a]phenanthren-3-yl 4-formylbenzoate (603 mg, 1.5 mmol, 1.0 equiv.) and 4-(trifluoromethyl)benzenesulfonohydrazide (360 mg, 1.5 mmol, 1.0 equiv.). Isolated as a white solid after filtration and flash-chromatography over silica, eluting with pentane:ethyl acetate (5:1 to 1:1)(610 mg, 65% yield).

^{1}H NMR (400 MHz, $CDCl_3$) δ 8.15 (dd, *J* = 8, 6 Hz, 4H), 7.86 (s, 1H), 7.78 (d, *J* = 8 Hz, 2H), 7.67 (d, *J* = 8 Hz, 2H), 7.33 (d, *J* = 9 Hz, 1H), 6.97 (dd, *J* = 8, 3 Hz, 1H), 6.93 (d, *J* = 2 Hz, 1H), 2.97 – 2.89 (m, 2H), 2.59 – 2.47 (m, 1H), 2.42 (dt, *J* = 13, 4 Hz, 1H), 2.30 (td, *J* = 11, 4 Hz, 1H), 2.24 – 1.87 (m, 4H), 1.71 – 1.38 (m, 7H), 0.92 (s, 3H).
^{13}C NMR (101 MHz, $CDCl_3$) δ 165.0, 148.8, 146.7, 142.0, 138.3, 137.8, 137.8, 135.07 (q, *J* = 33 Hz), 131.2, 130.6, 128.6, 127.4, 126.7, 126.37 (q, *J* = 4 Hz), 123.25 (q, *J* = 273 Hz), 121.7, 118.9, 106.9, 50.6, 48.1, 44.3, 40.2, 38.1, 36.0, 31.7, 29.5, 26.4, 25.9, 21.7, 14.0.
^{19}F NMR (282 MHz, CDCl3) δ -63.21.
HRMS (ESI+) m/z $[M+H]^+$ calcd. for $C_{33}H_{31}F_3N_2O_5S_1$ 625.1984, found 625.1973.

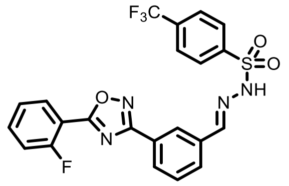

N'-(3-(5-(2-fluorophenyl)-1,2,4-oxadiazol-3-yl)benzylidene)-4-(trifluoromethyl)benzenesulfonohydrazide (1ai). Prepared according to GP1 from 3-(5-(2-fluorophenyl)-1,2,4-oxadiazol-3-yl)benzaldehyde (600 mg, 2.2 mmol, 1.0 equiv.) and 4-(trifluoromethyl)benzenesulfonohydrazide (537 mg, 2.2 mmol, 1.0 equiv.). Isolated as a white solid after filtration (965 mg, 88% yield).
^{1}H NMR (400 MHz, DMSO) δ 12.07 (s, 1H), 8.28 – 8.18 (m, 2H), 8.16 – 8.06 (m, 4H), 8.02 (d, *J* = 8 Hz, 2H), 7.85 – 7.74 (m, 2H), 7.63 (t, *J* = 8 Hz, 1H), 7.58 – 7.45 (m, 2H).
^{13}C NMR (101 MHz, DMSO) δ 172.7 (d, *J* = 4 Hz), 167.6, 161.3, 158.7, 147.1, 142.9, 135.8 (d, *J* = 9 Hz), 134.5, 132.8 (q, *J* = 32 Hz), 130.9, 130.0 (d, *J* = 3 Hz), 128.7, 128.2, 126.6 (q, *J* = 5 Hz), 126.6, 125.5 (d, *J* = 4 Hz), 125.3, 123.4 (q, *J* = 273 Hz), 117.3 (d, *J* = 21 Hz), 111.7 (d, *J* = 11 Hz).
^{19}F NMR (282 MHz, DMSO) δ -61.70, -109.47.
HRMS (ESI+) m/z $[M+H]^+$ calcd. for $C_{22}H_{14}F_4N_4O_3S_1$ 491.0801, found 491.0794.

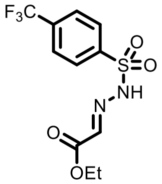

ethyl-2-(2-((4-(trifluoromethyl)phenyl)sulfonyl)hydrazineylidene)acetate (1aj). To a 0.3 M solution of 4-(trifluoromethyl)benzenesulfonohydrazide (1.20 g, 5.00 mmol, 1.0 equiv.) in ethanol, ethyl glyoxylate (50% soln. toluene, 1.02 g, 1.02 mL, 5.0 mmol, 1.0 equiv.) was added dropwise. The reaction mixture was stirred for 1.5 h, then the solvent was removed under vacuum. The resulting solid was washed with Hexane:Ethyl Acetate 10:1 and isolated as a white solid after filtration (1.45 g, 89% yield).

^{1}H NMR (400 MHz, $CDCl_3$) δ 12.2 (s, 1H), 8.1 (d, J = 8 Hz, 2H), 7.8 (d, J = 8 Hz, 2H), 6.8 (s, 1H), 4.3 (q, J = 7 Hz, 2H), 1.32 (t, J = 7 Hz, 3H).
^{13}C NMR (101 MHz, $CDCl_3$) δ 161.8, 141.9, 135.5 (q, J = 33 Hz), 129.2, 128.6, 126.5 (q, J = 4 Hz), 123.2 (q, J = 273 Hz), 62.2, 14.0.
^{19}F NMR (282 MHz, $CDCl_3$) δ -63.28.
HRMS (ESI+) m/z $[M+H]^+$ calcd. for $C_{11}H_{11}F_3N_2O_4S$ 325.0470, found 325.0467.

12.2 Characterization of hydrazide intermediate 2a

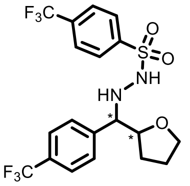

***N'-((tetrahydrofuran-2-yl)(4-(trifluoromethyl)phenyl)methyl)-4-(trifluoromethyl)benzenesulfonohydrazide* (2a).** Prepared according to GP2 from **1a** (159 mg, 0.40 mmol, 1.0 equiv.) without performing the subsequent base-promoted cleavage. The vial was removed from the photochemical reactor and the solvent was evaporated under reduced pressure. *d.r.* ratio was determined via ^{1}H NMR of the reaction crude to be ca. 1:1. The obtained reaction crude was purified via flash column chromatography on silica gel (from Pentane:Ethyl Acetate 90:10 to 80:20) to afford the two diastereomers as an inseparable mixture as colorless oil (120 mg, 64% yield).
*N.B. **2a** is unstable right after isolation. Therefore a full characterization of the pure compound was not possible to achieve. After 24 h ^{1}H NMR analysis showed completely decomposition of the **2a**.*

^{1}H NMR (400 MHz, $CDCl_3$) δ 7.99 (d, J = 8 Hz, 2H), 7.90 (d, J = 8 Hz, 2H), 7.74 (d, J = 8 Hz, 2H), 7.69 (d, J = 8 Hz, 2H), 7.51 (d, J = 8 Hz, 2H), 7.44 (d, J = 8 Hz, 2H), 7.29 (d, J = 8 Hz, 2H), 7.20 (d, J = 8 Hz, 2H), 6.45 (s, 1H), 6.27 (s, 1H), 4.57 – 4.16 (m, 2H), 4.17 – 4.06 (m, 1H), 4.02 (d, J = 4 Hz, 1H), 3.92 – 3.83 (m, 2H), 3.77 (dd, J = 7.7, 5.0 Hz, 2H), 3.67 (t, J = 6 Hz, 2H), 1.92 – 1.69 (m, 4H), 1.66 – 1.38 (m, 4H).
^{19}F NMR (282 MHz, $CDCl_3$) δ -62.67, -62.69, -63.20, -63.21.
HRMS (ESI+) m/z $[M+H]^+$ calcd. for $C_{19}H_{18}F_6N_2O_3S$, 469.1021, found 469.1030.

12.3 Characterization of compounds 3 – 34

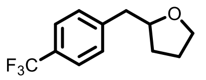

***2-(4-(trifluoromethyl)benzyl)tetrahydrofuran* (3).** Prepared according to GP2 from **1a** (163 mg, 0.4 mmol, 1.0 equiv.). Purified via flash column chromatography on silica gel (Hexane:Ethyl Acetate 40:1) to afford the product as a colorless oil (74 mg, 80% yield).

Characterization data are in accordance with literature.[25]

^{1}H NMR (400 MHz, $CDCl_3$) δ 7.5 (d, *J* = 8 Hz, 2H), 7.3 (d, *J* = 8 Hz, 2H), 4.1 (p, *J* = 6 Hz, 1H), 3.93 – 3.85 (m, 1H), 3.7 (q, *J* = 8 Hz, 1H), 2.9 (dd, *J* = 14, 7 Hz, 1H), 2.8 (dd, *J* = 14, 6 Hz, 1H), 2.0 – 1.9 (m, 1H), 1.9 – 1.8 (m, 2H), 1.6 (dt, *J* = 11, 8 Hz, 1H).
^{13}C NMR (101 MHz, $CDCl_3$) δ 143.3, 129.7, 128.6 (q, *J* = 32 Hz), 125.3 (q, *J* = 4 Hz), 124.5 (q, *J* = 272 Hz), 79.6, 68.2, 41.8, 31.2, 25.7.
^{19}F NMR (282 MHz, $CDCl_3$) δ -62.38.

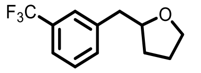

***2-(3-(trifluoromethyl)benzyl)tetrahydrofuran* (4).** Prepared according to GP2 from **1h** (159 mg, 0.4 mmol, 1.0 equiv.). Purified via flash column chromatography on silica gel (Hexane:Ethyl Acetate 30:1) to afford the product as a colorless oil (57 mg, 62% yield).

^{1}H NMR (400 MHz, CDCl3) δ 7.51 – 7.36 (m, 4H), 4.08 (tt, *J* = 8, 6 Hz, 1H), 3.89 (dt, *J* = 8, 7 Hz, 1H), 3.74 (dt, *J* = 8, 7 Hz, 1H), 2.93 (dd, *J* = 14, 7 Hz, 1H), 2.84 (dd, *J* = 14, 6, 1H), 2.01 – 1.81 (m, 3H), 1.61 – 1.49 (m, 1H).
^{13}C NMR (101 MHz, CDCl3) δ 140.1, 132.8, 130.7 (q, *J* = 32 Hz), 128.8, 126.1 (q, *J* = 4 Hz), 124.4 (q, J = 272 Hz), 123.2 (q, *J* = 4), 79.6, 68.2, 41.8, 31.2, 25.8.
^{19}F NMR (282 MHz, CDCl3) δ -62.53.
HRMS (FI+) m/z $[M\text{-}C_4H_7O]^+$ calcd. for $C_{12}H_{13}F_3O$ 159.0422, found 159.0425, m/z $[M\text{-}C_8H_6F_3]^+$ calcd. for $C_{12}H_{13}F_3O$ 71.0497, found 71.0493.

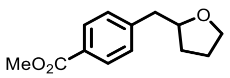

***ethyl 4-((tetrahydrofuran-2-yl)methyl)benzoate* (5).** Prepared according to GP2 from **1i** (155 mg, 0.4 mmol, 1.0 equiv.). Purified via flash column chromatography on silica gel (Hexane:Ethyl Acetate 95:5) to afford the product as a colorless oil (50 mg, 57% yield).

^{1}H NMR (400 MHz, $CDCl_3$) δ 8.01 – 7.90 (m, 2H), 7.33 – 7.26 (m, 2H), 4.08 (dq, *J* = 8, 6 Hz, 1H), 3.89 (s, 4H), 3.82 – 3.66 (m, 1H), 2.93 (dd, *J* = 14, 7 Hz, 1H), 2.81 (dd, *J* = 14, 6 Hz, 1H), 2.01 – 1.75 (m, 3H), 1.63 – 1.45 (m, 1H).
^{13}C NMR (101 MHz, $CDCl_3$) δ 167.2, 144.6, 129.7, 129.4, 128.3, 79.6, 68.1, 52.1, 42.0, 31.1, 25.7.
HRMS (FI+) (m/z): $[M]^+$ calcd. for $C_{13}H_{16}O_3$, 220.1099; found: 71.0476 $[M\text{-}C_9H_9O_2]$; 149.0568 $[M\text{-}C_4H_7O]$

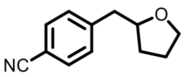

***4-((tetrahydrofuran-2-yl)methyl)benzonitrile* (6).** Prepared according to GP2 from **1j** (141 mg, 0.4 mmol, 1.0 equiv.). Purified via flash column chromatography on silica gel (from Hexane:Ethyl Acetate 90:10 to 80:20) to afford the product as a colorless oil (45 mg, 60% yield).

^{1}H NMR (400 MHz, $CDCl_3$) δ 7.62 – 7.53 (m, 2H), 7.39 – 7.31 (m, 2H), 4.06 (tt, J = 8, 6 Hz, 1H), 3.87 (dt, J = 8, 7 Hz, 1H), 3.73 (dt, J = 8, 7 Hz, 1H), 2.96 – 2.80 (m, 2H), 2.03 – 1.92 (m, 1H), 1.91 – 1.82 (m, 2H), 1.60 – 1.47 (m, 1H).
^{13}C NMR (101 MHz, $CDCl_3$) δ 144.9, 132.2, 130.2, 119.2, 110.2, 79.3, 68.1, 42.1, 31.3, 25.7.
HRMS (ESI+) (m/z): $[M]^+$ calcd. for $C_{12}H_{13}NO$, 187.0997; found: 71.0450 $[M-C_8H_6N]$; 116.0447 $[M-C_4H_7O]$

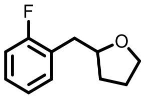

***2-(2-fluorobenzyl)tetrahydrofuran* (7).** Prepared according to GP2 from **1k** (139 mg, 0.4 mmol, 1.0 equiv.). Purified via flash column chromatography on silica gel (from Pentane to Pentane:Ethyl Acetate 99:1) to afford the product as a colorless oil (38 mg, 53% yield).

^{1}H NMR (300 MHz, $CDCl_3$) δ 7.33 – 7.25 (m, 1H), 7.25 – 7.15 (m, 1H), 7.15 – 6.97 (m, 2H), 4.21 – 4.06 (m, 1H), 3.92 (ddd, J = 8.2, 7.1, 6.1 Hz, 1H), 3.83 – 3.70 (m, 1H), 3.00 – 2.79 (m, 2H), 2.05 – 1.77 (m, 3H), 1.73 – 1.47 (m, 1H).
^{13}C NMR (75 MHz, $CDCl_3$) δ 161.3 (d, J = 245 Hz), 131.7 (d, J = 5 Hz), 128.0 (d, J = 8 Hz), 126.0 (d, J = 16 Hz), 124.0 (d, J = 4 Hz), 115.3 (d, J = 22 Hz), 79.0 (d, J = 1 Hz), 68.1, 34.87 (d, J = 1.5 Hz), 31.1, 25.7.
^{19}F NMR (282 MHz, $CDCl_3$) δ -118.19.
HRMS (FI+) (m/z): $[M]^+$ calcd. for $C_{11}H_{13}FO$, 180.0950; found: 180.0948.

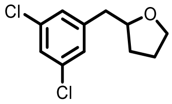

***2-(3,5-dichlorobenzyl)tetrahydrofuran* (8).** Prepared according to GP2 from **1l** (159 mg, 0.4 mmol, 1.0 equiv.). Purified via flash column chromatography on silica gel (Hexane:Ethyl Acetate 98:2) to afford the product as a colorless oil (48 mg, 52% yield).

^{1}H NMR (400 MHz, $CDCl_3$) δ 7.21 (t, J = 2 Hz, 1H), 7.15 – 7.11 (m, 2H), 4.04 (tt, J = 8, 6 Hz, 1H), 3.88 (ddd, J = 8, 7, 6 Hz, 1H), 3.74 (ddd, J = 8, 7, 6 Hz, 1H), 2.78 (d, J = 7 Hz, 1H), 2.75 (d, J = 5 Hz, 1H), 2.02 – 1.92 (m, 1H), 1.91 – 1.78 (m, 2H), 1.59 – 1.46 (m, 1H).
^{13}C NMR (101 MHz, $CDCl_3$) δ 142.6, 134.8, 127.9, 126.6, 79.3, 68.2, 41.4, 31.2, 25.7.
HRMS (ESI+) (m/z): $[M+H]^+$ calcd. for $C_{11}H_{12}Cl_2O$, 231.0343; found: 231.0378.

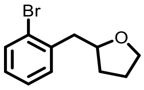

***2-(2-bromobenzyl)tetrahydrofuran* (9).** Prepared according to GP2 from **1m** (163 mg, 0.4 mmol, 1.0 equiv.). Purified via flash column chromatography on silica gel (from Hexane:Ethyl Acetate 99:1 to 98:2) to afford the product as a colorless oil (45 mg, 47% yield).

^{1}H NMR (400 MHz, $CDCl_3$) δ 7.53 (dd, *J* = 8, 1 Hz, 1H), 7.31 (dd, *J* = 8, 2 Hz, 1H), 7.24 (td, *J* = 7, 1 Hz, 1H), 7.11 – 7.02 (m, 1H), 4.17 (tt, *J* = 7, 6 Hz, 1H), 3.99 – 3.84 (m, 1H), 3.80 – 3.70 (m, 1H), 3.01 (dd, *J* = 14, 7 Hz, 1H), 2.94 (dd, *J* = 14, 6 Hz, 1H), 2.03 – 1.79 (m, 3H), 1.69 – 1.56 (m, 1H).
^{13}C NMR (101 MHz, $CDCl_3$) δ 138.6, 132.8, 131.5, 128.0, 127.5, 124.9, 78.5, 68.0, 41.8, 31.2, 25.7.
HRMS (ESI+) (m/z): $[M+H]^+$ calcd. for $C_{11}H_{13}BrO$, 241.0228; found: 241.0164.

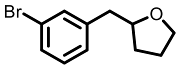

***2-(3-bromobenzyl)tetrahydrofuran* (10).** Prepared according to GP2 from **1n** (163 mg, 0.4 mmol, 1.0 equiv.). Purified via flash column chromatography on silica gel (Hexane:Ethyl Acetate 30:1) to afford the product as a yellowish oil (61 mg, 63% yield).

^{1}H NMR (400 MHz, $CDCl_3$) δ 7.40 – 7.37 (m, 1H), 7.35 – 7.30 (m, 1H), 7.18 – 7.13 (m, 2H), 4.05 (dt, *J* = 14 Hz, 1H), 3.88 (dt, *J* = 9 Hz, 1H), 3.77 – 3.70 (m, 1H), 2.85 (dd, *J* = 15 Hz, 1H), 2.73 (dd, *J* = 15 Hz, 1H), 2.02 – 1.80 (m, 3H), 1.53 (dq, *J* = 12 Hz, 1H).
^{13}C NMR (101 MHz, $CDCl_3$) δ 141.5, 132.3, 130.0, 129.4, 128.0, 122.5, 79.7, 68.1, 41.6, 31.2, 25.7.
HRMS (ESI+) (m/z): $[M+H]^+$ calcd. for $C_{11}H_{13}BrO$, 241.0228; found: 241.0229.

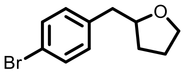

***2-(4-bromobenzyl)tetrahydrofuran* (11).** Prepared according to GP2 from **1o** (163 mg, 0.4 mmol, 1.0 equiv.). Purified via flash column chromatography on silica gel (Hexane:Ethyl Acetate 30:1) to afford the product as a colorless oil (62 mg, 64% yield).

Characterization data are in accordance with literature.[26]
^{1}H NMR (400 MHz, $CDCl_3$) δ 7.40 (d, *J* = 8 Hz, 2H), 7.11 (d, *J* = 8 Hz, 2H), 4.06-3.99 (m, 1H), 3.91-3.84 (m, 1H), 3.76-3.69 (m, 1H), 2.83 (dd, *J* = 15 Hz, 1H), 2.72 (dd, *J* = 15 Hz, 1H), 2.00 – 1.77 (m, 3H), 1.59 – 1.47 (m, 1H).
^{13}C NMR (101 MHz, $CDCl_3$) δ 138.1, 131.5, 131.1, 120.2, 79.8, 68.1, 41.4, 31.1, 25.7.

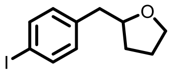

***2-(4-iodobenzyl)tetrahydrofuran* (12).** Prepared according to GP2 from **1p** (182 mg, 0.4 mmol, 1.0 equiv.). Purified via flash column chromatography on silica gel (from Hexane:Ethyl Acetate 99:1 to 98:2) to afford the product as a colorless oil (50 mg, 43% yield).

^{1}H NMR (400 MHz, $CDCl_3$) δ 7.57 – 7.49 (m, 2H), 6.96 – 6.87 (m, 2H), 3.95 (dq, *J* = 8, 6 Hz, 1H), 3.80 (dt, *J* = 8, 7 Hz, 1H), 3.65 (td, *J* = 8, 6 Hz, 1H), 2.74 (dd, *J* = 14, 7 Hz, 1H), 2.64 (dd, *J* = 14, 6 Hz, 1H), 1.92 – 1.69 (m, 3H), 1.53 – 1.38 (m, 1H).
^{13}C NMR (101 MHz, $CDCl_3$) δ 138.8, 137.4, 131.5, 91.6, 79.8, 68.1, 41.5, 31.1, 25.7.
HRMS (GC-EI+) (m/z): $[M]^+$ calcd. for $C_{11}H_{13}IO$, 288.0011; found: 288.0021.

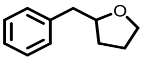

***2-benzyltetrahydrofuran* (13).** Prepared according to GP2 from **1q** (131 mg, 0.4 mmol, 1.0 equiv.). Purified via flash column chromatography on silica gel (Pentane:Ethyl Acetate 30:1) to afford the product as a colorless oil (36 mg, 55% yield).

Characterization data are in accordance with literature.[27]
^{1}H NMR (400 MHz, CDCl3) δ 7.38 – 7.19 (m, 5H), 4.09 (p, *J* = 7 Hz, 1H), 3.96 – 3.88 (m, 1H), 3.81 – 3.71 (m, 1H), 2.95 (dd, *J* = 14, 7 Hz, 1H), 2.77 (dd, *J* = 14, 7 Hz, 1H), 2.02 – 1.79 (m, 3H), 1.65 – 1.51 (m, 1H).
^{13}C NMR (101 MHz, CDCl3) δ 139.1, 129.3, 128.4, 126.3, 80.2, 68.0, 42.1, 31.1, 25.7.

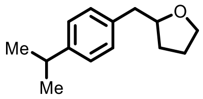

***2-(4-isopropylbenzyl)tetrahydrofuran* (14).** Prepared according to GP2 from **1r** (148 mg, 0.4 mmol, 1.0 equiv.). Purified via flash column chromatography on silica gel (Hexane:Ethyl Acetate 50:1) to afford the product as a colorless oil (70 mg, 86% yield).

^{1}H NMR (400 MHz, $CDCl_3$) δ 7.18 – 7.15 (m, 4H), 4.11 – 4.03 (m, 1H), 3.92 (ddd, *J* = 8, 7, 6 Hz, 1H), 3.81 – 3.70 (m, 1H), 2.90 (ddd, *J* = 14, 7, 2 Hz, 2H), 2.73 (dd, *J* = 14, 6 Hz, 1H), 2.01 – 1.78 (m, 3H), 1.64 – 1.51 (m, 1H), 1.25 (d, *J* = 7 Hz, 6H).
^{13}C NMR (101 MHz, $CDCl_3$) δ 146.7, 136.4, 129.2, 126.5, 80.3, 68.0, 41.7, 33.8, 31.2, 25.7, 24.2, 24.2.
HRMS (FI+) (m/z): $[M]^+$ calcd. for $C_{14}H_{20}O$, 204.1514; found: 204.1520.

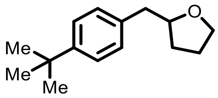

***2-(4-(tert-butyl)benzyl)tetrahydrofuran* (15).** Prepared according to GP2 from **1s** (154 mg, 0.4 mmol, 1.0 equiv.). Purified via flash column chromatography on silica gel (Pentane:Ethyl Acetate 20:1) to afford the product as a colorless oil (72 mg, 82% yield).

^{1}H NMR (400 MHz, CDCl3) δ 7.35 – 7.30 (m, 2H), 7.20 – 7.15 (m, 2H), 4.12 – 4.03 (m, 1H), 3.96 – 3.87 (m, 1H), 3.75 (td, *J* = 8, 6 Hz, 1H), 2.91 (dd, *J* = 14, 7 Hz, 1H), 2.73 (dd, *J* = 14, 6 Hz, 1H), 2.02 – 1.78 (m, 3H), 1.65 – 1.51 (m, 1H), 1.32 (s, 9H).
^{13}C NMR (101 MHz, CDCl3) δ 148.9, 136.0, 128.8, 125.3, 80.2, 67.9, 41.5, 34.4, 31.4, 31.2, 25.6.
HRMS (EI+) m/z $[M]^+$ calcd. for $C_{15}H_{22}O_1$ 218.1671, found 218.1674.

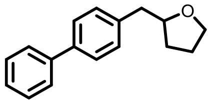

***2-([1,1'-biphenyl]-4-ylmethyl)tetrahydrofuran* (16).** Prepared according to GP2 from **1t** (162 mg, 0.4 mmol, 1.0 equiv.). Purified via flash column chromatography on silica gel (Hexane:Ethyl Acetate 20:1) to afford the product as a white solid (57 mg, 60% yield).

Characterization data are in accordance with literature.[28]
^{1}H NMR (400 MHz, $CDCl_3$) δ 7.60 (dt, J = 8, 2 Hz, 2H), 7.57 – 7.52 (m, 2H), 7.47 – 7.41 (m, 2H), 7.38 – 7.31 (m, 3H), 4.13 (dt, J = 14 Hz, 1H), 3.99 – 3.90 (m, 1H), 3.78 (td, J = 9 Hz, 1H), 2.97 (dd, J = 15 Hz, 1H), 2.82 (dd, J = 15 Hz, 1H), 2.02 – 1.78 (m, 3H), 1.71 – 1.47 (m, 1H).
^{13}C NMR (101 MHz, $CDCl_3$) δ 141.2, 139.2, 138.3, 129.7, 128.8, 127.2, 127.1, 127.1, 80.1, 68.1, 41.7, 31.2, 25.8.

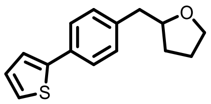

***2-(4-(thiophen-2-yl)benzyl)tetrahydrofuran* (17).** Prepared according to GP2 from **1u** (164 mg, 0.4 mmol, 1.0 equiv.). Purified via flash column chromatography on silica gel (Hexane:Ethyl Acetate 30:1) to afford the product as a colorless oil (70 mg, 72% yield).

^{1}H NMR (400 MHz, CDCl3) δ 7.55 (d, J = 8 Hz, 2H), 7.30 – 7.22 (m, 4H), 7.07 (dd, J = 5, 4 Hz, 1H), 4.14 – 4.03 (m, 1H), 3.91 (dt, J = 8, 7 Hz, 1H), 3.75 (td, J = 8, 6 Hz, 1H), 2.92 (dd, J = 14, 7 Hz, 1H), 2.78 (dd, J = 14, 6 Hz, 1H), 2.05 – 1.76 (m, 3H), 1.63 – 1.53 (m, 1H).
^{13}C NMR (101 MHz, CDCl3) δ 144.6, 138.6, 132.5, 129.8, 128.1, 126.1, 124.5, 122.8, 80.0, 68.1, 41.7, 31.1, 25.7.
HRMS (FD+) m/z $[M]^+$ calcd. for $C_{15}H_{16}O_1S_1$ 244.0922, found 244.0915.

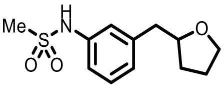

***N-(3-((tetrahydrofuran-2-yl)methyl)phenyl)methanesulfonamide* (18).** Prepared according to GP2 from **1v** (190 mg, 0.45 mmol, 1.0 equiv.). Purified via flash column chromatography on silica gel (Hexane:Ethyl Acetate 30:1) to afford the product as a colorless oil (63 mg, 55% yield).

^{1}H NMR (400 MHz, CDCl3) δ 7.42 (t, J = 8 Hz, 1H), 7.31 – 7.19 (m, 4H), 4.25 (p, J = 7 Hz, 1H), 4.06 (dt, J = 8, 7 Hz, 1H), 3.96 – 3.86 (m, 1H), 3.15 (s, 3H), 3.03 (dd, J = 14, 7 Hz, 1H), 2.93 (dd, J = 14, 6 Hz, 1H), 2.22 – 1.96 (m, 3H), 1.80 – 1.66 (m, 1H).
^{13}C NMR (101 MHz, CDCl3) δ 141.0, 137.0, 129.7, 126.3, 121.6, 118.5, 79.8, 68.0, 41.8, 39.3, 31.2, 25.7.
HRMS (ESI+) m/z $[M+H]^+$ calcd. for $C_{12}H_{17}NO_3S$ 256.1007, found 256.0889.

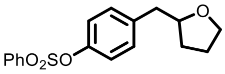

***4-((tetrahydrofuran-2-yl)methyl)phenyl benzenesulfonate* (19).** Prepared according to GP2 from **1w** (194 mg, 0.4 mmol, 1.0 equiv.). Purified via flash column chromatography on silica gel (from Hexane:Ethyl Acetate 95:5 to 90:10) to afford the product as a colorless oil (70 mg, 55% yield).

^{1}H NMR (400 MHz, $CDCl_3$) δ 7.87 – 7.77 (m, 2H), 7.69 – 7.59 (m, 1H), 7.56 – 7.44 (m, 2H), 7.16 – 7.09 (m, 2H), 6.92 – 6.83 (m, 2H), 4.00 (dq, *J* = 8, 6 Hz, 1H), 3.84 (dt, *J* = 8, 7 Hz, 1H), 3.70 (dt, *J* = 8, 7 Hz, 1H), 2.82 (dd, *J* = 14, 7 Hz, 1H), 2.71 (dd, *J* = 14, 6 Hz, 1H), 1.96 – 1.76 (m, 3H), 1.56 – 1.42 (m, 1H).
^{13}C NMR (101 MHz, $CDCl_3$) δ 148.0, 138.3, 135.5, 134.2, 130.4, 129.2, 128.5, 122.1, 79.6, 68.0, 41.2, 31.1, 25.7.
HRMS (FD+) (m/z): $[M]^+$ calcd. for $C_{17}H_{18}O_4S$, 318.0926; found: 318.0930.

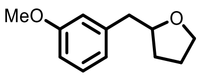

***2-(3-methoxybenzyl)tetrahydrofuran* (20).** Prepared according to GP2 from **1x** (143 mg, 0.4 mmol, 1.0 equiv.). Purified via flash column chromatography on silica gel (Pentane:Dichloromethane 1:1) to afford the product as a colorless oil (38 mg, 49% yield).

^{1}H NMR (400 MHz, CDCl3) δ 7.21 (t, *J* = 8 Hz, 1H), 6.86 – 6.72 (m, 3H), 4.11 – 4.03 (m, 1H), 3.94 – 3.86 (m, 1H), 3.80 (s, 3H), 3.77 – 3.71 (m, 1H), 2.91 (dd, *J* = 14, 6 Hz, 1H), 2.72 (dd, *J* = 14, 6 Hz, 1H), 1.99 – 1.76 (m, 3H), 1.59 – 1.50 (m, 1H).
^{13}C NMR (101 MHz, CDCl3) δ 159.7, 140.7, 129.4, 121.7, 115.1, 111.6, 80.1, 68.1, 55.3, 42.1, 31.1, 25.7.
HRMS (FD+) m/z $[M]^+$ calcd. for $C_{12}H_{16}O_2$ 192.1150, found 192.1148.

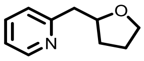

***2-((tetrahydrofuran-2-yl)methyl)pyridine* (21).** Prepared according to GP2 from **1y** (132 mg, 0.4 mmol, 1.0 equiv.). Purified via flash column chromatography on silica gel (Hexane:Ethyl Acetate 20:1) to afford product as a yellowish oil (35 mg, 54% yield).

^{1}H NMR (400 MHz, CDCl3) δ 8.55 – 8.49 (m, 1H), 7.59 (td, *J* = 8, 2 Hz, 1H), 7.25 – 7.21 (m, 1H), 7.11 (ddd, *J* = 8, 5, 1 Hz, 1H), 4.34 – 4.15 (m, 1H), 3.92 – 3.84 (m, 1H), 3.77 – 3.69 (m, 1H), 3.02 (dd, *J* = 14, 7 Hz, 1H), 2.97 (dd, *J* = 14, 6 Hz, 1H), 2.06 – 1.93 (m, 1H), 1.93 – 1.79 (m, 2H), 1.70 – 1.57 (m, 1H).
^{13}C NMR (101 MHz, CDCl3) δ 159.4, 149.2, 136.5, 123.9, 121.5, 78.9, 68.1, 44.3, 31.3, 25.7.
HRMS (ESI+) m/z $[M+H]^+$ calcd. for $C_{10}H_{13}N_1O_1$ 164.1075, found 164.1070.

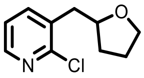

***2-chloro-3-((tetrahydrofuran-2-yl)methyl)pyridine* (22).** Prepared according to GP2 from **1z** (145 mg, 0.4 mmol, 1.0 equiv.). Purified via flash column chromatography on silica gel (from Hexane:Ethyl Acetate 99:1 to 98:2) to afford the product as a colorless oil (40 mg, 51% yield).

^{1}H NMR (400 MHz, $CDCl_3$) δ 8.25 (dd, *J* = 5, 2 Hz, 1H), 7.67 (dd, *J* = 7, 2 Hz, 1H), 7.17 (dd, *J* = 7, 5 Hz, 1H), 4.16 (tdd, *J* = 8, 6, 5 Hz, 1H), 3.88 (ddd, *J* = 8, 7, 6 Hz, 1H), 3.79 – 3.69 (m, 1H), 2.97 (dd, *J* = 14, 5 Hz, 1H), 2.89 (dd, *J* = 14.3, 7.7 Hz, 1H), 2.02 (dddd, *J* = 12, 8, 6, 5 Hz, 1H), 1.96 – 1.81 (m, 2H), 1.59 (ddt, *J* = 12, 8, 7 Hz, 1H).
^{13}C NMR (101 MHz, $CDCl_3$) δ 151.5, 147.6, 140.2, 133.7, 122.6, 77.7, 68.1, 38.8, 31.4, 25.7.
HRMS (ESI+) (m/z): $[M+H]^+$ calcd. for $C_{10}H_{12}ClNO$, 198.0686; found:198.0680.

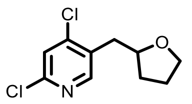

***2,4-dichloro-5-((tetrahydrofuran-2-yl)methyl)pyridine* (23).** Prepared according to GP2 from **1aa** (159 mg, 0.4 mmol, 1.0 equiv.). Purified via flash column chromatography on silica gel (Hexane:Ethyl Acetate 20:1) to afford product as a colorless oil (50 mg, 54% yield).

^{1}H NMR (400 MHz, $CDCl_3$) δ 8.28 (s, 1H), 7.34 (s, 1H), 4.10 (p, *J* = 6 Hz, 1H), 3.93 – 3.80 (m, 1H), 3.78 – 3.68 (m, 1H), 2.94 – 2.86 (m, 2H), 2.05 – 1.95 (m, 1H), 1.94 – 1.82 (m, 2H), 1.61 – 1.51 (m, 1H).
^{13}C NMR (101 MHz, CDCl3) δ 151.6, 149.9, 145.7, 131.9, 124.4, 77.5, 68.1, 35.9, 31.2, 25.7.
HRMS (ESI+) m/z $[M+H]^+$ calcd. for $C_{10}H_{11}Cl_2N_1O_1$ 232.0296, found 232.0291.

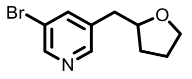

***3-bromo-5-((tetrahydrofuran-2-yl)methyl)pyridine* (24).** Prepared according to GP2 from **1ab** (163 mg, 0.4 mmol, 1.0 equiv.). Purified via flash column chromatography on silica gel (Hexane:Ethyl Acetate 3:1) to afford the product as a colorless oil (48 mg, 50% yield).

^{1}H NMR (400 MHz, $CDCl_3$) δ 8.52 (d, *J* = 2 Hz, 1H), 8.40 – 8.36 (m, 1H), 7.75 (t, *J* = 2 Hz, 1H), 4.11 – 3.97 (m, 1H), 3.87 (dt, *J* = 9 Hz, 1H), 3.78 – 3.67 (m, 1H), 2.79 (d, *J* = 6 Hz, 2H), 1.99 (dq, *J* = 13 Hz, 1H), 1.91 – 1.82 (m, 2H), 1.52 (dq, *J* = 13 Hz, 1H).
^{13}C NMR (101 MHz, $CDCl_3$) δ 148.9, 148.8, 139.5, 136.4, 120.6, 79.0, 68.2, 38.5, 31.2, 25.7.
HRMS (ESI+) (m/z): $[M+H]^+$ calcd. for $C_{10}H_{12}BrNO$, 242.0181; found: 242.0178.

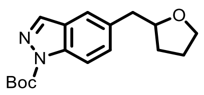

***tert-butyl 5-((tetrahydrofuran-2-yl)methyl)-1H-indazole-1-carboxylate* (25).** Prepared according to GP2 from **1ac** (187 mg, 0.4 mmol, 1.0 equiv.). Purified via flash column chromatography on silica gel (Hexane:Ethyl Acetate 20:1) to afford the product as a colorless oil (43 mg, 36% yield).

^{1}H NMR (400 MHz, CDCl3) δ 8.13 – 8.07 (m, 2H), 7.63 (dd, J = 8 Hz, 0.8, 1H), 7.22 (dd, J = 8, 1 Hz, 1H), 4.20 – 4.09 (m, 1H), 3.94 – 3.86 (m, 1H), 3.79 – 3.70 (m, 1H), 3.05 (dd, J = 14, 7 Hz, 1H), 2.93 (dd, J = 14, 6 Hz, 1H), 2.03 – 1.83 (m, 2H), 1.72 (s, 9H), 1.68 – 1.54 (m, 2H).
^{13}C NMR: (101 MHz, CDCl3) δ 149.5, 140.9, 140.4, 139.6, 125.7, 124.6, 120.8, 114.9, 84.8, 80.0, 68.1, 42.6, 31.3, 28.3, 25.8.
HRMS (FD+) m/z $[M]^+$ calcd. for $C_{17}H_{22}N_2O_3$ 302.1630, found 302.1646.

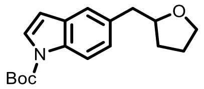

***tert-butyl 5-((tetrahydrofuran-2-yl)methyl)-1H-indole-1-carboxylate* (26).** Prepared according to GP2 from **1ad** (187 mg, 0.4 mmol, 1.0 equiv.). Purified via flash column chromatography on silica gel (Pentane:Dichloromethane 1:1) to afford the product as a colorless oil (32 mg, 27% yield).

^{1}H NMR (400 MHz, CDCl3) δ 8.04 (d, J = 9 Hz, 1H), 7.56 (d, *J* = 4, 1H), 7.41 (s, 1H), 7.18 (dd, *J* = 9, 2 Hz, 1H), 6.52 (d, *J* = 4 Hz, 1H), 4.14 – 4.06 (m, 1H), 3.94 – 3.86 (m, 1H), 3.78 – 3.70 (m, 1H), 3.02 (dd, *J* = 14, 6 Hz, 1H), 2.83 (dd, *J* = 14, 7 Hz, 1H), 1.93 – 1.80 (m, 3H), 1.67 (s, 9H), 1.65 – 1.52 (m, 1H).
^{13}C NMR (101 MHz, CDCl3) δ 149.8, 133.9, 133.3, 130.8, 126.0, 125.7, 121.2, 114.9, 107.2, 83.5, 80.4, 68.0, 41.8, 30.9, 28.2, 25.6.
HRMS (FD+) m/z $[M]^+$ calcd. for $C_{18}H_{23}N_1O_3$ 301.1678, found 301.1667.

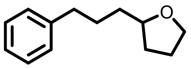

***2-(3-phenylpropyl)tetrahydrofuran* (27).** Prepared according to GP2. Purified via flash column chromatography on silica gel (Hexane:Ethyl Acetate 50:1) to afford the product as a colorless oil (34 mg, 45% yield).

Characterization data are in accordance with literature.[29]
^{1}H NMR (400 MHz, $CDCl_3$) δ 7.23 – 7.17 (m, 2H), 7.13 – 7.08 (m, 3H), 3.82 – 3.70 (m, 2H), 3.67 – 3.60 (m, 1H), 2.57 (t, *J* = 7 Hz, 2H), 2.00 – 1.26 (m, 8H).
^{13}C NMR (101 MHz, $CDCl_3$) δ 142.6, 128.5, 128.4, 125.8, 79.4, 67.8, 36.1, 35.4, 31.5, 28.3, 25.8.

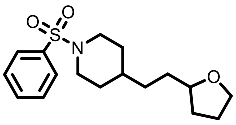

***1-(phenylsulfonyl)-4-(2-(tetrahydrofuran-2-yl)ethyl)piperidine* (28).** Prepared according to GP2. Purified via flash column chromatography on silica gel (Hexane:Ethyl Acetate 20:1) to afford the product as a white solid (53 mg, 41% yield).

^{1}H NMR (400 MHz, $CDCl_3$) δ 7.77 – 7.72 (m, 2H), 7.61-7.56 (m, 1H), 7.54-7.49 (m, 2H), 3.86 – 3.73 (m, 3H), 3.72-3.64 (m, 2H), 2.22 (td, *J* = 12 Hz, 2H), 1.99 – 1.80 (m, 3H), 1.75 – 1.66 (m, 2H), 1.58 – 1.11 (m, 8H).
^{13}C NMR (101 MHz, $CDCl_3$) δ 136.3, 132.7, 129.0, 127.8, 79.4, 67.8, 46.6, 35.3, 32.8, 31.6, 31.6, 31.5, 25.8.
HRMS (FD+) (m/z): $[M+H]^+$ calcd. for $C_{17}H_{25}NO_3S$, 324.1647; found: 324.1633.

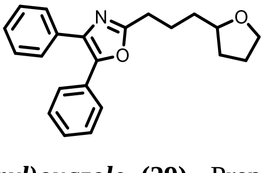

***4,5-diphenyl-2-(3-(tetrahydrofuran-2-yl)propyl)oxazole* (29).** Prepared according to GP2. Purified via flash column chromatography on silica gel (Toluene:Ethyl Acetate 10:1) to afford the product as a yellowish oil (23 mg, 34% yield).

^{1}H NMR (300 MHz, $CDCl_3$) δ 7.67 – 7.61 (m, 2H), 7.61 – 7.55 (m, 2H), 7.40 – 7.29 (m, 6H), 3.98 – 3.79 (m, 2H), 3.77 – 3.66 (m, 1H), 2.90 (t, *J* = 8 Hz, 2H), 2.10 – 1.82 (m, 5H), 1.78 – 1.56 (m, 2H), 1.54 – 1.37 (m, 1H).
^{13}C NMR (101 MHz, $CDCl_3$) δ 163.6, 145.2, 135.2, 132.8, 129.3, 128.7, 128.7, 128.4, 126.6, 79.0, 67.8, 35.2, 31.5, 28.4, 25.8, 24.2.
HRMS (ESI+) m/z $[M+H]^+$ calcd. for $C_{22}H_{23}N_1O_2$ 334.1807, found 334.1799.

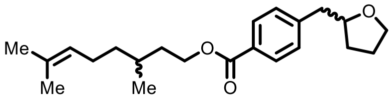

***3,7-dimethyloct-6-en-1-yl 4-((tetrahydrofuran-2-yl)methyl)benzoate* (30).** Prepared according to GP2 from **1ae** (204 mg, 0.4 mmol, 1.0 equiv.). Purified via flash column chromatography on silica gel (Toluene:Ethyl Acetate 60:1) to afford the two diastereomers as an inseparable mixture as a colorless oil (75 mg, 54% yield). *d.r.* ratio of product was determined via chiral HPLC to be 1:1 (Conditions: Daicel Chiralpak OD-H column, 250 x 4.6 mm I.D. 5 µm; eluent: Heptane/Isopropanol 99.7/0.3; flow rate: 1 mL/min; UV @ 254 nm; see attached below).

^{1}H NMR (400 MHz, $CDCl_3$) δ 7.98 – 7.93 (m, 2H), 7.31 – 7.26 (m, 2H), 5.09 (ddq, J = 9, 6, 2 Hz, 1H), 4.39 – 4.28 (m, 2H), 4.07 (dq, J = 8, 6 Hz, 1H), 3.88 (dt, J = 8, 7 Hz, 1H), 3.73 (td, J = 8, 6 Hz, 1H), 2.93 (dd, J = 14, 7 Hz, 1H), 2.82 (dd, J = 14, 6 Hz, 1H), 2.10 – 1.73 (m, 6H), 1.70 – 1.49 (m, 9H), 1.40 (dddd, J = 13, 9, 7, 5 Hz, 1H), 1.23 (dddd, J = 14, 9, 8, 6 Hz, 1H), 0.96 (d, J = 7 Hz, 3H).
^{13}C NMR: (101 MHz, CDCl3) δ 166.8 (*xx*+*yy*), 144.5 (*xx*+*yy*), 131.4 (*xx*+*yy*), 129.7 (*xx*+*yy*), 129.3 (*xx*+*yy*), 128.6 (*xx*+*yy*), 124.7 (*xx*+*yy*), 79.6 (*xx*+*yy*), 68.1 (*xx*+*yy*), 63.4 (*xx*+*yy*), 42.0 (*xx*+*yy*), 37.1 (*xx*+*yy*), 35.6 (*xx*+*yy*), 31.1 (*xx*+*yy*), 29.6 (*xx*+*yy*), 25.8 (*xx*+*yy*), 25.7 (*xx*+*yy*), 25.5 (*xx*+*yy*), 19.6 (*xx*+*yy*), 17.8 (*xx*+*yy*).
HRMS (FI+) m/z $[M]^+$ calcd. for $C_{22}H_{32}O_3$ 344.2351, found 344.2335.

Peak 1: Rt= 11,17 ; Area= 24,64% Area (Peak 1 + Peak 2) = 50,02%
Peak 2: Rt= 11,76 ; Area= 25,38%

Peak 3: Rt= 12,52 ; Area= 24,66% Area (Peak 3 + Peak 4) = 49,98%
Peak 4: Rt= 13,37 ; Area= 25,32%

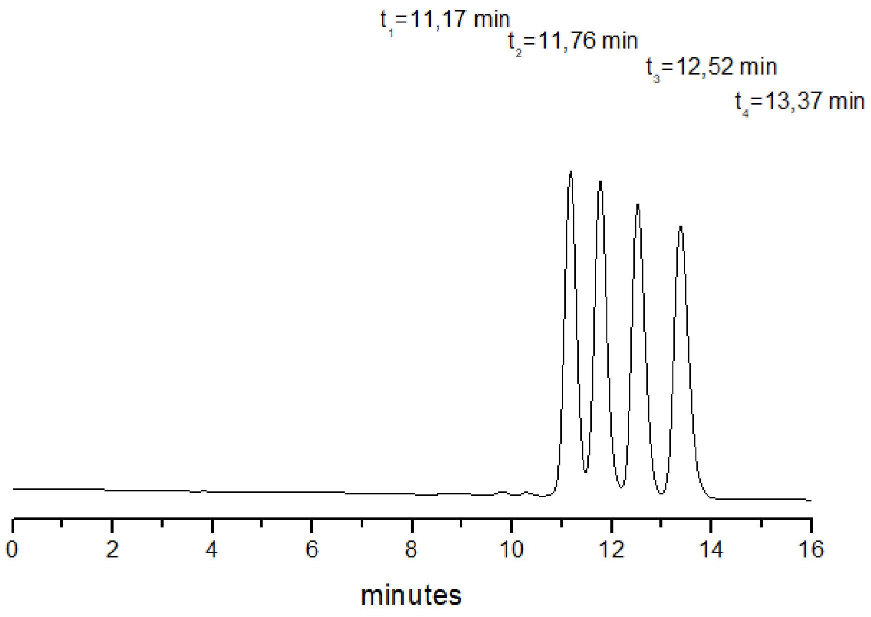

Figure S8: HPLC separation of the two diastereomers of **30**

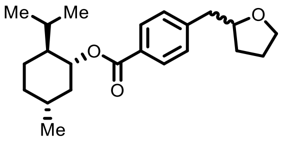

***(1R,2S,5R)-2-isopropyl-5-methylcyclohexyl 4-((tetrahydrofuran-2-yl)methyl)benzoate* (31).** Prepared according to GP2 from **1af** (204 mg, 0.4 mmol, 1.0 equiv.). Purified via flash column chromatography on silica gel (Toluene:Ethyl Acetate 60:1) to afford the two diastereomers as an inseparable mixture as a colorless oil (91 mg, 66% yield). *d.r.* ratio of **31** was determined via ^{13}C NMR to be 1:1.

^{1}H NMR (400 MHz, CDCl3) δ 8.00 – 7.93 (m, 2H), 7.31 – 7.27 (m, 2H), 4.91 (td, *J* = 11, 4 Hz, 1H), 4.12 – 4.00 (m, 1H), 3.92 – 3.82 (m, 1H), 3.77 – 3.67 (m, 1H), 2.92 (ddd, *J* = 14, 7, 2 Hz, 1H), 2.81 (ddd, *J* = 14, 6, 2 Hz, 1H), 2.16 – 2.05 (m, 1H), 2.03 – 1.77 (m, 4H), 1.76 – 1.65 (m, 2H), 1.62 – 1.47 (m, 3H), 1.19 – 1.01 (m, 2H), 0.97 – 0.85 (m, 7H), 0.78 (d, *J* = 7 Hz, 3H).
^{13}C NMR (101 MHz, $CDCl_3$) δ 166.2 (***xx+yy***), 144.4, 144.4, 129.7 (***xx+yy***), 129.3, 129.3, 128.9 (***xx+yy***), 79.7, 79.6, 74.7 (***xx+yy***), 68.0 (***xx+yy***), 47.4 (***xx+yy***), 42.0, 42.0, 41.1 (***xx+yy***), 34.4 (***xx+yy***), 31.5 (***xx+yy***), 31.1, 31.1, 26.5, 26.5, 25.7 (***xx+yy***), 23.7, 23.7, 22.1 (***xx+yy***), 20.9 (***xx+yy***), 16.6, 16.6.
HRMS (FD+) m/z $[M+H]^+$ calcd. for $C_{22}H_{32}O_3$ 345.2430, found 345.2494.

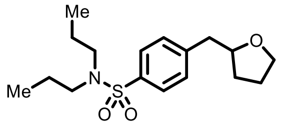

***N,N-dipropyl-4-((tetrahydrofuran-2-yl)methyl)benzenesulfonamide* (32).** Prepared according to GP2 from **1ag** (197 mg, 0.4 mmol, 1.0 equiv.). Purified via flash column chromatography on silica gel (Pentane:Ethyl Acetate 5:1) to afford the product as a colorless oil (84 mg, 65% yield).

^{1}H NMR 1H NMR (400 MHz, CDCl3) δ 7.74 – 7.69 (m, 2H), 7.37 – 7.33 (m, 2H), 4.14 – 4.03 (m, 1H), 3.88 (dt, *J* = 8, 7 Hz, 1H), 3.79 – 3.66 (m, 1H), 3.09 – 2.99 (m, 4H), 2.92 (dd, *J* = 14, 7 Hz, 1H), 2.83 (dd, *J* = 14, 6 Hz, 1H), 2.01 – 1.80 (m, 3H), 1.63 – 1.48 (m, 5H), 0.86 (t, *J* = 7 Hz, 6H).
^{13}C NMR (101 MHz, CDCl3) δ 143.9, 137.9, 129.8, 127.1, 79.3, 68.1, 50.2, 41.7, 31.1, 25.6, 22.1, 11.2.
HRMS (FD+) m/z $[M]^+$ calcd. for $C_{17}H_{27}N_1O_3S_1$ 325.1712, found 325.1702.

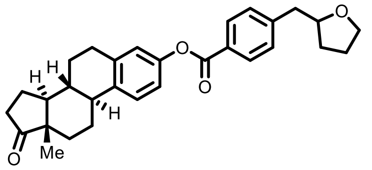

***(8R,9S,13S,14S)-13-methyl-17-oxo-7,8,9,11,12,13,14,15,16,17-decahydro-6H-cyclopenta[a]phenanthren-3-yl 4-((tetrahydrofuran-2-yl)methyl)benzoate* (33).** Prepared according to GP2 from **1ah** (250 mg, 0.4 mmol, 1.0 equiv.). Purified via flash column chromatography on silica gel (From Dichloromethane to Dichloromethane:Ethyl Acetate 60:1) to afford the two diastereomers as an inseparable mixture as white solid. (69 mg, 38% yield). *d.r.* ratio of

product was determined via chiral HPLC to be 1:1 (Analytical condition: Daicel Chiralpak OD-H column, 250 x 4.6 mm I.D. 5 µm; eluent: Heptane/1-Propanol 95/5; flow rate: 1 mL/min; UV @ 254 nm).

^{1}H NMR (400 MHz, $CDCl_3$) δ 8.11 (d, *J* = 8 Hz, 2H), 7.36 (d, *J* = 8 Hz, 2H), 7.32 (d, *J* = 8 Hz, 1H), 7.01 – 6.89 (m, 2H), 4.11 (p, *J* = 7 Hz, 1H), 3.90 (dt, *J* = 8, 7 Hz, 1H), 3.80 – 3.70 (m, 1H), 3.02 – 2.81 (m, 4H), 2.57 – 2.47 (m, 1H), 2.47 – 2.38 (m, 1H), 2.31 (td, *J* = 11, 4 Hz, 1H), 2.22 – 1.80 (m, 6H), 1.71 – 1.37 (m, 8H), 0.92 (s, 3H).
^{13}C NMR (101 MHz, $CDCl_3$) δ 221.1, 165.6, 149.0, 145.4, 138.1, 137.4, 130.3, 129.6, 127.7, 127.1, 126.5, 121.8, 119.0, 79.6, 68.1, 50.5, 48.1, 44.3, 42.0, 38.1, 36.0, 31.6, 31.1, 29.5, 26.4, 25.9, 25.7, 21.7, 13.9.
HRMS (FD+) m/z $[M]^+$ calcd. for $C_{30}H_{34}O_4$ 458.2457, found 458.2454.

Peak 1: Rt= 24,83 ; Area= 50,22%
Peak 2: Rt= 26,77 ; Area= 49,78%

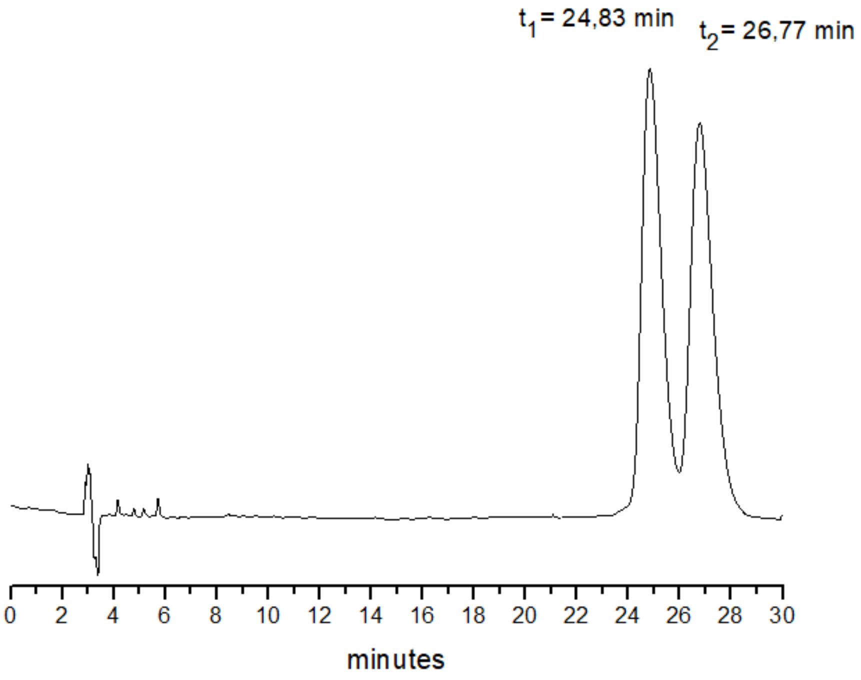

Figure S9: HPLC separation of the two diastereomers of **33**

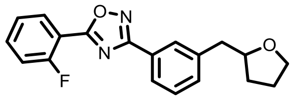

***5-(2-fluorophenyl)-3-(3-((tetrahydrofuran-2-yl)methyl)phenyl)-1,2,4-oxadiazole* (34).** Prepared according to GP2 from **1ai** (196 mg, 0.4 mmol, 1.0 equiv.) using 4 mL of tetrahydrofuran. Purified via flash column chromatography on silica gel (Pentane:Ethyl Acetate 10:1) to afford the product as white solid (50 mg, 77% yield).

^{1}H NMR (400 MHz, CDCl3) δ 8.14 (td, *J* = 8, 2 Hz, 1H), 8.00 – 7.92 (m, 2H), 7.51 (dddd, *J* = 9, 7, 5, 2 Hz, 1H), 7.41 – 7.30 (m, 2H), 7.30 – 7.15 (m, 2H), 4.12 – 4.00 (m, 1H), 3.83 (dt, *J* = 8, 7 Hz, 1H), 3.72 – 3.62 (m, 1H), 2.91 (dd, *J* = 14, 7 Hz, 1H), 2.78 (dd, *J* = 14, 7 Hz, 1H), 1.94 – 1.66 (m, 3H), 1.58 – 1.45 (m, 1H).
^{13}C NMR (101 MHz, CDCl3) δ 172.8 (d, *J* = 4 Hz), 168.9, 160.9 (d, *J* = 261 Hz), 140.0, 134.7 (d, *J* = 9 Hz), 132.4, 131.1 (d, *J* = 1 Hz), 129.0, 128.4, 126.8, 125.6, 124.8 (d, *J* = 4 Hz), 117.2 (d, *J* = 21 Hz), 113.0 (d, *J* = 11 Hz), 79.9, 68.1, 41.9, 31.1, 25.7.
^{19}F NMR (282 MHz, CDCl3) δ -108.3.
HRMS (FD+) m/z [M]$^+$ calcd. for $C_{19}H_{17}F_1N_2O_2$ 324.1274, found 324.1280.

12.4 Characterization of compounds 35 – 61

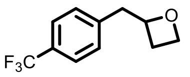

***2-(4-(trifluoromethyl)benzyl)oxetane* (35).** Prepared according to GP3 using oxetane (465 mg, 520 µL 8.0 mmol, 20 equiv.). Purified via flash column chromatography on silica gel (from Hexane:Ethyl Acetate 30:1 to 15:1) to afford the product as a colorless oil (45 mg, 52% yield).

^{1}H NMR (400 MHz, $CDCl_3$) δ 7.55 (d, *J* = 8 Hz, 2H), 7.34 (d, *J* = 8 Hz, 2H), 5.05 (m, 1H), 4.65 (td, *J* = 9 Hz, 1H), 4.46 (dt, *J* = 10 Hz, 1H), 3.13 (dd, *J* = 15 Hz, 1H), 3.02 (dd, *J* = 15 Hz, 1H), 2.73 – 2.59 (m, 1H), 2.41 (ddt, *J* = 12, 7 Hz, 1H).
^{13}C NMR (101 MHz, $CDCl_3$) δ 141.5, 129.7, 128.9 (q, *J* = 32 Hz), 125.4 (q, *J* = 4 Hz), 124.4 (q, *J* = 272 Hz), 82.2, 68.1, 43.8, 27.1.
^{19}F NMR (282 MHz, $CDCl_3$) δ -62.43.
HRMS (FI+) (m/z): [M]$^+$ calcd. for $C_{11}H_{11}F_3O$, 216.0758; found: 216.0762.

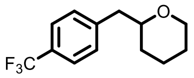

***2-(4-(trifluoromethyl)benzyl)tetrahydro-2H-pyran* (36).** Prepared according to GP3 using tetrahydropyran (689 mg, 783 µL, 8.0 mmol, 20 equiv.). Purified via flash column chromatography on silica gel (Hexane:Ethyl Acetate 40:1) to afford the product as a colorless oil (40 mg, 41% yield).

Characterization data are in accordance with literature.[30]
^{1}H NMR (400 MHz, $CDCl_3$) δ 7.53 (d, *J* = 8 Hz, 2H), 7.33 (d, *J* = 8 Hz, 2H), 3.99 – 3.94 (m, 1H), 3.50 (dddd, *J* = 11, 7, 6, 2 Hz, 1H), 3.39 (td, *J* = 12, 3 Hz, 1H), 2.89 (dd, *J* = 14, 7 Hz, 1H), 2.72 (dd, *J* = 14, 6 Hz, 1H), 1.83 (dq, *J* = 9, 3 Hz, 1H), 1.64 – 1.39 (m, 4H), 1.31 (m, 1H).
^{13}C NMR (101 MHz, $CDCl_3$) δ 143.2, 129.8, 128.6 (q, *J* = 32 Hz), 125.2 (q, *J* = 4 Hz), 124.5 (q, *J* = 272 Hz), 78.4, 68.8, 43.0, 31.7, 26.1, 23.6.
^{19}F NMR (282 MHz, $CDCl_3$) δ -62.34.

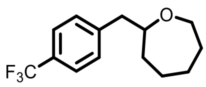

***2-(4-(trifluoromethyl)benzyl)oxepane* (37).** Prepared according to GP3 using 1,3-dioxolane (593 mg, 544 µL 8.0 mmol, 20 equiv.). Purified via flash column chromatography on silica gel (Hexane:Ethyl Acetate 90:1) to afford the product as a colorless oil (53 mg, 51% yield).

^{1}H NMR (400 MHz, $CDCl_3$) δ 7.53 (d, *J* = 8 Hz, 2H), 7.32 (d, *J* = 8 Hz, 2H), 3.85 – 3.76 (m, 1H), 3.70 (tt, *J* = 9 Hz, 1H), 3.44 (ddd, *J* = 13, 4 Hz, 1H), 2.86 (dd, *J* = 15 Hz, 1H), 2.71 (dd, *J* = 15 Hz, 1H), 1.87 – 1.69 (m, 3H), 1.69 – 1.46 (m, 5H).
^{13}C NMR (101 MHz, $CDCl_3$) δ 143.9, 129.7, 128.4 (q, *J* = 32 Hz), 125.2 (q, *J* = 4 Hz), 123.3 (q, *J* = 273 Hz), 80.3, 68.6, 43.1, 35.8, 31.1, 26.7, 25.9.
^{19}F NMR (282 MHz, $CDCl_3$) δ -62.45.
HRMS (CI+) (m/z): $[M+H]^+$ calcd. for $C_{14}H_{17}F_3O$, 259.1310; found: 259.1315

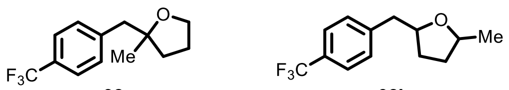

38 **38'**

***2-methyl-2-(4-(trifluoromethyl)benzyl)tetrahydrofuran* (38)*;* *2-methyl-5-(4-(trifluoromethyl)benzyl)tetrahydrofuran* (38').** Prepared according to GP3 using methyl tetrahydrofuran (689 mg, 807 µL, 8.0 mmol, 20 equiv.). *r:r* ratio was determined via ^{1}H NMR of the reaction crude to be ca. 1.5 (**38**):1 (**38'**). Purified via flash column chromatography on silica gel (from Hexane:Ethyl Acetate 98:2 to 96:4) to afford the two regioisomers as an inseparable mixture as a colorless oil (57 mg, 58% yield). *d.r.* ratio of regioisomer **38'** was determined via ^{1}H NMR after flash column chromatography to be ca. 1.8:1.

^{1}H NMR (400 MHz, $CDCl_3$) δ 7.56 – 7.49 (m, 4H, **38**+**38'**), 7.38 – 7.30 (m, 4H, **38**+**38'**), 4.29 – 3.92 (m, 2H, **38'**), 3.90 – 3.81 (m, 1H, **38**), 3.81 – 3.71 (m, 1H, **38**), 2.99 – 2.90 (m, 1H, **38'**), 2.84 (s, 2H, **38**), 2.82 – 2.73 (m, 1H, **38'**), 2.08 – 1.52 (m, 8H, **38**+**38'**), 1.25 – 1.19 (m, 3H, **38'**), 1.17 (s, 3H, **38**).
^{13}C NMR (101 MHz, $CDCl_3$, **38**) δ 142.8, 130.9, 128.6 (q, *J* = 32 Hz), 124.9 (q, *J* = 4 Hz), 124.6 (q, *J* = 272 Hz), 82.6, 67.7, 46.8, 36.7, 26.5, 26.1.
^{13}C NMR (101 MHz, $CDCl_3$, **38'**) δ 143.3 (*major*+*minor*), 129.8 (*minor*), 129.8 (*major*), 128.6 (q, *J* = 32 Hz, *major*+*minor*) 125.42 – 125.14 (m, *major*+*minor*), 124.5 (q, *J* = 272 Hz, *major*+*minor*), 79.6 (*minor*), 79.0 (*major*), 75.8 (*minor*), 75.1 (*major*), 42.4 (*minor*), 42.2 (*major*), 33.9 (*major*), 32.8 (*minor*), 32.0 (*major*), 31.0 (*minor*), 21.6 (*minor*), 21.5 (*major*).
^{19}F NMR (282 MHz, $CDCl_3$) δ -62.33, -62.36.

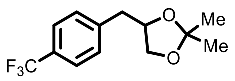

***2,2-dimethyl-4-(4-(trifluoromethyl)benzyl)-1,3-dioxolane* (39).** Prepared according to GP3 using 2,2-dimethyl-1,3-dioxolane (2 mL). Purified via flash column chromatography on silica gel (from Hexane:Ethyl Acetate 40:1 to 20:1) to afford the product as a colorless oil (52 mg, 50% yield).

^{1}H NMR (400 MHz, $CDCl_3$) δ 7.56 (d, *J* = 8 Hz, 2H), 7.34 (d, *J* = 8 Hz, 2H), 4.33 (p, *J* = 6 Hz, 1H), 4.01 (dd, *J* = 8, 6 Hz, 1H), 3.64 (dd, *J* = 8, 7 Hz, 1H), 3.01 (dd, *J* = 14, 7 Hz, 1H), 2.86 (dd, *J* = 14, 6 Hz, 1H), 1.43 (s, 3H), 1.35 (s, 3H).

^{13}C NMR (101 MHz, CDCl$_3$) δ 141.9, 129.7, 129.0 (q, *J* = 32 Hz), 125.5 (q, *J* = 4 Hz), 124.4 (q, *J* = 271 Hz), 109.5, 76.3, 69.0, 40.1, 27.1, 25.8.
^{19}F NMR (282 MHz, CDCl$_3$) δ -62.47.
HRMS (CI+) (m/z): $[M+H]^+$ calcd. for $C_{13}H_{15}F_3O2$, 261.1102; found: 261.1107.

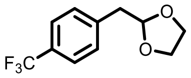

***2-(4-(trifluoromethyl)benzyl)-1,3-dioxolane* (40).** Prepared according to GP3 using 1,3-dioxolane (593 mg, 544 µL 8.0 mmol, 20 equiv.). Purified via flash column chromatography on silica gel (Hexane:Ethyl Acetate 40:1) to afford the product as a white solid (51 mg, 55% yield).

^{1}H NMR (400 MHz, CDCl$_3$) δ 7.56 (d, *J* = 8 Hz, 2H), 7.39 (d, *J* = 8 Hz, 2H), 5.08 (t, *J* = 5 Hz, 1H), 3.96 – 3.80 (m, 4H), 3.02 (d, *J* = 5 Hz, 2H).
^{13}C NMR (101 MHz, CDCl$_3$) δ 140.3, 130.3, 129.1 (q, *J* = 32 Hz), 125.3 (q, *J* = 4 Hz), 124.4 (q, *J* = 273 Hz), 104.2, 65.2, 40.7.
^{19}F NMR (282 MHz, CDCl$_3$) δ -62.45.
HRMS (CI+) (m/z): $[M+H]^+$ calcd. for $C_{11}H_{11}F_3O_2$, 233.0793; found: 233.0789.

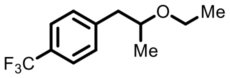

***1-(2-ethoxypropyl)-4-(trifluoromethyl)benzene* (41).** Prepared according to GP3 using diethyl ether (593 mg, 840 µL, 8.0 mmol, 20 equiv.). Purified via flash column chromatography on silica gel (from Hexane to Hexane:Ethyl Acetate 98:2) to afford the product as a colorless oil (46 mg, 50% yield).

^{1}H NMR (400 MHz, CDCl$_3$) δ 7.57 – 7.50 (m, 2H), 7.36 – 7.29 (m, 2H), 3.62 (d, *J* = 6 Hz, 1H), 3.54 (dq, *J* = 9, 7 Hz, 1H), 3.38 (dq, *J* = 9, 7 Hz, 1H), 2.91 (dd, *J* = 14, 7 Hz, 1H), 2.71 (dd, *J* = 14, 6 Hz, 1H), 1.21 – 1.10 (m, 6H).
^{13}C NMR (101 MHz, CDCl$_3$) δ 143.5, 129.9,128.5 (q, *J* = 32 Hz), 125.2 (q, *J* = 4 Hz), 124.5 (q, *J* = 272 Hz), 76.1, 64.2, 43.1, 19.8, 15.6.
^{19}F NMR (282 MHz, CDCl$_3$) δ -62.33.

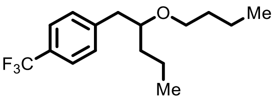

1-(2-butoxypentyl)-4-(trifluoromethyl)benzene (42). Prepared according to GP3 using dibutyl ether (1.0 g, 1.3 mL 8.0 mmol, 20 equiv.). Purified via flash column chromatography on silica gel (Hexane) to afford the product as a colorless oil (60 mg, 52% yield).

^{1}H NMR ^{1}H NMR (400 MHz, CDCl$_3$) δ 7.53 (d, *J* = 8 Hz, 2H), 7.32 (d, *J* = 8 Hz, 2H), 3.50 – 3.28 (m, 3H), 2.86 – 2.74 (m, 2H), 1.52 – 1.23 (m, 8H), 0.94-0.83 (m, 6H).
^{13}C NMR (101 MHz, CDCl$_3$) δ 143.8, 129.9, 128.4 (q, *J* = 32 Hz), 125.14 (q, *J* = 4 Hz), 124.6 (q, *J* = 272 Hz), 80.4, 69.5, 40.8, 36.6, 32.3, 19.5, 18.9, 14.3, 14.0.
^{19}F NMR (282 MHz, CDCl$_3$) δ -62.32.

HRMS (FI+) (m/z): $[M]^+$ calcd. for $C_{16}H_{23}F_3O$, 288.1701; found: 288.1698.

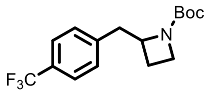

tert-butyl 2-(4-(trifluoromethyl)benzyl)azetidine-1-carboxylate (43). Prepared according to GP3 using tert-butyl azetidine-1-carboxylate (314 mg, 2.0 mmol, 5.0 equiv.). Purified via flash column chromatography on silica gel (Dichloromethane:Hexane 1:1) to afford the product as a colorless oil (71 mg, 56% yield).

^{1}H NMR (400 MHz, $CDCl_3$) δ 7.55 (d, J = 8 Hz, 2H), 7.30 (d, J = 8 Hz, 2H), 4.47 – 4.39 (m, 1H), 3.83 – 3.75 (m, 1H), 3.66 – 3.55 (m, 1H), 3.20 (dd, J = 13, 3 Hz, 1H), 3.01 (dd, J = 14, 8 Hz, 1H), 2.21 – 2.09 (m, 1H), 1.86 (ddt, J = 12, 9, 6 Hz, 1H), 1.44 (s, 9H).
^{13}C NMR (101 MHz, $CDCl_3$) δ 156.4, 141.6, 129.8, 128.8 (q, J = 32 Hz), 125.3 (q, J = 4 Hz), 124.3 (q, J = 272 Hz), 79.5, 61.9, 46.2, 40.7, 28.4, 21.0.
^{19}F NMR (282 MHz, $CDCl_3$) δ -62.50.
HRMS (FD+) (m/z): $[M]^+$ calcd. for $C_{16}H_{20}F_3NO_2$, 315.1446; found: 315.1462

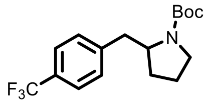

tert-butyl 2-(4-(trifluoromethyl)benzyl)pyrrolidine-1-carboxylate **(44).** Prepared according to GP3 using tert-butyl pyrrolidine-1-carboxylate (342 mg, 2.0 mmol, 5.0 equiv.). Purified via flash column chromatography on silica gel (from Hexane:Dichloromethane 50:50 to 40:60 to 30:70) to afford the product (mixture of rotamers) as a colorless oil (72 mg, 55% yield).

Characterization data are in accordance with literature.[31]
^{1}H NMR (400 MHz, $CDCl_3$) δ 7.53 (d, J = 8 Hz, 2H), 7.34 – 7.27 (m, 2H), 4.09 – 3.90 (m, 1H), 3.44 – 3.25 (s, 2H), 3.20 – 3.03 (m, 1H), 2.63 (dd, J = 13, 9 Hz, 1H), 1.90 – 1.59 (m, 4H), 1.49 (s, 9H).
^{13}C NMR (101 MHz, $CDCl_3$) δ 154.6, 143.5 (q, J = 2 Hz), 129.9, 128.7 (q, J = 33 Hz), 125.4 (q, J = 4 Hz), 124.4 (q, J = 273 Hz), 79.5, 58.6, 46.7, 40.4, 29.6, 28.7, 23.2.
^{19}F NMR (282 MHz, $CDCl_3$) δ -62.36.

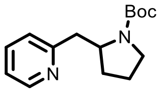

tert-butyl 2-(pyridin-2-ylmethyl)pyrrolidine-1-carboxylate **(45).** Prepared according to GP3 using tert-butyl pyrrolidine-1-carboxylate (342 mg, 2.0 mmol, 5.0 equiv.) and hydrazone **1y** (132 mg, 0.4 mmol, 1.0 equiv.). *N.B. The solvent was removed under reduced pressure. Then the crude was dissolved in diethyl ether (5 mL) and extracted with 0.5 M HCl (5 mL). The aqueous phase was washed with diethyl ether (2 × 5 mL). The collected aqueous phases were basified to pH 9-10 with a saturated solution of potassium carbonate and extracted with diethyl ether (4 x 5 mL). The combined organic phases were dried over* Na_2SO_4 *and concentrated under reduced pressure.* The obtained crude was purified via flash column chromatography on silica gel (from Hexane:Ethyl Acetate 80:20 to 60:40) to afford the product (mixture of rotamers) as a yellowish oil (48 mg, 46% yield).

Characterization data are in accordance with literature.[32]

^{1}H NMR (400 MHz, $CDCl_3$) δ 8.50 (d, *J* = 5 Hz, 1H), 7.58 (td, *J* = 8, 2 Hz, 1H), 7.25 – 7.03 (m, 2H), 4.12 (d, *J* = 9 Hz, 1H), 3.49 – 3.05 (m, 3H), 2.79 (dd, *J* = 13, 9 Hz, 1H), 1.95 – 1.68 (m, 4H), 1.46 (s, 9H).

^{13}C NMR (101 MHz, $CDCl_3$) δ 159.6, 154.6, 149.4 & 149.2 (rotameric signals), 136.4, 124.0 & 123.8 (rotameric signals), 121.4, 79.4 & 79.2 (rotameric signals), 58.1, 46.8 & 46.4 (rotameric signals), 43.0 & 42.1 (rotameric signals), 30.1 & 29.1 (rotameric signals), 28.7, 23.6 & 22.8 (rotameric signals).

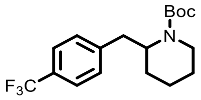

***tert-butyl 2-(4-(trifluoromethyl)benzyl)piperidine-1-carboxylate* (46).** Prepared according to GP3 using tert-butyl piperidine-1-carboxylate (371 mg, 2.0 mmol, 5.0 equiv.). Purified via flash column chromatography on silica gel (from Hexane:Ethyl Acetate 99:1 to 98:2) to afford the product (mixture of rotamers) as a colorless oil (58 mg, 42% yield).

^{1}H NMR (400 MHz, $CDCl_3$) δ 7.53 (d, *J* = 8 Hz, 2H), 7.28 (d, *J* = 15 Hz, 3H), 4.44 (s, 1H), 4.06 (s, 1H), 2.99 (dd, *J* = 13, 8 Hz, 1H), 2.91 (td, *J* = 13, 3 Hz, 1H), 2.78 (dd, *J* = 13, 7 Hz, 1H), 1.75 – 1.61 (m, 3H), 1.61 – 1.50 (m, 2H), 1.29 (s, 9H).

^{13}C NMR (101 MHz, $CDCl_3$) δ 154.9, 143.7, 129.7, 128.7 (q, *J* = 32 Hz), 125.39 (q, *J* = 4 Hz), 124.4 (q, *J* = 272 Hz), 79.4, 52.2, 39.1, 36.1, 28.4, 28.0, 25.6, 19.1.

^{19}F NMR (282 MHz, $CDCl_3$) δ -62.4.

HRMS (FD+) (m/z): $[M]^+$ calcd. for $C_{18}H_{24}F_3NO_2$, 343.1759; found: 343.1768.

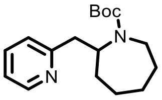

***tert-butyl 2-(pyridin-2-ylmethyl)azepane-1-carboxylate* (47).** Prepared according to GP3 using tert-butyl azepane-1-carboxylate (399 mg, 2.0 mmol, 5.0 equiv.) and hydrazone **1y** (132 mg, 0.4 mmol, 1.0 equiv.). *N.B. The solvent was removed under reduced pressure. Then the crude was dissolved in diethyl ether (5 mL) and extracted with 0.5 M HCl (5 mL). The aqueous phase was washed with diethyl ether (2 × 5 mL). The collected aqueous phases were basified to pH 9-10 with a saturated solution of potassium carbonate and extracted with diethyl ether (4 x 5 mL). The combined organic phases were dried over Na_2SO_4 and concentrated under reduced pressure.*

The obtained crude was via flash column chromatography on silica gel (from Hexane:Ethyl Acetate 10:1 to 5:1) to afford the product (mixture of rotamers) as a yellowish oil (60 mg, 52% yield).

^{1}H NMR (400 MHz, $CDCl_3$) δ 8.55 – 8.44 (m, 1H), 7.62 – 7.51 (m, 1H), 7.24 & 7.04 (rotameric d, *J* = 8 Hz, 1H), 7.12 (dd, *J* = 8, 4 Hz, 1H), 4.37 & 4.27 (rotameric dt, *J* = 13, 7 Hz, 1H), 3.77 & 3.60 (rotameric d, *J* = 15 Hz, 1H), 3.02 – 2.81 (rotameric m, 2H), 2.72 (tdd, *J* = 14, 5, 1.4 Hz, 1H), 2.10 – 1.86 (m, 1H), 1.80 – 1.50 (m, 4H), 1.39 – 1.37 (m, 4H), 1.33 – 1.12 (m, 8H).

^{13}C NMR (101 MHz, $CDCl_3$) δ 159.5 & 159.2 (rotameric signals), 155.7 & 155.5 (rotameric signals), 149.1 & 148.4 (rotameric signals), 136.8 & 136.5 (rotameric signals), 124.1 & 124.0 (rotameric signals), 121.4 &121.4 (rotameric signals), 79.0 & 79.0 (rotameric signals), 56.6 & 55.9 (rotameric signals), 43.5 & 43.07 (rotameric signals), 42.3 & 41.6 (rotameric signals), 34.8 & 33.7 (rotameric signals), 29.9 & 29.7 (rotameric signals), 29.4 & 28.7 (rotameric signals), 28.6 & 28.4 (rotameric signals), 25.3 & 25.2 (rotameric signals).

HRMS (FD+) (m/z): $[M]^+$ calcd. for $C_{17}H_{26}N_2O_2$, 290.1994; found: 290.1984.

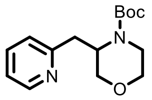

tert-butyl 3-(pyridin-2-ylmethyl)morpholine-4-carboxylate **(48).** Prepared according to GP3 using tert-butyl morpholine-1-carboxylate (749 mg, 4.0 mmol, 10 equiv.). *N.B. The solvent was removed under reduced pressure. Then the crude was dissolved in diethyl ether (5 mL) and extracted with 0.5 M HCl (5 mL). The aqueous phase was washed with diethyl ether (2 × 5 mL). The collected aqueous phases were basified to pH 9-10 with a saturated solution of potassium carbonate and extracted with diethyl ether (4 x 5 mL). The combined organic phases were dried over* Na_2SO_4 *and concentrated under reduced pressure.* Later, the obtained crude was purified via flash column chromatography on silica gel (from Hexane:Ethyl Acetate 70:30 to 50:50) to afford the product (mixture of rotamers) as a colorless oil (38 mg, 34% yield).

^{1}H NMR (400 MHz, $CDCl_3$) δ 8.56 – 8.50 (m, 1H), 7.57 (td, *J* = 8, 2 Hz, 1H), 7.24 – 7.04 (m, 2H), 4.40 – 4.30 (m, 1H), 4.00 – 3.67 (m, 3H), 3.59 – 3.42 (m, 2H), 3.40 – 3.27 (m, 1H), 3.28 – 2.98 (m, 2H), 1.39 – 1.18 (m, 9H).
^{13}C NMR (101 MHz, $CDCl_3$) δ 158.9, 154.4, 149.4, 136.4, 123.8, 121.4, 79.7, 69.1, 67.0, 52.4, 38.7, 37.5, 28.2.
HRMS (FD+) (m/z): $[M]^+$ calcd. for $C_{15}H_{22}N_2O_3$, 278.1630; found: 278.1639.

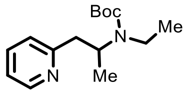

tert-butyl ethyl(1-(pyridin-2-yl)propan-2-yl)carbamate **(49).** Prepared according to GP3 using tert-butyl diethylcarbamate (1.3 g, 8.0 mmol, 20.0 equiv.) and **1y** (132 mg, 0.4 mmol, 1.0 equiv.). Purified via flash column chromatography on silica gel (from Hexane:Ethyl Acetate 10:1 to 5:1) to afford the product (mixture of rotamers) as a colorless oil (53 mg, 50% yield).

^{1}H NMR (400 MHz, $CDCl_3$) δ 8.54 – 8.46 (m, 1H), 7.55 (td, *J* = 8, 2 Hz, 1H), 7.22 – 7.02 (m, 2H), 4.47 – 4.08 (m, 1H), 3.19 – 2.99 (m, 3H), 2.87 (dd, *J* = 13, 6 Hz, 1H), 1.38 (s, 9H), 1.27 – 1.20 (m, 3H), 1.01 – 0.93 (m, 3H).
^{13}C NMR (101 MHz, $CDCl_3$) δ 159.7, 155.3, 149.3, 136.3, 123.7, 121.4, 79.0, 53.8 & 53.0 (rotameric signals), 43.5 & 43.5 (rotameric signals), 39.6 & 39.4 (rotameric signals), 28.6, 19.9 & 18.9 (rotameric signals), 15.3 & 14.7 (rotameric signals).
HRMS (ESI+) (m/z): $[M+H]^+$ calcd. for $C_{15}H_{24}N_2O_2$, 265.1916; found: 165.1914.

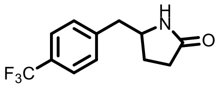

5-(4-(trifluoromethyl)benzyl)pyrrolidin-2-one **(50).** Prepared according to GP3 using pyrrolidin-2-one (170 mg, 2.0 mmol, 5.0 equiv.). Purified via flash column chromatography on silica gel (from Hexane:Ethyl Acetate 2:1 to 1:1) to afford the product as a white solid (54 mg, 56% yield).

^{1}H NMR (400 MHz, $CDCl_3$) δ 7.57 (d, *J* = 8 Hz, 2H), 7.30 (d, *J* = 8 Hz, 2H), 6.24 (s, 1H), 3.96 – 3.87 (m, 1H), 2.88 (dd, *J* = 13, 6 Hz, 1H), 2.82 (dd, *J* = 13, 7 Hz, 1H), 2.36 – 2.16 (m, 3H), 1.88 – 1.79 (m, 1H).

^{13}C NMR (101 MHz, $CDCl_3$) δ 178.1, 141.6, 129.9, 129.1 (q, J = 33 Hz), 125.8 (q, J = 4 Hz), 124.2 (q, J = 272 Hz), 55.4, 42.8, 30.1, 26.9.
^{19}F NMR (282 MHz, $CDCl_3$) δ -62.50.
HRMS (FD+) (m/z): $[M+H]^+$ calcd. for $C_{12}H_{12}F_3NO$, 244.0966; found: 244.0949.

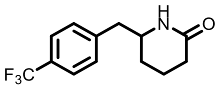

***6-(4-(trifluoromethyl)benzyl)piperidin-2-one* (51).** Prepared according to GP3 using 2-piperidone (793 mg, 8.0 mmol, 20 equiv.). Purified via flash column chromatography on silica gel (Dichloromethane:Methanol 99:1) to afford the product as a white solid (46 mg, 45% yield).

^{1}H NMR (400 MHz, $CDCl_3$) δ 7.58 (d, J = 8 Hz, 2H), 7.30 (d, J = 8 Hz, 2H), 5.94 (s, 1H), 3.69 – 3.58 (m, 1H), 2.89 (dd, J = 13, 6 Hz, 1H), 2.76 (dd, J = 13, 8 Hz, 1H), 2.48 – 2.22 (m, 2H), 1.92 (qdd, J = 10, 5, 3 Hz, 2H), 1.78 – 1.57 (m, 1H), 1.52 – 1.37 (m, 1H).
^{13}C NMR (101 MHz, $CDCl_3$) δ 172.4, 141.1 (q, J = 2 Hz), 129.7, 129.6 (q, J = 273 Hz), 125.9 (q, J = 4 Hz), 124.2 (q, J = 272 Hz), 54.2, 43.3, 31.5, 28.6, 19.7.
^{19}F NMR (282 MHz, $CDCl_3$) δ -62.54.
HRMS (FD+) (m/z): $[M]^+$ calcd. for $C_{13}H_{14}F_3NO$, 258.1106; found: 258.1104.

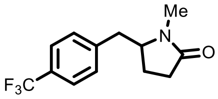

***1-methyl-5-(4-(trifluoromethyl)benzyl)pyrrolidin-2-one* (52).** Prepared according to GP3 from N-methyl-2-pyrrolidone (198 mg, 192 µL, 2.0 mmol, 5.0 equiv.). Purified via flash column chromatography on silica gel (from Dichloromethane to Dichloromethane:Methanol 99:1) to afford the product as a yellowish oil (62 mg, 60% yield).

^{1}H NMR (400 MHz, $CDCl_3$) δ 7.57 (d, J = 8 Hz, 2H), 7.28 (d, J = 8 Hz, 2H), 3.77 (tt, J = 8, 4 Hz, 1H), 3.10 (dd, J = 13, 4 Hz, 1H), 2.88 (s, 3H), 2.70 (dd, J = 13, 8 Hz, 1H), 2.31 – 2.11 (m, 2H), 2.07 – 1.90 (m, 1H), 1.70 (dddd, J = 13, 9, 6, 4 Hz, 1H).
^{13}C NMR (101 MHz, $CDCl_3$) δ 175.2, 141.2, 129.7, 129.4 (q, J = 32 Hz), 125.7 (q, J = 4 Hz), 124.2 (q, J = 273 Hz), 60.9, 39.4, 29.7, 28.4, 23.6.
^{19}F NMR (282 MHz, $CDCl_3$) δ -62.48.
HRMS (ESI+) (m/z): $[M+H]^+$ calcd. for $C_{13}H_{14}F_3NO$, 258.1106; found: 258.1101

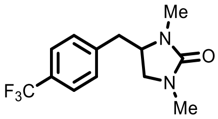

***1,3-dimethyl-4-(4-(trifluoromethyl)benzyl)imidazolidin-2-one* (53).** Prepared according to GP3 using 1,3-dimethylimidazolidin-2-one (228 mg, 216µL, 2.0 mmol, 5.0 equiv.). Purified via flash column chromatography on silica gel (Dichloromethane:Ethyl Acetate 6:1) to afford the product as a yellowish oil (56 mg, 51% yield).

^{1}H NMR (400 MHz, $CDCl_3$) δ 7.58 (d, *J* = 8 Hz, 2H), 7.30 (d, *J* = 8 Hz, 2H), 3.69 – 3.60 (m, 1H), 3.24 – 3.13 (m, 2H), 2.93 – 2.86 (m, 1H), 2.81 (s, 3H), 2.79 – 2.66 (m, 4H).
^{13}C NMR (101 MHz, $CDCl_3$) δ 161.6, 141.1, 129.6, 129.4 (q, *J* = 32 Hz), 125.8 (q, *J* = 4 Hz), 124.2 (q, *J* = 272 Hz), 56.9, 50.5, 38.7, 31.3, 30.0.
^{19}F NMR (282 MHz, $CDCl_3$) δ -62.51.
HRMS (FD+) (m/z): $[M]^+$ calcd. for $C_{13}H_{15}F_3N_2O$, 272.1136; found: 272.1144.

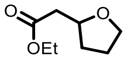

***ethyl 2-(tetrahydrofuran-2-yl)acetate* (54).** Prepared according to GP4 using tetrahydrofuran (144 mg, 2.0 mmol, 162 µL, 5.0 equiv.). Purified via flash column chromatography on silica gel (Pentane:Ethyl Acetate 60:1) to afford the product as a colorless oil. *N.B. ^{1}H NMR yield reported due to volatility of the product.*

Characterization data are in accordance with literature.[33]
^{1}H NMR (400 MHz, $CDCl_3$) δ 4.24 (p, *J* = 7 Hz, 1H), 4.14 (q, *J* = 7 Hz, 2H), 3.87 (q, *J* = 7 Hz, 1H), 3.74 (q, *J* = 8 Hz, 1H), 2.58 (dd, *J* = 15, 7 Hz, 1H), 2.45 (dd, *J* = 15, 6 Hz, 1H), 2.08 (td, *J* = 12, 6 Hz, 1H), 1.91 (ddd, *J* = 14, 8, 5 Hz, 2H), 1.54 (dq, *J* = 12, 8 Hz, 1H), 1.25 (t, *J* = 7 Hz, 3H).
^{13}C NMR (101 MHz, $CDCl_3$) δ 171.5, 75.4, 68.1, 60.6, 40.8, 31.4, 25.7, 14.3.

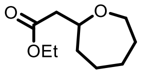

***ethyl 2-(oxepan-2-yl)acetate* (55).** Prepared according to GP4 from oxepane (200 mg, 0.223 mL, 2.0 mmol, 5.0 equiv.). Purified via flash column chromatography on silica gel (from Pentane:Toluene 70:30 to Pentane:Diethyl Ether 90:10) to afford the product as a colorless oil (46 mg, 61% yield).

Characterization data are in accordance with literature.[34]
^{1}H NMR (400 MHz, $CDCl_3$) δ 4.13 (q, *J* = 7 Hz, 2H), 3.95 (tdd, *J* = 9, 5, 3 Hz, 1H), 3.80 (ddd, *J* = 12, 6, 5 Hz, 1H), 3.56 (ddd, *J* = 12, 7, 4 Hz, 1H), 2.50 (dd, *J* = 15, 9 Hz, 1H), 2.35 (dd, *J* = 15, 5 Hz, 1H), 1.85 – 1.39 (m, 7H), 1.24 (t, *J* = 7 Hz, 3H).
^{13}C NMR (101 MHz, $CDCl_3$) δ 171.7, 76.2, 69.0, 60.4, 42.2, 35.7, 31.0, 26.4, 26.1, 14.3.

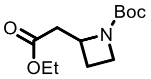

***tert-butyl 2-(2-ethoxy-2-oxoethyl)azetidine-1-carboxylate* (56).** Prepared according to GP4 using tert-butyl azetidine-1-carboxylate (314 mg, 2.0 mmol, 5.0 equiv.). Purified via flash column chromatography on silica gel (from Pentane:Ethyl Acetate 30:1 to 15:1) to afford the product as a colorless oil (41 mg, 42% yield).

^{1}H NMR (400 MHz, $CDCl_3$) δ 4.54 – 4.44 (m, 1H), 4.13 (q, *J* = 7 Hz, 2H), 3.89 – 3.76 (m, 2H), 3.00 – 2.91 (m, 1H), 2.63 (dd, *J* = 16, 9 Hz, 1H), 2.37 (dtd, *J* = 12, 9, 5 Hz, 1H), 2.04 – 1.92 (m, 1H), 1.42 (s, 9H), 1.25 (t, *J* = 7 Hz, 3H).
^{13}C NMR (101 MHz, $CDCl_3$) δ 170.9, 156.3, 79.7, 60.6, 58.1, 46.6, 40.1, 28.5, 22.4, 14.3.
HRMS (ESI+) (m/z): $[M+Na]^+$ calcd. for $C_{12}H_{21}NO_4$, 266.1368; found: 266.1360.

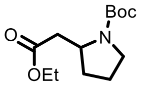

tert-butyl 2-(2-ethoxy-2-oxoethyl)pyrrolidine-1-carboxylate **(57).** Prepared according to GP4 from tert-butyl pyrrolidine-1-carboxylate (342 mg, 2.0 mmol, 5.0 equiv.). Purified via flash column chromatography on silica gel (Petroleum Ether:Ethyl Acetate 30:1) to afford the product as a colorless oil (58 mg, 56% yield).

Characterization data are in accordance with literature.[35]

^{1}H NMR (400 MHz, $CDCl_3$) δ 4.19 – 4.00 (m, 3H), 3.39 – 3.23 (m, 2H), 2.93 & 2.77 (rotameric d, J = 14 Hz, 1H), 2.27 (dd, J = 15, 10 Hz, 1H), 2.02 (dt, J = 12, 8 Hz, 1H), 1.88 – 1.68 (m, 3H), 1.44 (s, 9H), 1.23 (t, J = 7 Hz, 3H).

^{13}C NMR (101 MHz, $CDCl_3$) δ 171.7, 154.4, 79.6 & 79.3 (rotameric signals), 60.4, 54.2, 46.6 & 46.3 (rotameric signals), 39.5 & 38.7 (rotameric signals), 31.3 & 30.6 (rotameric signals), 28.6, 23.6 & 22.9 (rotameric signals), 14.3.

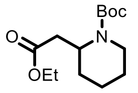

tert-butyl 2-(2-ethoxy-2-oxoethyl)piperidine-1-carboxylate **(58).** Prepared according to GP4 from tert-butyl piperidine-1-carboxylate (371 mg, 2.0 mmol, 5.0 equiv.). Purified via flash column chromatography on silica gel (from Pentane:Ethyl Acetate 98:2 to 95:5) to afford the product as a colorless oil (77 mg, 71% yield).
When the same reaction was performed with 2.5 equivalent of tert-butyl piperidine-1-carboxylate, product **58** was formed in 52% yield (^{1}H-NMR, trichloroethylene as the external standard).

^{1}H NMR (400 MHz, $CDCl_3$) δ 4.66 (q, J = 7 Hz, 1H), 4.08 (q, J = 7 Hz, 2H), 3.95 (d, J = 14 Hz, 1H), 2.75 (t, J = 13 Hz, 1H), 2.59 – 2.44 (m, 2H), 1.67 – 1.29 (m, 15H), 1.22 (t, J = 7 Hz, 3H).

^{13}C NMR (101 MHz, $CDCl_3$) δ 171.5, 154.8, 79.6, 60.5, 48.0, 39.3, 35.4, 28.5, 28.3, 25.4, 18.9, 14.2.

HRMS (ESI+) (m/z): $[M+Na]^+$ for $C_{14}H_{25}NO_4$, 294.1681; found:294.1681.

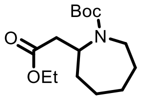

tert-butyl 2-(2-ethoxy-2-oxoethyl)azepane-1-carboxylate **(59).** Prepared according to GP4 using using tert-butyl azepane-1-carboxylate (399 mg, 2.0 mmol, 5.0 equiv.). Purified via flash column chromatography on silica gel (Pentane:Ethyl Acetate 20:1) to afford the product (mixture of rotamers) as a colorless oil (87 mg, 76% yield).

Characterization data are in accordance with literature.[35]

^{1}H NMR (400 MHz, $CDCl_3$) δ 4.43 – 4.33 & 4.27 – 4.17 (rotameric m, 1H), 4.08 (p, J = 7 Hz, 3H), 3.71 & 3.60 (rotameric d, J = 15 Hz, 1H), 2.80 – 2.67 (m, 1H), 2.47 (dt, J = 13, 6 Hz, 1H), 2.30 (dt, J = 14, 7 Hz, 1H), 2.12 – 1.94 (m, 1H), 1.79 – 1.67 (m, 3H), 1.55 – 1.38 (m, 10H), 1.36 – 1.19 (m, 6H).

^{13}C NMR (101 MHz, $CDCl_3$) δ 171.5 & 171.5 (rotameric signals), 155.6 & 155.4 (rotameric signals), 79.6 & 79.3 (rotameric signals), 60.5, 53.6 & 52.6 (rotameric signals), 42.4 & 42.2 (rotameric signals), 40.3 & 39.8 (rotameric signals), 33.9 & 33.6 (rotameric signals), 29.5 & 29.4 (rotameric signals), 29.5 & 29.1 (rotameric signals), 28.5, 25.5 & 25.2 (rotameric signals), 14.2.

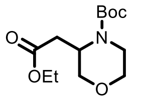

***tert-butyl 3-(2-ethoxy-2-oxoethyl)morpholine-4-carboxylate* (60).** Prepared according to GP4 from tert-butyl morpholine-4-carboxylate (749 mg, 4.0 mmol, 10 equiv.). Purified via flash column chromatography on silica gel (from Pentane:Diethyl Ether 98:2 to 90:10 to 80:20) to afford the product as a colorless oil (38 mg, 35% yield).

^{1}H NMR (400 MHz, $CDCl_3$) δ 4.45 – 4.28 (m, 1H), 4.12 (q, J = 7 Hz, 2H), 3.96 – 3.67 (m, 3H), 3.57 (dd, J = 12, 3 Hz, 1H), 3.52 – 3.35 (m, 1H), 3.21 – 3.00 (m, 1H), 2.82 (dd, J = 15, 9 Hz, 1H), 2.55 (dd, J = 15, 6 Hz, 1H), 1.48 – 1.43 (m, 9H), 1.25 (t, J = 7 Hz, 3H).
^{13}C NMR (101 MHz, $CDCl_3$) δ 171.3, 154.5, 80.4, 69.0, 67.0, 60.7, 47.7, 39.3, 33.9, 28.5, 14.3.
HRMS (ESI+) (m/z): $[M+Na]^+$ calcd. for $C_{13}H_{23}NO_5$, 296.1474; found: 296.1476.

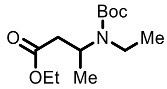

***ethyl 3-((tert-butoxycarbonyl)(ethyl)amino)butanoate* (61).** Prepared according to GP4 using tert-butyl diethylcarbamate (650 mg, 4.0 mmol, 10 equiv.). Purified via flash column chromatography on silica gel (Pentane:Ethyl Acetate 30:1) to afford the product as a colorless oil (68 mg, 66% yield).

^{1}H NMR (400 MHz, $CDCl_3$) δ 4.49 – 3.97 (m, 3H), 3.31 – 3.06 (m, 2H), 2.64 (dd, J = 15, 7 Hz, 1H), 2.43 (dd, J = 15, 7 Hz, 1H), 1.45 (s, 9H), 1.24 (t, J = 7 Hz, 6H), 1.10 (t, J = 7 Hz, 3H).
^{13}C NMR (101 MHz, $CDCl_3$) δ 171.7, 155.1, 79.4, 60.5, 49.5, 40.6, 28.6, 22.5, 19.1, 15.5, 14.3.
HRMS (ESI+) (m/z): $[M+Na]^+$ calcd. for $C_{13}H_{25}NO_4$, 282.1681; found: 282.1680.

12.5 Characterization of compounds 68 – 71

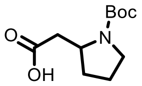

***2-(1-(tert-butoxycarbonyl)pyrrolidin-2-yl)acetic acid* (68).**

Compound **68** was synthesized adapting a procedure reported in the literature.[36]

Compound **57** (693 mg, 2.85 mmol, 1.0 equiv.) was dissolved in 10 mL of a solution 1:1 v:v of MeOH-H_2O. Then, NaOH (114 mg, 2.85 mmol, 1.0 equiv.) was added and the obtained solution was heated at reflux temperature for 5 hours. The solvent was removed under reduced pressure and the crude residue was partitioned in a 1:1 v:v solution of diethyl ether: H_2O (20 mL). The water phase was washed three times with diethyl ether (10 mL), acidified until pH=3 using 1M HCl and extracted three times with diethyl ether (10 mL). The organic layer was dried over sodium sulfate and the solvent evaporated under reduced pressure to afford **68** in quantitative yield as a white solid (613 mg, quant. yield).

Characterization data are in accordance with literature.[37]

^{1}H NMR (400 MHz, $CDCl_3$) δ 4.21 – 4.04 (m, 1H), 3.46 – 3.25 (m, 2H), 3.07 – 2.78 (m, 1H), 2.46 – 2.29 (m, 1H), 2.17 – 1.96 (m, 1H), 1.94 – 1.75 (m, 3H), 1.45 (s, 9H).

^{13}C NMR (101 MHz, $CDCl_3$) δ 177.0, 155.0, 80.1, 54.0, 46.4, 39.4, 31.4, 28.6.

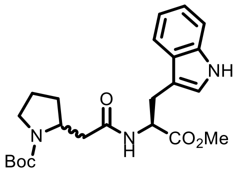

***tert-butyl-2-(2-(((S)-3-(1H-indol-3-yl)-1-methoxy-1-oxopropan-2-yl)amino)-2-oxoethyl)pyrrolidine-1-carboxylate* (69).**

Compound **69** was synthesized adapting a procedure reported in the literature.[38]

Compound **68** (racemic, 613 mg, 2.85 mmol, 1.0 equiv.) and L-tryptophan methyl ester hydrochloride (723 mg, 2.85 mmol, 1.0 equiv.) were dissolved in 28.5 mL of dry DMF. Then EDCI·HCl (563 mg, 3.14 mmol, 1.1 equiv.) and TEA (993 µL, 7.13 mmol, 2.5 equiv.) were added under a nitrogen atmosphere at 0° and stirred at the same temperature for 1 hour. The reaction was left stirring warming to room temperature overnight. Then, 10 mL of an HCl 0.1 M solution was added and the aqueous phase was extracted three times with DCM (20 mL). The organic layer was washed with 20 mL of saturated $NaHCO_3$ solution, 20 mL of Brine, 20 mL of H_2O and dried over sodium sulfate. The solvent was then evaporated under reduced pressure to afford the crude dipeptide. Purified via flash column chromatography on silica gel (Pentane:Ethyl Acetate 20:1) to afford product **69** (1:1 mixture of rotamers) as an inseparable mixture of diastereomers as a yellowish foam (870 mg, 84% yield).

^{1}H NMR (400 MHz, $CDCl_3$) δ 8.64 – 8.31 (br m, 1H, *Ind*-NH), 7.53 (d, J = 8 Hz, 1H), 7.34 (d, J = 8 Hz, 1H), 7.17 (t, J = 7 Hz, 1H), 7.10 (dt, J = 7, 2 Hz, 1H), 7.01 – 6.95 (m, 1H), 6.80 – 6.43 & 6.18 – 5.76 (rotameric br m, 1H, NH), 5.05 – 4.80 (m, 1H), 4.19 – 3.90 (m, 1H), 3.80 – 3.54 (br s, 3H, OCH_3), 3.44 – 3.15 (m, 4H), 2.84 – 2.53 (m, 1H), 2.28 – 2.07 (m, 1H), 1.99 – 1.85 (m, 1H), 1.84 – 1.67 (m, 3H), 1.50 – 1.35 (m, 9H).

^{13}C NMR (101 MHz, $CDCl_3$) δ 172.6, 170.8, 154.7, 136.3, 127.7, 122.9, 122.2, 119.7, 118.6, 111.4, 110.0, 79.6, 54.9, 52.9, 52.4, 46.8, 41.1, 30.8, 28.6, 27.8, 23.6.

HRMS (ESI+) (m/z): $[M+H]^+$ calcd. for $C_{23}H_{31}N_3O_5$, 430.2342; found: 430.2349.

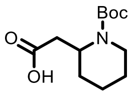

***2-(1-(tert-butoxycarbonyl)piperidin-2-yl)acetic acid* (70).**

Compound **70** was synthesized adapting a procedure reported in the literature.[36]

Compound **58** (836 mg, 3.25 mmol, 1.0 equiv.) was dissolved in 12 mL of a solution 1:1 v:v of MeOH-H_2O. Then, NaOH (130 mg, 3.25 mmol, 1.0 equiv.) was added and the obtained solution was heated at reflux temperature for 5 hours. The solvent was removed under reduced pressure and the crude residue was partitioned in a 1:1 v:v solution of diethyl ether: H_2O (25 mL). The water phase was washed three times with diethyl ether (15 mL), acidified until pH=3 using 1M HCl and extracted three times with diethyl ether (15 mL). The organic layer was dried over sodium sulfate and the solvent evaporated under reduced pressure to afford **70** in quantitative yield as a white solid (745 mg).

Characterization data are in accordance with literature.[37]

^{1}H NMR (400 MHz, CDCl3) δ 4.76 – 4.60 (m, 1H), 3.99 (d, J = 13 Hz, 1H), 2.77 (t, J = 13 Hz, 1H), 2.67 – 2.49 (m, 2H), 1.63 (d, J = 11 Hz, 4H), 1.51 – 1.38 (m, 11H).

^{13}C NMR (101 MHz, $CDCl_3$) δ 176.9, 155.1, 80.0, 47.9, 39.3, 35.4, 28.5, 25.3, 19.0.

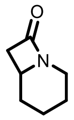

***1-azabicyclo[4.2.0]octan-8-one* (71).**

Compound **71** was synthesized adapting a procedure reported in the literature.[39]

Compound **70** (745 mg, 3.25 mmol, 1.0 equiv.) was dissolved in 17 mL of glacial acetic acid and 5.4 mL of a 6 M solution of HCl in Dioxane were added. The resulting solution was stirred for 30 minutes at room temperature. Then the solvent was removed under reduced pressure. The obtained crude was triturated with diethyl ether to give the corresponding salt after vacuum filtration. To a stirred suspension of the salt in 150 mL of dry dichloromethane was added 2-chloro-1-methyl pyridinium iodide (913 mg, 3.58 mmol, 1.0 equiv.) and TEA (1.36 mL, 9.75 mmol, 3.0 equiv.) in 6 mL of dry dichloromethane. The reaction was stirred six hours at room temperature and then the solvent was removed under reduced pressure. Purified via flash column chromatography on silica gel (Pentane:Ethyl Acetate 50:50) to afford product **71** as a yellowish oil (244 mg, 60% yield).

^{1}H NMR (300 MHz, $CDCl_3$) δ 3.81 (dd, J = 13, 5 Hz, 1H), 3.40 – 3.26 (m, 1H), 3.06 (ddd, J = 14, 4, 2 Hz, 1H), 2.77 – 2.63 (m, 1H), 2.56 (dd, J = 14, 2 Hz, 1H), 2.13 – 1.99 (m, 1H), 1.97 – 1.81 (m, 1H), 1.70 – 1.57 (m, 1H), 1.52 – 1.32 (m, 2H), 1.32 – 1.16 (m, 1H).j

^{13}C NMR (75 MHz, $CDCl_3$) δ 166.2, 47.5, 44.8, 39.1, 31.1, 24.7, 22.5.

13. Limitation of the scope

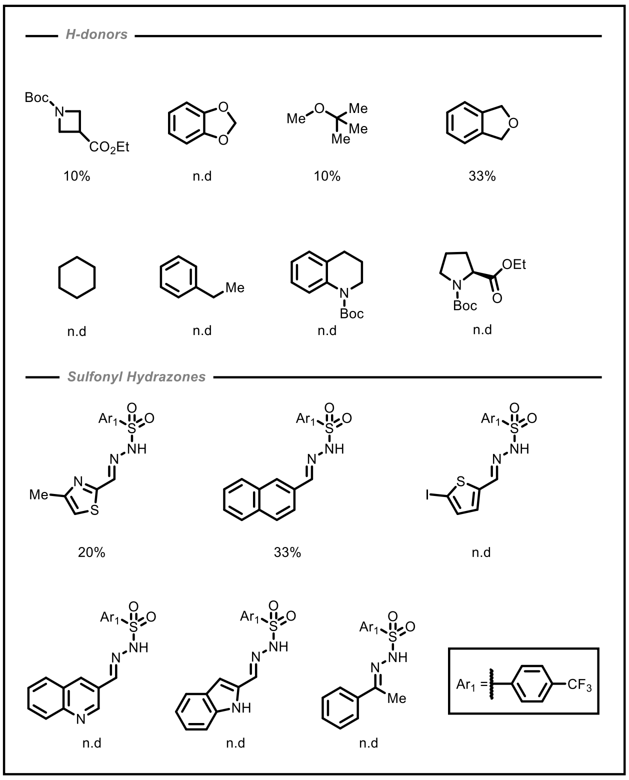

14. References

(1) Protti, S.; Ravelli, D.; Fagnoni, M.; Albini, A. Solar Light-Driven Photocatalyzed Alkylations. Chemistry on the Window Ledge. *Chem. Commun.* **2009**, 47, 7351–7353.

(2) Shen, Y.; Gu, Y.; Martin, R. Sp^3 C–H Arylation and Alkylation Enabled by the Synergy of Triplet Excited Ketones and Nickel Catalysts. *J. Am. Chem. Soc.* **2018**, *140*, 12200–12209.

(3) Capaldo, L.; Bonciolini, S.; Pulcinella, A.; Nuño, M.; Nöel, T. Modular Allylation of $C(Sp^3)$ –H Bonds by Combining Decatungstate Photocatalysis and HWE Olefination in Flow. *Chem. Sci.* **2022**, *13*, 7325-7331.

(4) Ackerman, L. K. G.; Martinez Alvarado, J. I.; Doyle, A. G. Direct C–C Bond Formation from Alkanes Using Ni-Photoredox Catalysis. *J. Am. Chem. Soc.* **2018**, *140*, 14059–14063.

(5) Abadie, B.; Jardel, D.; Pozzi, G.; Toullec, P.; Vincent, J. Dual Benzophenone/Copper-Photocatalyzed Giese-Type Alkylation of $C(Sp^3)$–H Bonds. *Chem. Eur. J.* **2019**, *25*, 16120–16127.

(6) Cons, B. D.; Bunt, A. J.; Bailey, C. D.; Willis, C. L. Total Synthesis of (-)-Blepharocalyxin D and Analogues. *Org. Lett.* **2013**, *15*, 2046–2049.

(7) Slade, D. J.; Pelz, N. F.; Bodnar, W.; Lampe, J. W.; Watson, P. S. Indazoles: Regioselective Protection and Subsequent Amine Coupling Reactions. *J. Org. Chem.* **2009**, *74*, 6331–6334.

(8) Huang, H.; Li, X.; Yu, C.; Zhang, Y.; Mariano, P. S.; Wang, W. Visible-Light-Promoted Nickel- and Organic-Dye-Cocatalyzed Formylation Reaction of Aryl Halides and Triflates and Vinyl Bromides with Diethoxyacetic Acid as a Formyl Equivalent. *Angew. Chem. Int. Ed.* **2017**, *129*, 1522–1527.

(9) Han, X.; Civiello, R. L.; Fang, H.; Wu, D.; Gao, Q.; Chaturvedula, P. v.; Macor, J. E.; Dubowchik, G. M. Catalytic Asymmetric Syntheses of Tyrosine Surrogates. *J. Org. Chem.* **2008**, *73*, 8502–8510.

(10) Pearson, M. S. M.; Carbery, D. R. Studies toward the Photochemical Synthesis of Functionalized [5]- and [6] Carbohelicenes. *J. Org. Chem.* **2009**, *74*, 5320–5325.

(11) Ou, J.; He, S.; Wang, W.; Tan, H.; Liu, K. Highly Efficient Oxidative Cleavage of Olefins with O_2 under Catalyst-, Initiator- And Additive-Free Conditions. *Org. Chem. Front.* **2021**, *8*, 3102–3109.

(12) Jiang, X.; Wang, G.; Zheng, Z.; Yu, X.; Hong, Y.; Xia, H.; Yu, C. Autocatalytic Synthesis of Thioesters via Thiocarbonylation of Gem-Difluoroalkenes. *Org. Lett.* **2020**, *22*, 9762–9766.

(13) Zhang, S.; Perveen, S.; Ouyang, Y.; Xu, L.; Yu, T.; Zhao, M.; Wang, L.; Song, P.; Li, P. Design and Synthesis of Tunable Chiral 2,2′-Bipyridine Ligands: Application to the Enantioselective Nickel-Catalyzed Reductive Arylation of Aldehydes. *Angew. Chem. Int. Ed.* **2022**, *61*, e202117843.

(14) Vásquez-Céspedes, S.; Ferry, A.; Candish, L.; Glorius, F. Heterogeneously Catalyzed Direct C–H Thiolation of Heteroarenes. *Angew. Chem. Int. Ed.* **2015**, *127*, 5864–5868.

(15) Costa, S. P. G.; Batista, R. M. F.; Cardoso, P.; Belsley, M.; Raposo, M. M. M. 2-Arylthienyl-Substituted 1,3-Benzothiazoles as New Nonlinear Optical Chromophores. *Eur. J. Org. Chem.* **2006**, *17*, 3938–3946.

(16) Iwasaki, T.; Imanishi, R.; Shimizu, R.; Kuniyasu, H.; Terao, J.; Kambe, N. Copper-Catalyzed Alkyl-Alkyl Cross-Coupling Reactions Using Hydrocarbon Additives: Efficiency of Catalyst and Roles of Additives. *J. Org. Chem.* **2014**, *79*, 8522–8532.

(17) Stevens, J. M.; MacMillan, D. W. C. Enantioselective α-Alkenylation of Aldehydes with Boronic Acids via the Synergistic Combination of Copper(II) and Amine Catalysis. *J. Am. Chem. Soc.* **2013**, *135*, 11756–11759.

(18) Cao, H. Q.; Liu, H. N.; Liu, Z. Y.; Qiao, B.; Zhang, F. G.; Ma, J. A. Silver-Promoted Direct Phosphorylation of Bulky C(Sp^2) –H Bond to Build Fully Substituted β-Phosphonodehydroamino Acids. *Org. Lett.* **2020**, *22*, 6414–6419.

(19) Cao, H.-Q.; Liu, H.-N.; Liu, Z.-Y.; Qiao, B.; Zhang, F.-G.; Ma, J.-A. Silver-Promoted Direct Phosphorylation of Bulky C(Sp^2)–H Bond to Build Fully Substituted β-Phosphonodehydroamino Acids. *Org. Lett.* **2020**, *22*, 6414–6419.

(20) Zhao, X.; Zhang, L.; Li, T.; Liu, G.; Wang, H.; Lu, K. P-Toluenesulphonic Acid-Promoted, I2-Catalysed Sulphenylation of Pyrazolones with Aryl Sulphonyl Hydrazides. *Chem. Commun.* **2014**, *50*, 13121–13123.

(21) Saladrigas, M.; Bonjoch, J.; Bradshaw, B. Iron Hydride Radical Reductive Alkylation of Unactivated Alkenes. *Org. Lett.* **2020**, *22*, 684–688.

(22) Wan, T.; Capaldo, L.; Laudadio, G.; Nyuchev, A. v.; Rincón, J. A.; García-Losada, P.; Mateos, C.; Frederick, M. O.; Nuño, M.; Noël, T. Decatungstate-Mediated C(Sp^3)–H Heteroarylation via Radical-Polar Crossover in Batch and Flow. *Angew. Chem. Int. Ed* **2021**, *60*, 17893–17897.

(23) Ni, M.; Zhang, J.; Liang, X.; Jiang, Y.; Loh, T.-P. Directed C–C Bond Cleavage of a Cyclopropane Intermediate Generated from *N* -Tosylhydrazones and Stable Enaminones: Expedient Synthesis of Functionalized 1,4-Ketoaldehydes. *Chem. Commun.* **2017**, *53*, 12286–12289.

(24) Allouche, E. M. D.; Al-Saleh, A.; Charette, A. B. Iron-Catalyzed Synthesis of Cyclopropanes by *in Situ* Generation and Decomposition of Electronically Diversified Diazo Compounds. *Chem. Commun.* **2018**, *54*, 13256–13259.

(25) Tsui, E.; Metrano, A. J.; Tsuchiya, Y.; Knowles, R. R. Catalytic Hydroetherification of Unactivated Alkenes Enabled by Proton-Coupled Electron Transfer. *Angew. Chem. Int. Ed* **2020**, *59*, 11845–11849.

(26) Sahoo, B.; Hopkinson, M. N.; Glorius, F. Combining Gold and Photoredox Catalysis: Visible Light-Mediated Oxy- and Aminoarylation of Alkenes. *J. Am. Chem. Soc.* **2013**, *135*, 5505–5508.

(27) Santos, M. S.; Corrêa, A. G.; Paixão, M. W.; König, B. C(Sp^3)–C(Sp^3) Cross-Coupling of Alkyl Bromides and Ethers Mediated by Metal and Visible Light Photoredox Catalysis. *Adv. Synth. Catal.* **2020**, *362*, 2367–2372.

(28) Hay, M. B.; Hardin, A. R.; Wolfe, J. P. Palladium-Catalyzed Synthesis of Tetrahydrofurans from γ-Hydroxy Terminal Alkenes: Scope, Limitations, and Stereoselectivity. *J. Org. Chem.* **2005**, 70, 3099–3107.

(29) Zhang, L.; Si, X.; Yang, Y.; Zimmer, M.; Witzel, S.; Sekine, K.; Rudolph, M.; Hashmi, A. S. K. The Combination of Benzaldehyde and Nickel-Catalyzed Photoredox C(Sp^3)–H Alkylation/Arylation. *Angew. Chem. Int. Ed.* **2019**, *58*, 1823–1827.

(30) Molander, G. A.; Argintaru, O. A.; Aron, I.; Dreher, S. D. Nickel-Catalyzed Cross-Coupling of Potassium Aryl- and Heteroaryltrifluoroborates with Unactivated Alkyl Halides. *Org. Lett.* **2010**, *12*, 5783–5785.

(31) Zhang, Z.; Cernak, T. The Formal Cross-Coupling of Amines and Carboxylic Acids to Form Sp^3–Sp^3 Carbon–Carbon Bonds. *Angew. Chem. Int. Ed.* **2021**, *60*, 27293–27298.

(32) Massah, A. R.; Ross, A. J.; Jackson, R. F. W. In Situ Trapping of Boc-2-Pyrrolidinylmethylzinc Iodide with Aryl Iodides: Direct Synthesis of 2-Benzylpyrrolidines. *J. Org. Chem.* **2010**, *75*, 8275–8278.

(33) Bellur, E.; Freifeld, I.; Böttcher, D.; Bornscheuer, U. T.; Langer, P. Synthesis of (Tetrahydrofuran-2-Yl)Acetates Based on a 'Cyclization/Hydrogenation/Enzymatic Kinetic Resolution' Strategy. *Tetrahedron* **2006**, *62*, 7132–7139.

(34) Homma, K.; Takenoshita, H.; Mukaiyama, T. Stereoselective Syntheses of α-Substituted Cyclic Ethers and *Syn* -1,3-Diols. *Bull. Chem. Soc. Jpn.* **1990**, *63*, 1898–1915.

(35) Narasaka, K.; Kohno, Y. Oxidative Generation of *N* -Acyliminium Ions from *N* -1-(Tributylstannyl)Alkyl Carboxamides and Carbamates and Their Reactions with Carbon Nucleophiles. *Bull. Chem. Soc. Jpn.* **1993**, *66*, 3456–3463.

(36) Kelleher, F.; Kelly, S.; Watts, J.; McKee, V. Structure-Reactivity Relationships of l-Proline Derived Spirolactams and α-Methyl Prolinamide Organocatalysts in the Asymmetric Michael Addition Reaction of Aldehydes to Nitroolefins. *Tetrahedron* **2010**, *66*, 3525–3536.

(37) Bertini Gross, K. M.; Beak, P. Complex-Induced Proximity Effects: The Effect of Varying Directing-Group Orientation on Carbamate-Directed Lithiation Reactions. *J. Am. Chem. Soc.* **2001**, *123*, 315–321.

(38) Reimler, J.; Studer, A. Visible-Light Mediated Tryptophan Modification in Oligopeptides Employing Acylsilanes. *Chem. Eur. J.* **2021**, *27*, 15392–15395.

(39) Huang, H.; Iwasawa, N.; Mukaiyama, T. A Convenient Method for the Construction of β-Lactam Compounds from β-Amino Acids Using 2-Chloro-1-Methylpyridinium Iodide as Condensating Reagent. *Chem. Lett.* **1984**, 1465-1466.

15. **NMR spectra of sulfonylhydrazones 1a – 1aj**

^{1}H NMR (400 MHz, $CDCl_3$) of **1a**

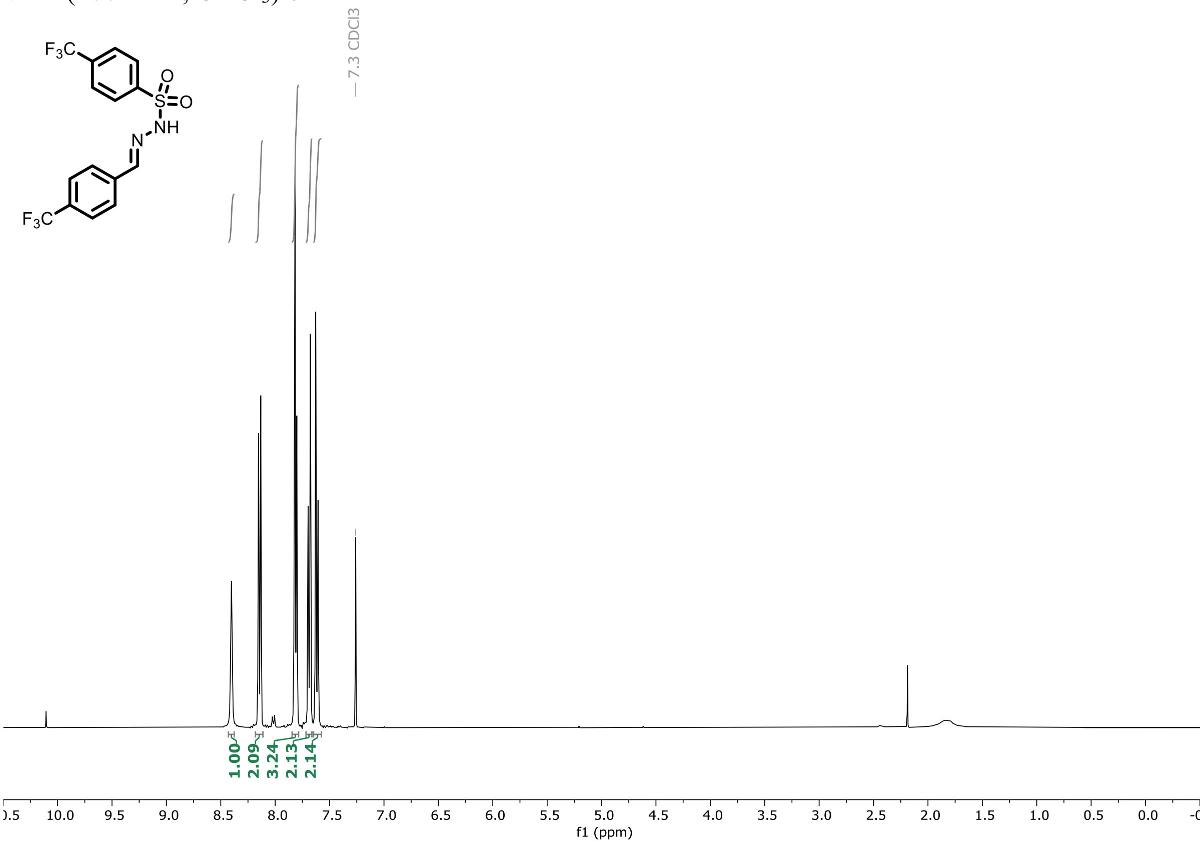

^{13}C NMR (101 MHz, $CDCl_3$) of **1a**

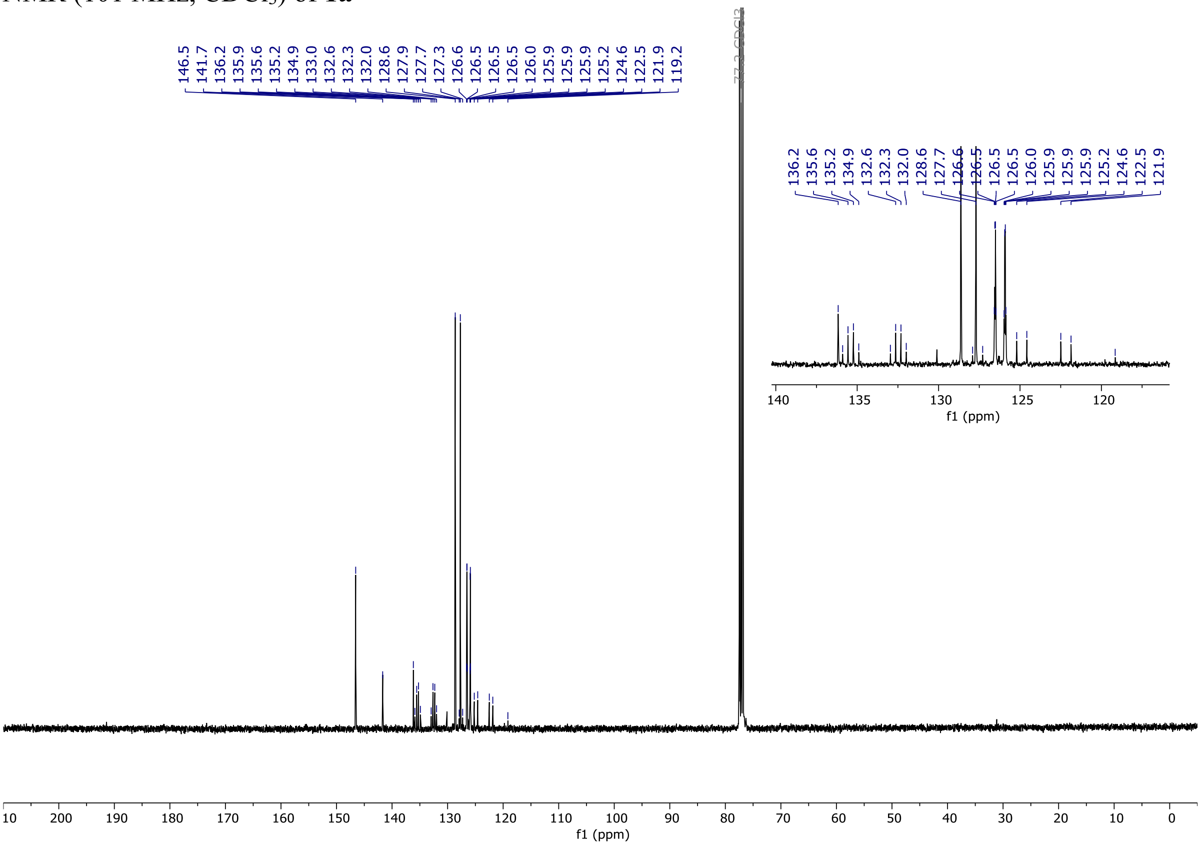

^{19}F NMR (282 MHz, $CDCl_3$) of **1a**

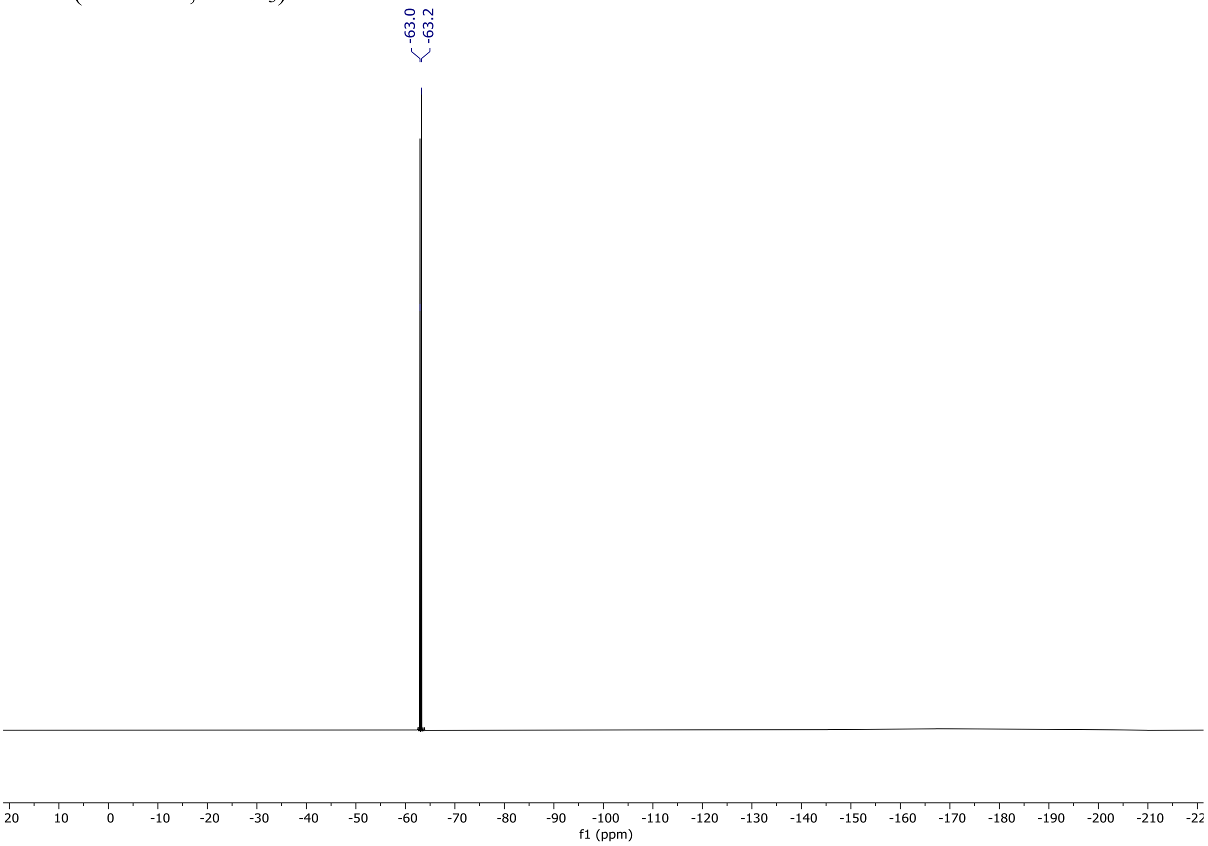

^{1}H NMR (400 MHz,CDCl$_3$) of **1b**

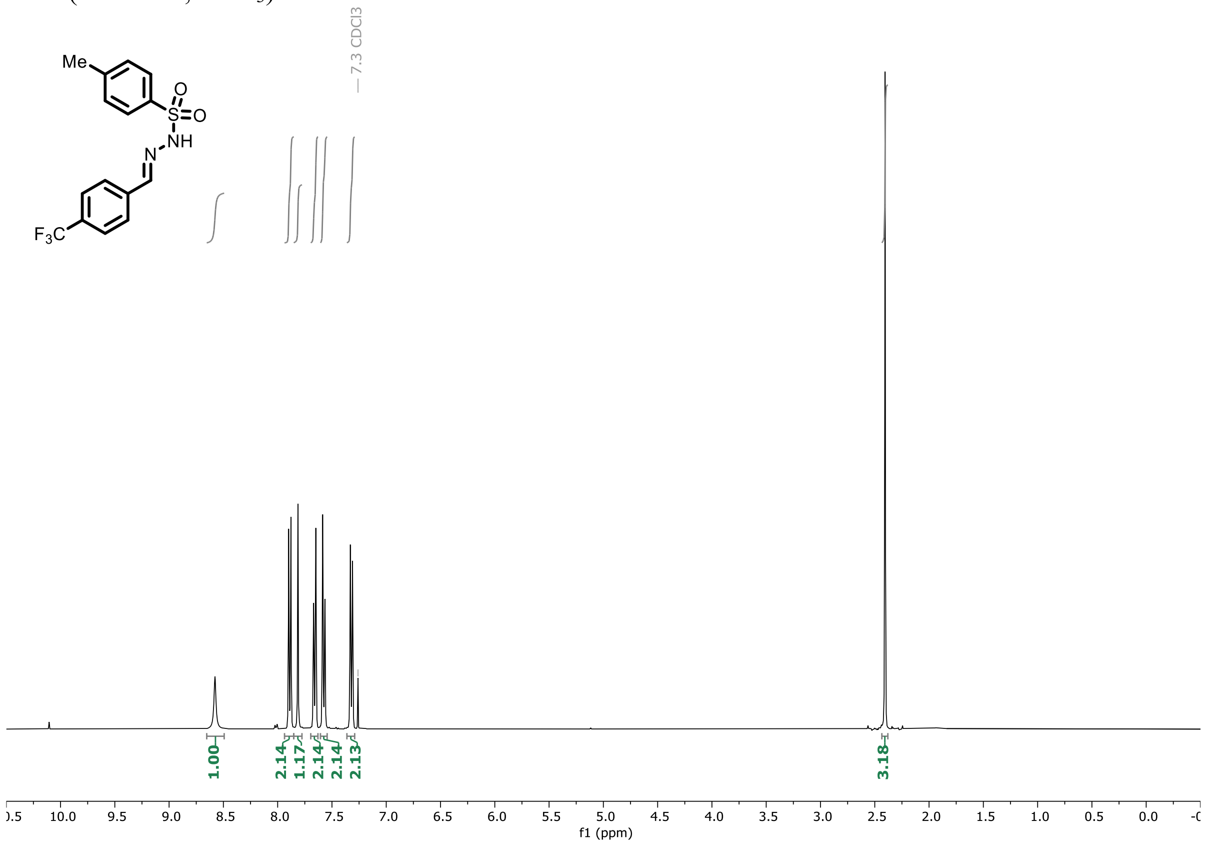

^{13}C NMR (101 MHz, CDCl$_3$) of **1b**

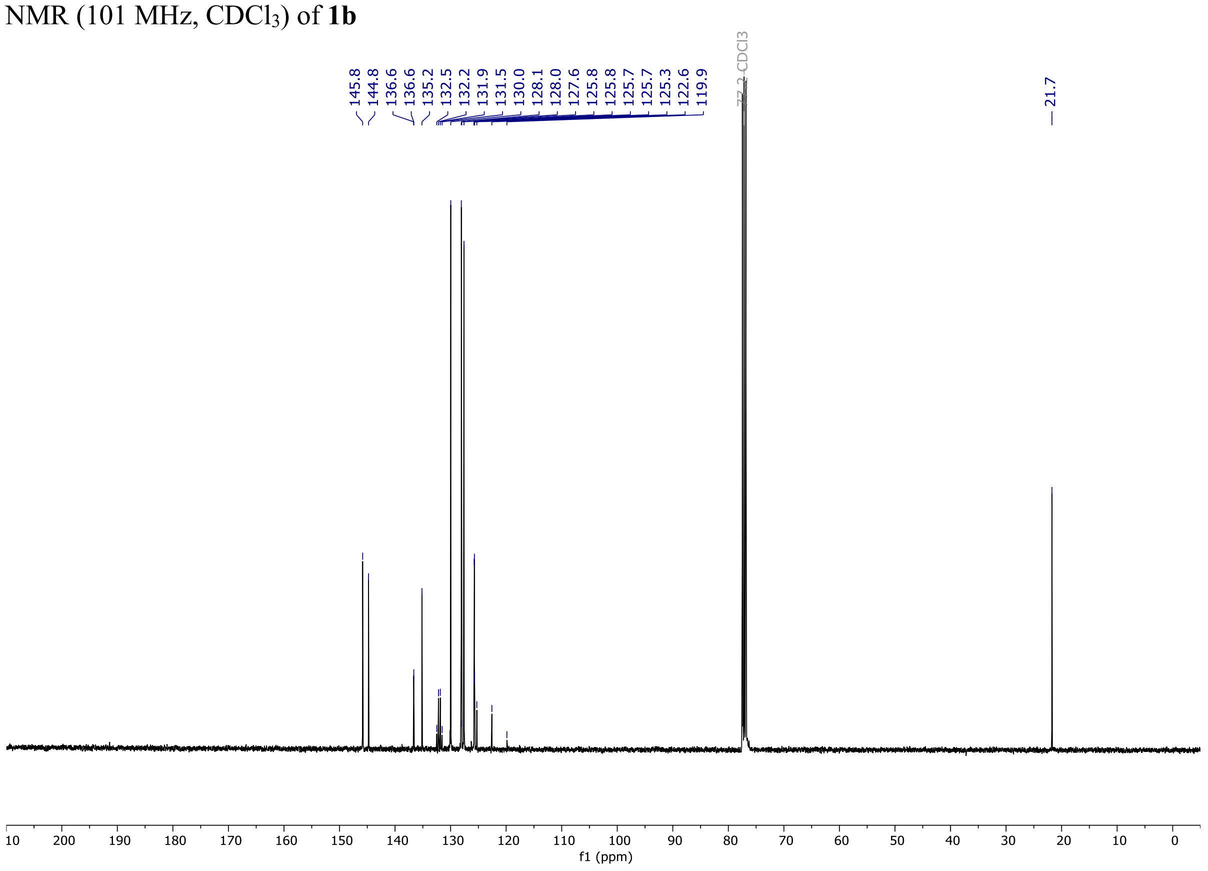

^{19}F NMR (282 MHz, $CDCl_3$) of **1b**

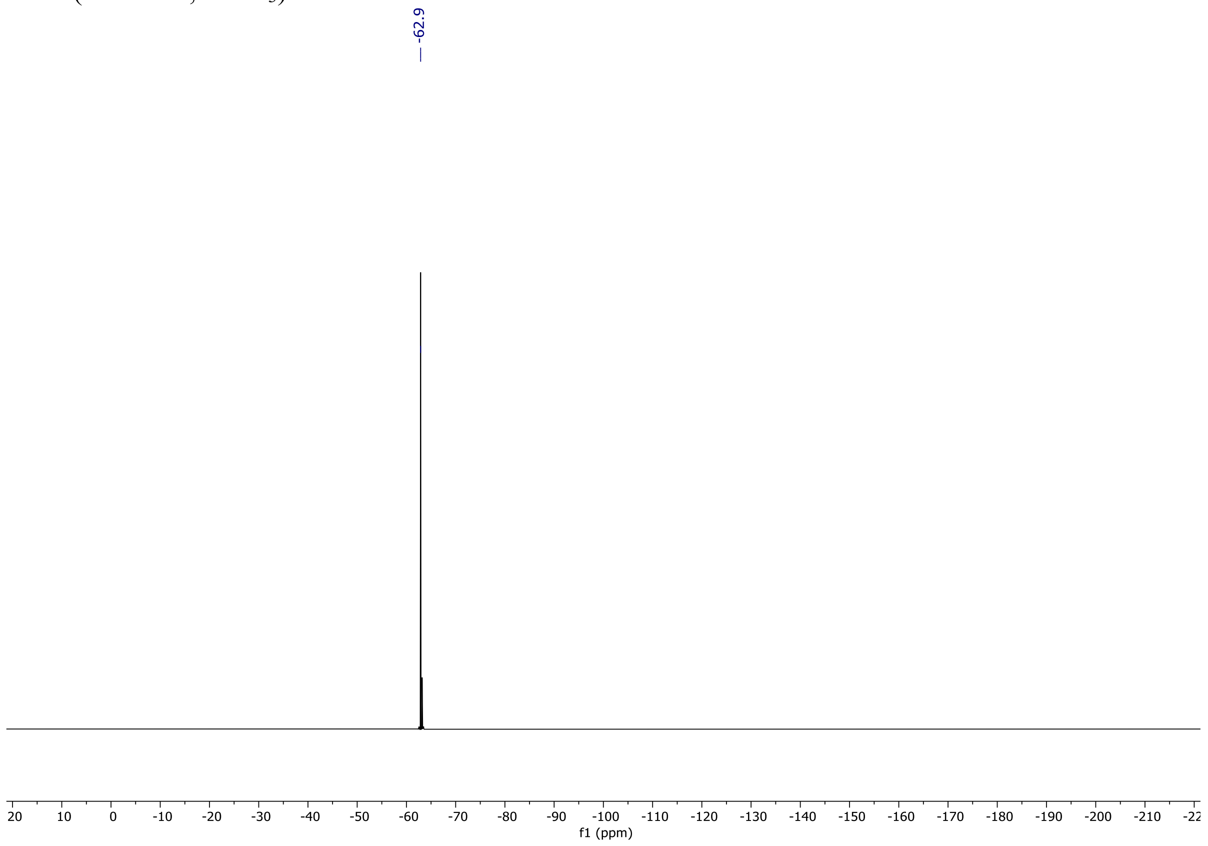

^{1}H NMR (400 MHz, DMSO) of **1c**

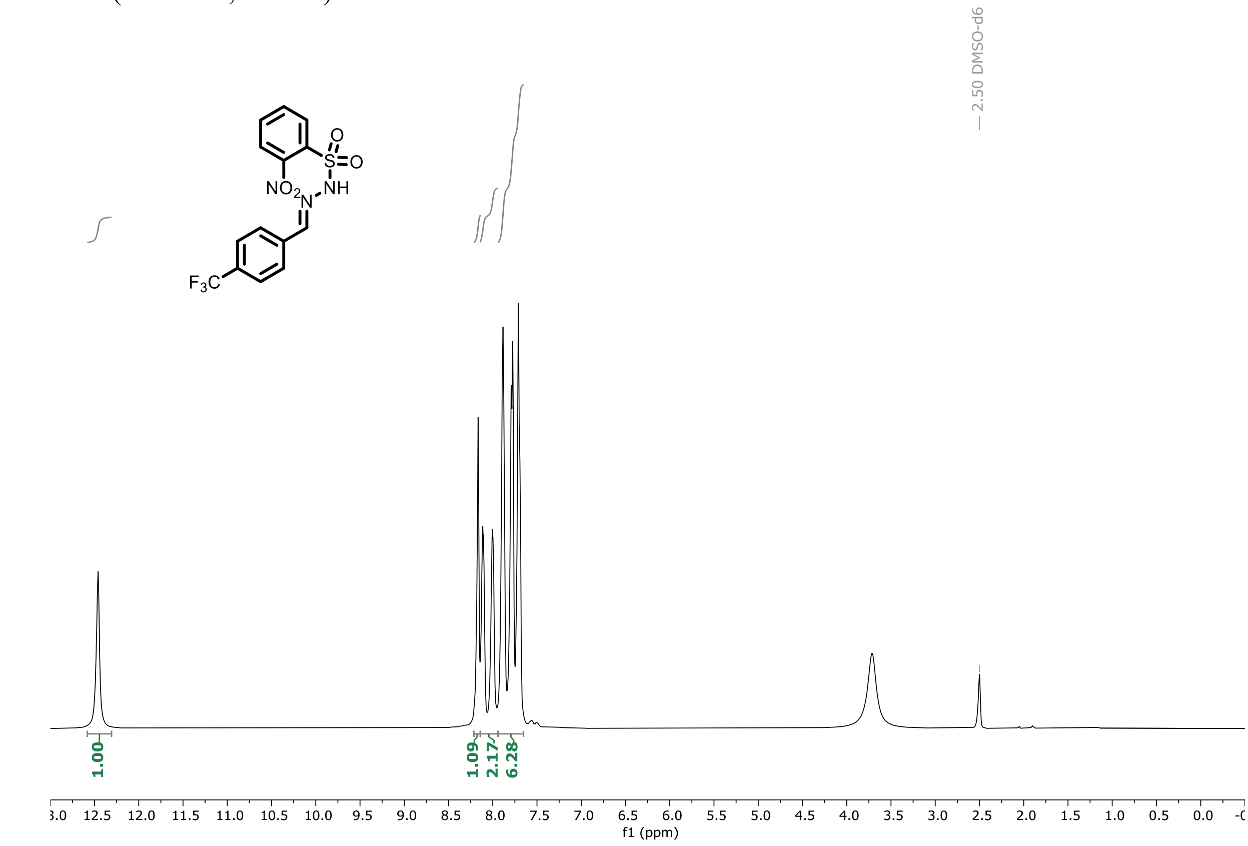

^{13}C NMR (101 MHz, DMSO) of **1c**

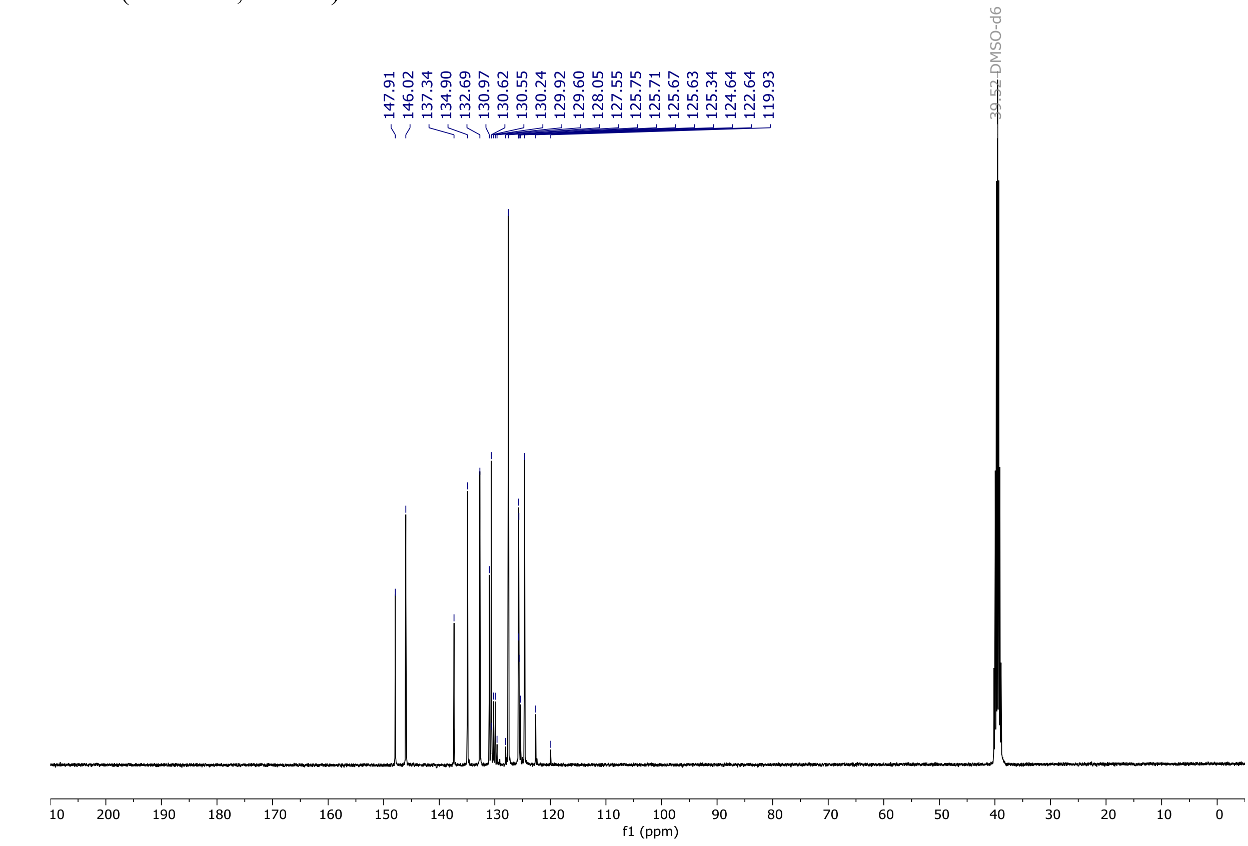

^{19}F NMR (282 MHz, DMSO) of **1c**

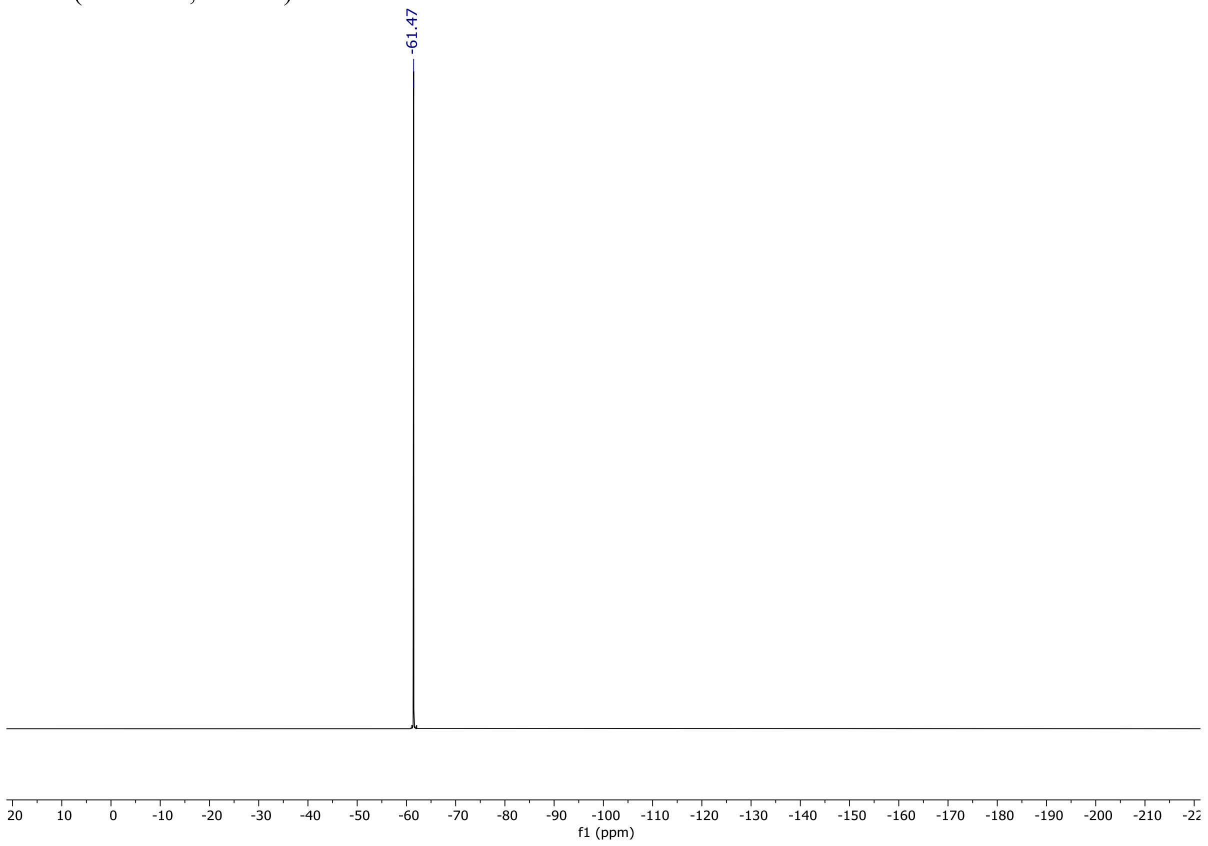

^{1}H NMR (400 MHz, DMSO) of **1d**

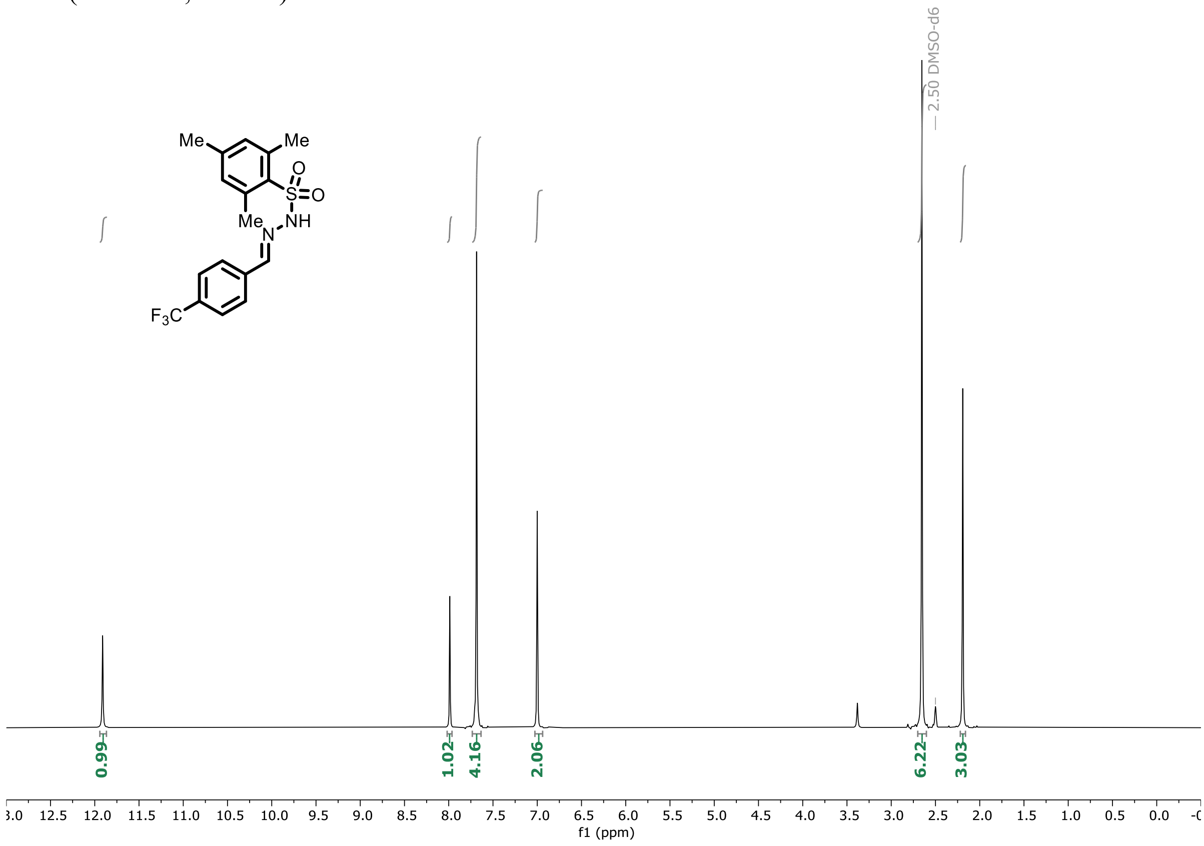

^{13}C NMR (101 MHz, DMSO) of **1d**

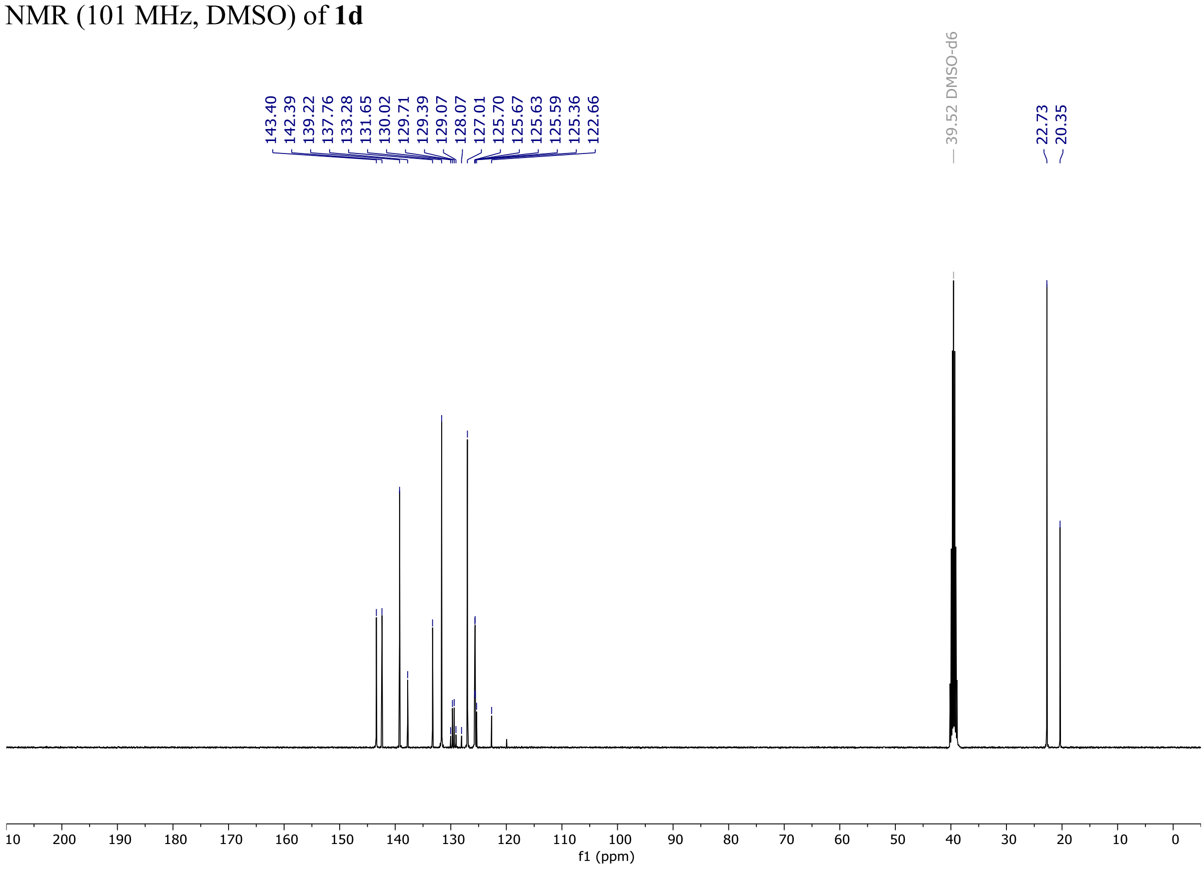

^{19}F NMR (282 MHz, DMSO) of **1d**

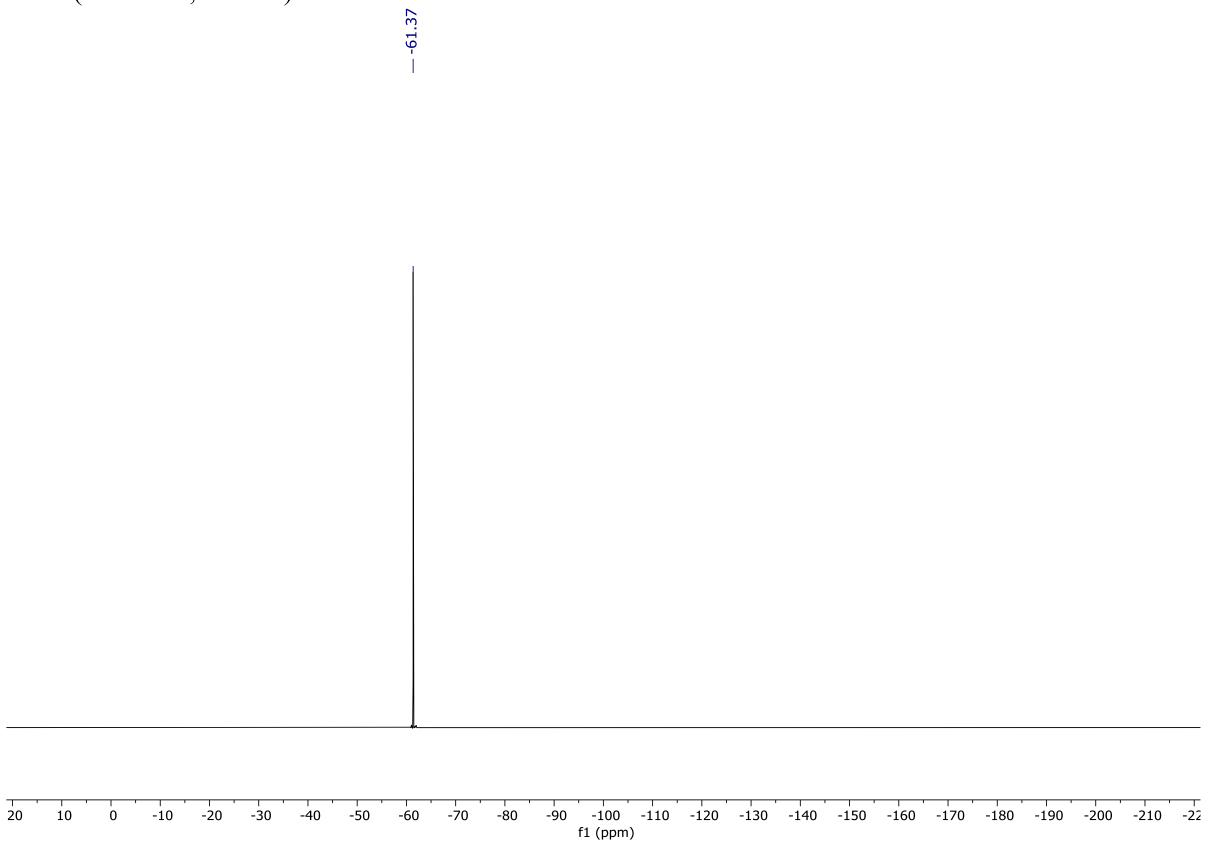

^{1}H NMR (400 MHz, DMSO) of **1e**

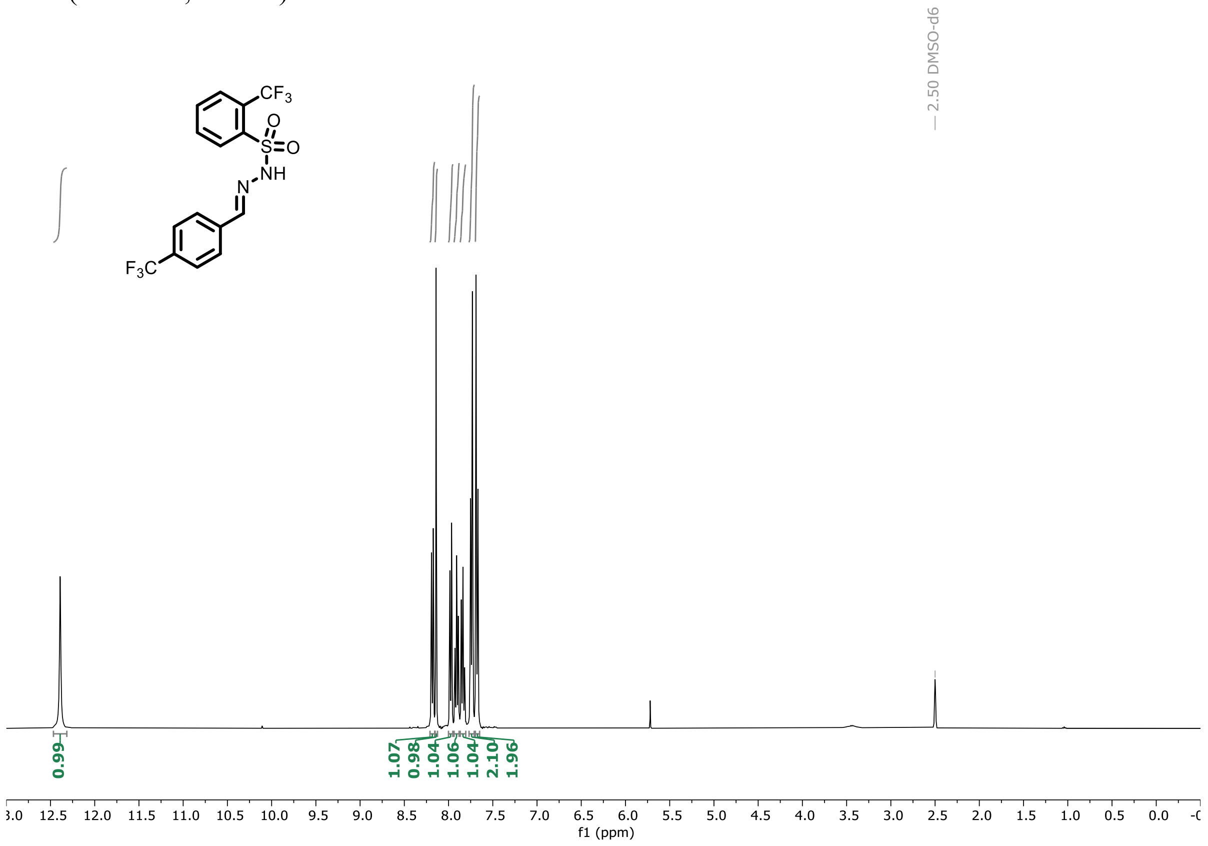

^{13}C NMR (101 MHz, DMSO) of **1e**

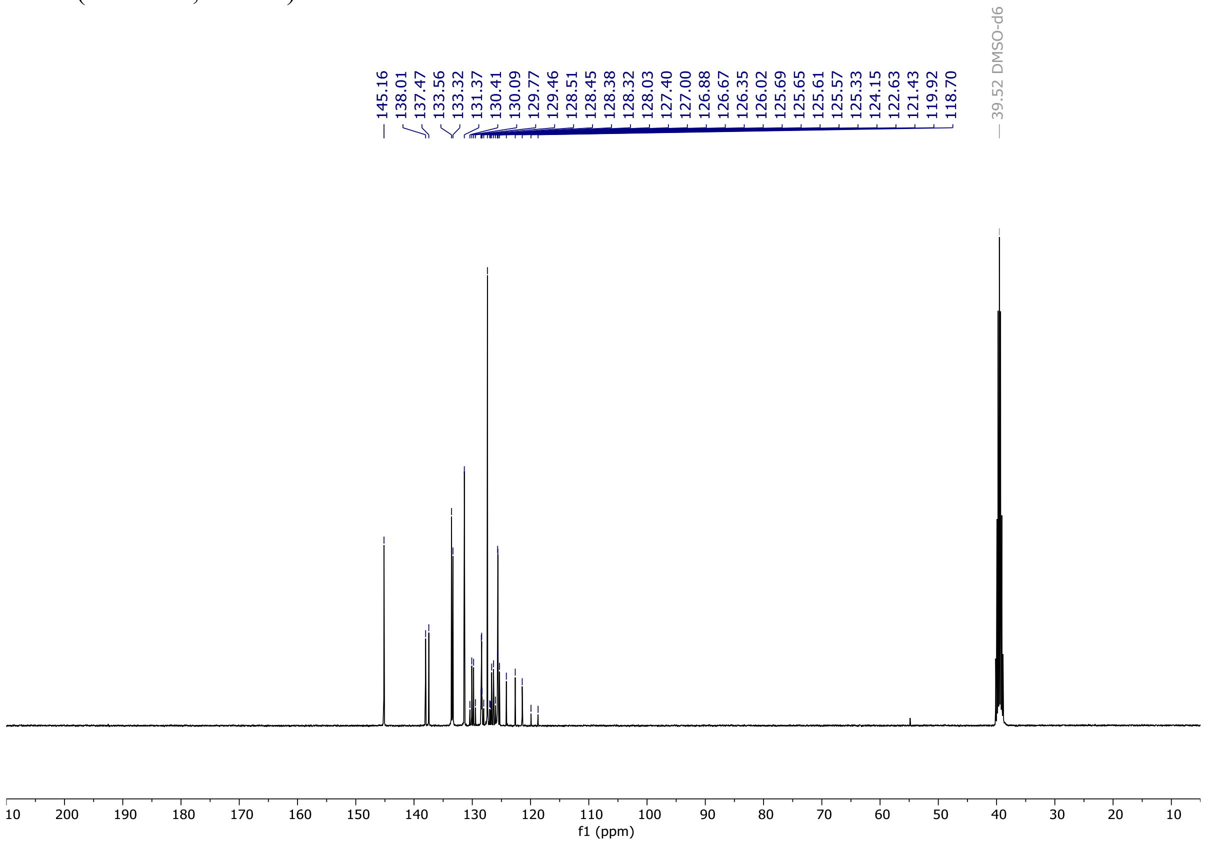

^{19}F NMR (282 MHz, DMSO) of **1e**

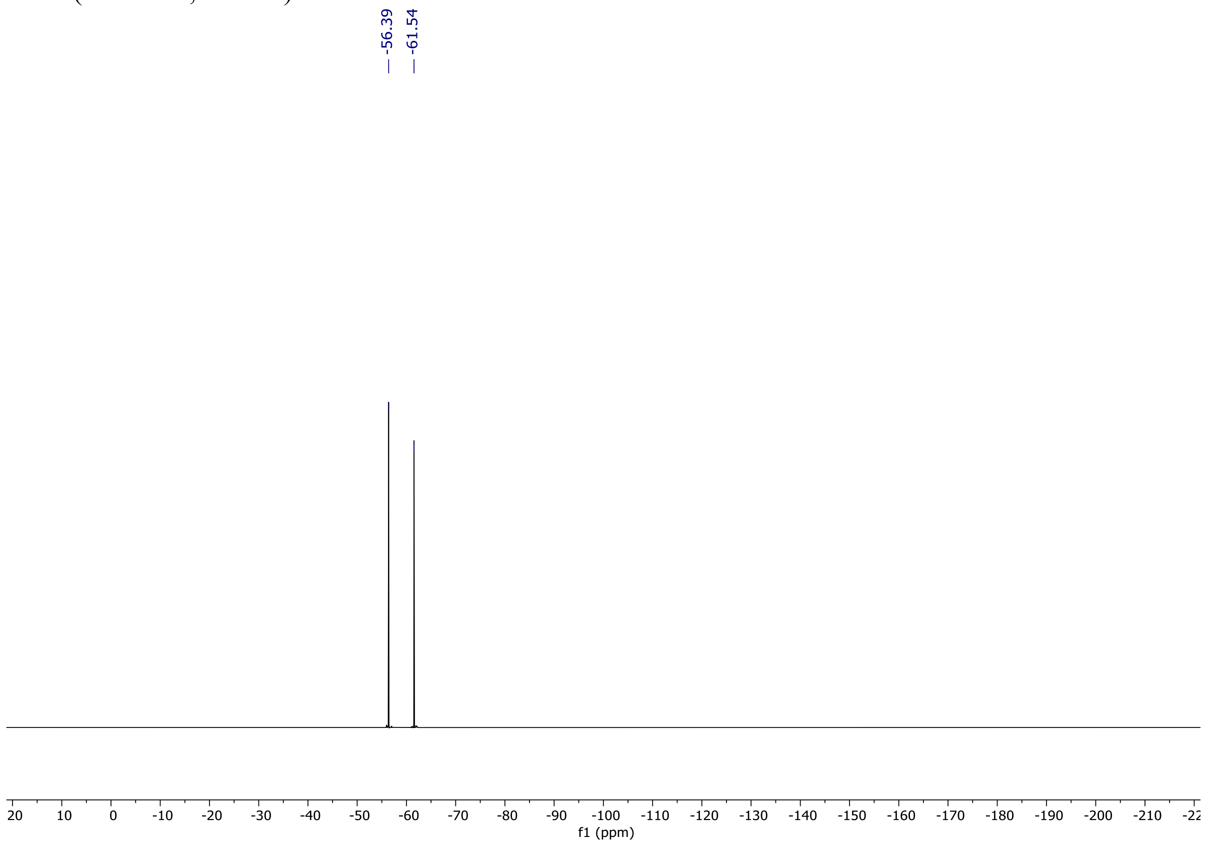

^{1}H NMR (400 MHz, DMSO) of **1f**

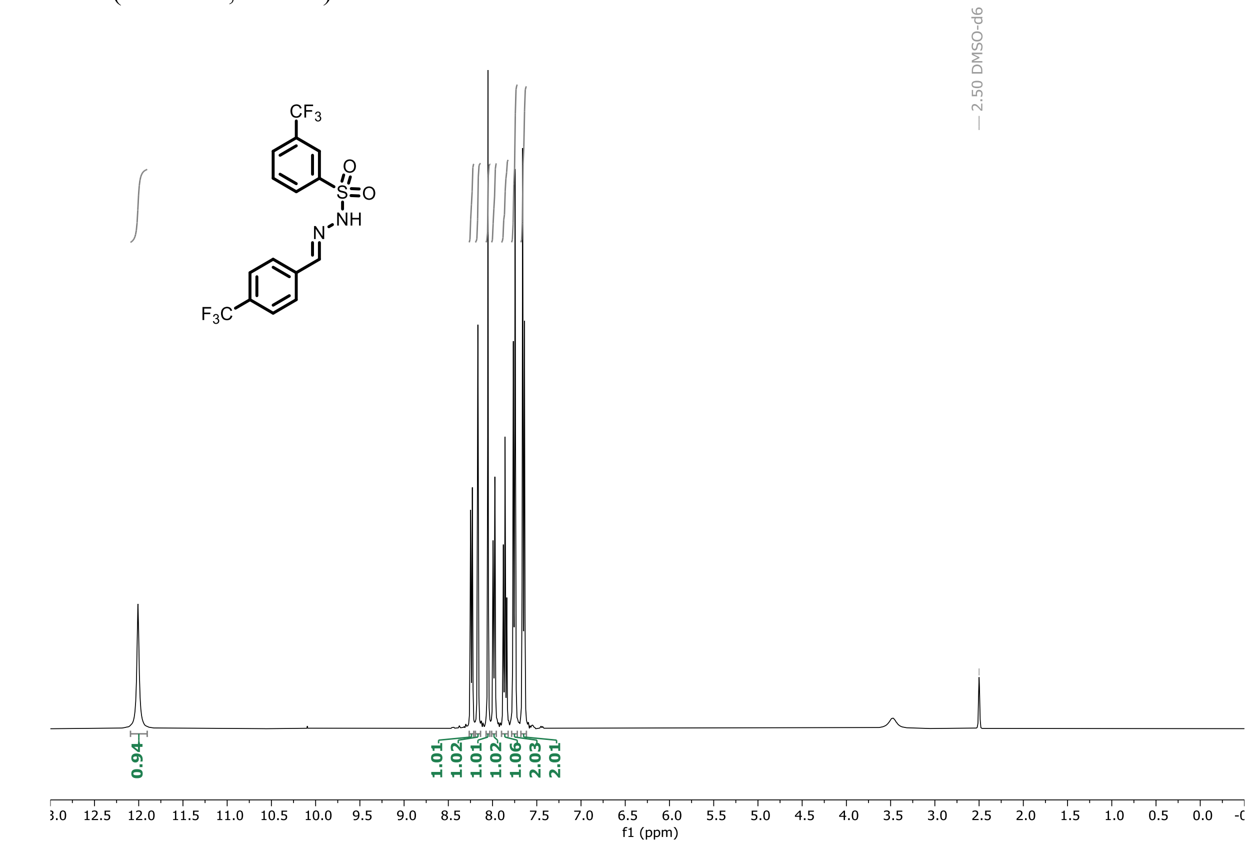

^{13}C NMR (101 MHz, DMSO) of **1f**

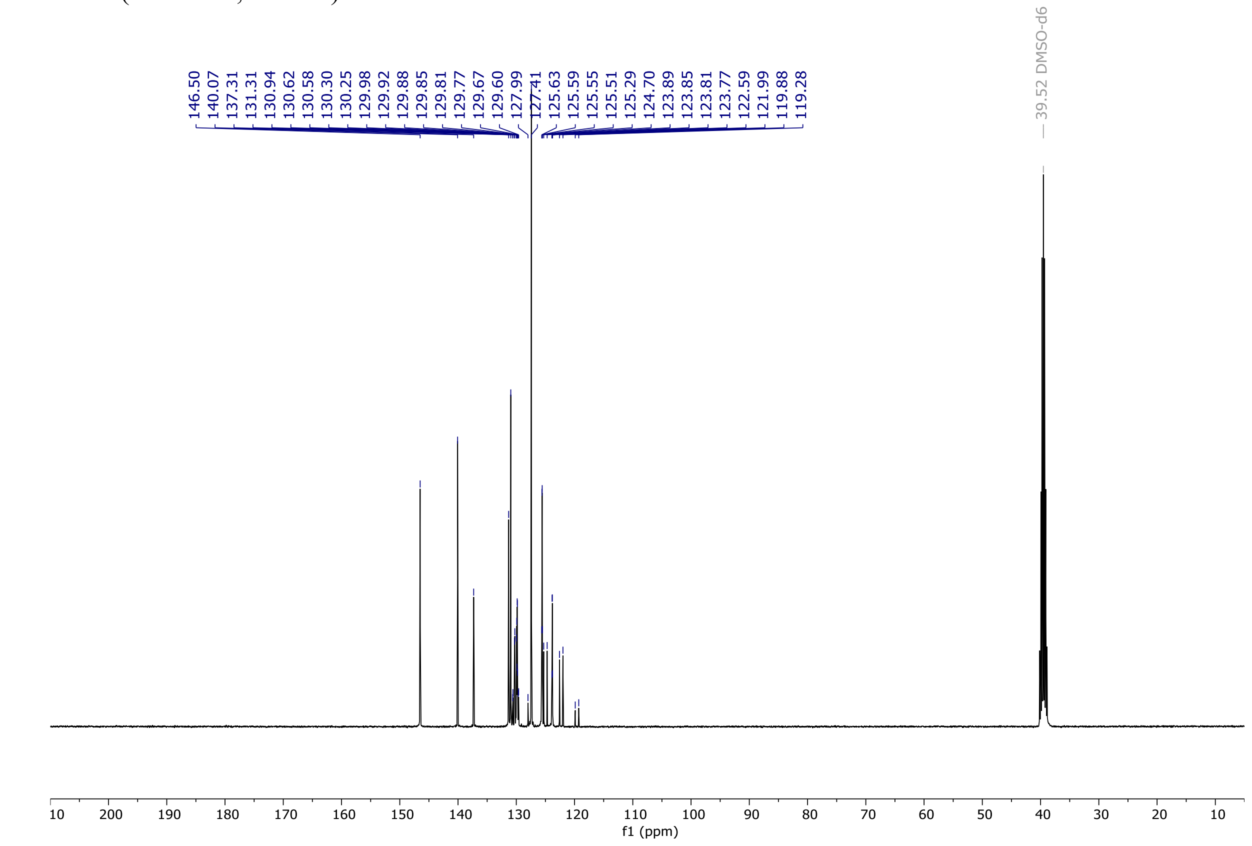

^{19}F NMR (282 MHz, DMSO) of **1f**

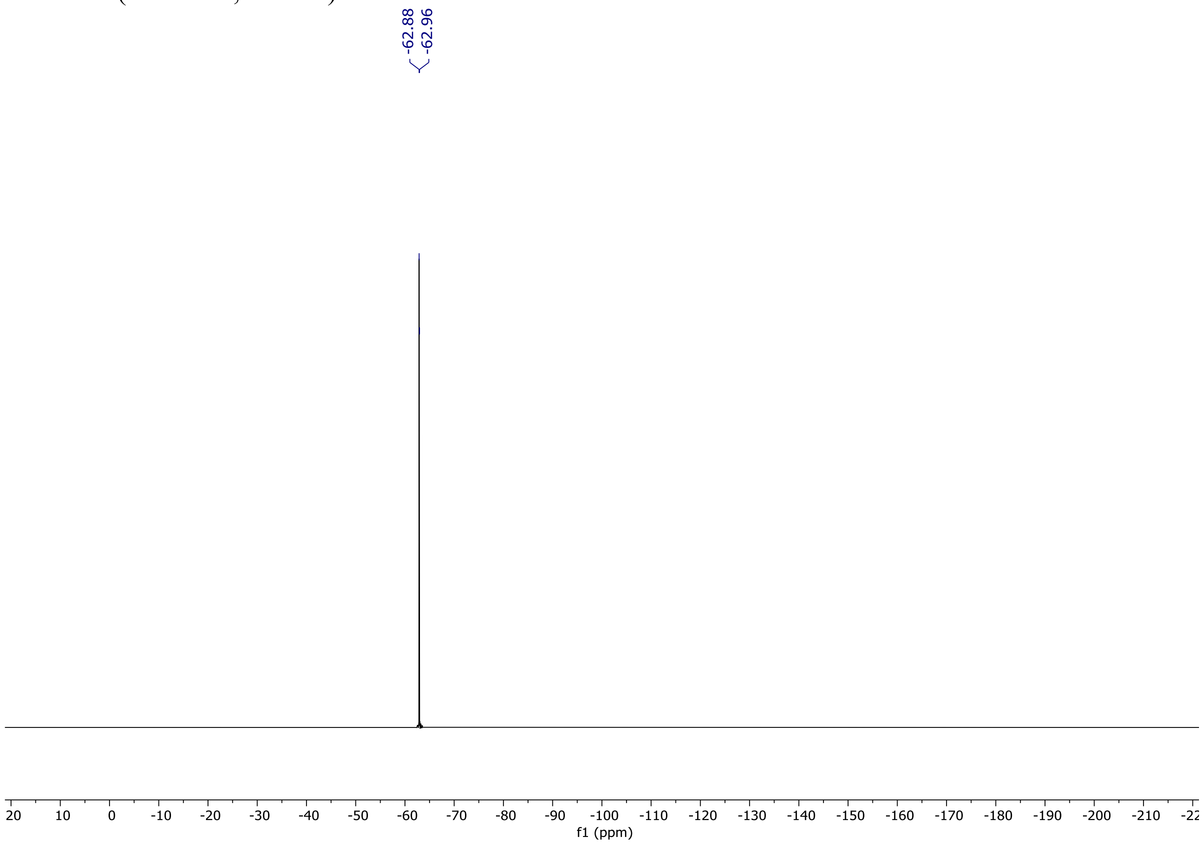

^{1}H NMR (400 MHz, DMSO) of **1g**

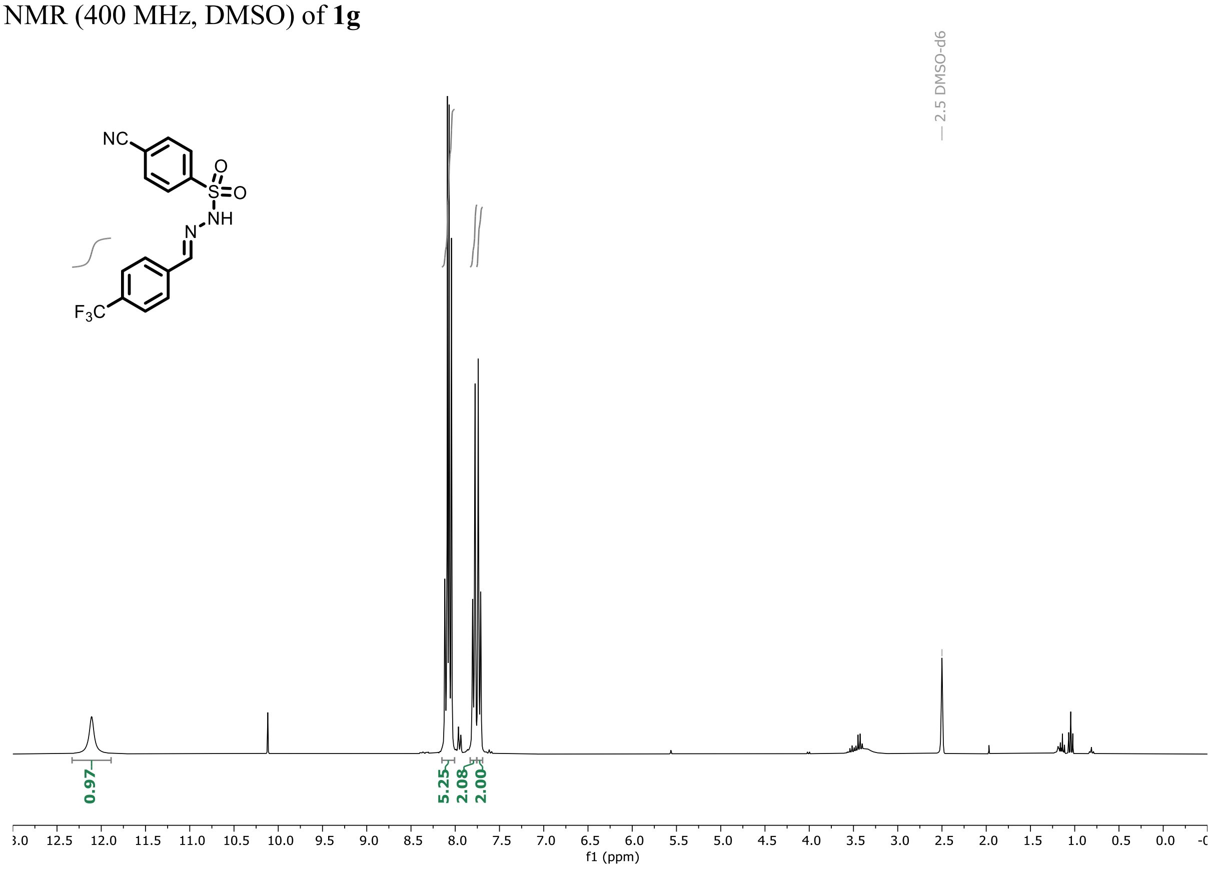

^{13}C NMR (101 MHz, DMSO) of **1g**

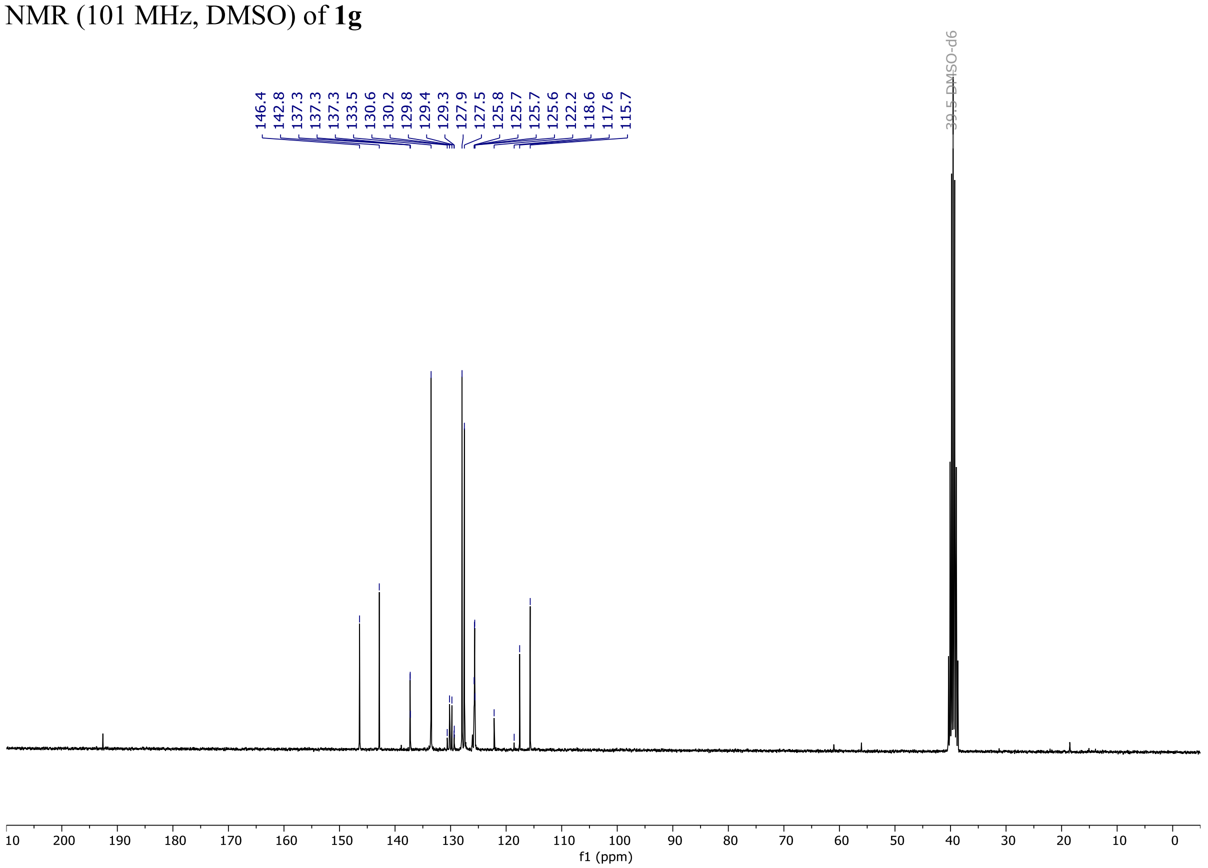

^{19}F NMR (282 MHz, DMSO) of **1g**

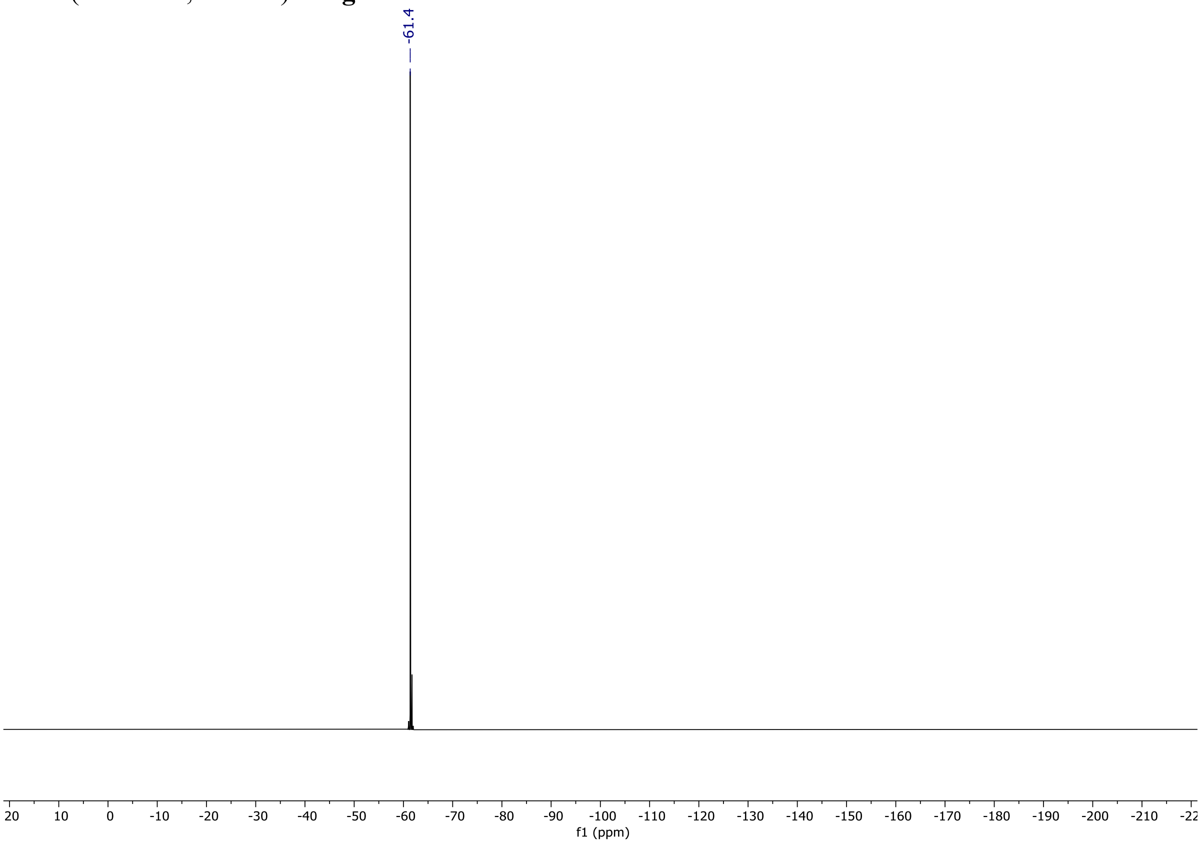

^{1}H NMR (400 MHz, $CDCl_3$) of **1h**

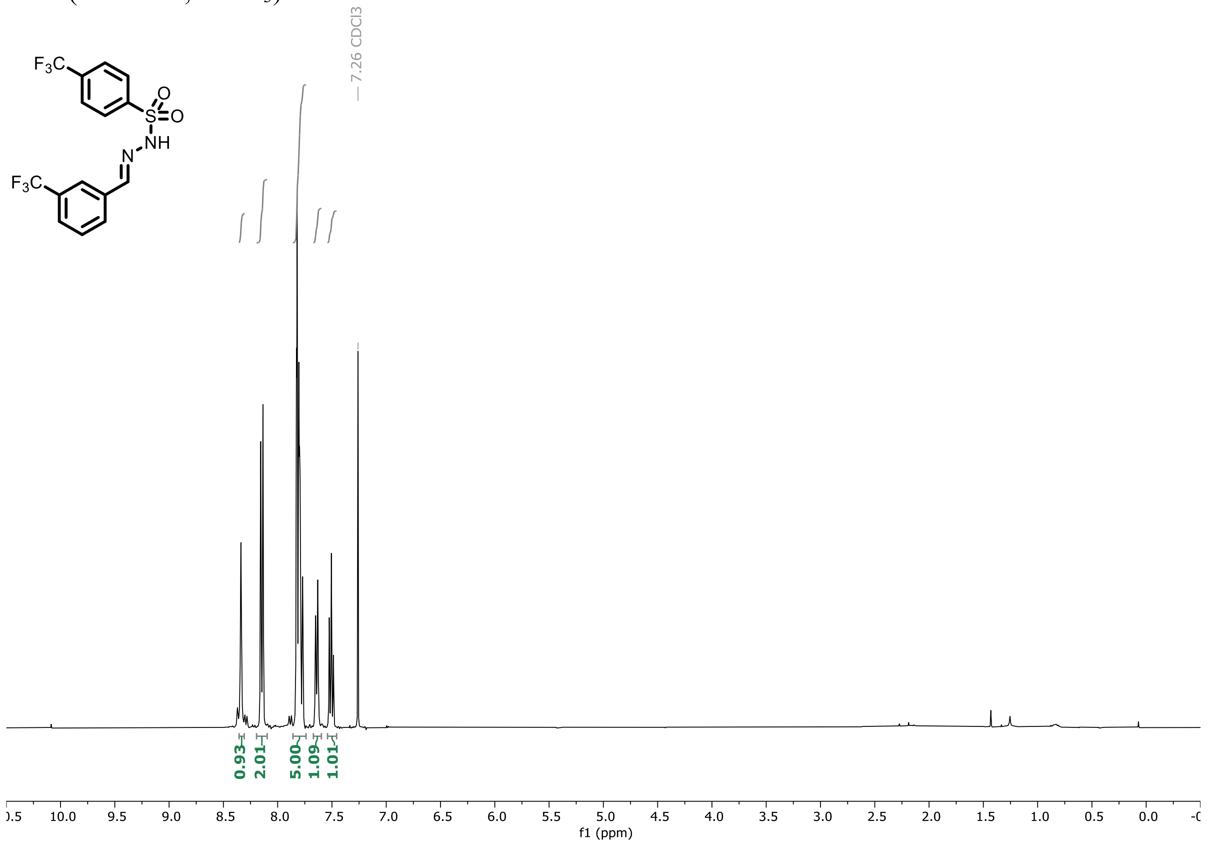

^{13}C NMR (101 MHz, $CDCl_3$) of **1h**

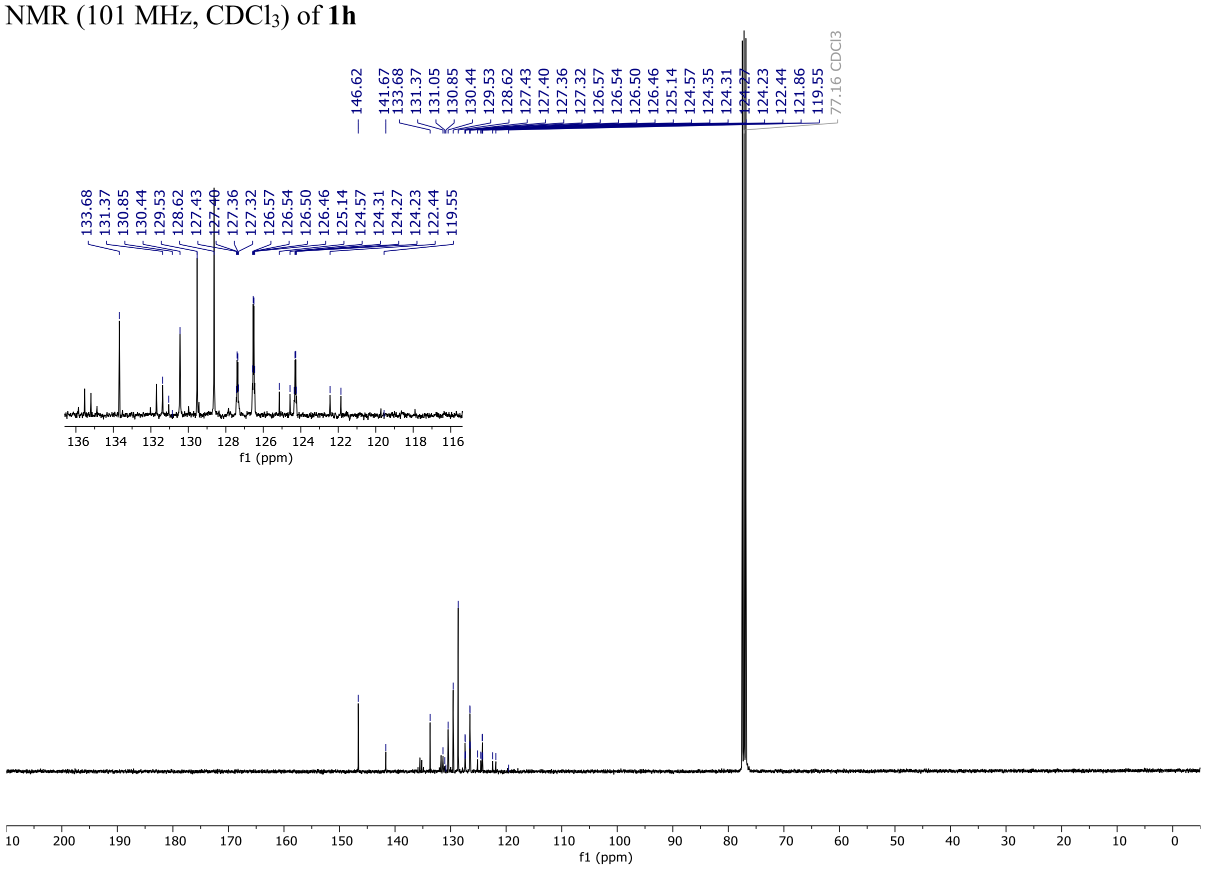

^{19}F NMR (282 MHz, $CDCl_3$) of **1h**

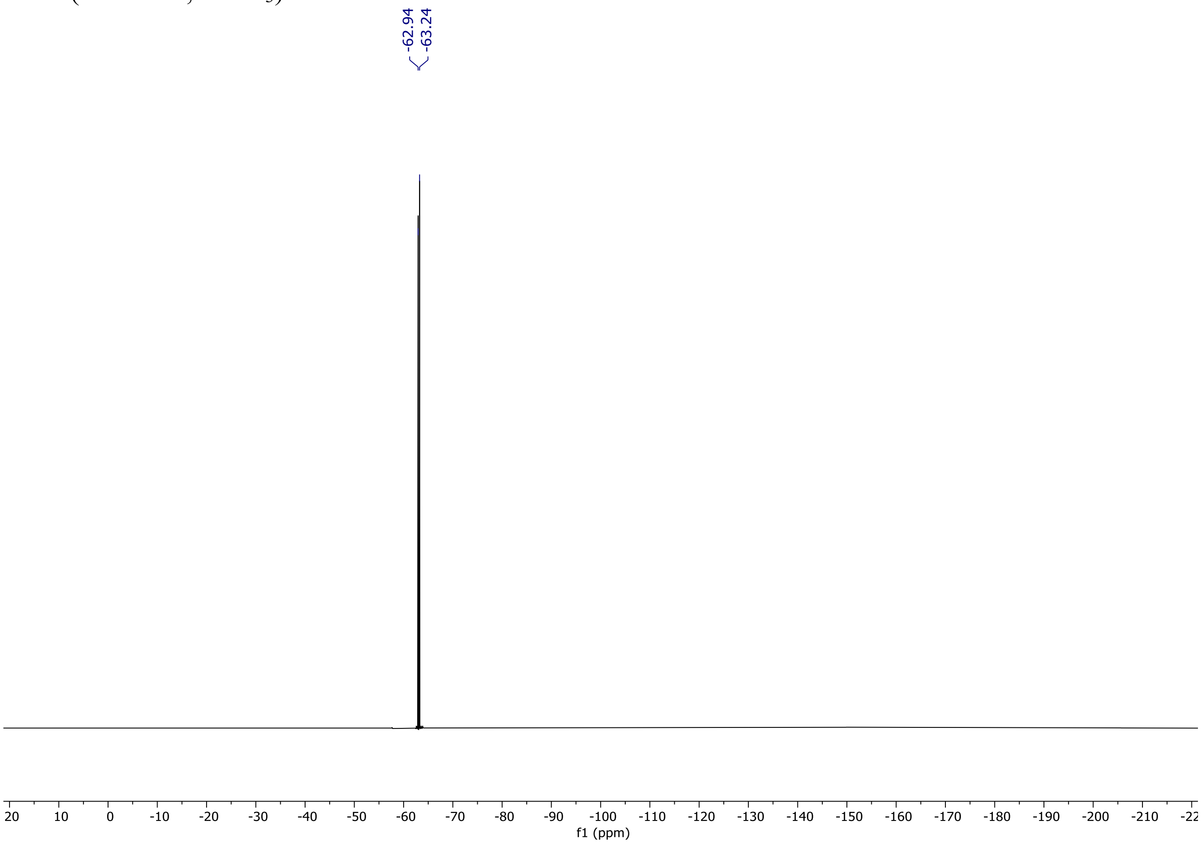

^{1}H NMR (400 MHz, DMSO) of **1i**

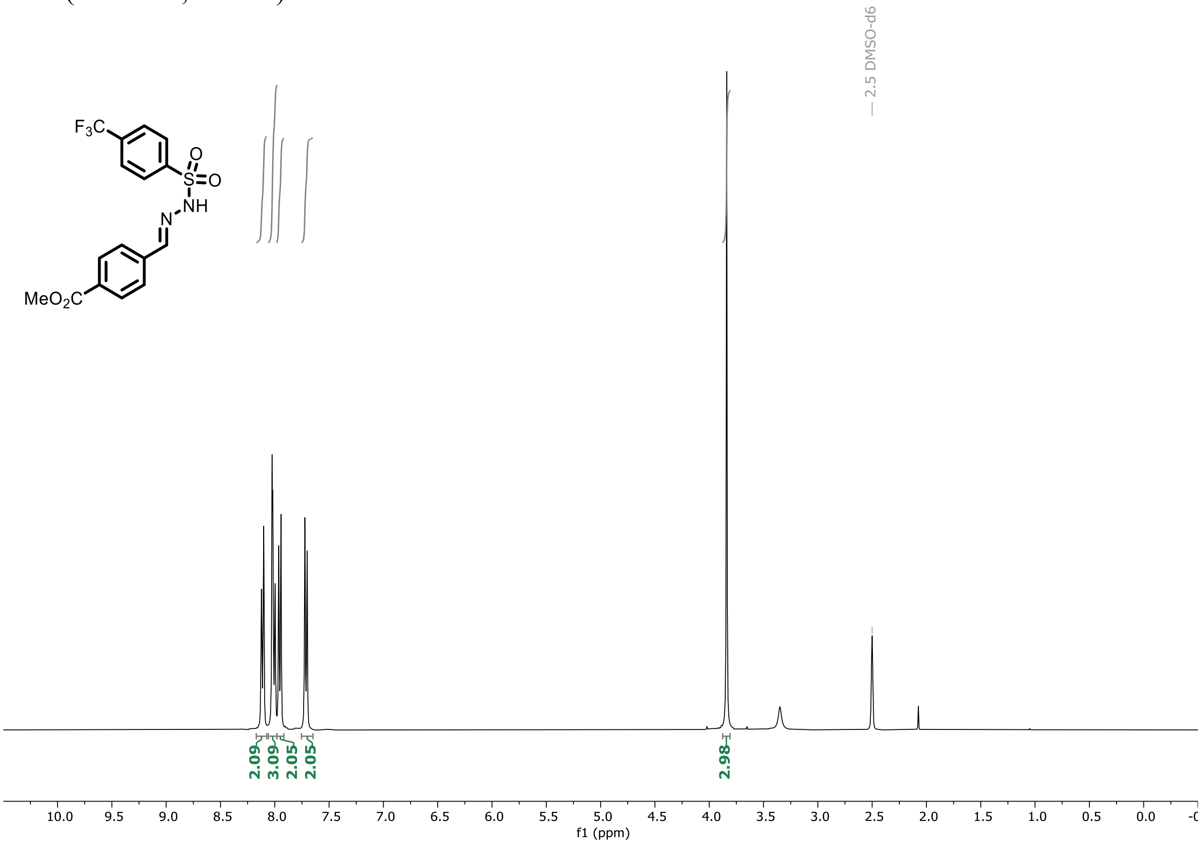

^{13}C NMR (101 MHz, DMSO) of **1i**

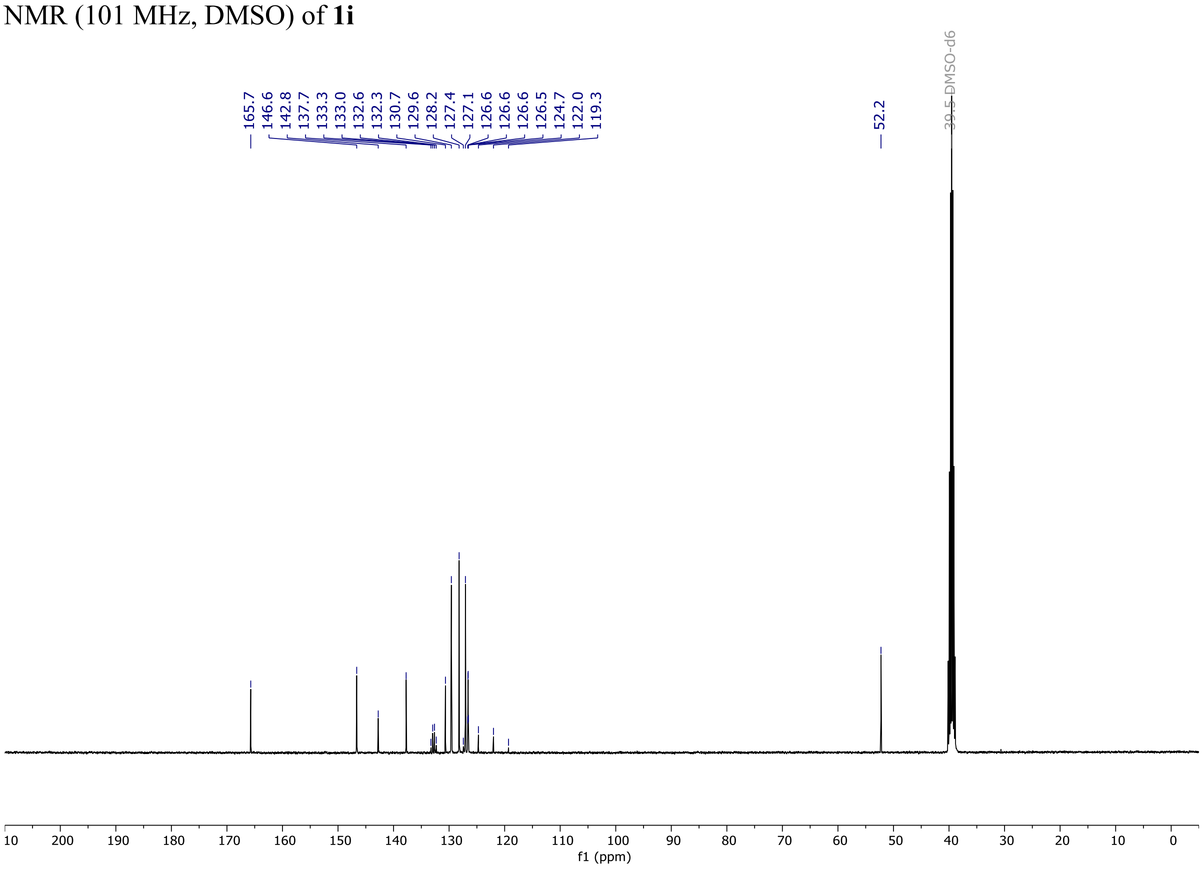

^{19}F NMR (282 MHz, DMSO) of **1i**

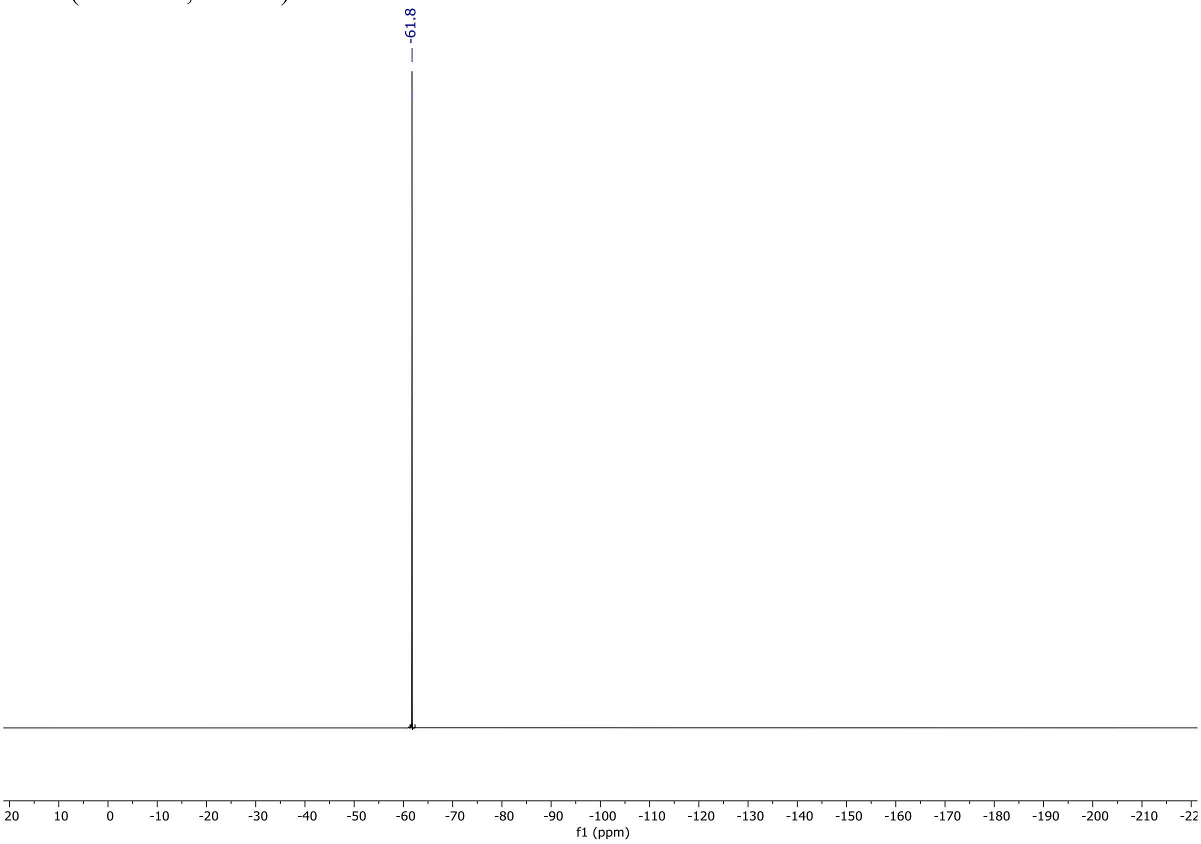

^{1}H NMR (400 MHz, DMSO) of **1j**

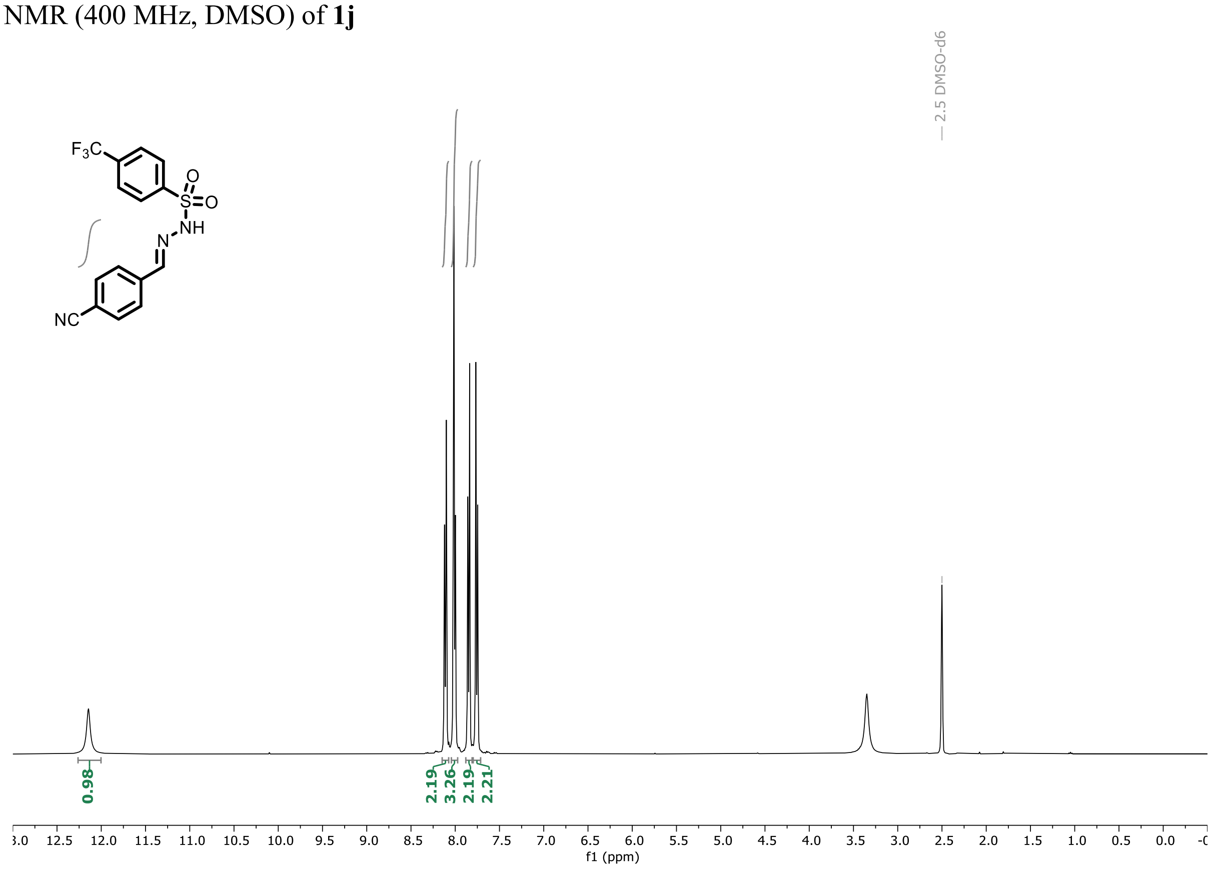

^{13}C NMR (101 MHz, DMSO) of **1j**

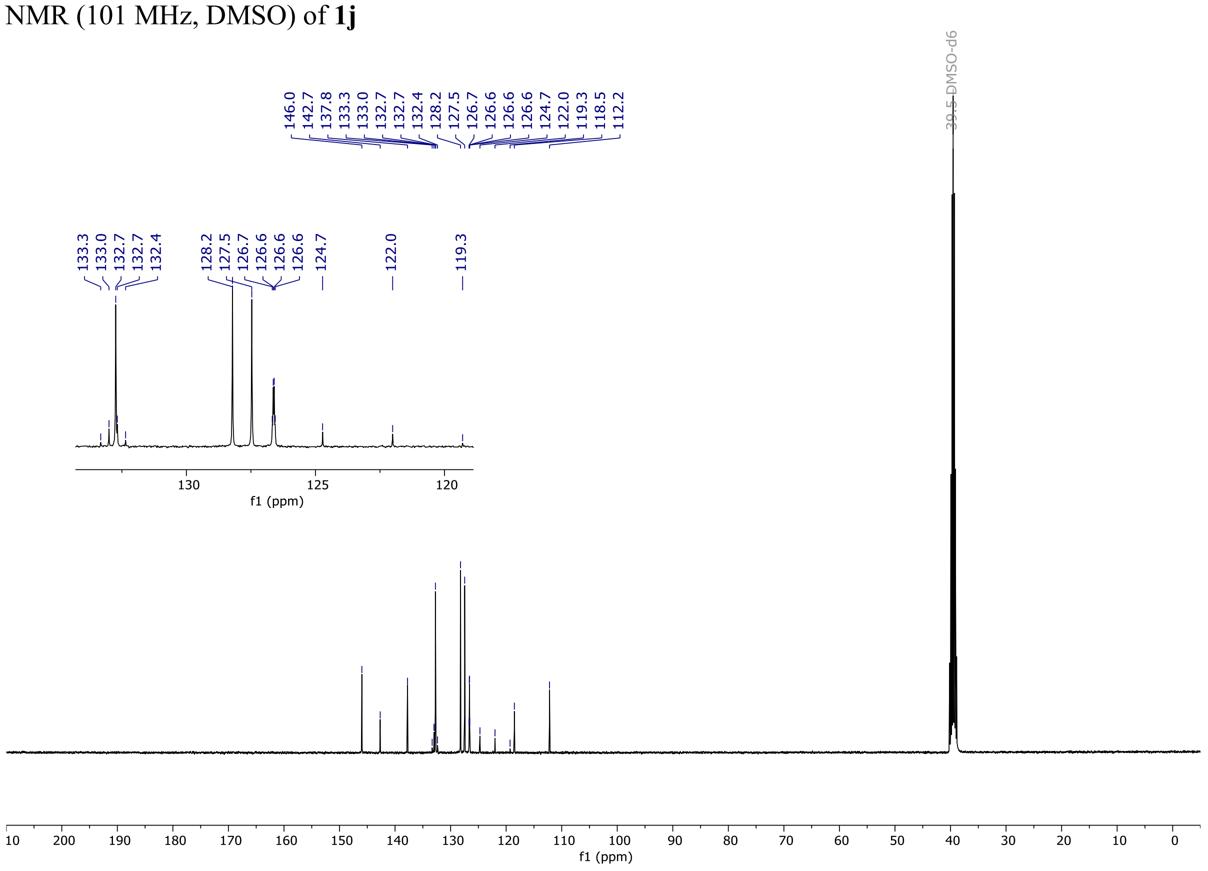

^{19}F NMR (282 MHz, DMSO) of **1j**

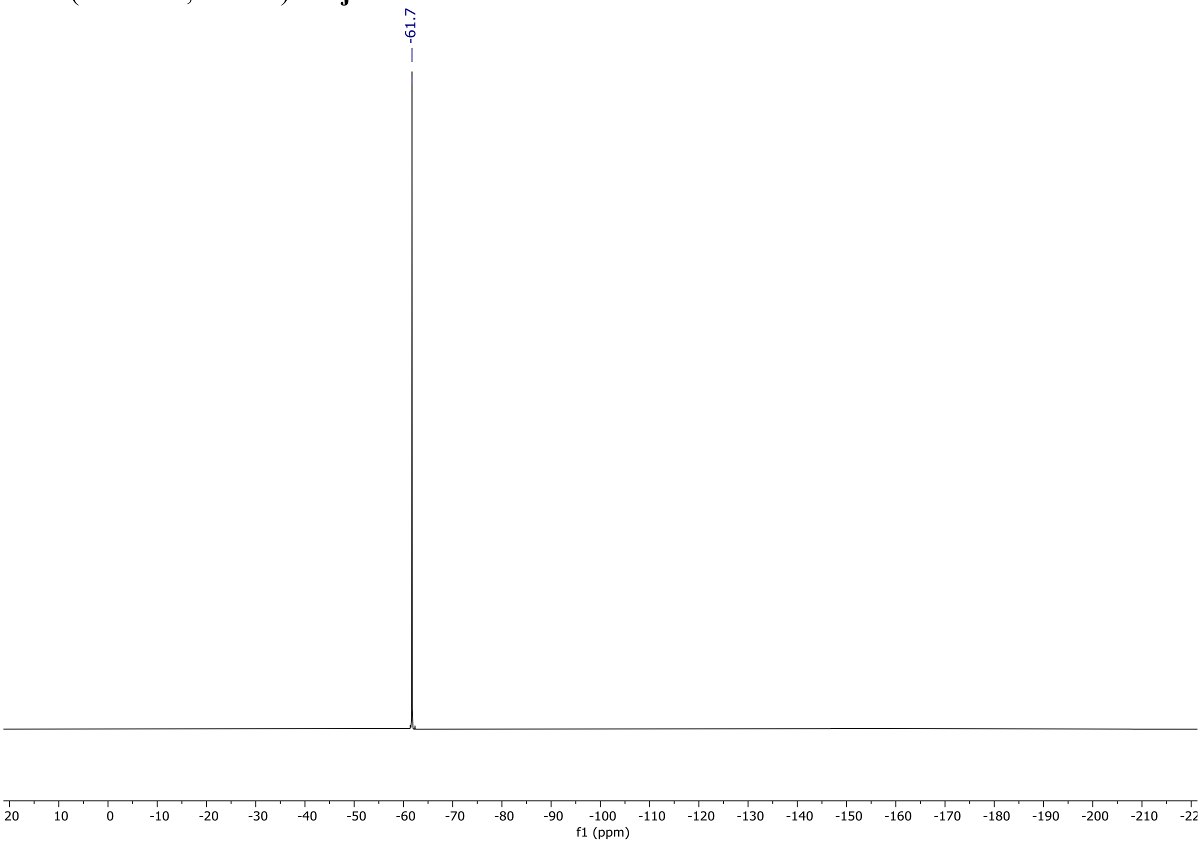

^{1}H NMR (300 MHz,$CDCl_3$) of **1k**

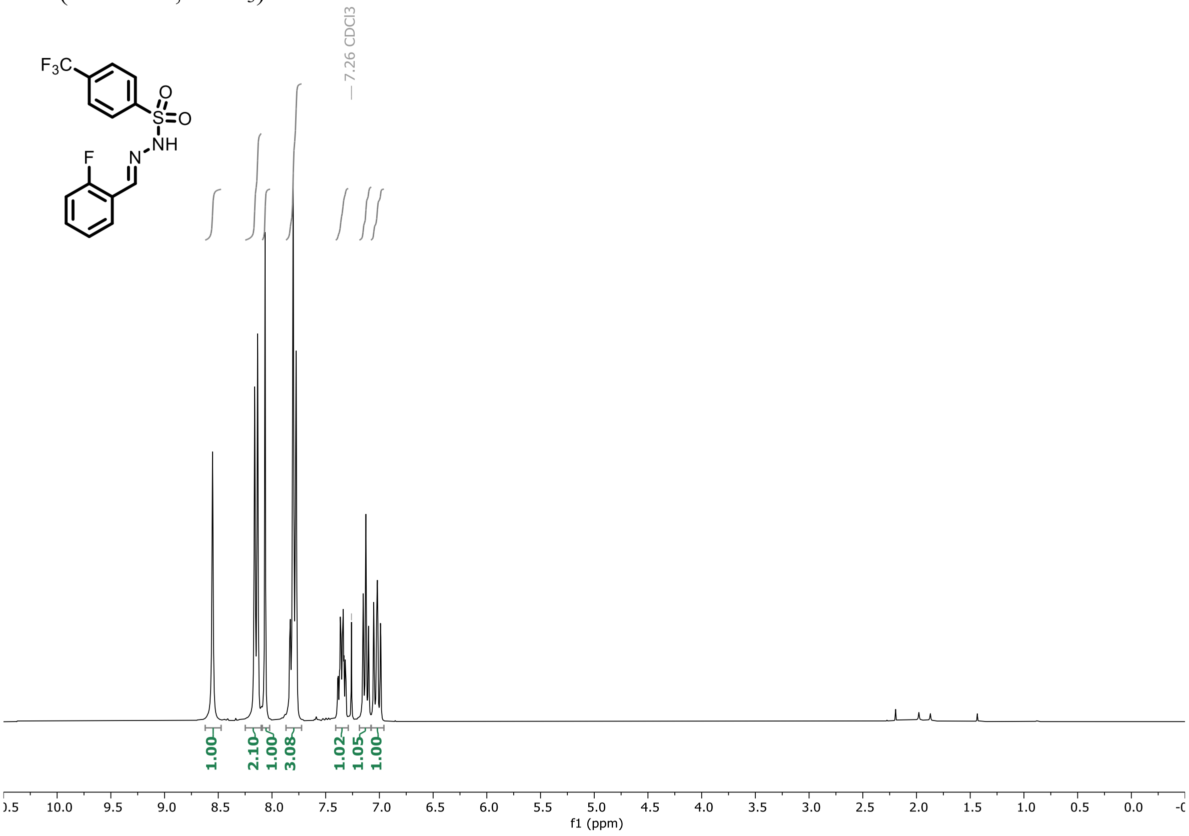

^{13}C NMR (101 MHz, $CDCl_3$) of **1k**

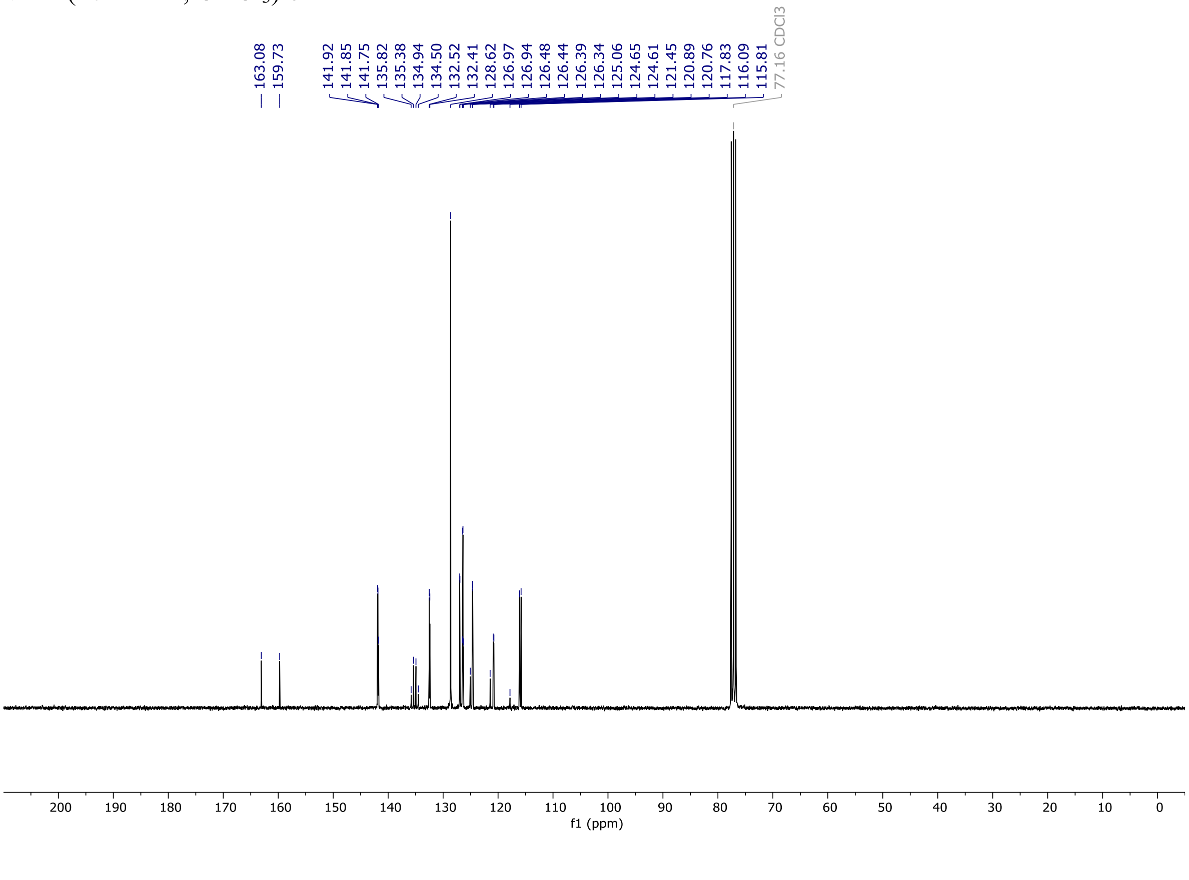

^{19}F NMR (282 MHz, $CDCl_3$) of **1k**

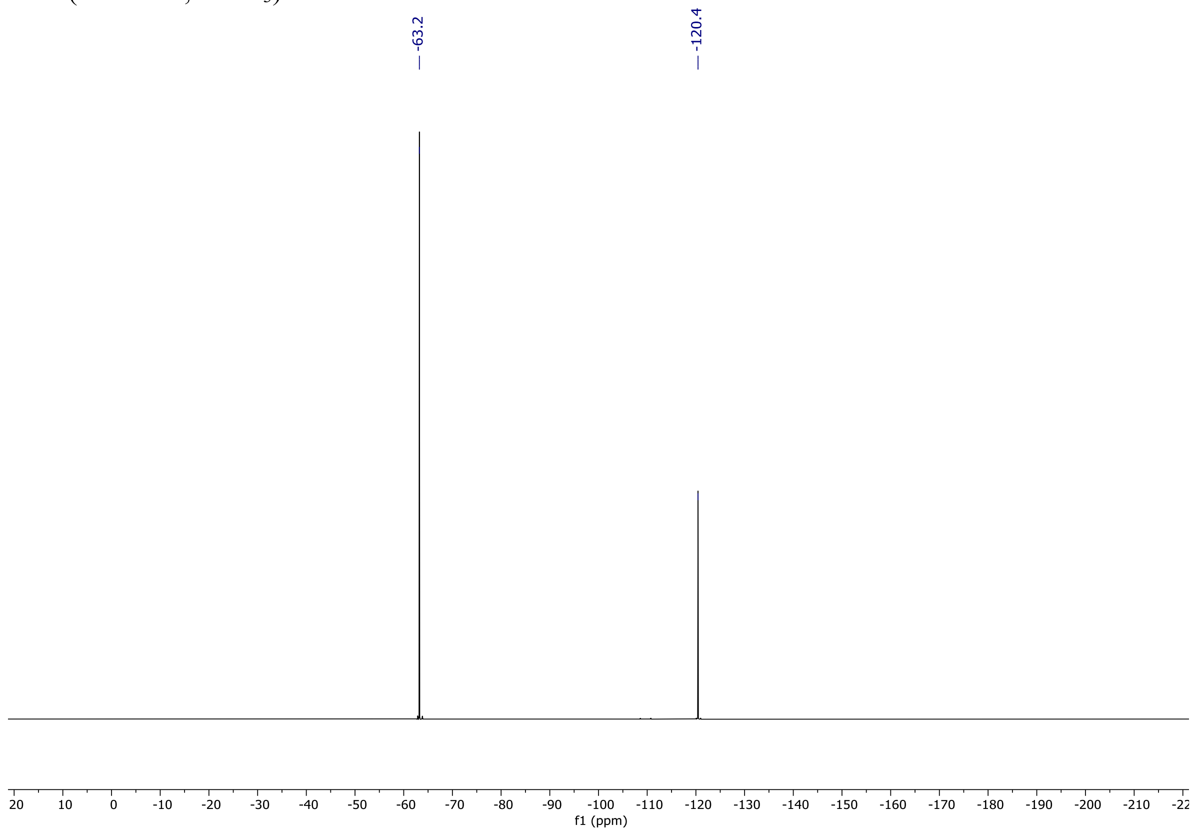

^{1}H NMR (400 MHz, DMSO) of **1l**

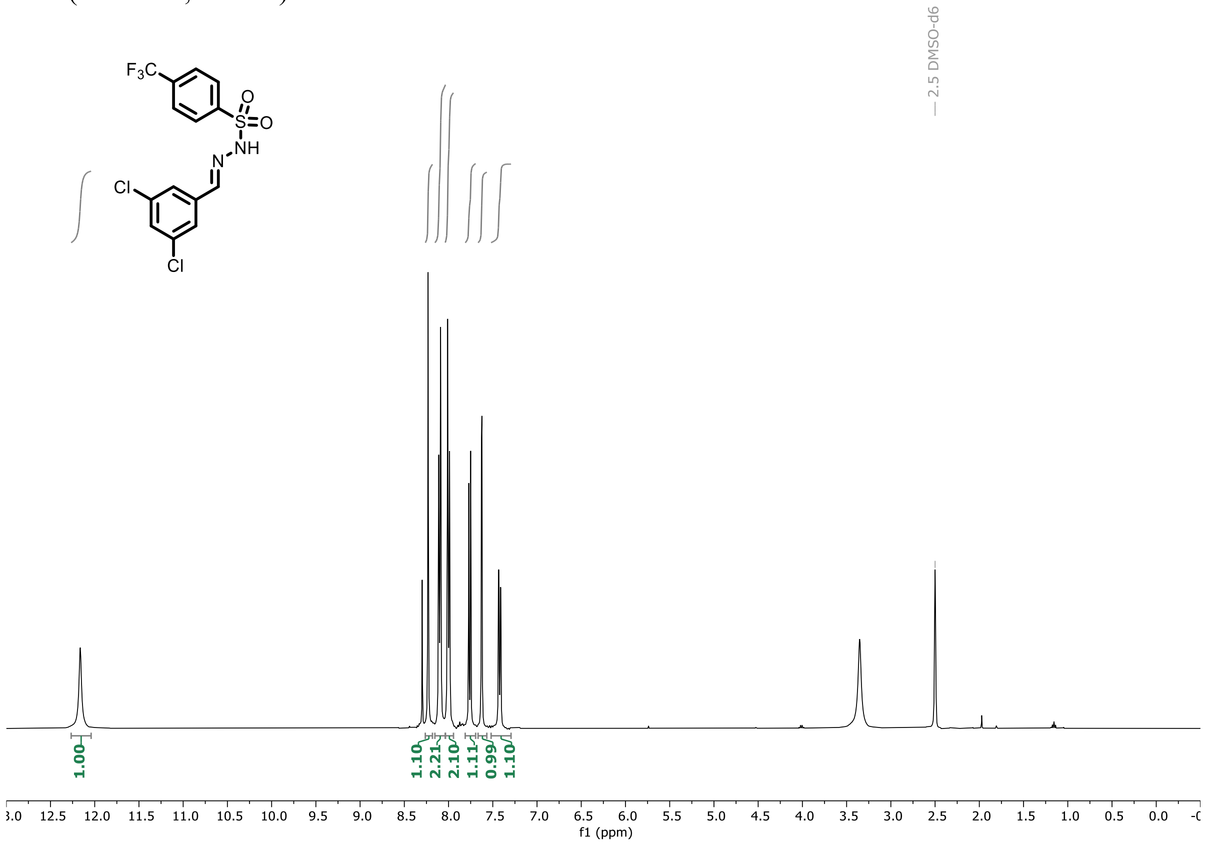

^{13}C NMR (101 MHz, DMSO) of **1l**

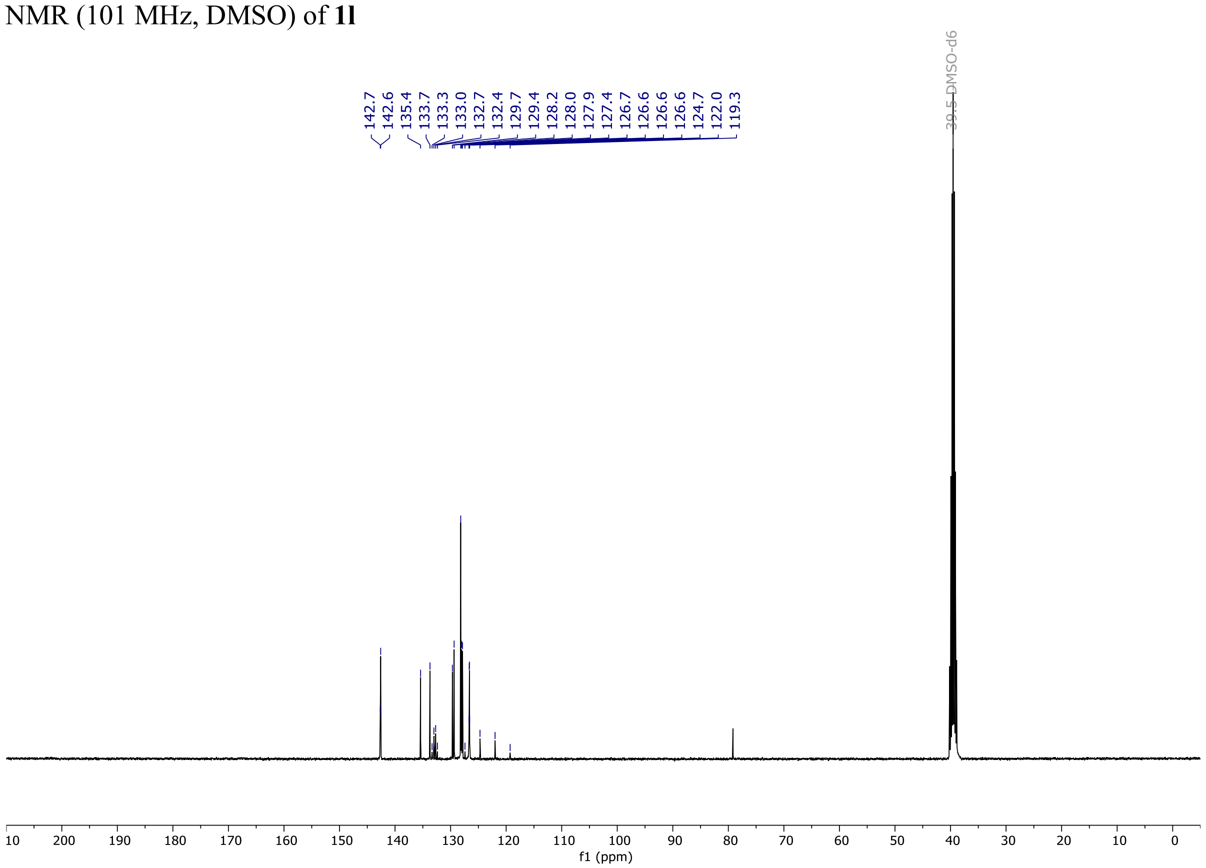

^{19}F NMR (282 MHz, DMSO) of **1l**

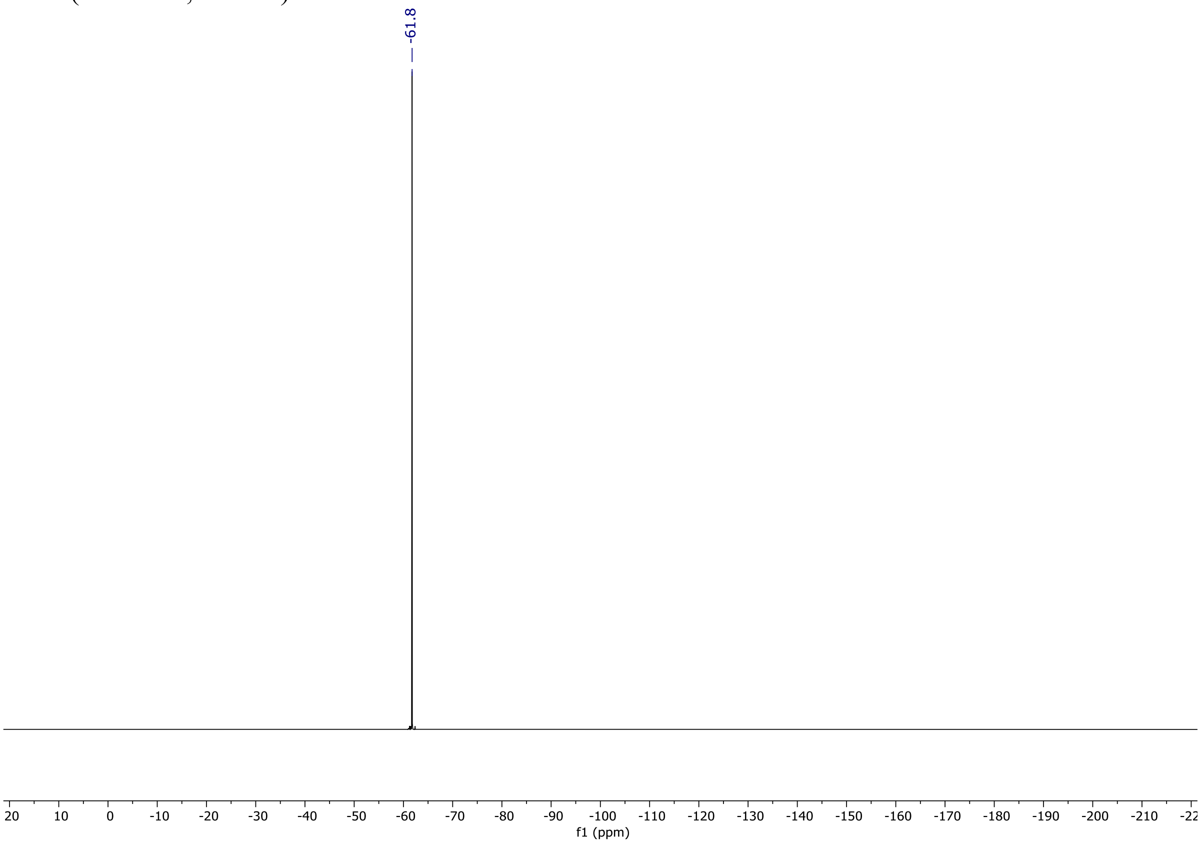

^{1}H NMR (400 MHz, DMSO) of **1m**

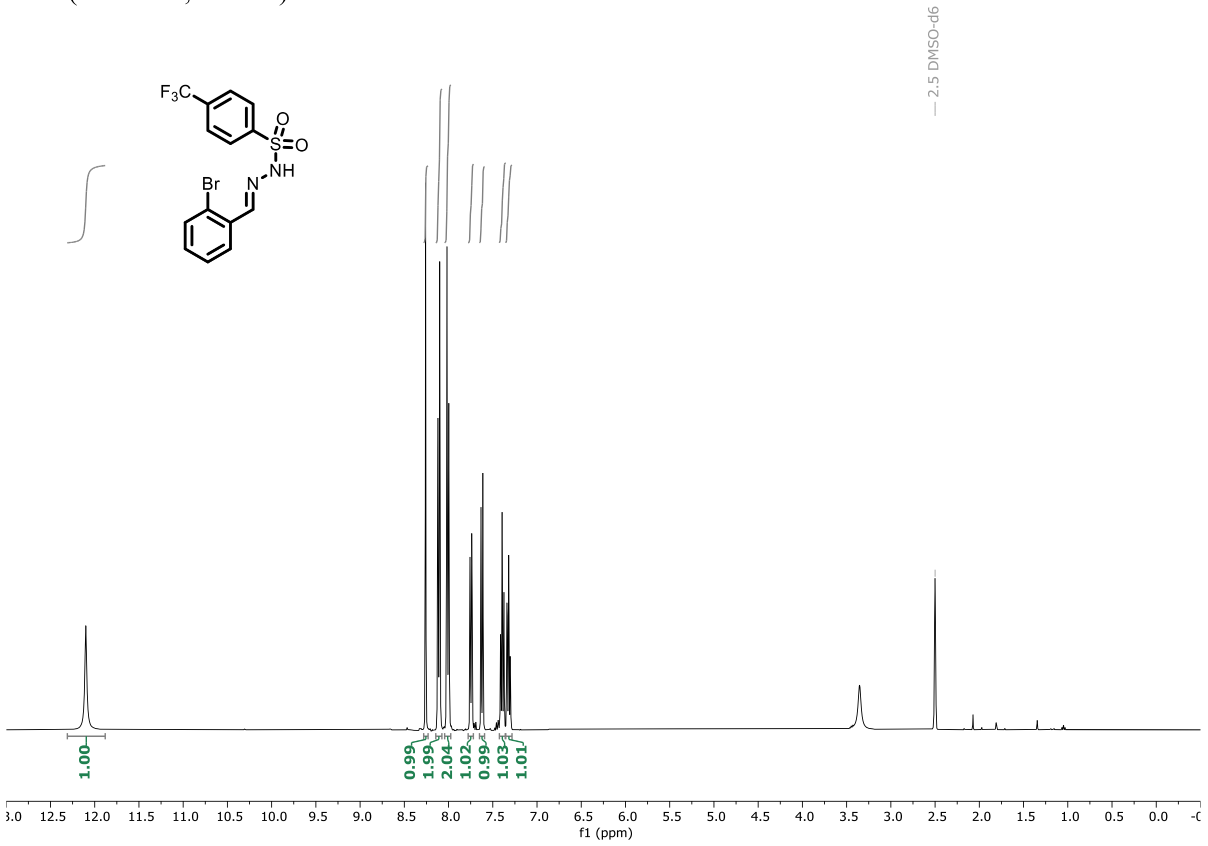

^{13}C NMR (101 MHz, DMSO) of **1m**

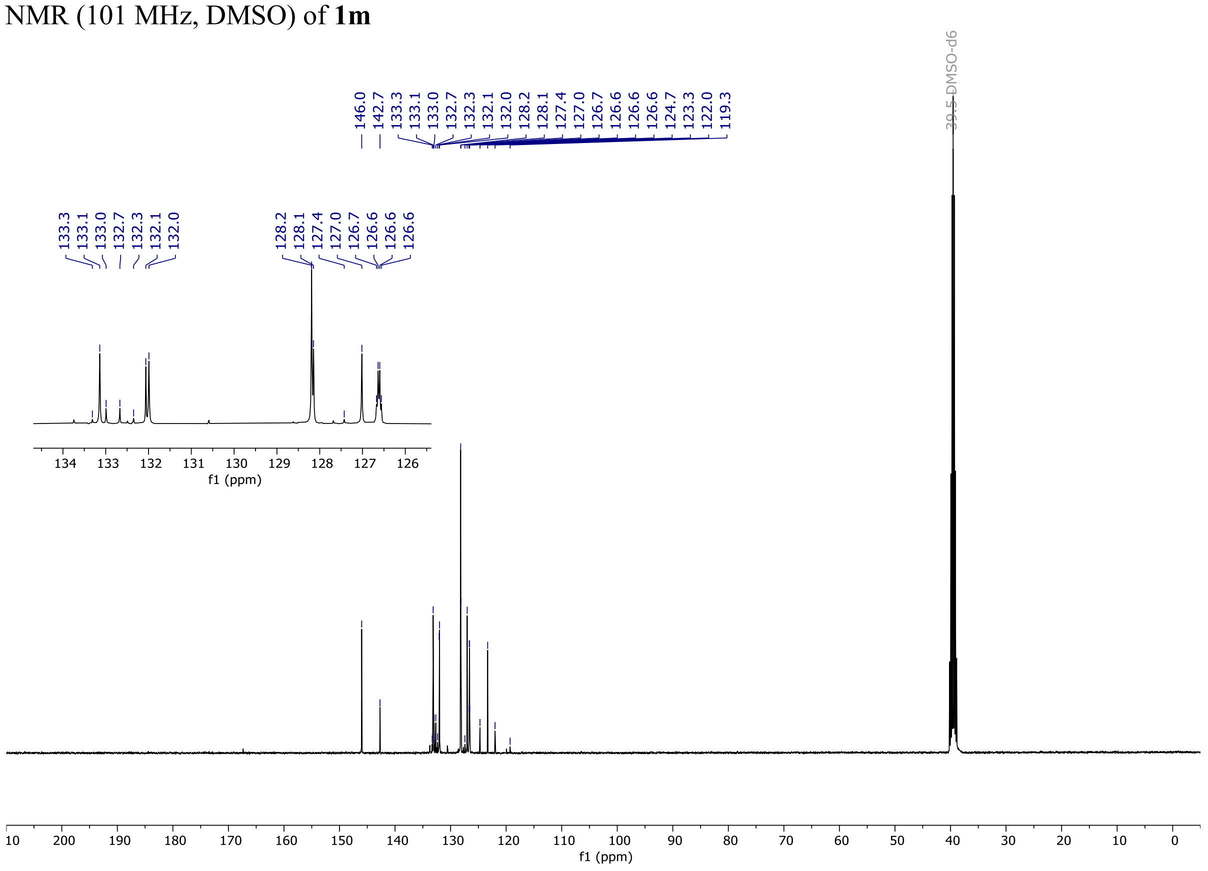

^{19}F NMR (282 MHz, DMSO) of **1m**

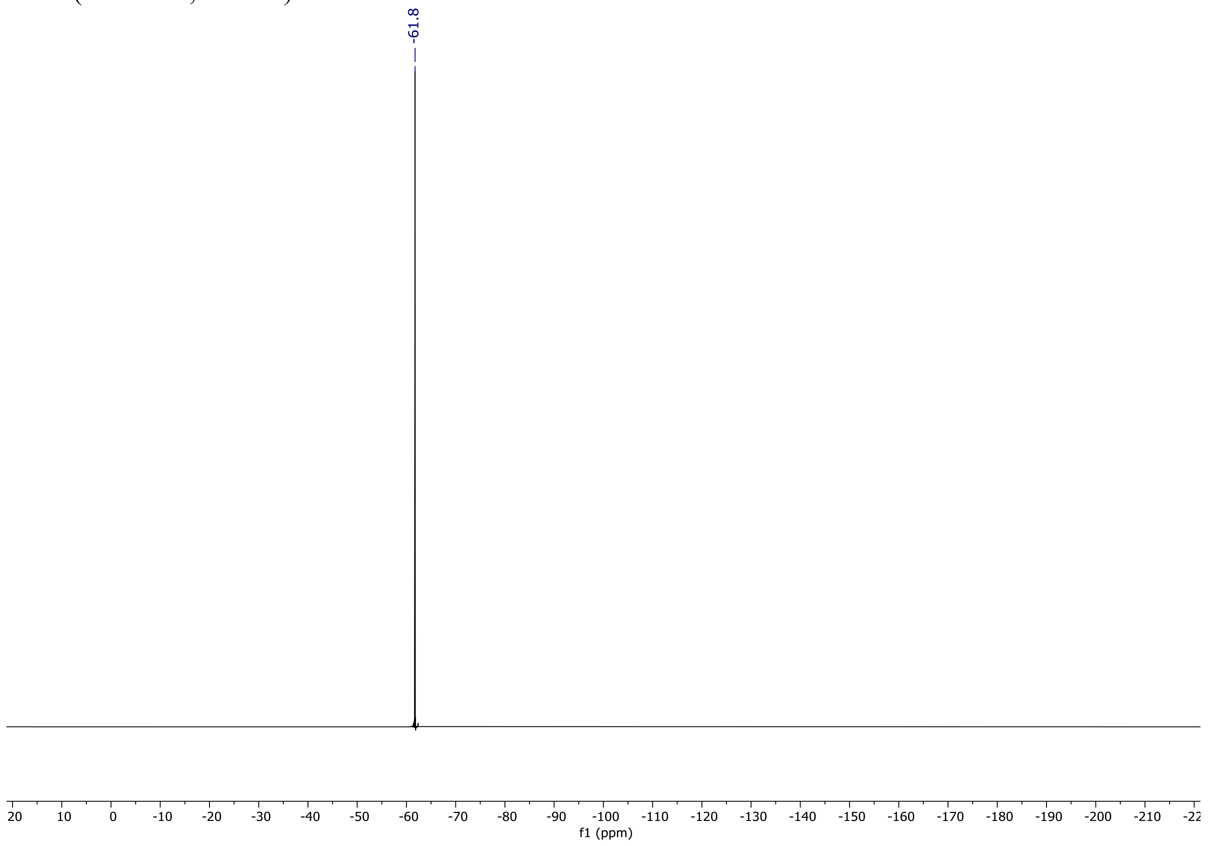

^{1}H NMR (400 MHz, DMSO) of **1n**

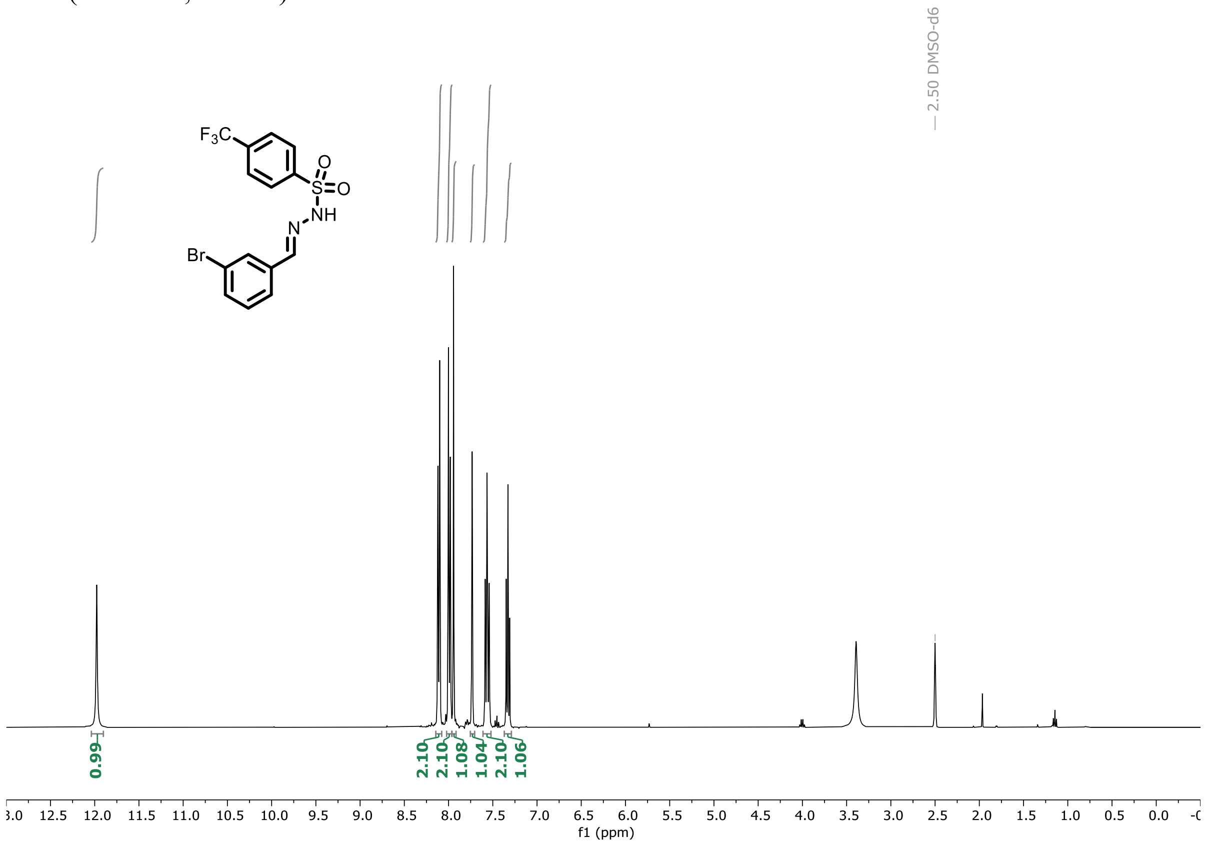

^{13}C NMR (101 MHz, DMSO) of **1n**

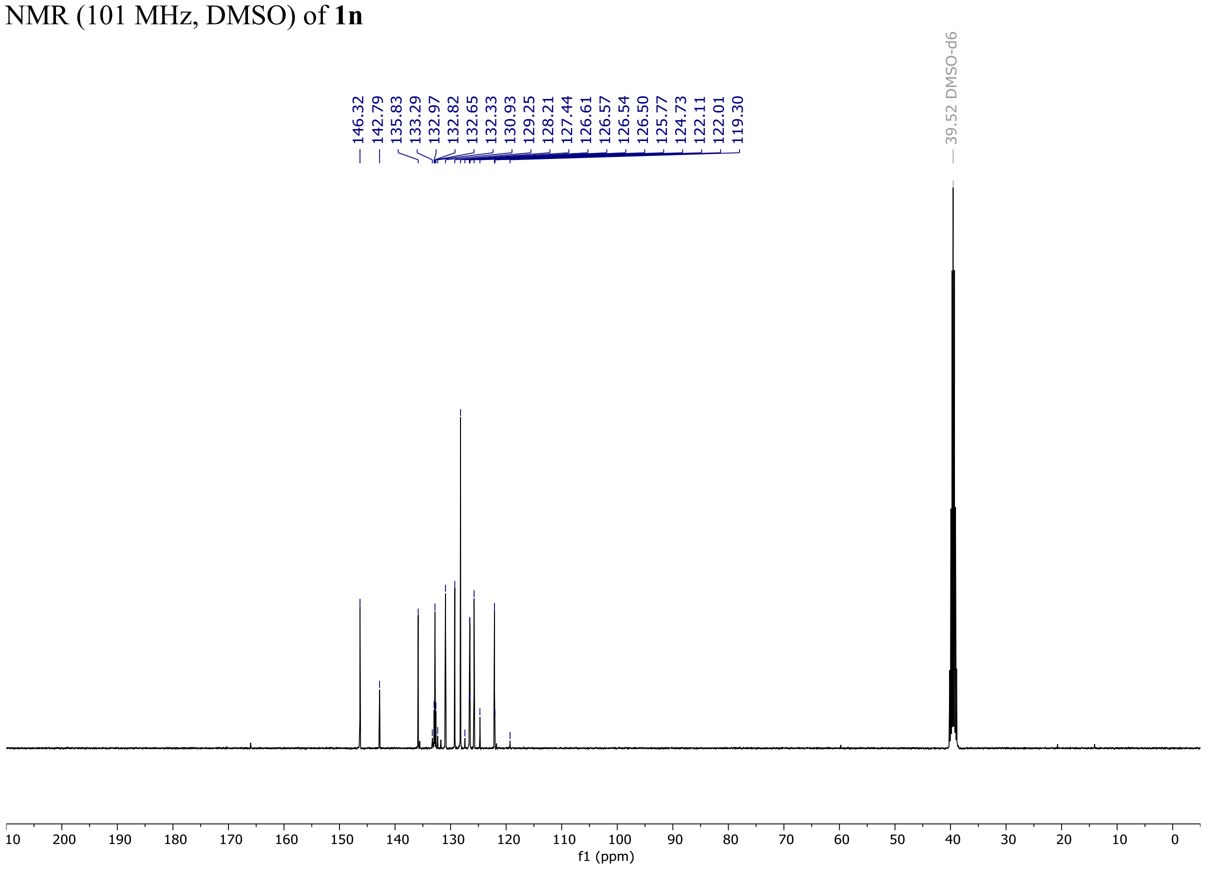

^{19}F NMR (282 MHz, DMSO) of **1n**

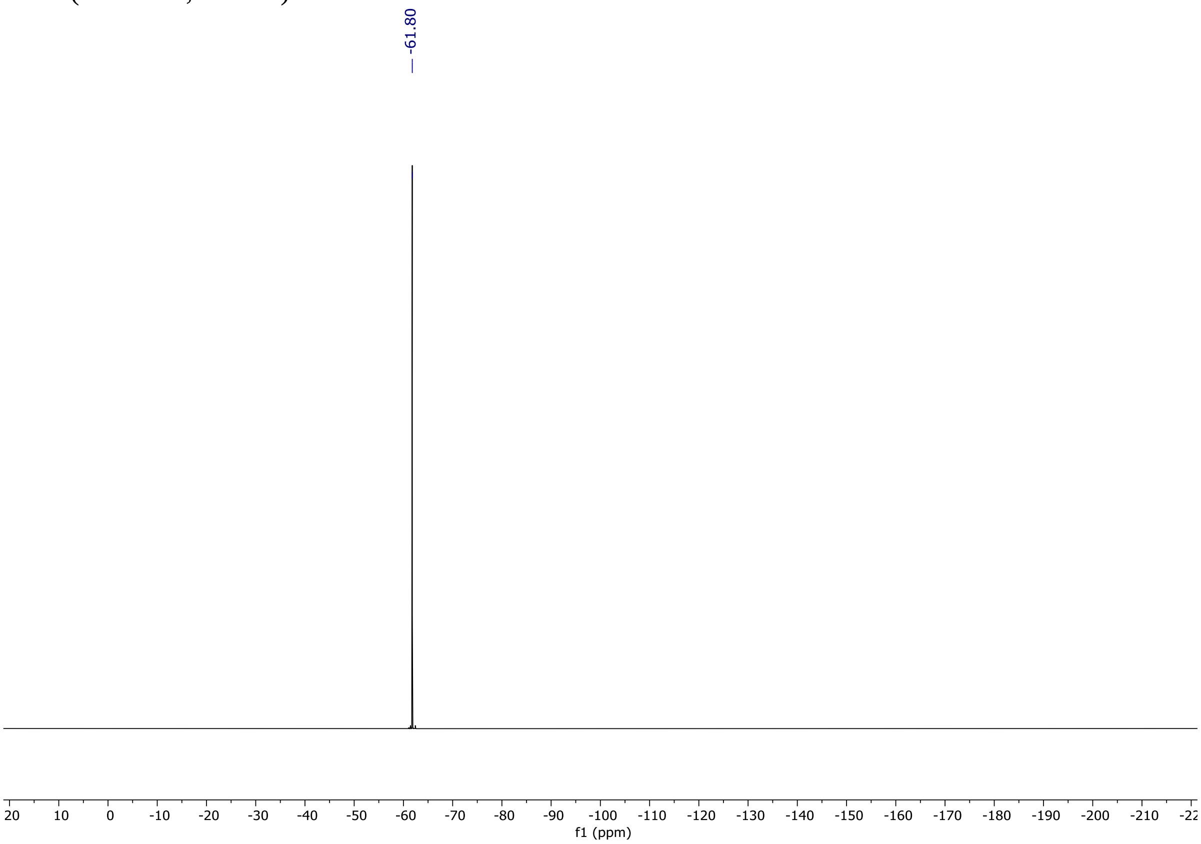

^{1}H NMR (400 MHz, DMSO) of **1o**

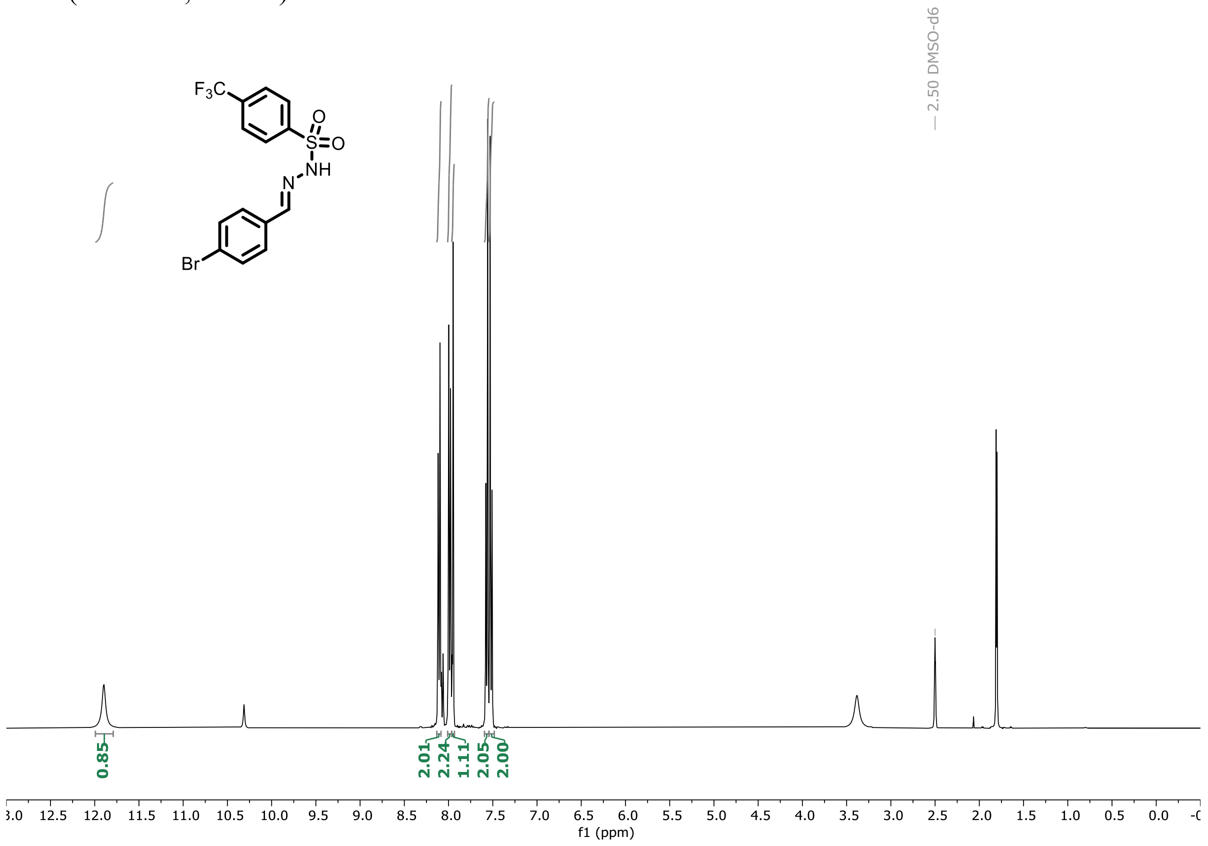

^{13}C NMR (101 MHz, DMSO) of **1o**

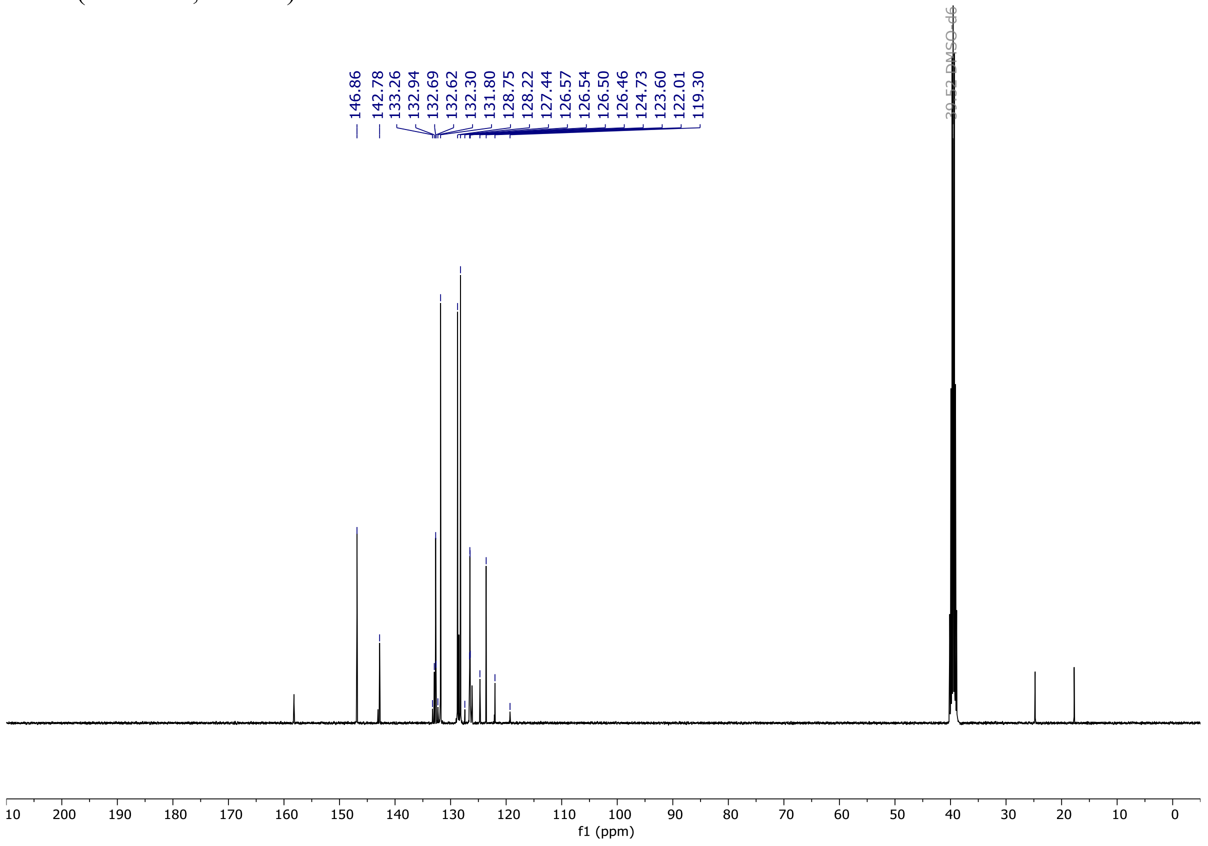

^{19}F NMR (282 MHz, DMSO) of **1o**

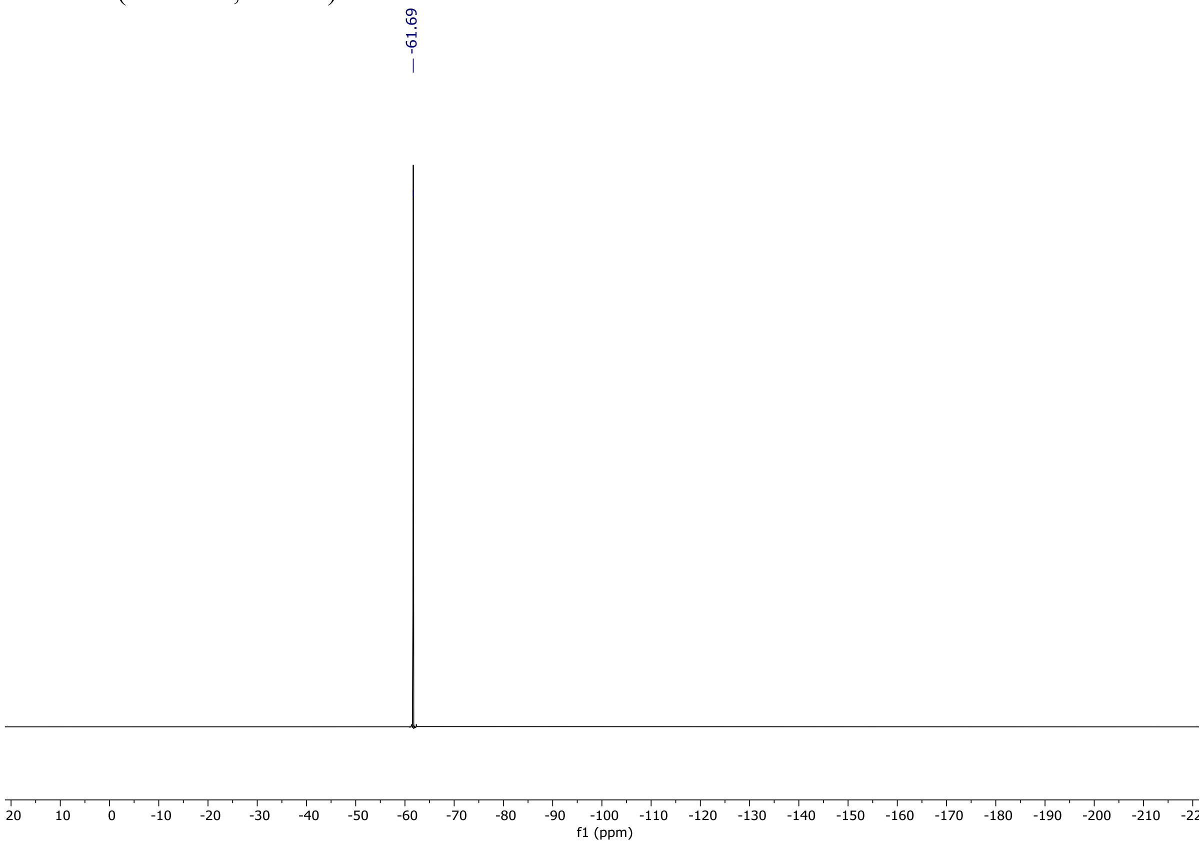

^{1}H NMR (400 MHz,CDCl$_3$) of **1p**

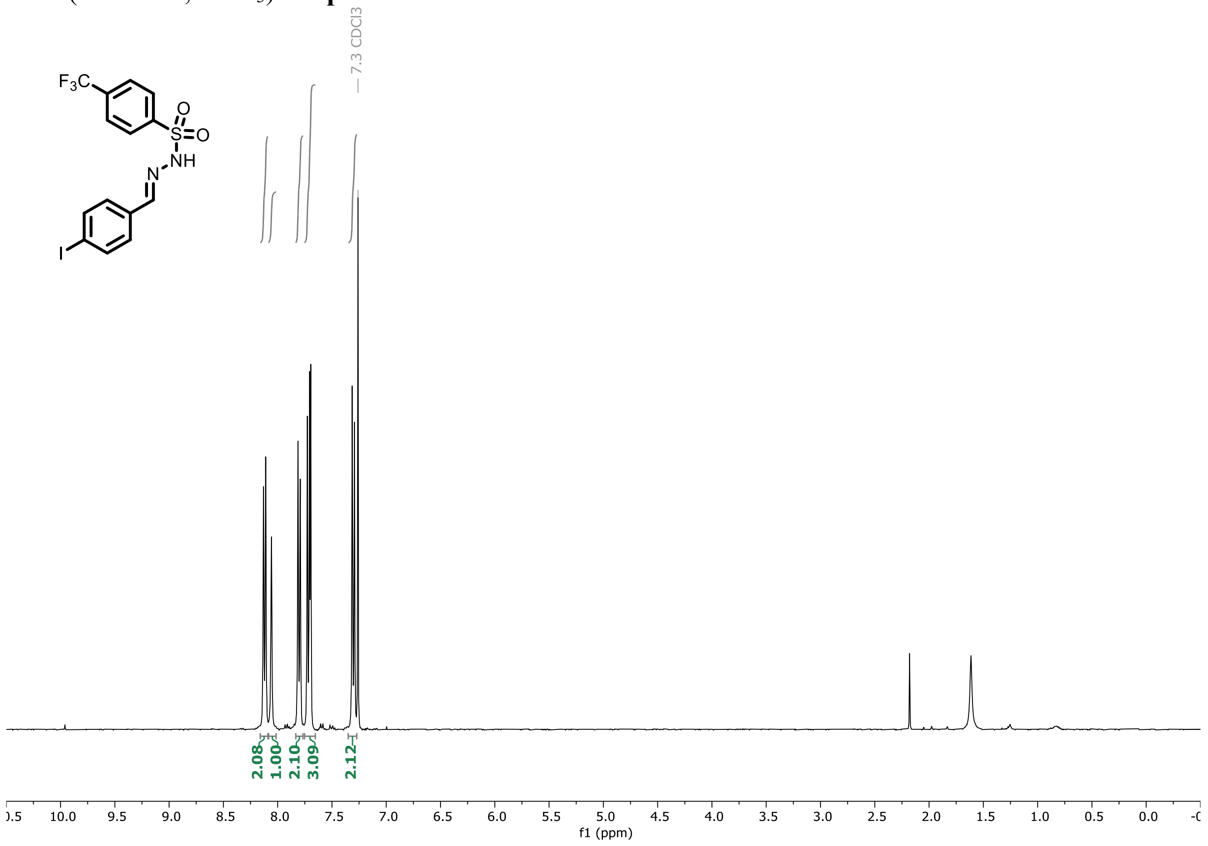

^{13}C NMR (101 MHz, CDCl$_3$) of **1p**

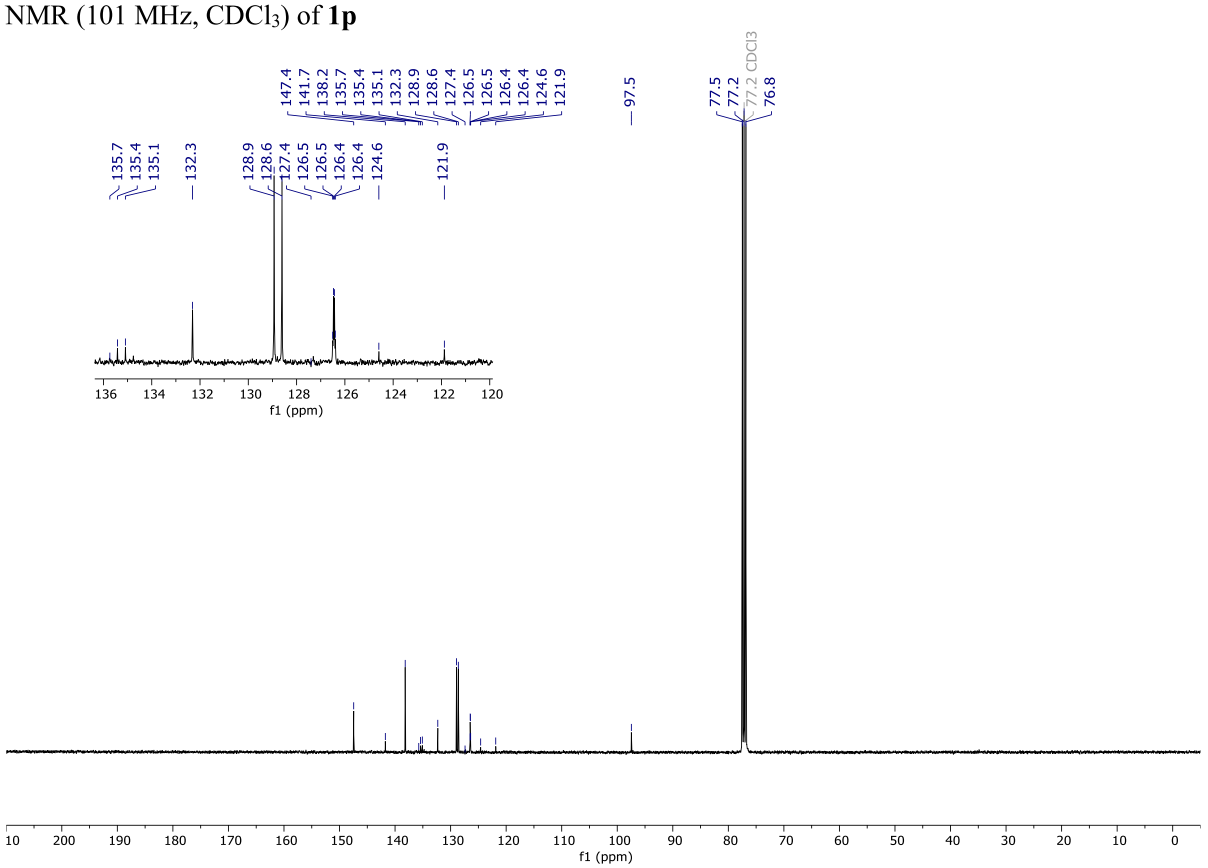

^{19}F NMR (282 MHz, $CDCl_3$) of **1p**

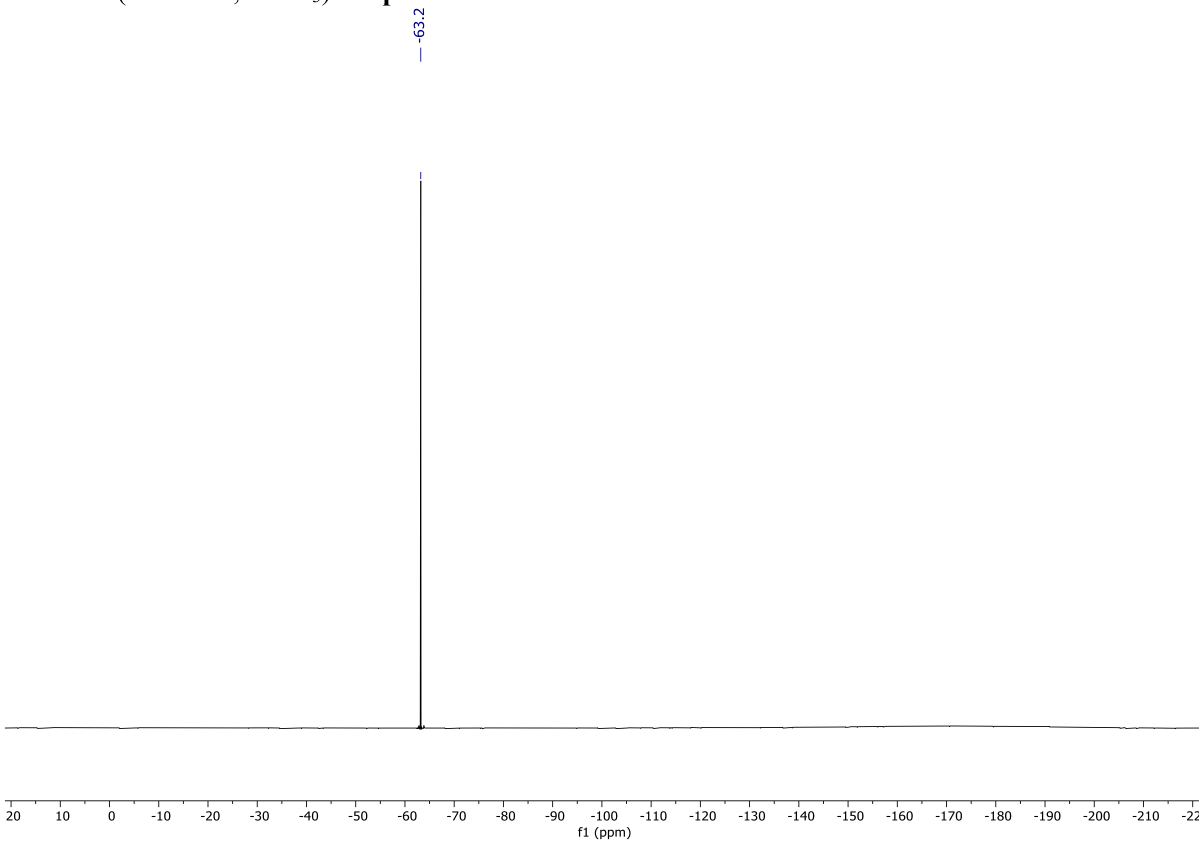

^{1}H NMR (400 MHz, DMSO) of **1q**

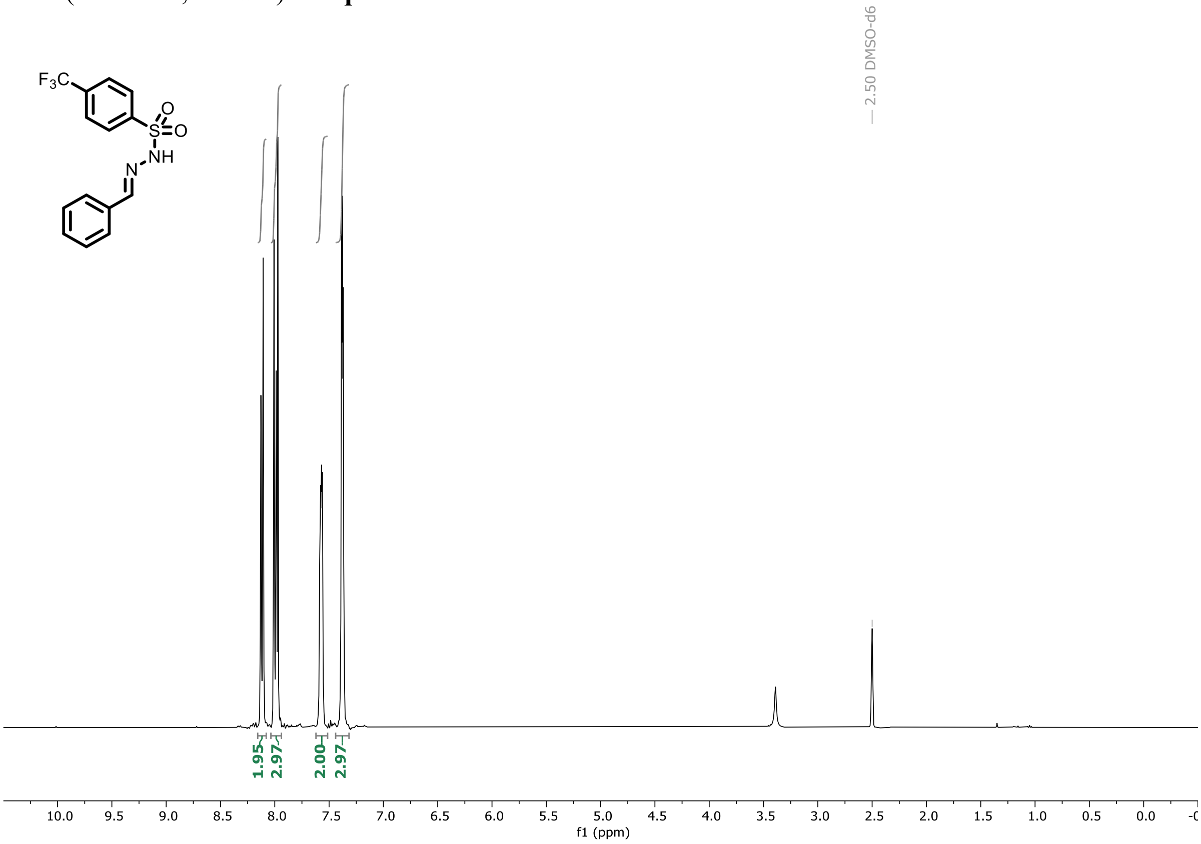

^{13}C NMR (101 MHz, DMSO) of **1q**

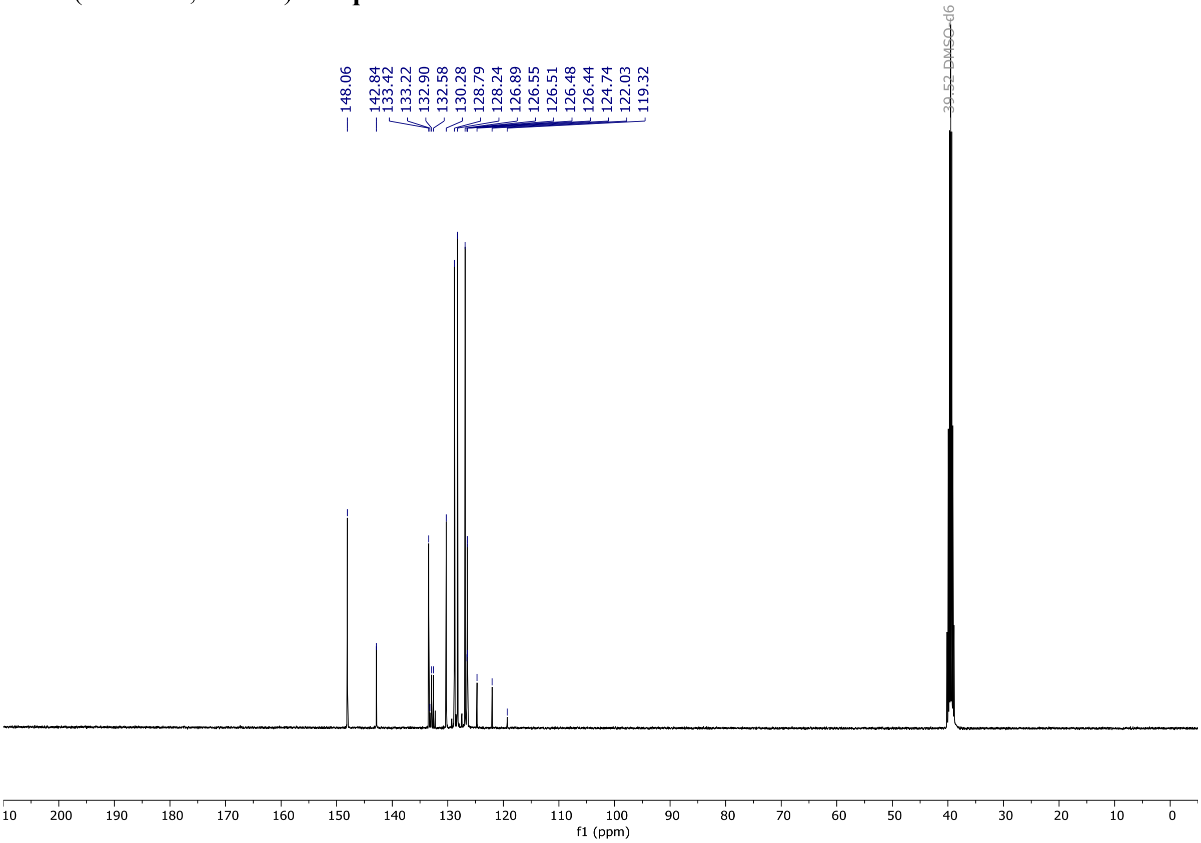

^{19}F NMR (282 MHz, DMSO) of **1q**

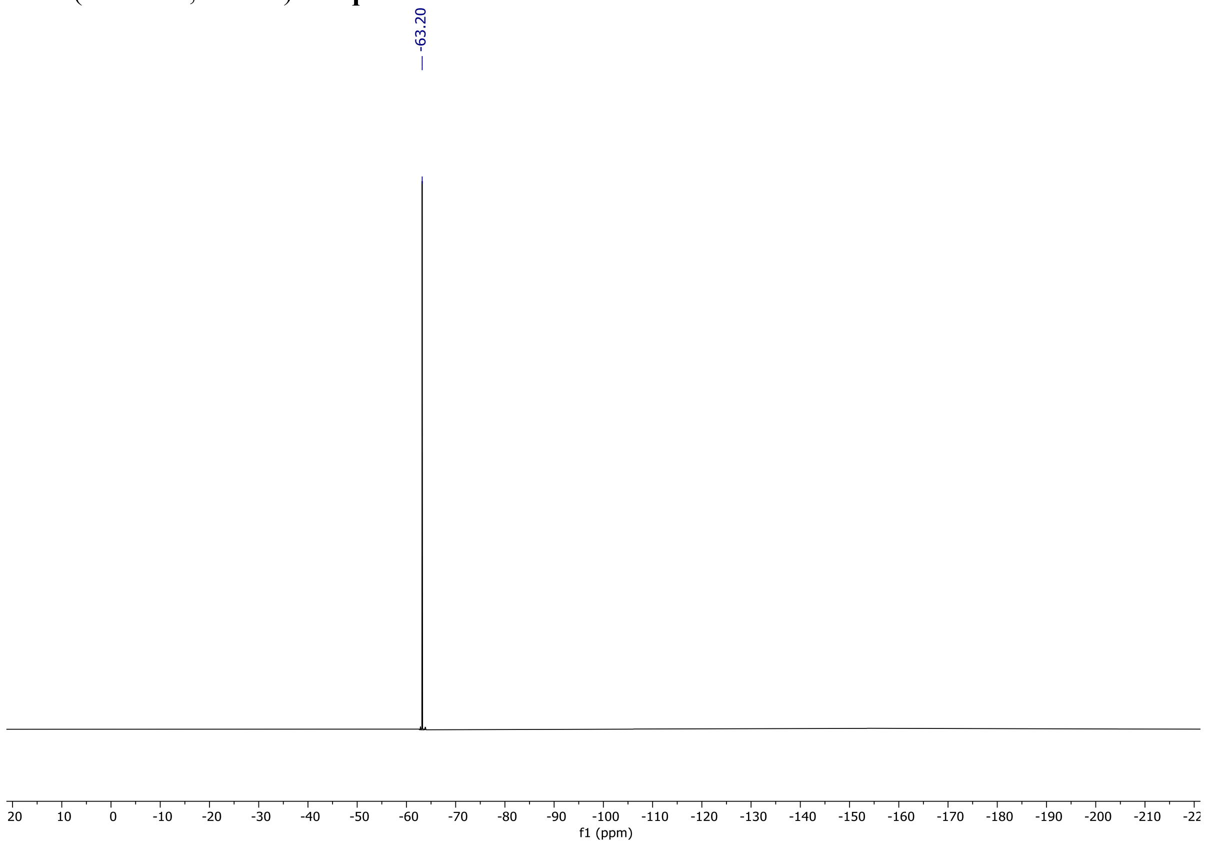

^{1}H NMR (400 MHz, DMSO) of **1r**

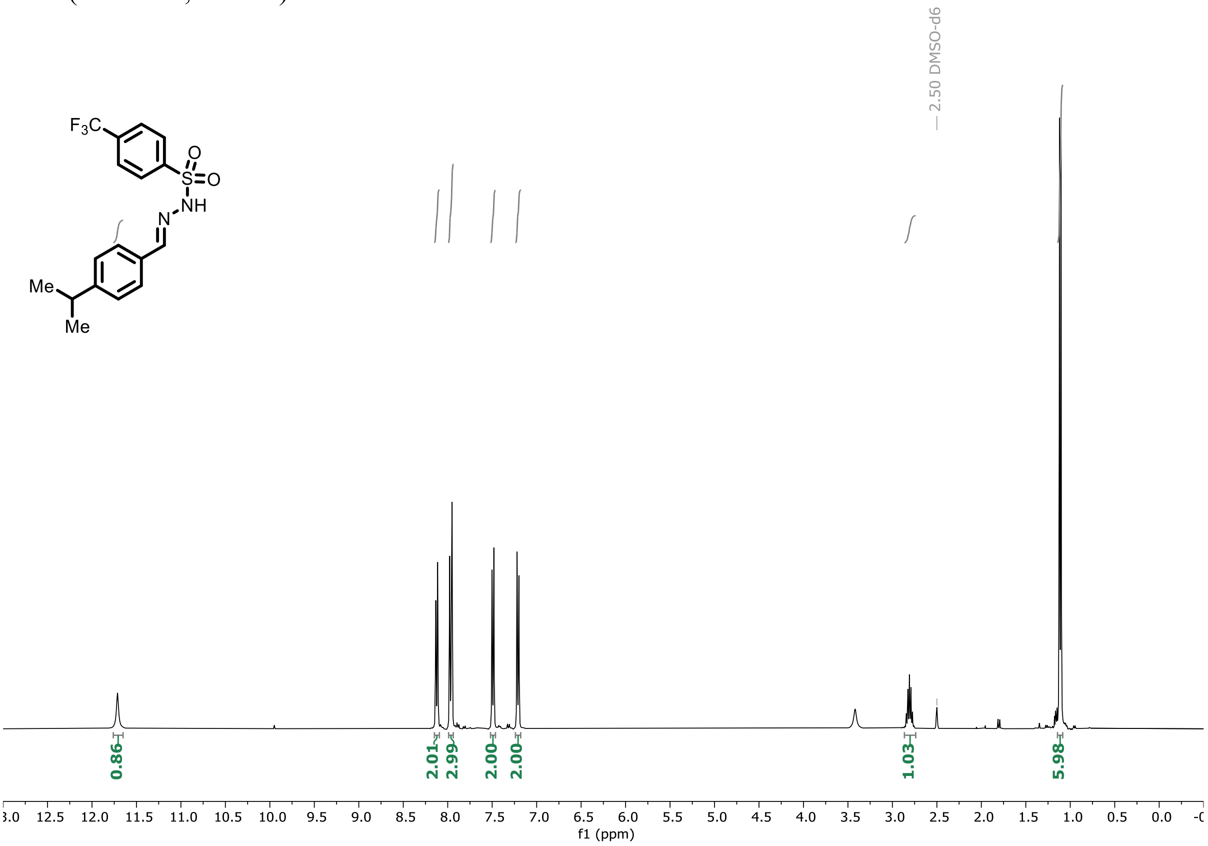

^{13}C NMR (101 MHz, DMSO) of **1r**

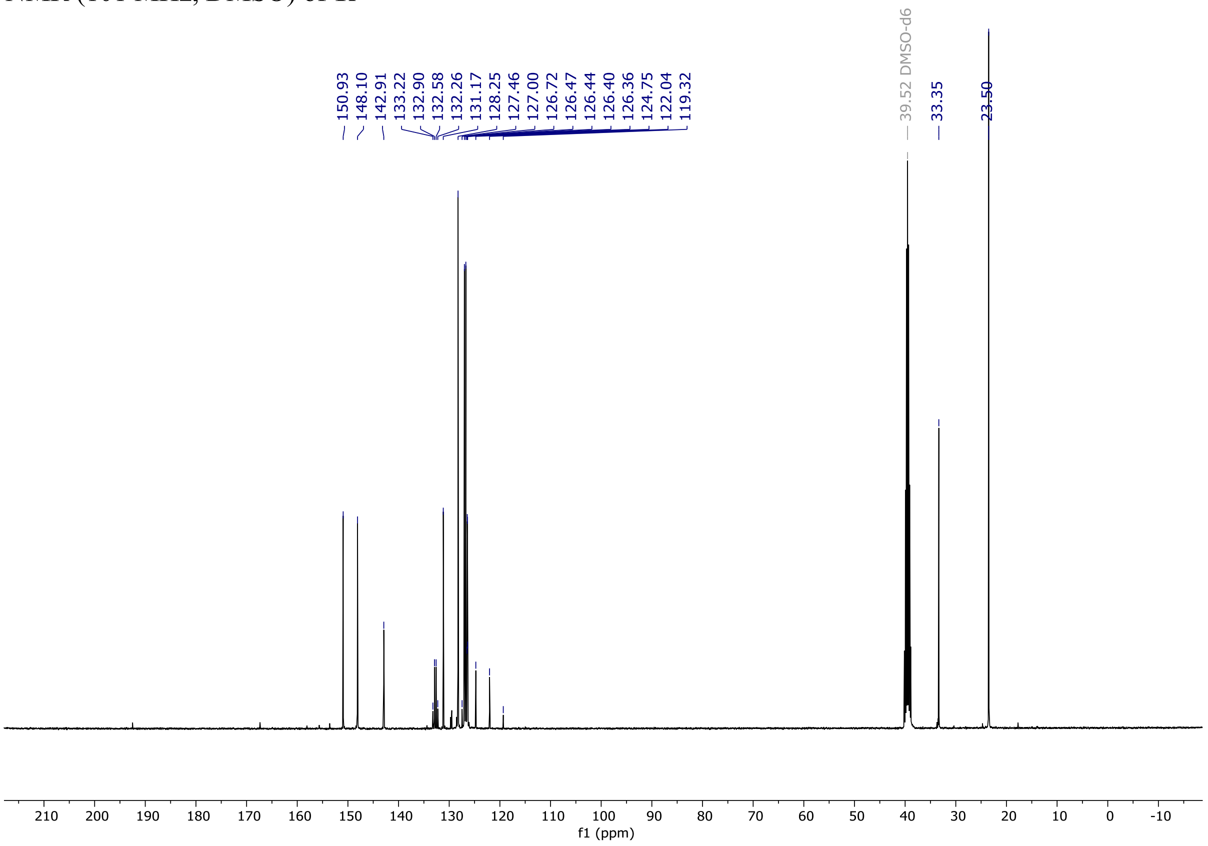

^{19}F NMR (282 MHz, DMSO) of **1r**

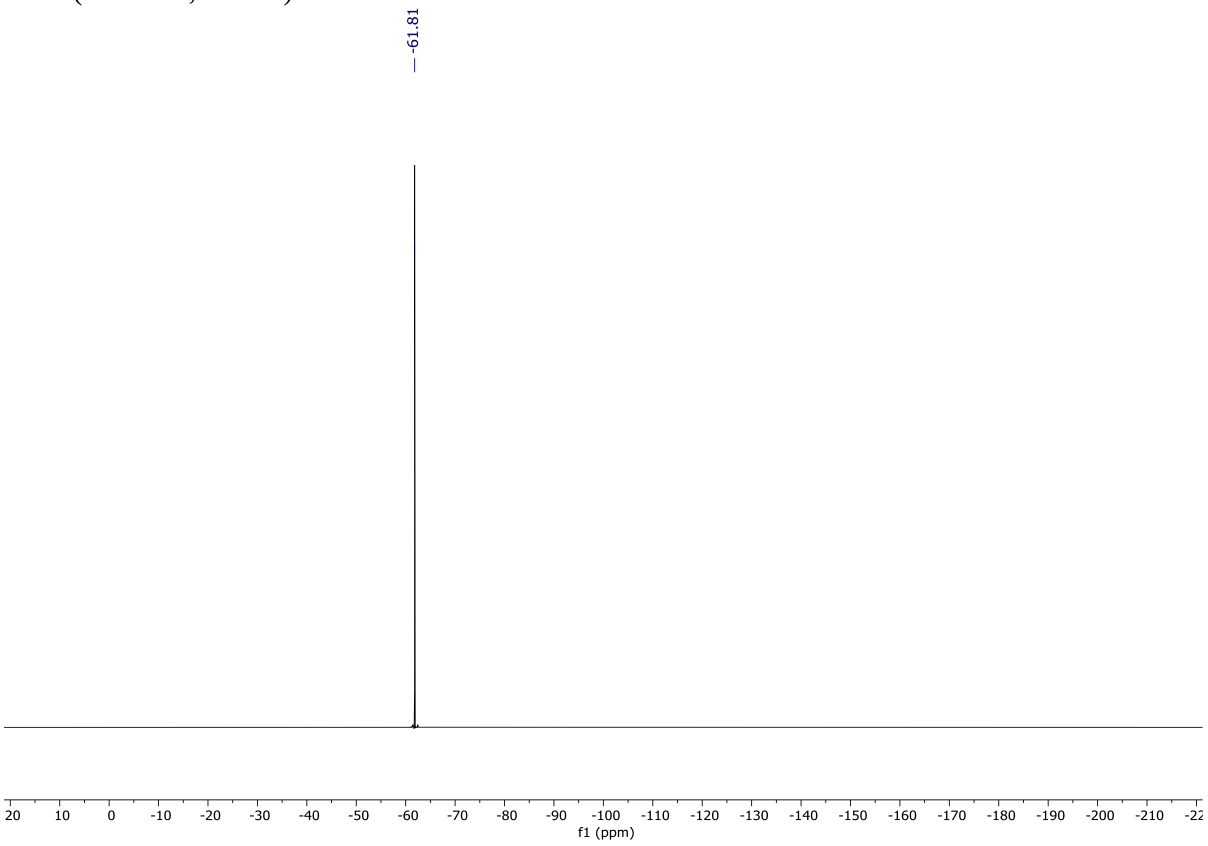

^{1}H NMR (400 MHz, DMSO) of **1s**

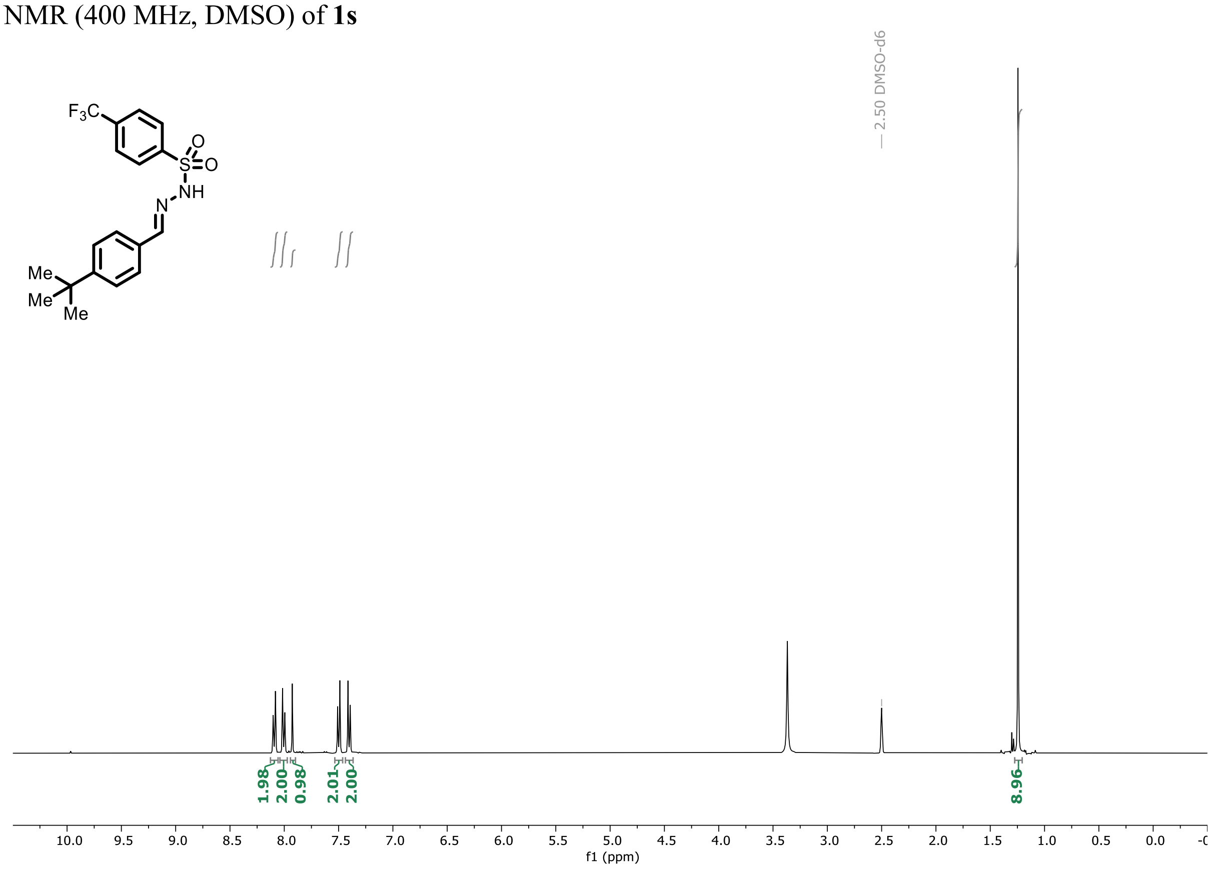

^{13}C NMR (101 MHz, DMSO) of **1s**

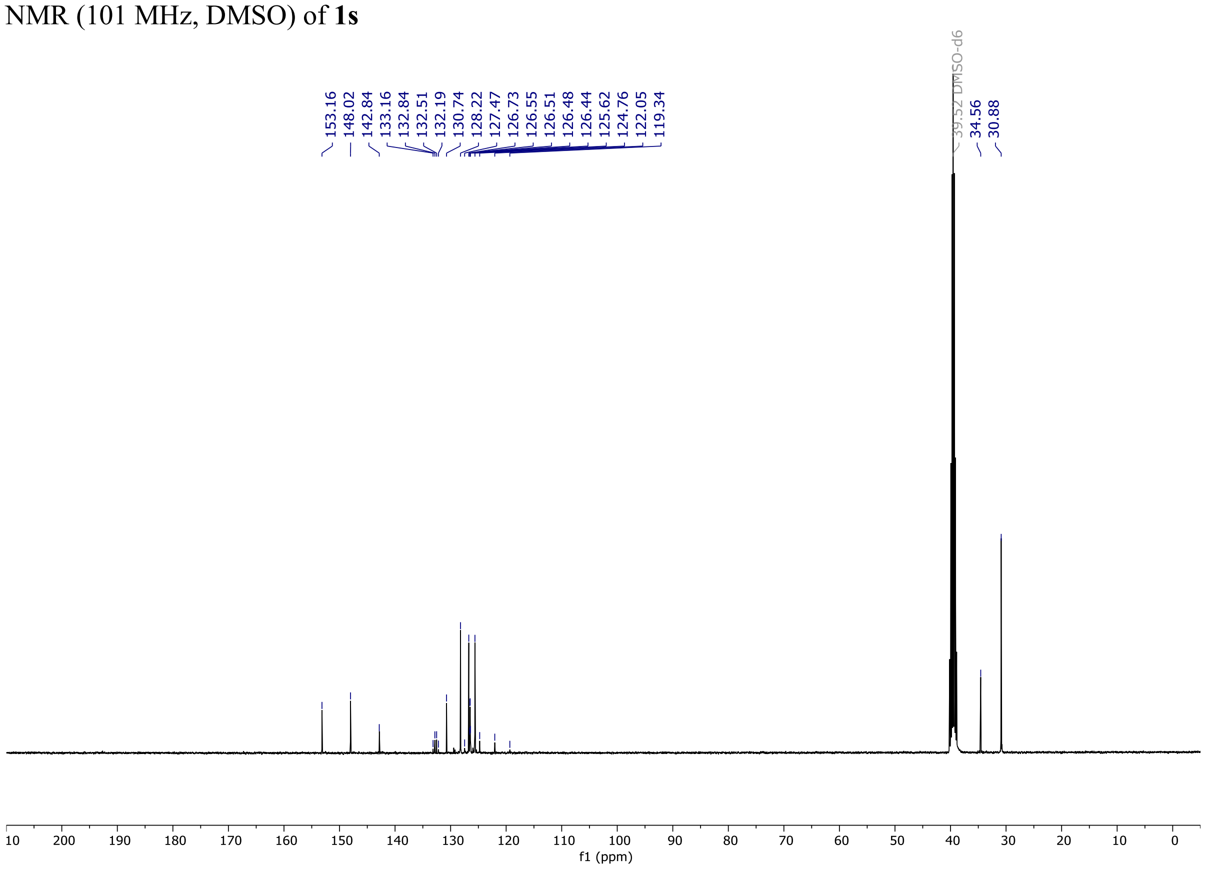

^{19}F NMR (282 MHz, DMSO) of **1s**

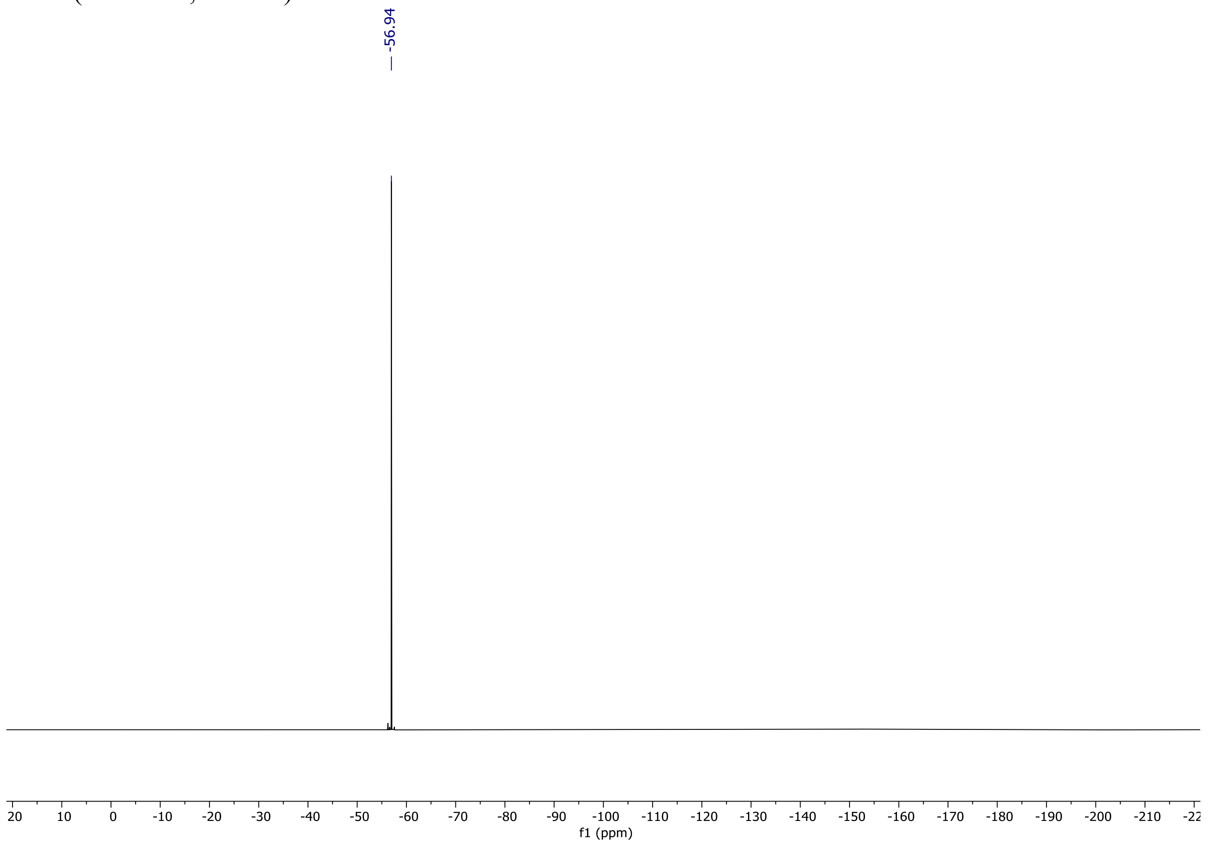

^{1}H NMR (400 MHz, DMSO) of **1t**

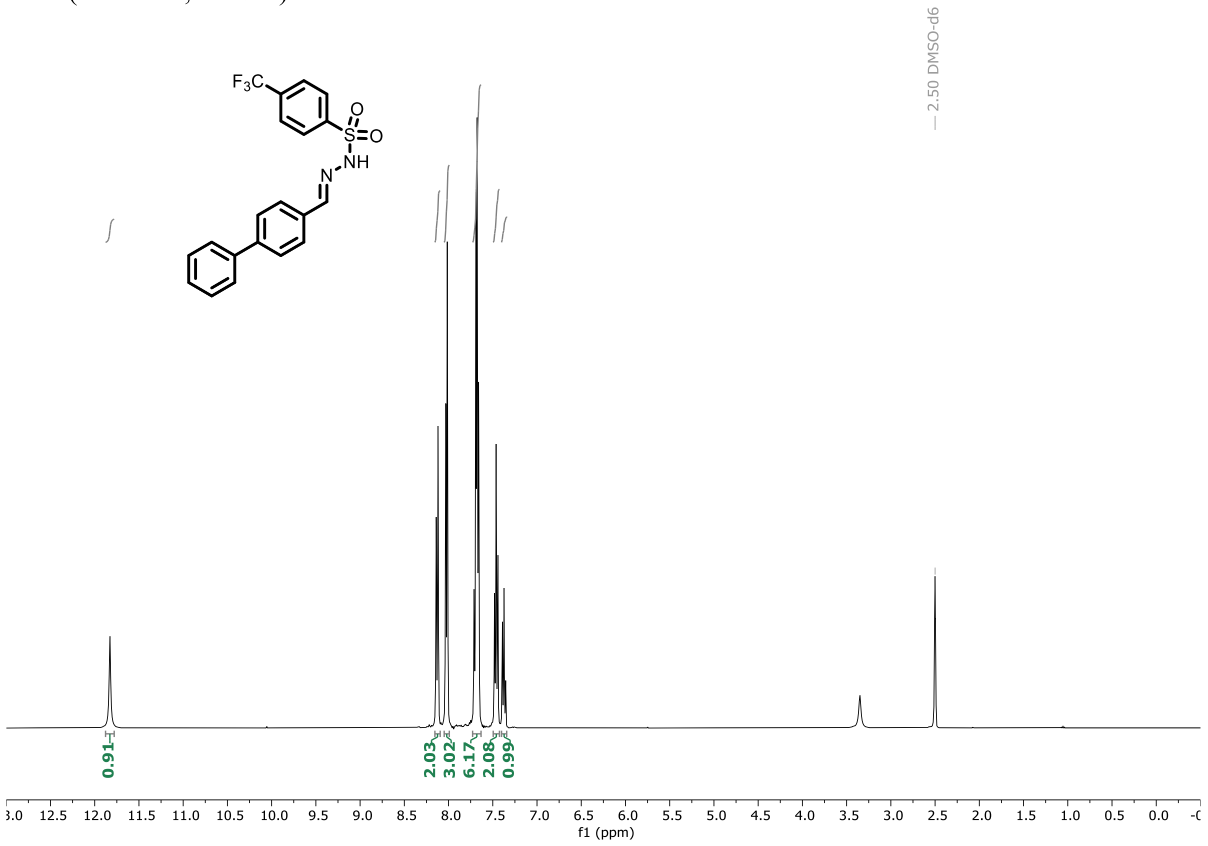

^{13}C NMR (101 MHz, DMSO) of **1t**

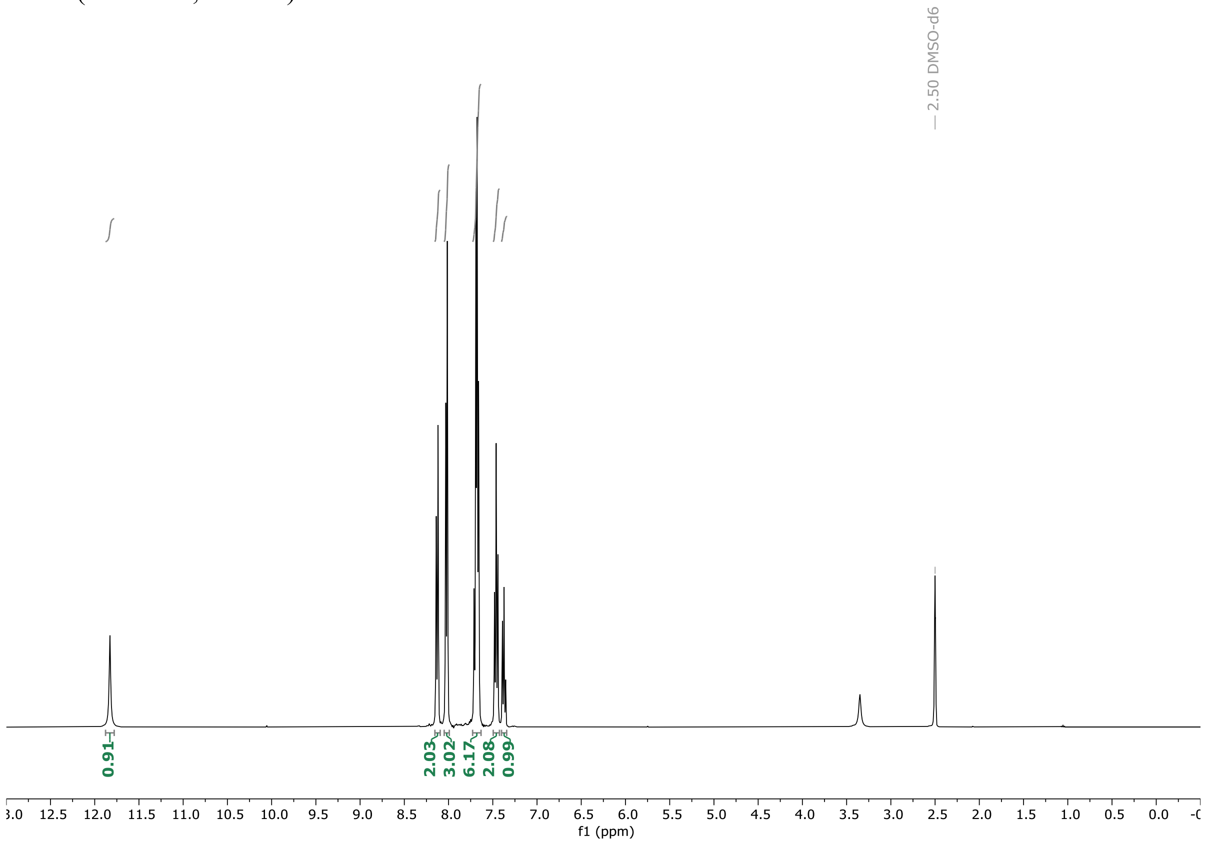

^{19}F NMR (282 MHz, DMSO) of **1t**

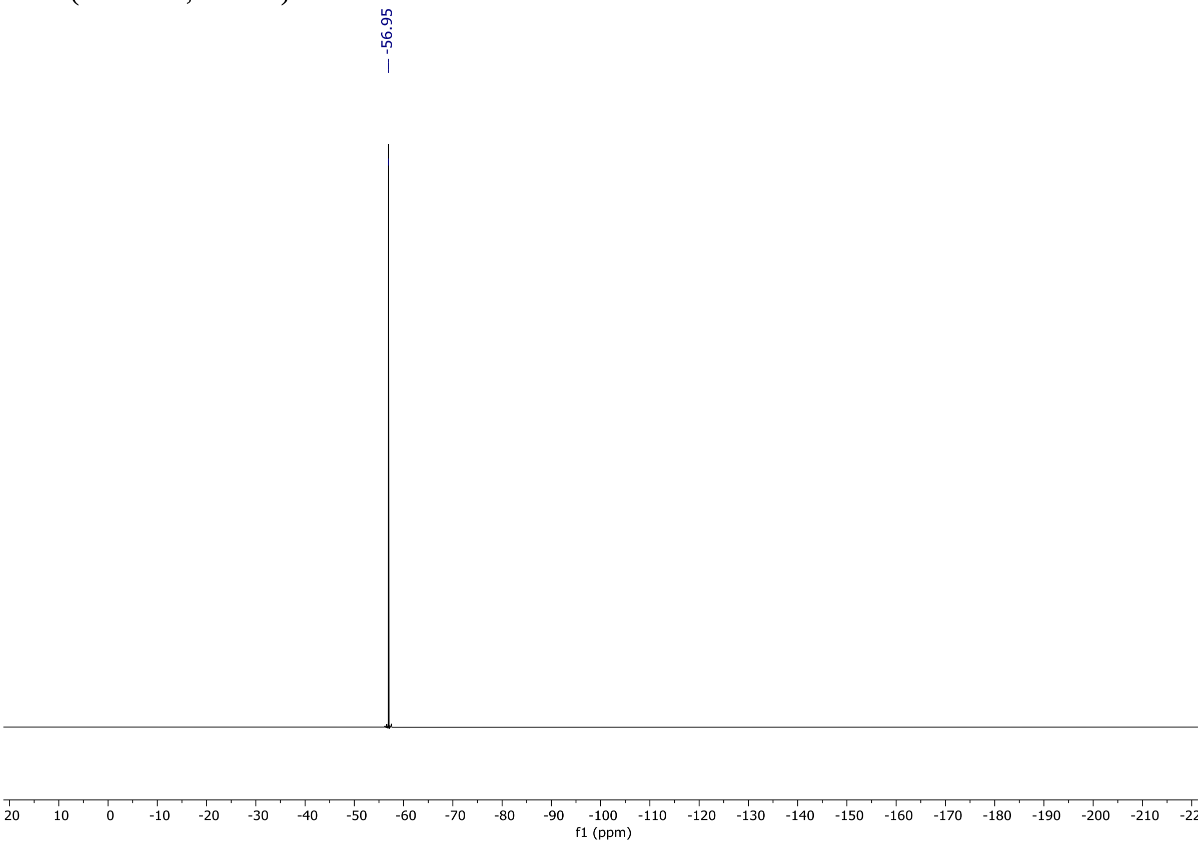

^1H NMR (400 MHz, DMSO) of **1u**

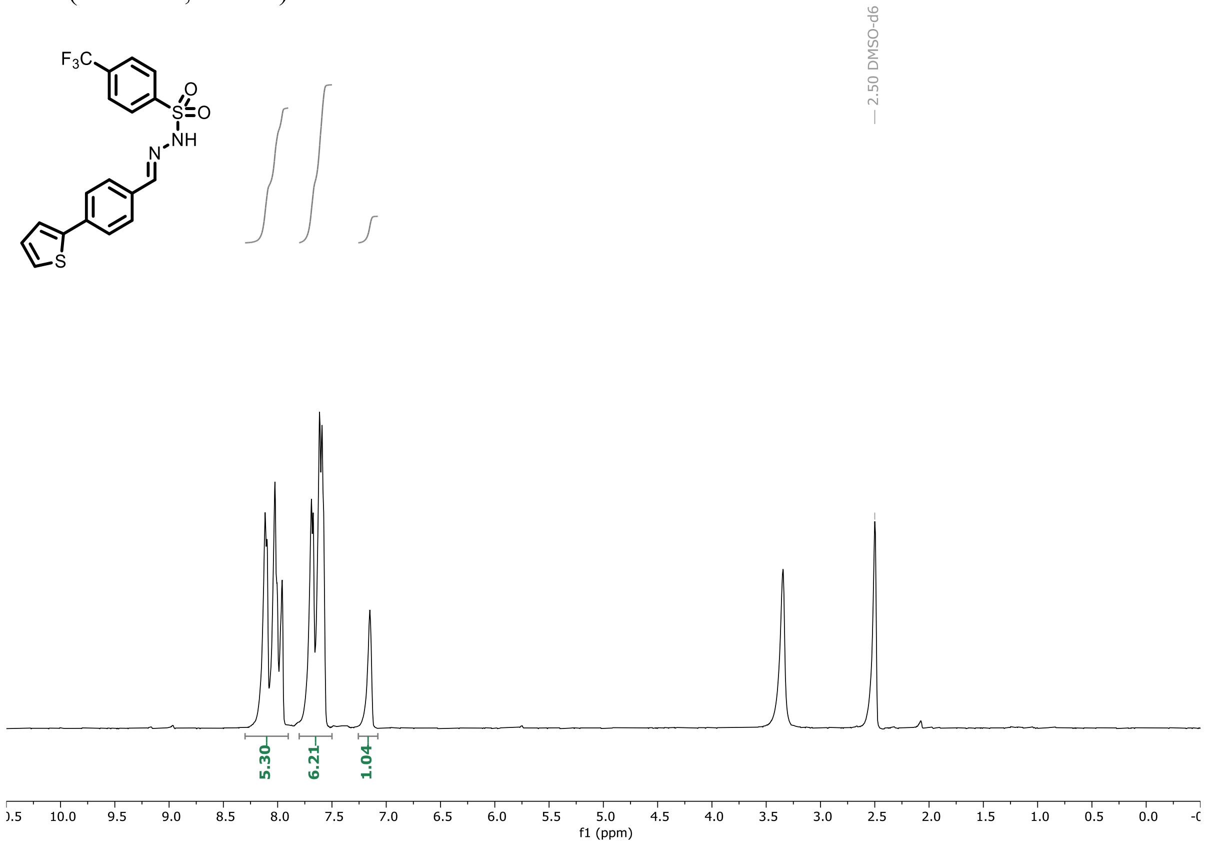

^{13}C NMR (101 MHz, DMSO) of **1u**

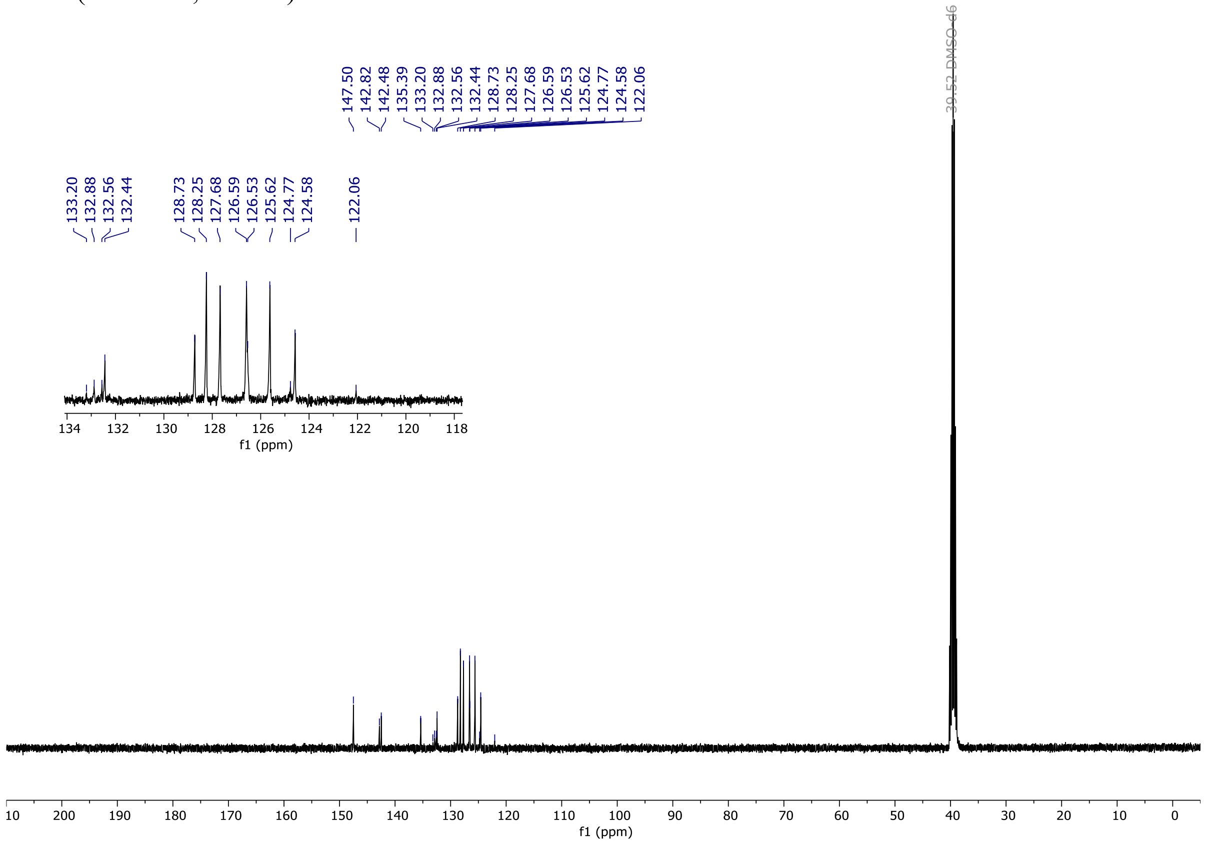

^{19}F NMR (282 MHz, DMSO) of **1u**

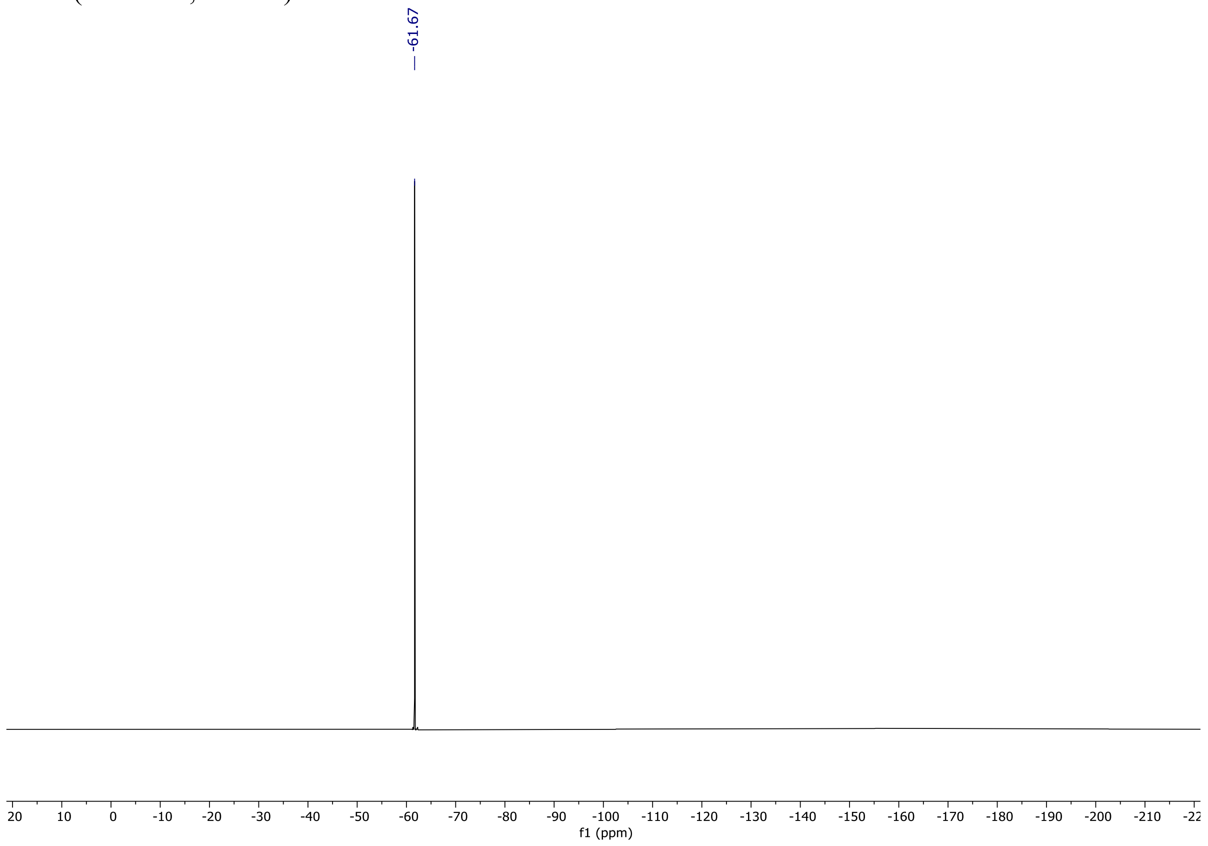

^{1}H NMR (400 MHz, DMSO) of **1v**

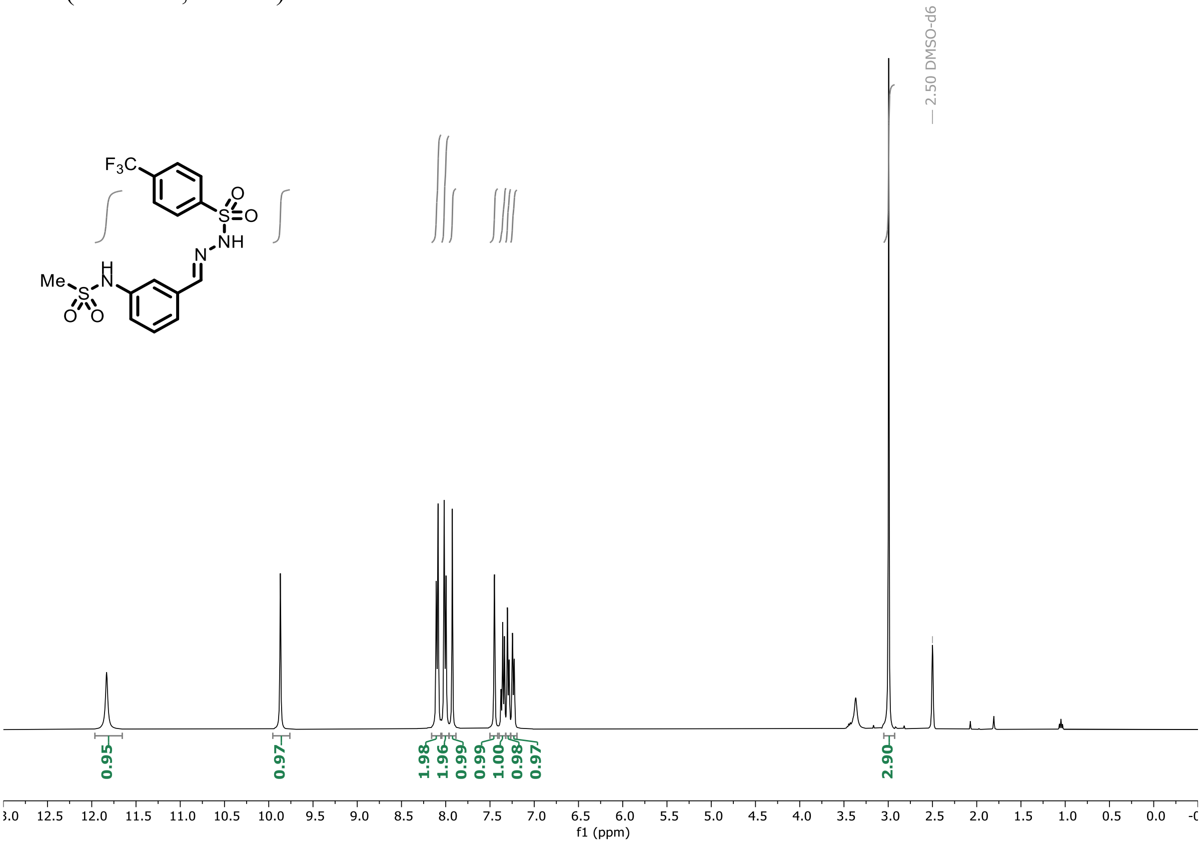

^{13}C NMR (101 MHz, DMSO) of **1v**

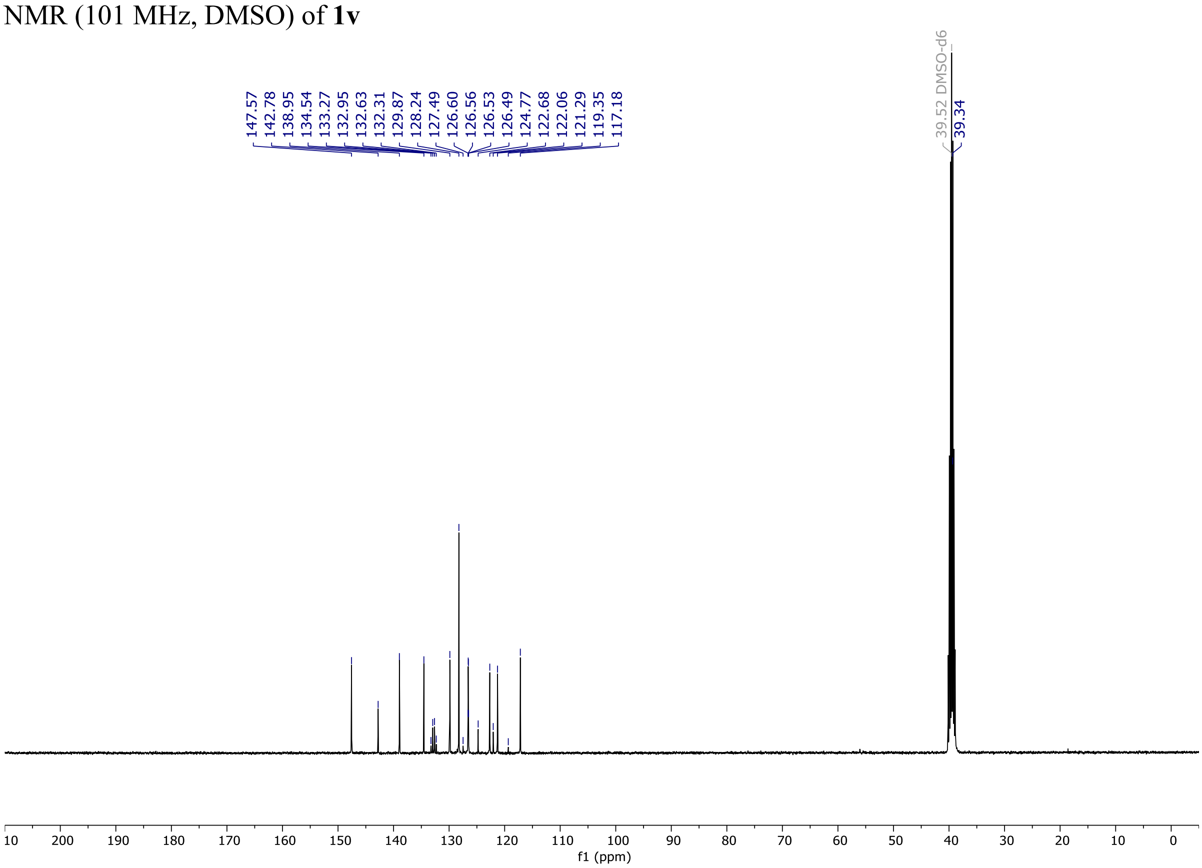

^{19}F NMR (282 MHz, DMSO) of **1v**

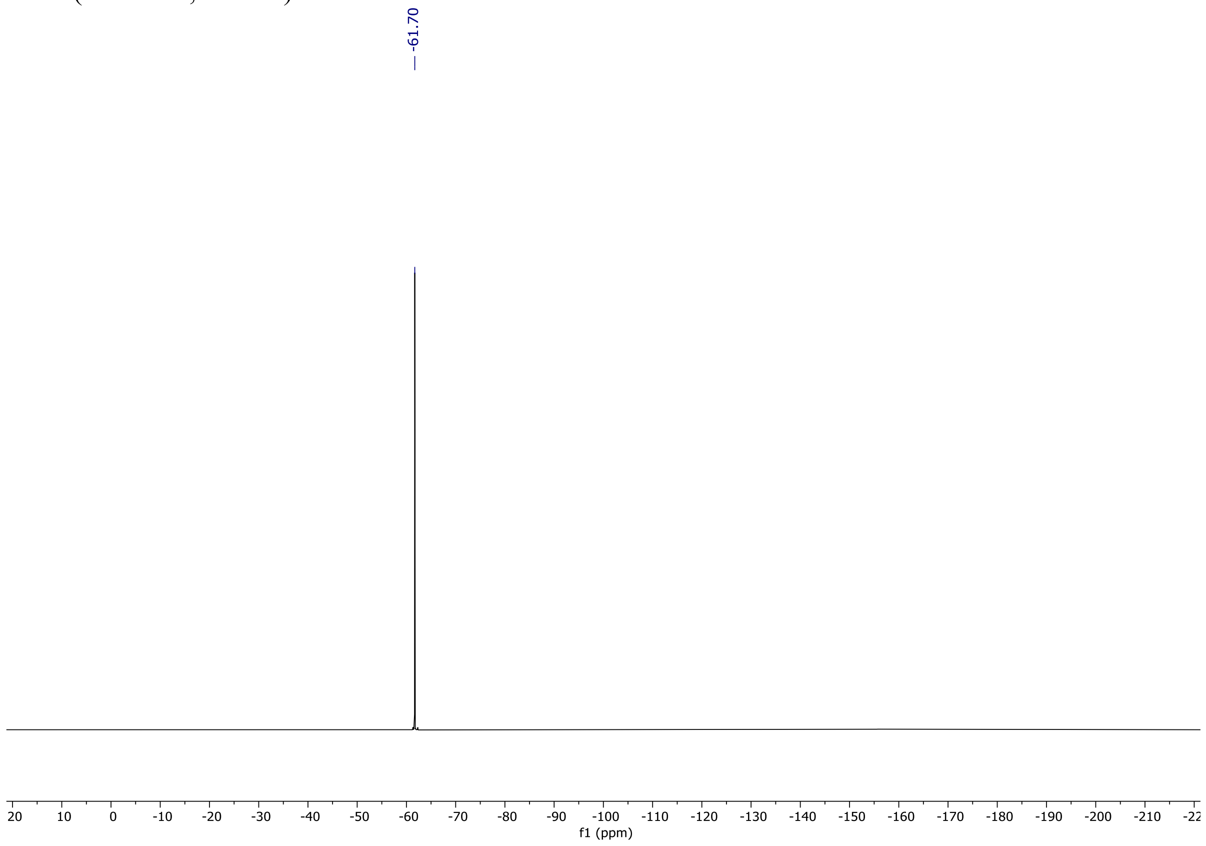

^{1}H NMR (400 MHz,$CDCl_3$) of **1w**

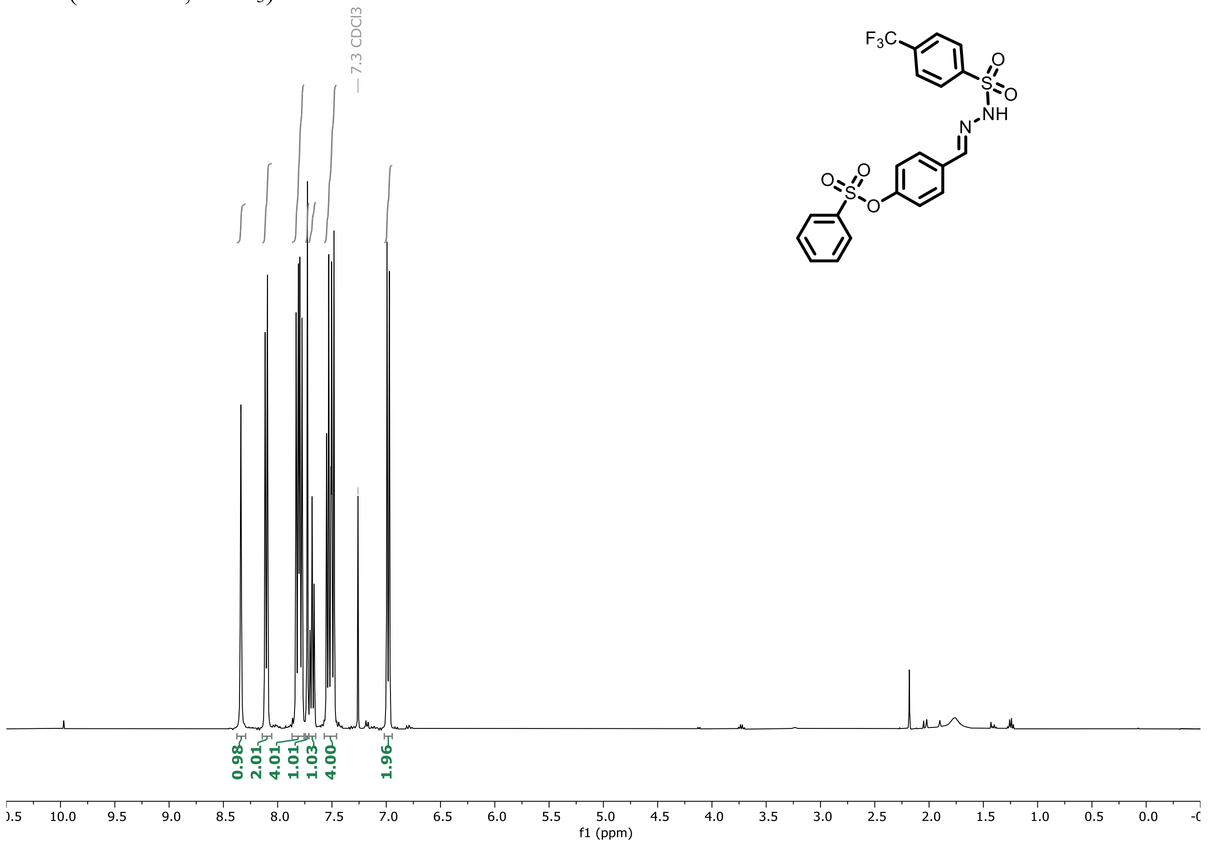

^{13}C NMR (101 MHz, $CDCl_3$) of **1w**

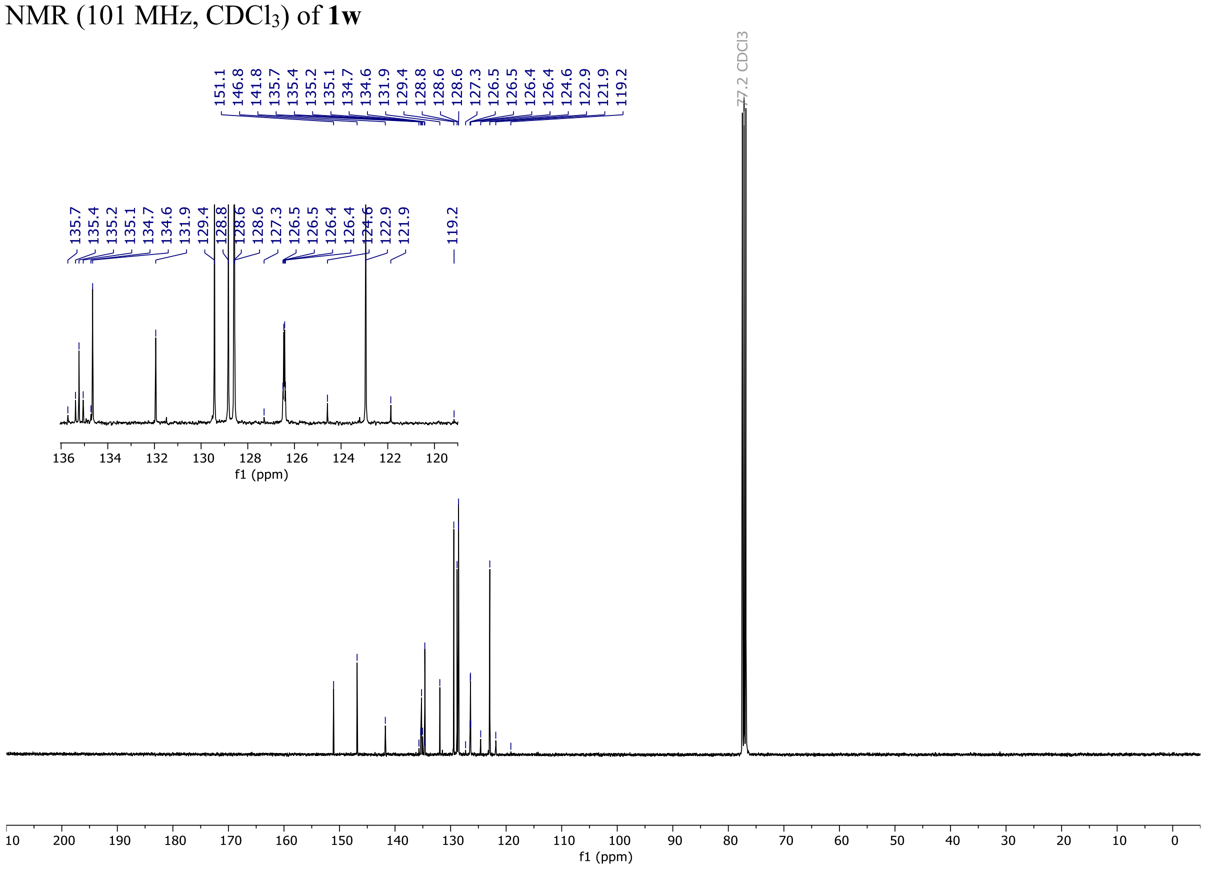

^{19}F NMR (282 MHz, $CDCl_3$) of **1w**

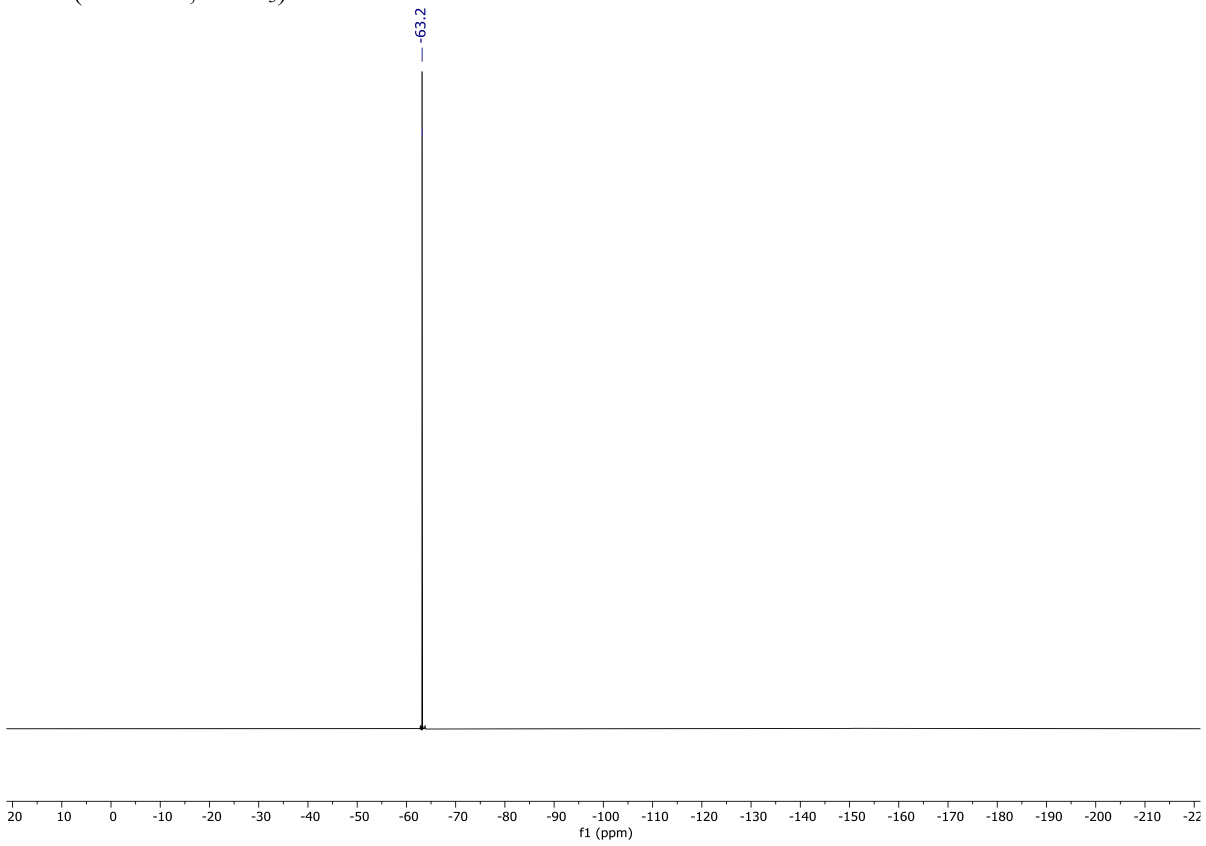

^{1}H NMR (400 MHz, $CDCl_3$) of **1x**

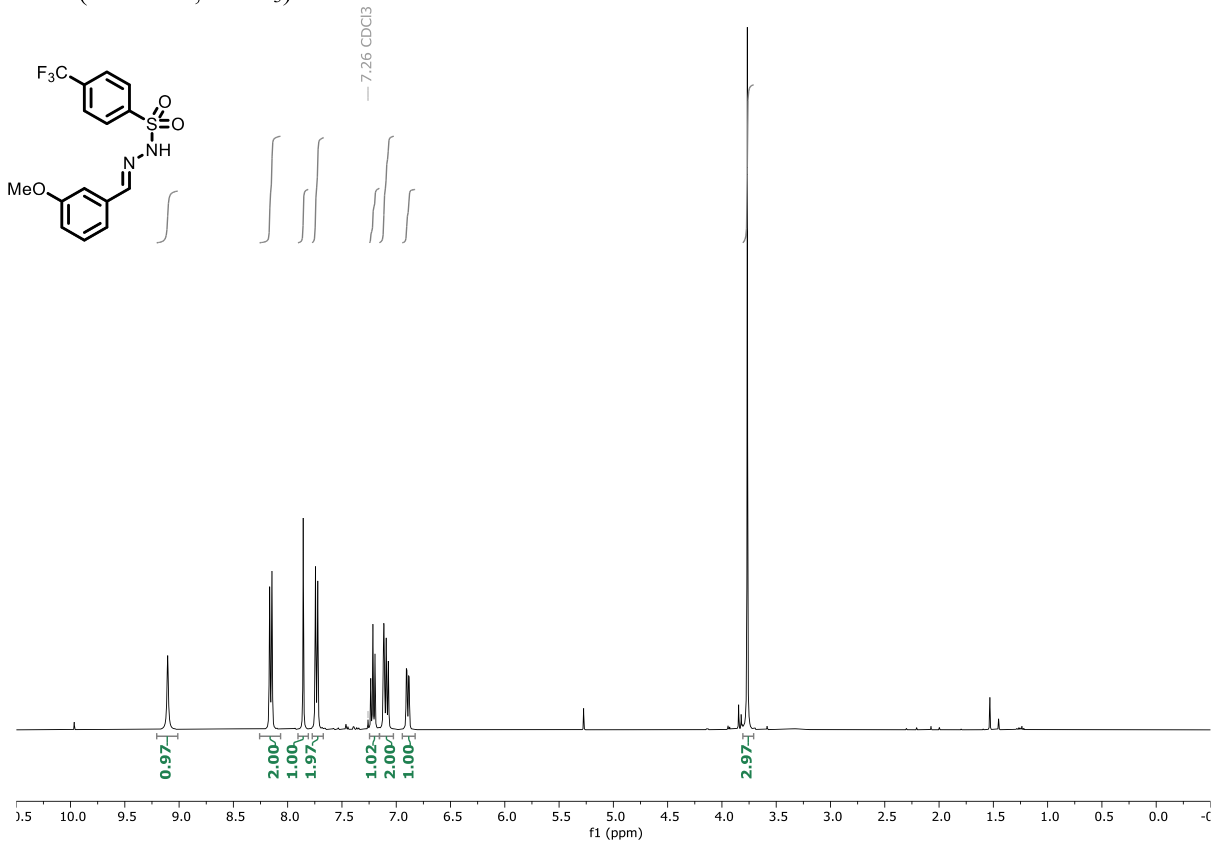

^{13}C NMR (101 MHz, $CDCl_3$) of **1x**

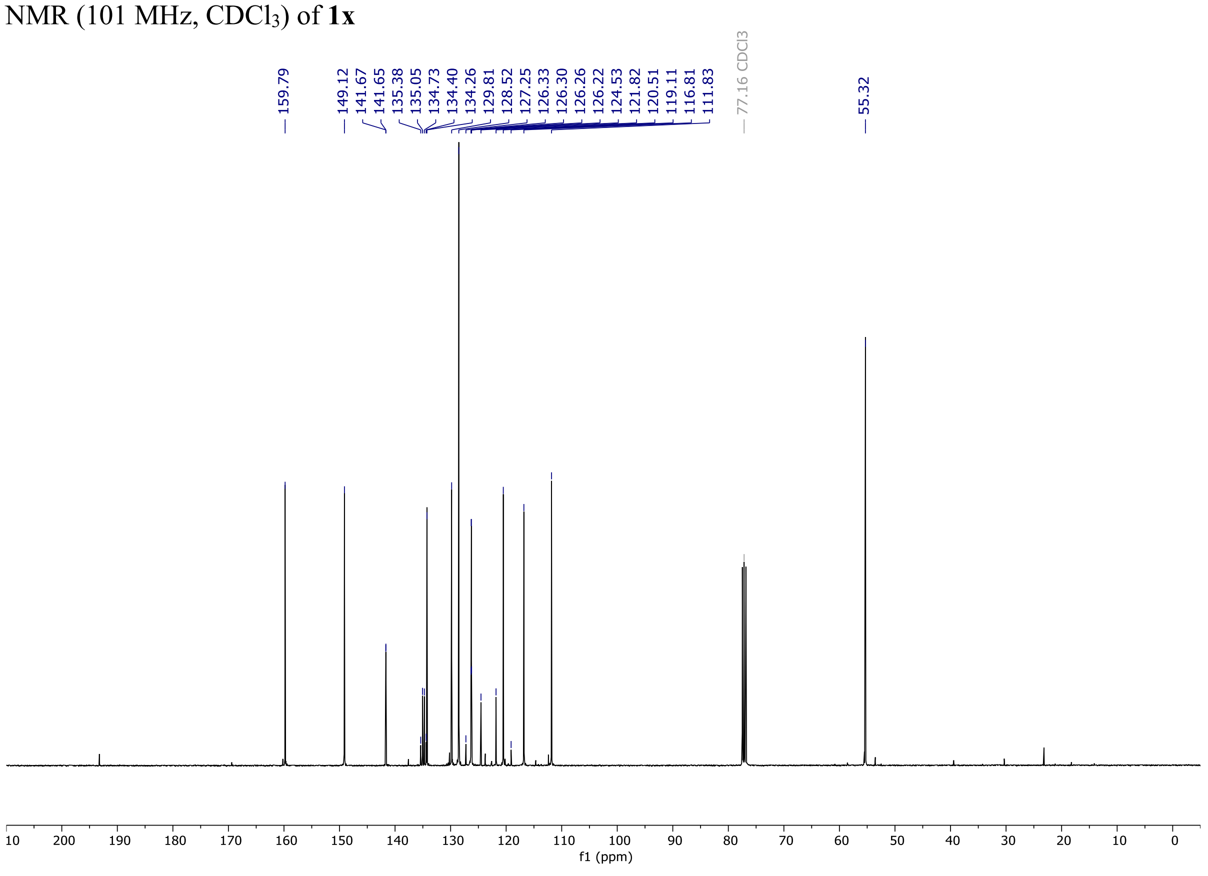

^{19}F NMR (282 MHz, $CDCl_3$) of **1x**

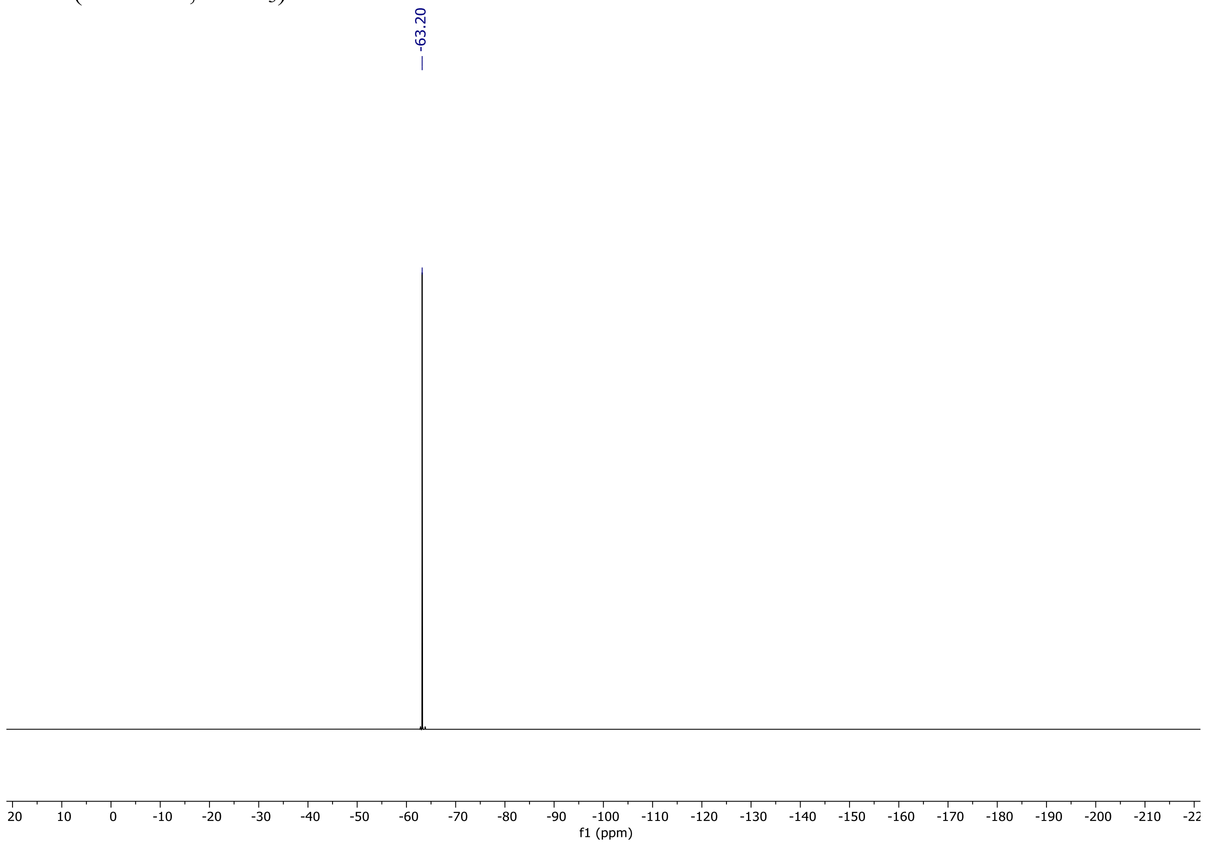

^{1}H NMR (400 MHz, DMSO) of **1y** (* = 10% impurity)

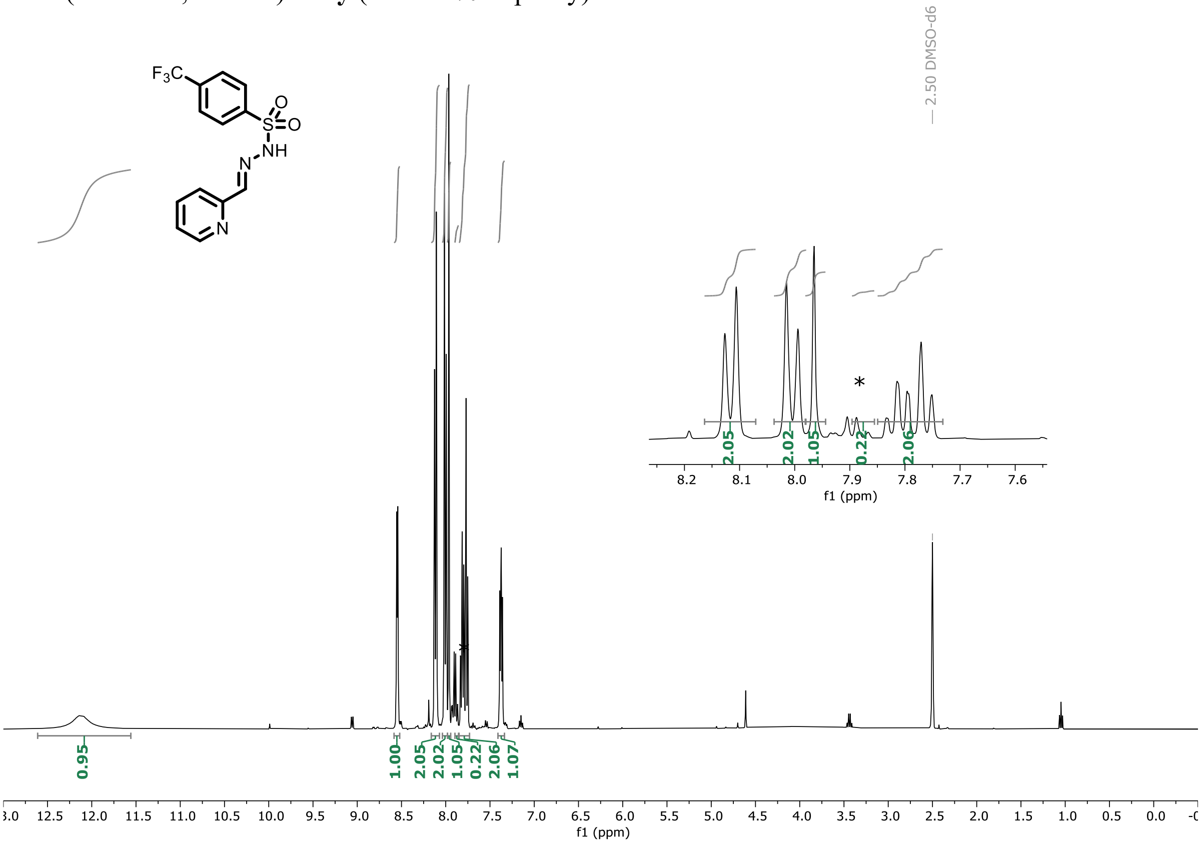

^{13}C NMR (101 MHz, DMSO) of **1y**

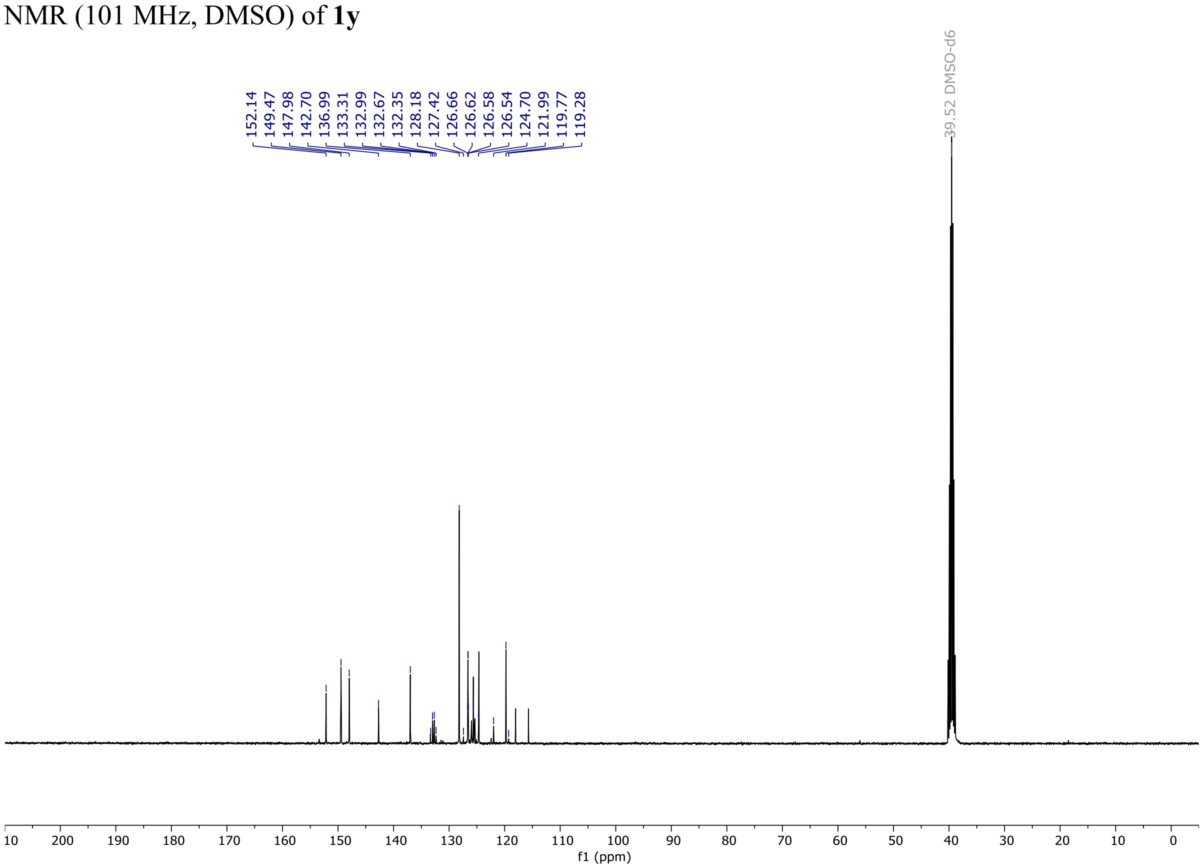

^{19}F NMR (282 MHz, DMSO) of **1y** (* = impurity)

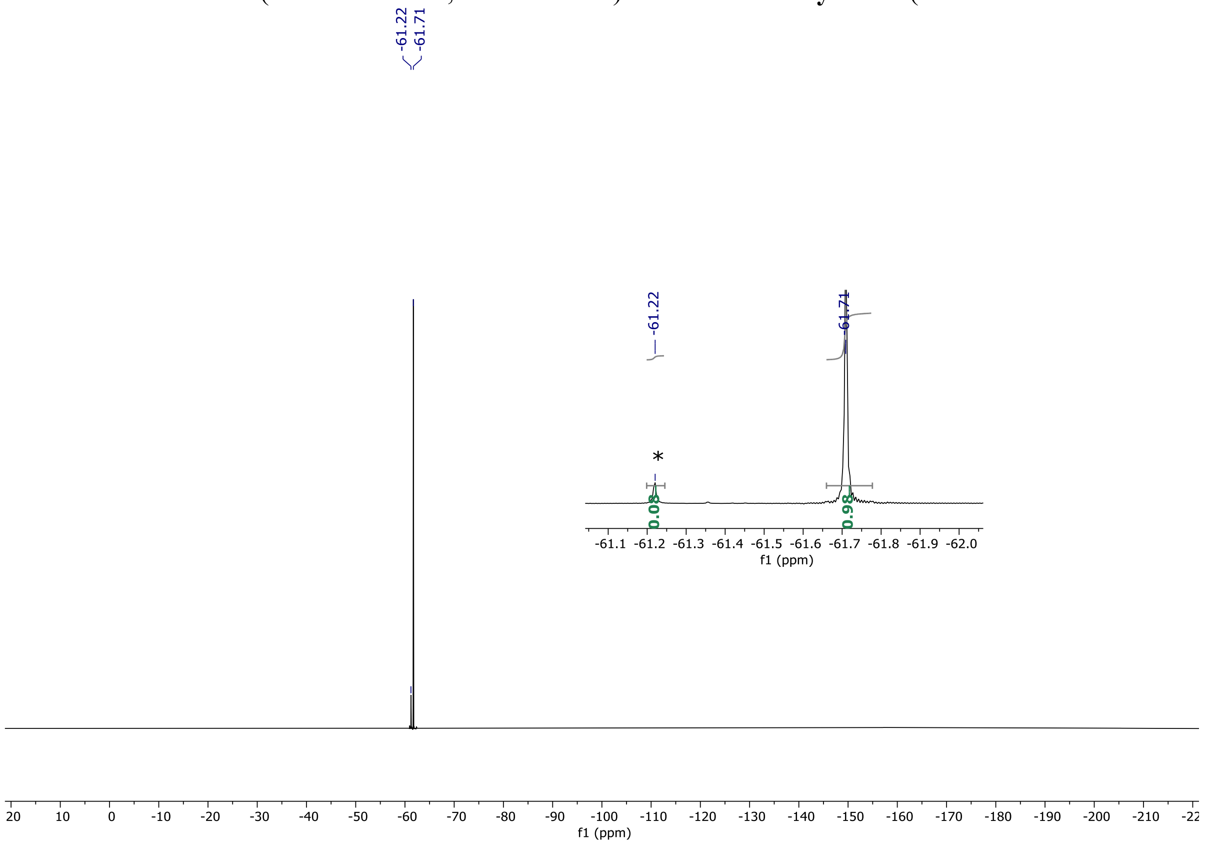

^{1}H NMR (400 MHz, $CDCl_3$) of **1z**

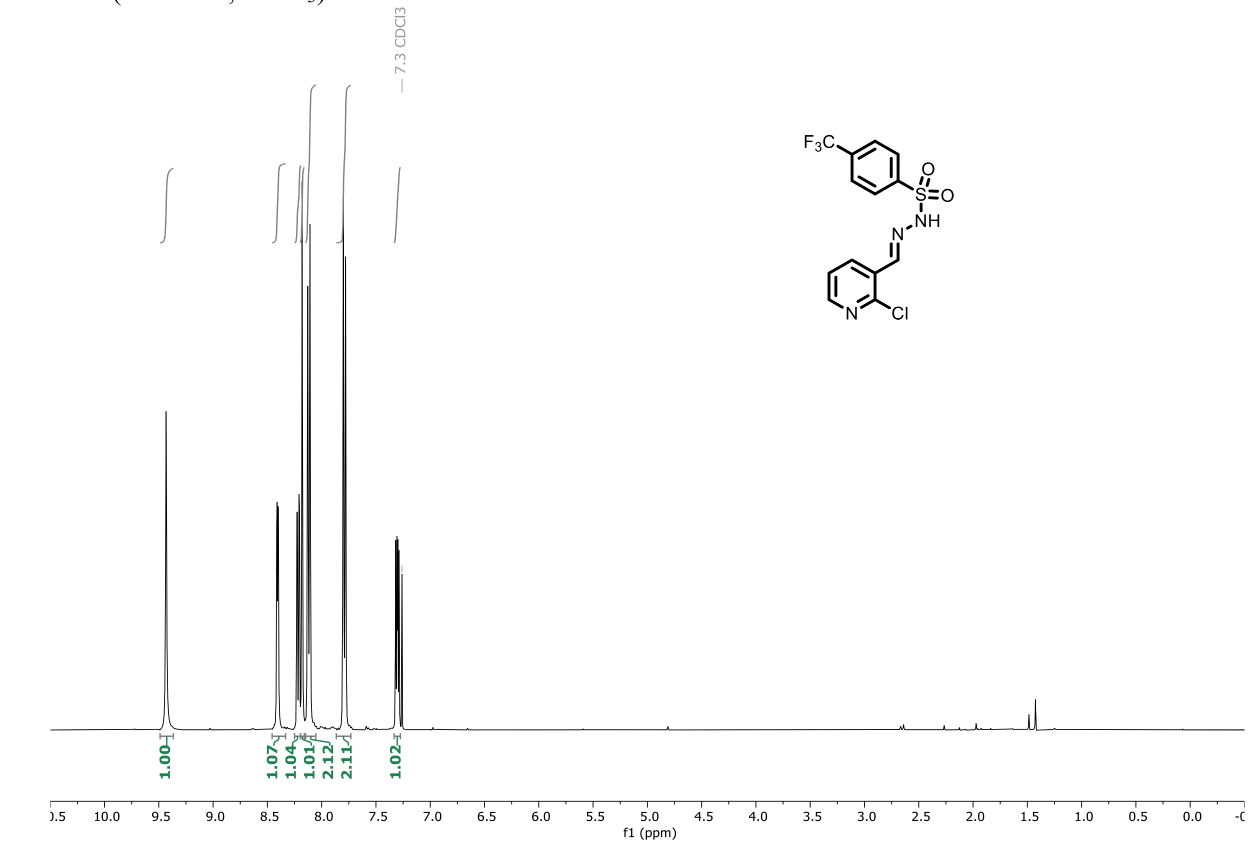

^{13}C NMR (101 MHz, $CDCl_3$) of **1z**

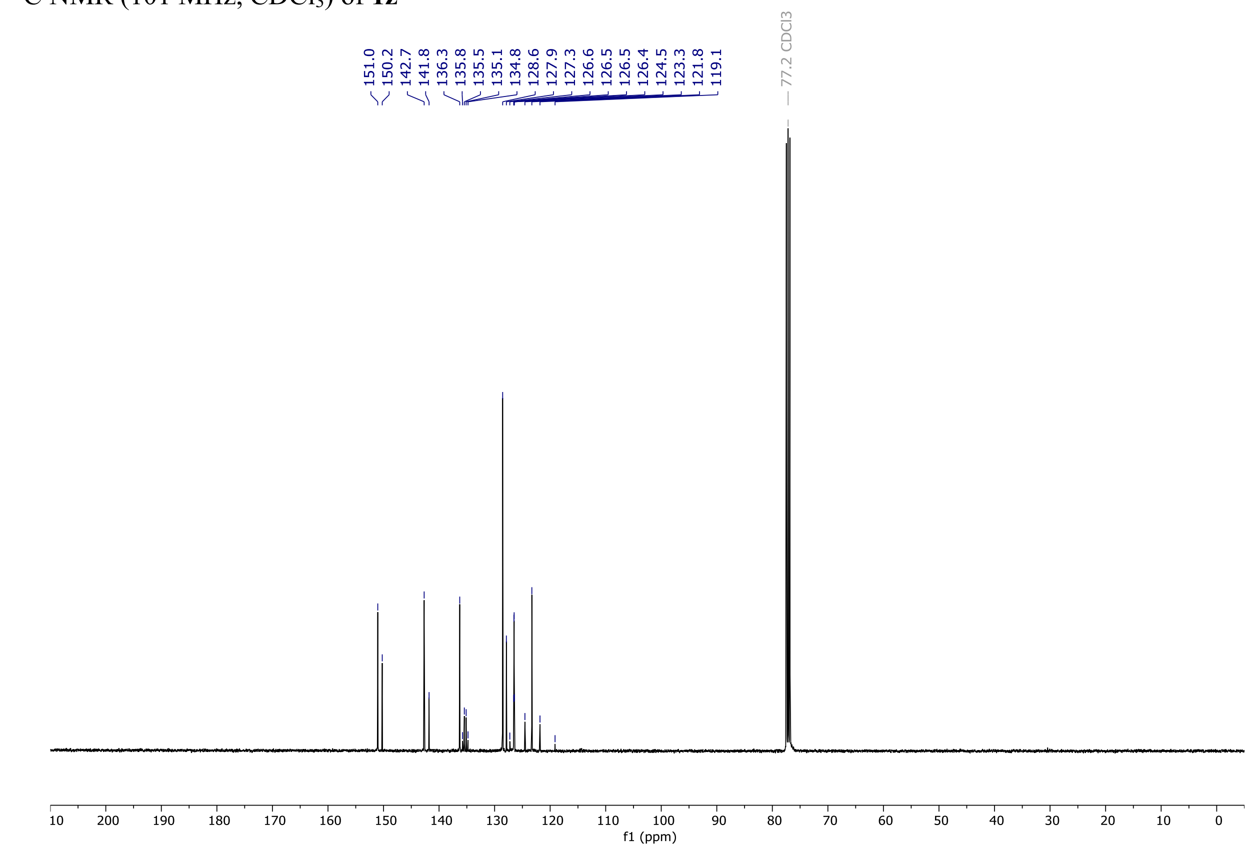

^{19}F NMR (282 MHz, $CDCl_3$) of **1z**

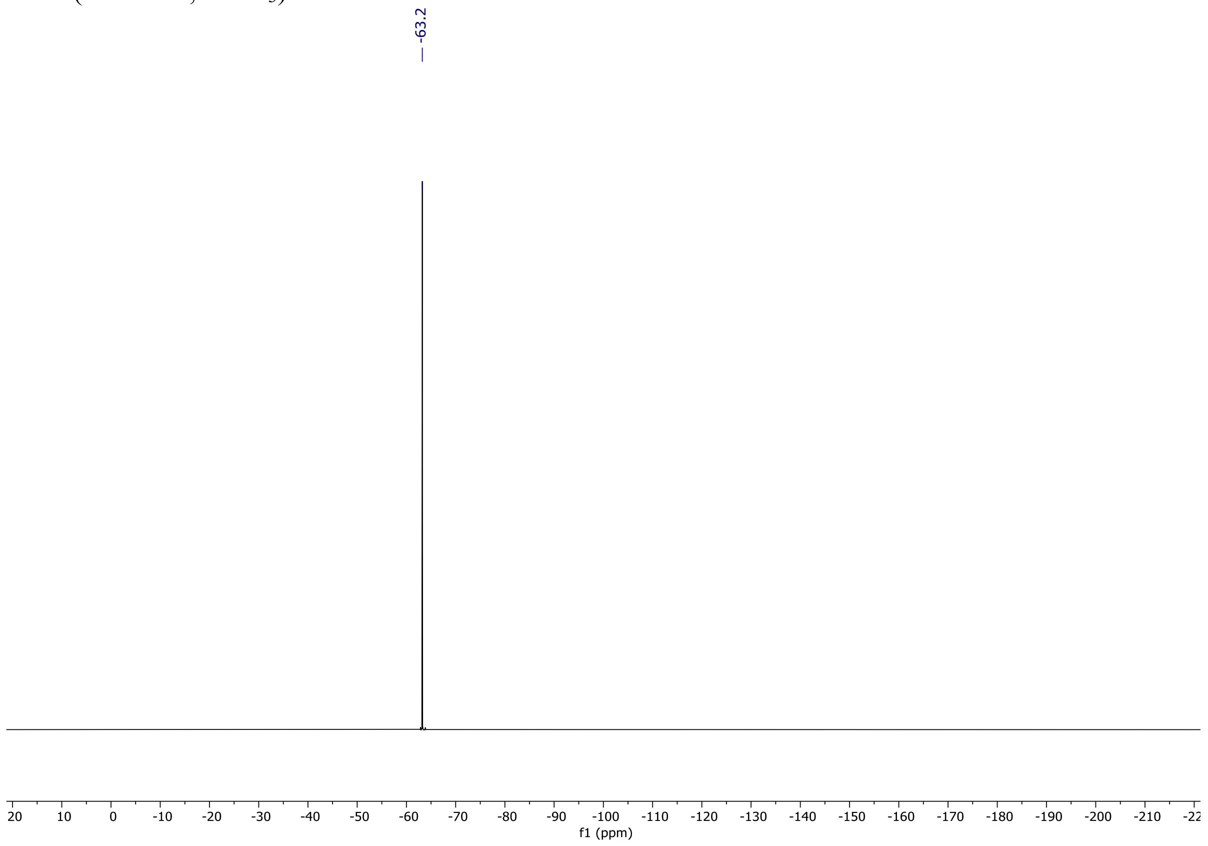

^{1}H NMR (400 MHz, DMSO) of **1aa**

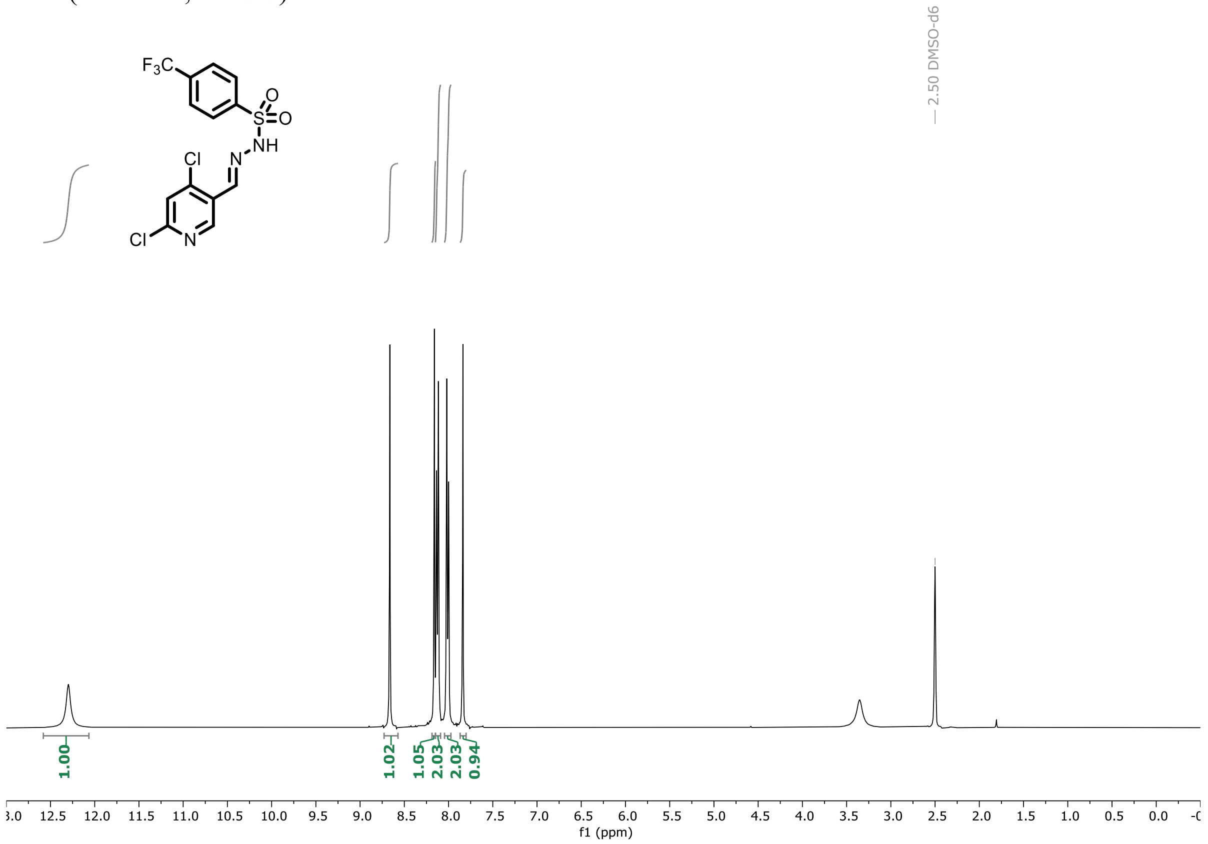

^{13}C NMR (101 MHz, DMSO) of **1aa**

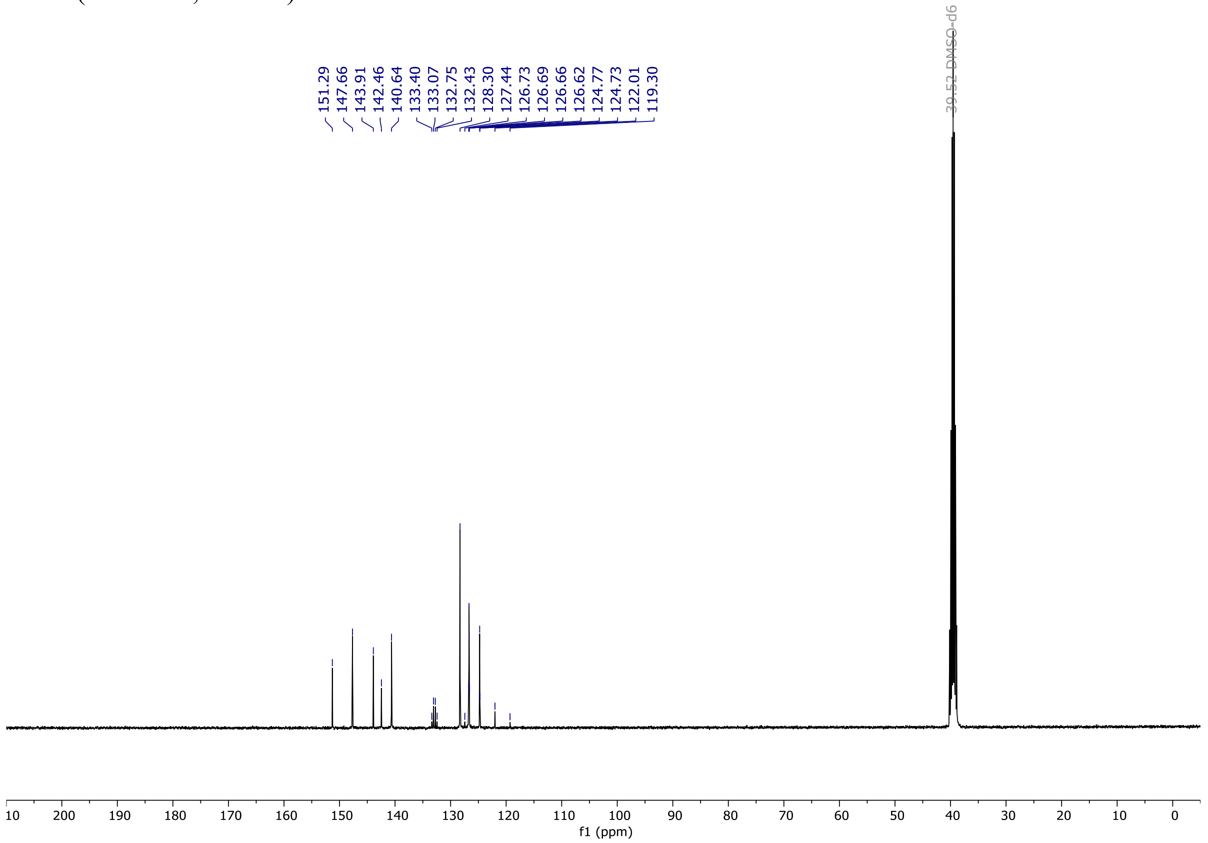

^{19}F NMR (282 MHz, DMSO) of **1aa**

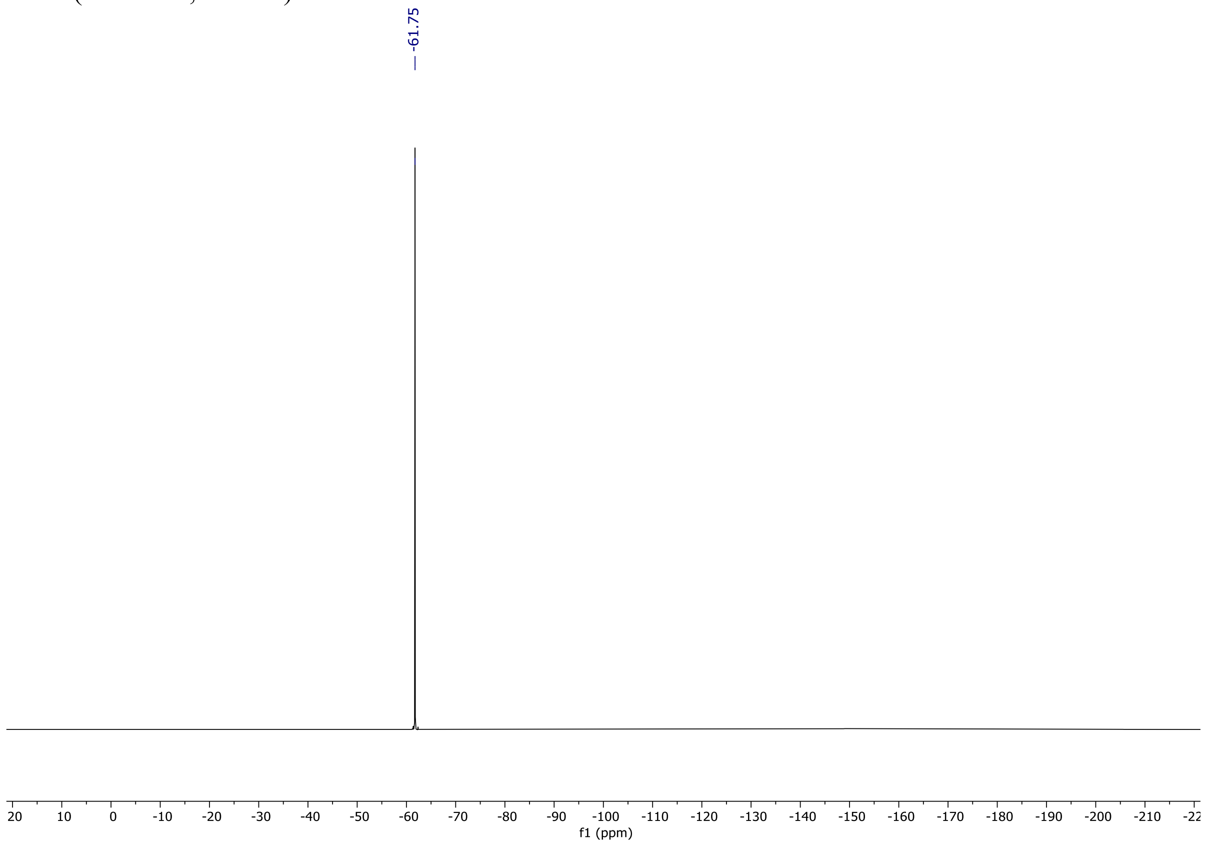

^{1}H NMR (400 MHz, DMSO) of **1ab**

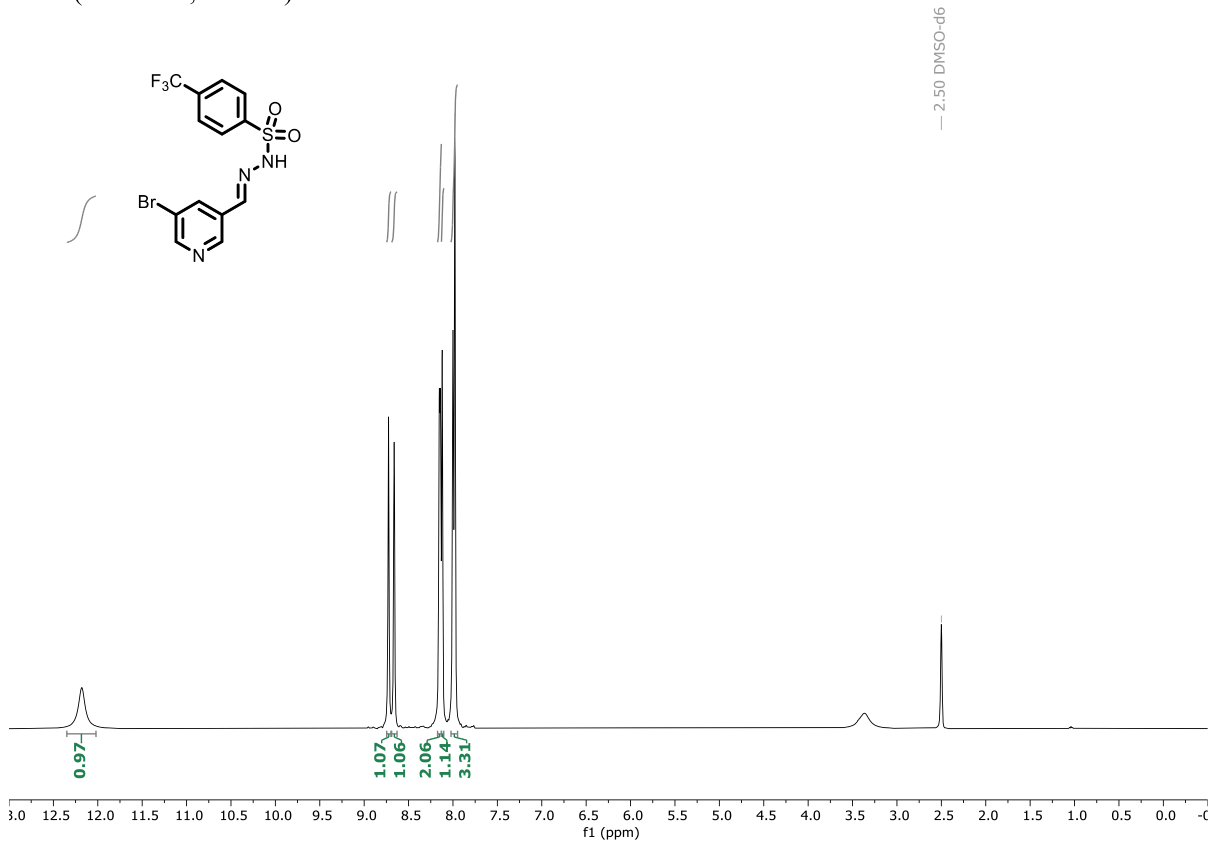

^{13}C NMR (101 MHz, DMSO) of **1ab**

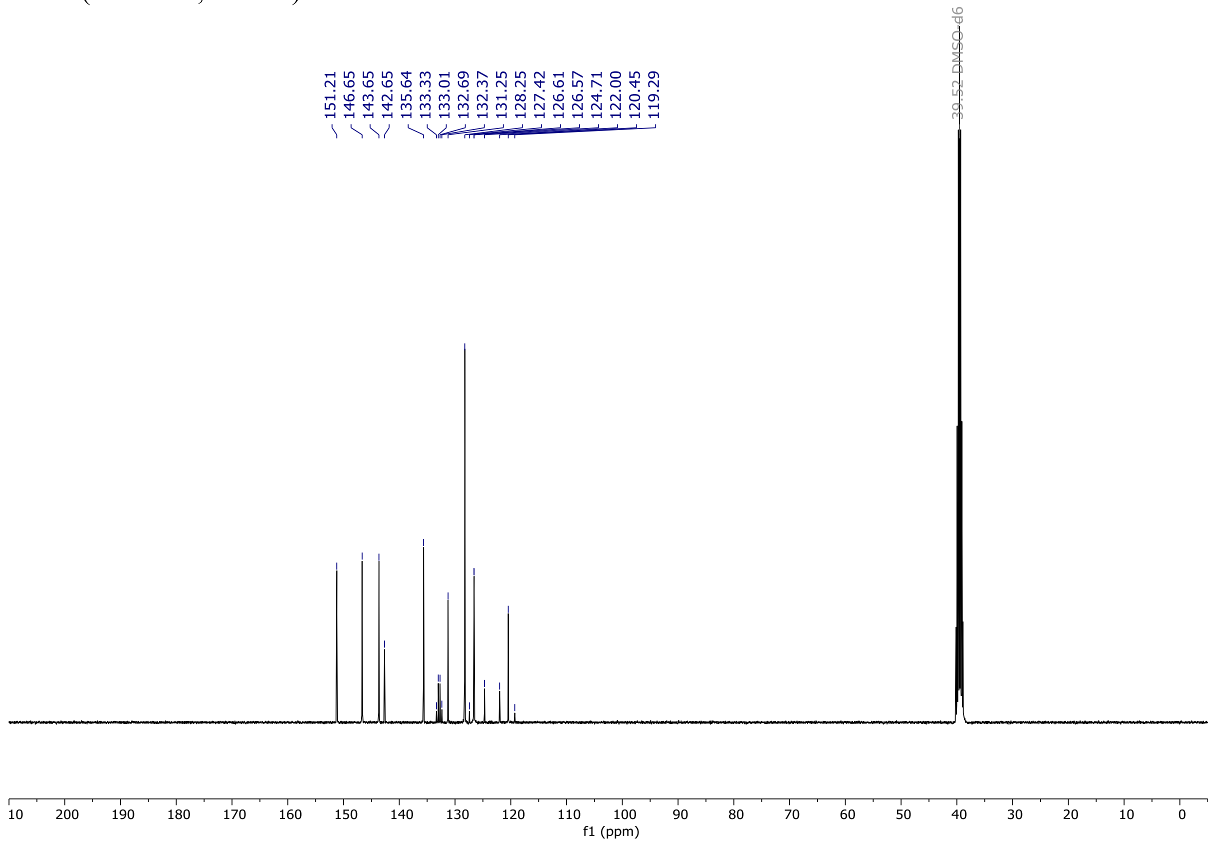

^{19}F NMR (282 MHz, DMSO) of **1ab**

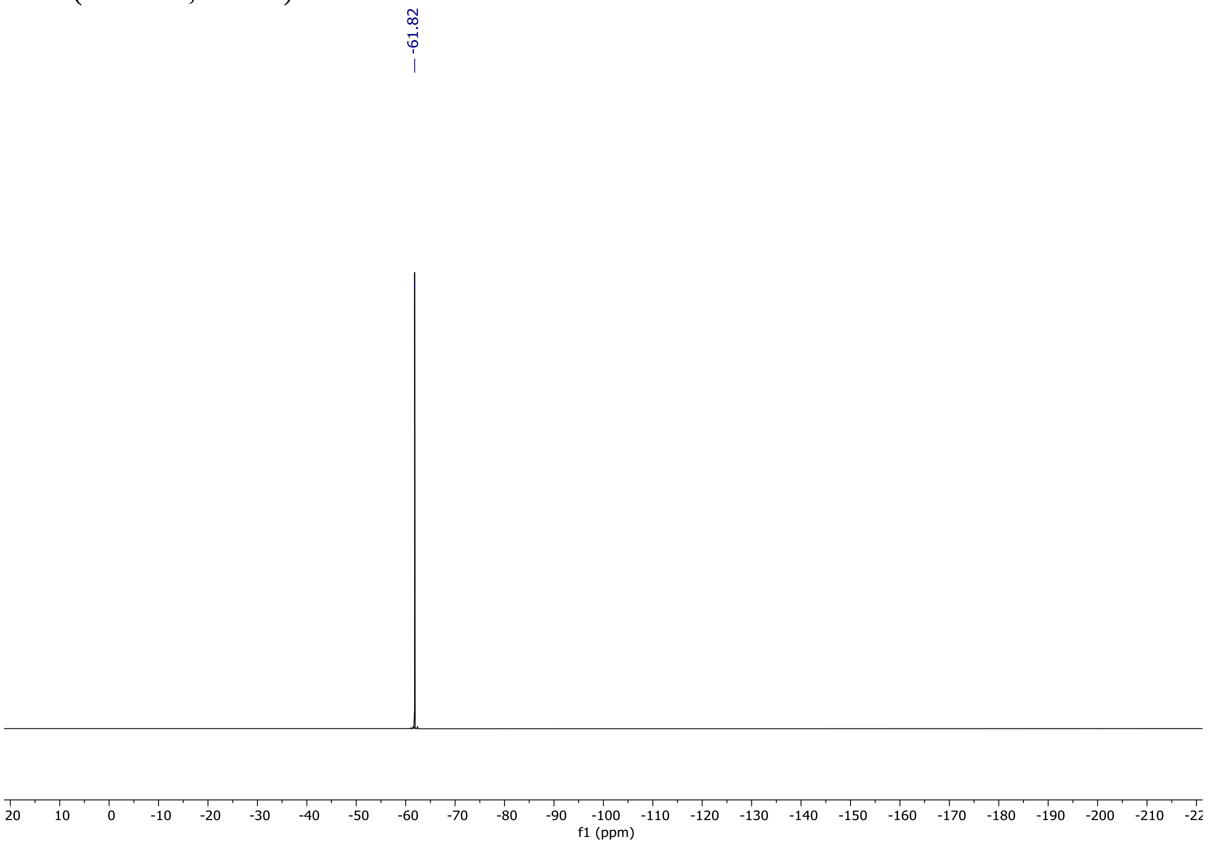

^{1}H NMR (400 MHz, $CDCl_3$) of **1ac**

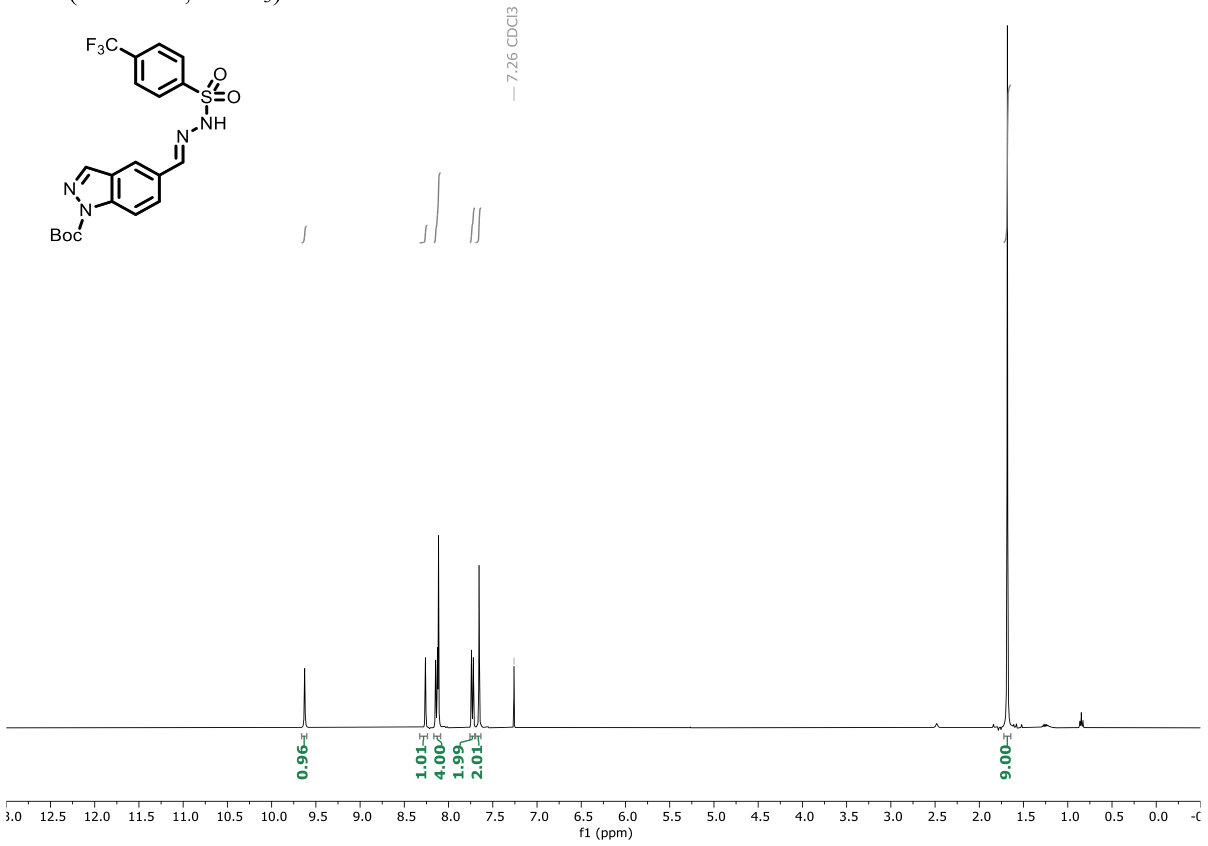

^{13}C NMR (101 MHz, $CDCl_3$) of **1ac**

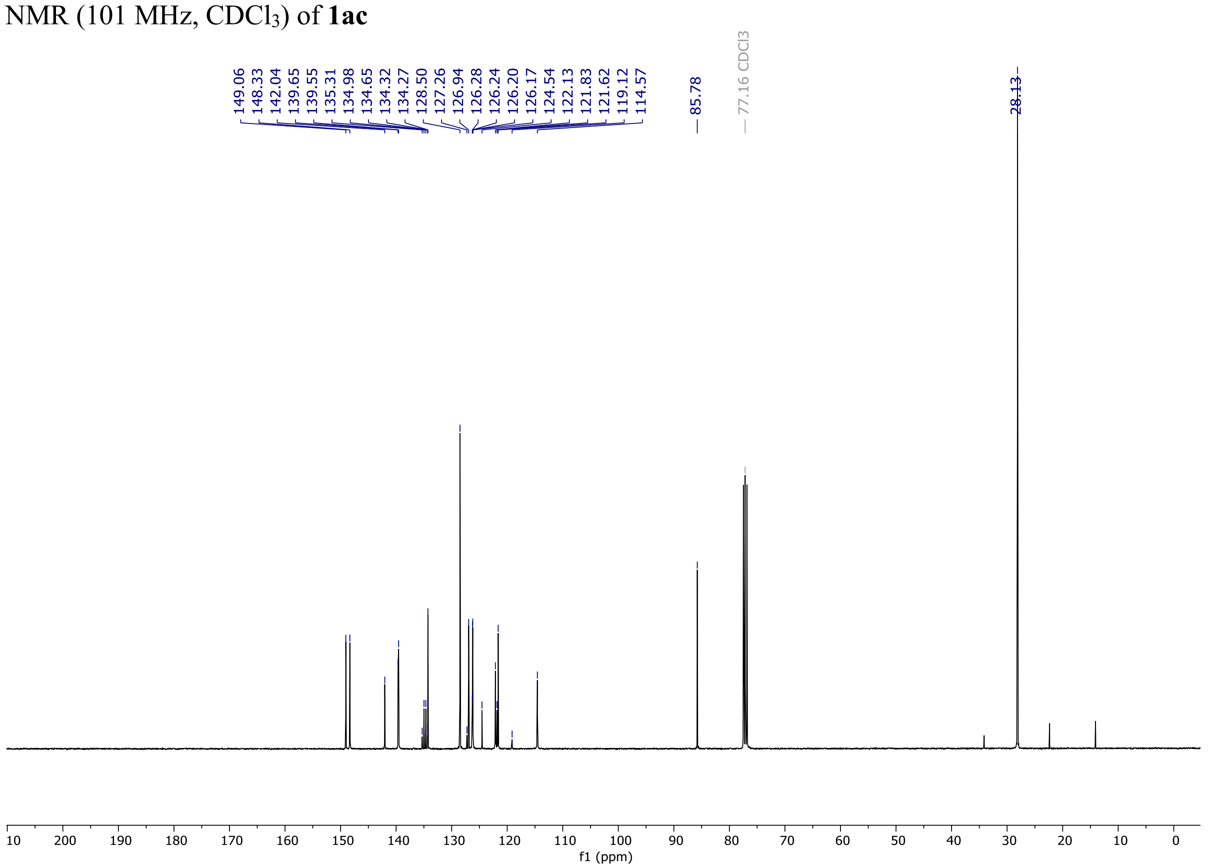

^{19}F NMR (282 MHz, $CDCl_3$) of **1ac**

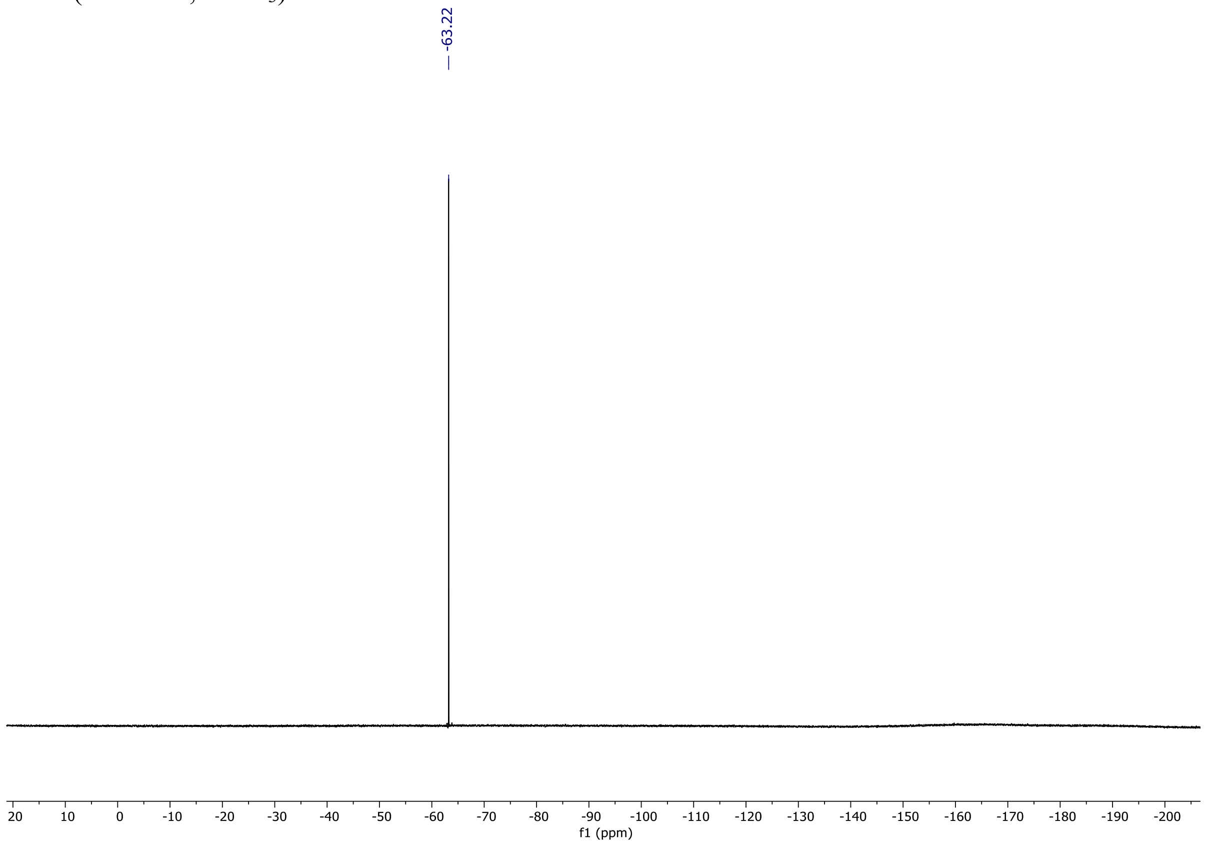

^{1}H NMR (400 MHz, DMSO) of **1ad**

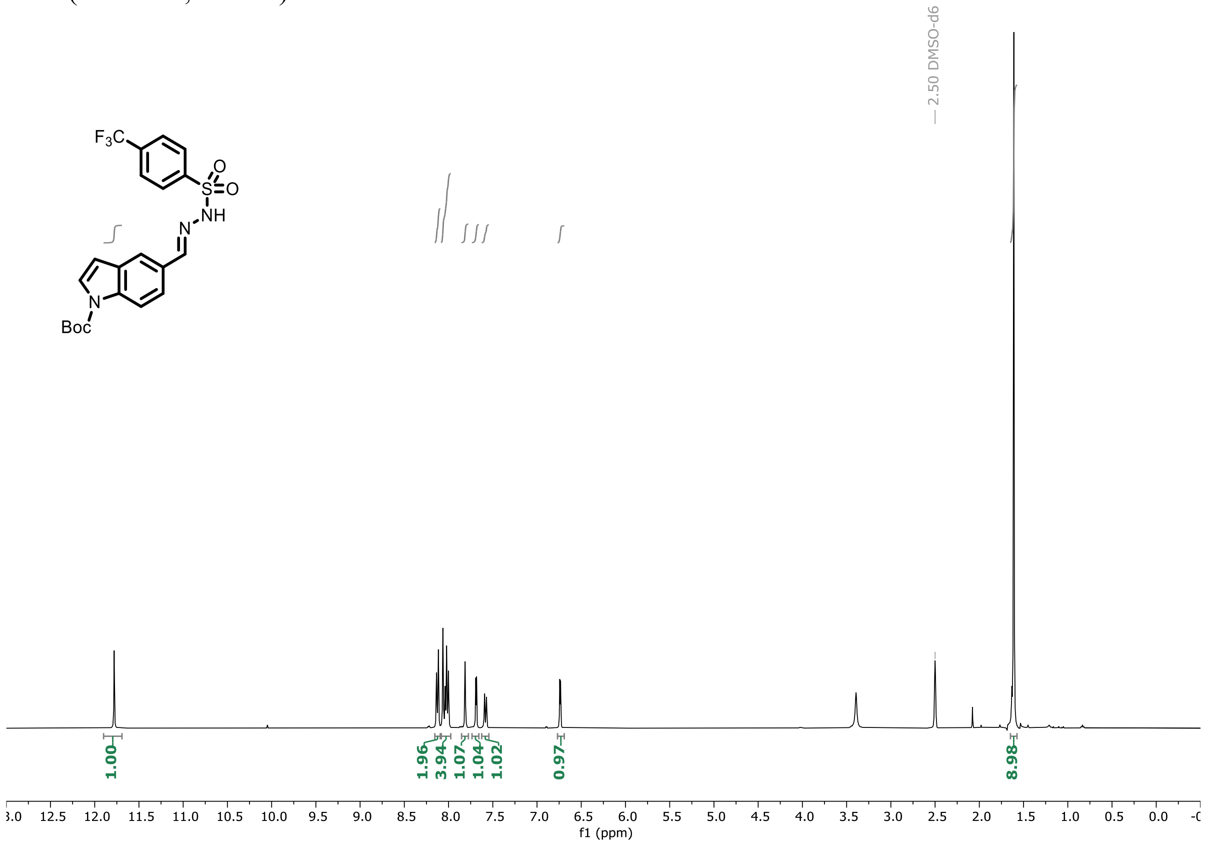

^{13}C NMR (101 MHz, DMSO) of **1ad**

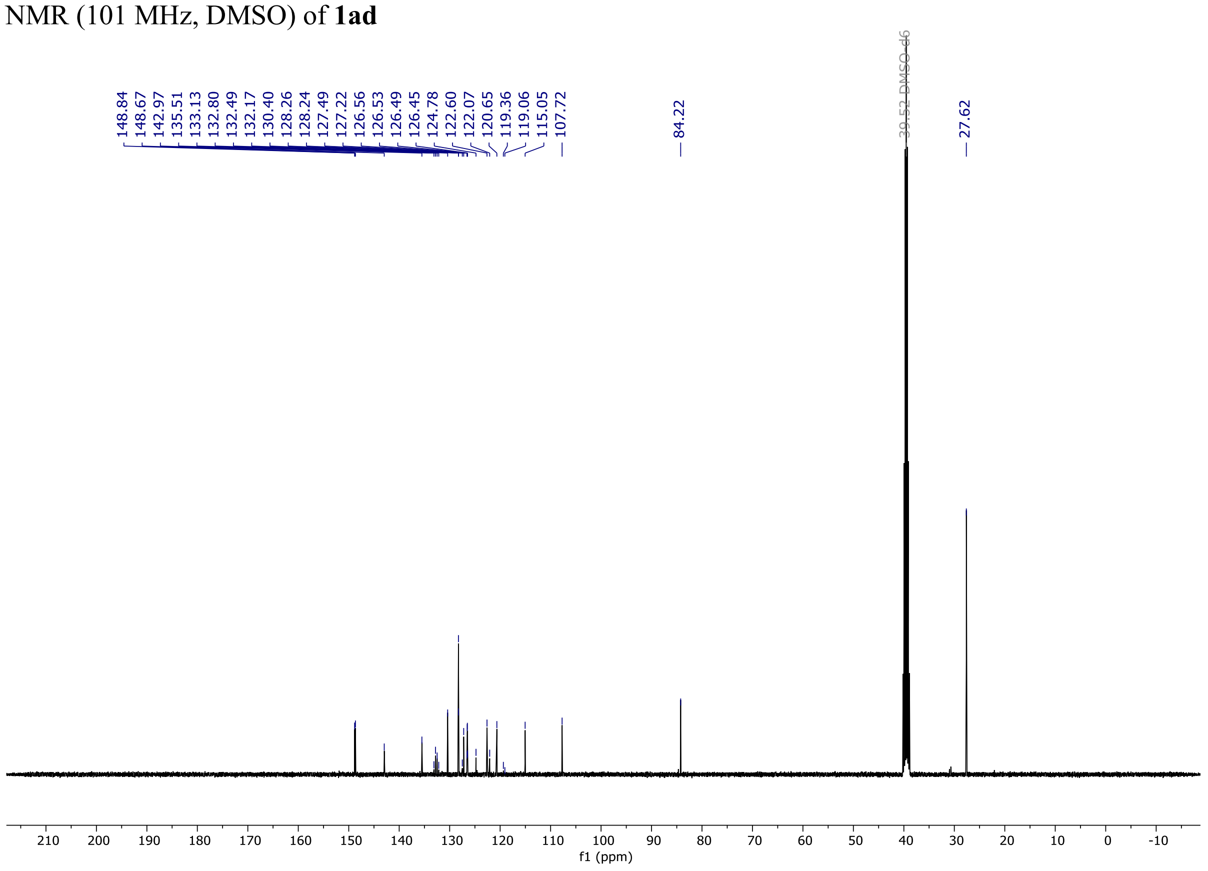

^{19}F NMR (282 MHz, DMSO) of **1ad**

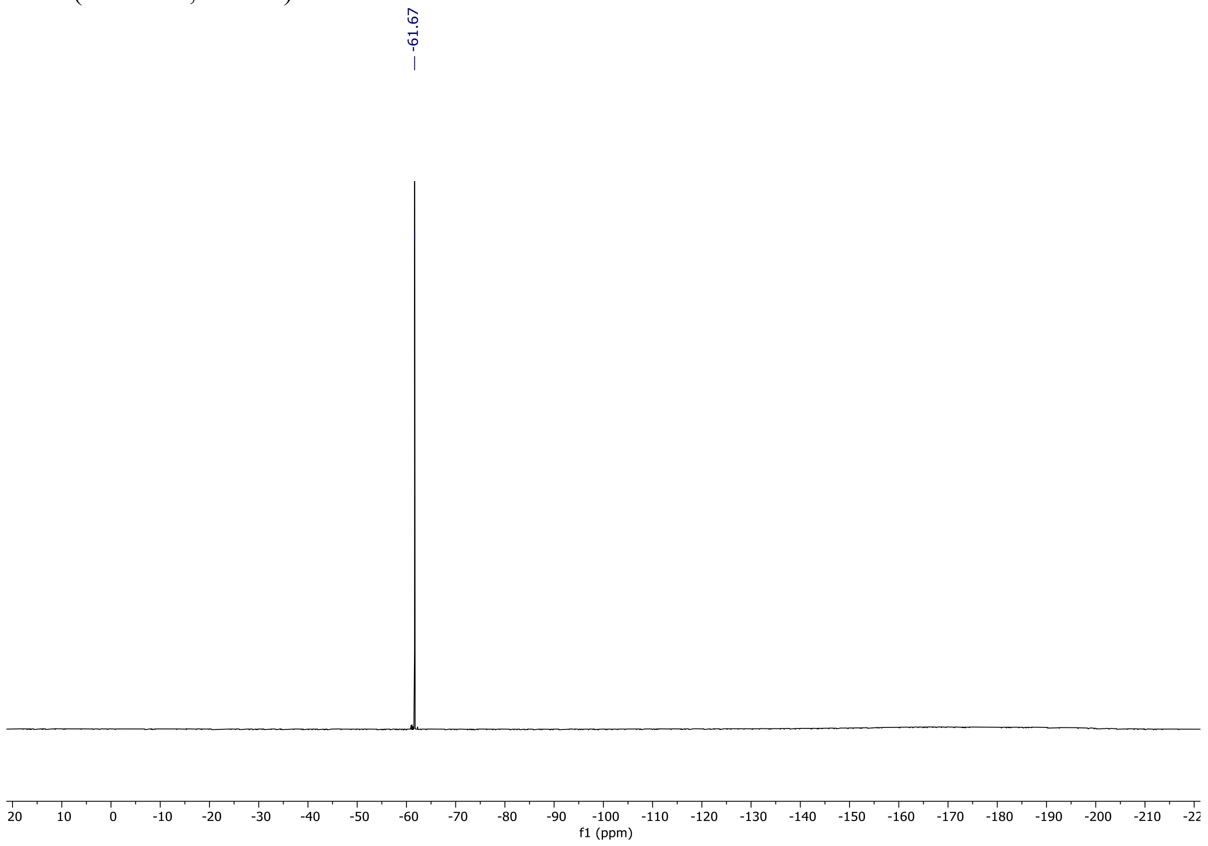

^{1}H NMR (400 MHz, $CDCl_3$) of **1ae**

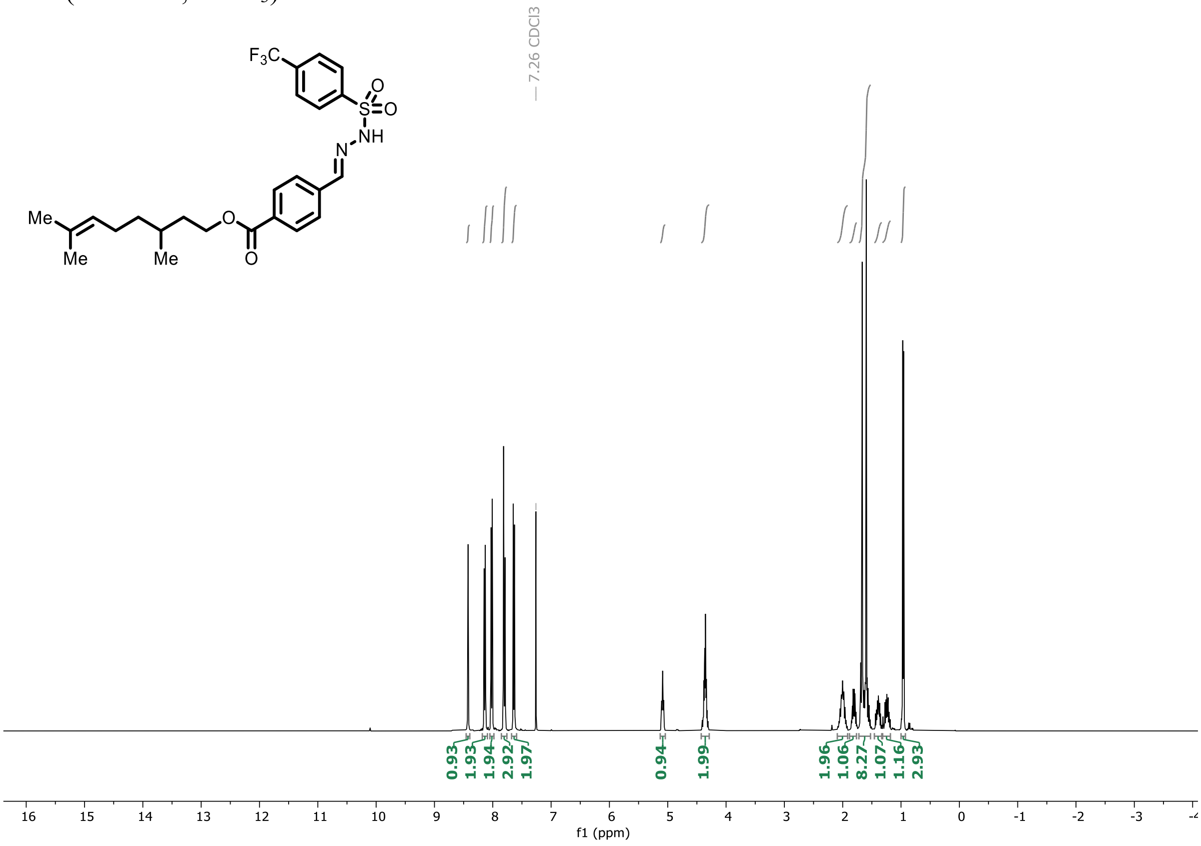

^{13}C NMR (101 MHz, $CDCl_3$) of **1ae**

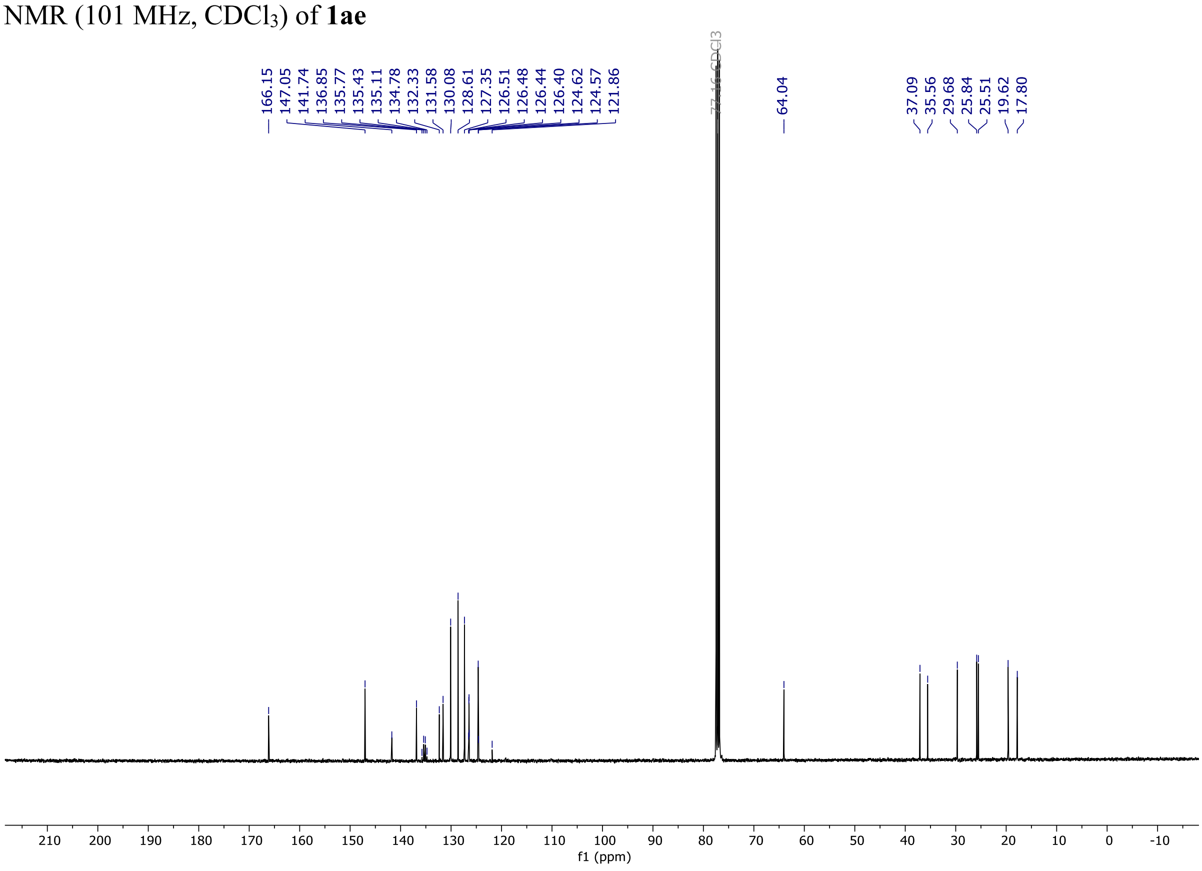

^{19}F NMR (282 MHz, $CDCl_3$) of **1ae**

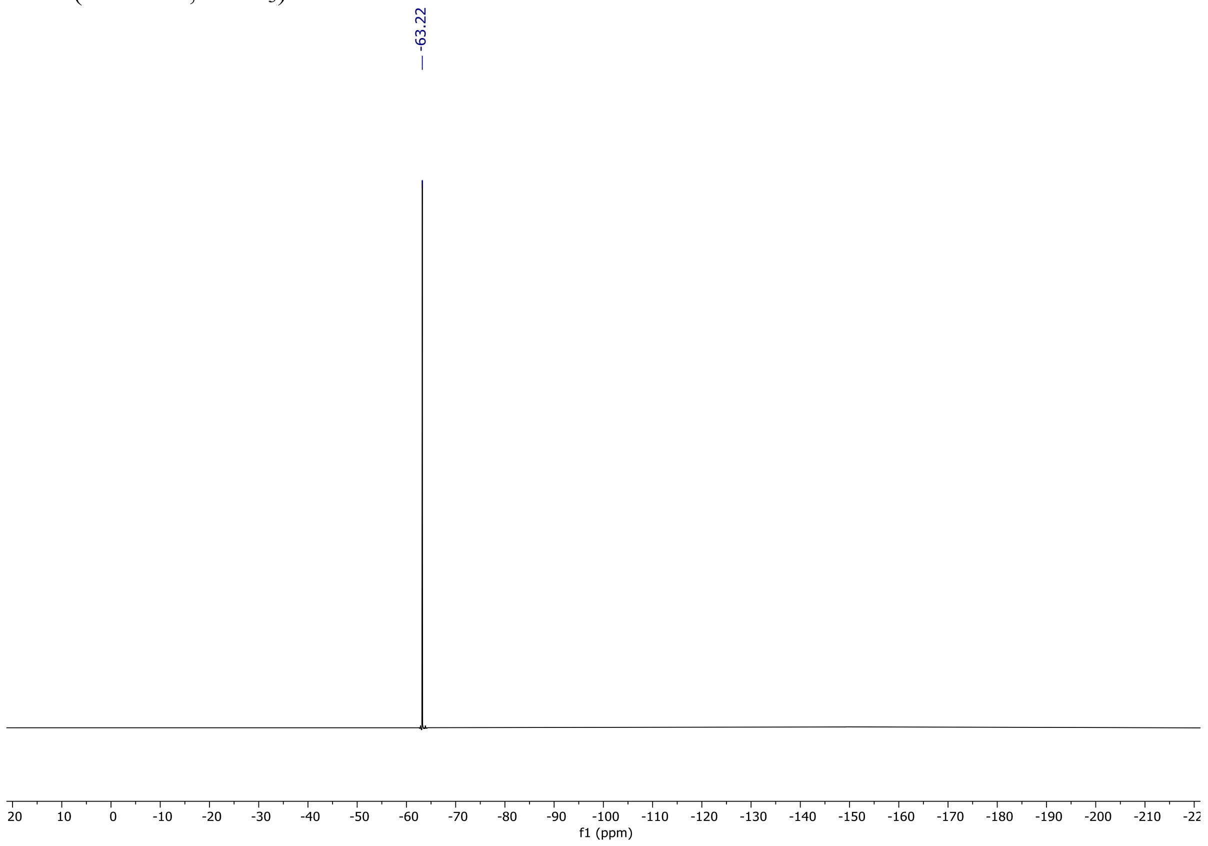

^{1}H NMR (400 MHz, $CDCl_3$) of **1af**

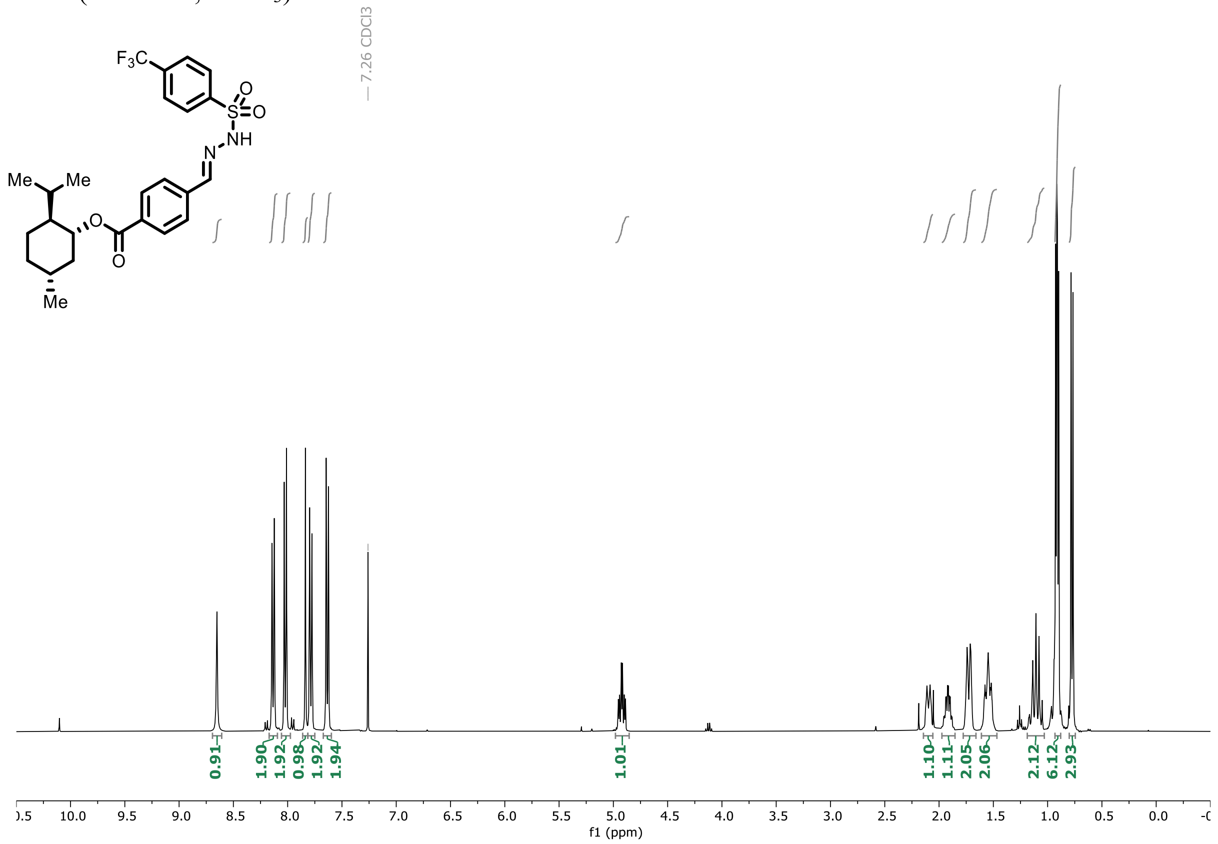

^{13}C NMR (101 MHz, $CDCl_3$) of **1af**

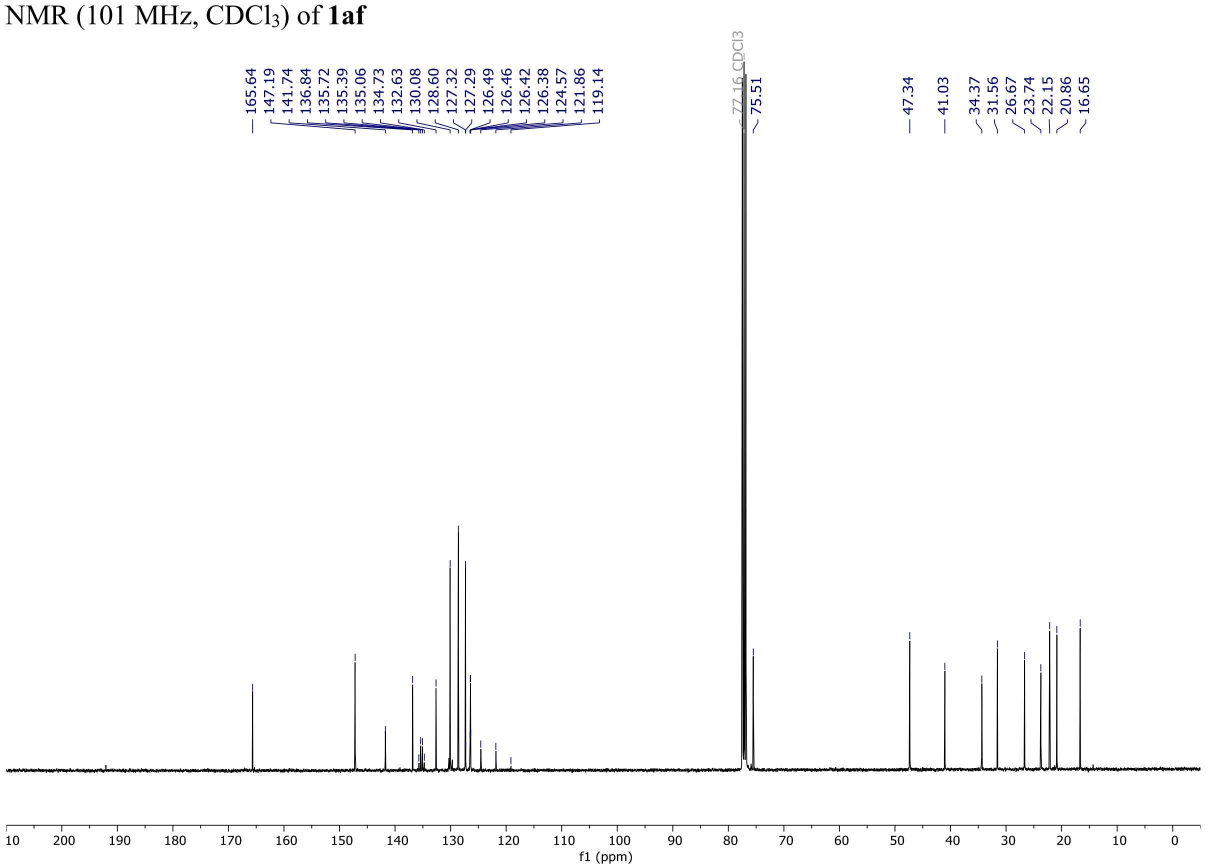

^{19}F NMR (282 MHz, $CDCl_3$) of **1af**

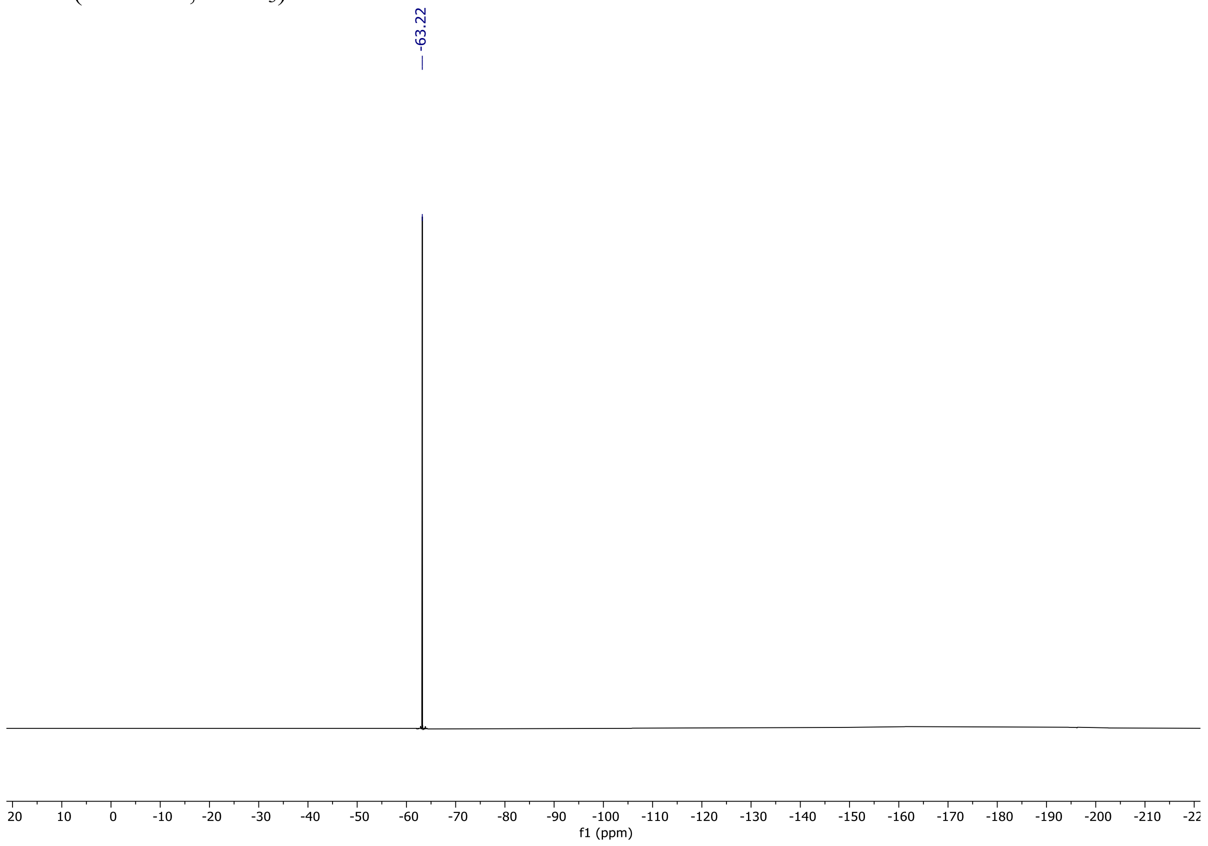

^{1}H NMR (400 MHz, $CDCl_3$) of **1ag**

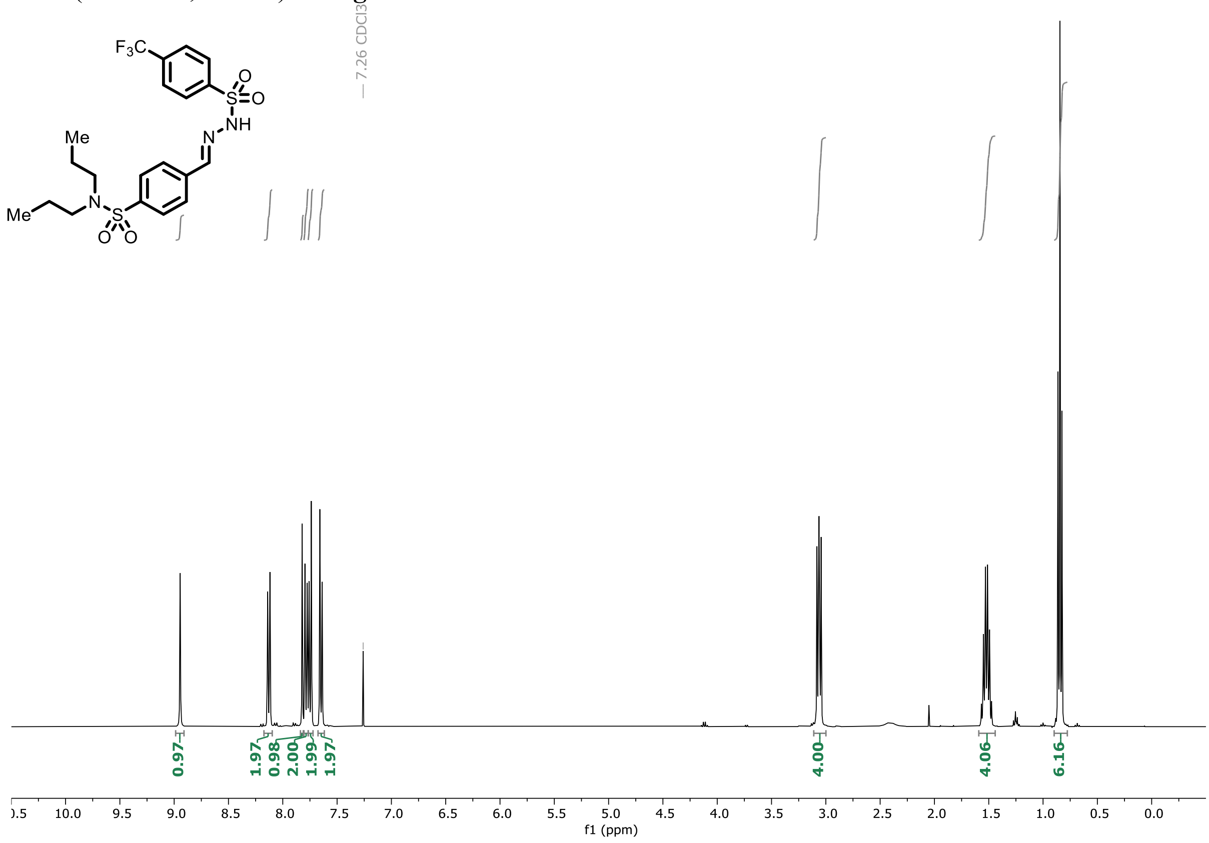

^{13}C NMR (101 MHz, $CDCl_3$) of **1ag**

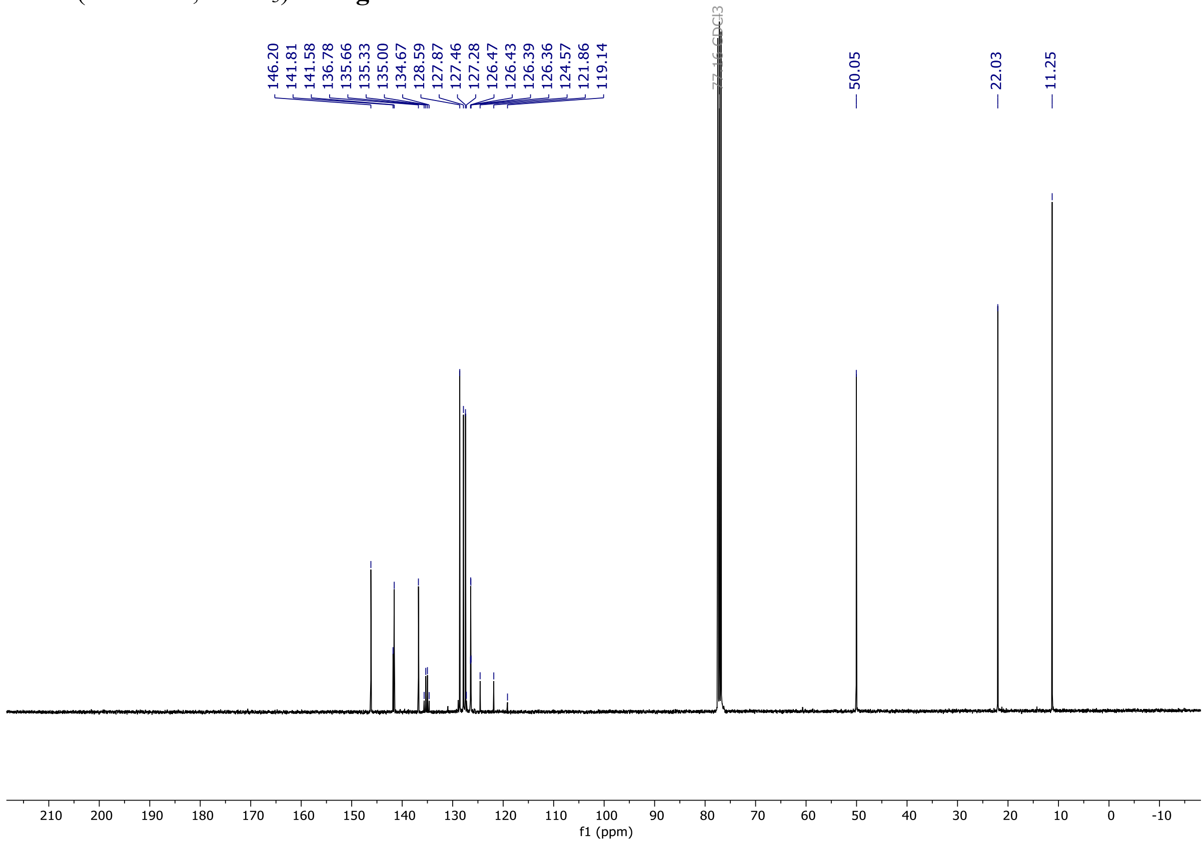

^{19}F NMR (282 MHz, $CDCl_3$) of **1ag**

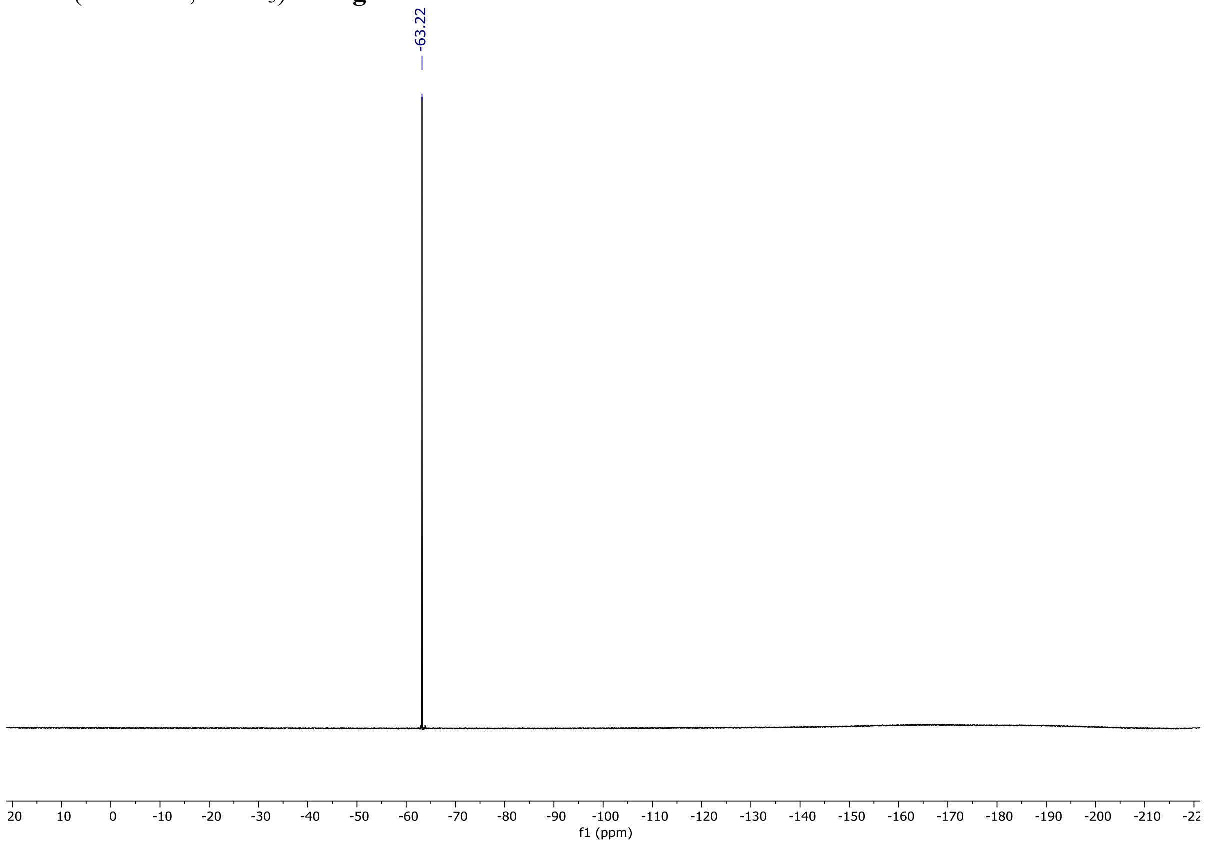

^{1}H NMR (400 MHz,CDCl$_3$) of **1ah**

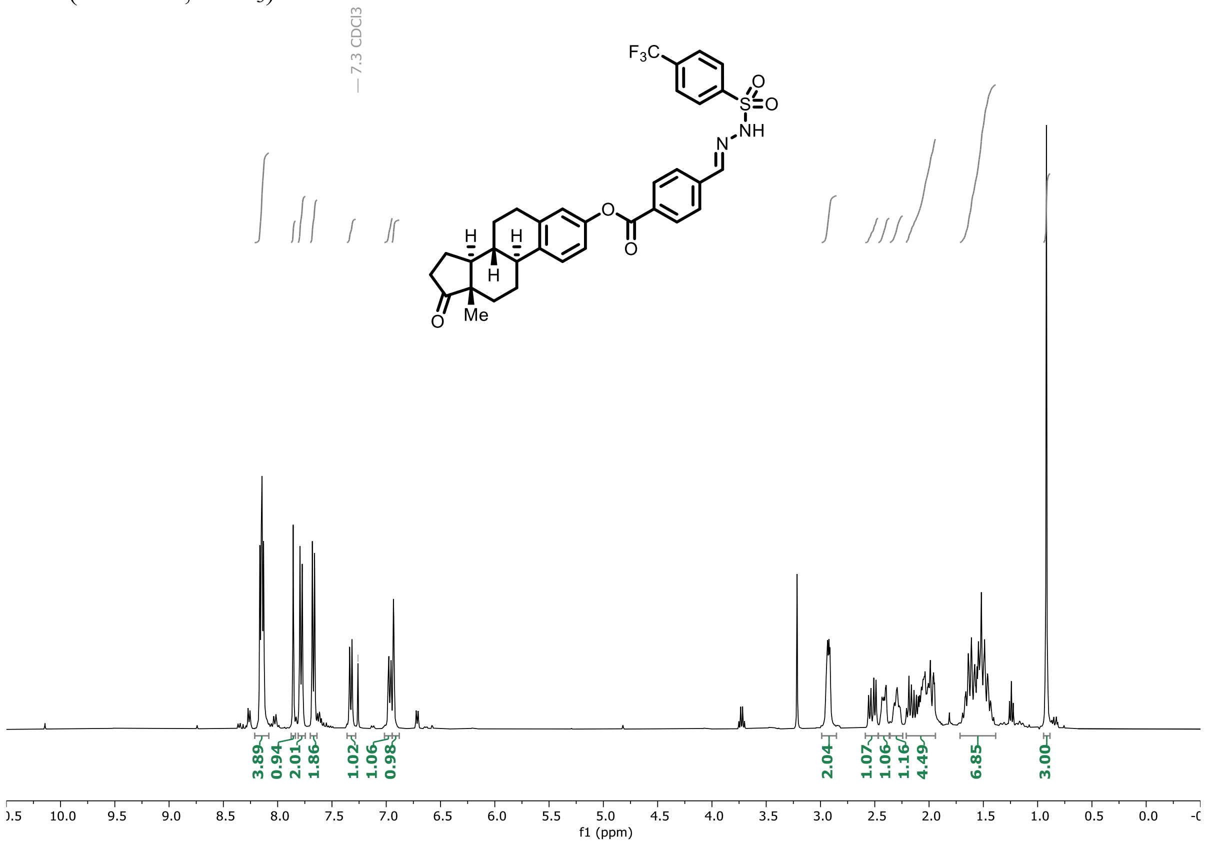

^{13}C NMR (101 MHz, CDCl$_3$) of **1ah**

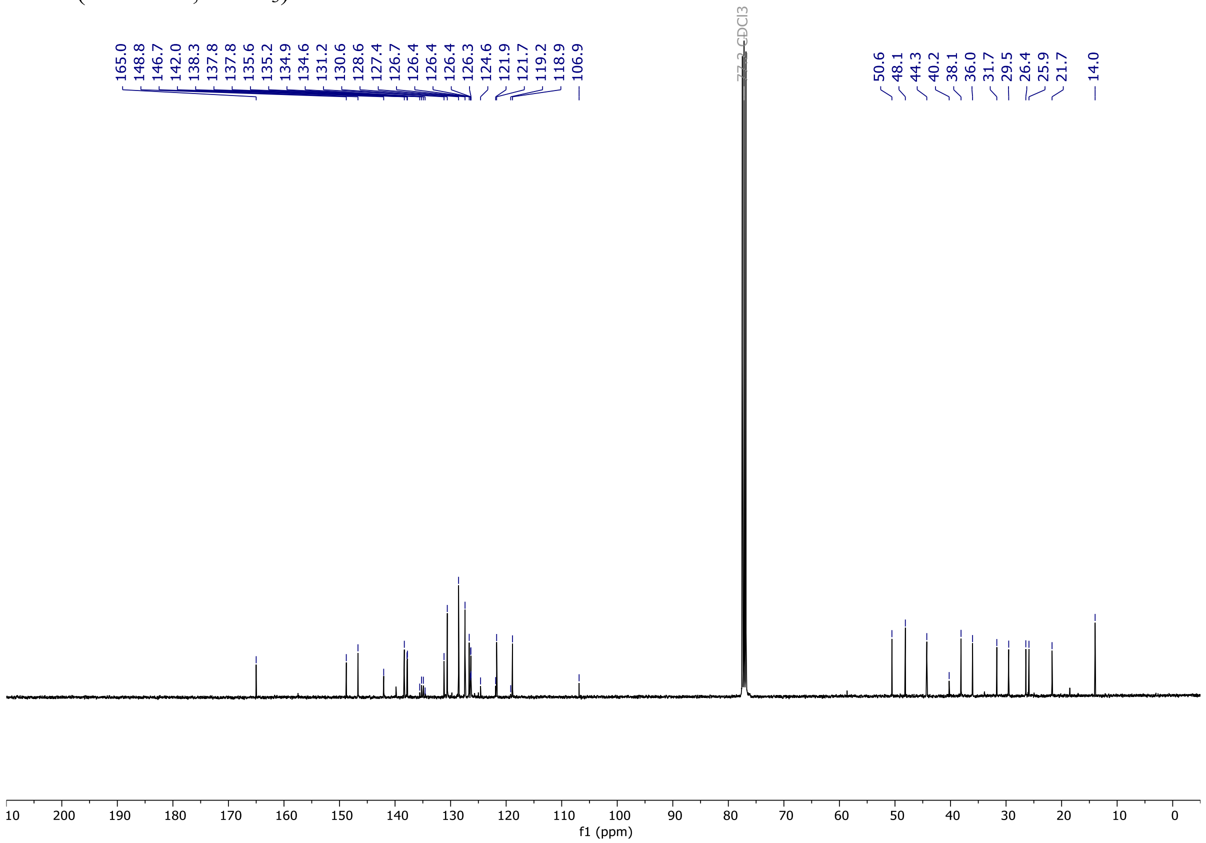

^{19}F NMR (282 MHz, $CDCl_3$) of **1ah**

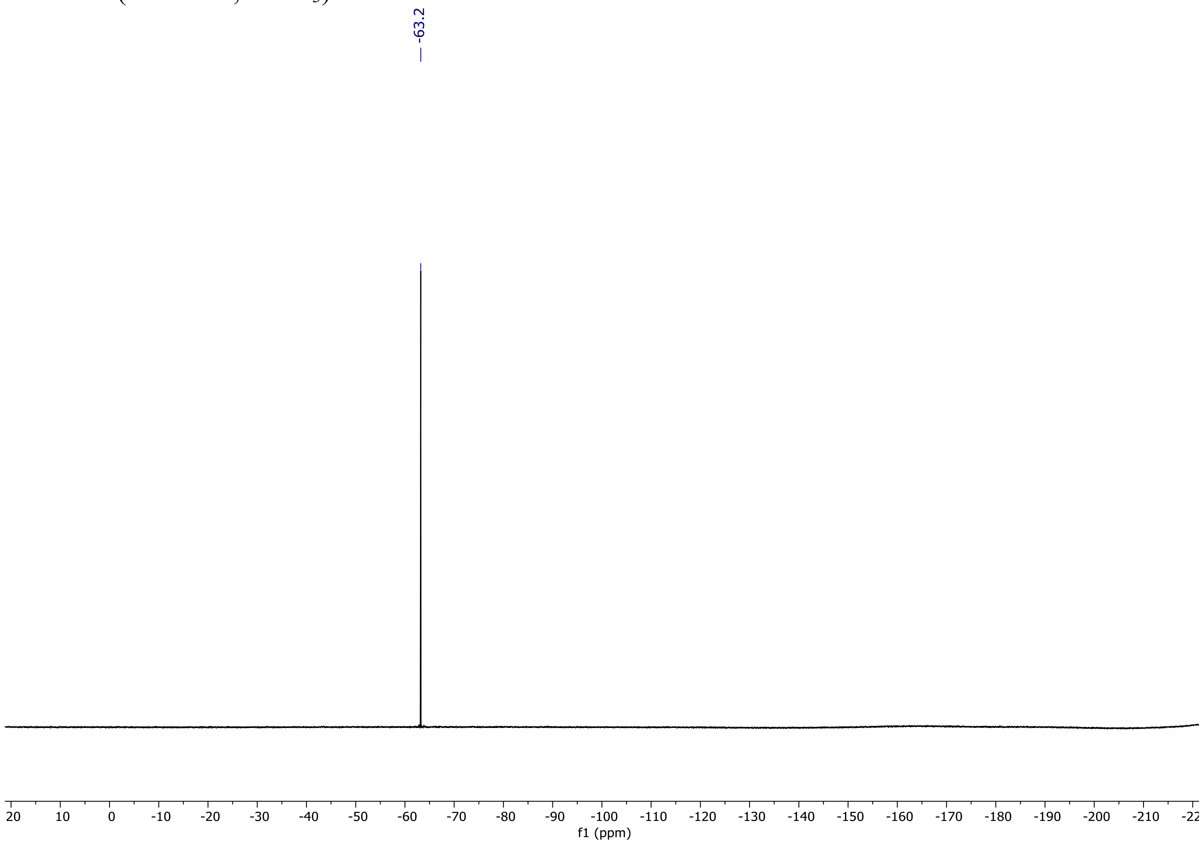

^{1}H NMR (400 MHz, DMSO) of **1ai**

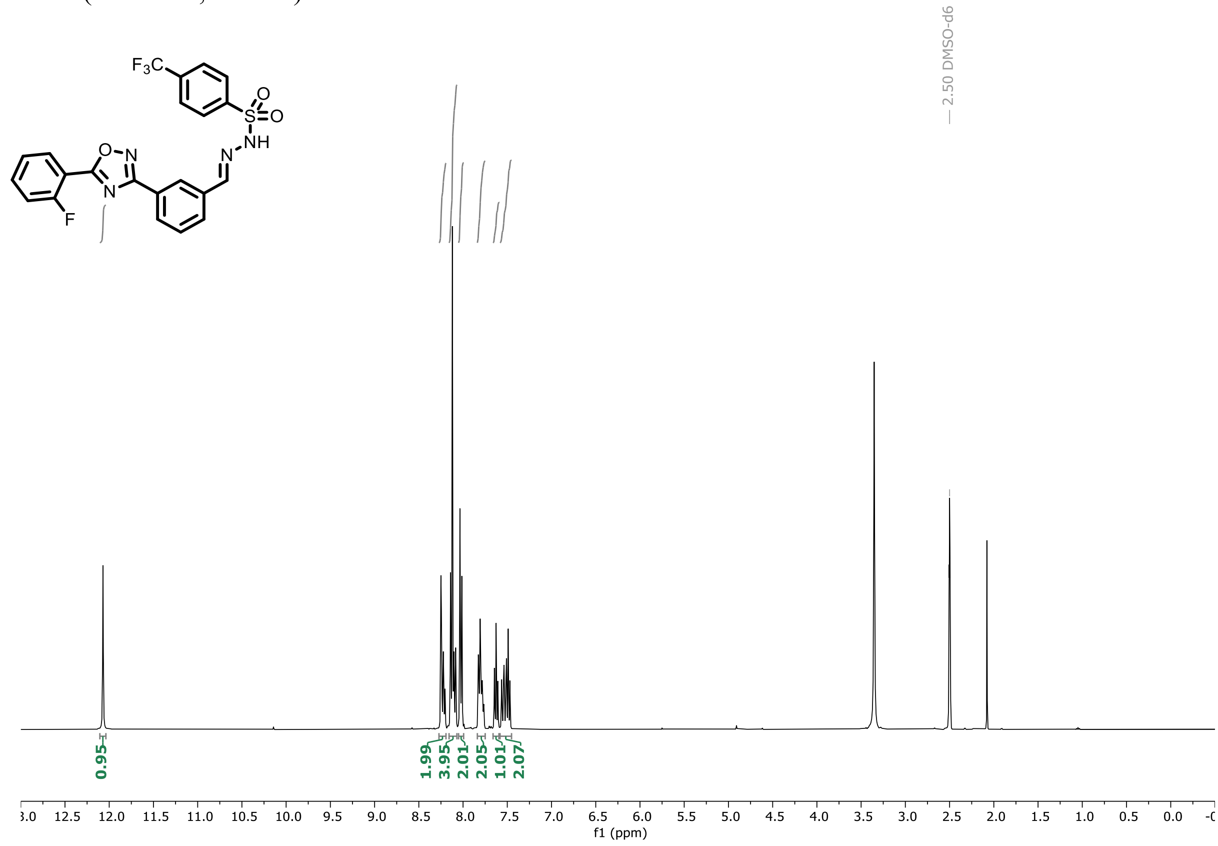

^{13}C NMR (101 MHz, DMSO) of **1ai**

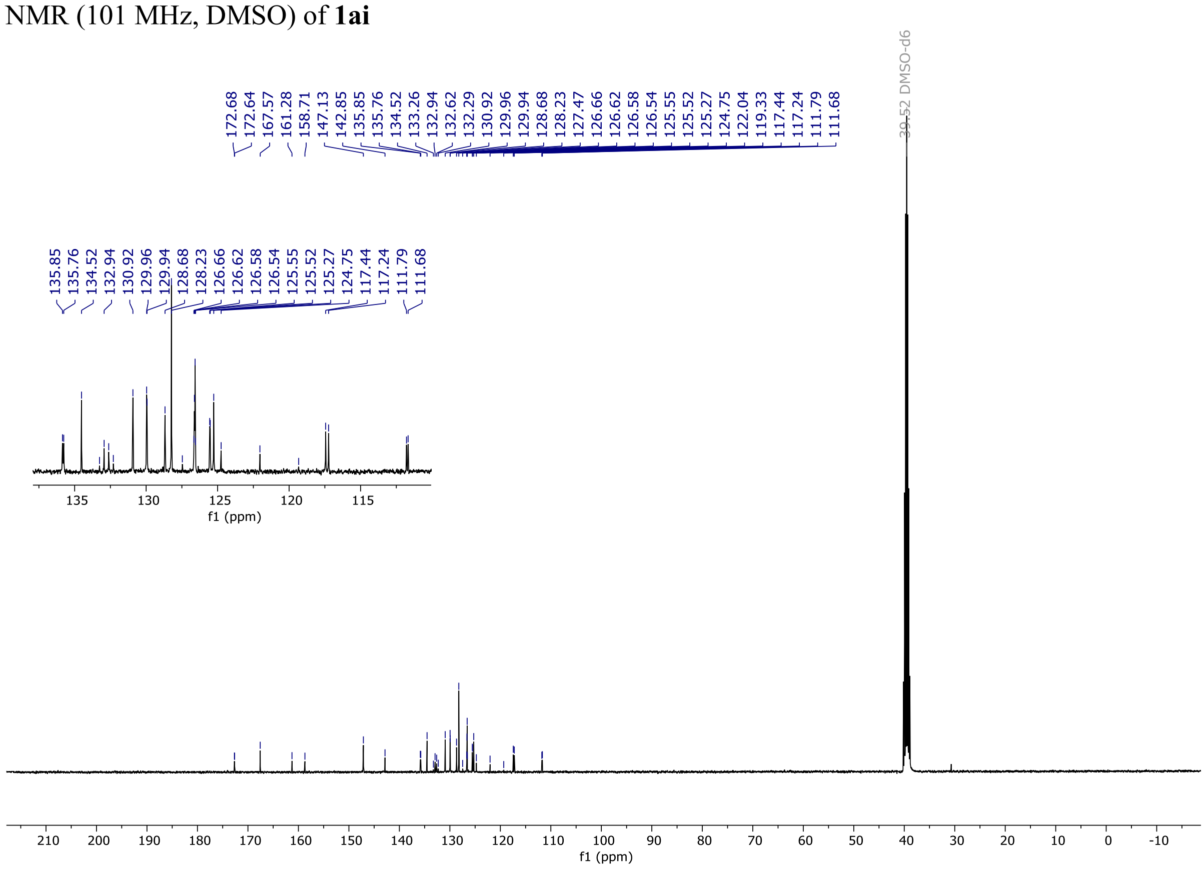

^{19}F NMR (282 MHz, DMSO) of **1ai**

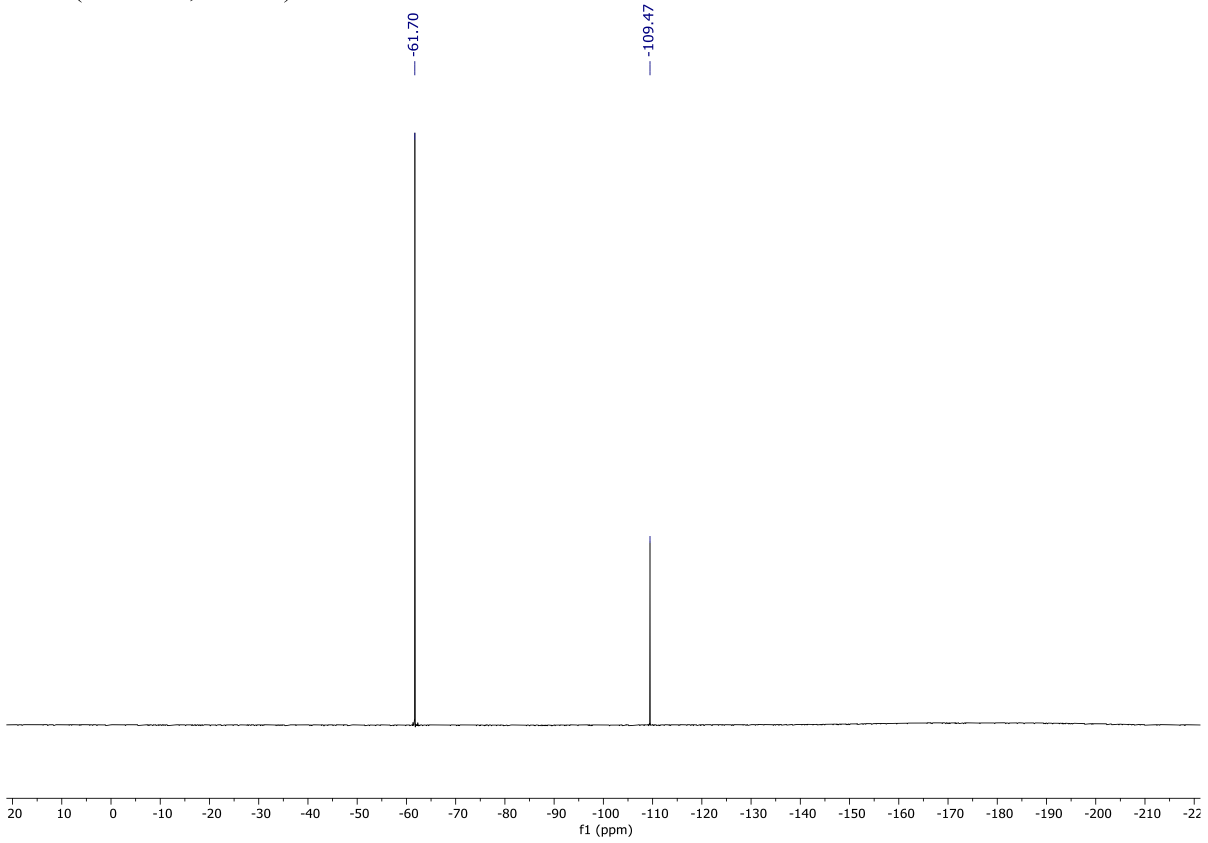

^{1}H NMR (400 MHz, $CDCl_3$) of **1aj**

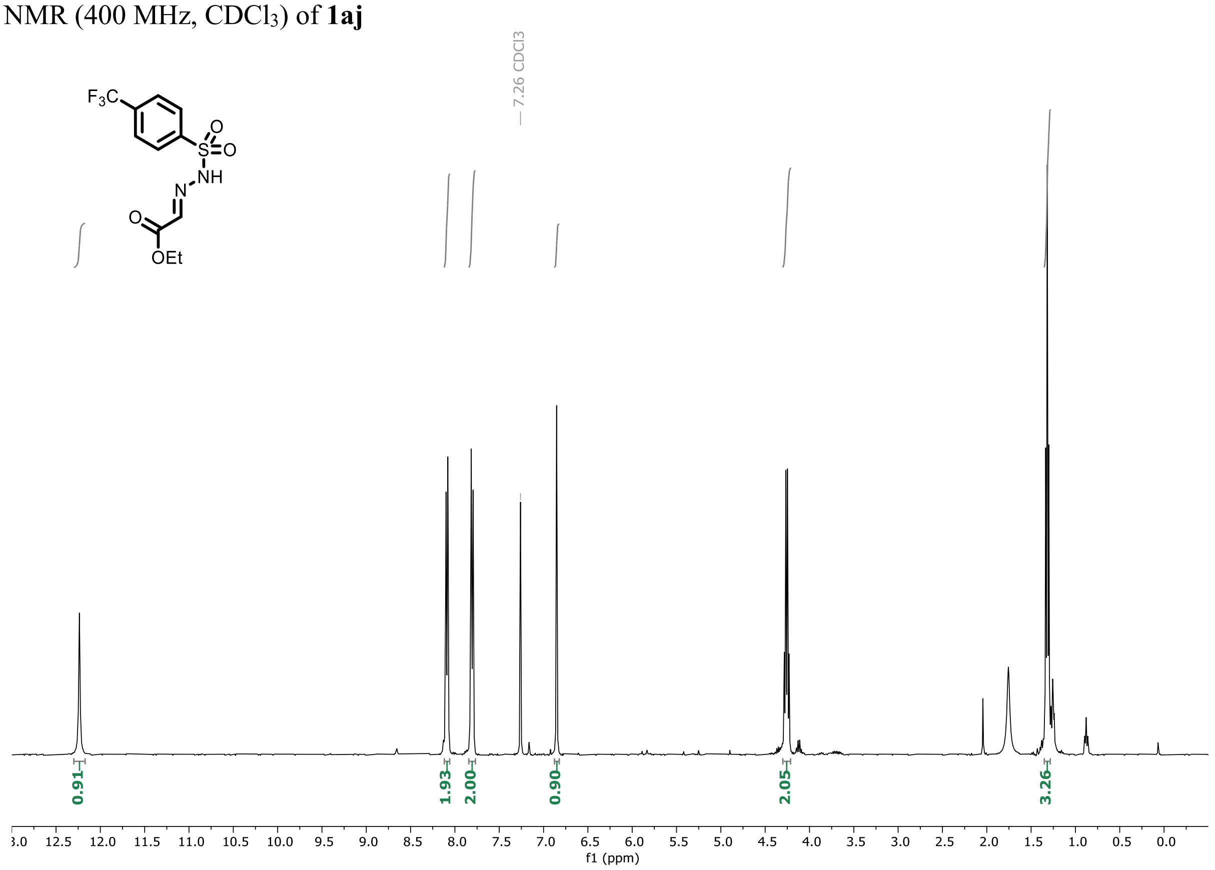

^{13}C NMR (101 MHz, $CDCl_3$) of **1aj**

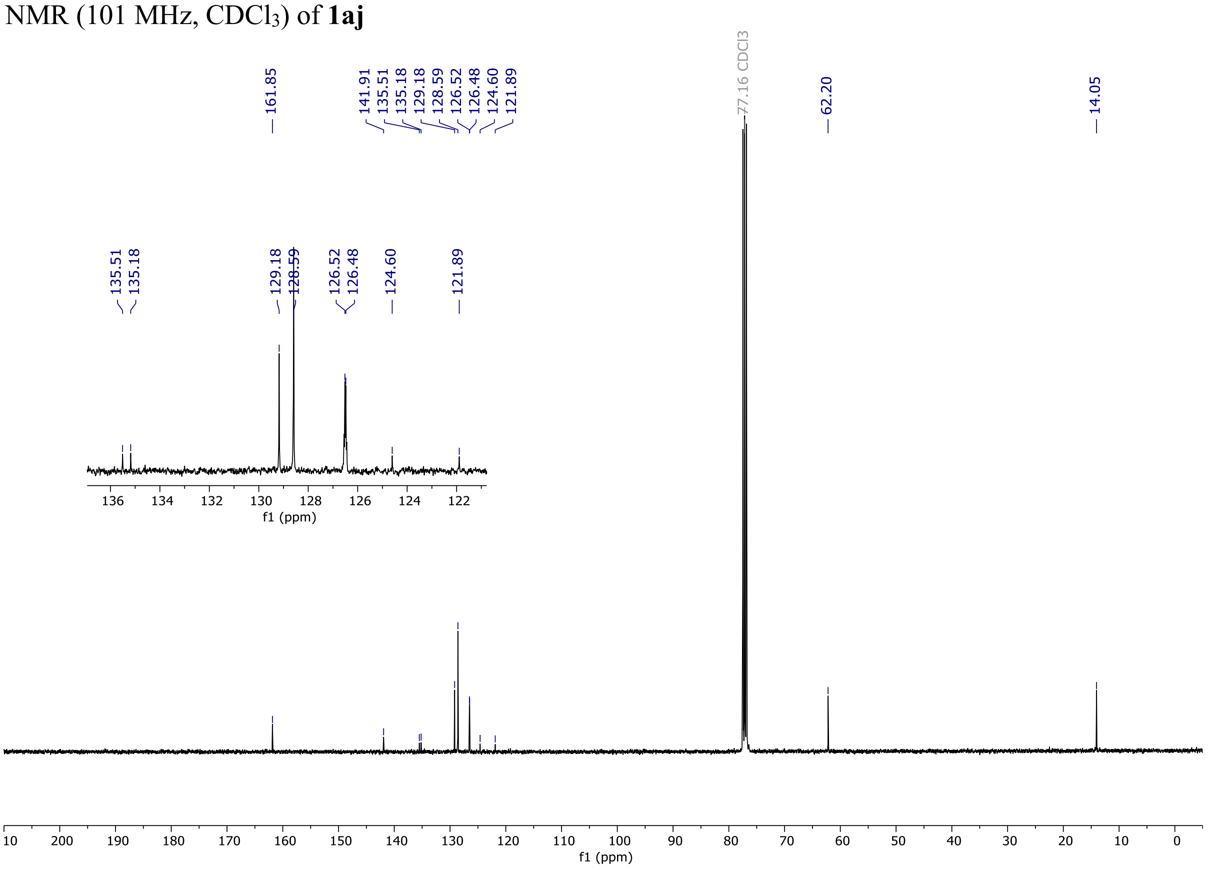

^{19}F NMR (282 MHz, $CDCl_3$) of **1aj**

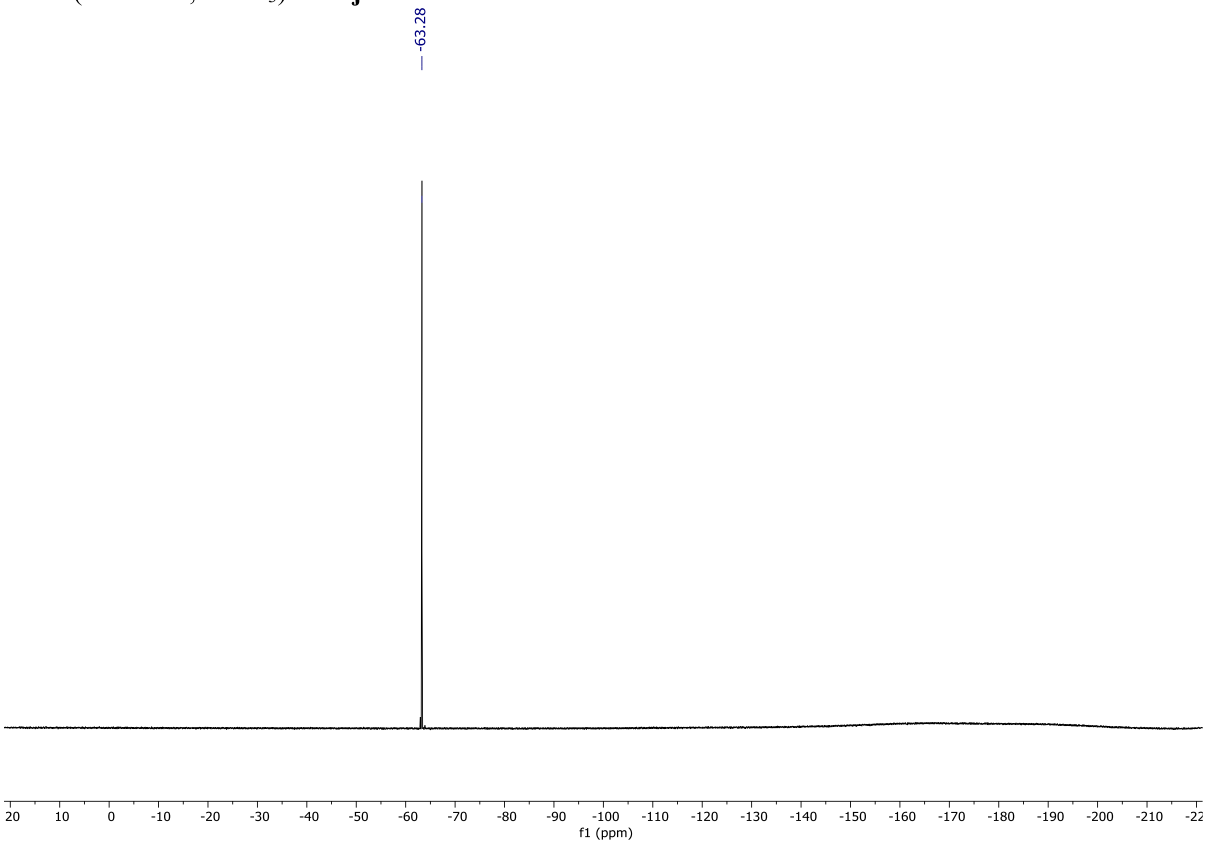

16. **NMR spectra of hydrazide intermediate 2a**

^{1}H NMR (400 MHz, $CDCl_3$) of **2a**

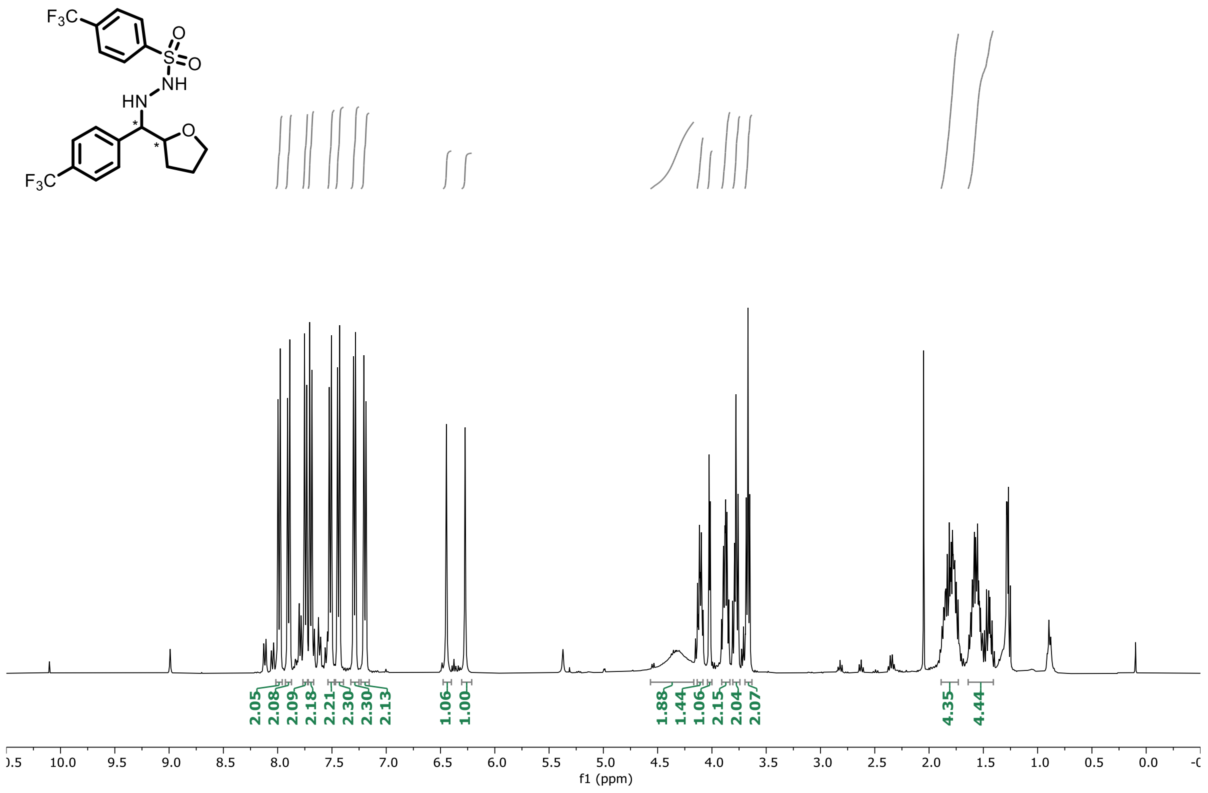

^{19}F NMR (282 MHz, $CDCl_3$) of **2a**

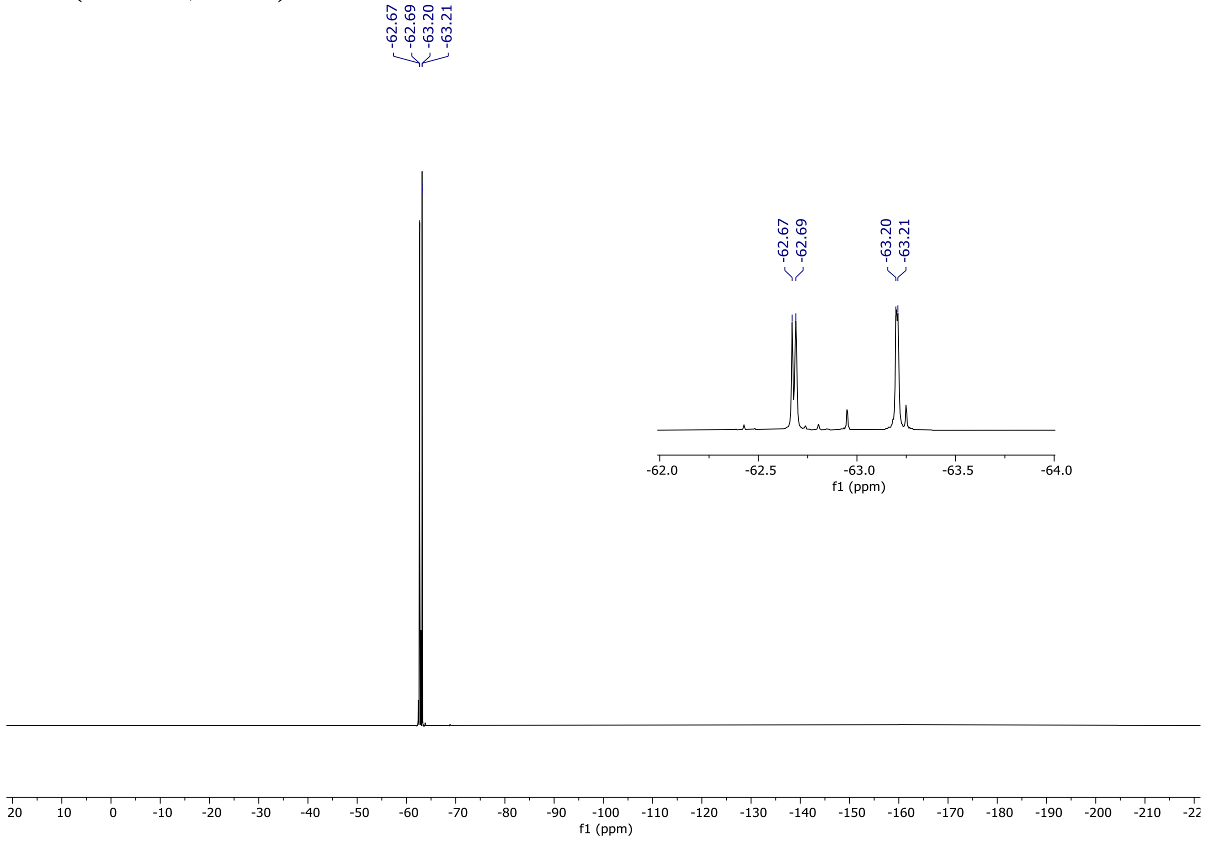

17. **NMR spectra of products 3 – 71**

^{1}H NMR (400 MHz, $CDCl_3$) of **3**

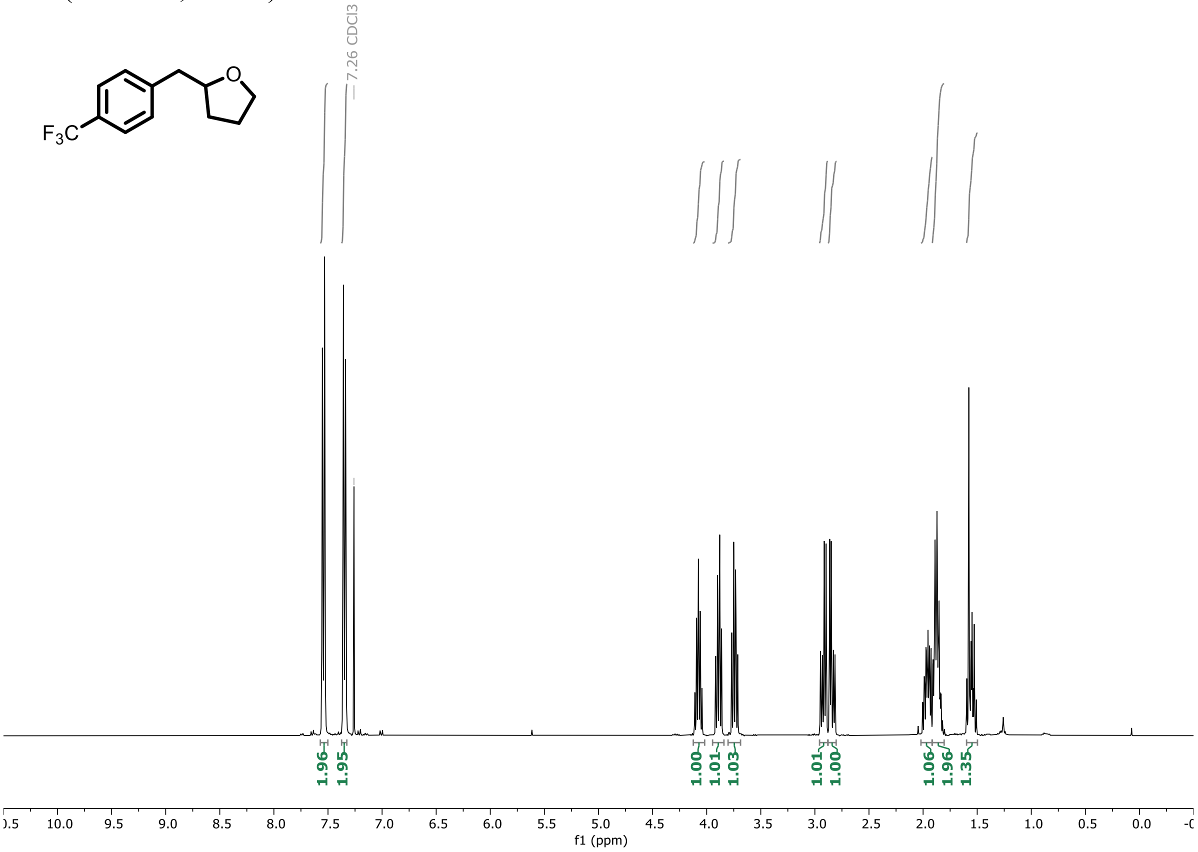

^{13}C NMR (101 MHz, $CDCl_3$) of **3**

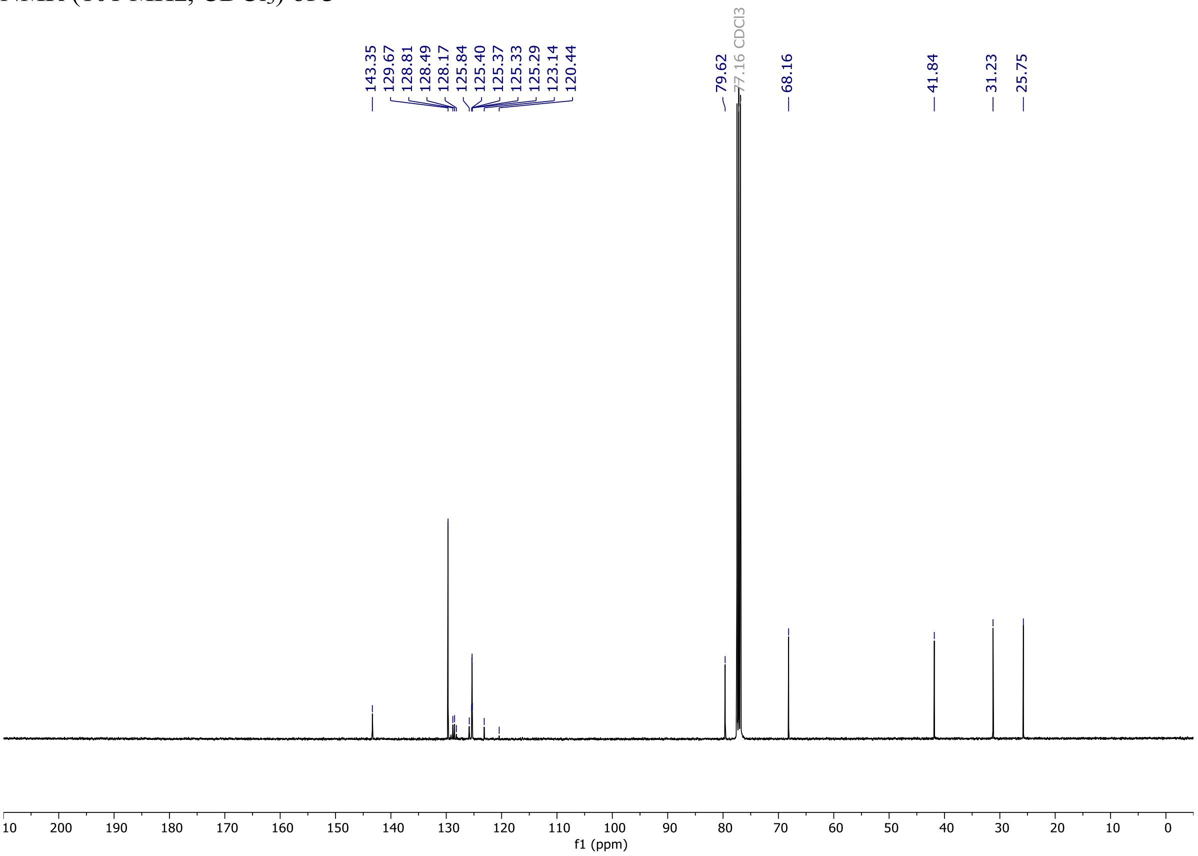

^{19}F NMR (282 MHz, $CDCl_3$) of **3**

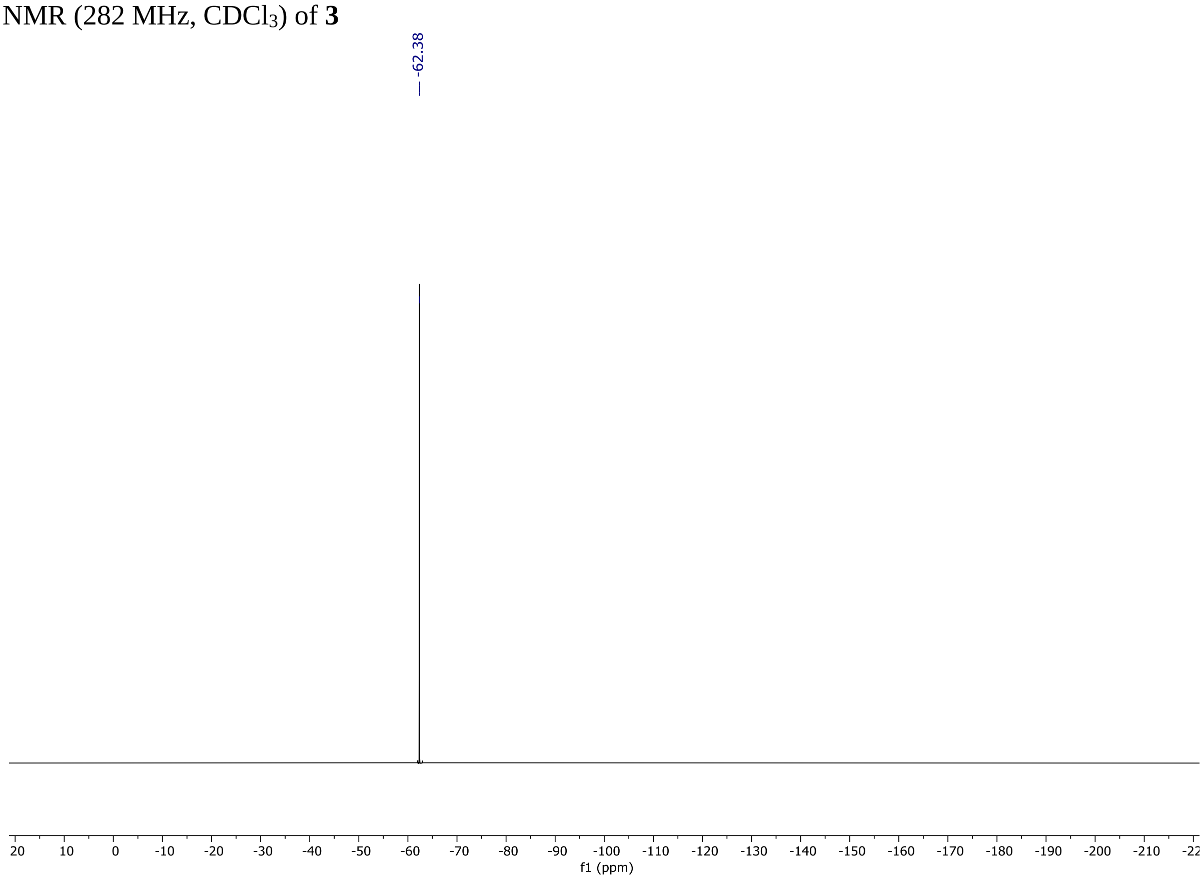

^{1}H NMR (400 MHz, $CDCl_3$) of **4**

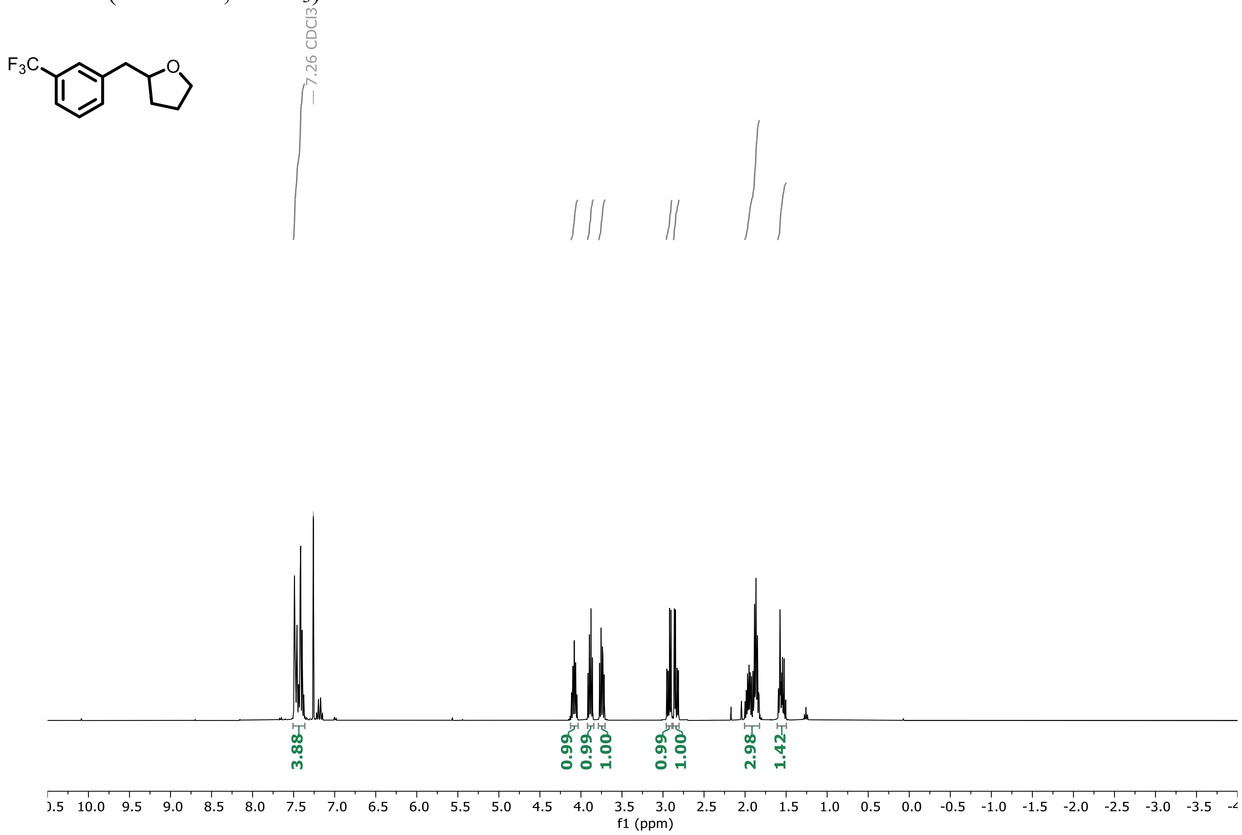

^{13}C NMR (101 MHz, $CDCl_3$) of **4**

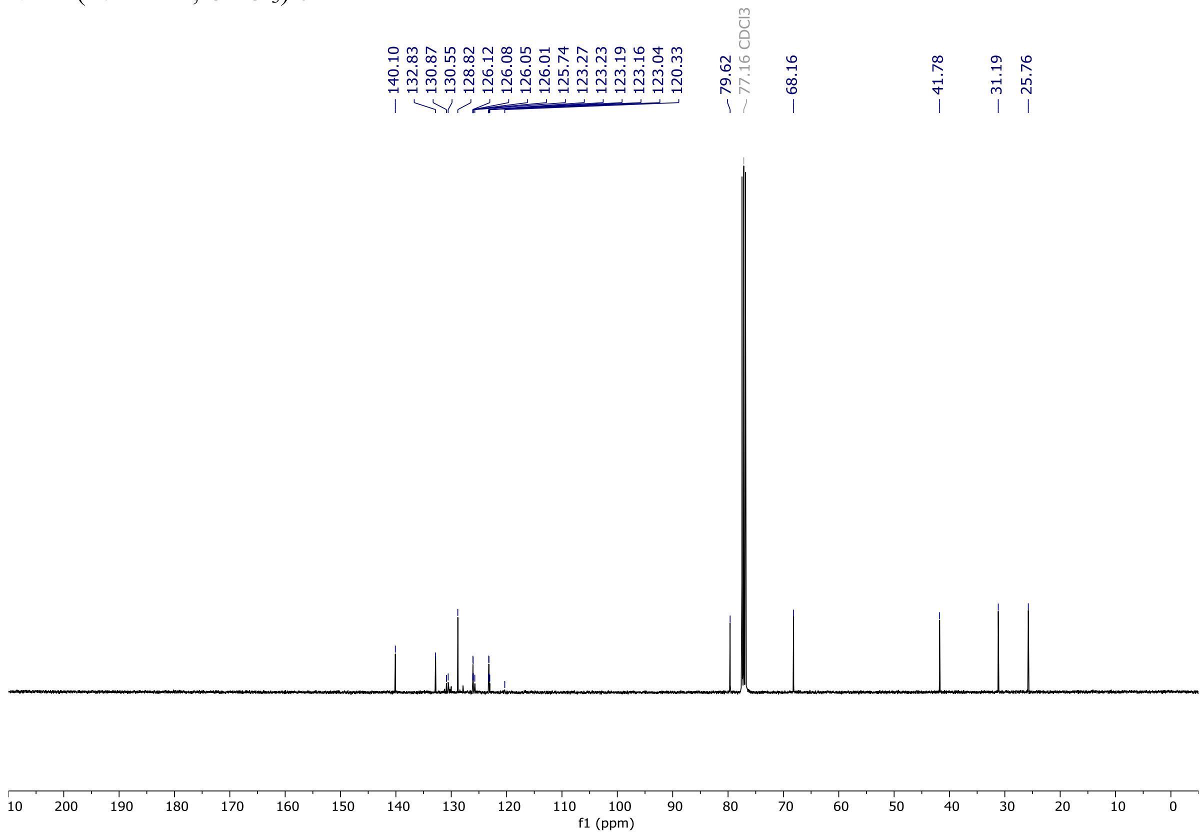

^{19}F NMR (282 MHz, $CDCl_3$) of **4**

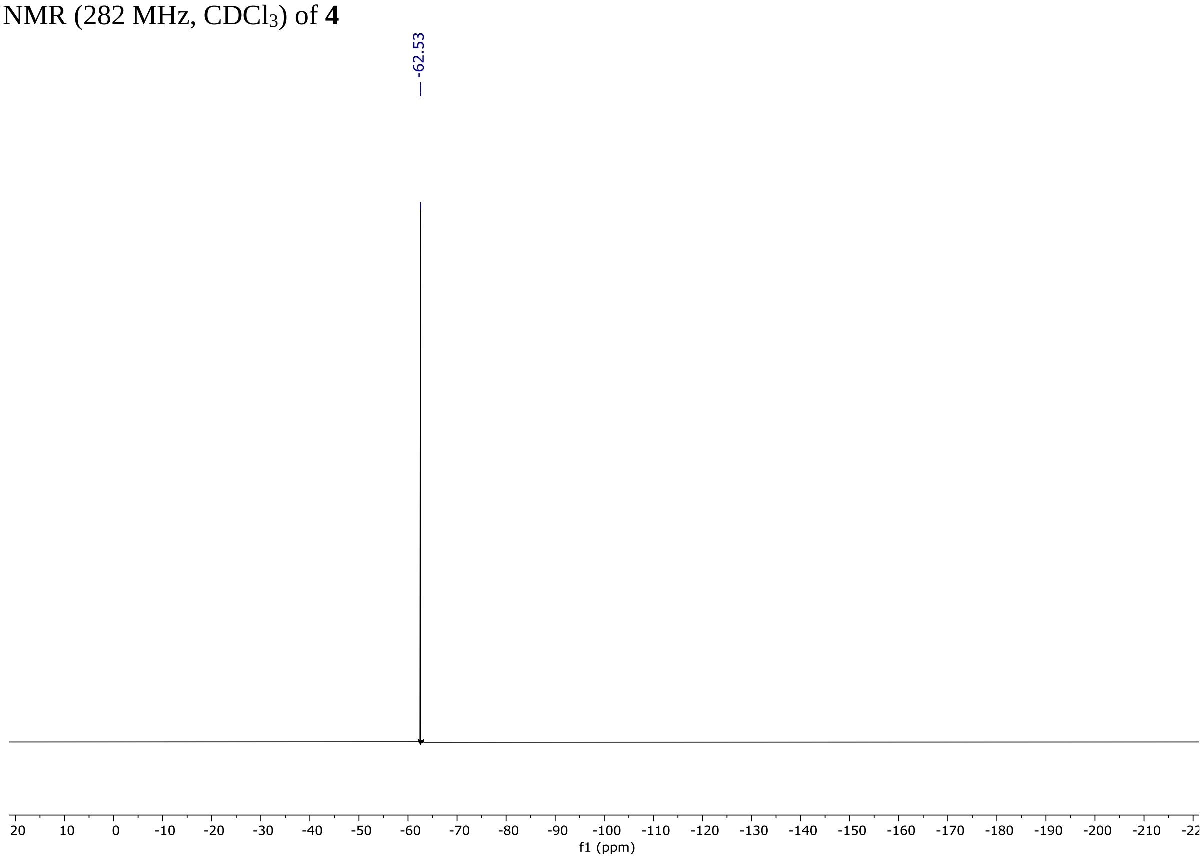

^{1}H NMR (400 MHz,CDCl3) of **5**

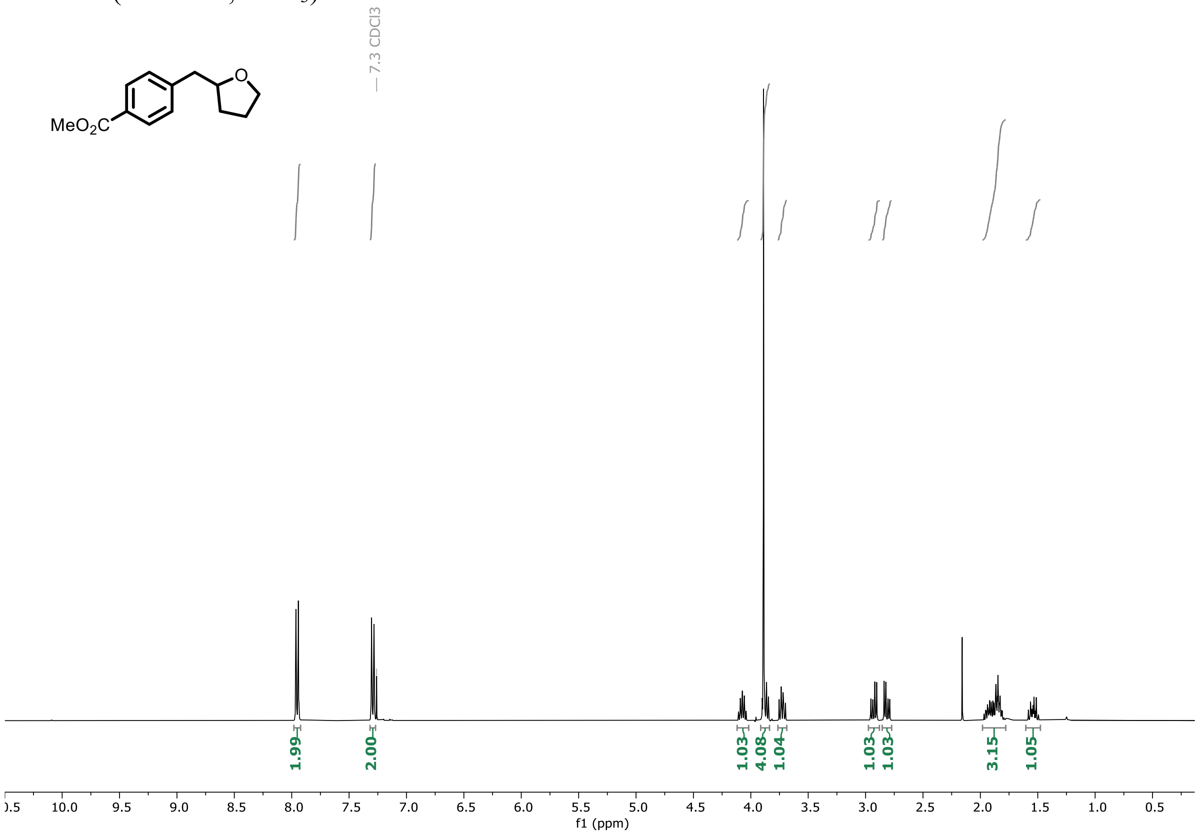

^{13}C NMR (101 MHz, CDCl3) of **5**

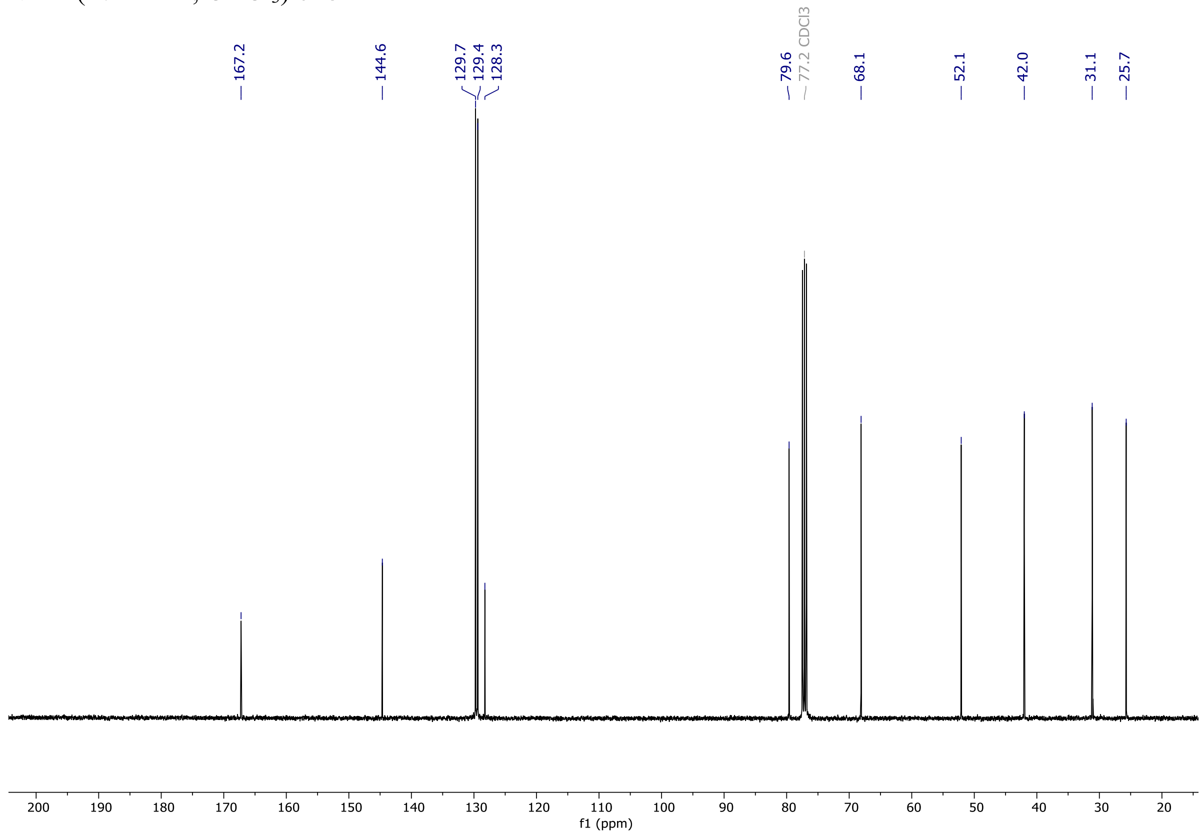

^{1}H NMR (400 MHz,$CDCl_3$) of **6**

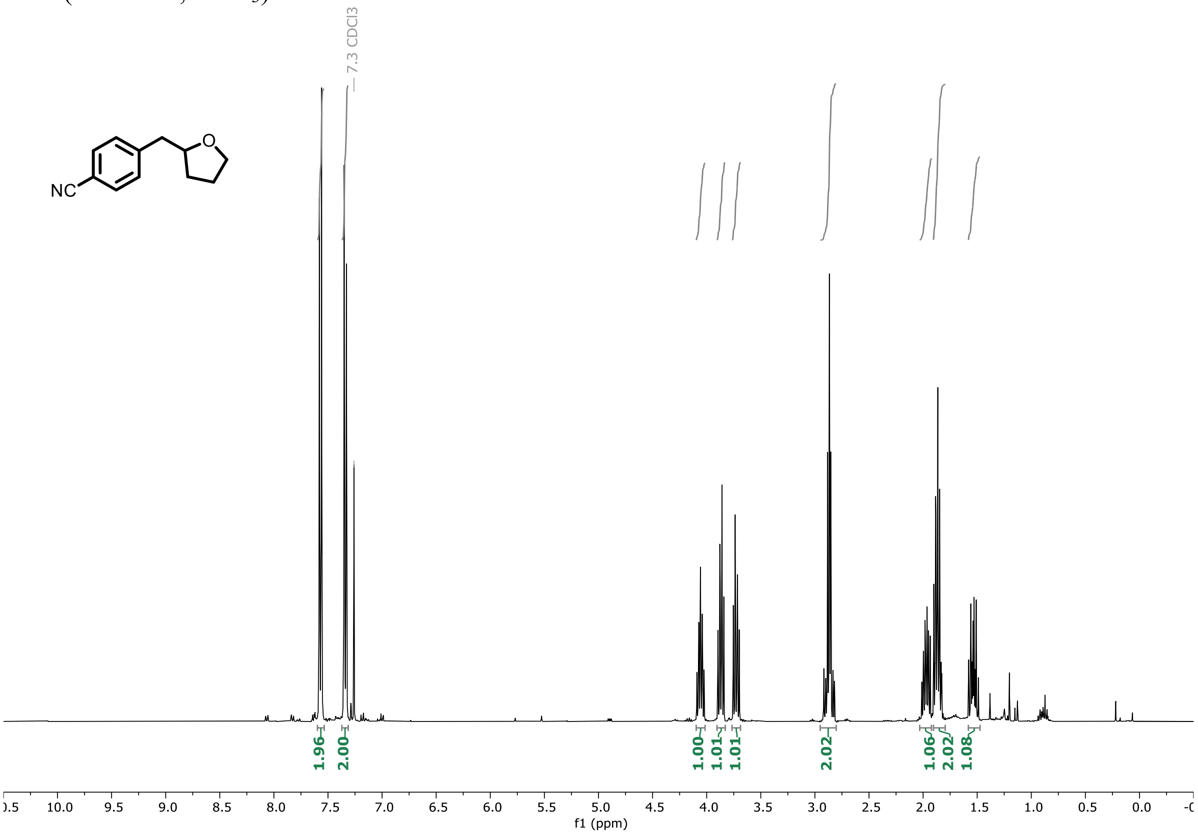

^{13}C NMR (101 MHz, $CDCl_3$) of **6**

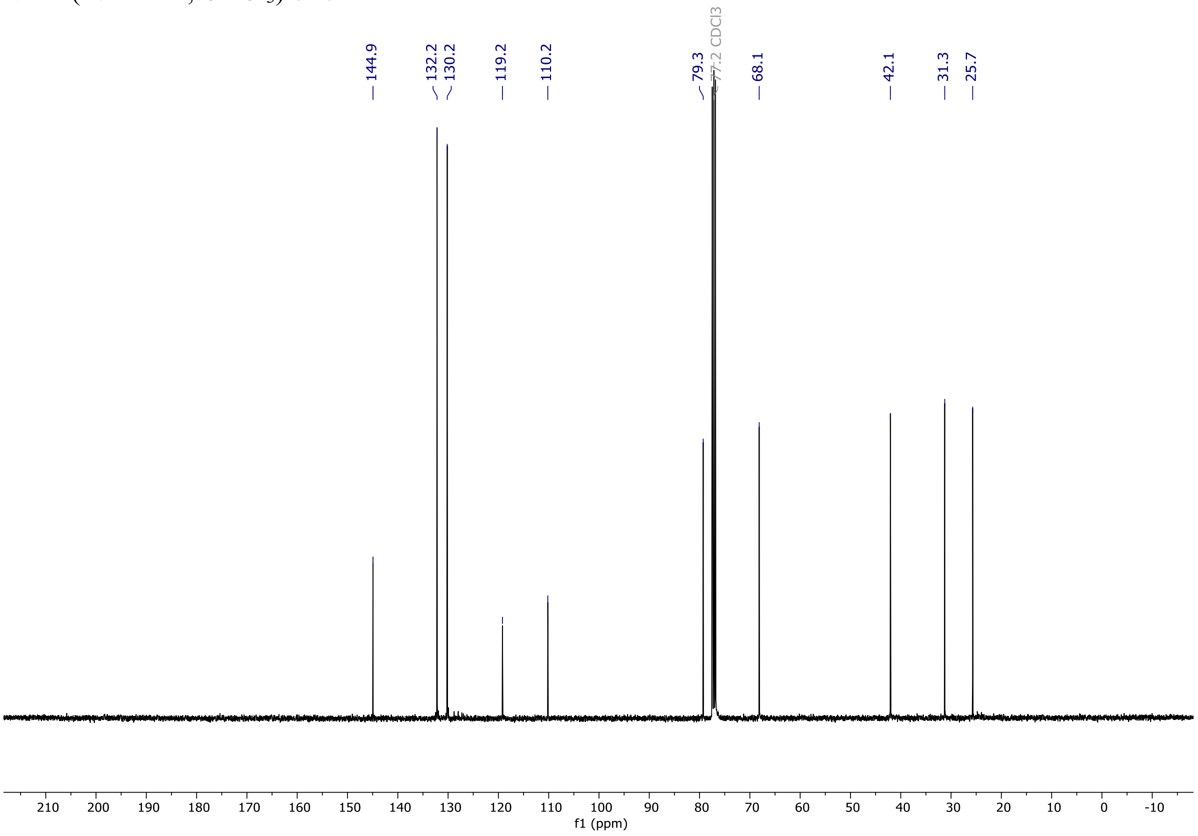

^{1}H NMR (300 MHz,CDCl$_3$) of **7**

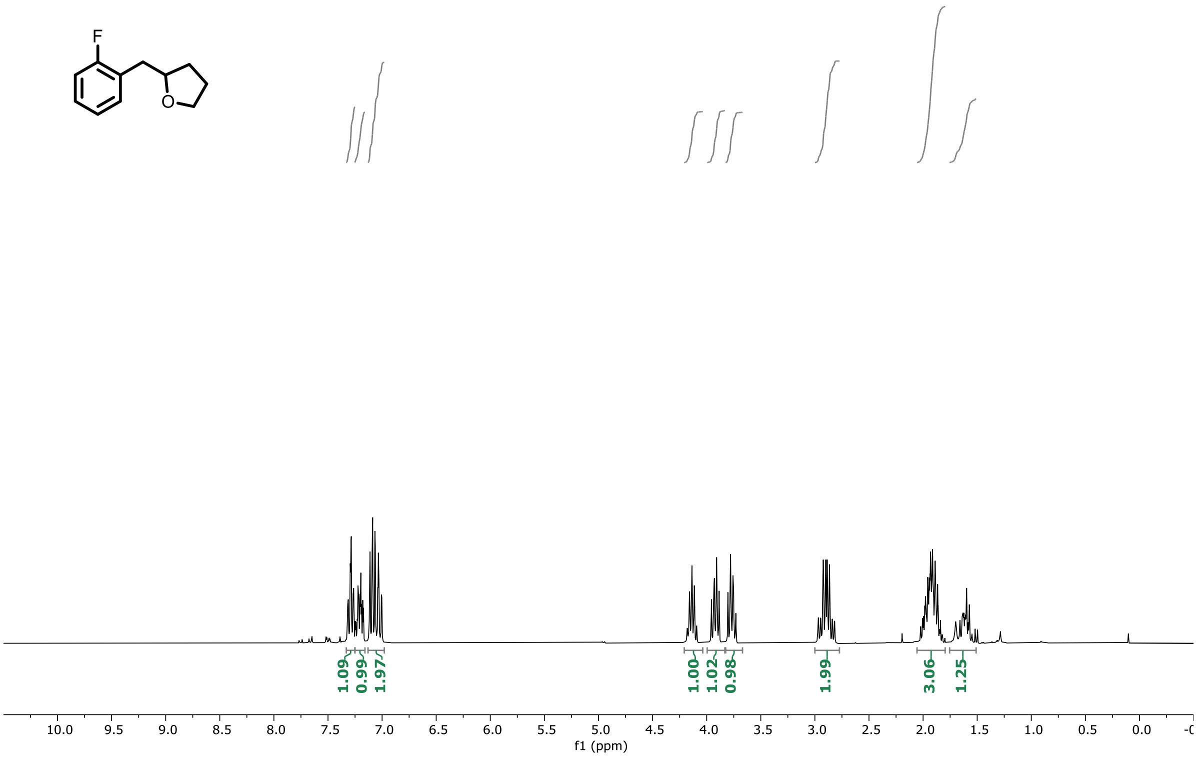

^{13}C NMR (75 MHz, CDCl$_3$) of **7**

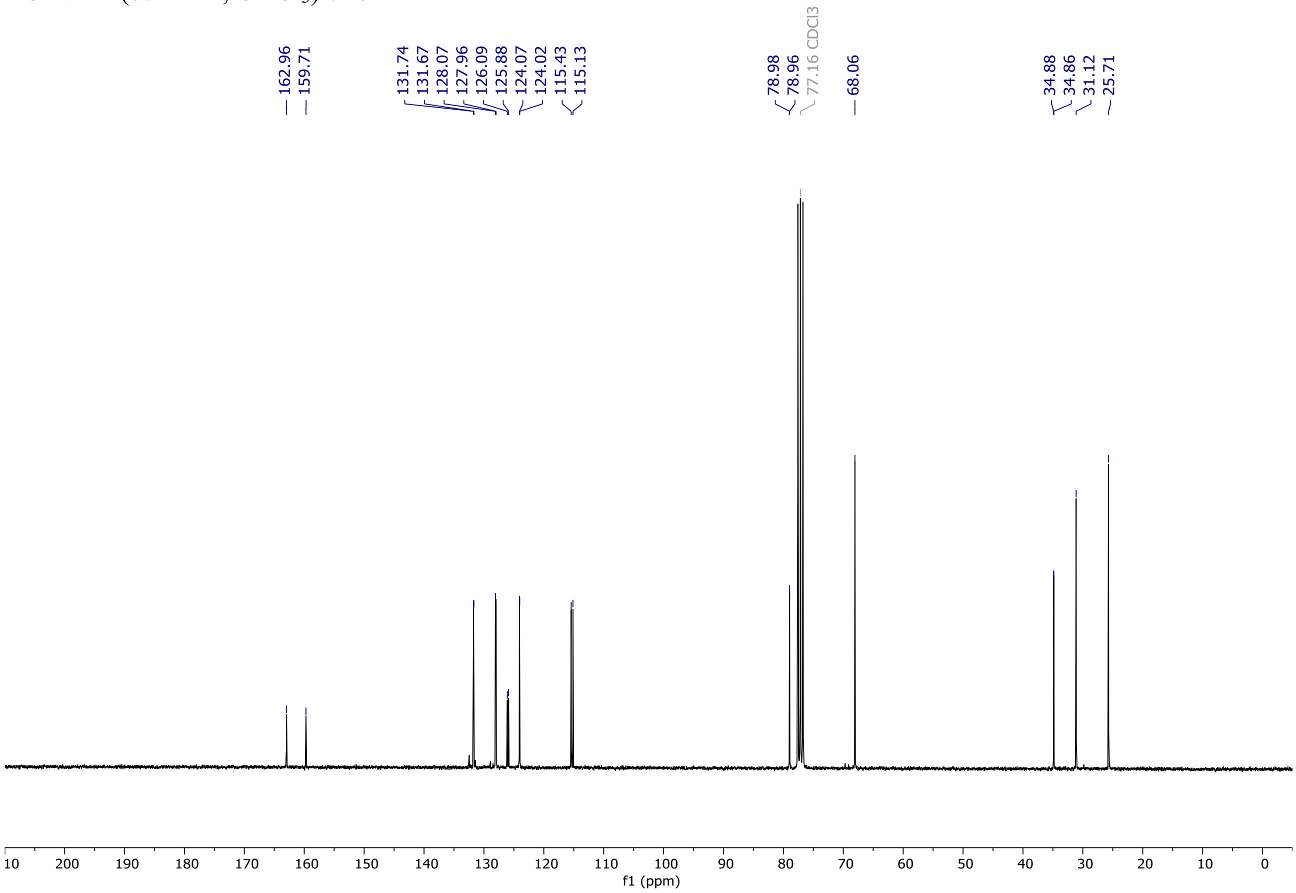

^{19}F NMR (282 MHz, $CDCl_3$) of **7**

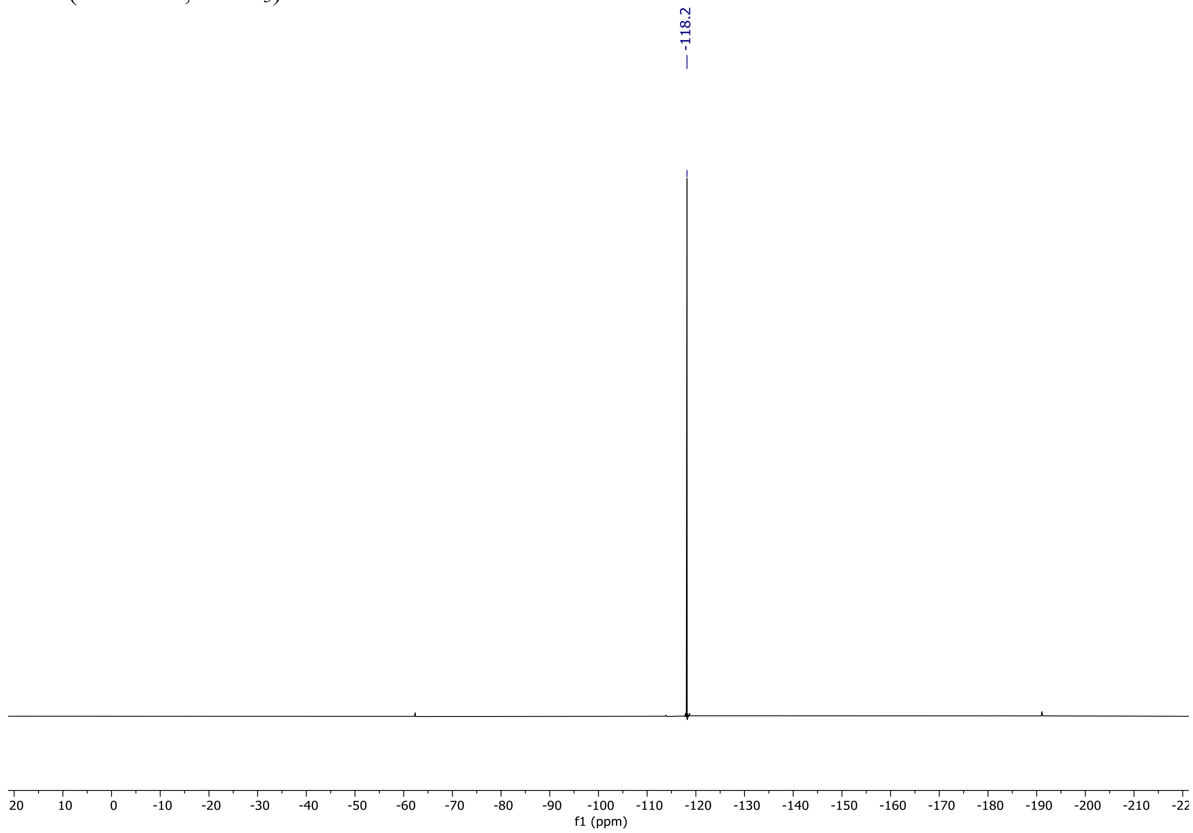

^{1}H NMR (400 MHz,$CDCl_3$) of **8**

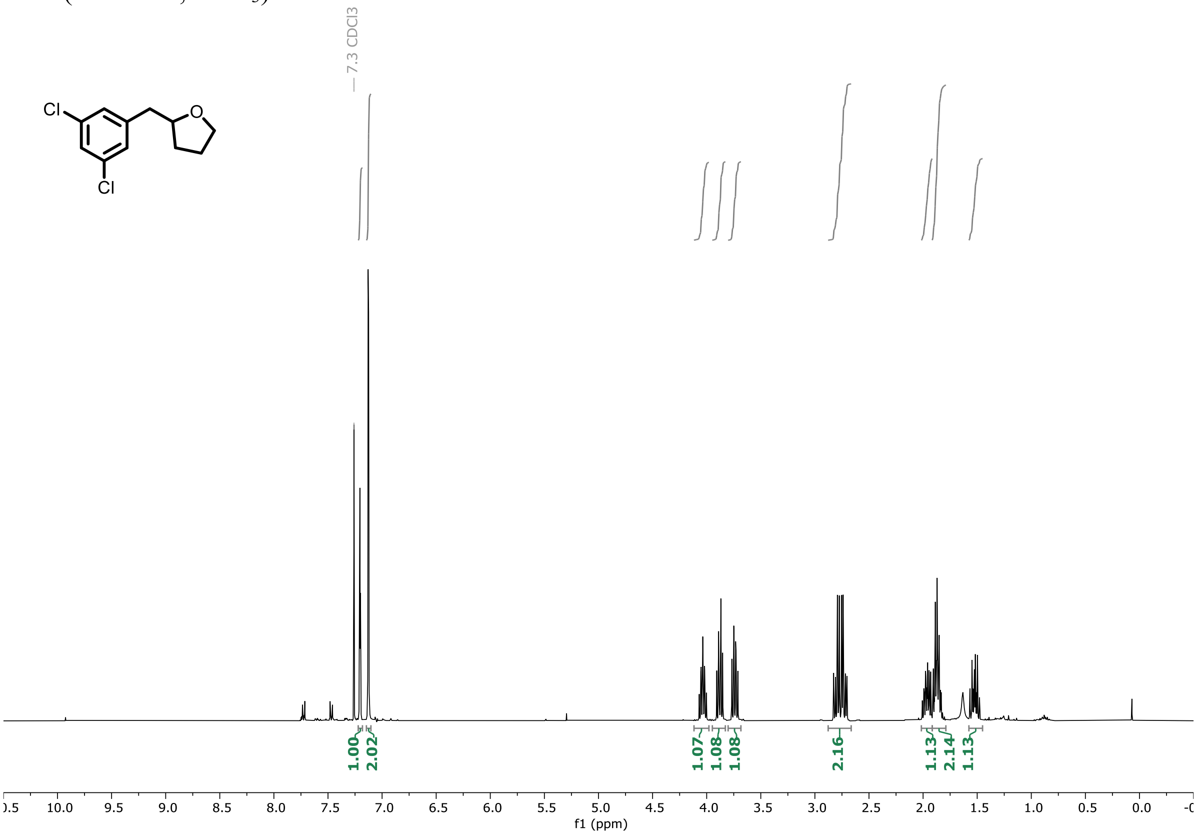

^{13}C NMR (101 MHz, $CDCl_3$) of **8**

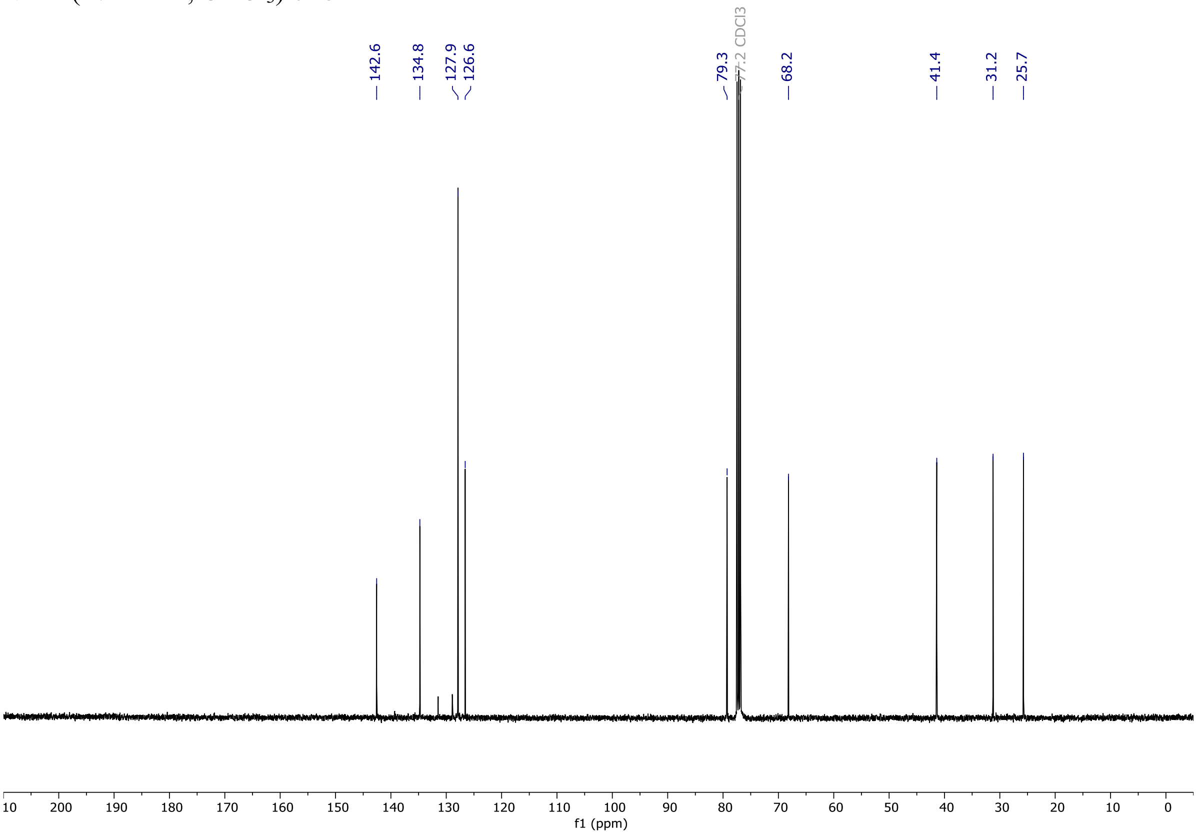

^{1}H NMR (400 MHz,$CDCl_3$) of **9**

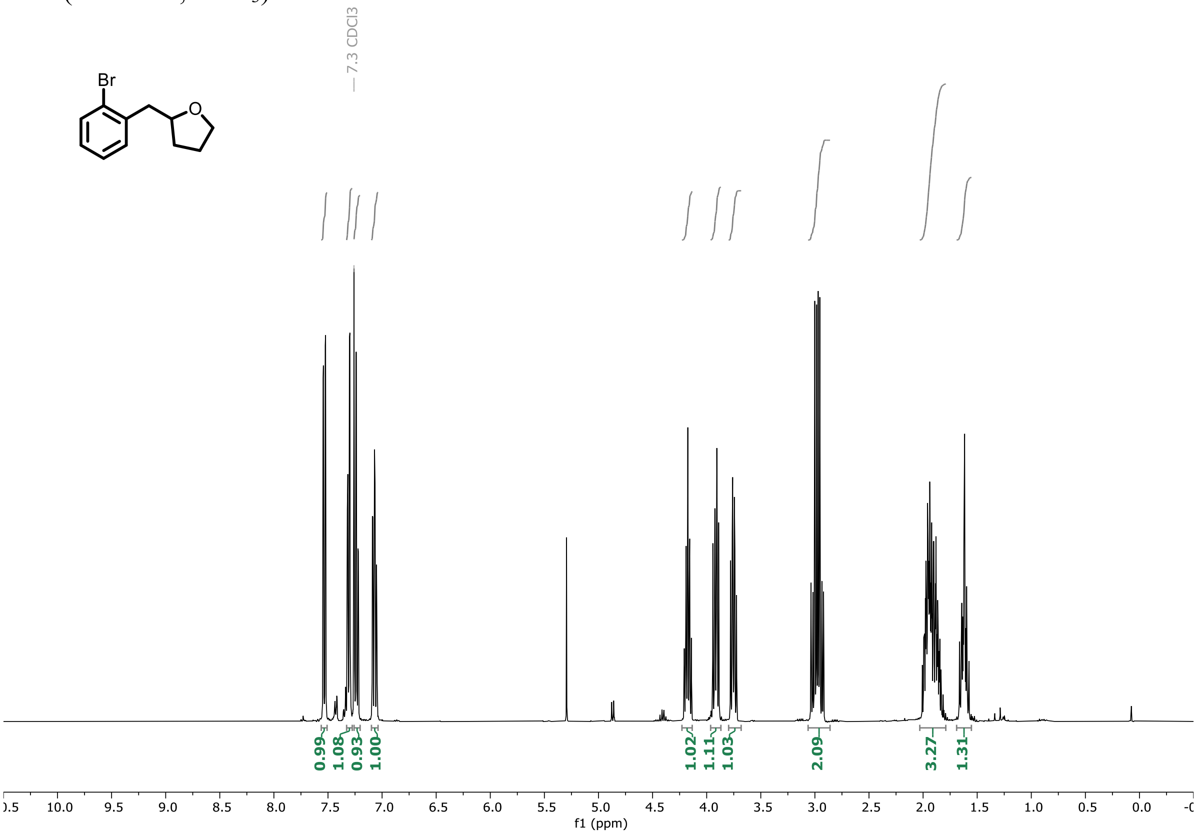

^{13}C NMR (101 MHz, $CDCl_3$) of **9**

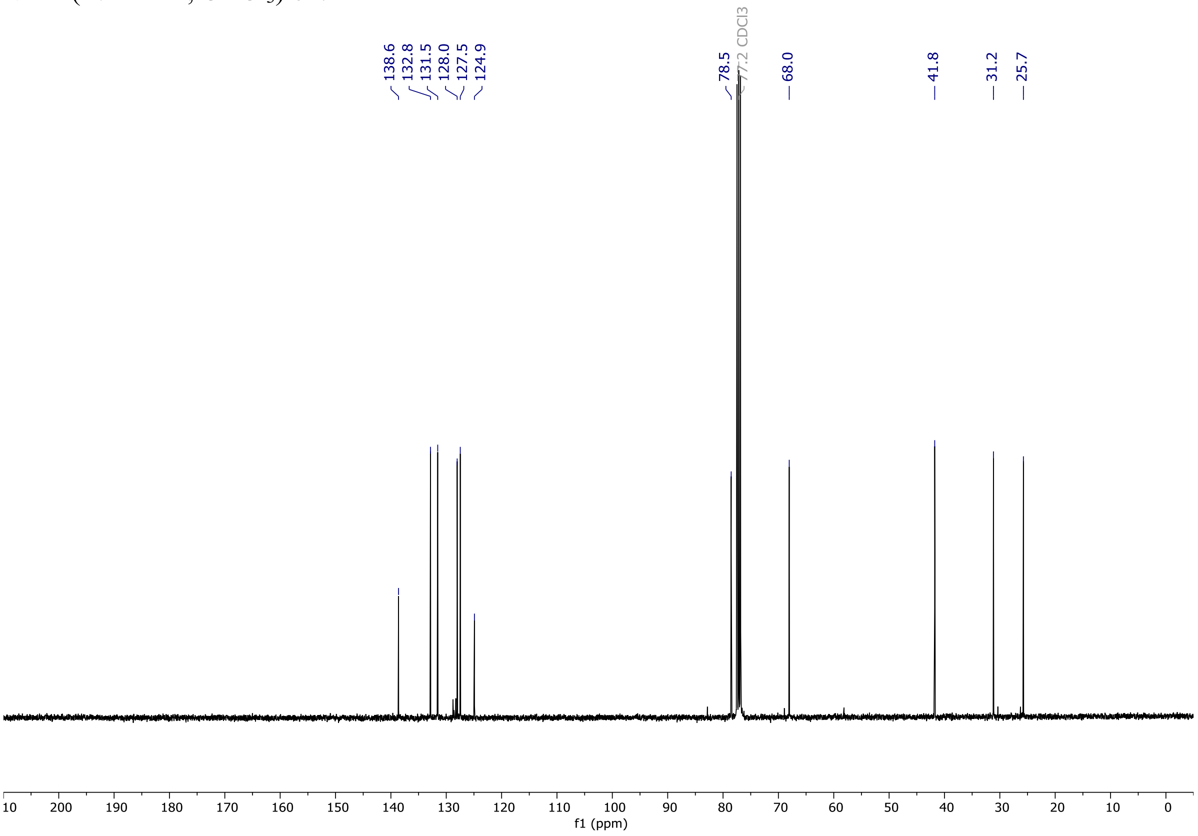

^{1}H NMR (400 MHz, $CDCl_3$) of **10**

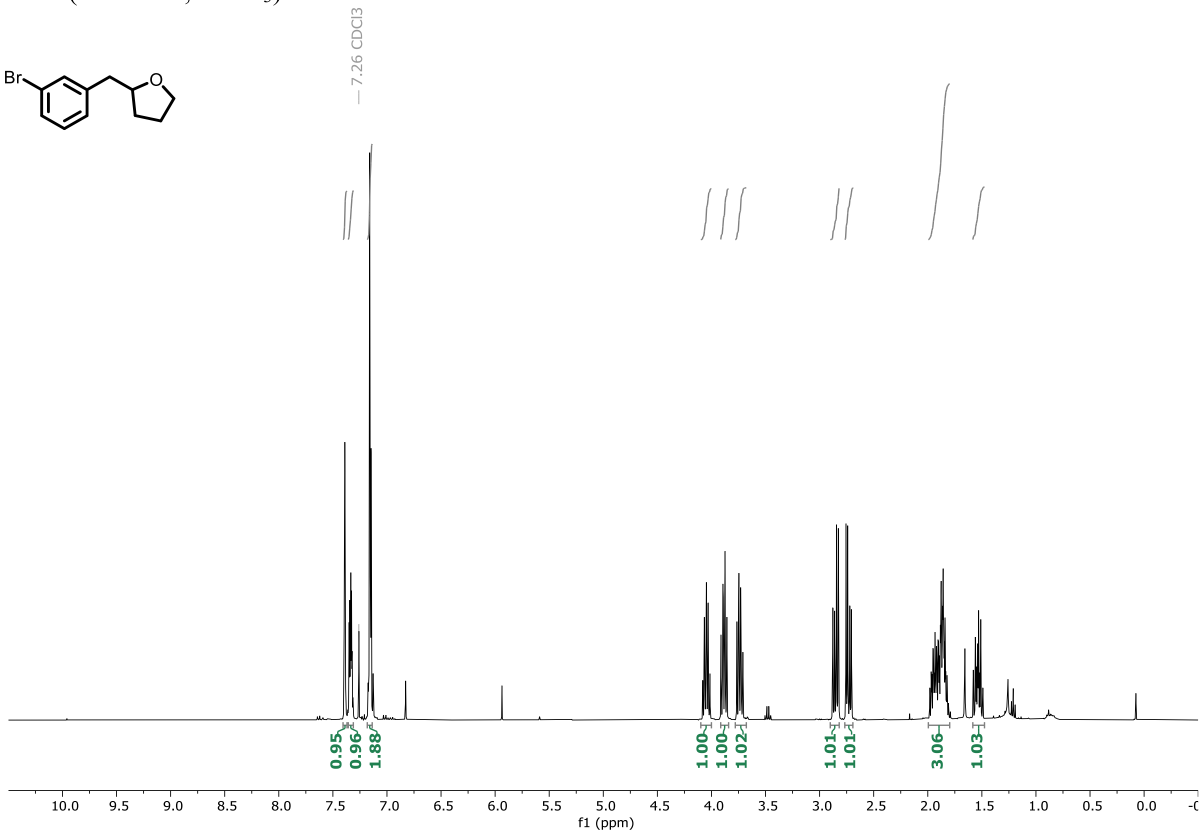

^{13}C NMR (101 MHz, $CDCl_3$) of **10**

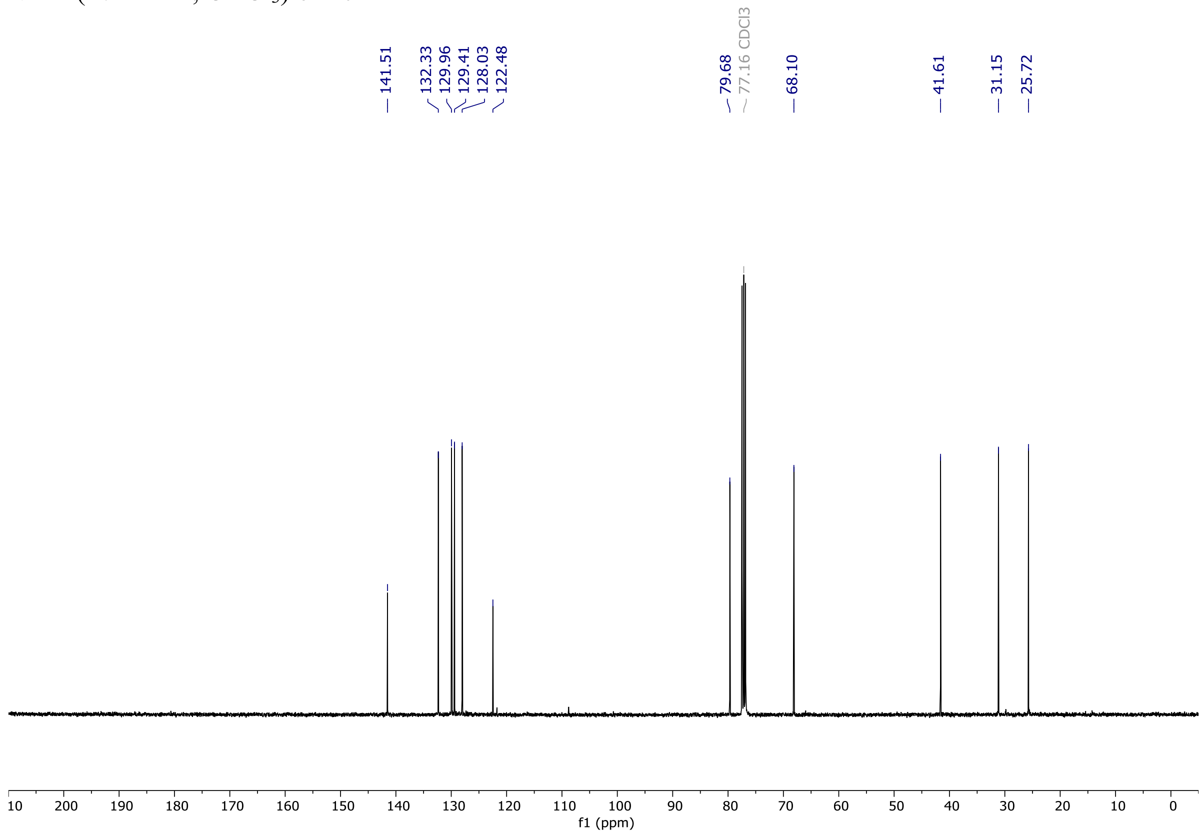

^{1}H NMR (400 MHz, $CDCl_3$) of **11**

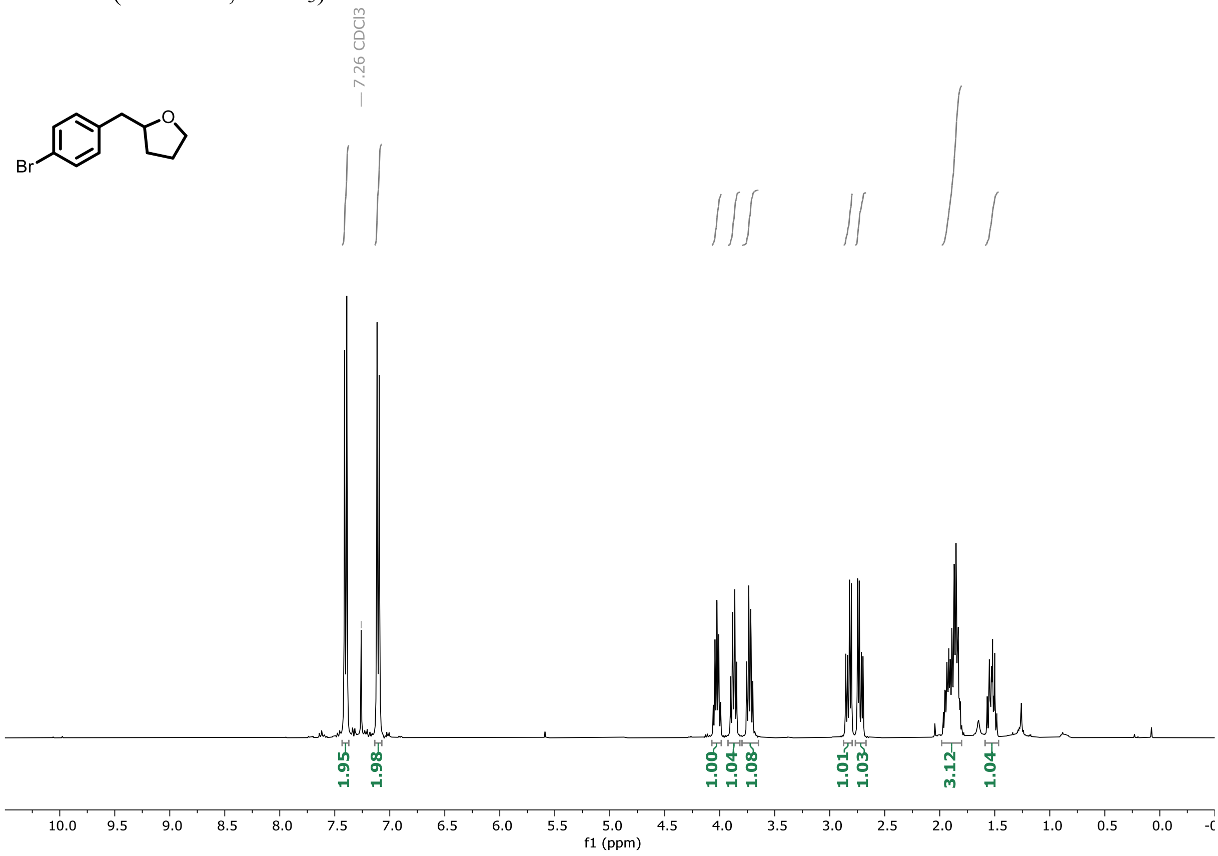

^{13}C NMR (101 MHz, $CDCl_3$) of **11**

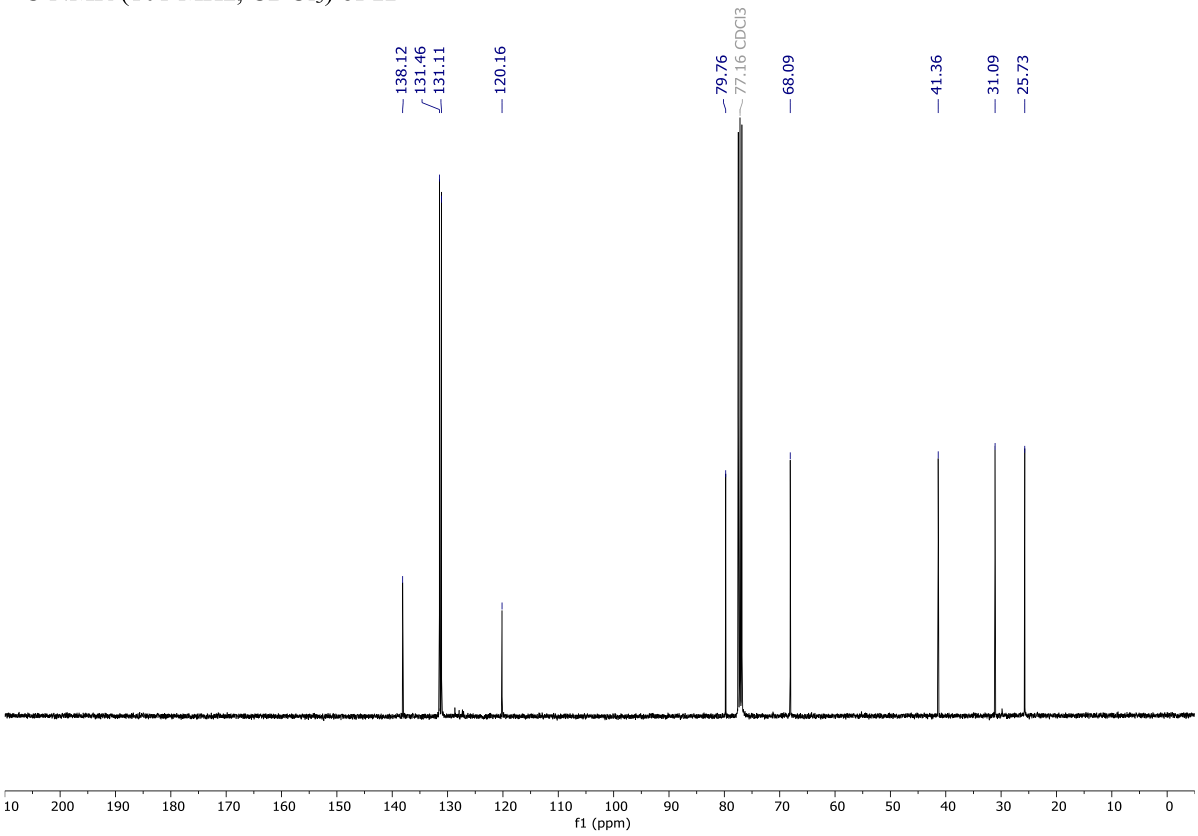

^{1}H NMR (400 MHz,CDCl$_3$) of **12**

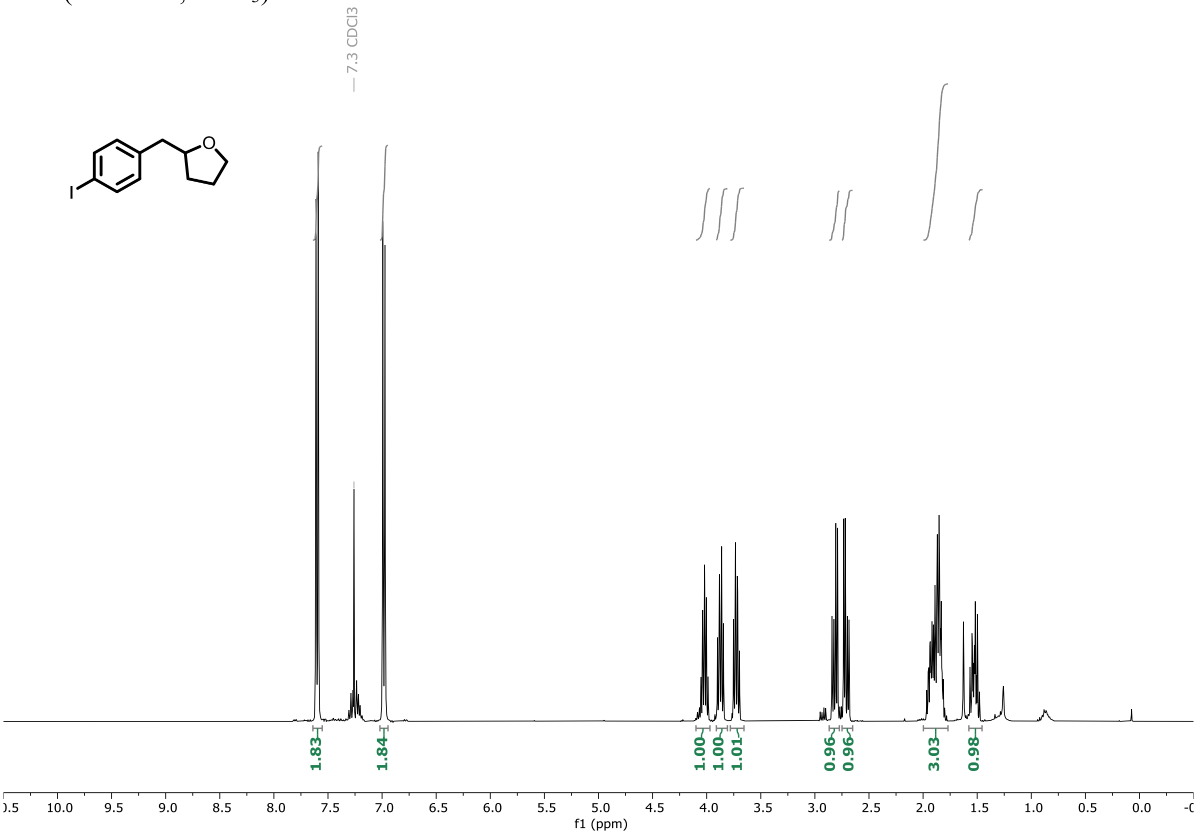

^{13}C NMR (101 MHz, CDCl$_3$) of **12**

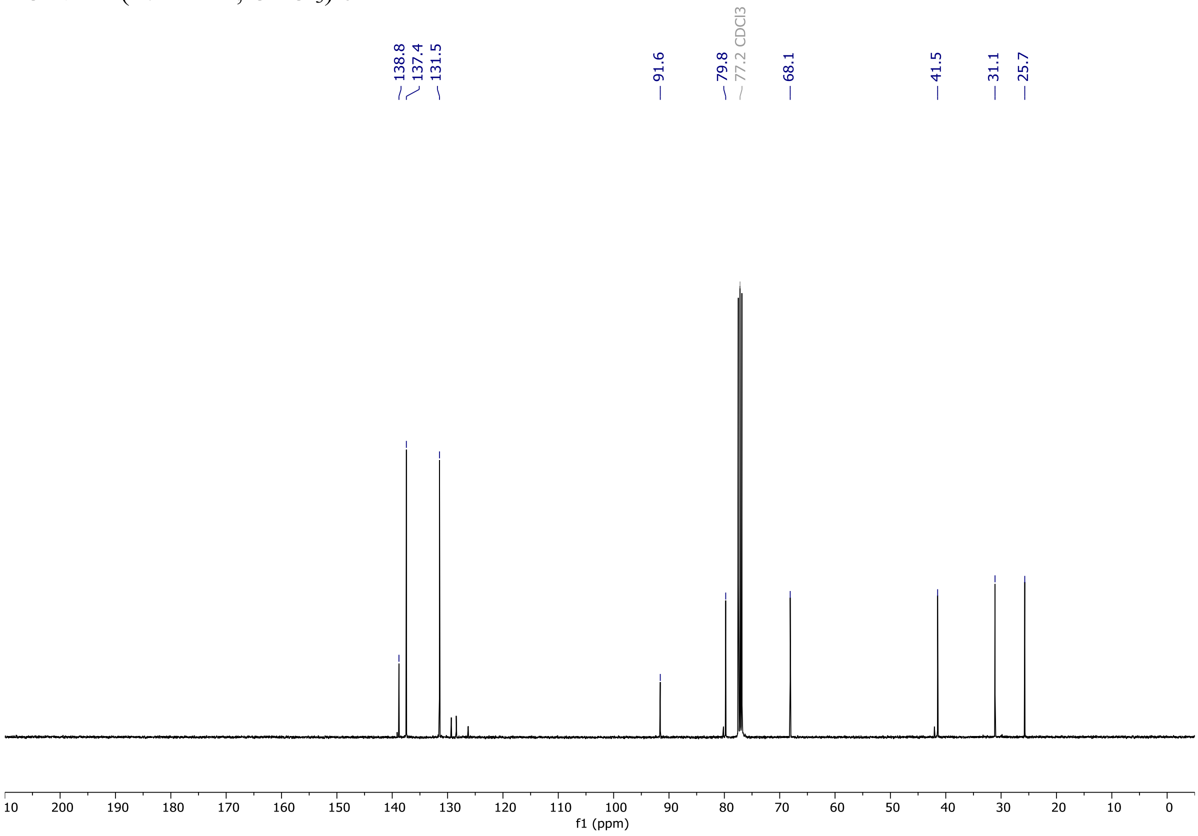

^{1}H NMR (400 MHz, $CDCl_3$) of **13**

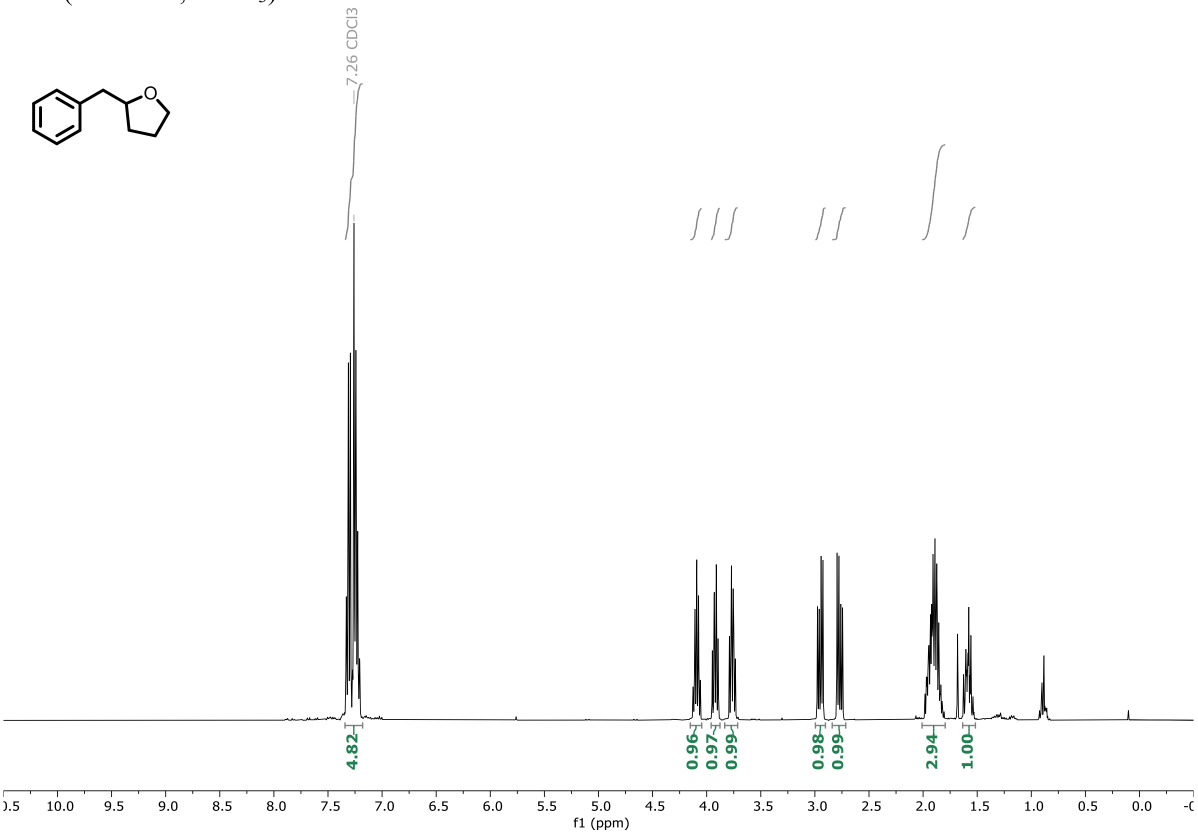

^{13}C NMR (101 MHz, $CDCl_3$) of **13**

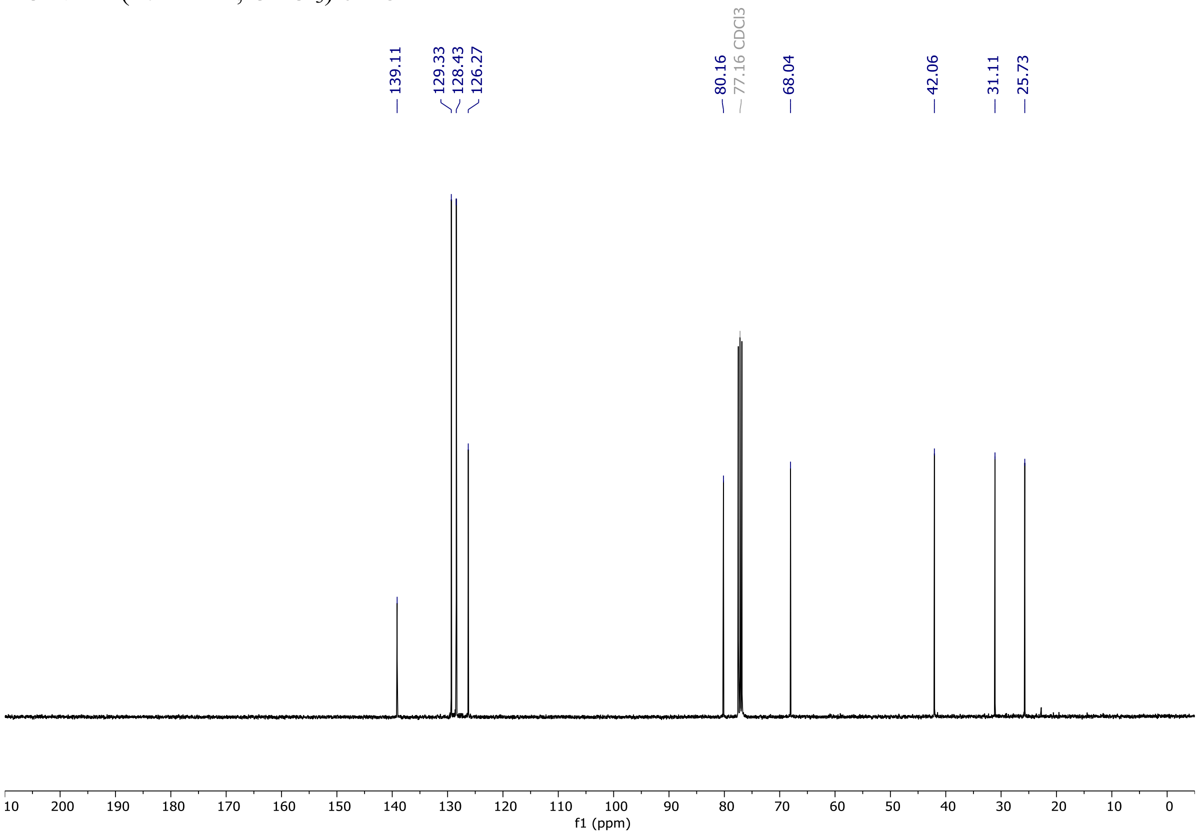

^{1}H NMR (400 MHz, $CDCl_3$) of **14**

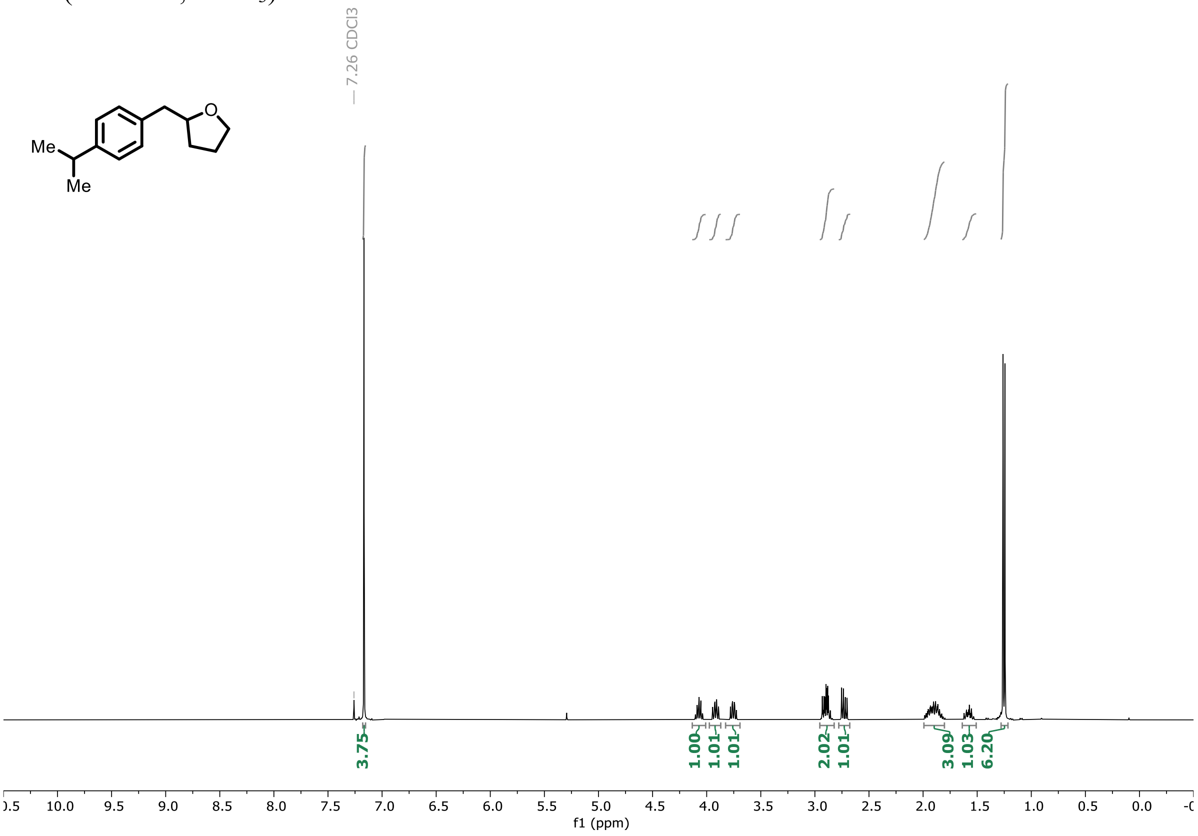

^{13}C NMR (101 MHz, $CDCl_3$) of **14**

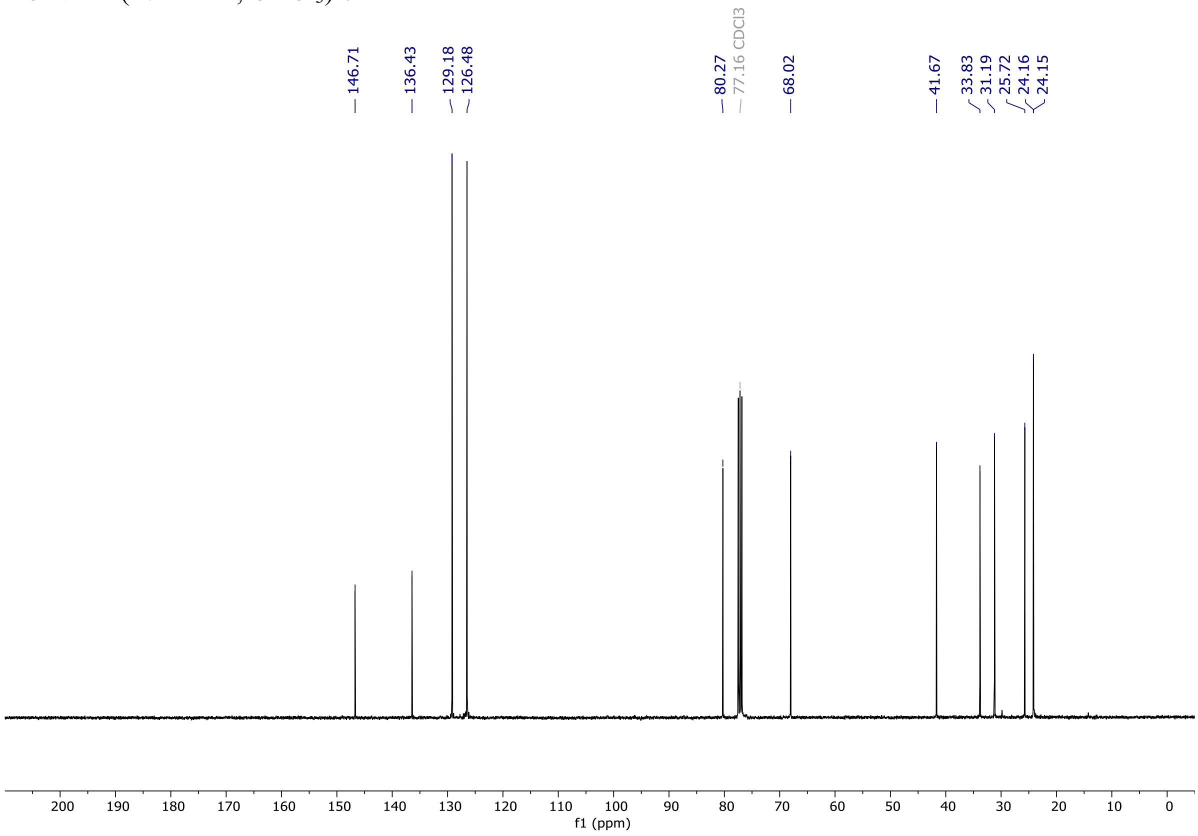

^{1}H NMR (400 MHz, $CDCl_3$) of **15**

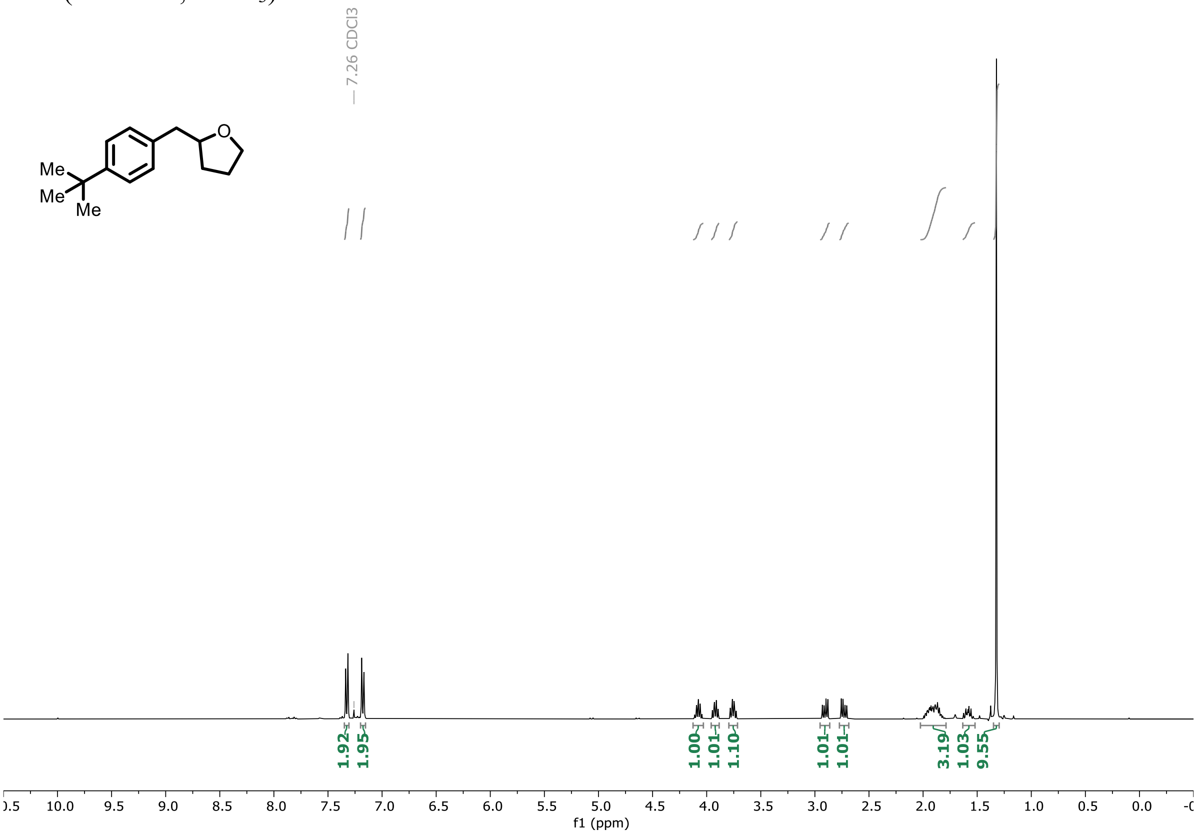

^{13}C NMR (101 MHz, $CDCl_3$) of **15**

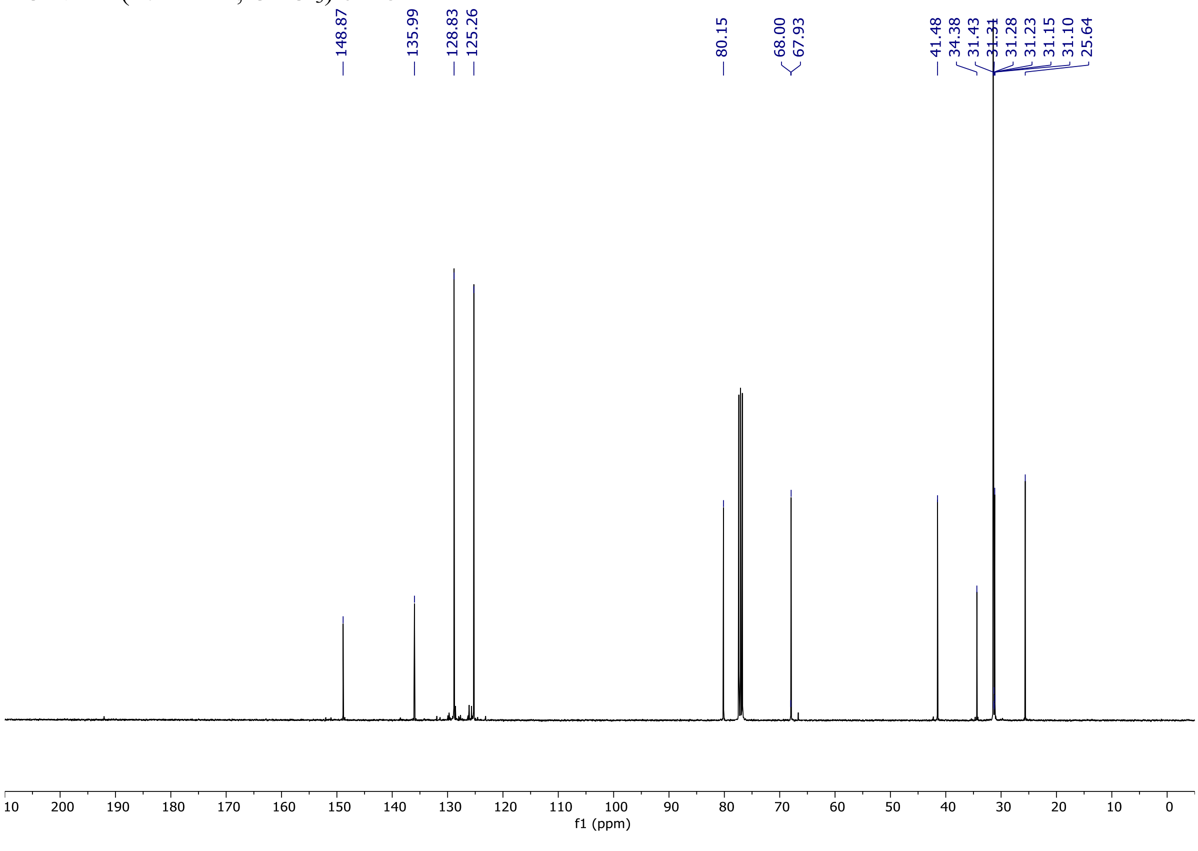

^{1}H NMR (400 MHz, $CDCl_3$) of **16**

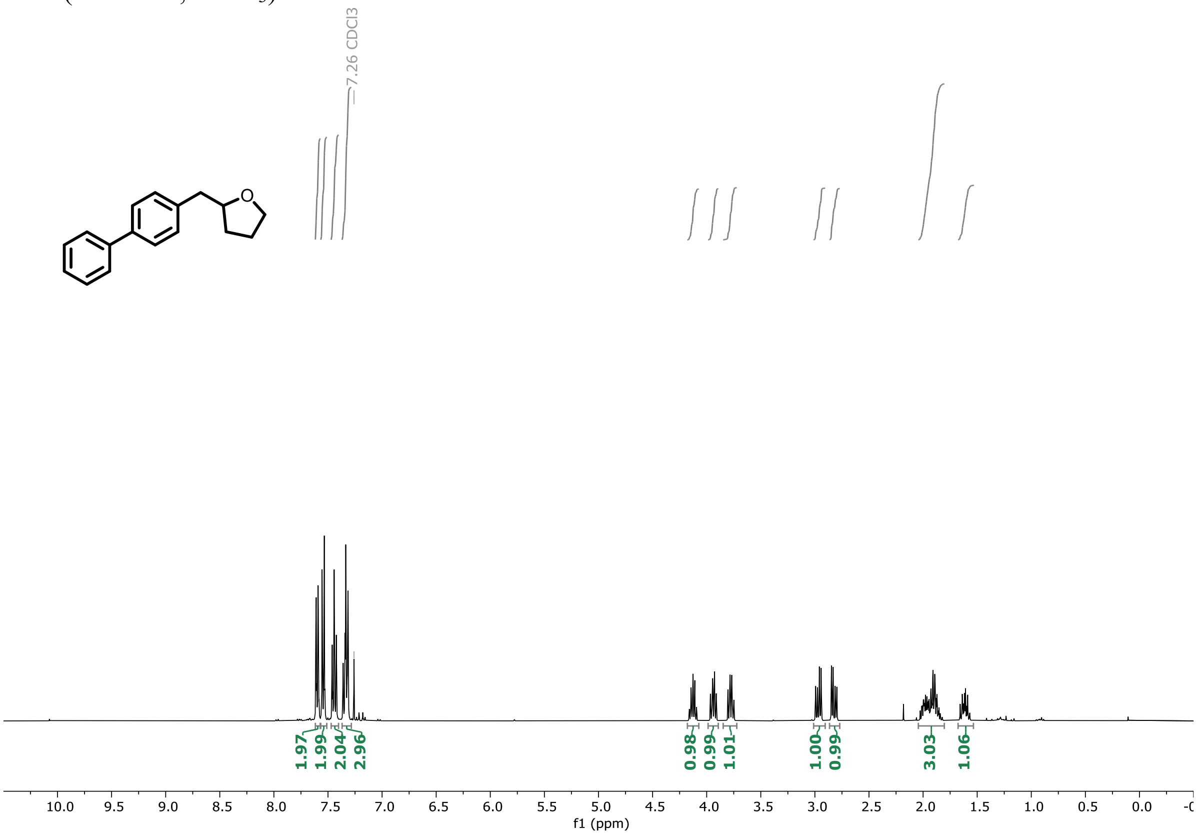

^{13}C NMR (101 MHz, $CDCl_3$) of **16**

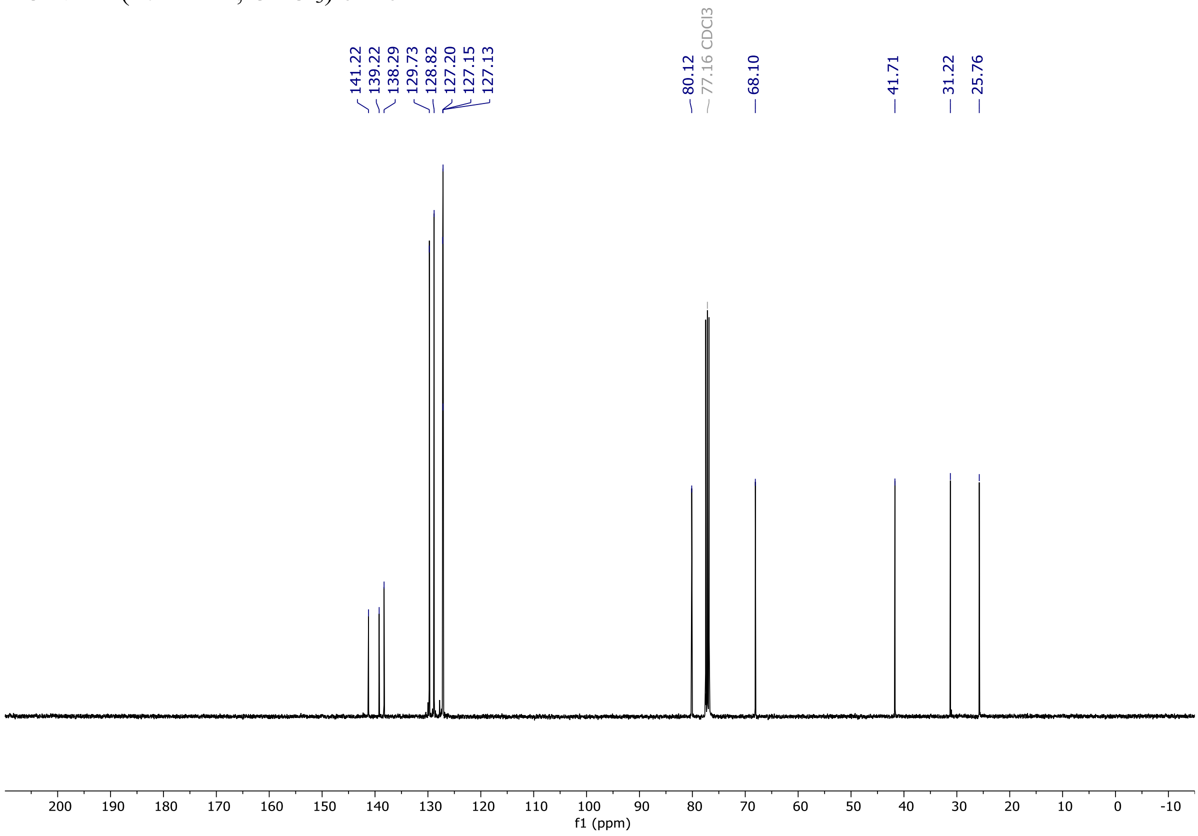

^{1}H NMR (400 MHz, $CDCl_3$) of **17**

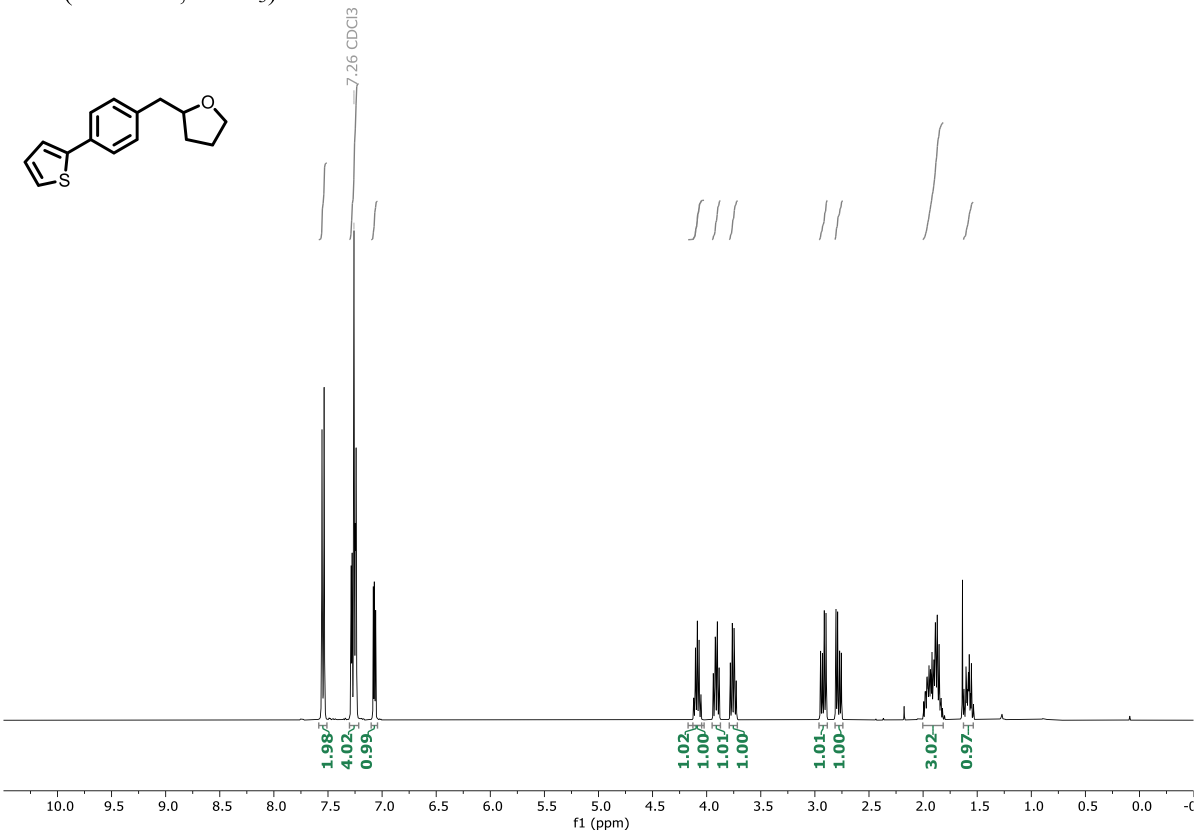

^{13}C NMR (101 MHz, $CDCl_3$) of **17**

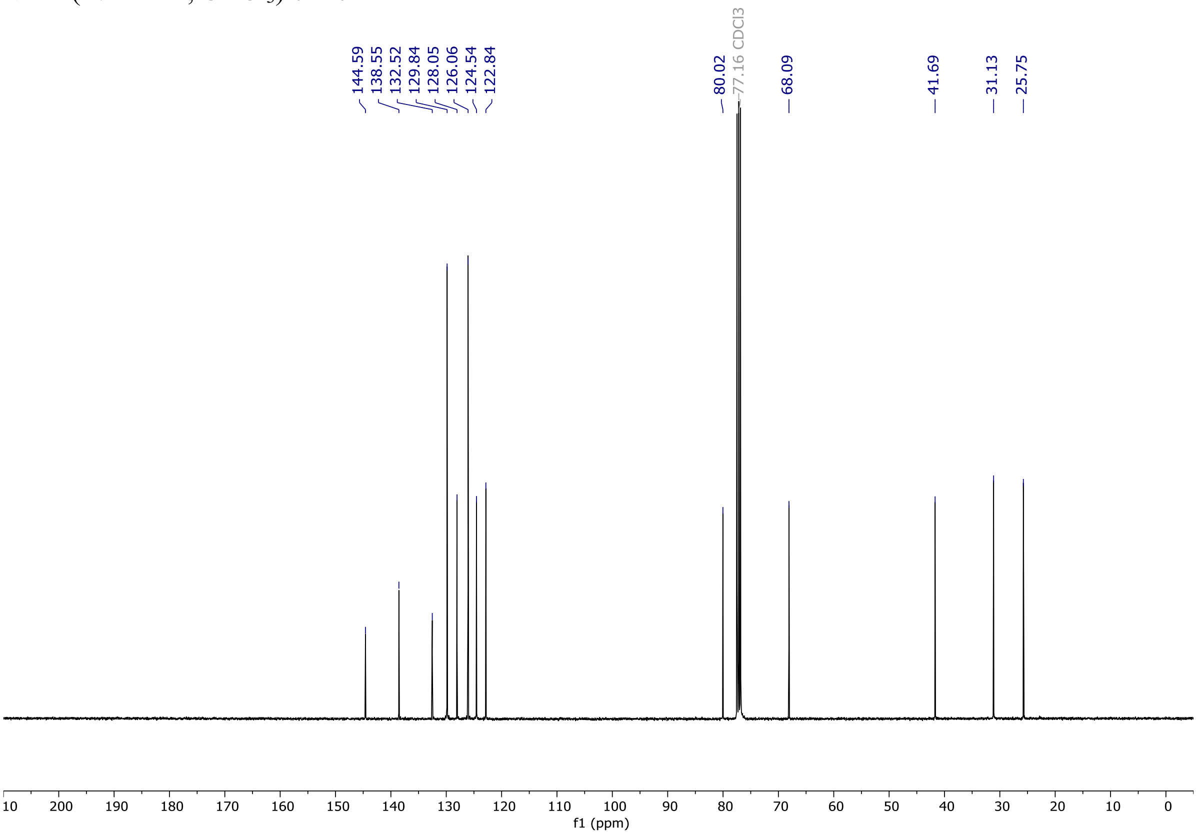

^{1}H NMR (400 MHz, $CDCl_3$) of **18**

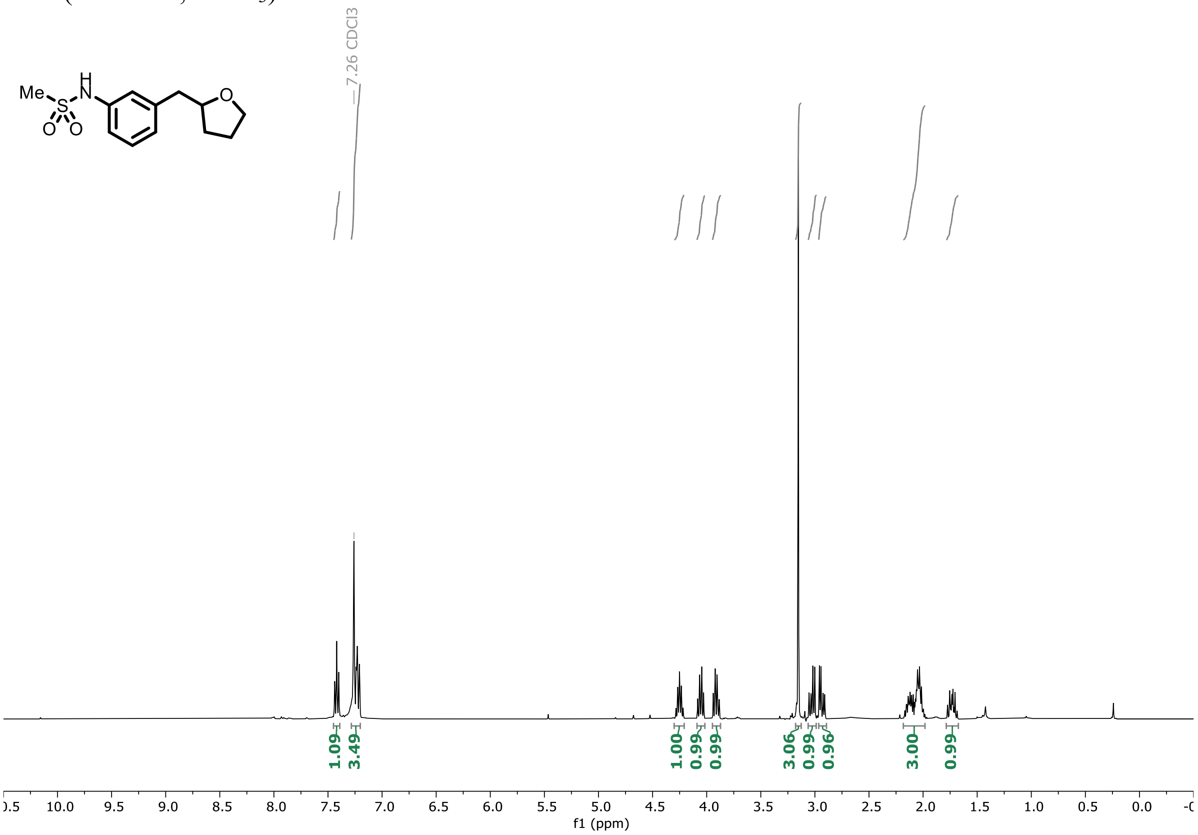

^{13}C NMR (101 MHz, $CDCl_3$) of **18**

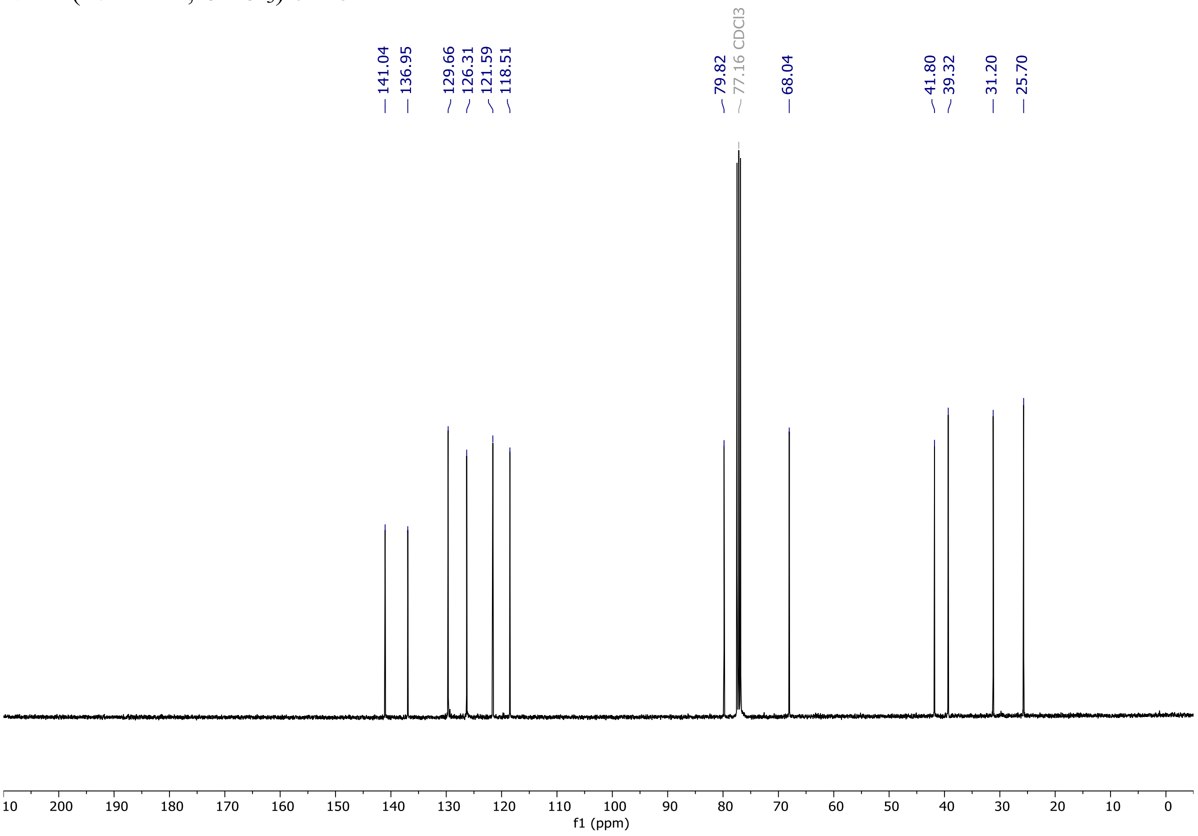

^1H NMR (400 MHz,$CDCl_3$) of **19**

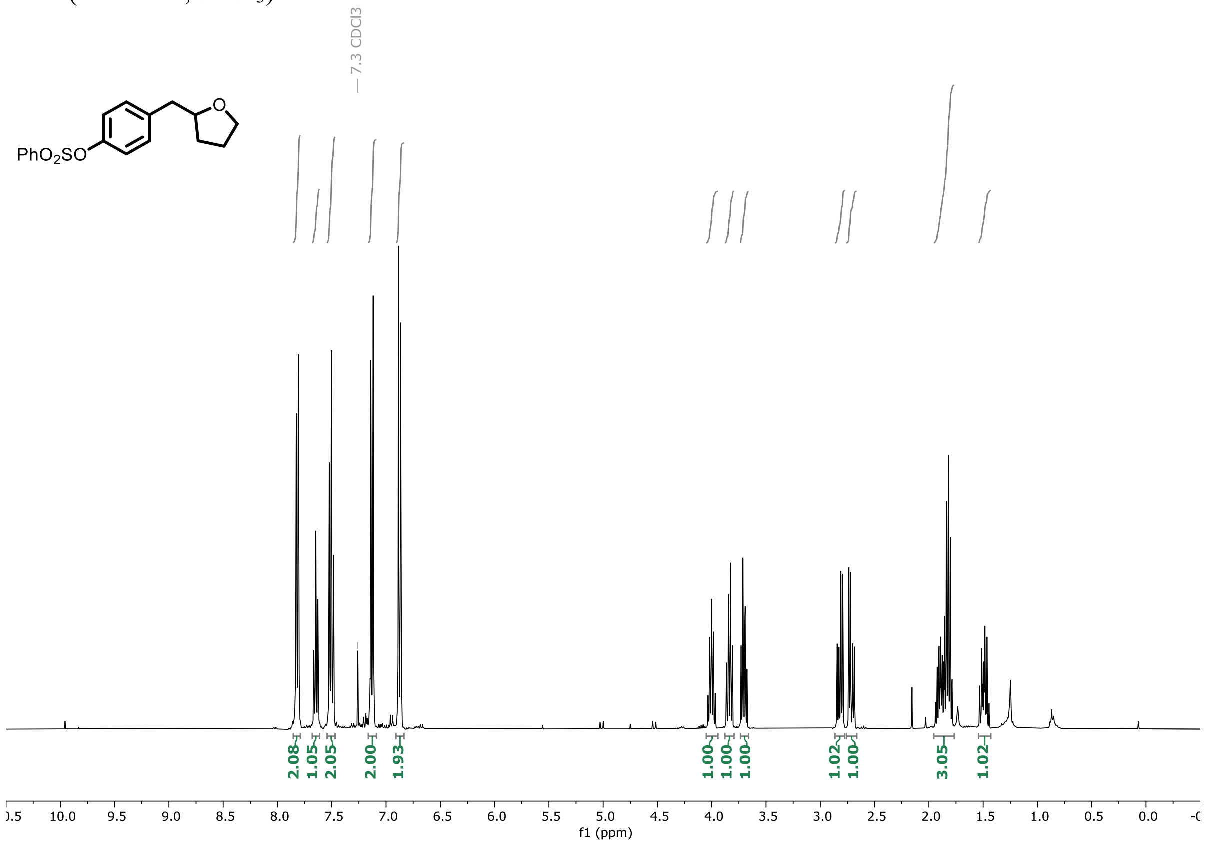

^{13}C NMR (101 MHz, $CDCl_3$) of **19**

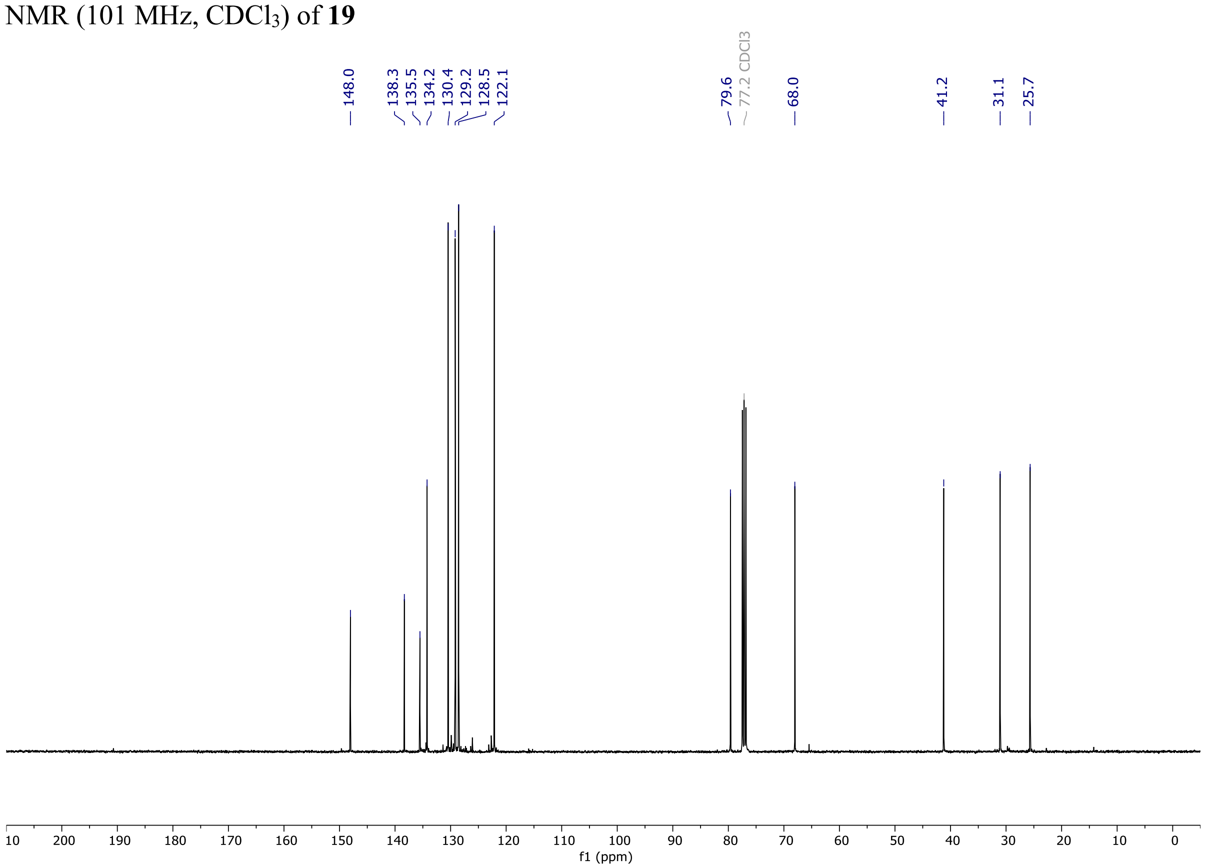

^{1}H NMR (400 MHz, $CDCl_3$) of **20**

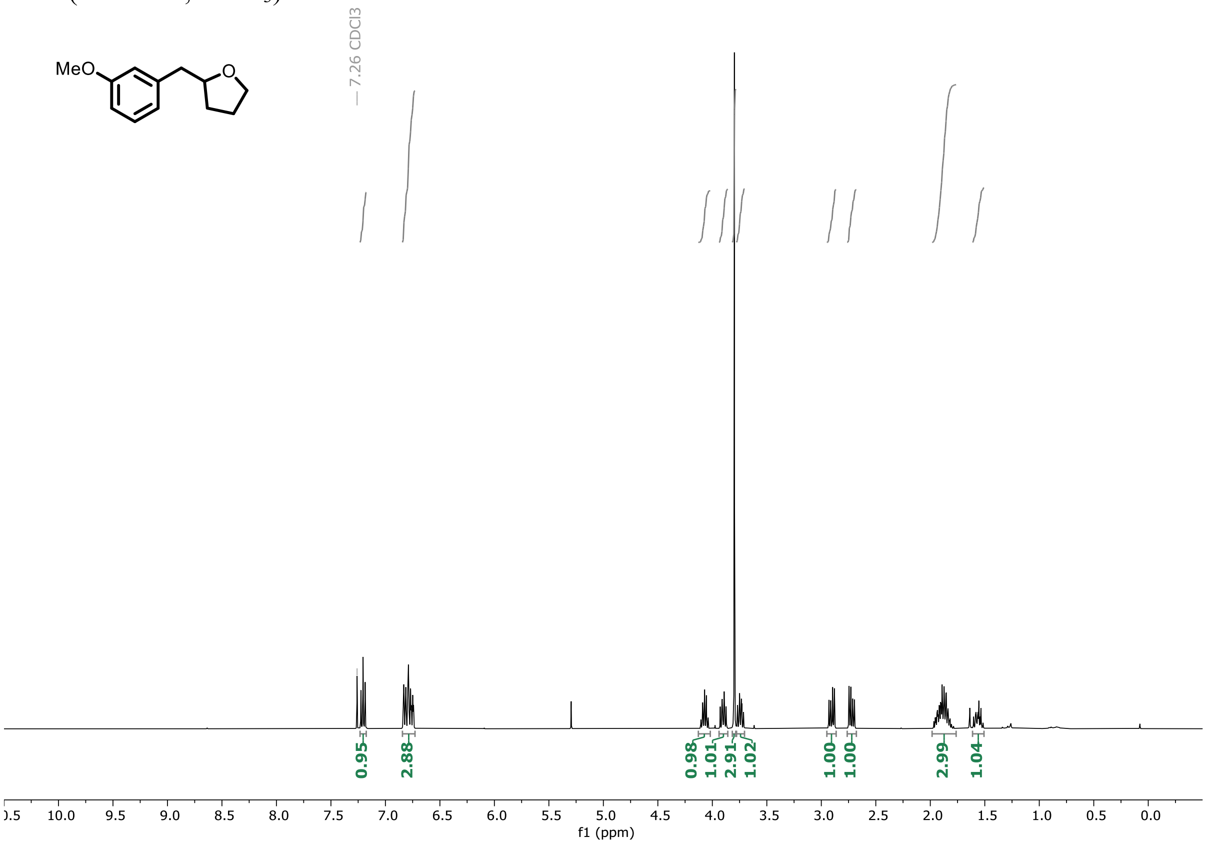

^{13}C NMR (101 MHz, $CDCl_3$) of **20**

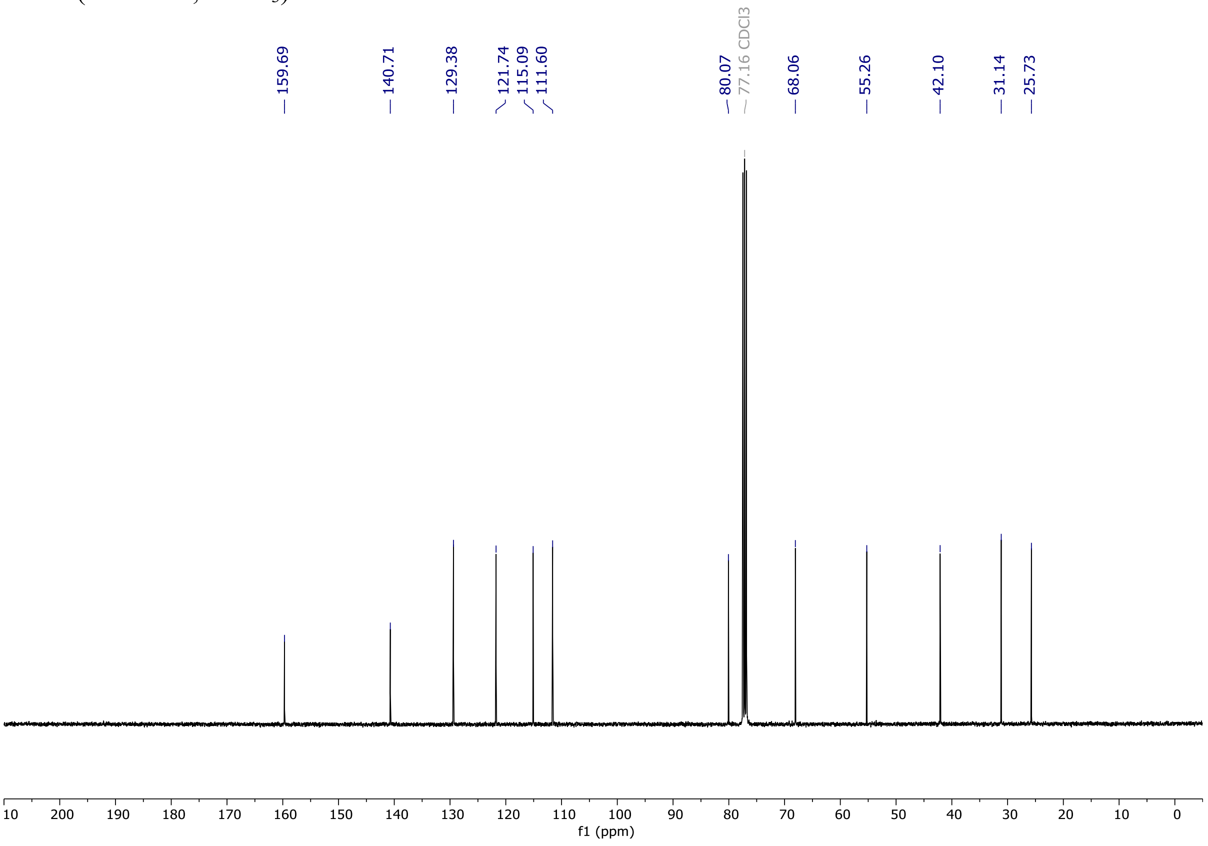

^{1}H NMR (400 MHz,$CDCl_3$) of **21**

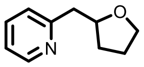

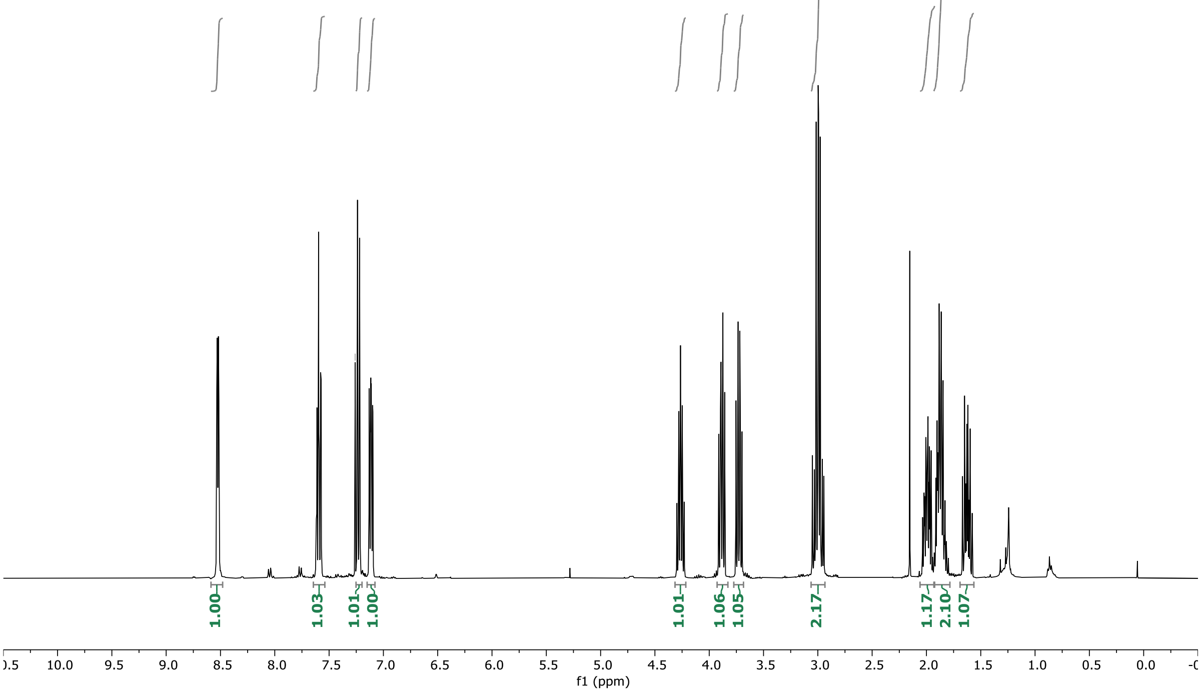

^{13}C NMR (101 MHz, $CDCl_3$) of **21**

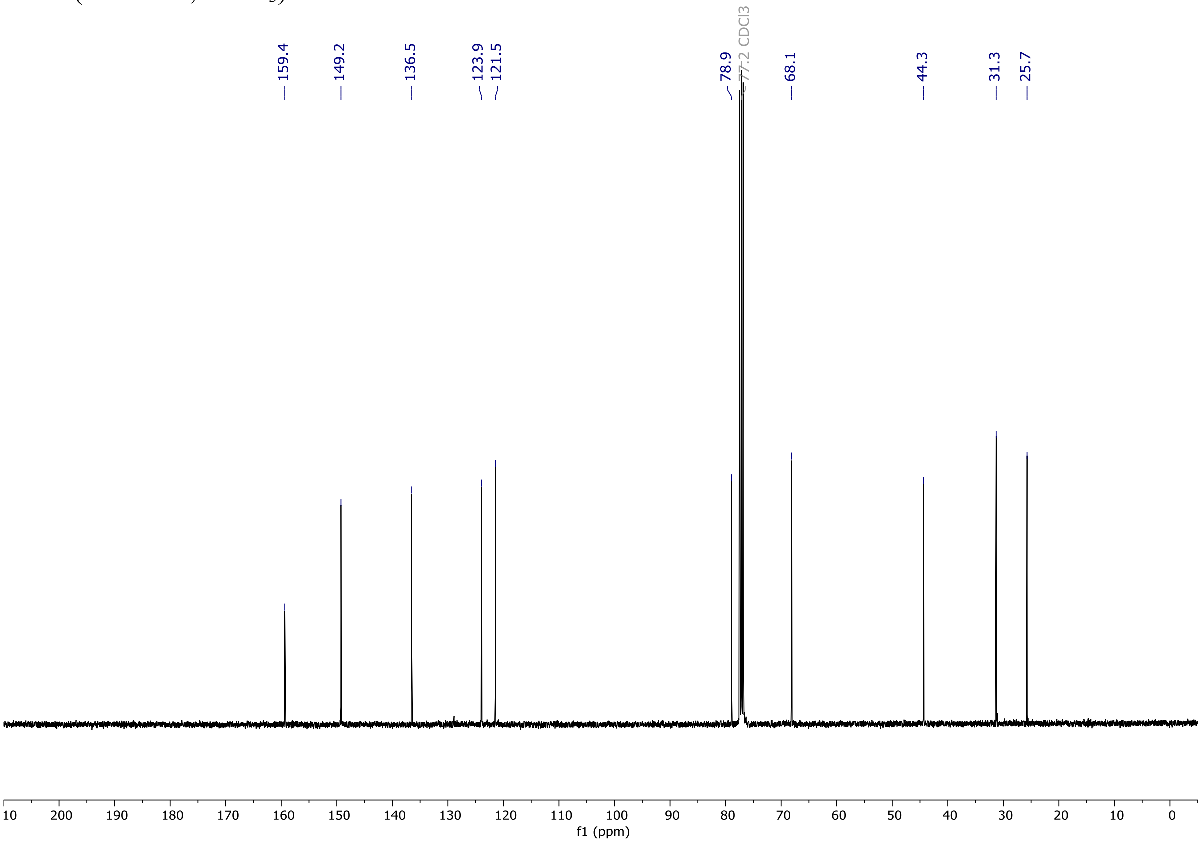

^{1}H NMR (400 MHz,CDCl$_3$) of **22**

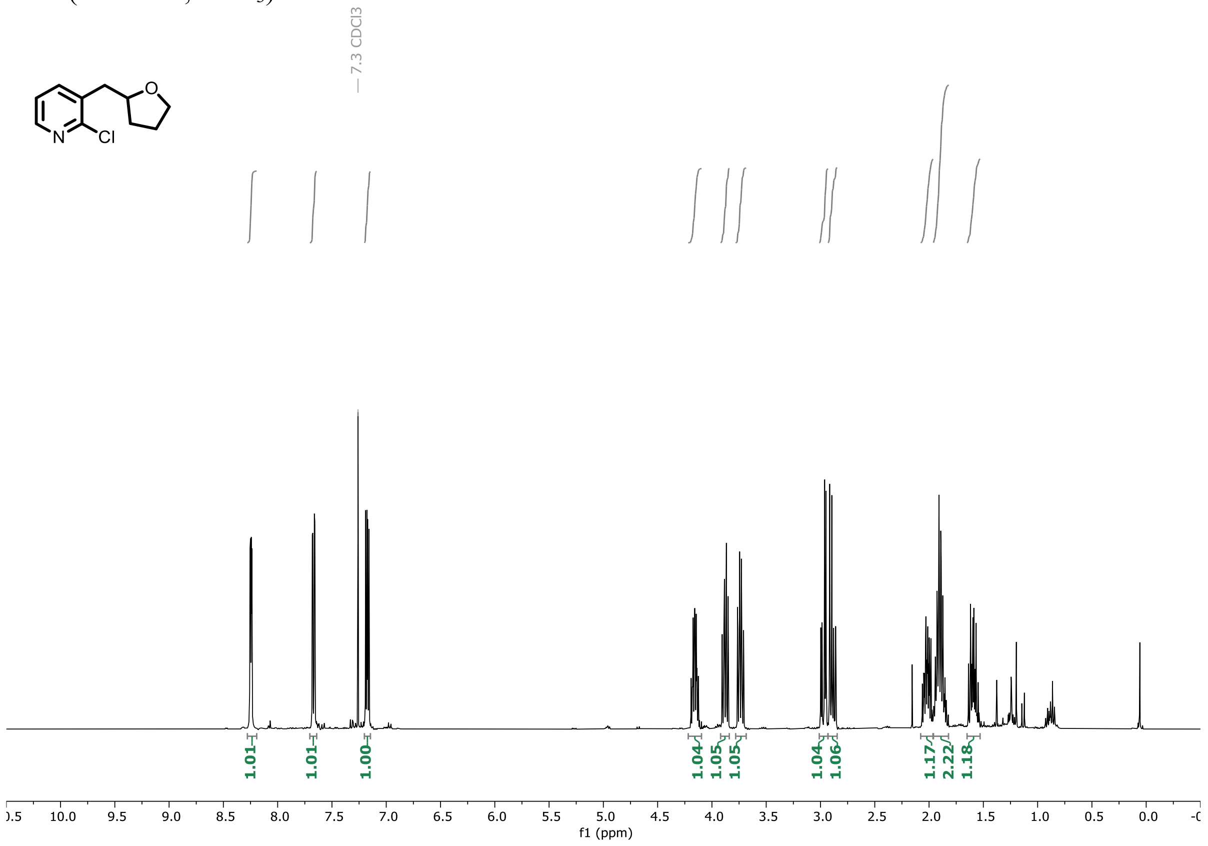

^{13}C NMR (101 MHz, CDCl$_3$) of **22**

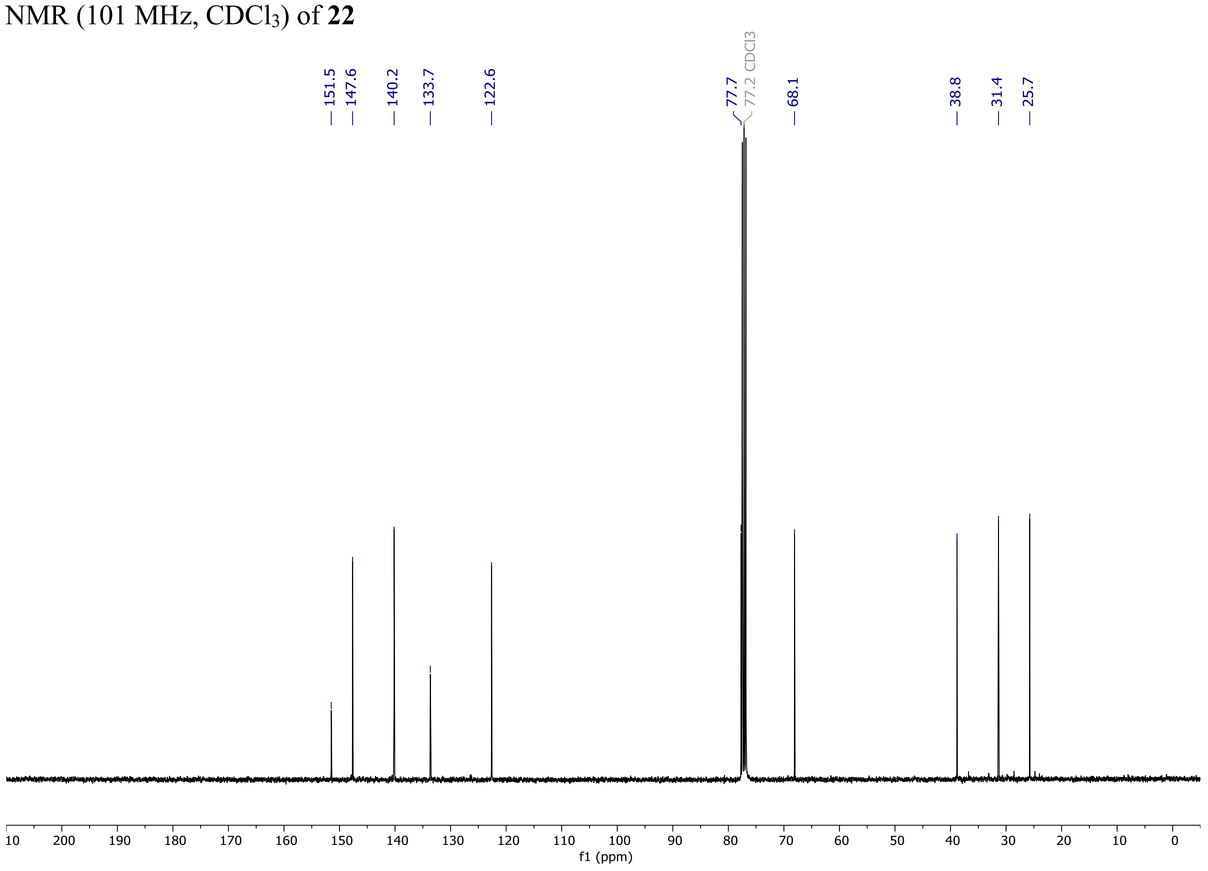

^1H NMR (400 MHz, $CDCl_3$) of **23**

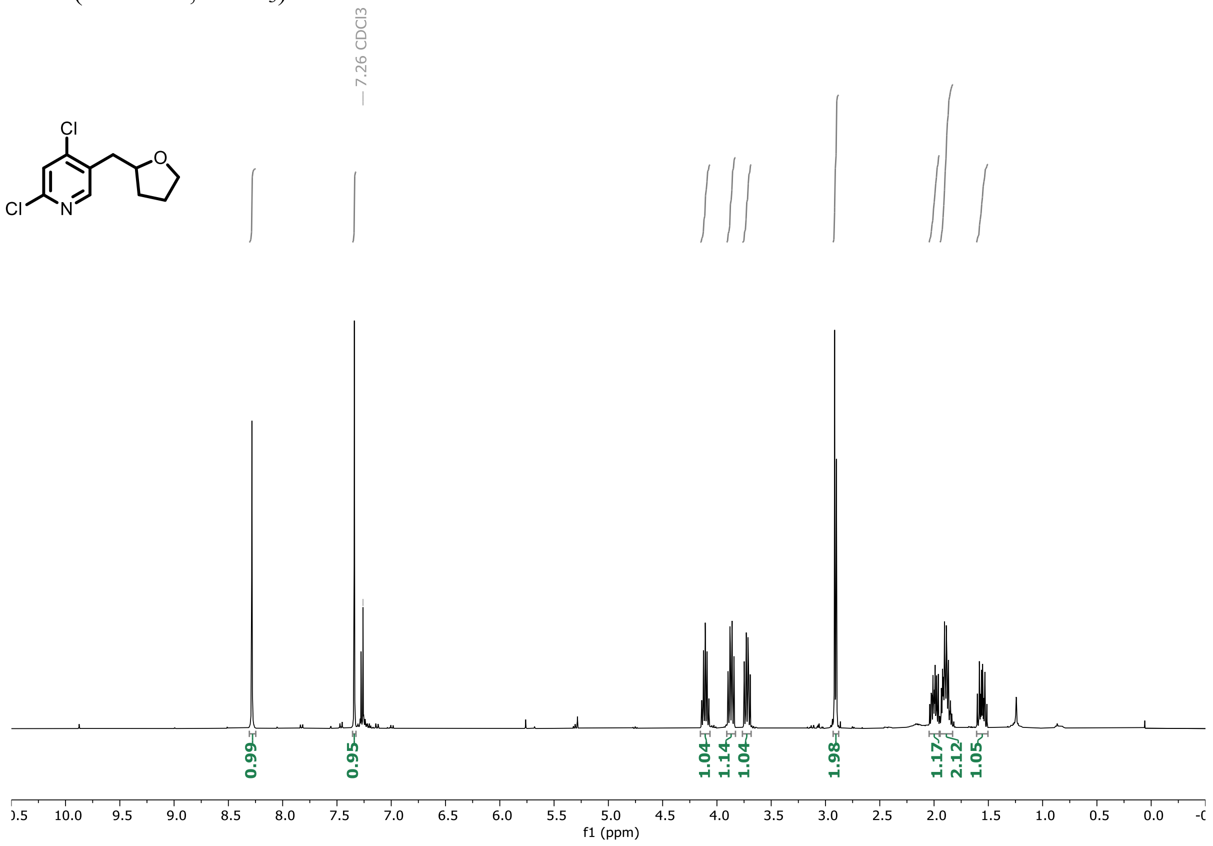

^{13}C NMR (101 MHz, $CDCl_3$) of **23**

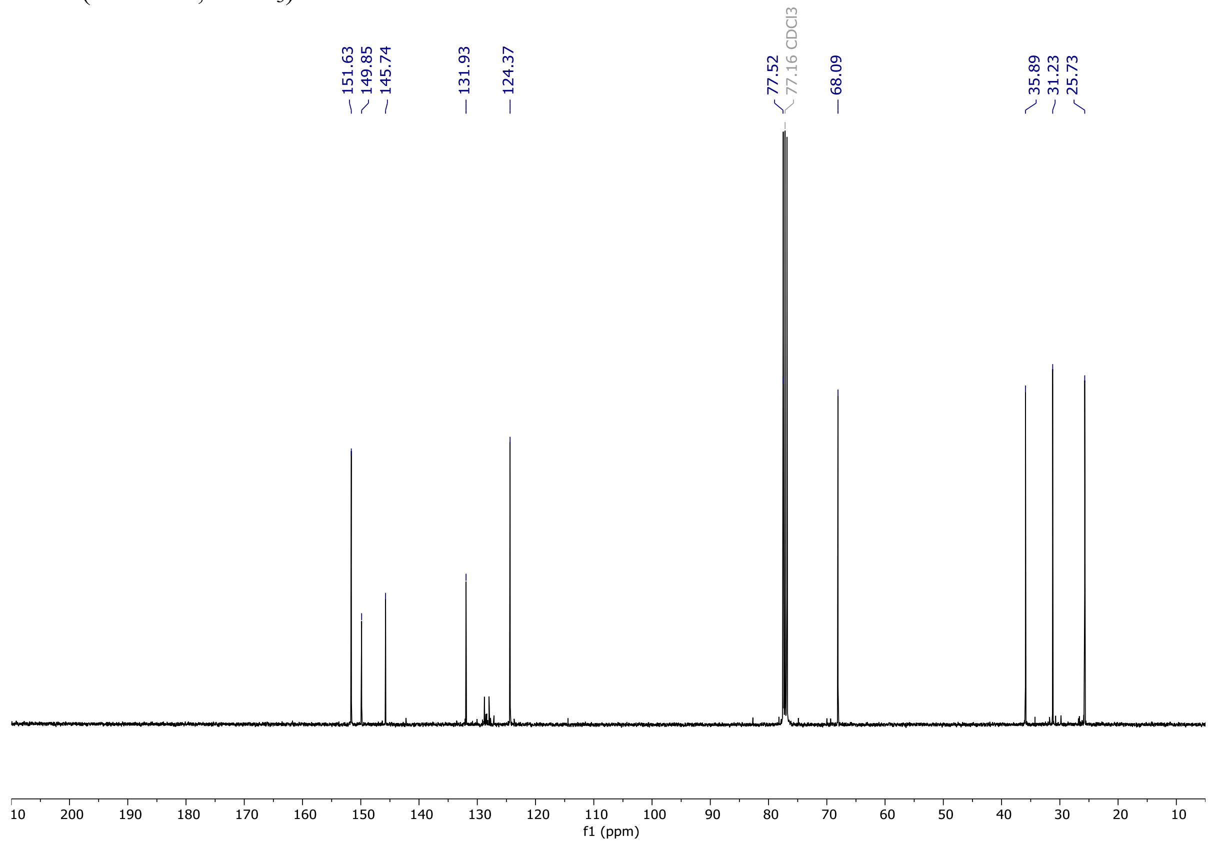

^{1}H NMR (400 MHz, $CDCl_3$) of **24**

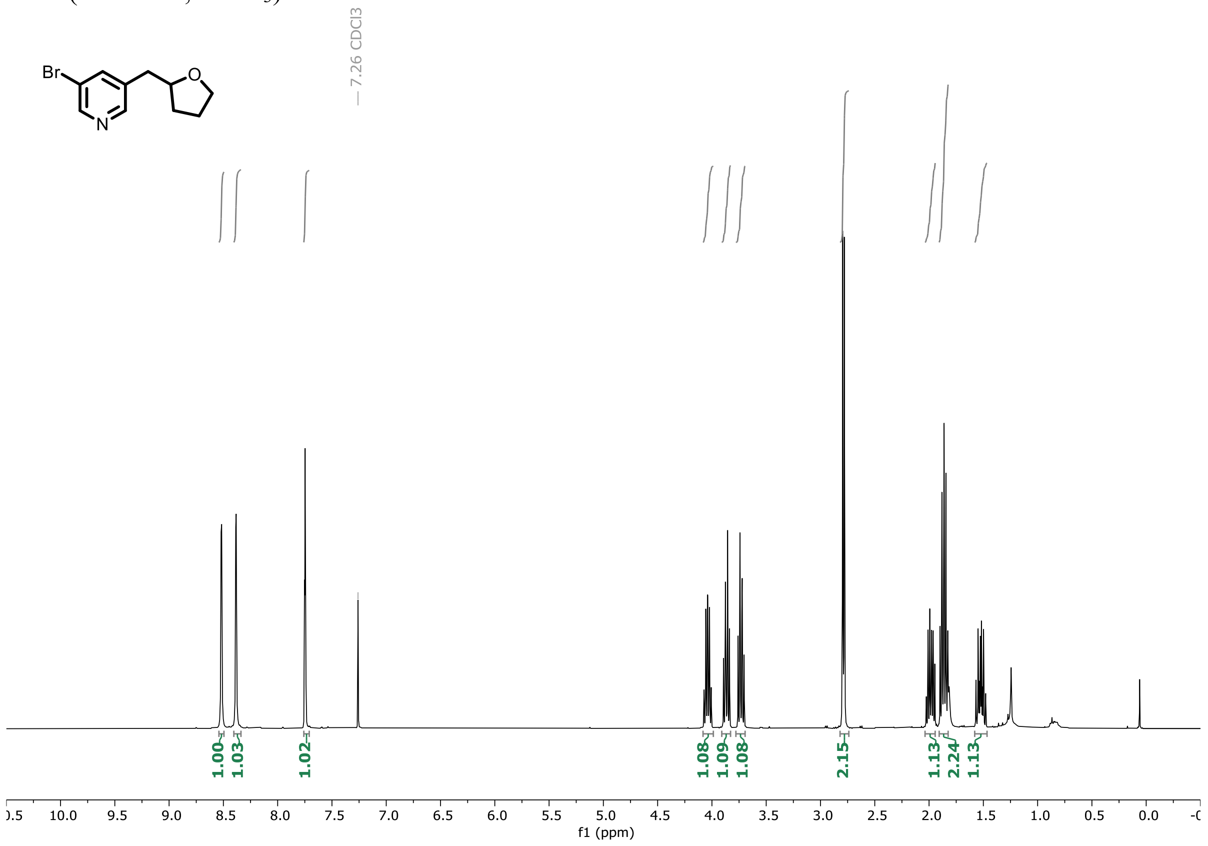

^{13}C NMR (101 MHz, $CDCl_3$) of **24**

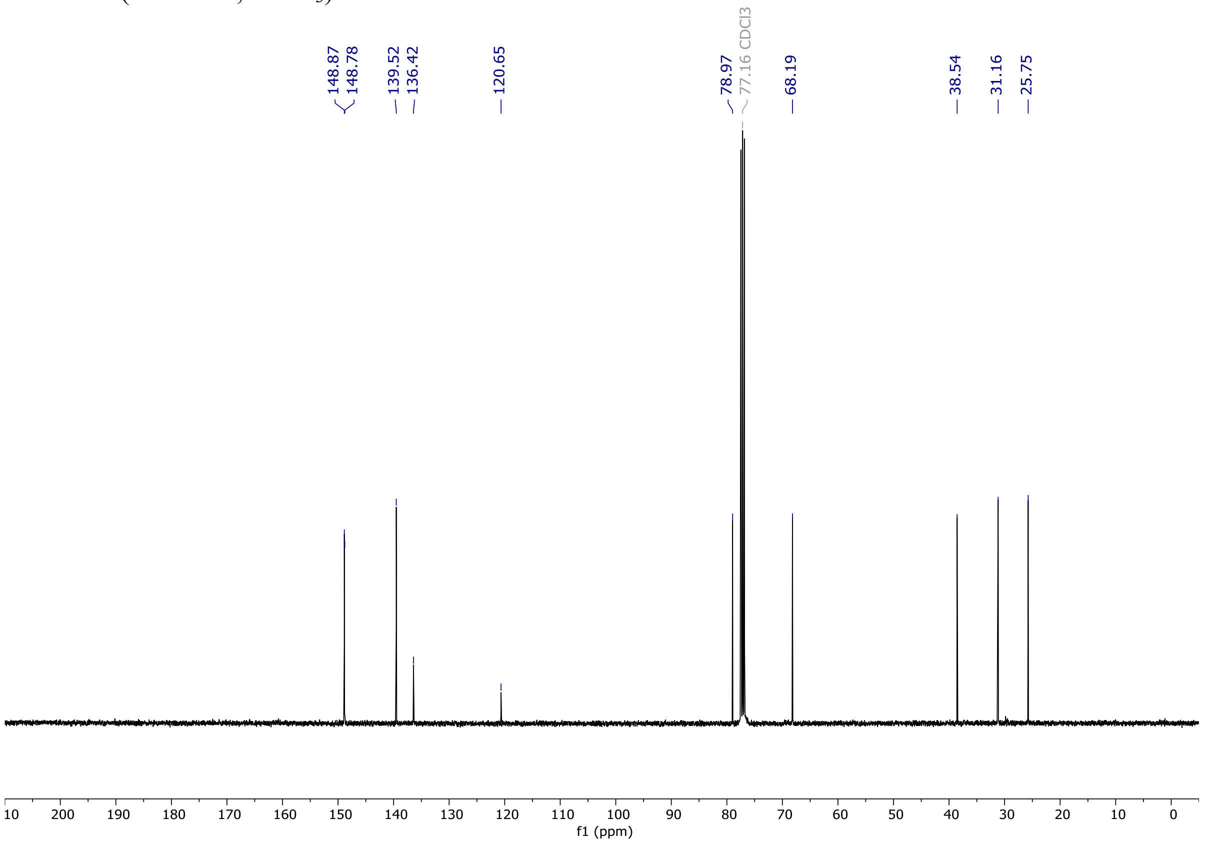

^{1}H NMR (400 MHz, $CDCl_3$) of **25**

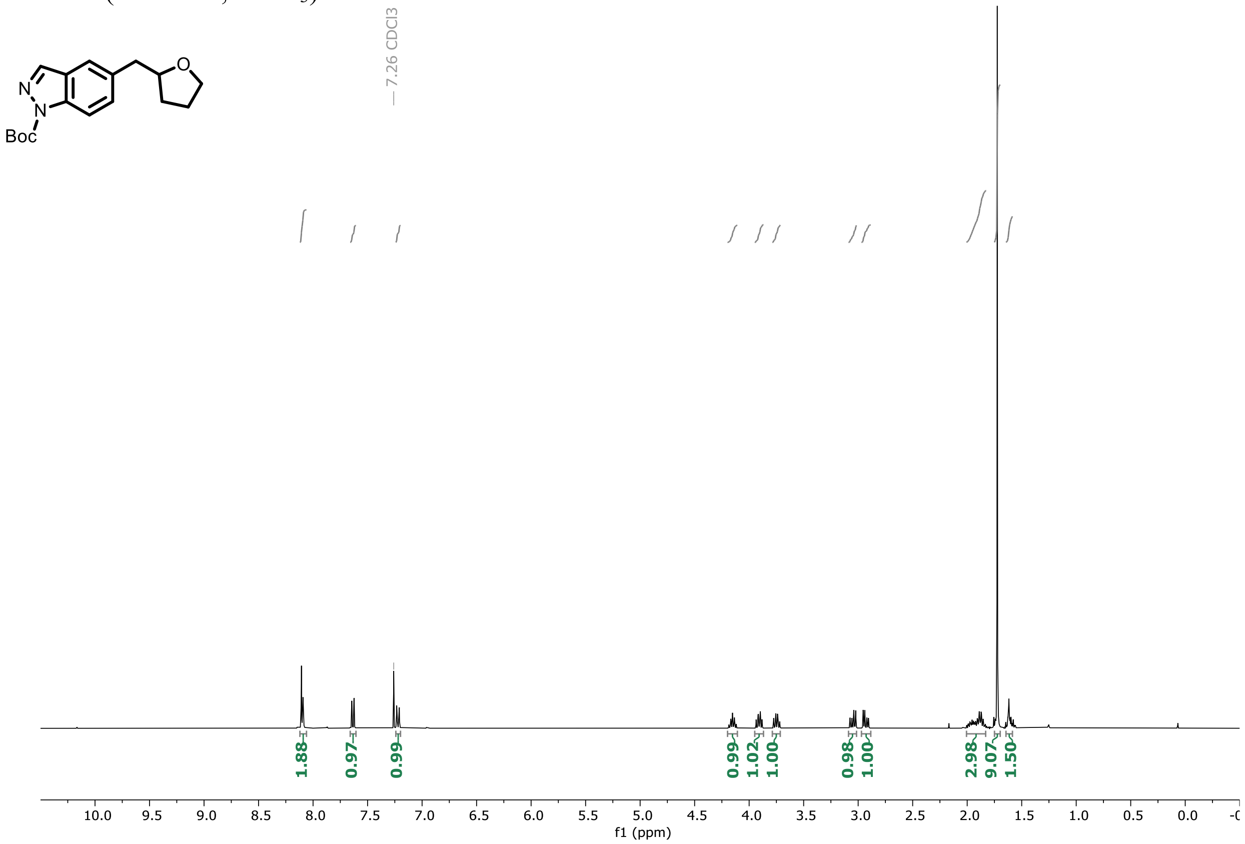

^{13}C NMR (101 MHz, $CDCl_3$) of **25**

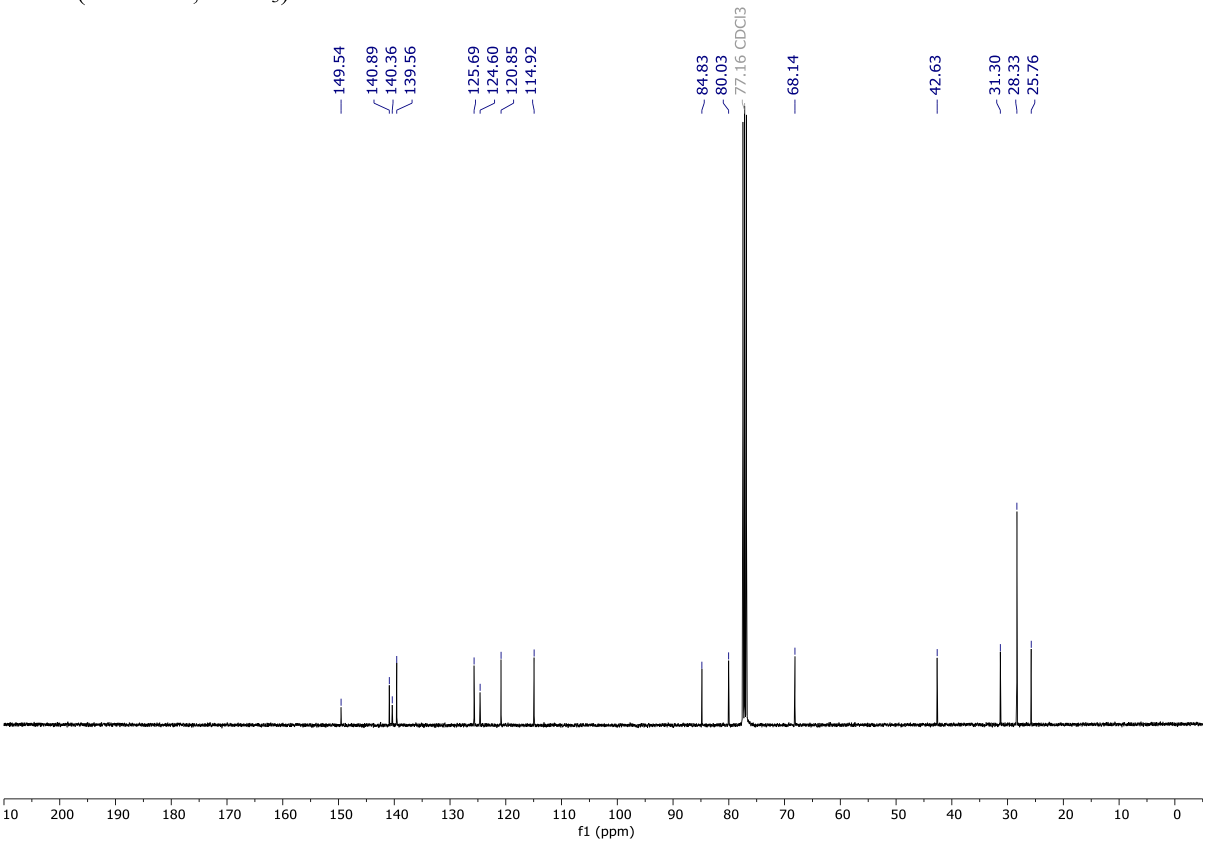

^{1}H NMR (400 MHz, $CDCl_3$) of **26**

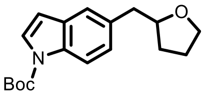

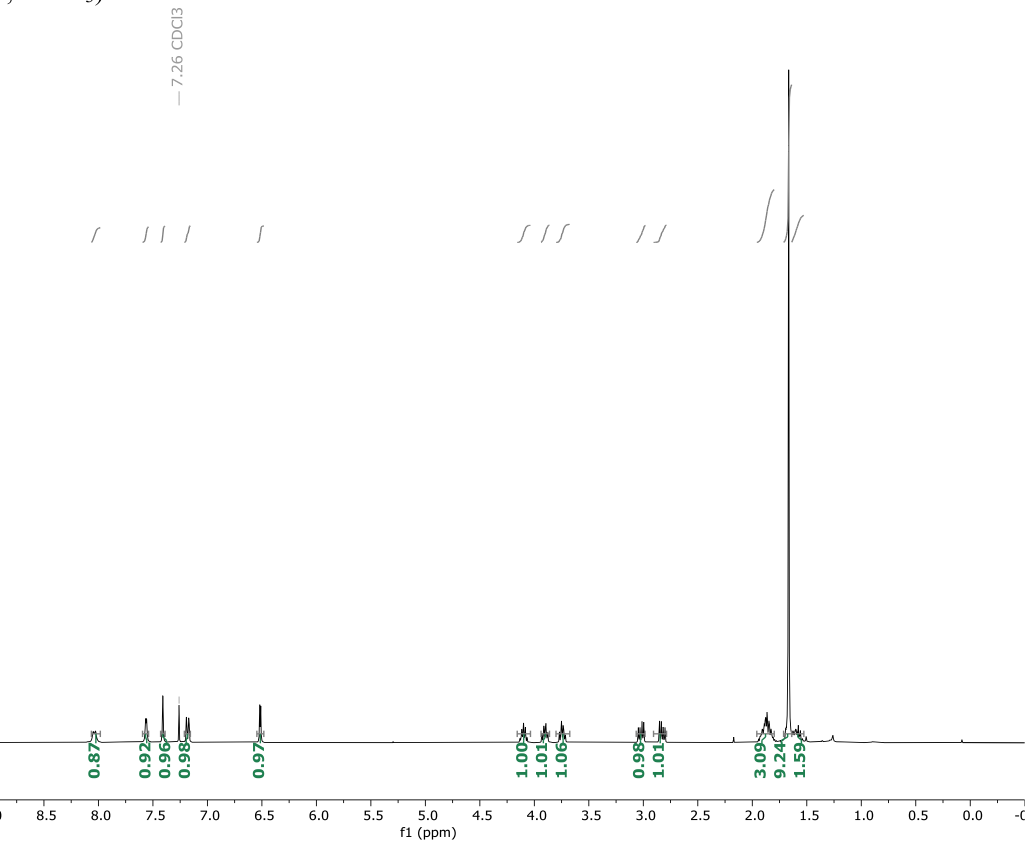

^{13}C NMR (101 MHz, $CDCl_3$) of **26**

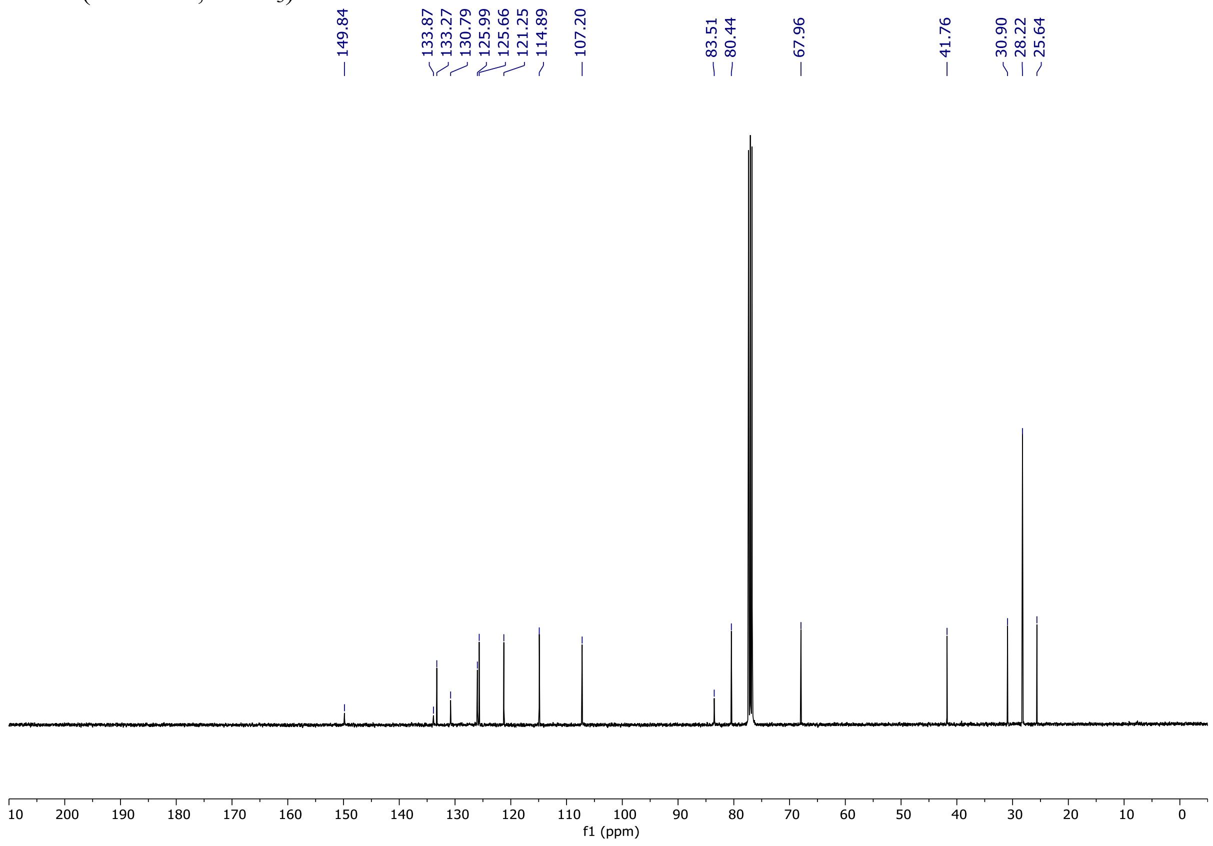

^{1}H NMR (400 MHz, $CDCl_3$) of **27**

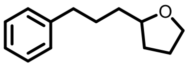

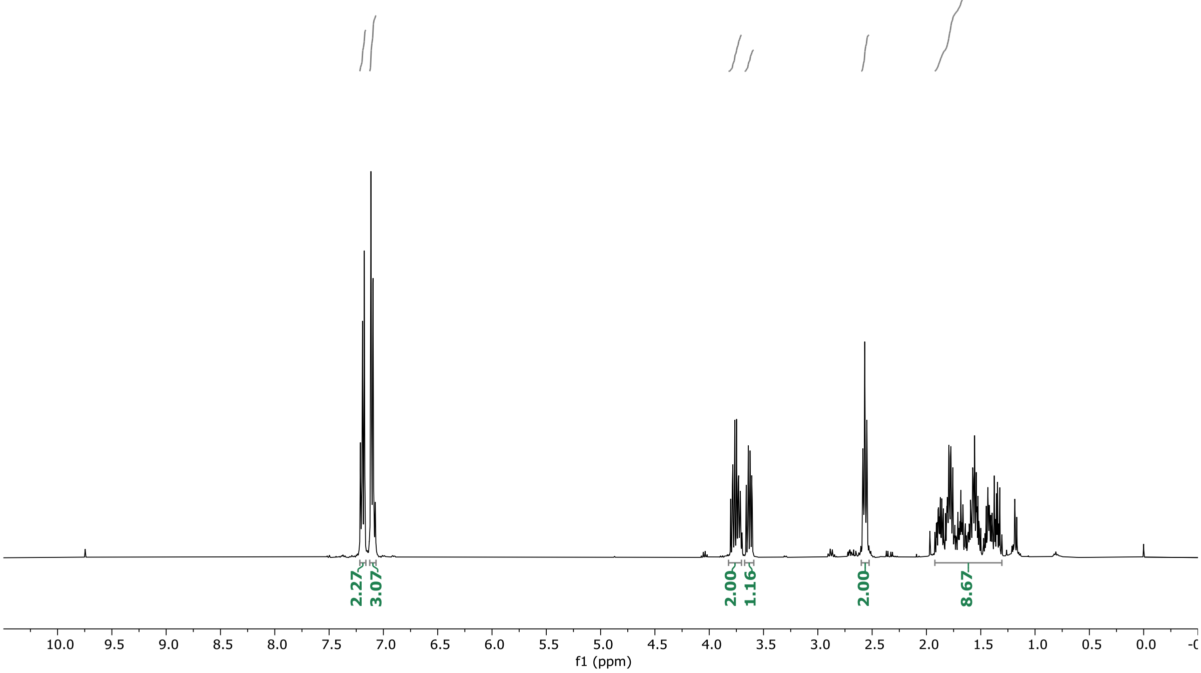

^{13}C NMR (101 MHz, $CDCl_3$) of **27**

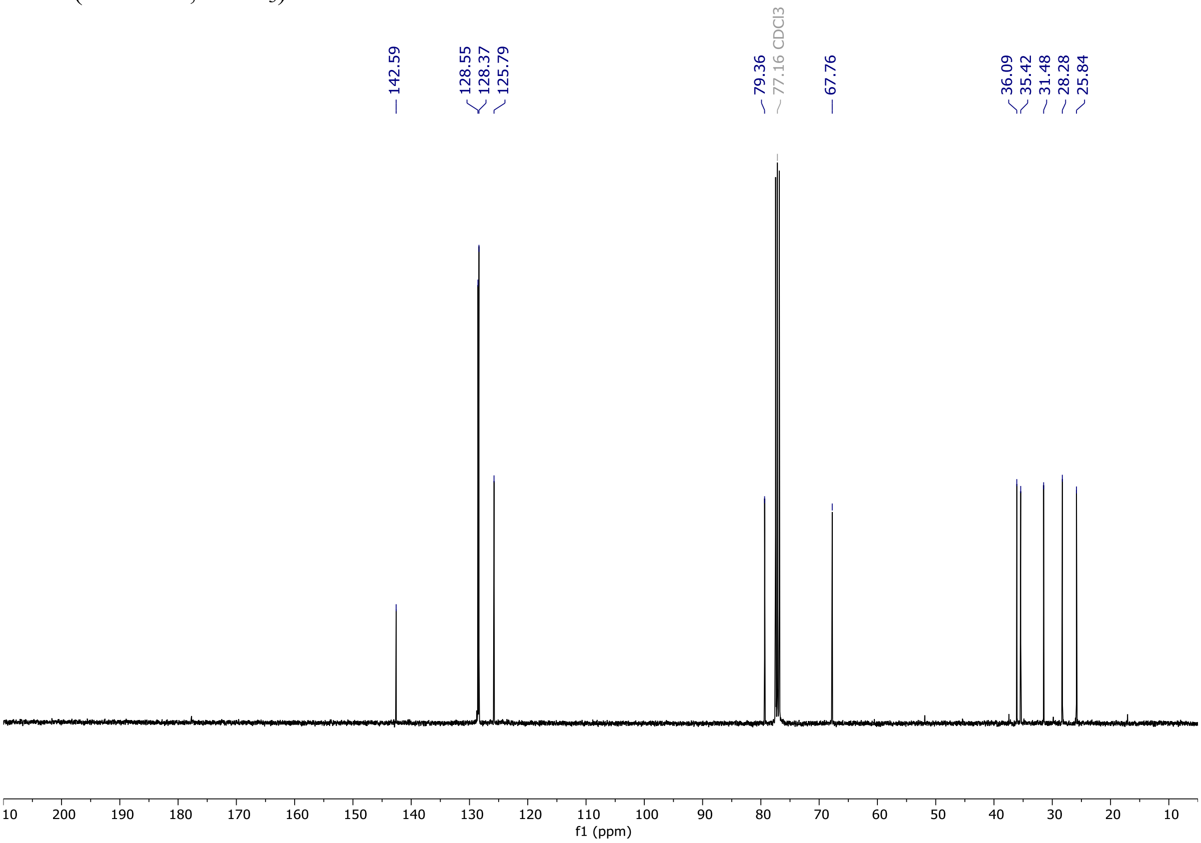

^{1}H NMR (400 MHz, $CDCl_3$) of **28**

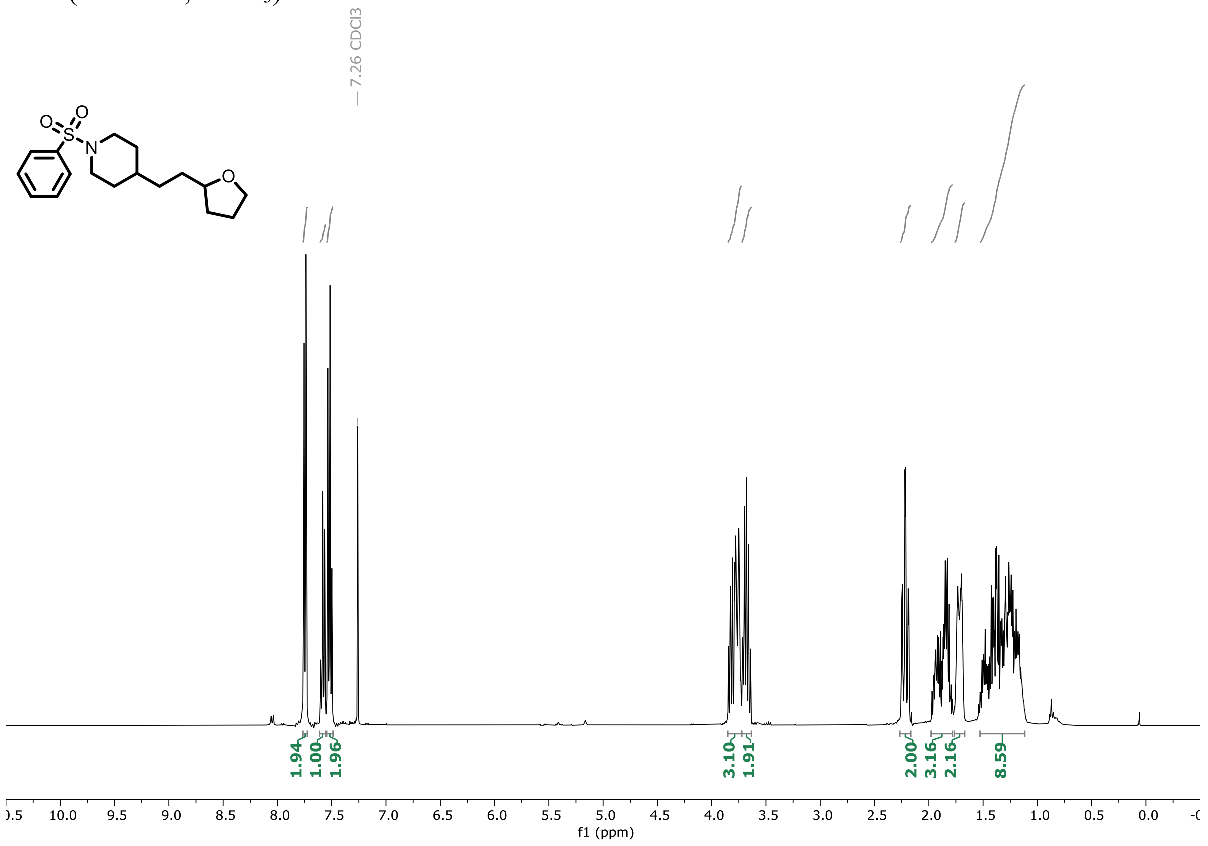

^{13}C NMR (101 MHz, $CDCl_3$) of **28**

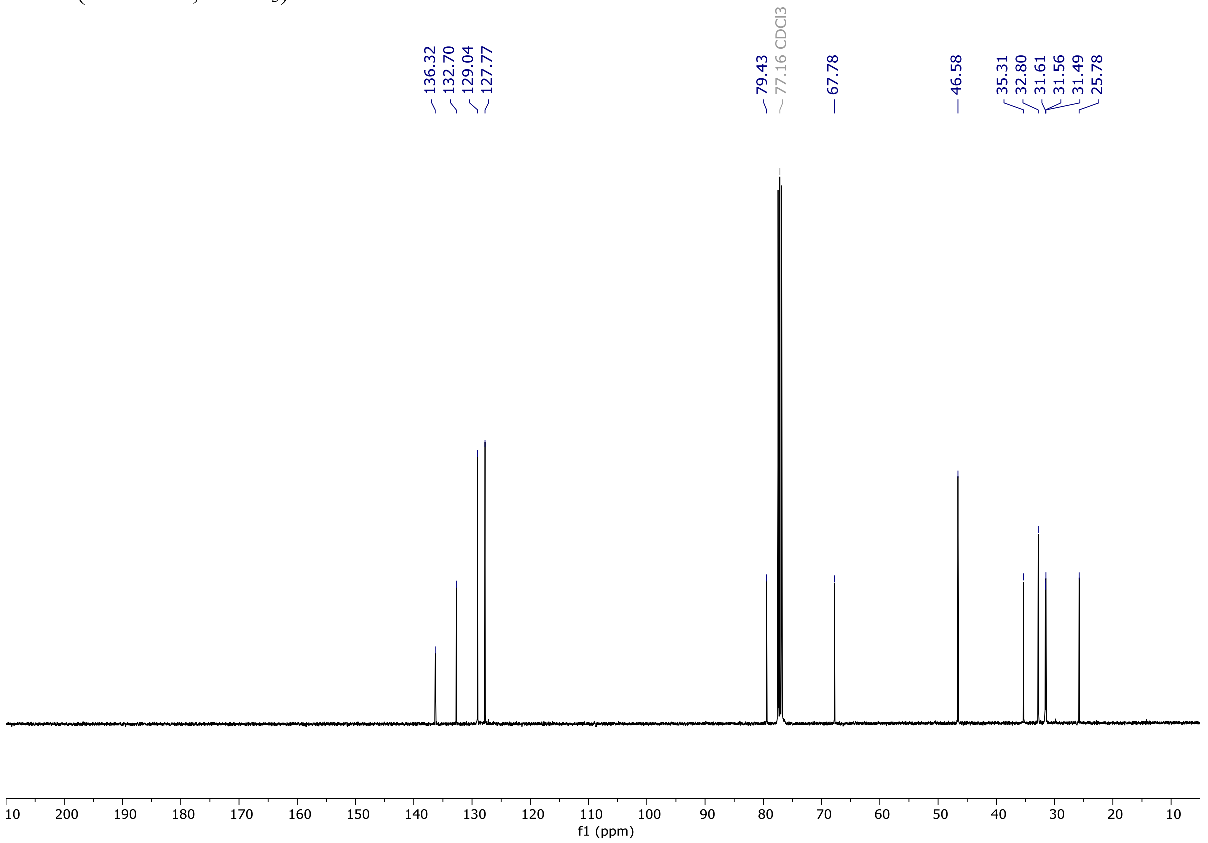

^{1}H NMR (400 MHz, $CDCl_3$) of **29**

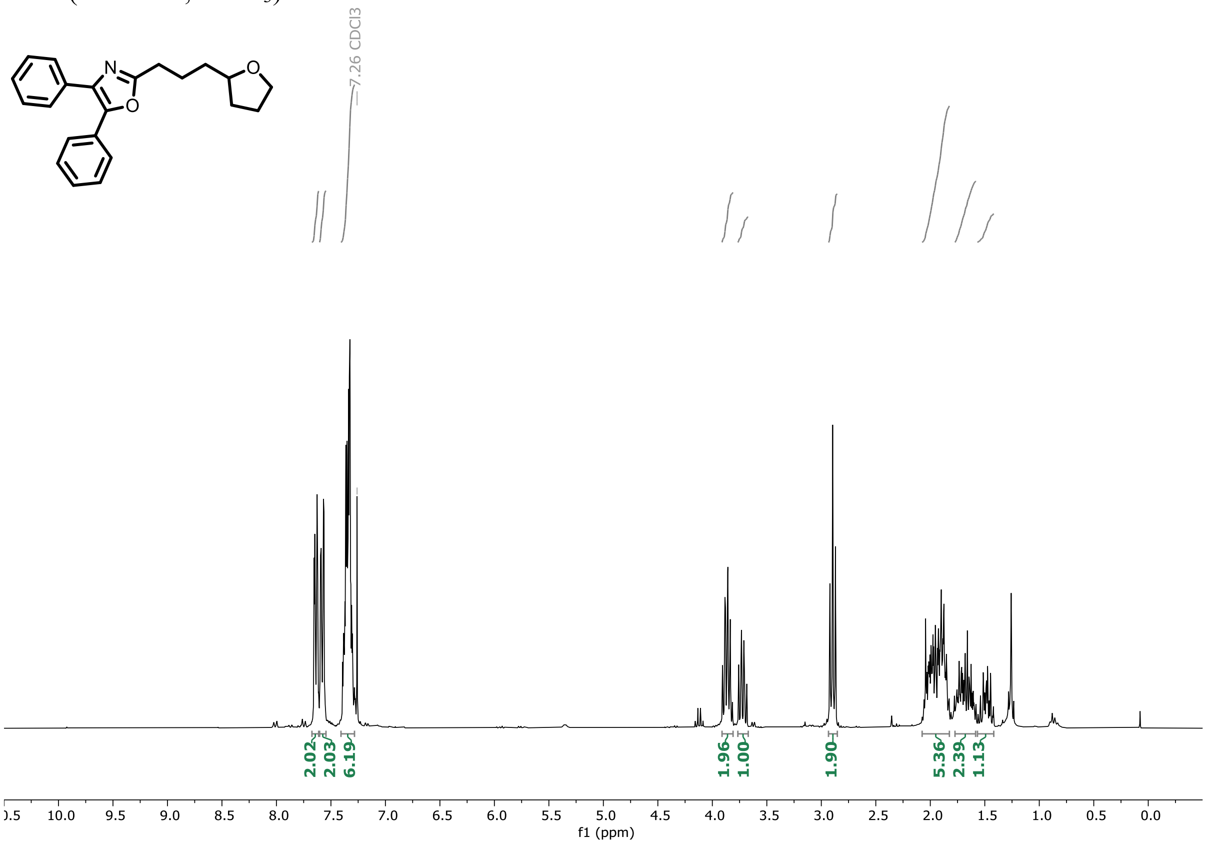

^{13}C NMR (101 MHz, $CDCl_3$) of **29**

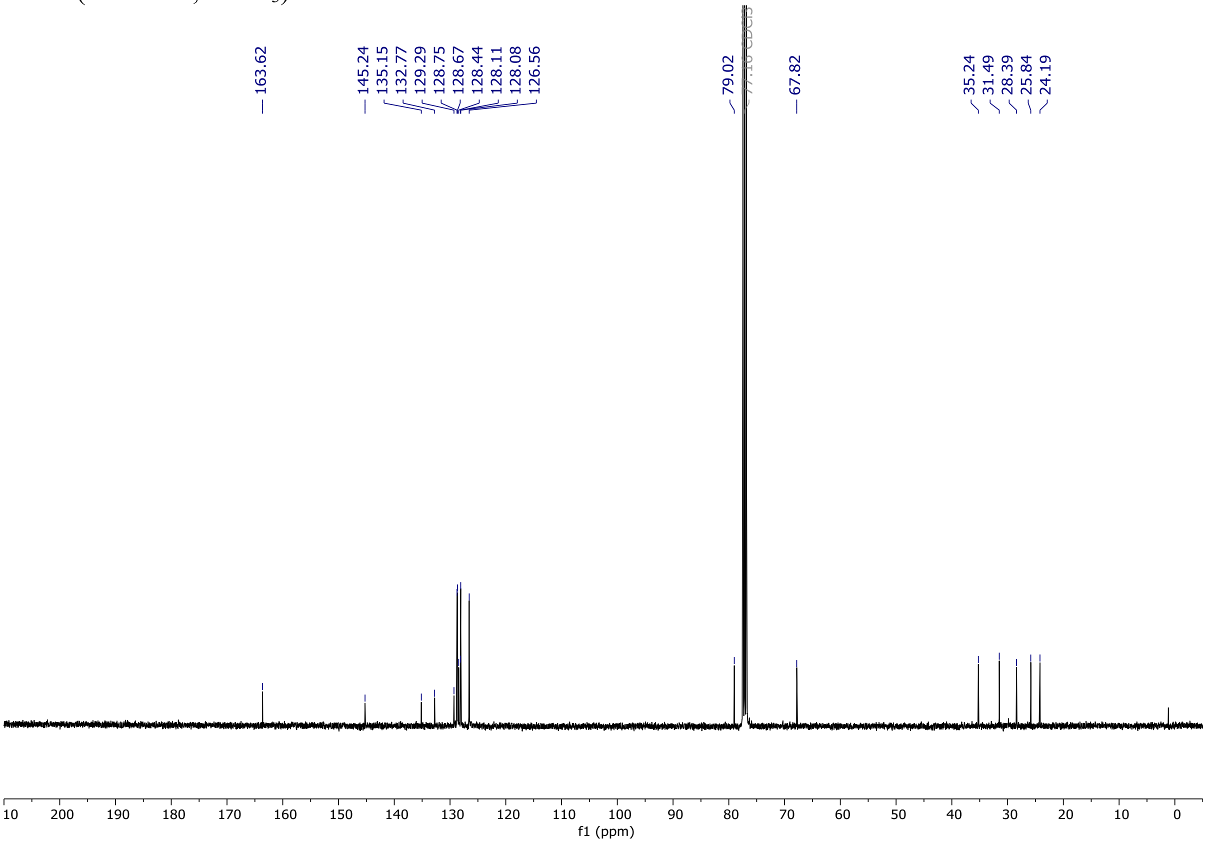

^{1}H NMR (400 MHz, $CDCl_3$) of **30**

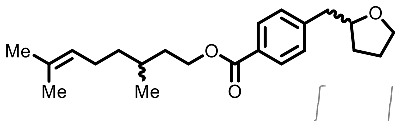

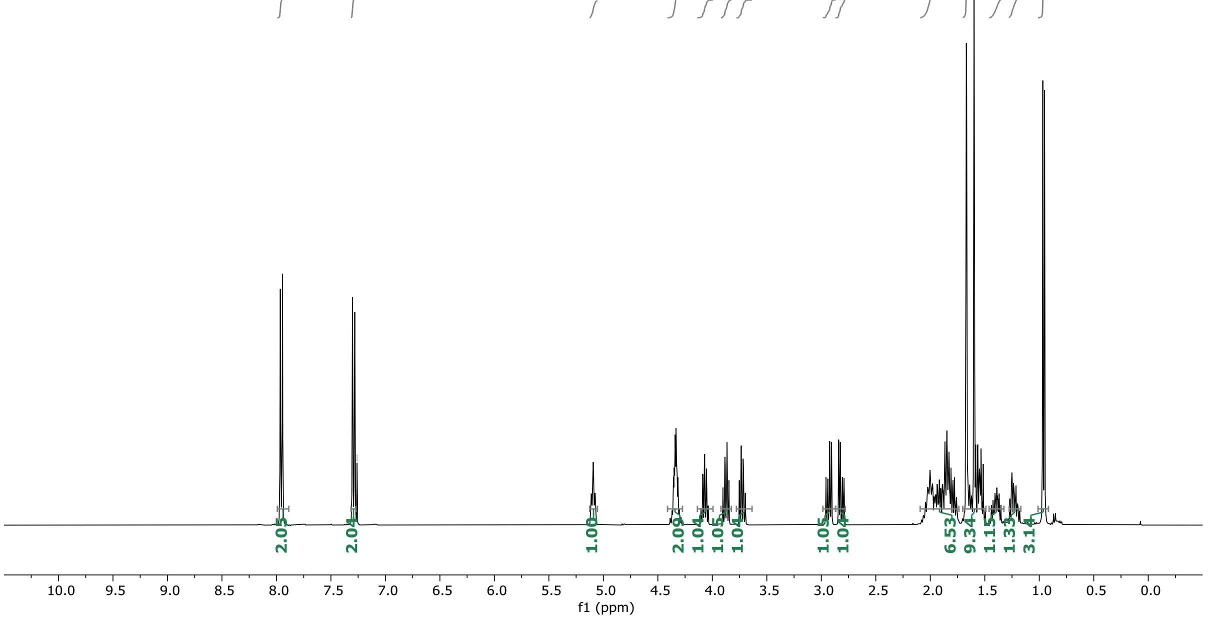

^{13}C NMR (101 MHz, $CDCl_3$) of **30**

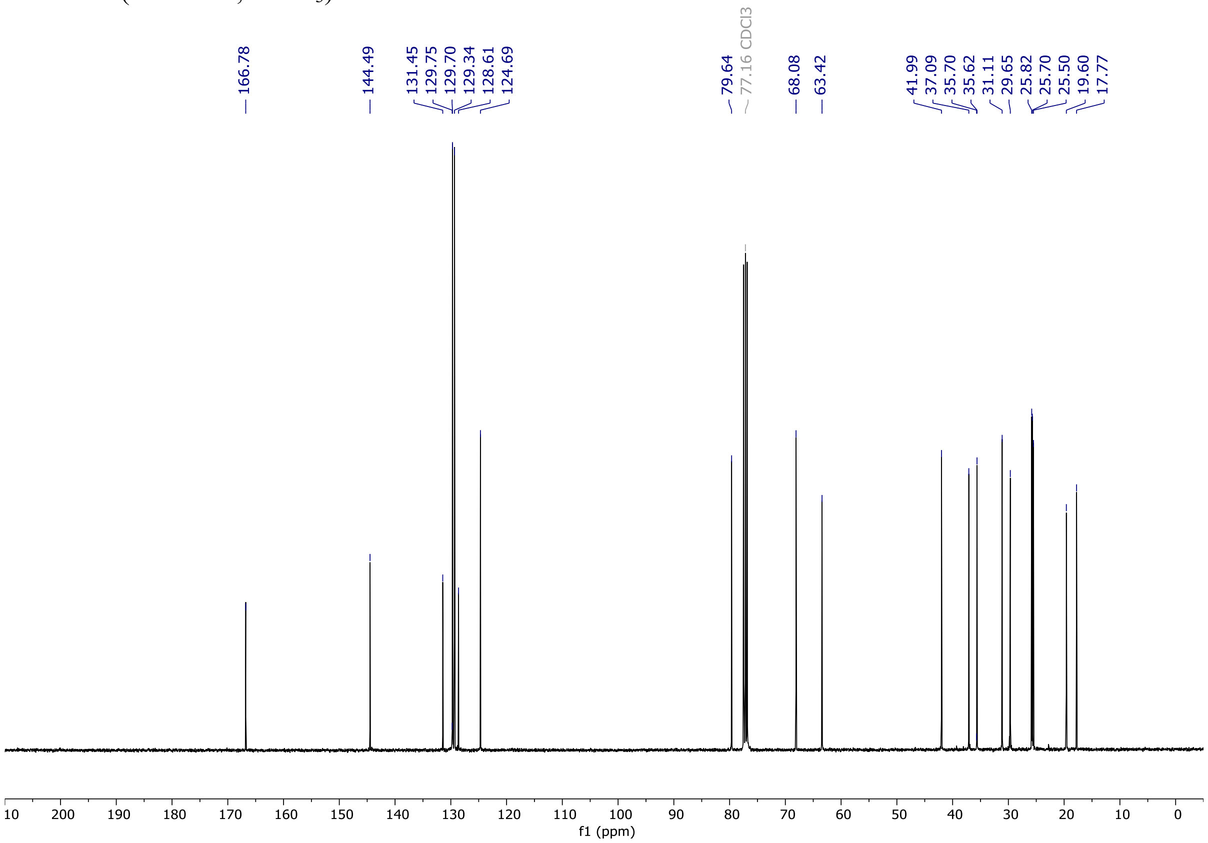

^{1}H NMR (400 MHz, $CDCl_3$) of **31**

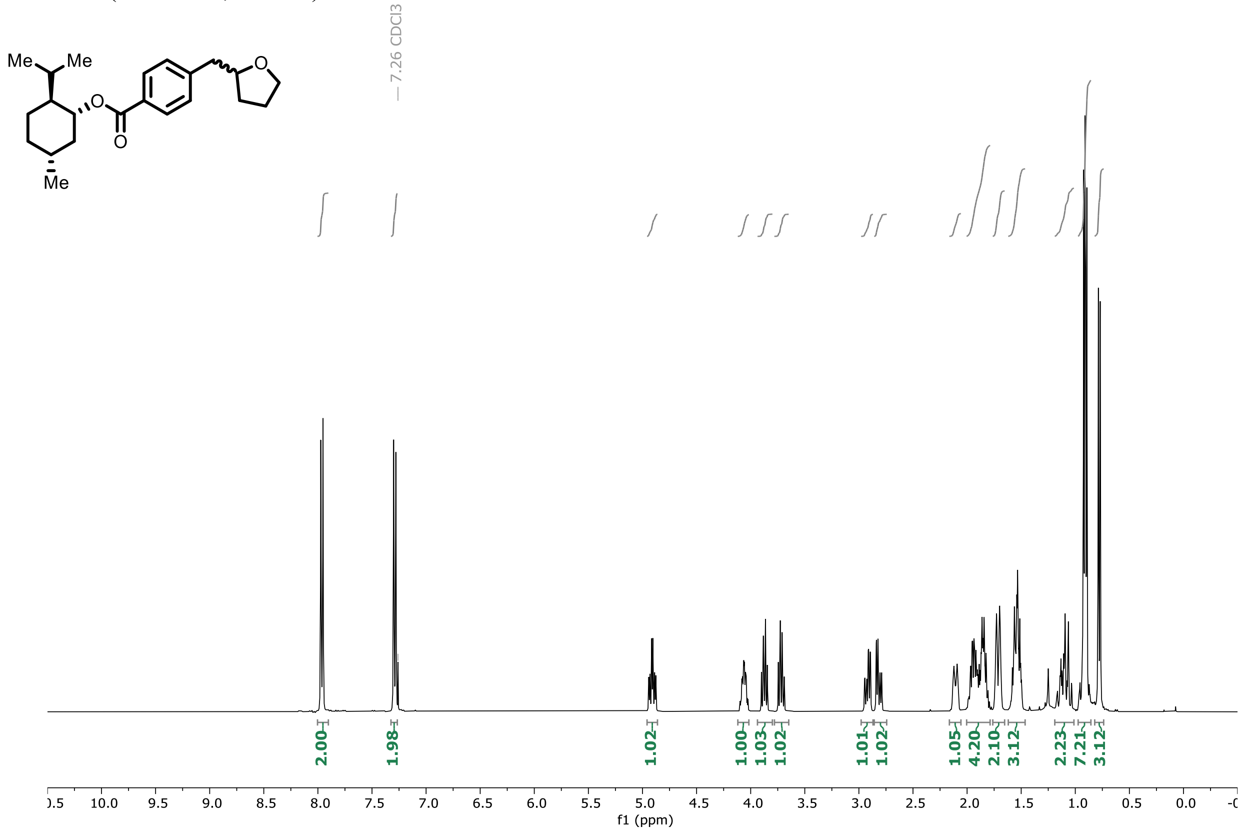

^{13}C NMR (101 MHz, $CDCl_3$) of **31**

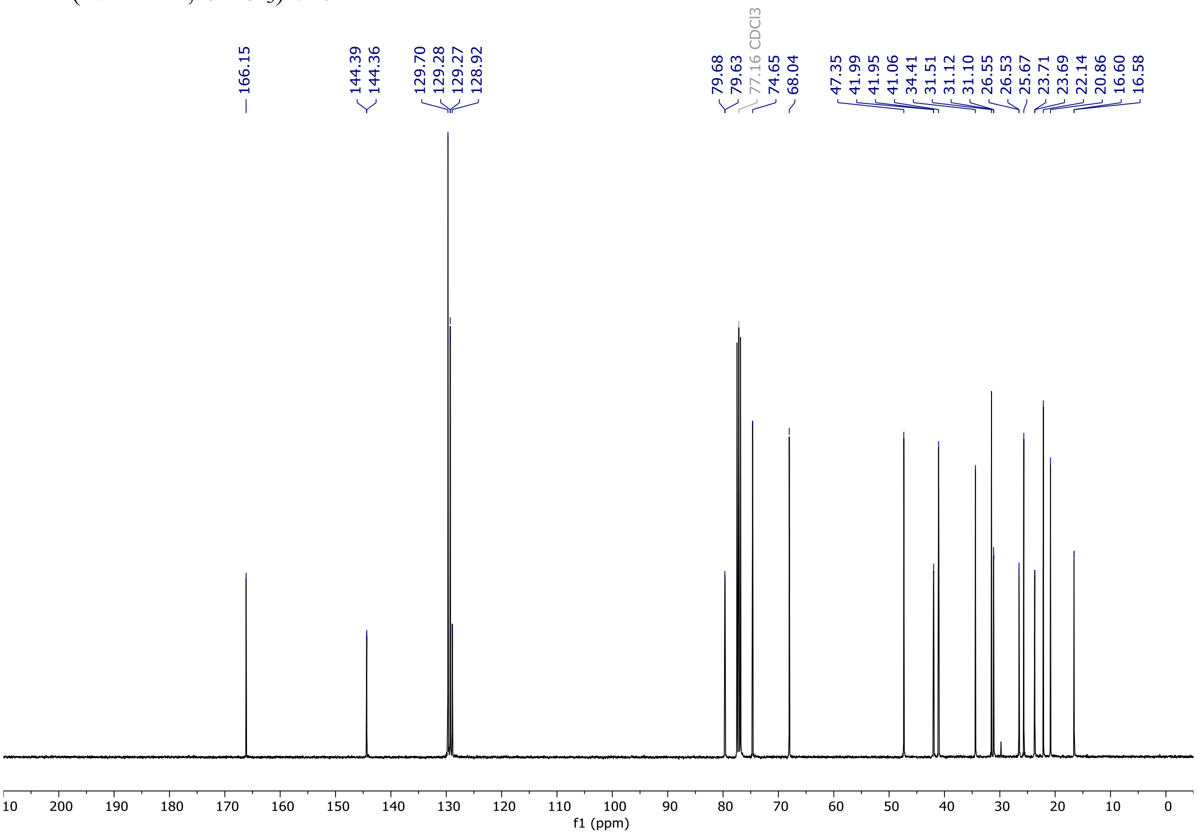

^{1}H NMR (400 MHz, $CDCl_3$) of **32**

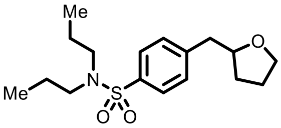

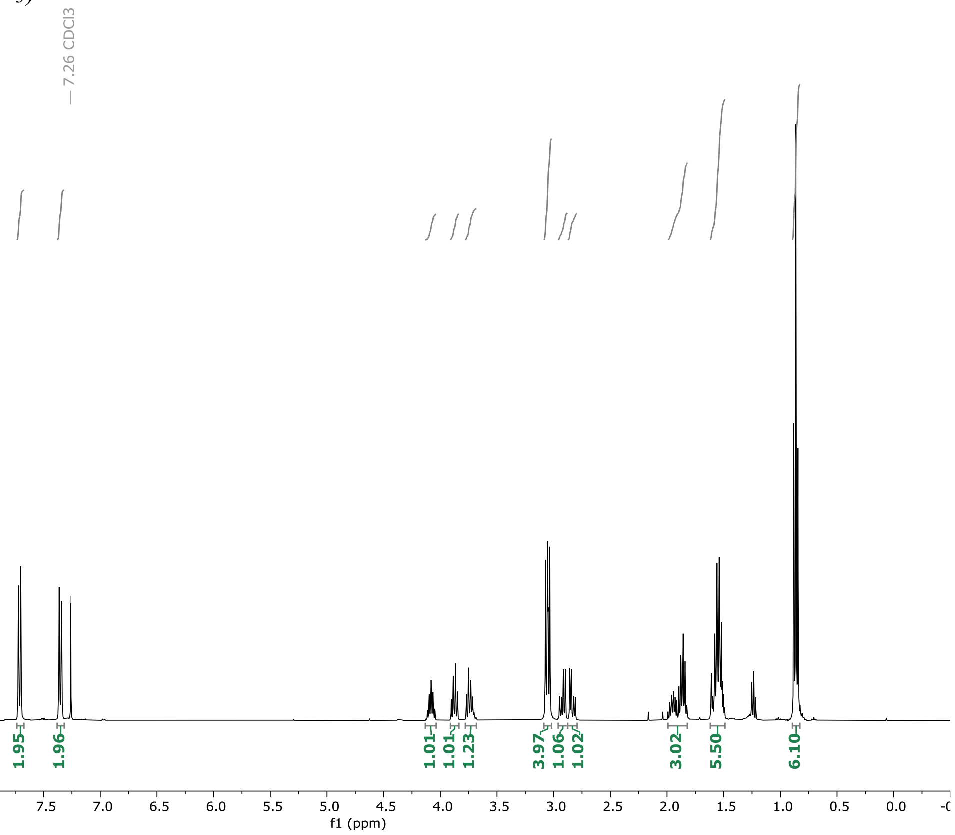

^{13}C NMR (101 MHz, $CDCl_3$) of **32**

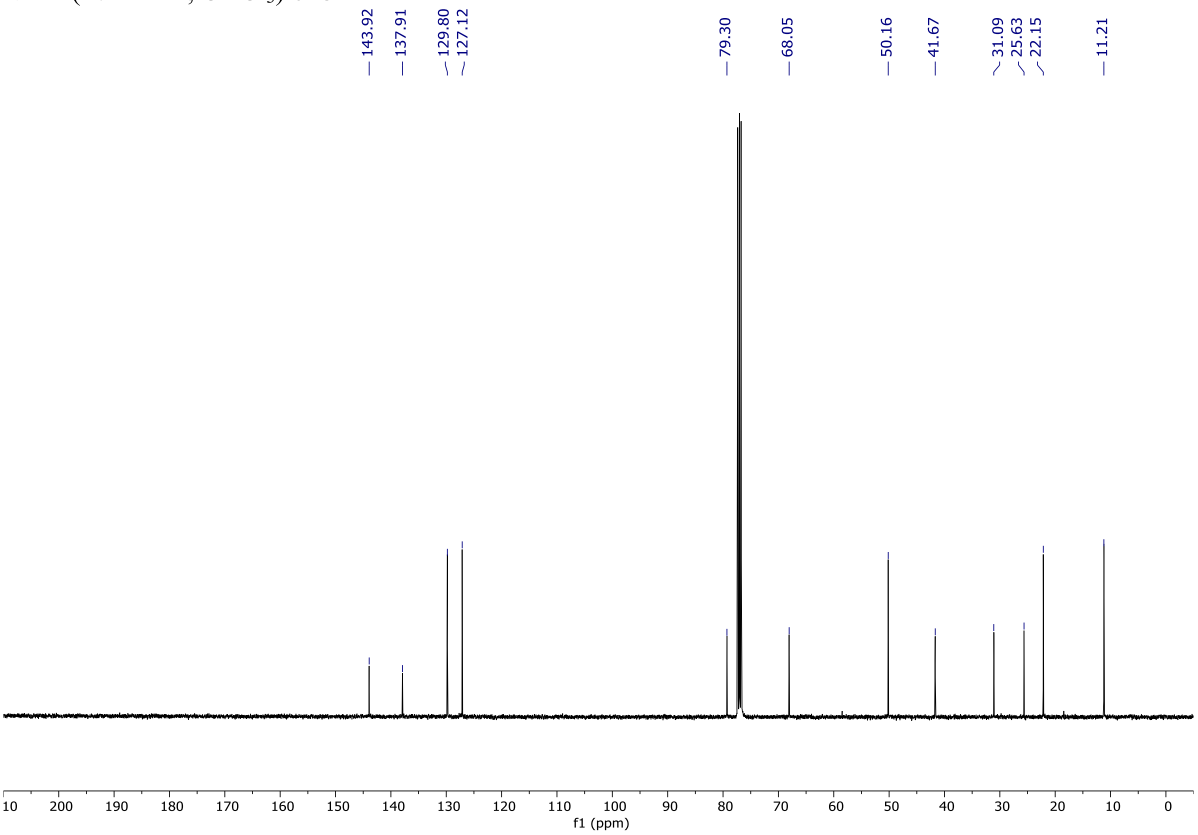

^{1}H NMR (400 MHz,$CDCl_3$) of **33**

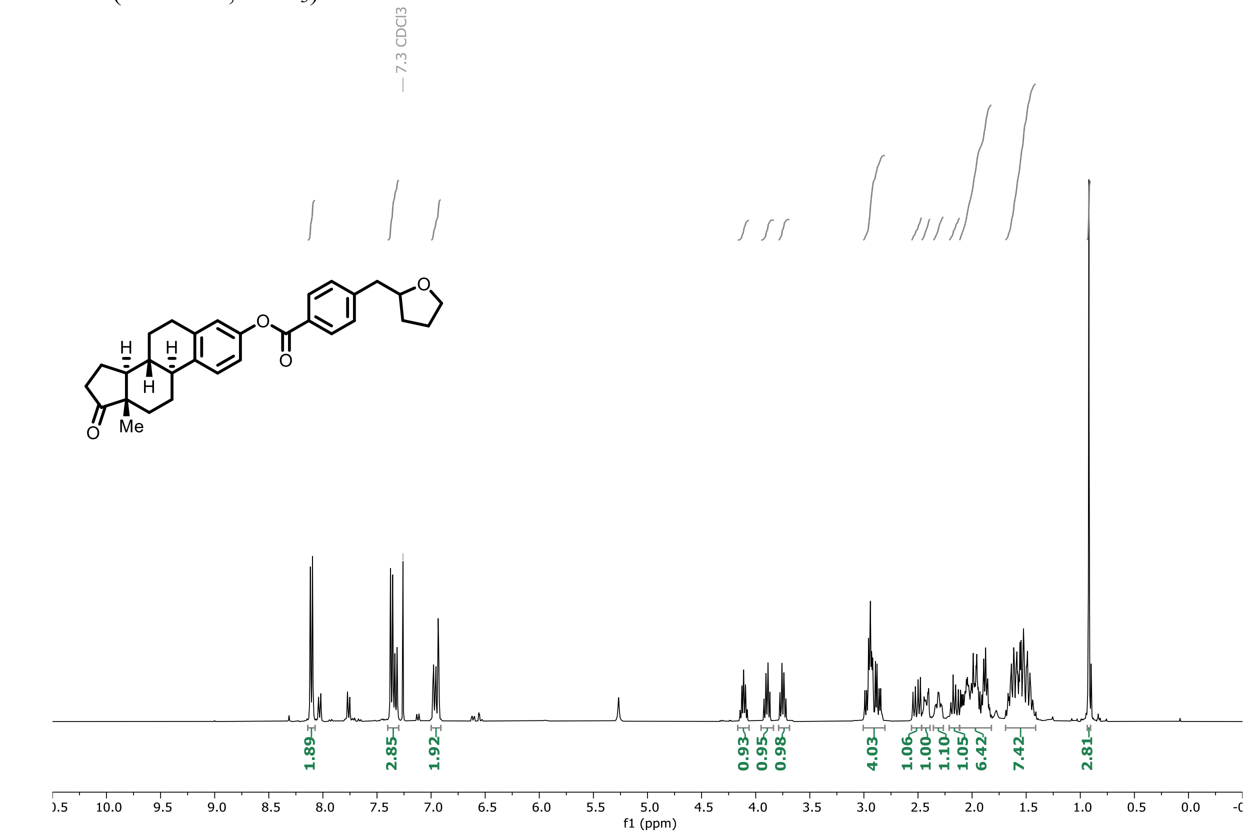

^{13}C NMR (101 MHz, $CDCl_3$) of **33**

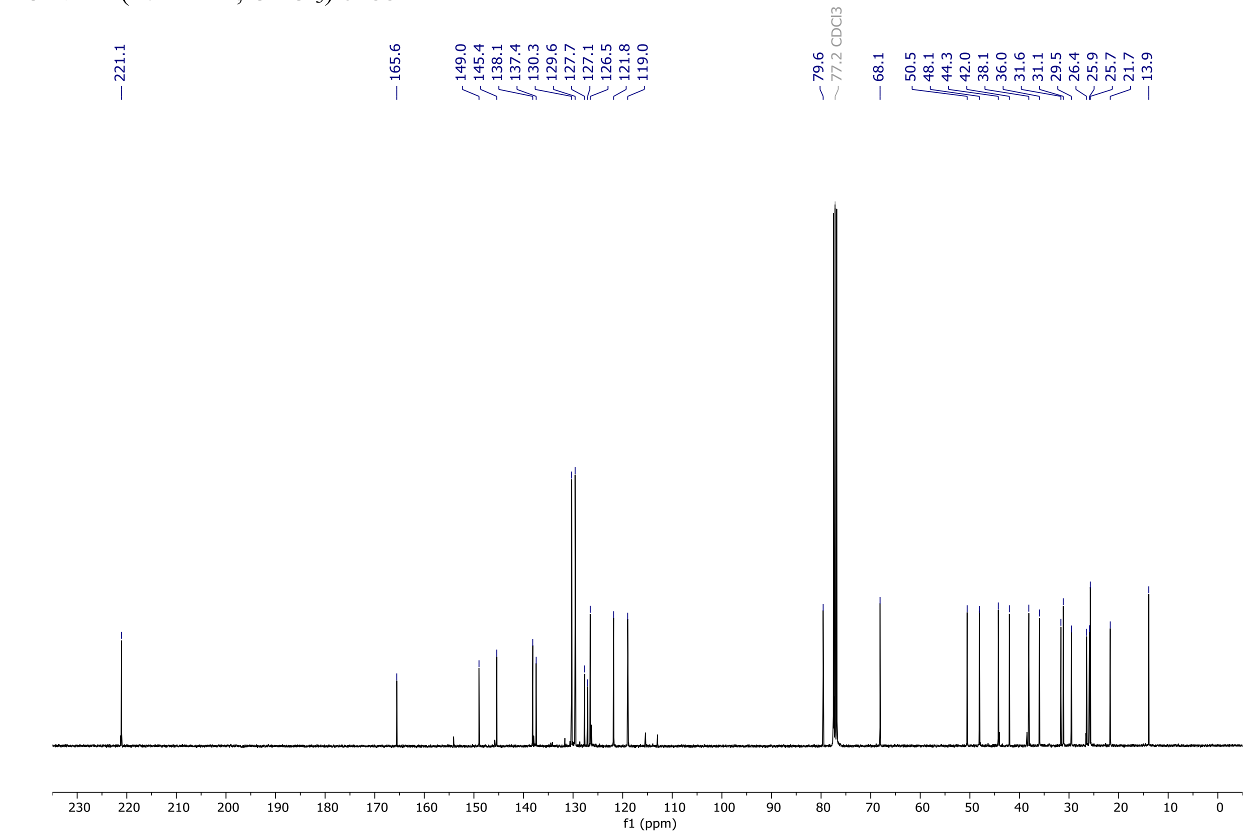

^{1}H NMR (400 MHz, $CDCl_3$) of **34**

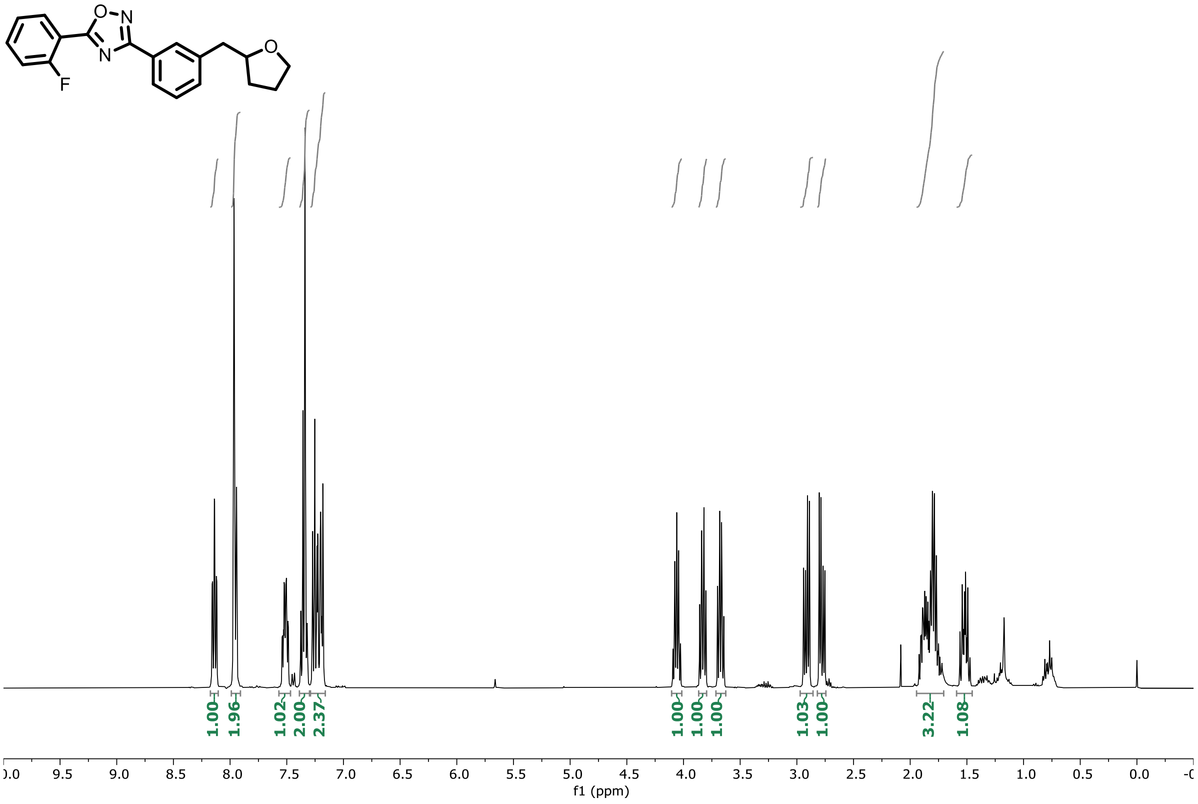

^{13}C NMR (101 MHz, $CDCl_3$) of **34**

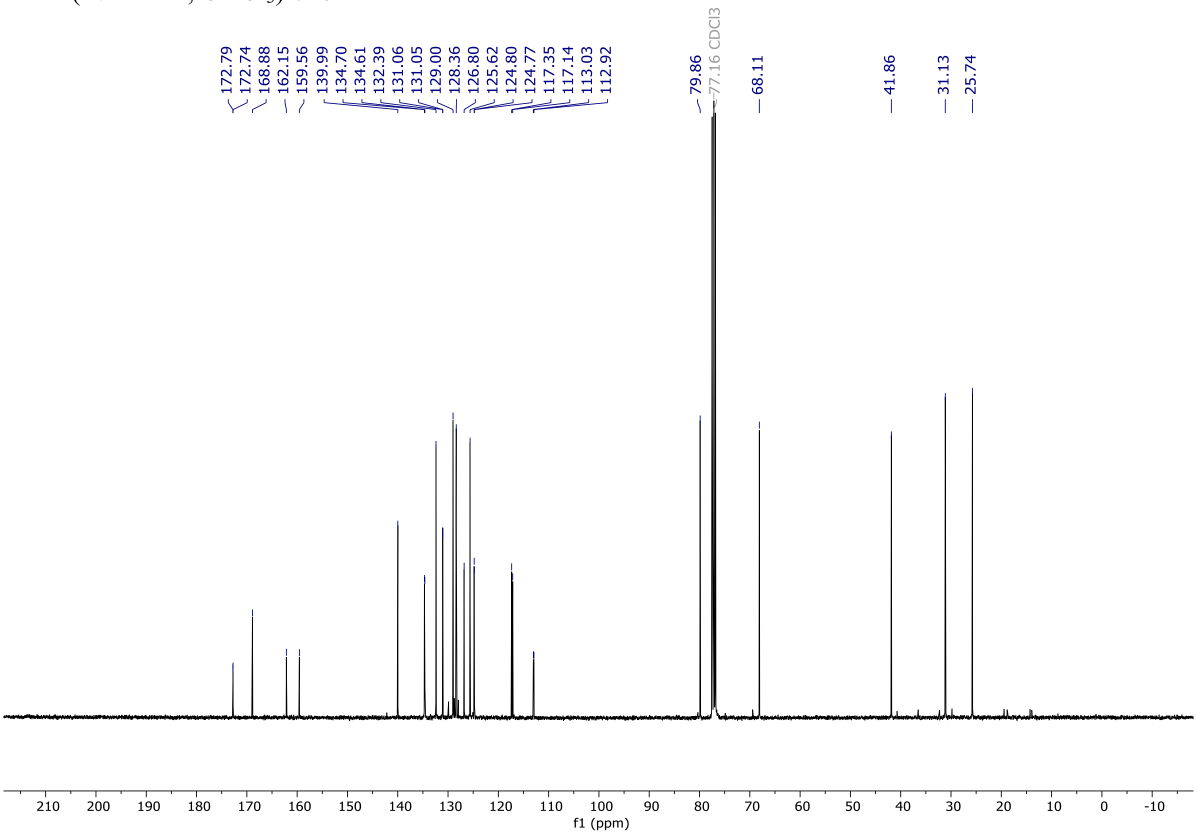

^{19}F NMR (282 MHz, $CDCl_3$) of **34**

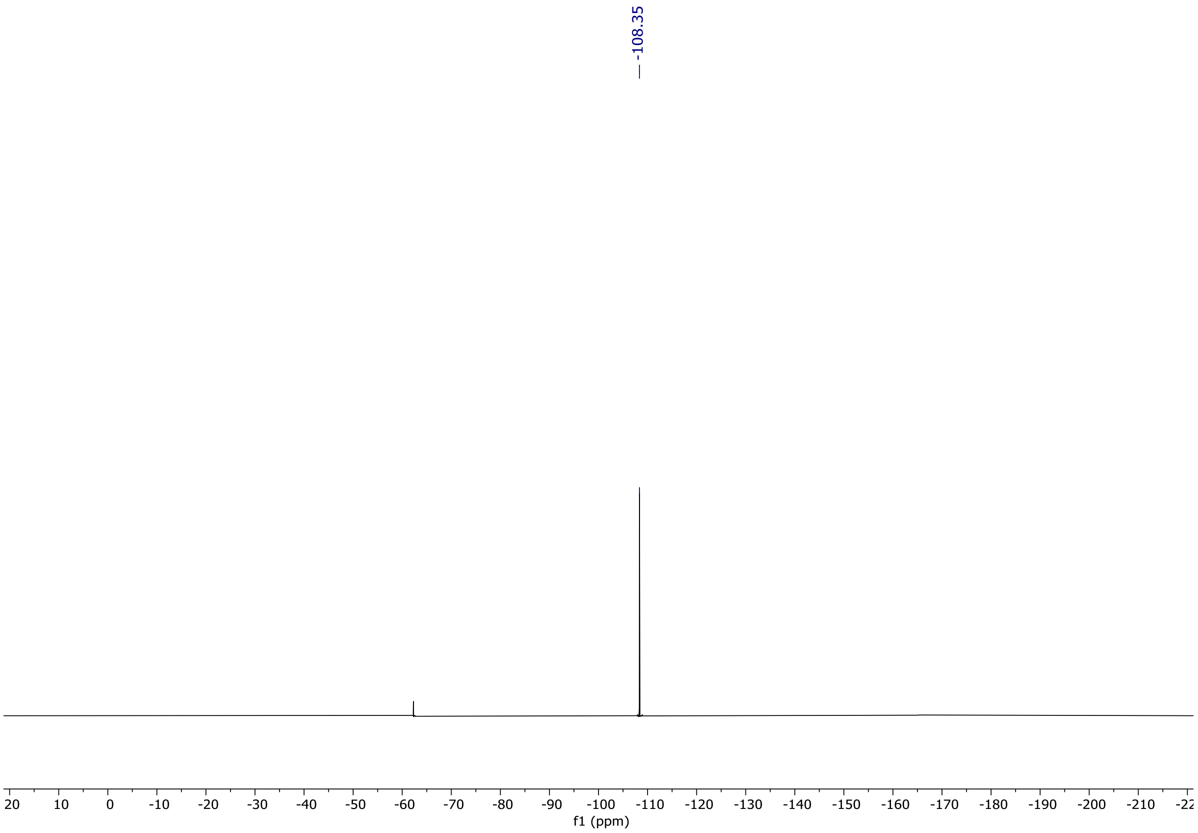
-108.35
20 10 0 -10 -20 -30 -40 -50 -60 -70 -80 -90 -100 -110 -120 -130 -140 -150 -160 -170 -180 -190 -200 -210
f1 (ppm)

^{1}H NMR (400 MHz, $CDCl_3$) of **35**

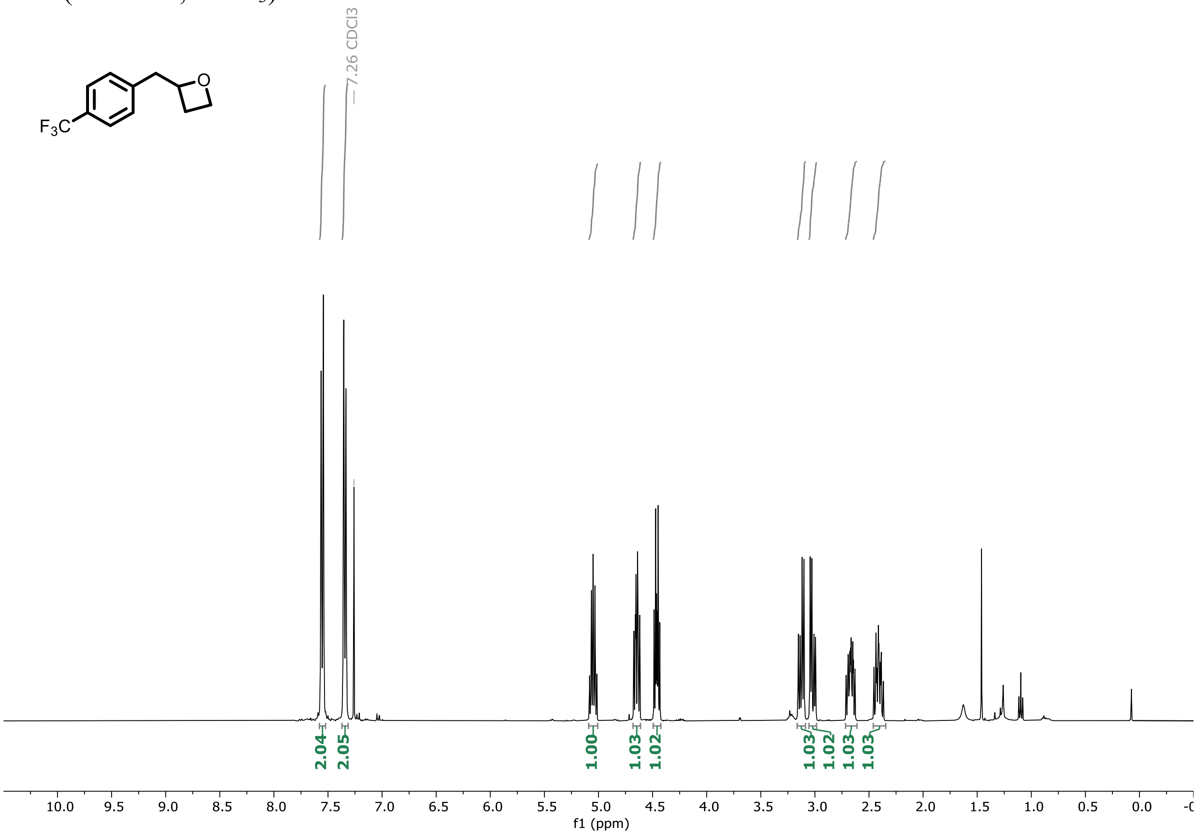

^{13}C NMR (101 MHz, $CDCl_3$) of **35**

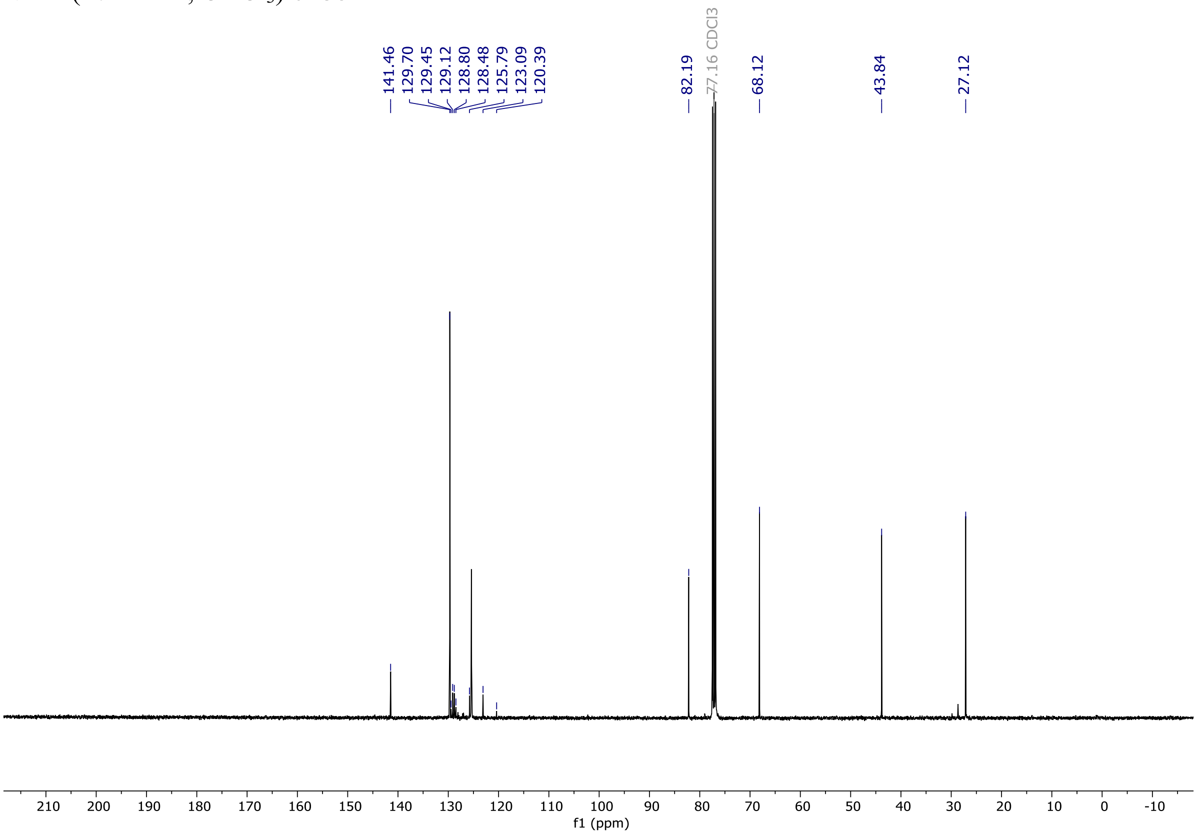

^{19}F NMR (282 MHz, $CDCl_3$) of **35**

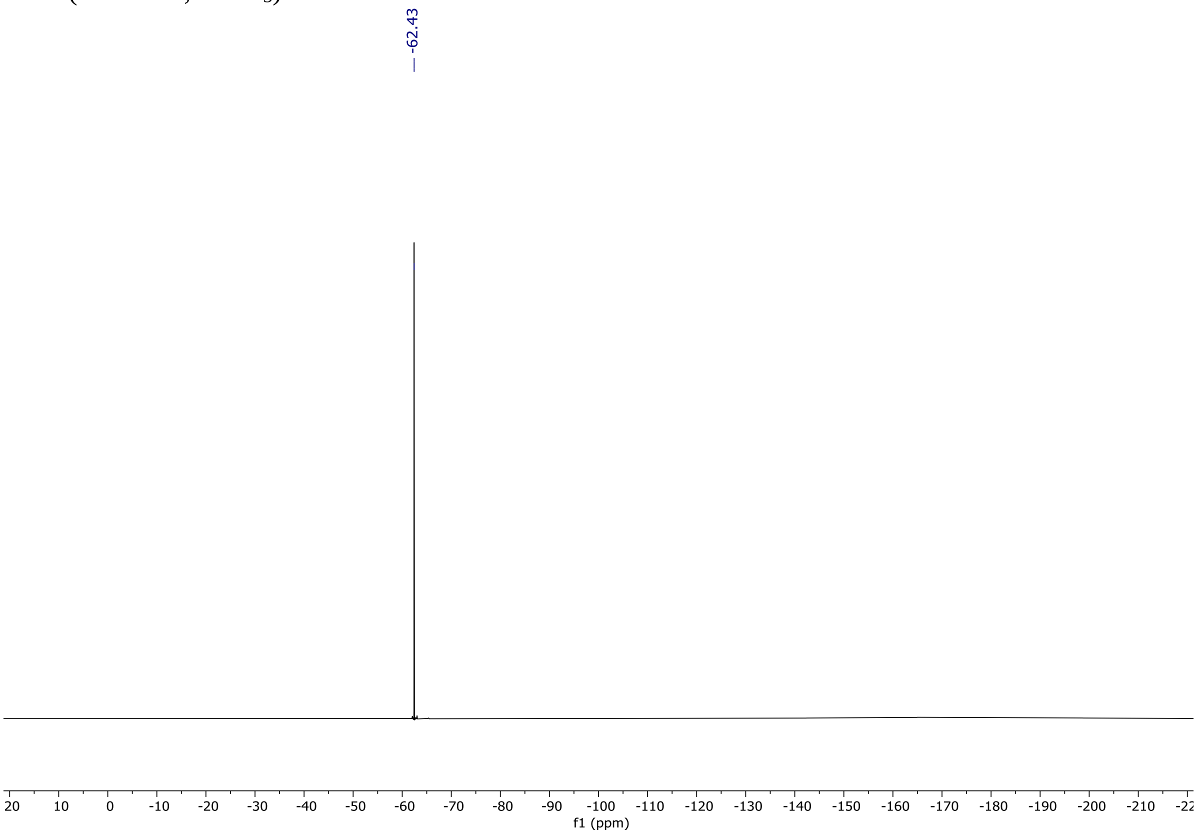

^{1}H NMR (400 MHz, $CDCl_3$) of **36**

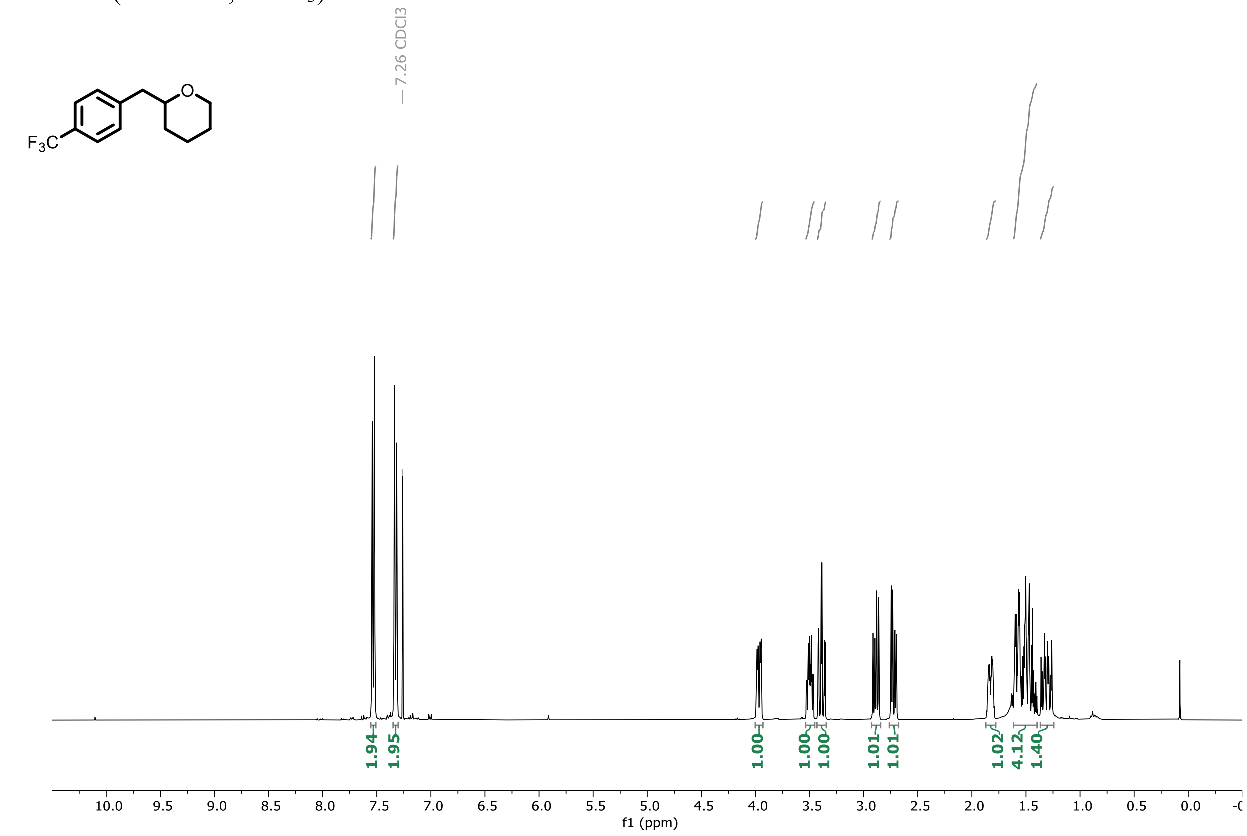

^{13}C NMR (101 MHz, $CDCl_3$) of **36**

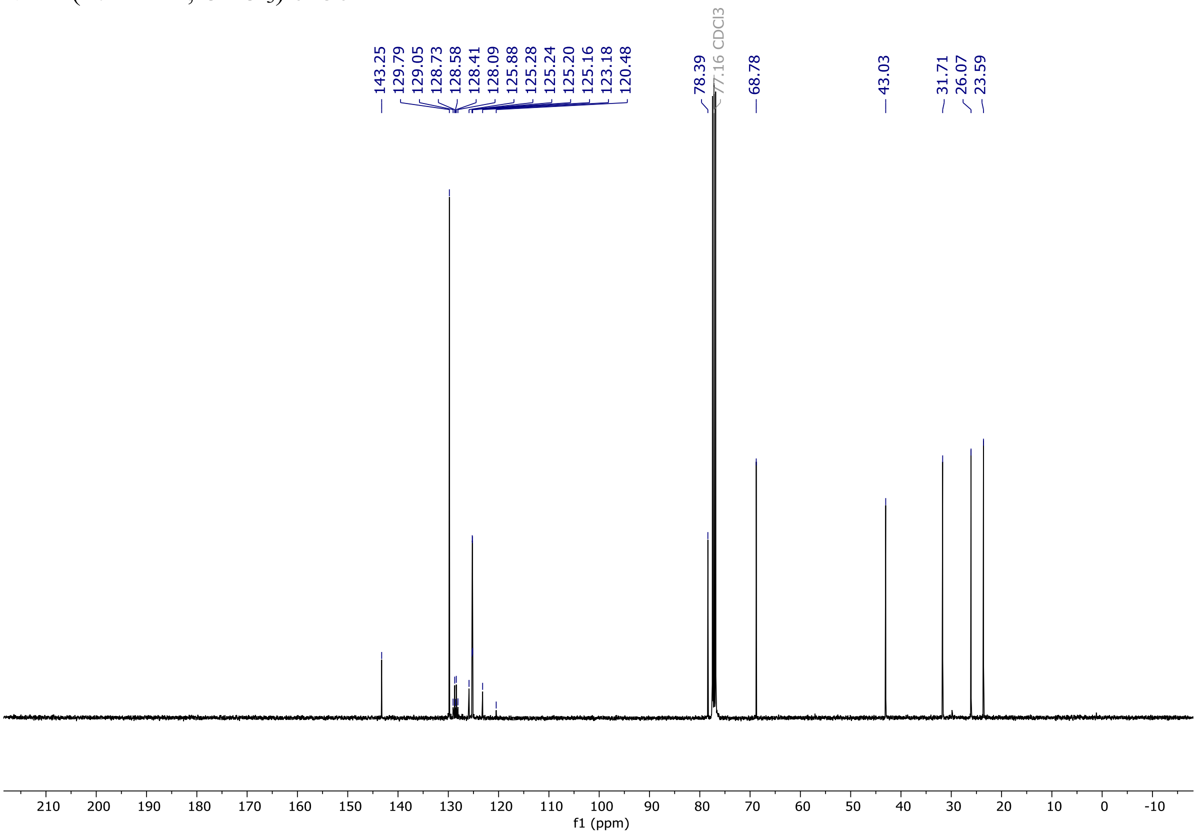

^{19}F NMR (282 MHz, $CDCl_3$) of **36**

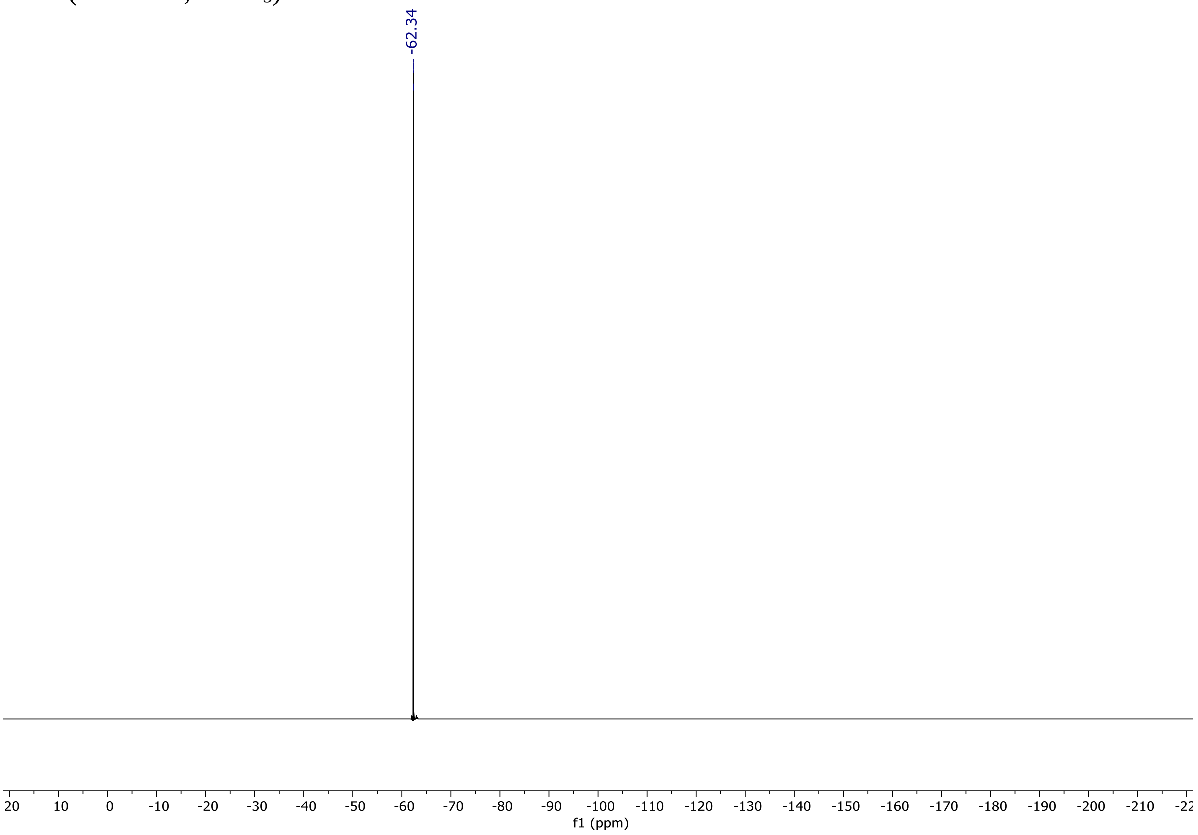

^{1}H NMR (400 MHz, $CDCl_3$) of **37**

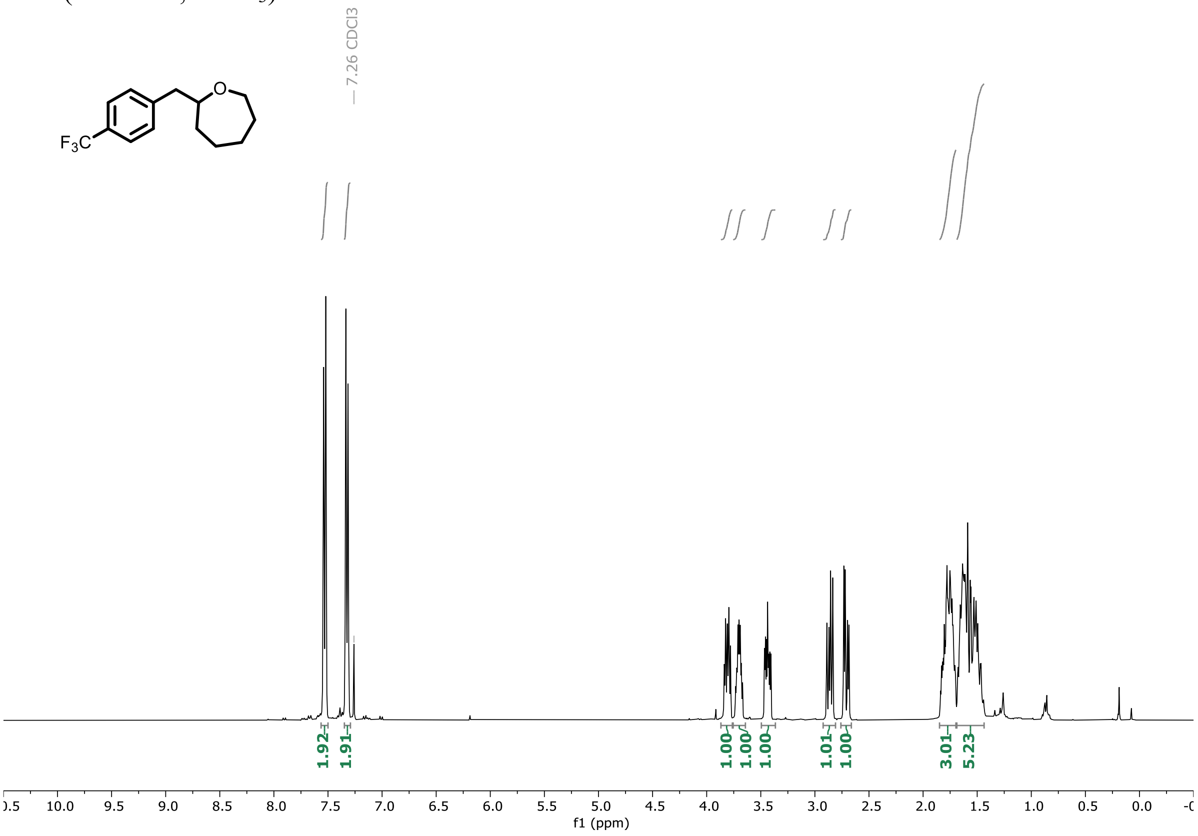

^{13}C NMR (101 MHz, $CDCl_3$) of **37**

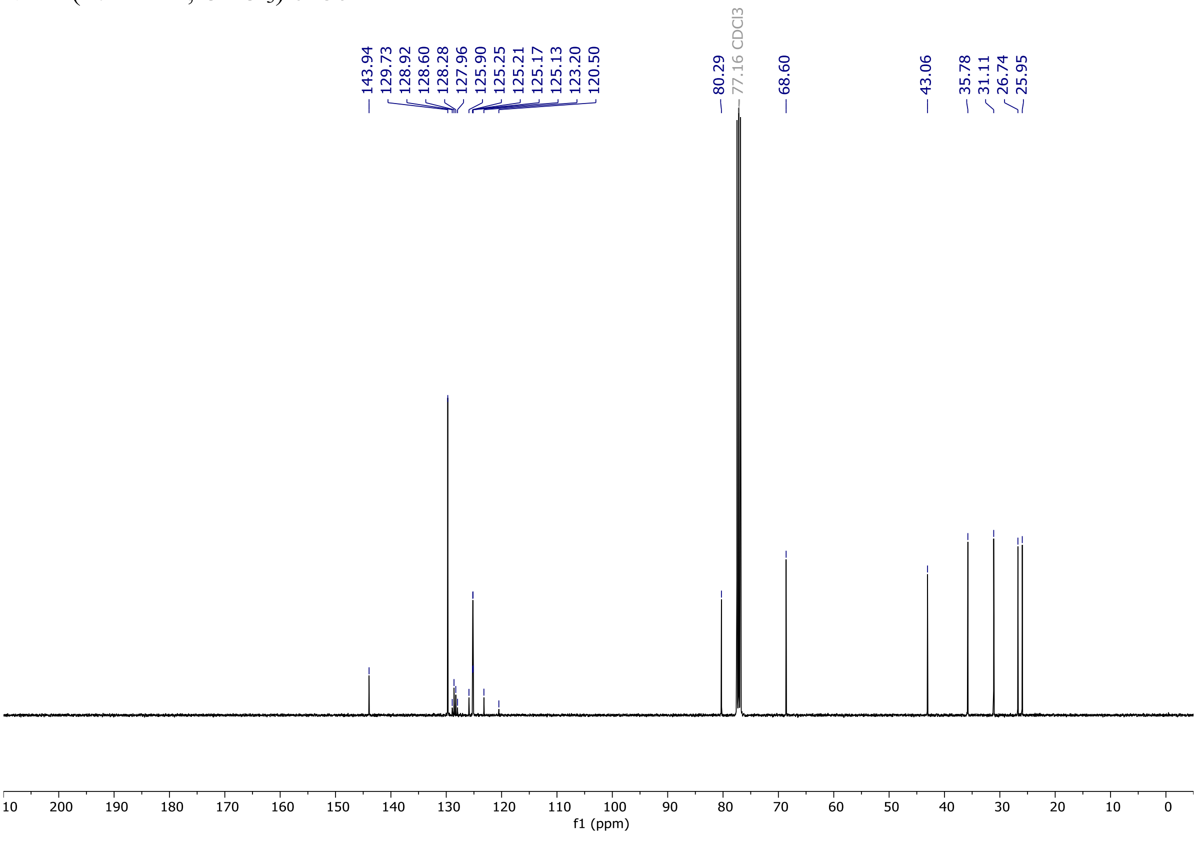

^{19}F NMR (282 MHz, $CDCl_3$) of **37**

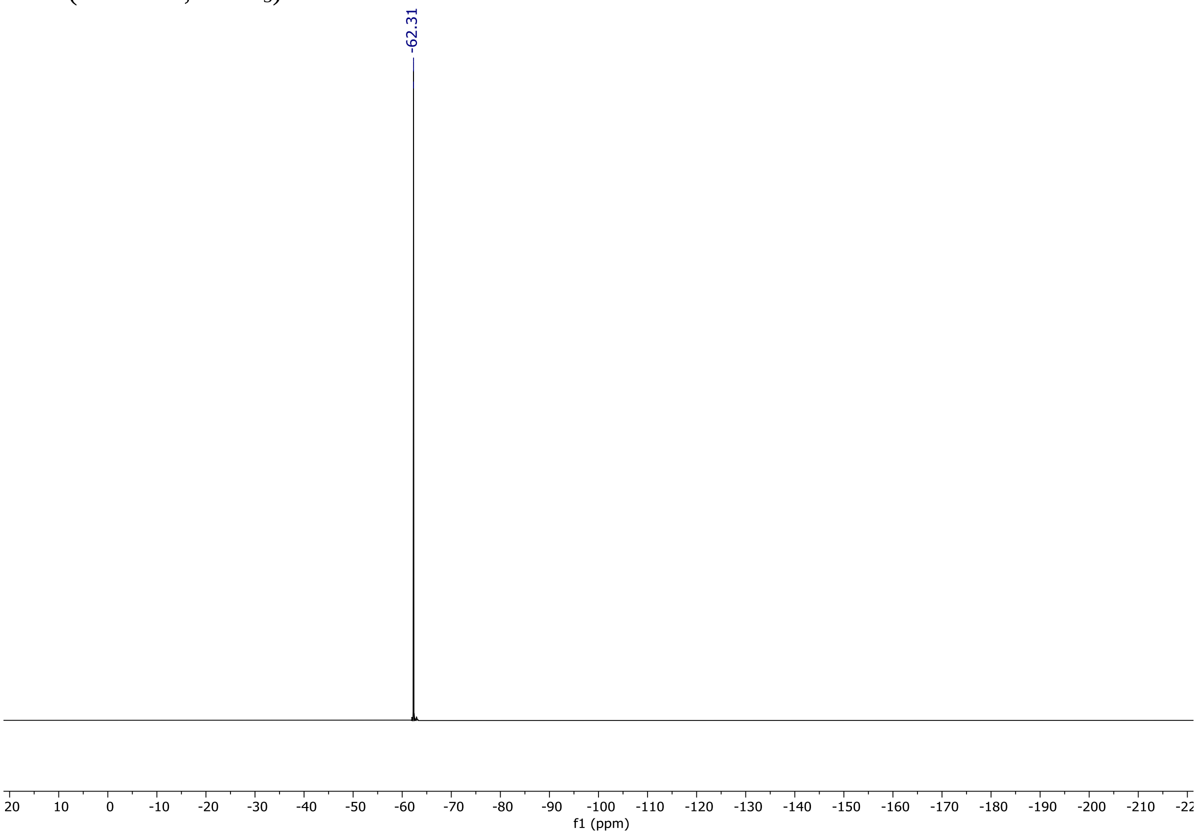

^{1}H NMR (400 MHz,$CDCl_3$) of **38**

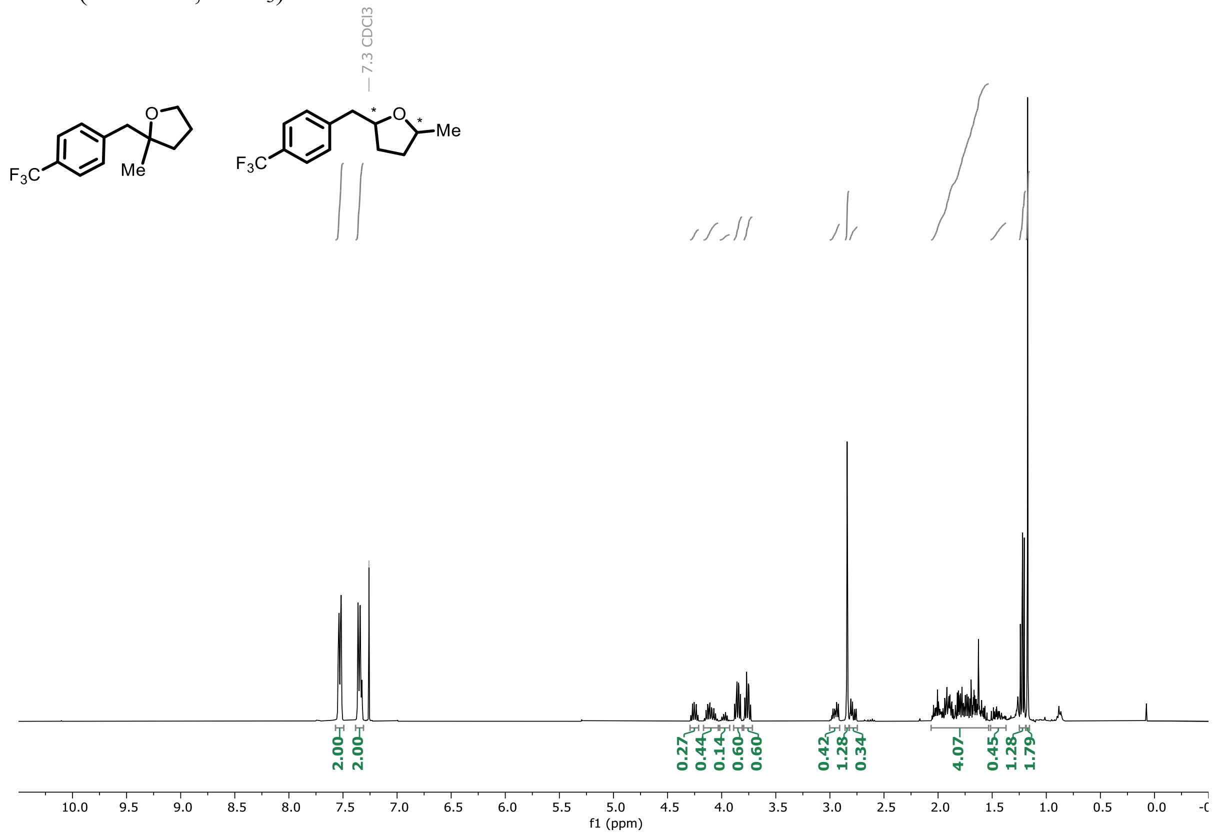

^{13}C NMR (101 MHz, $CDCl_3$) of **38**

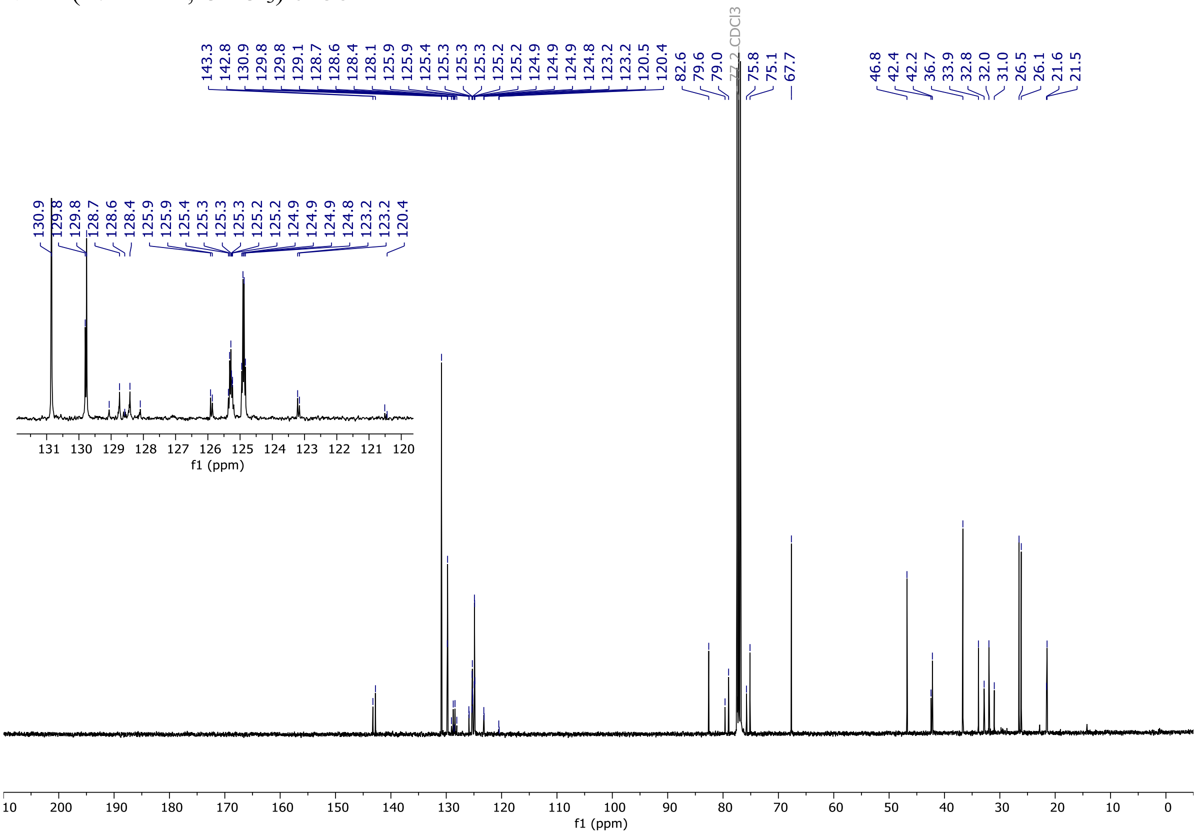

^{19}F NMR (282 MHz, $CDCl_3$) of **38**

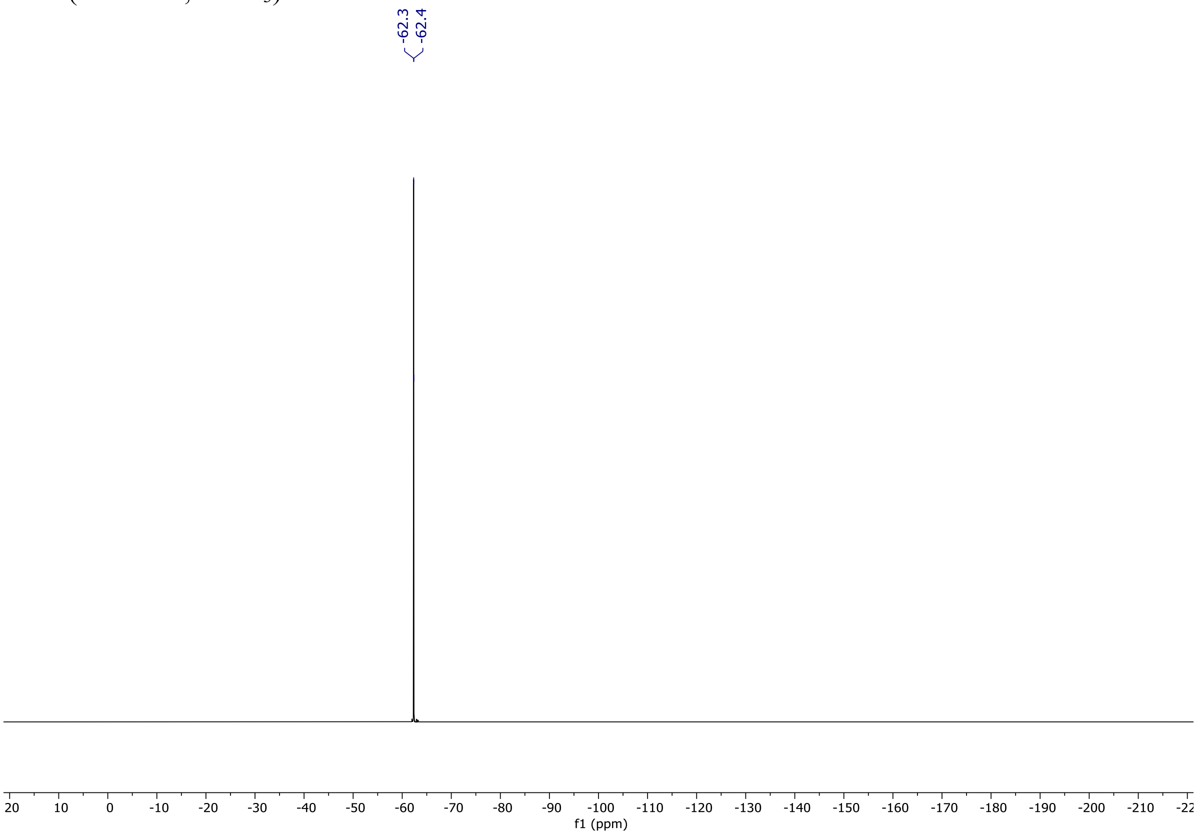

^{1}H NMR (400 MHz, $CDCl_3$) of **39**

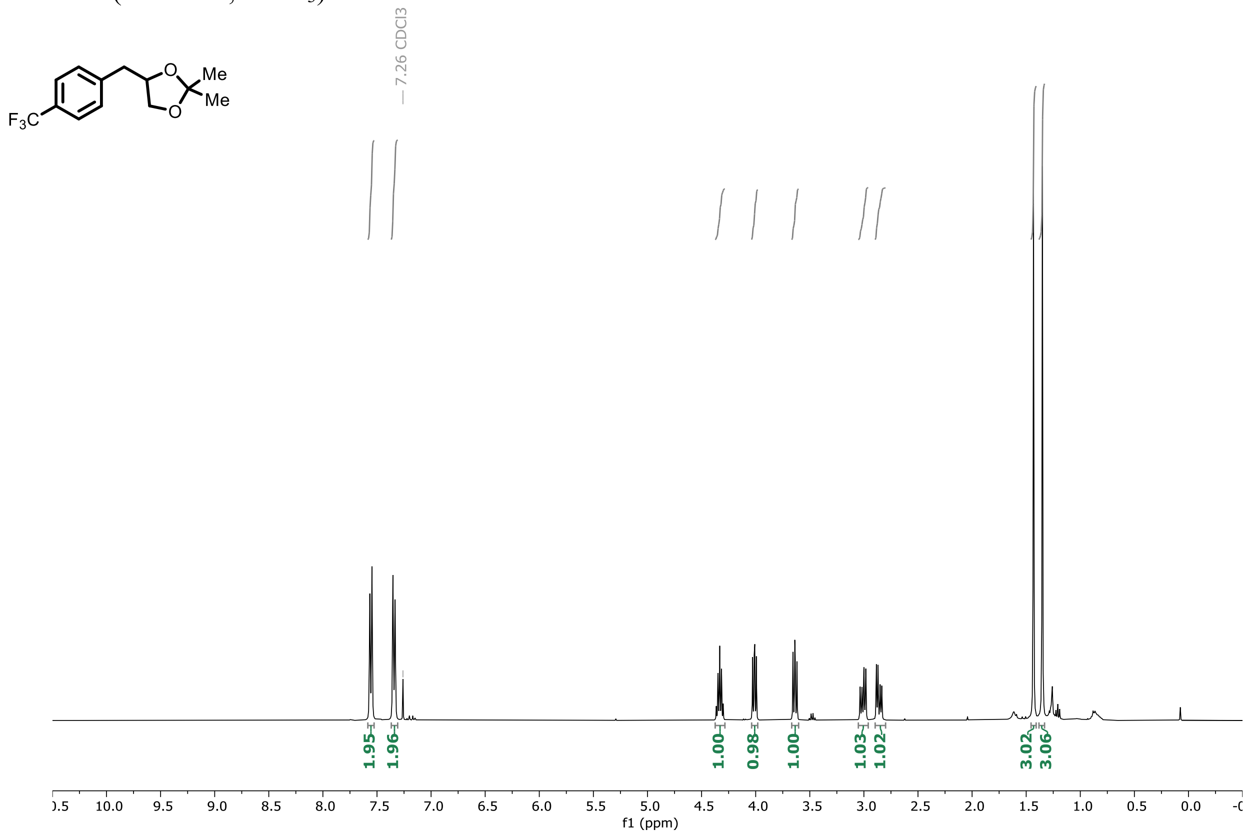

^{13}C NMR (101 MHz, $CDCl_3$) of **39**

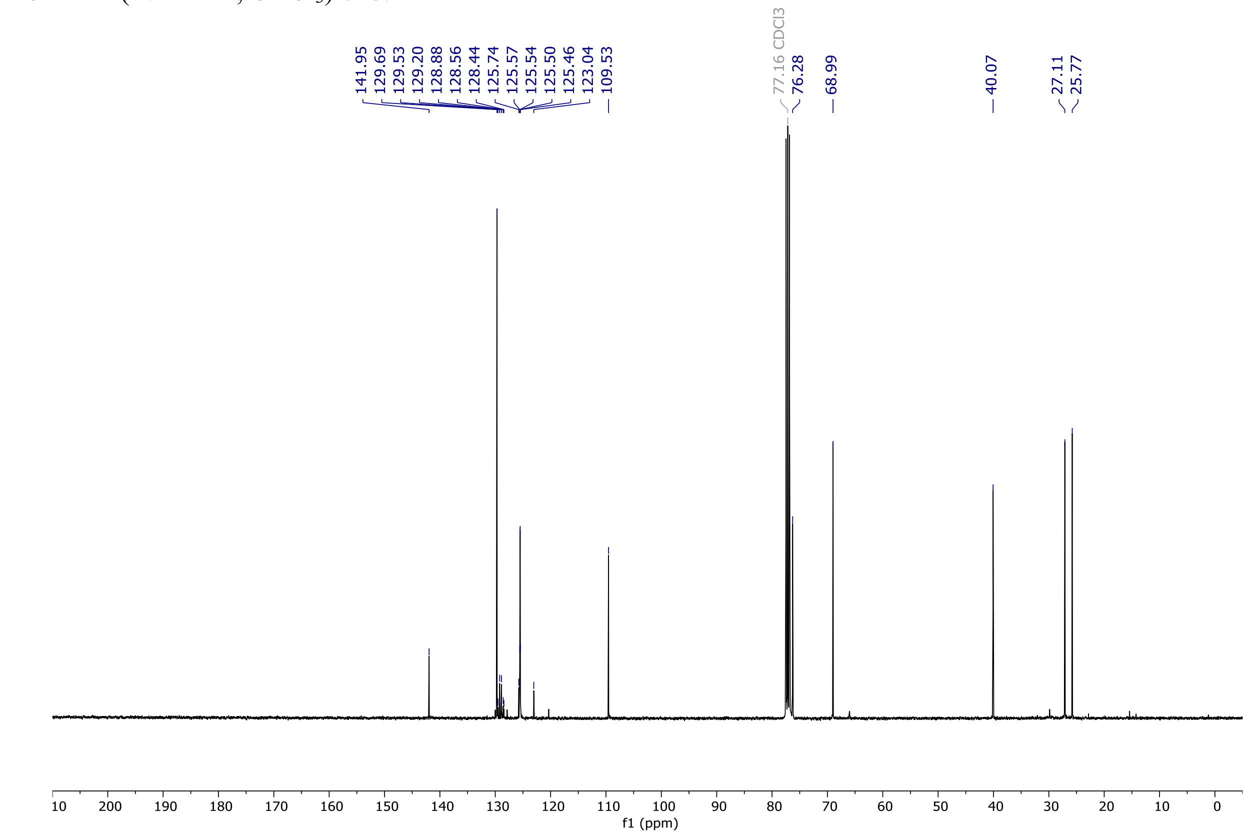

^{19}F NMR (282 MHz, $CDCl_3$) of **39**

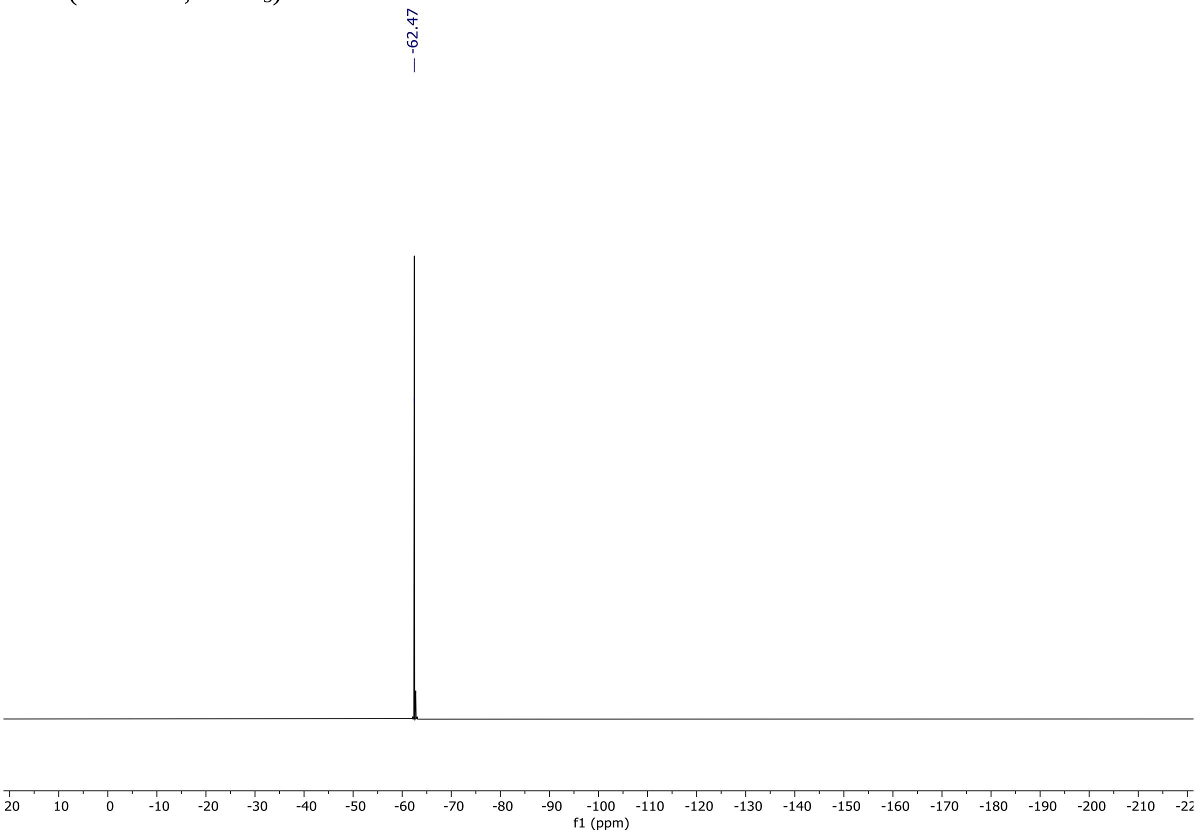

^{1}H NMR (400 MHz, $CDCl_3$) of **40**

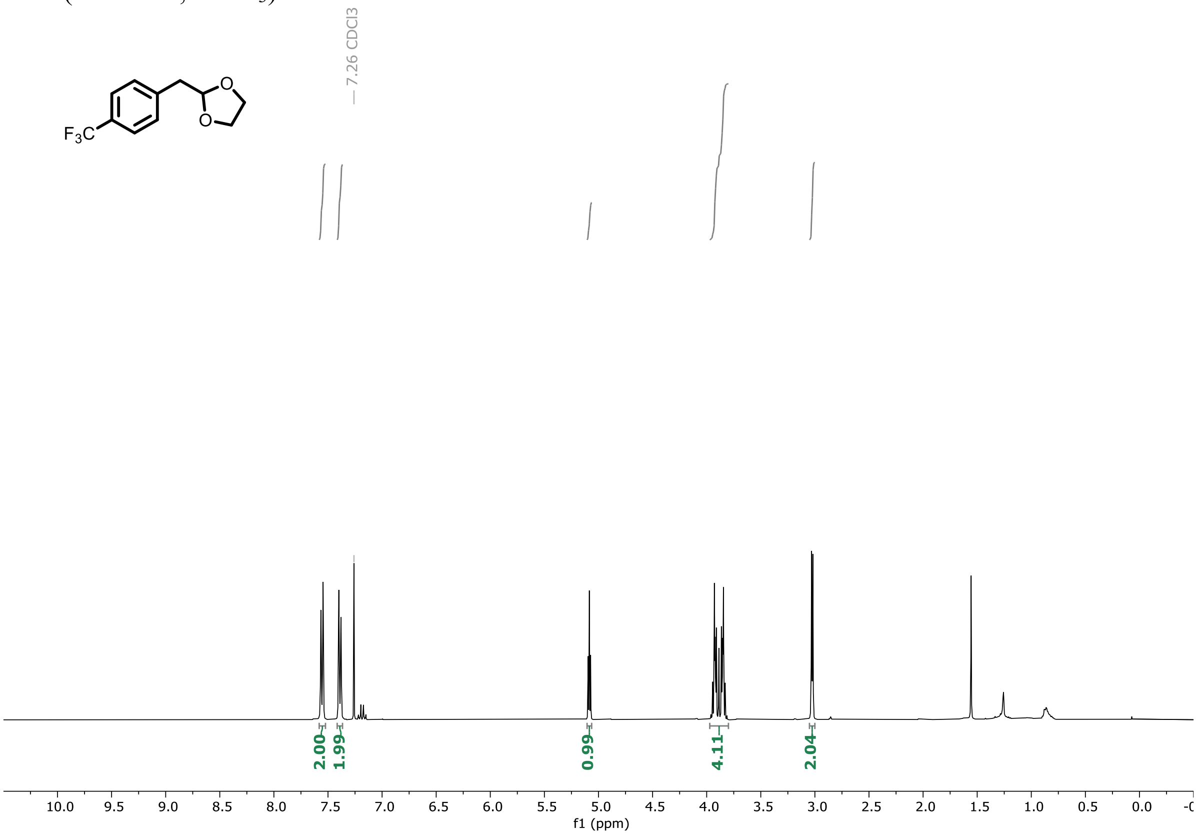

^{13}C NMR (101 MHz, $CDCl_3$) of **40**

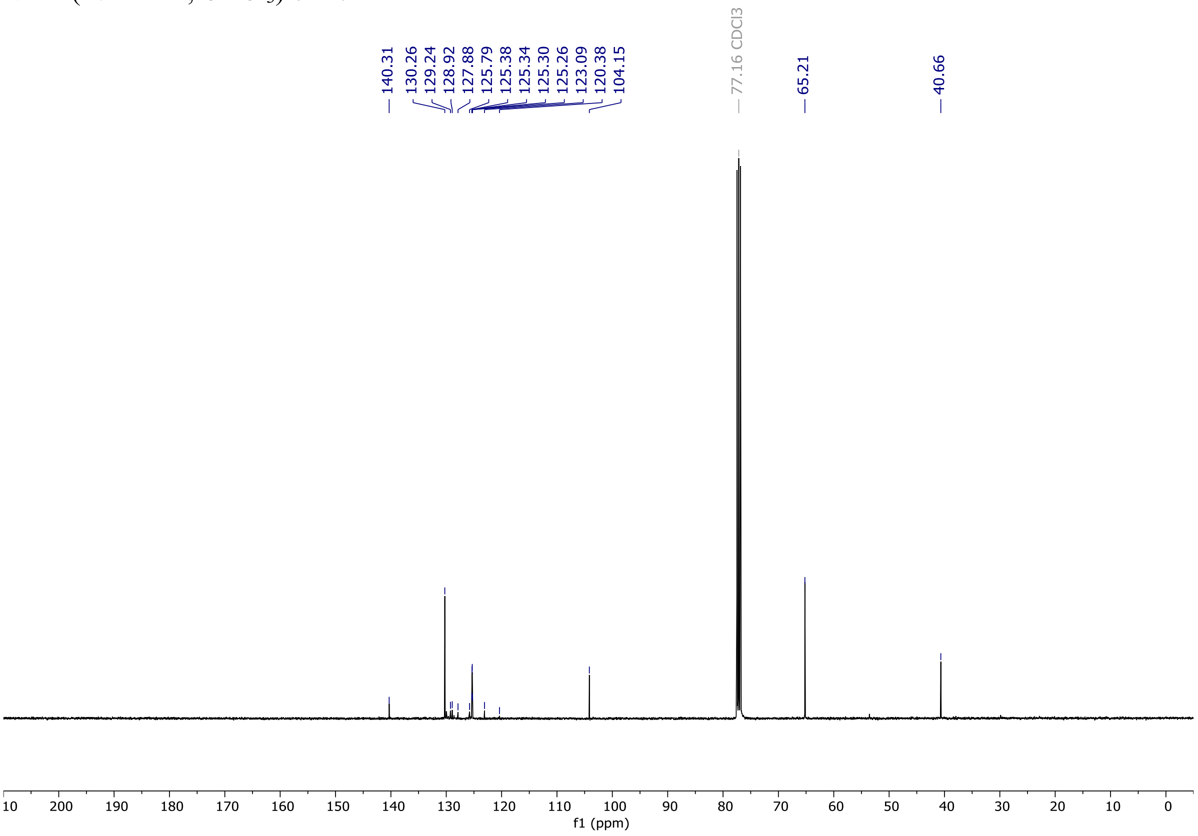

^{19}F NMR (282 MHz, $CDCl_3$) of **40**

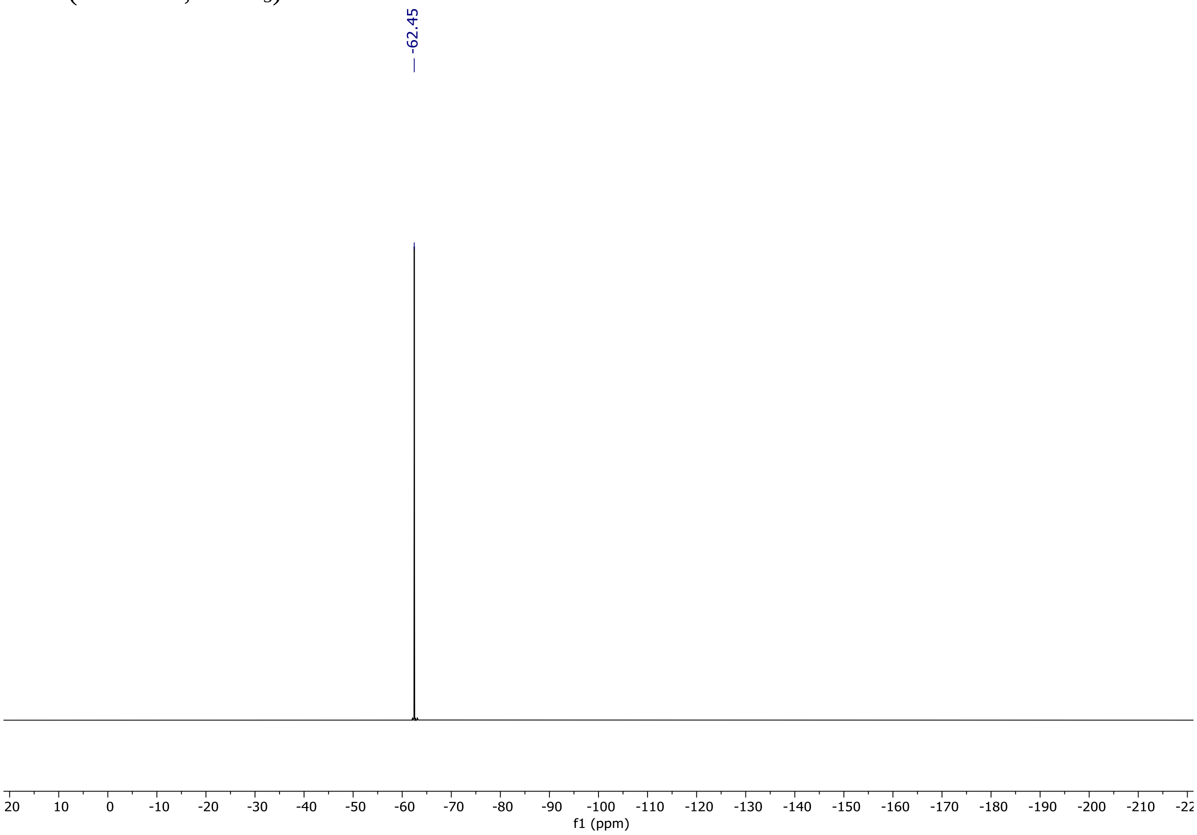

^{1}H NMR (400 MHz,CDCl$_3$) of **41**

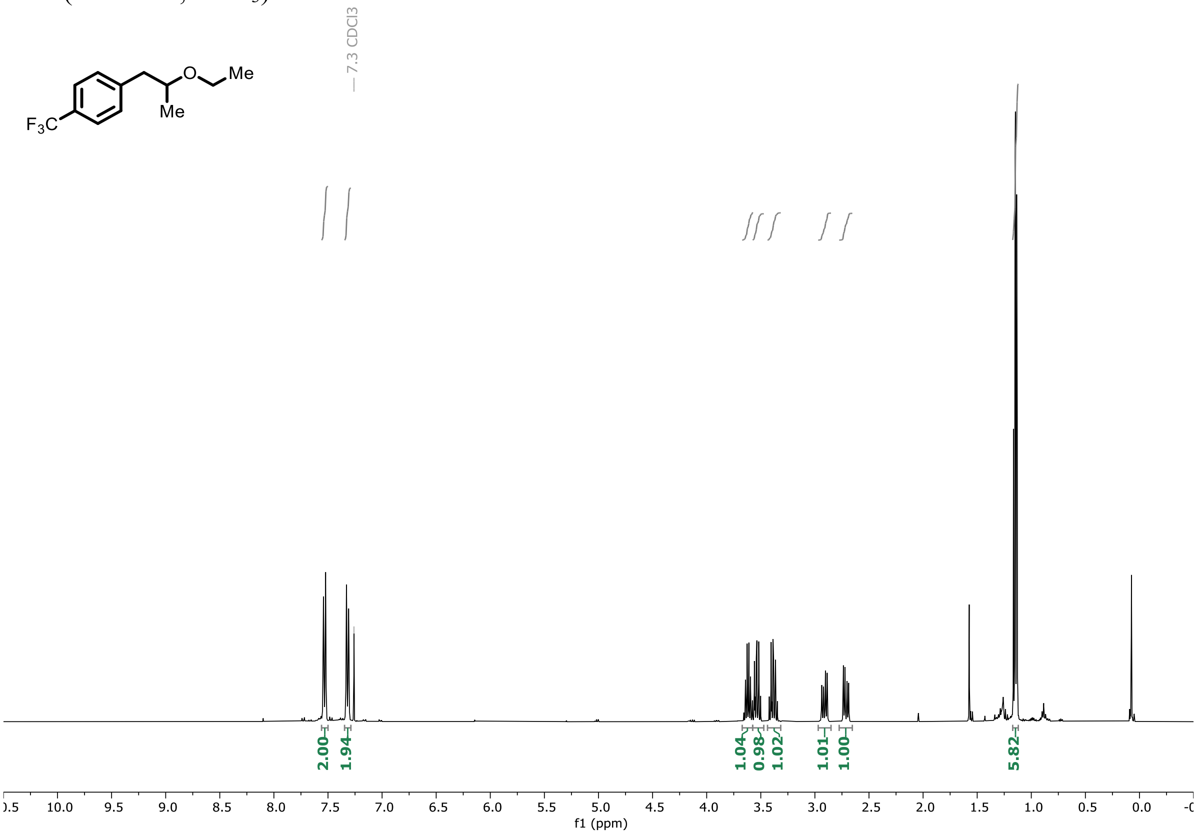

^{13}C NMR (101 MHz, CDCl$_3$) of **41**

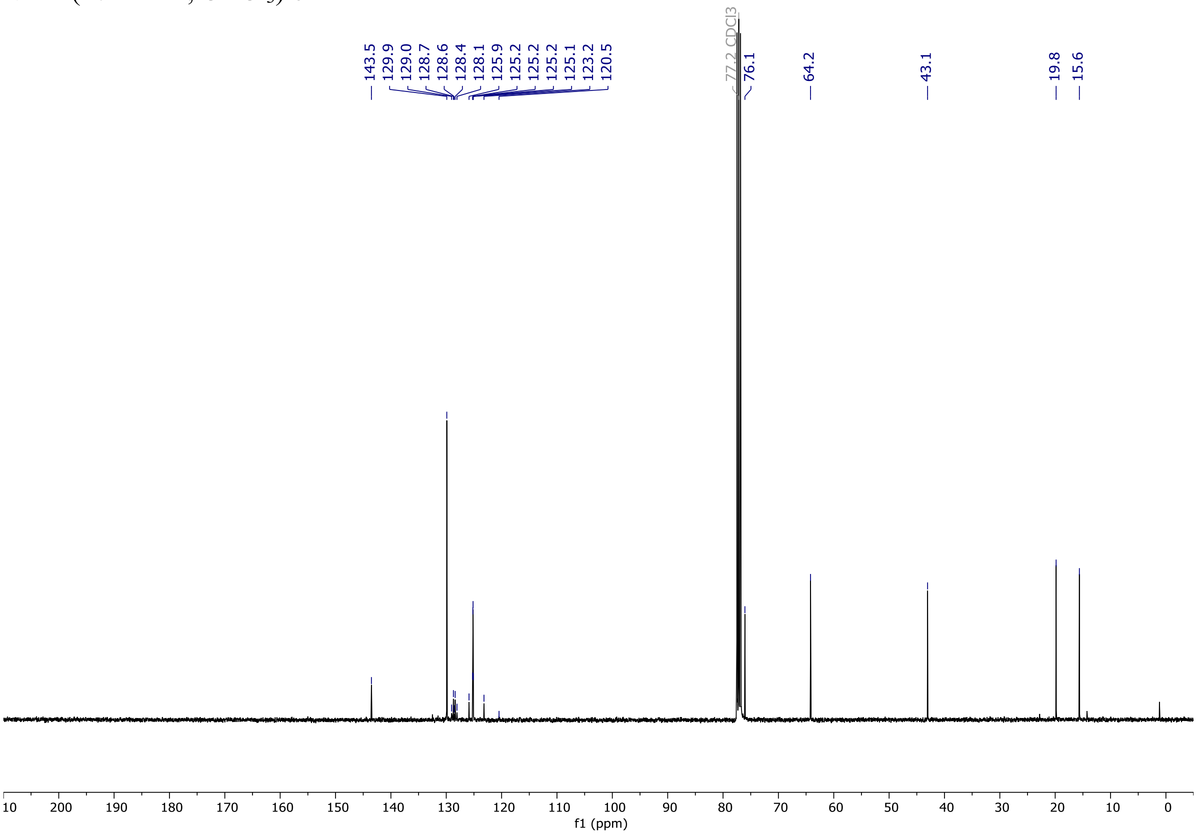

^{19}F NMR (282 MHz, $CDCl_3$) of **41**

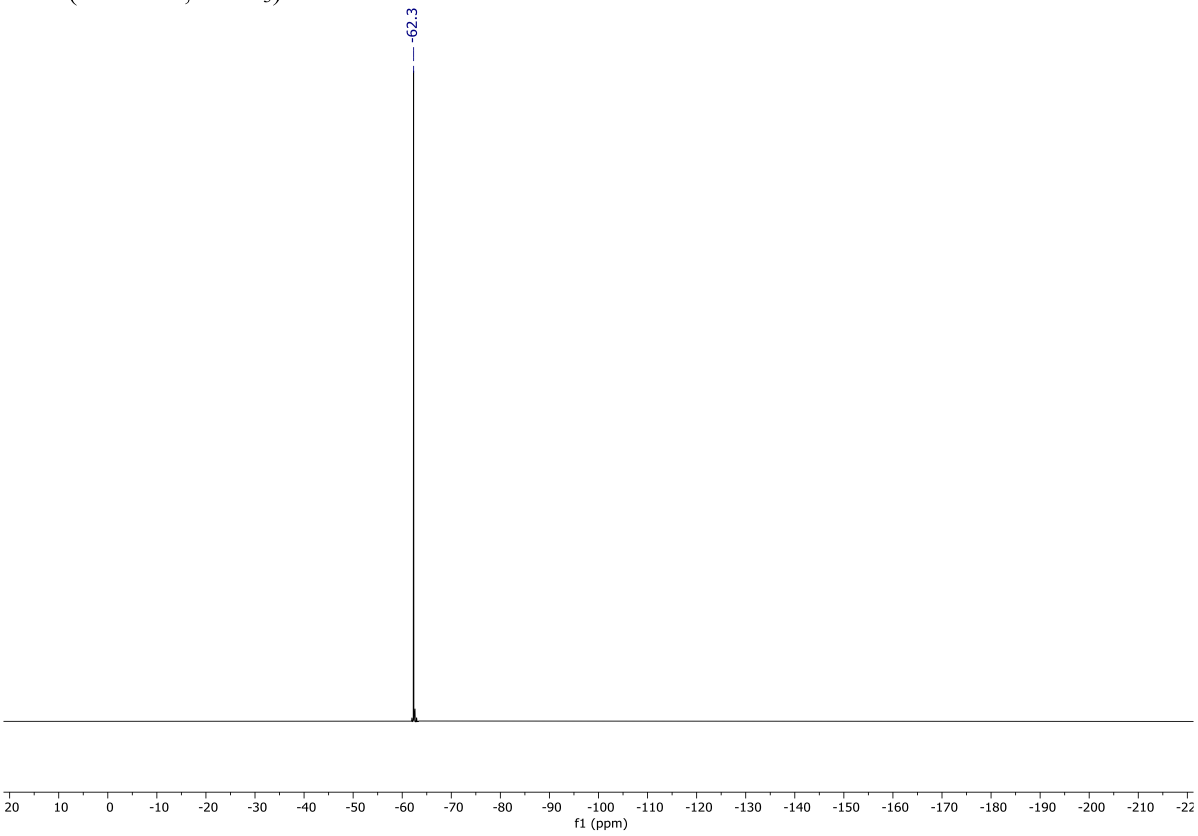

^{1}H NMR (400 MHz, $CDCl_3$) of **42**

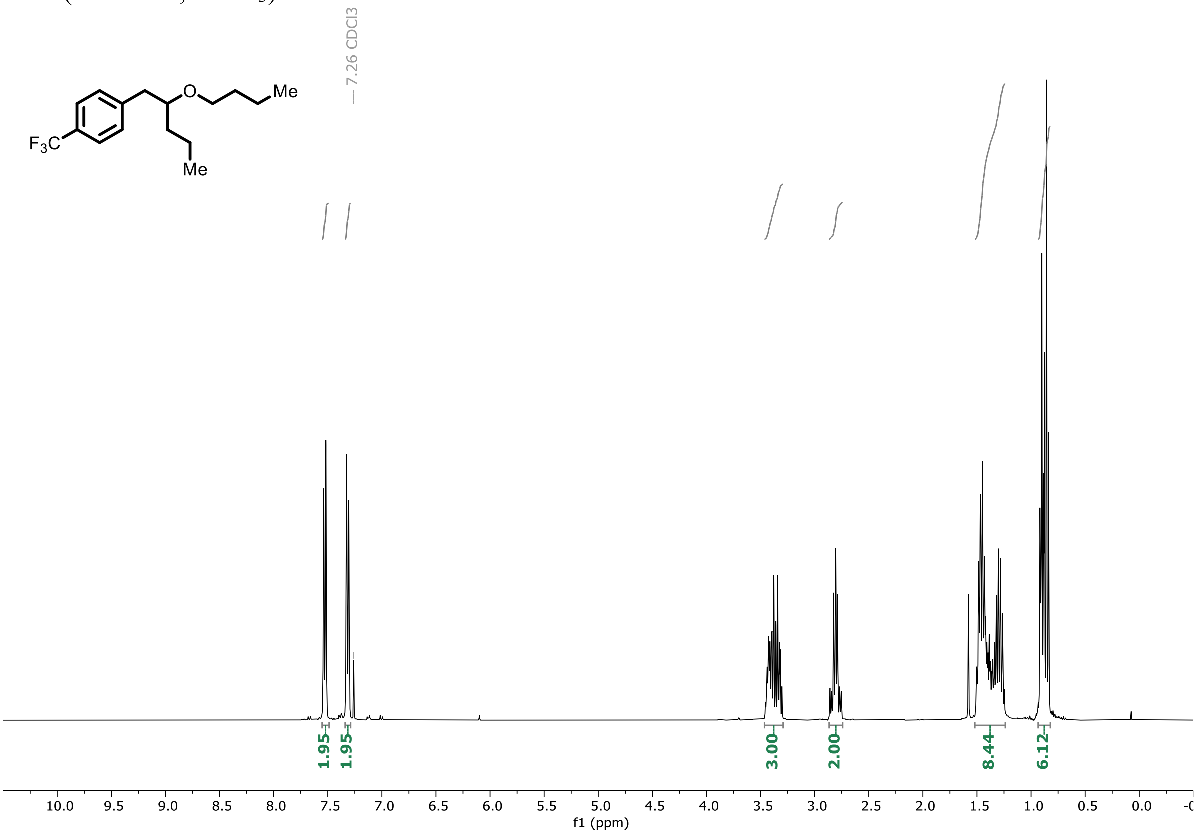

^{13}C NMR (101 MHz, $CDCl_3$) of **42**

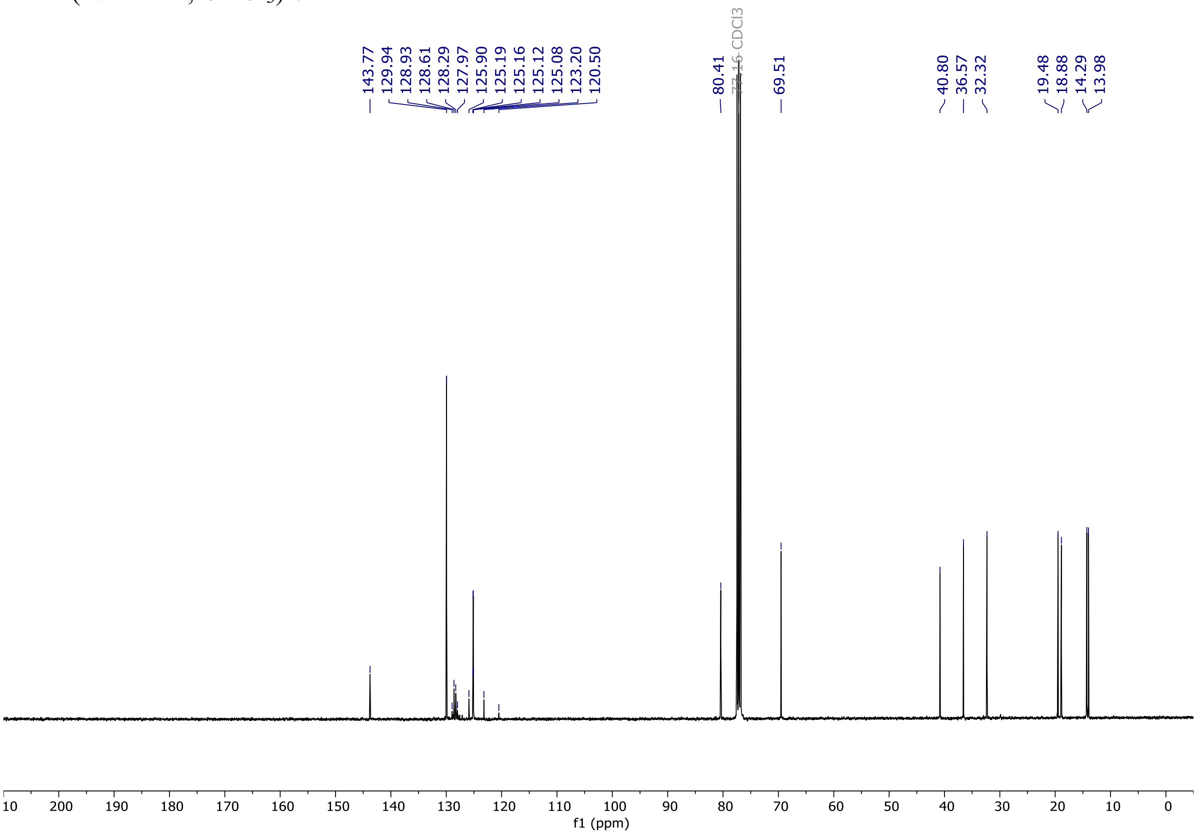

^{19}F NMR (282 MHz, $CDCl_3$) of **42**

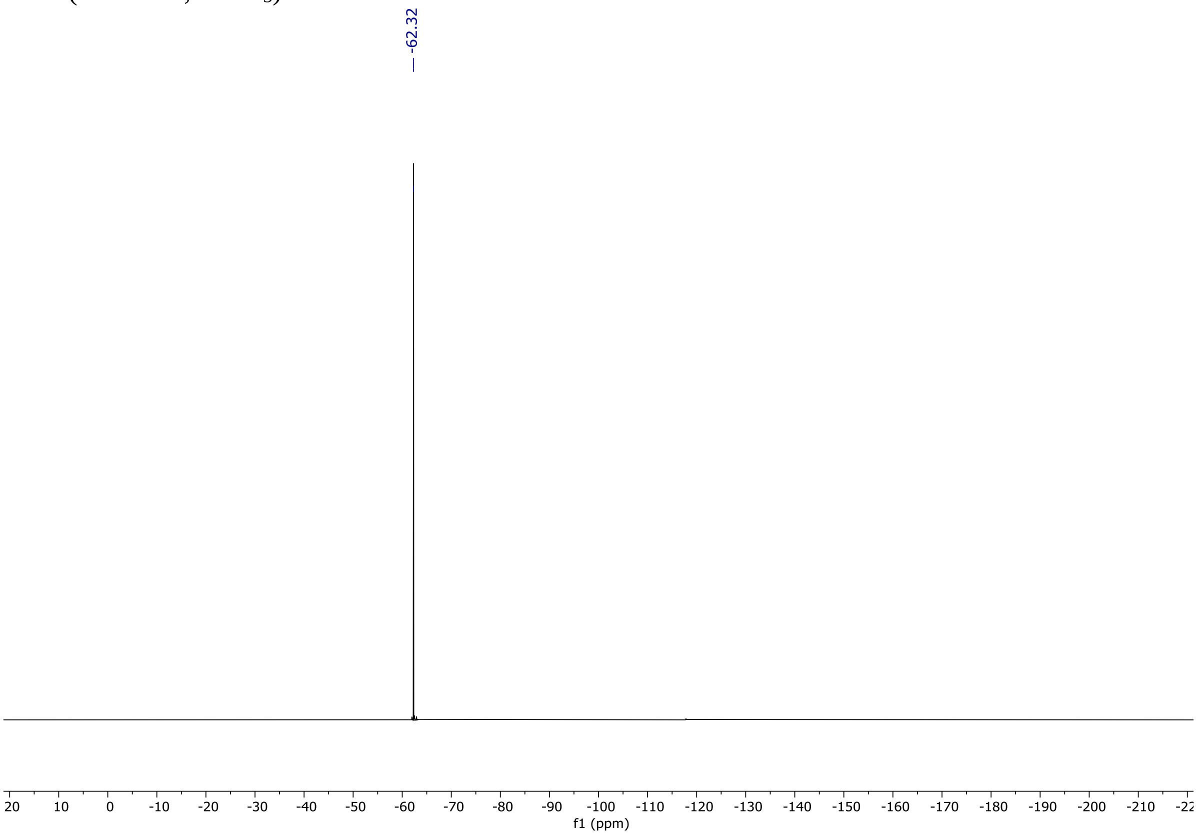

^{1}H NMR (400 MHz, $CDCl_3$) of **43**

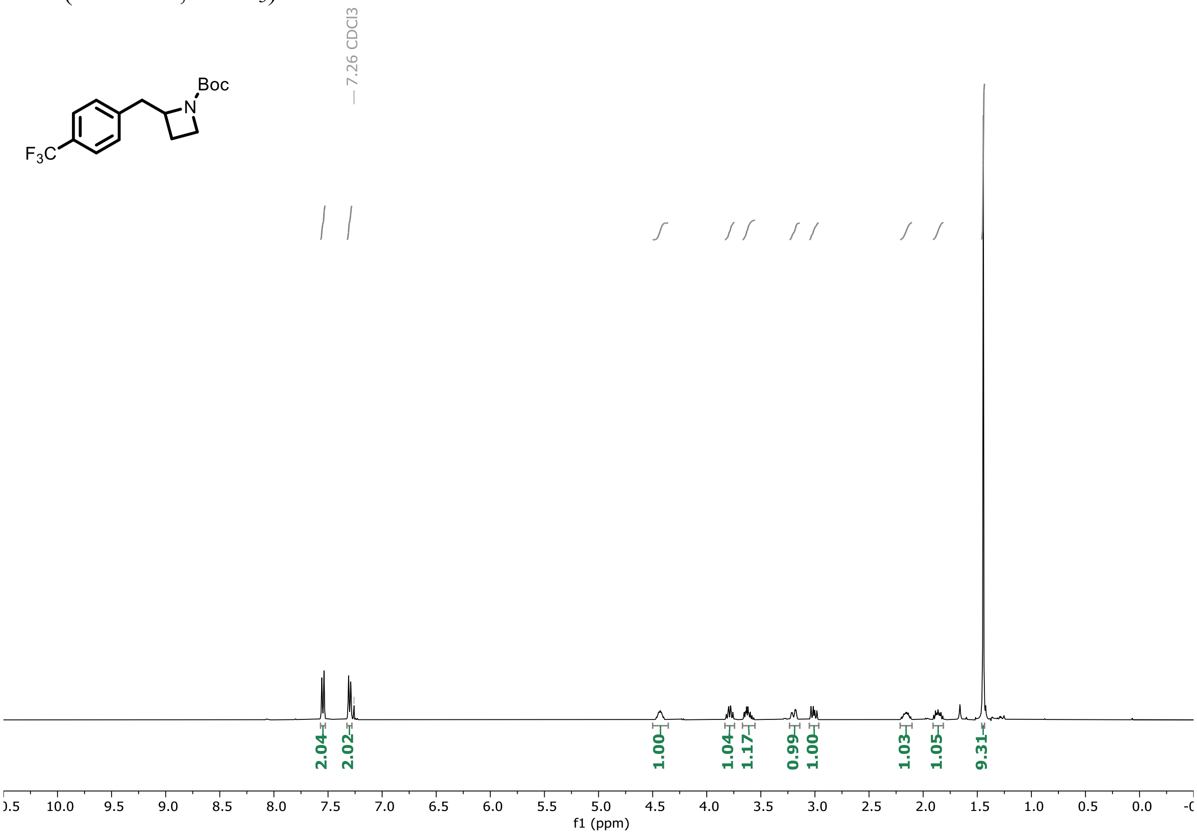

^{13}C NMR (101 MHz, $CDCl_3$) of **43**

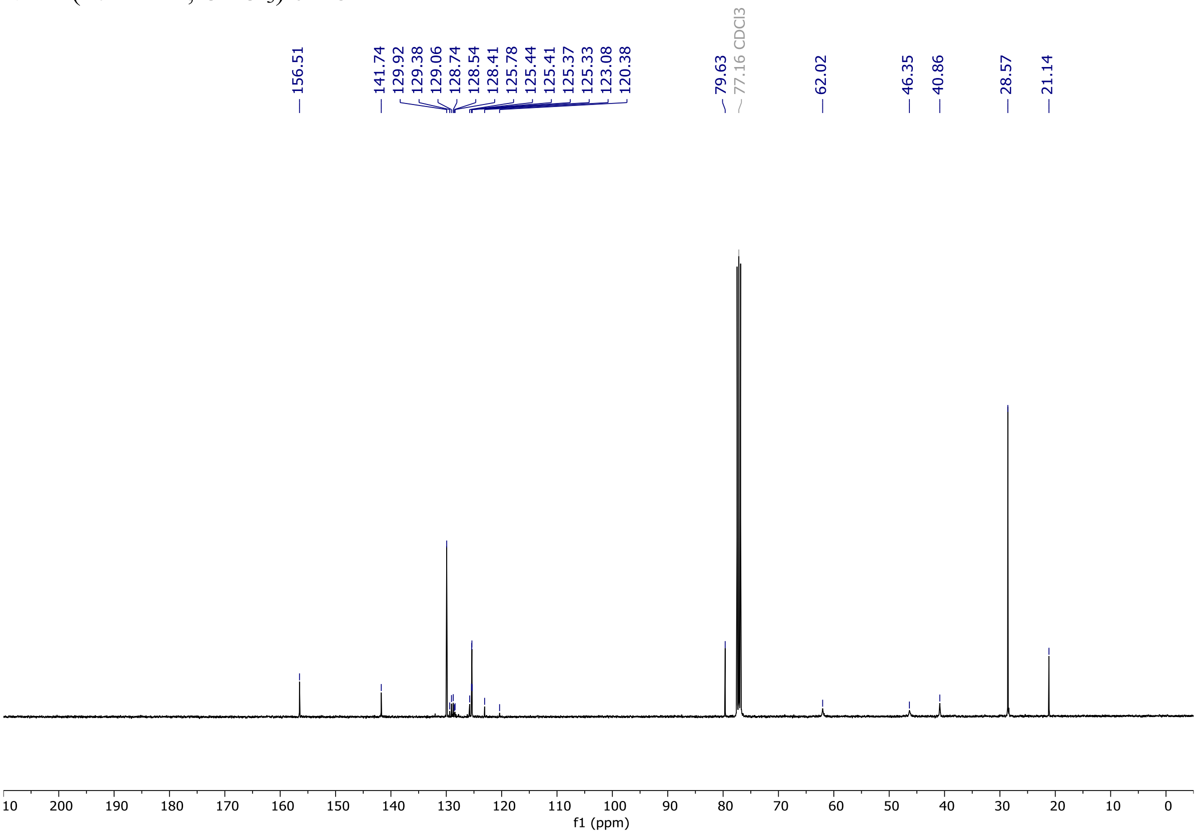

^{19}F NMR (282 MHz, $CDCl_3$) of **43**

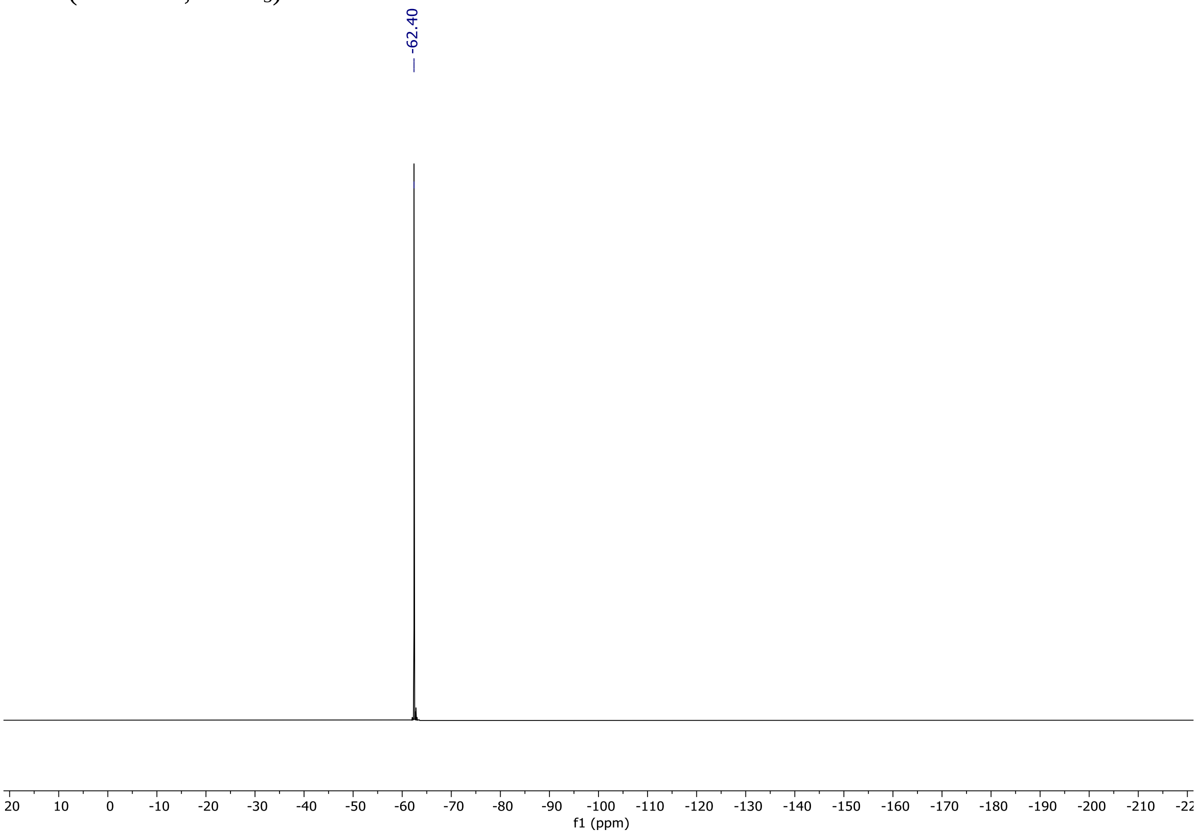

^{1}H NMR (400 MHz,CDCl$_3$) of **44**

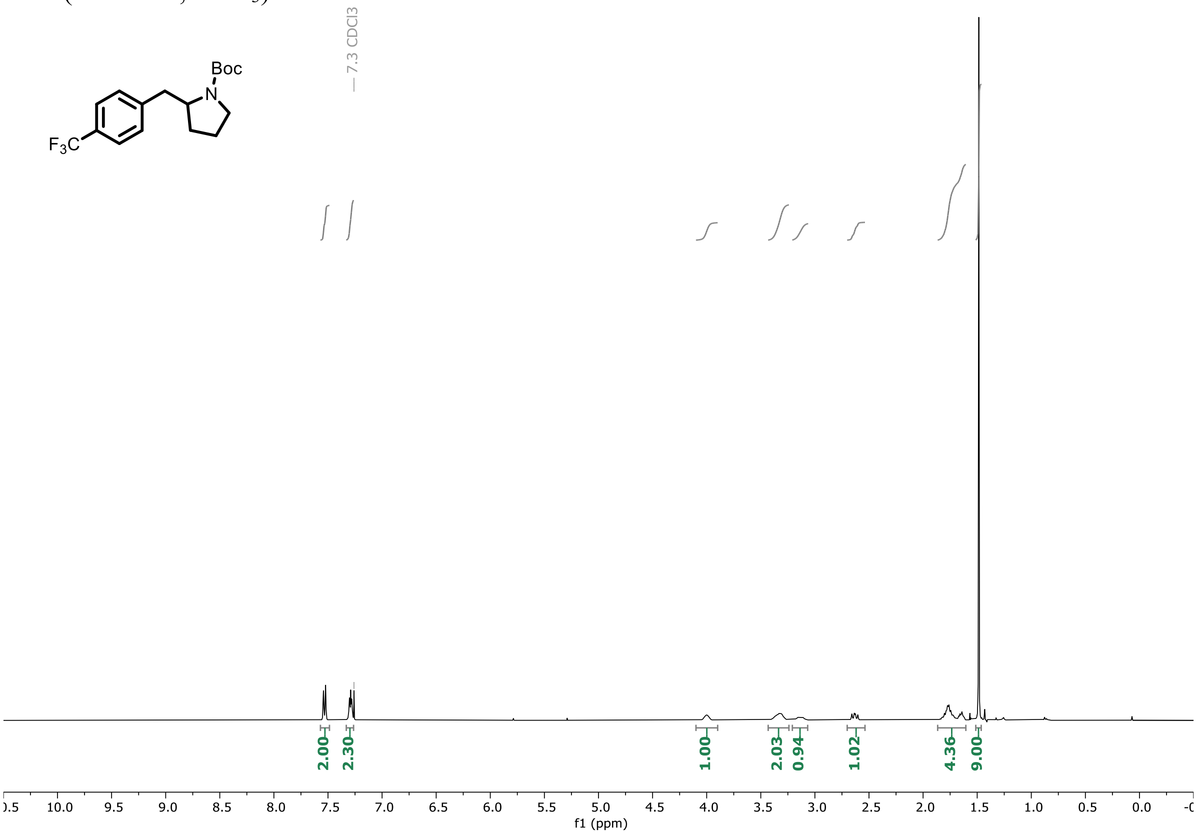

^{13}C NMR (101 MHz, CDCl$_3$) of **44**

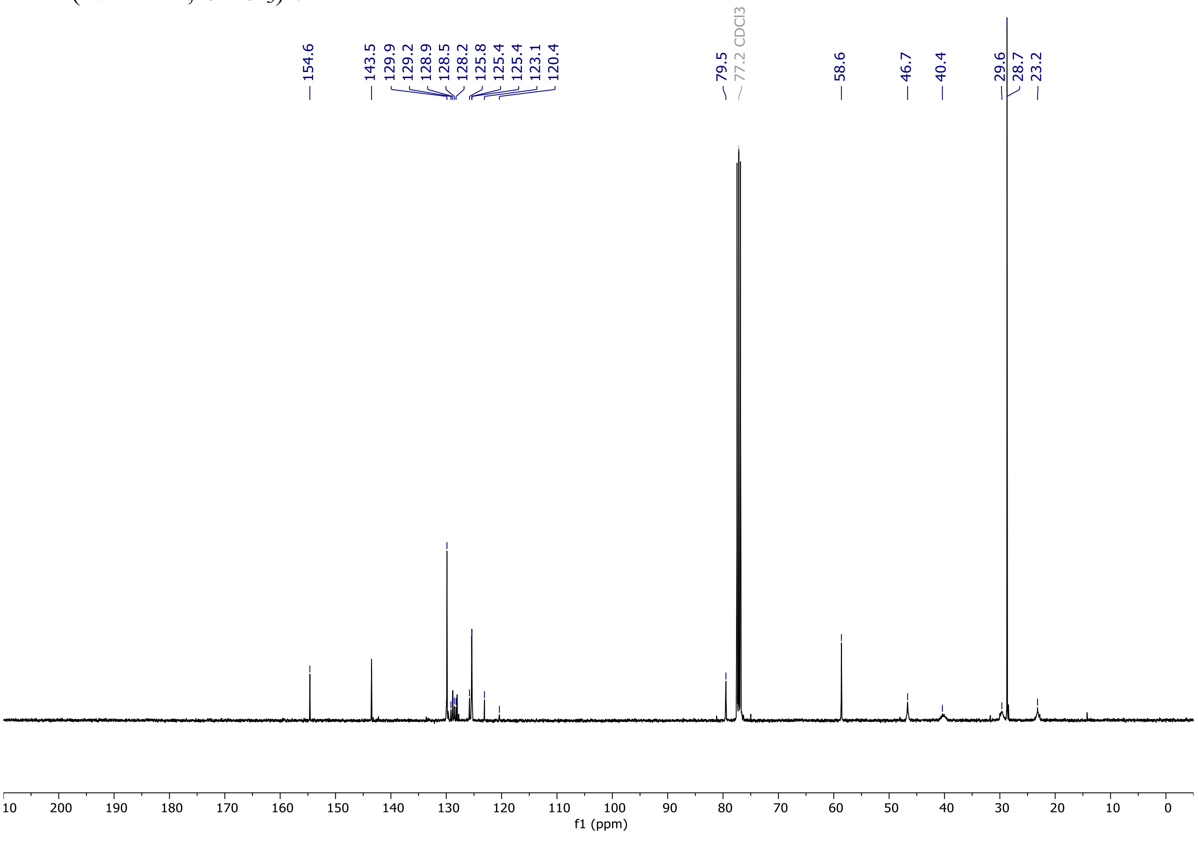

^{19}F NMR (282 MHz, $CDCl_3$) of **44**

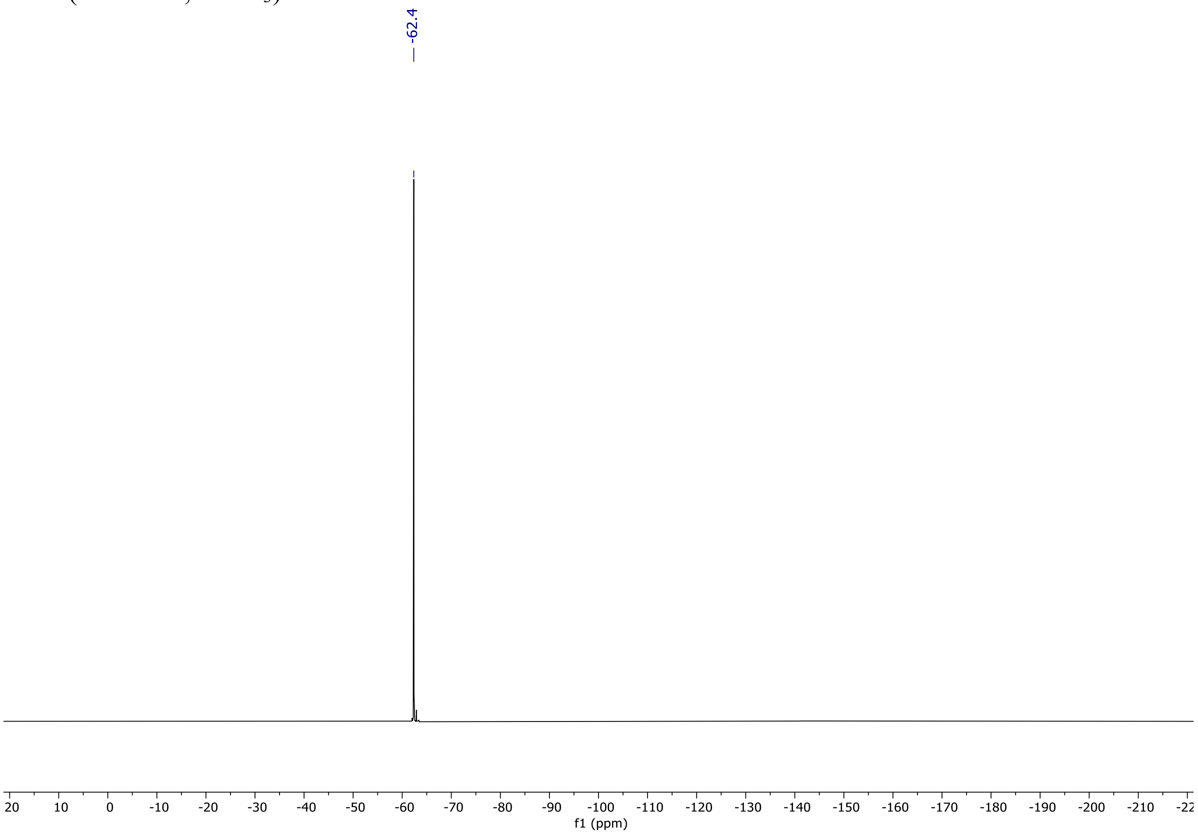

^{1}H NMR (400 MHz,$CDCl_3$) of **45**

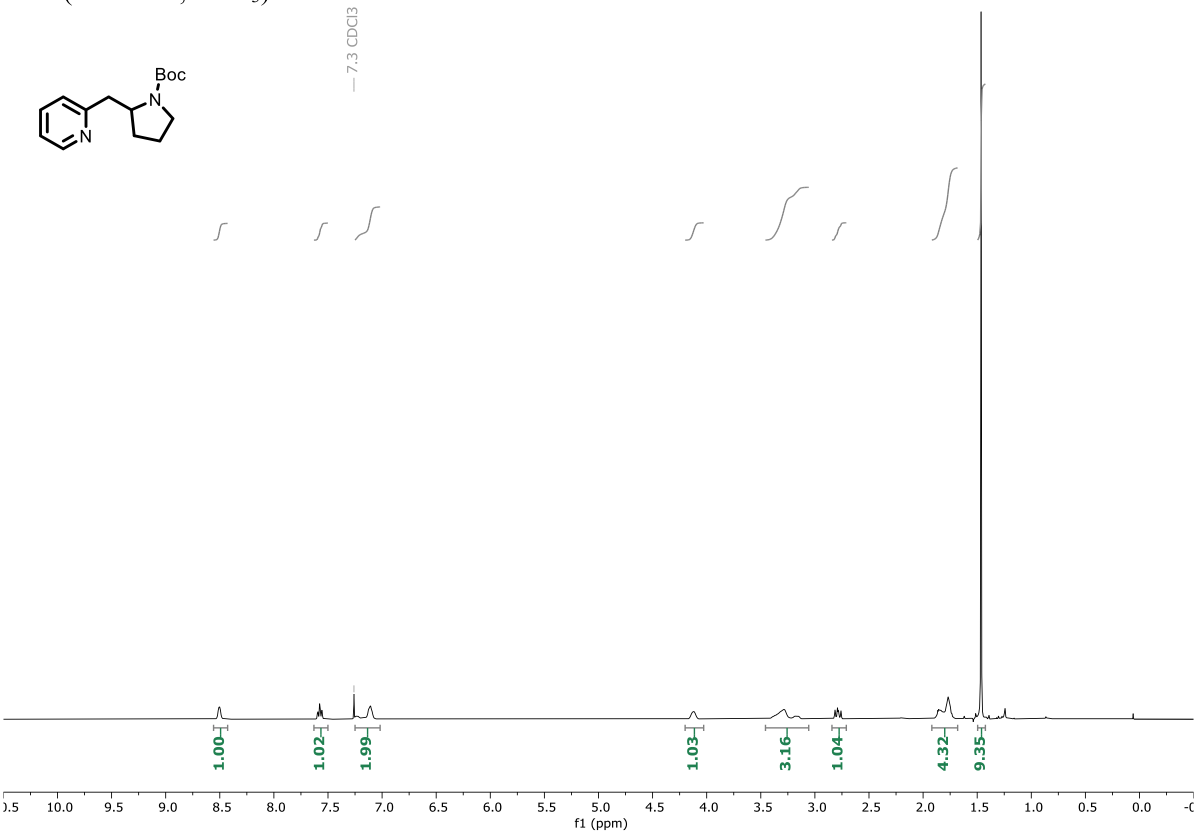

^{13}C NMR (101 MHz, $CDCl_3$) of **45**

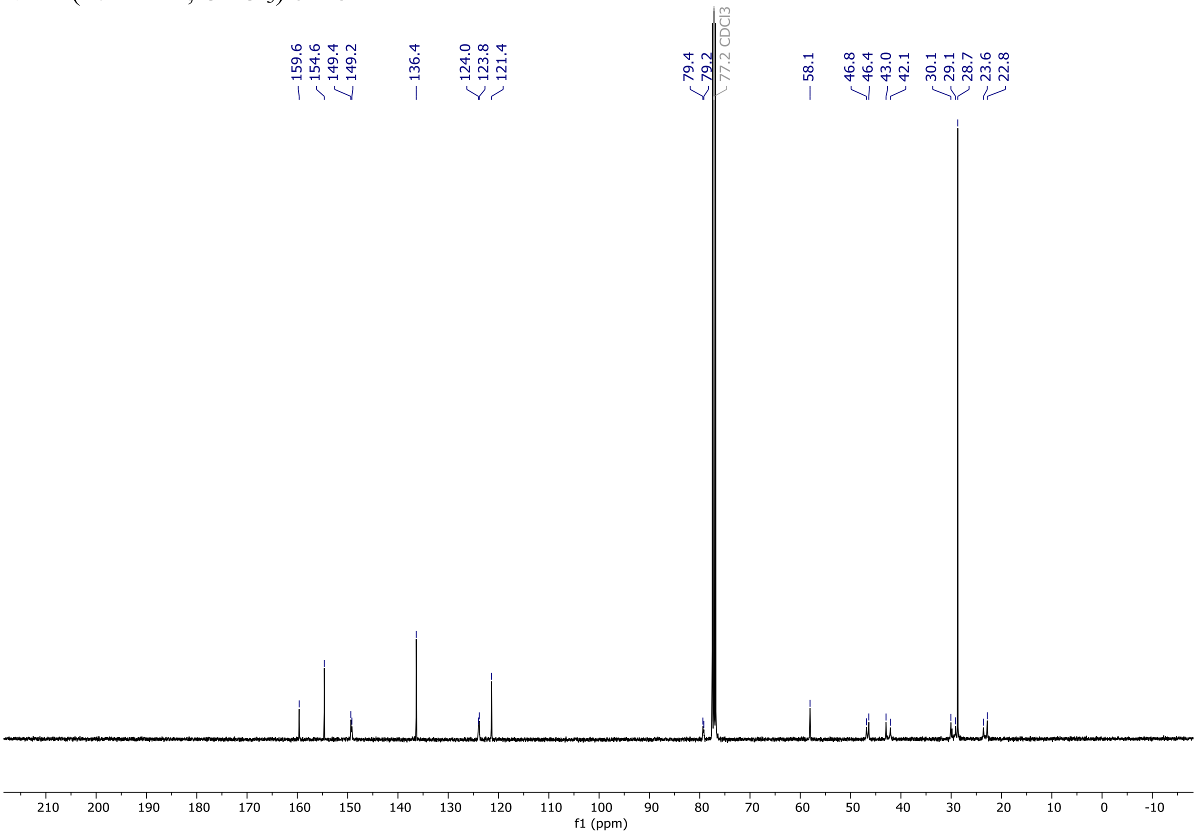

^{1}H NMR (400 MHz,CDCl$_3$) of **46**

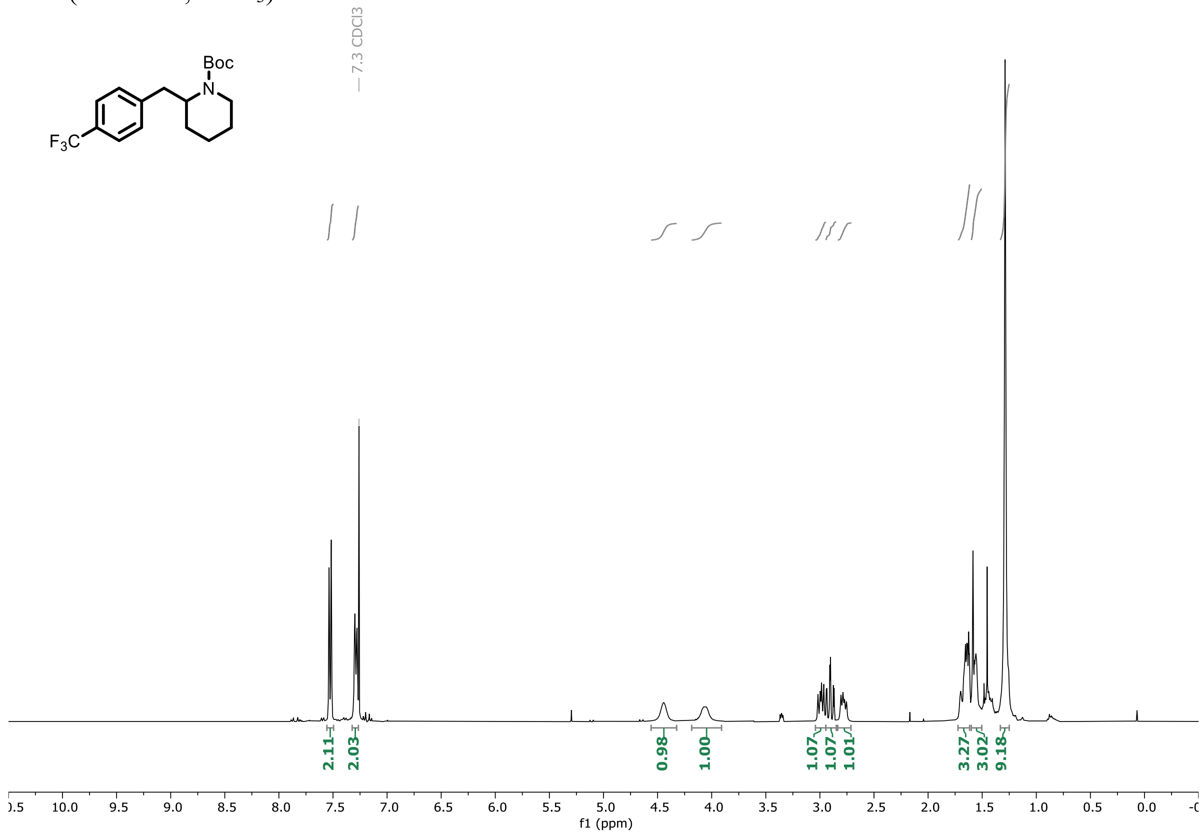

^{13}C NMR (101 MHz, CDCl$_3$) of **46**

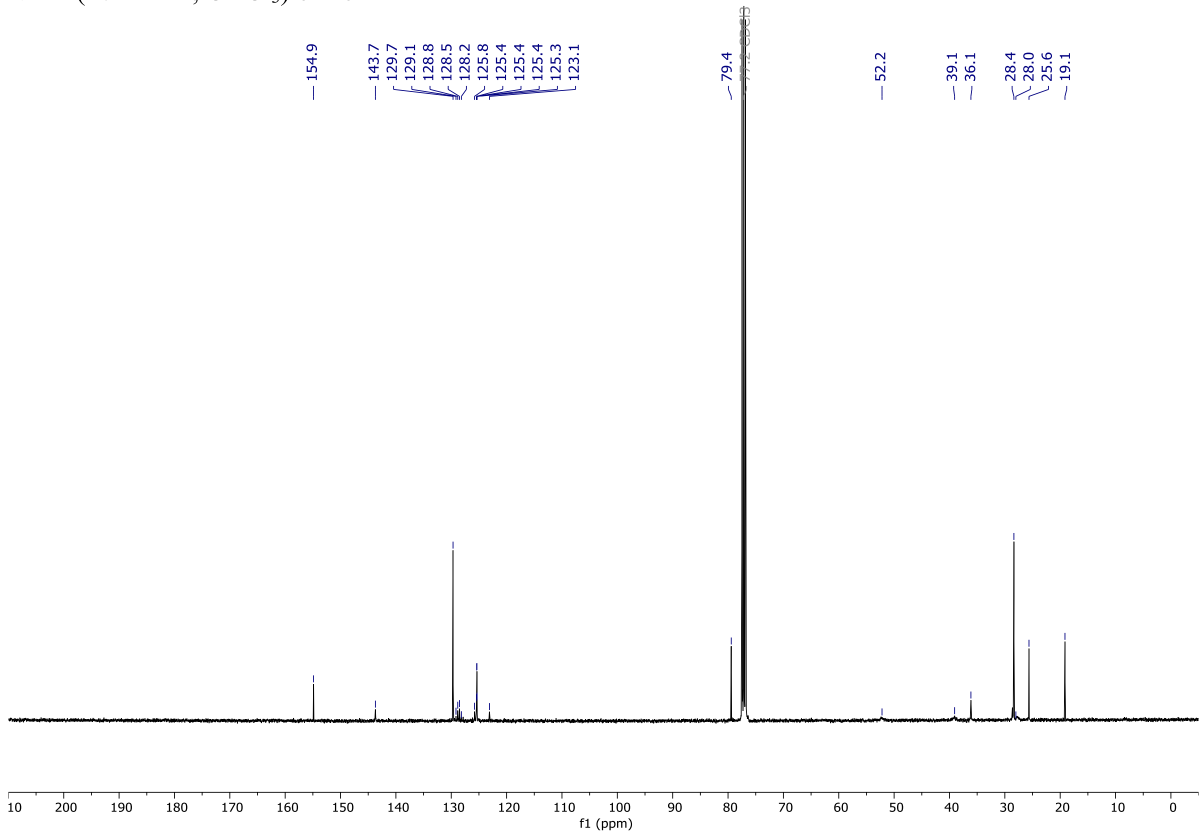

^{19}F NMR (282 MHz, $CDCl_3$) of **46**

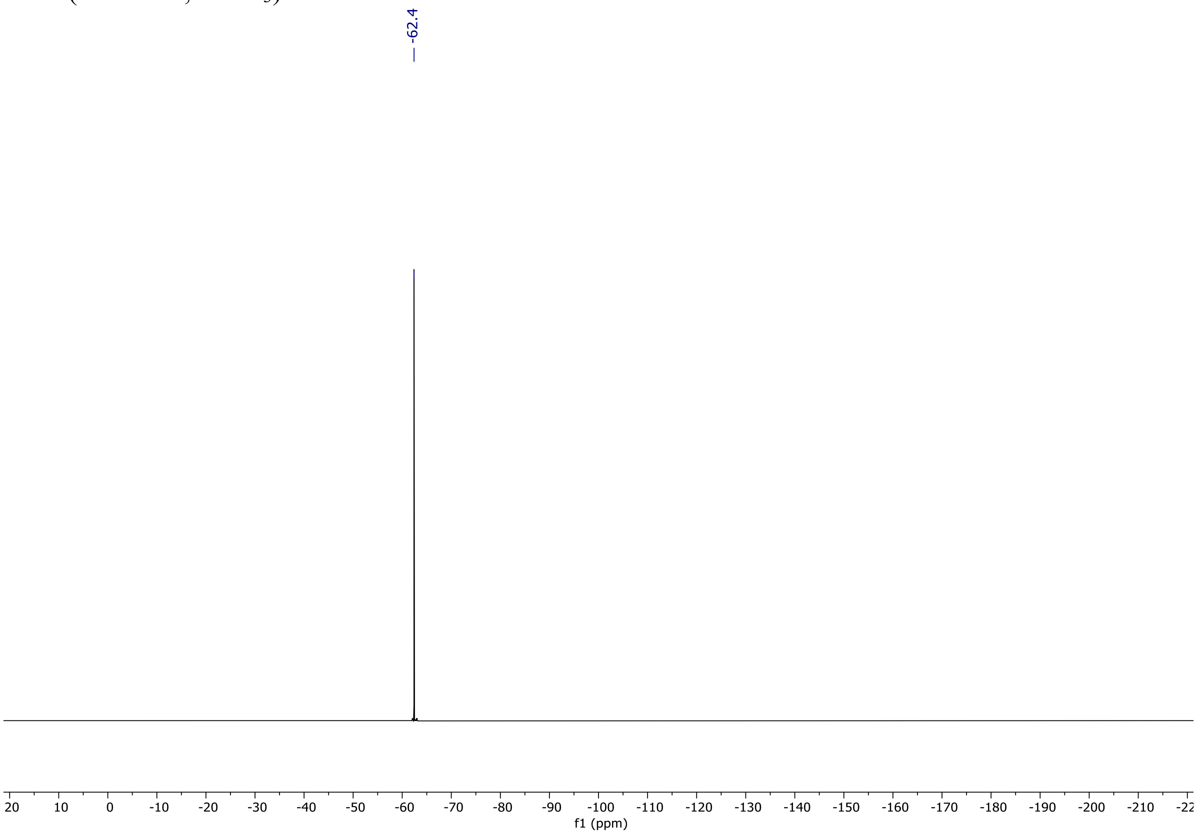

^{1}H NMR (400 MHz, $CDCl_3$) of **47**

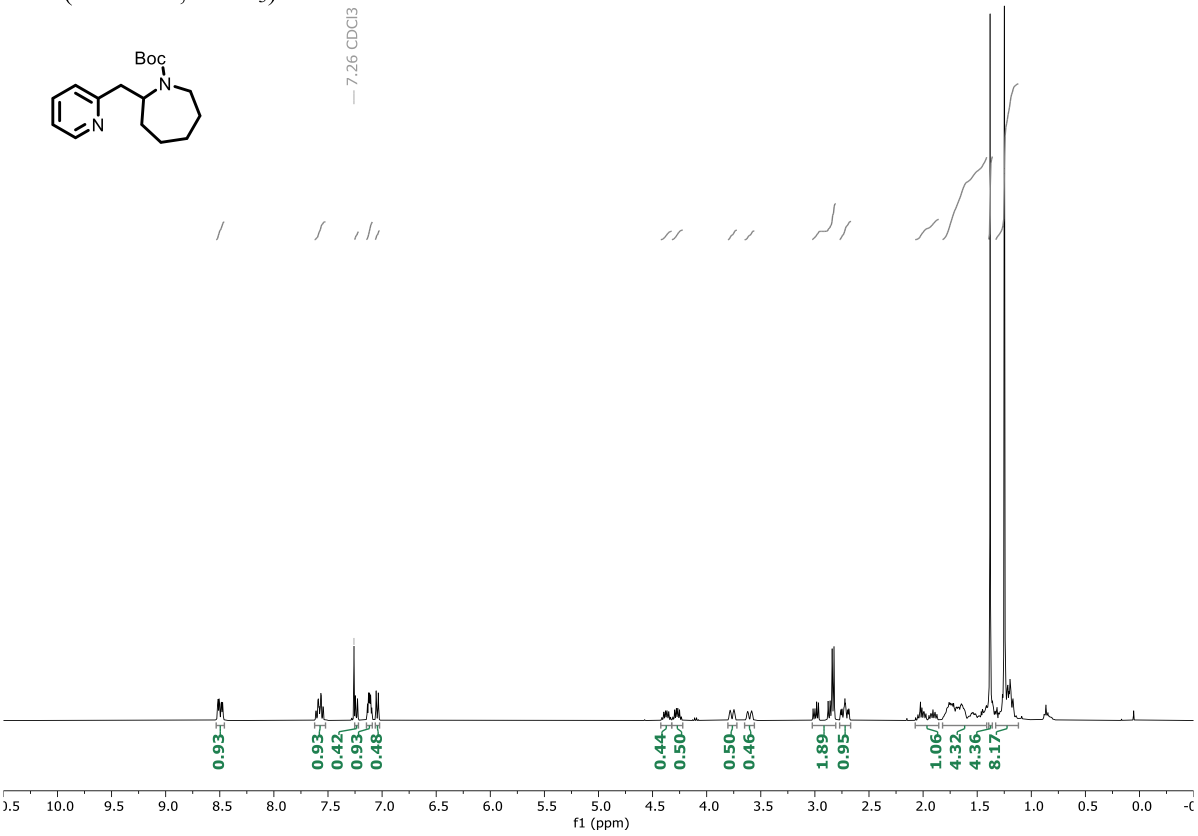

^{13}C NMR (101 MHz, $CDCl_3$) of **47**

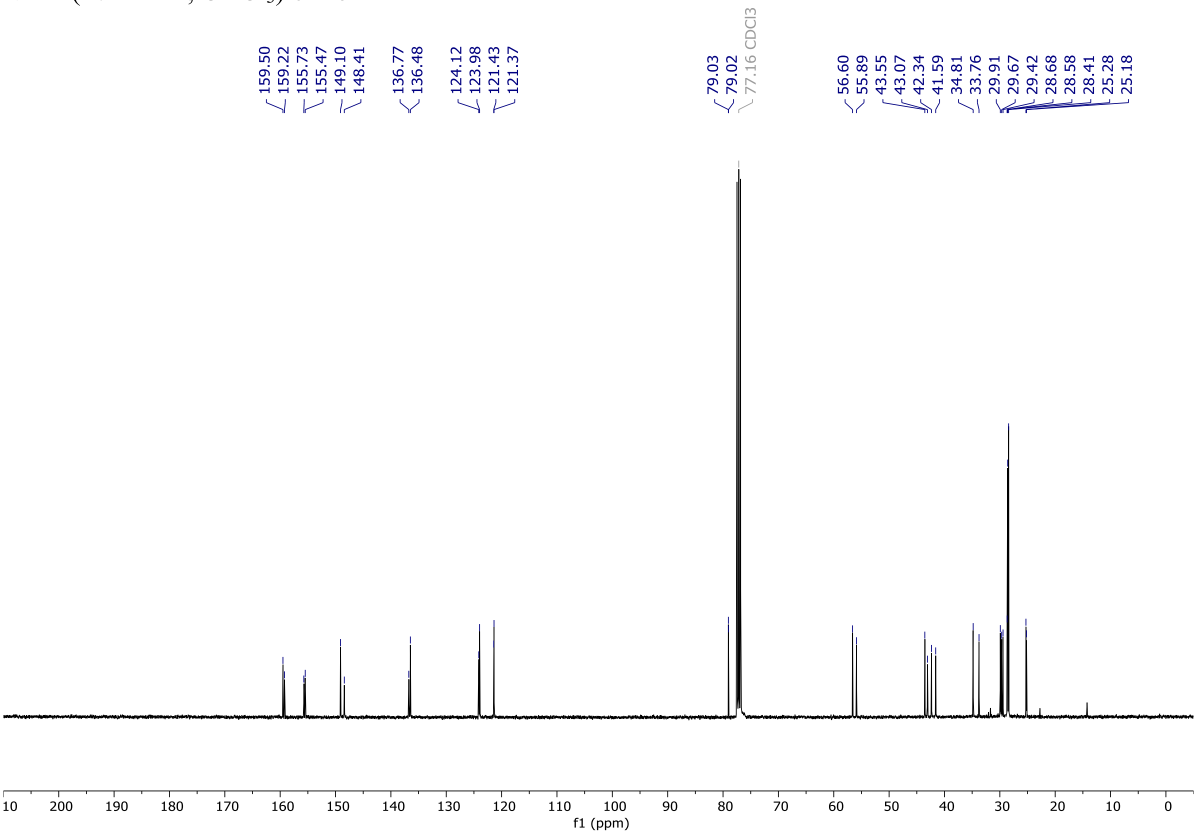

^{1}H NMR (400 MHz,$CDCl_3$) of **48**

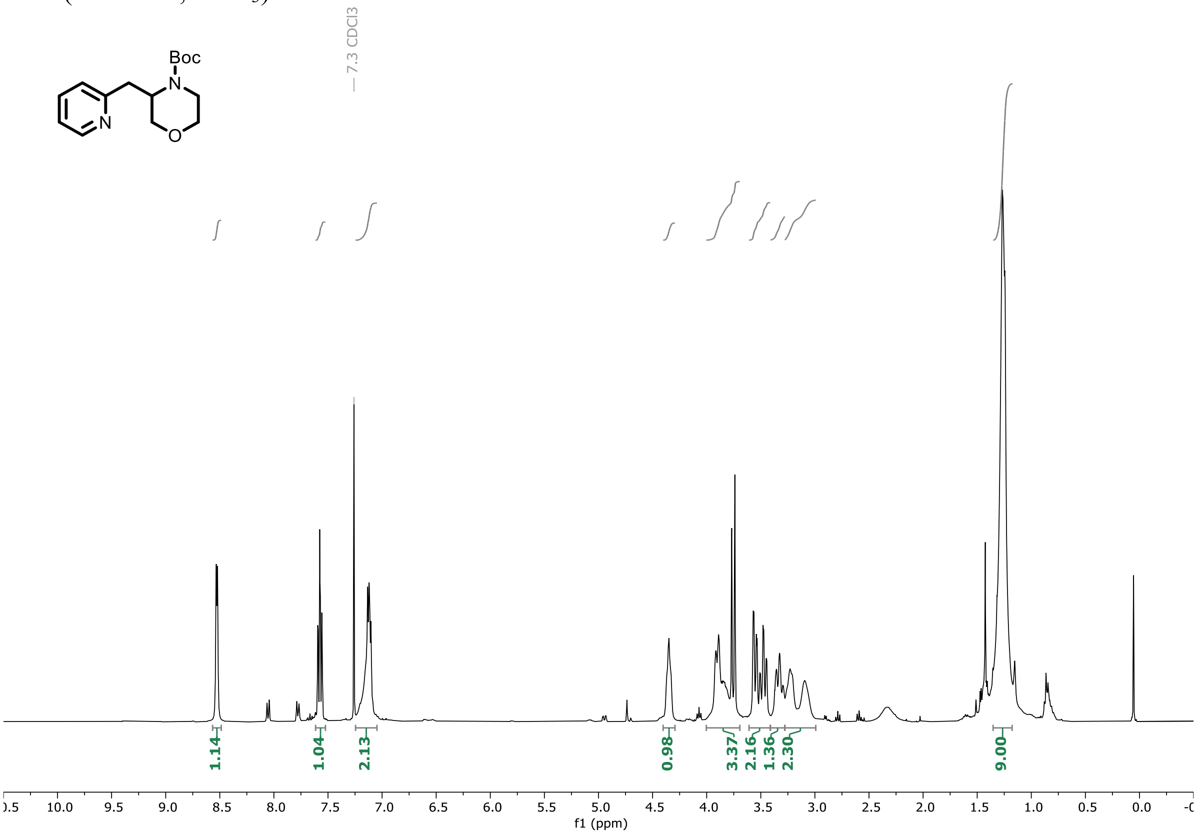

^{13}C NMR (101 MHz, $CDCl_3$) of **48**

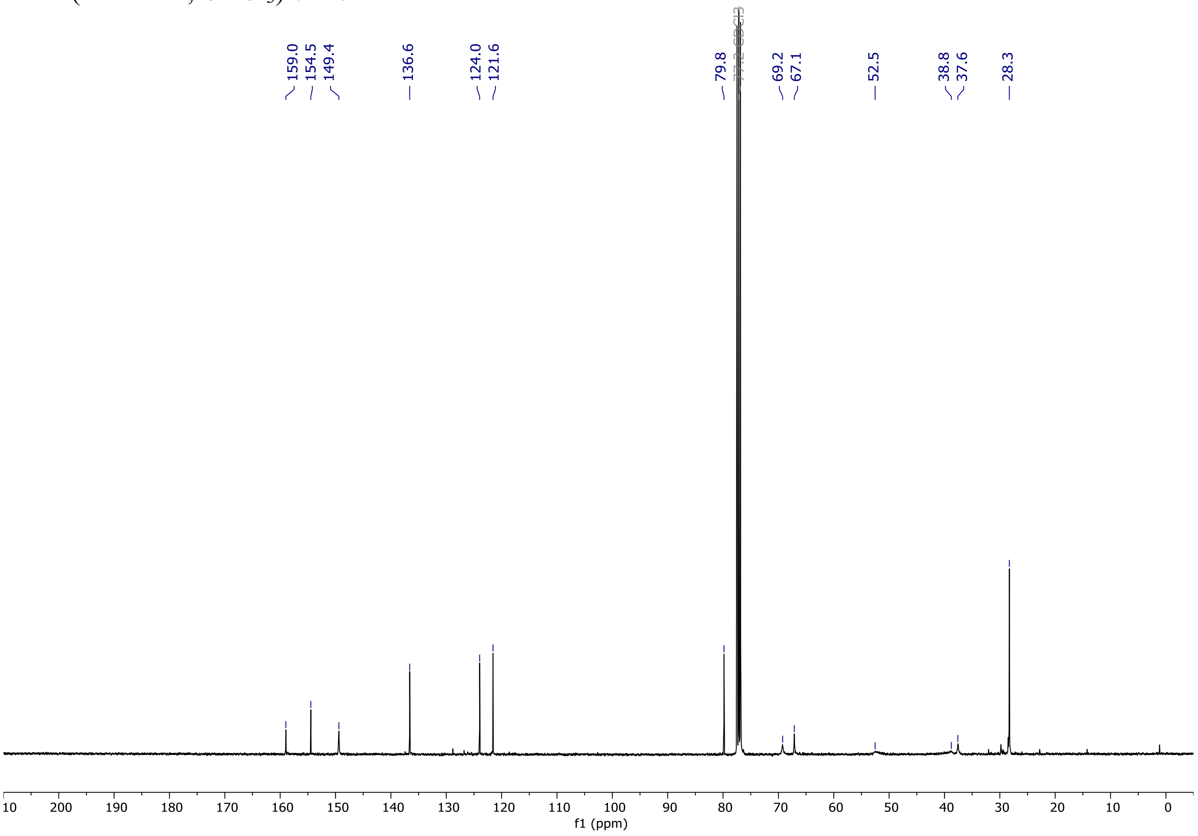

^{1}H NMR (400 MHz, $CDCl_3$) of **49**

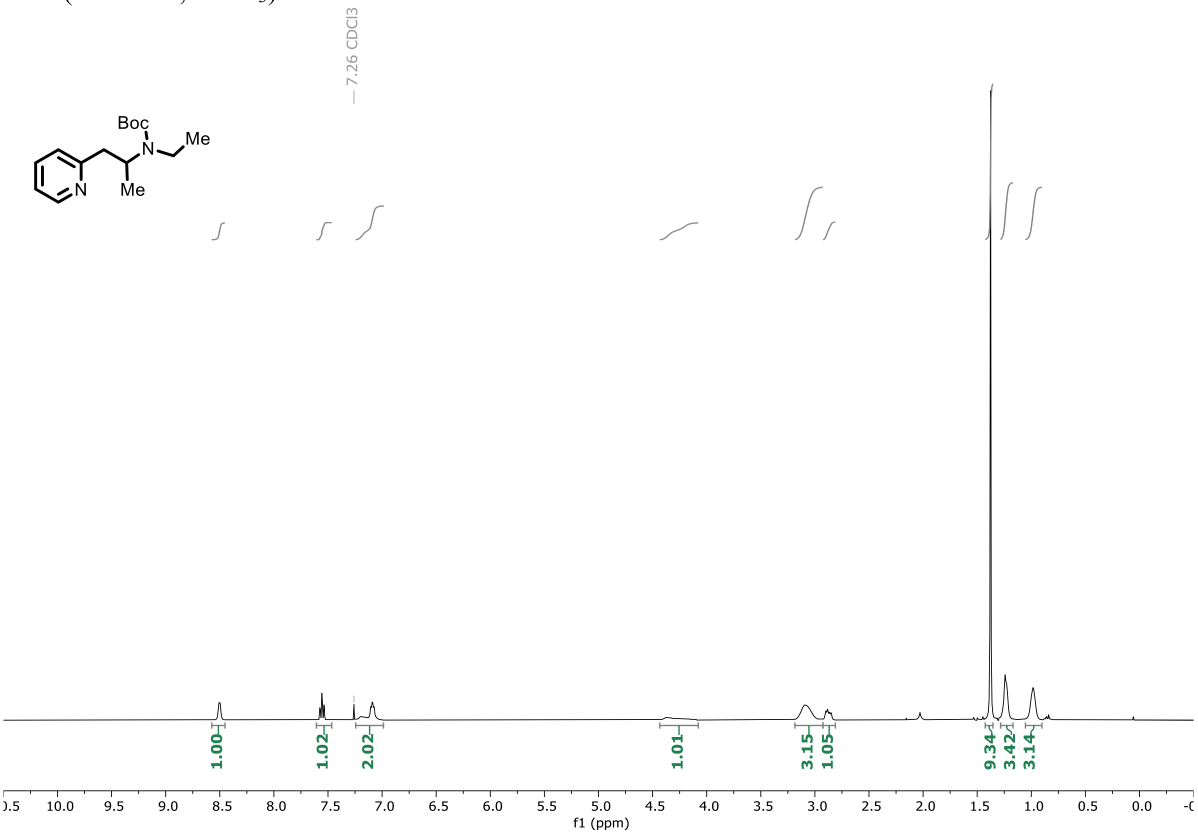

^{13}C NMR (101 MHz, $CDCl_3$) of **49**

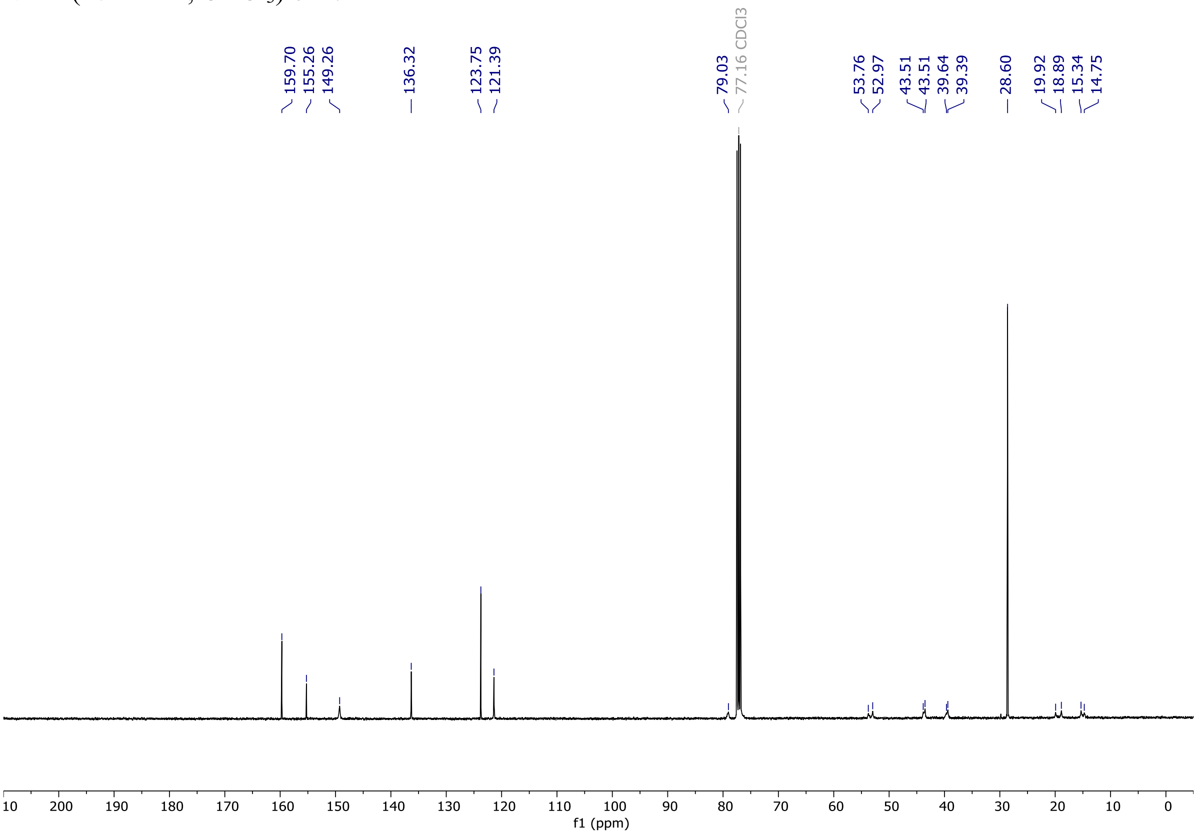

^{1}H NMR (400 MHz, $CDCl_3$) of **50**

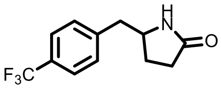

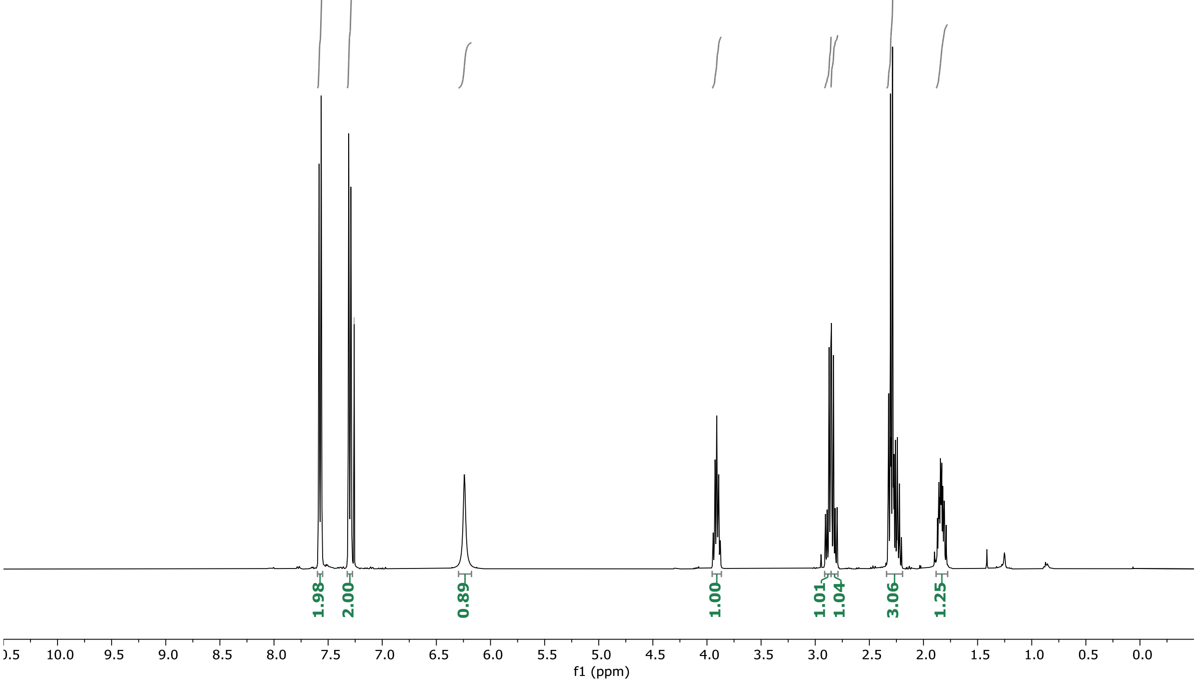

^{13}C NMR (101 MHz, $CDCl_3$) of **50**

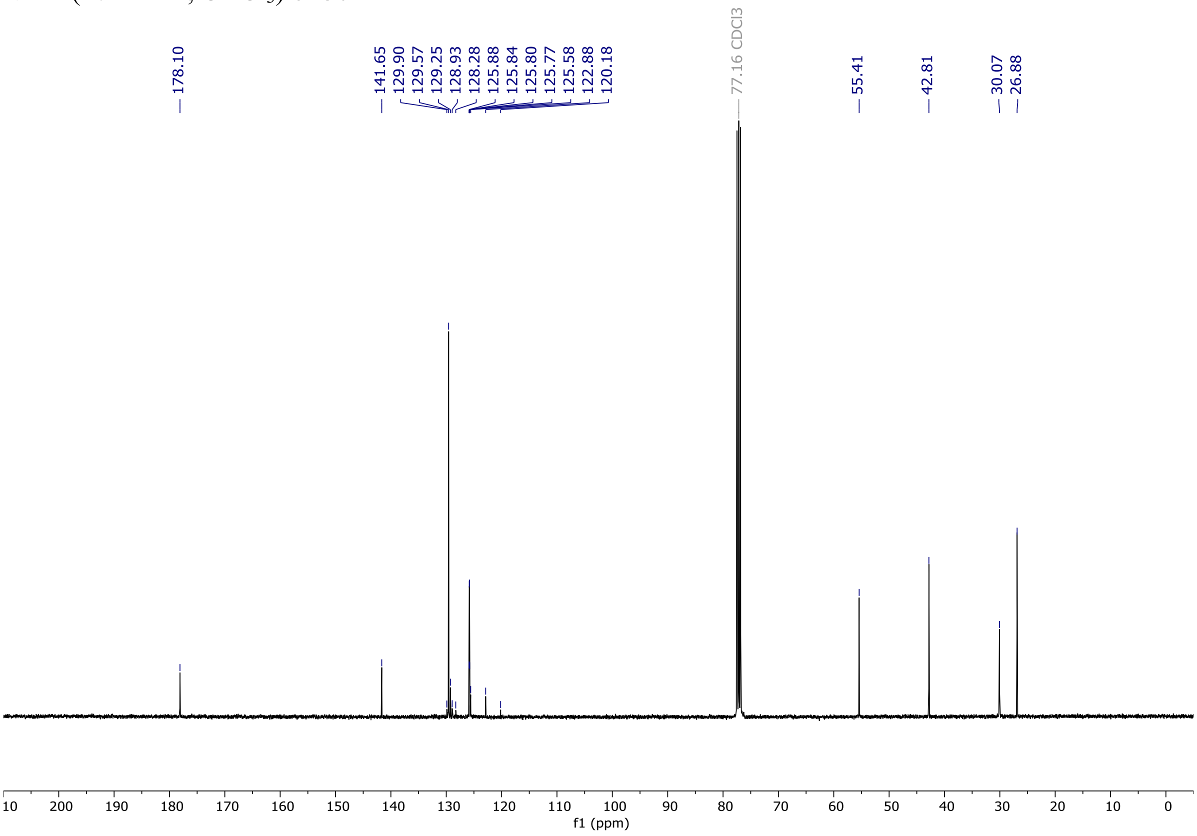

^{19}F NMR (282 MHz, $CDCl_3$) of **50**

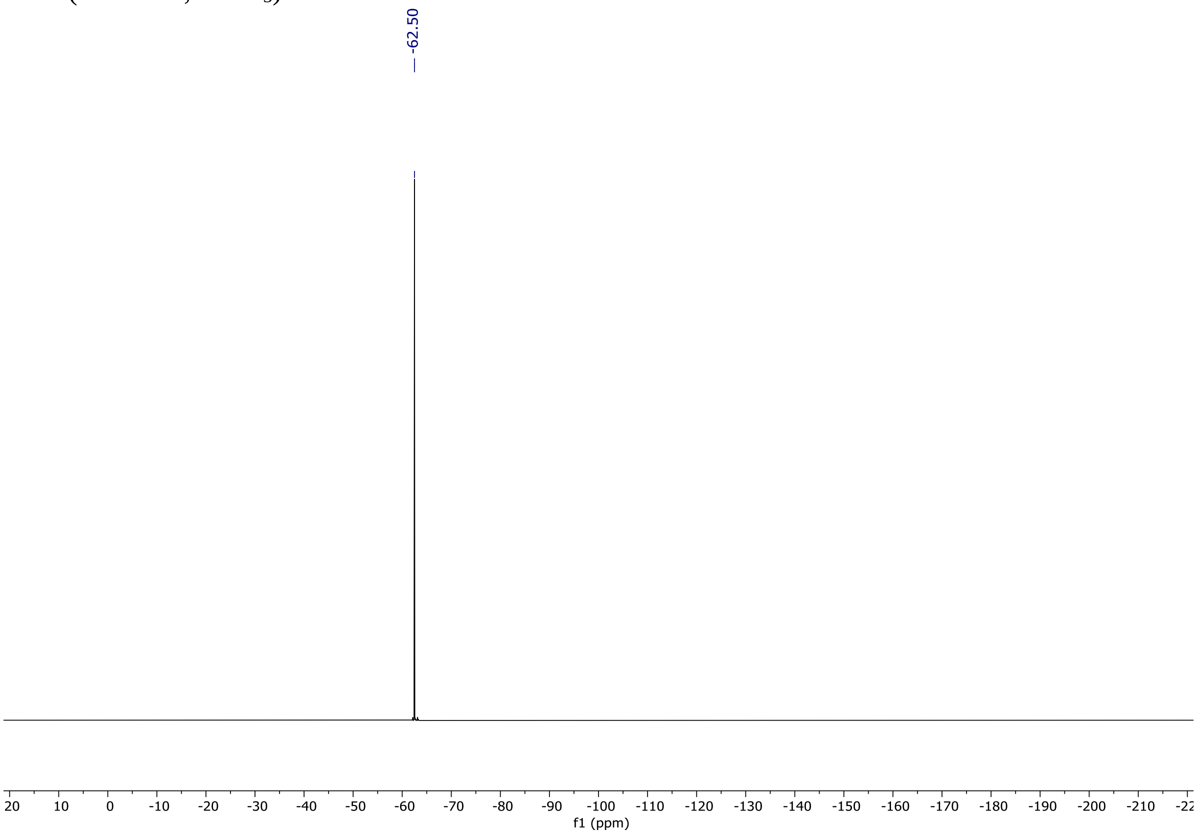

^{1}H NMR (400 MHz,CDCl$_3$) of **51**

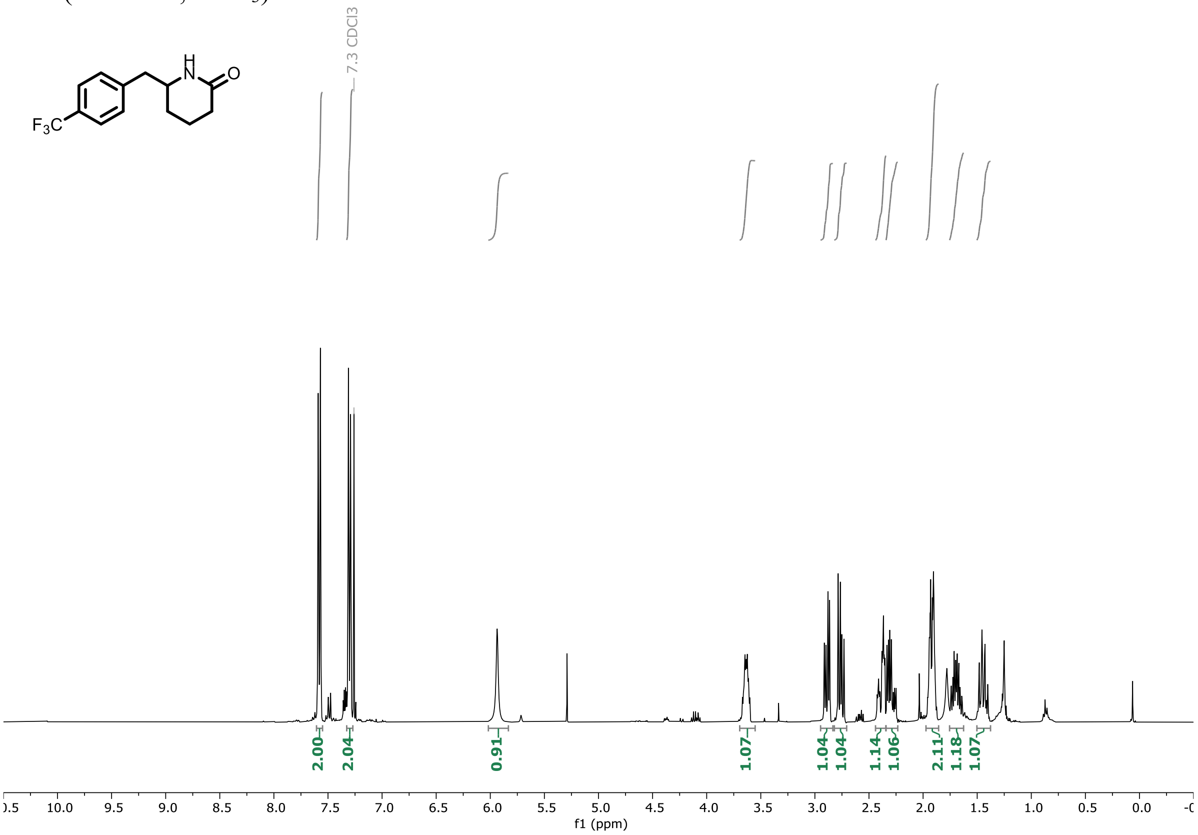

^{13}C NMR (101 MHz, CDCl$_3$) of **51**

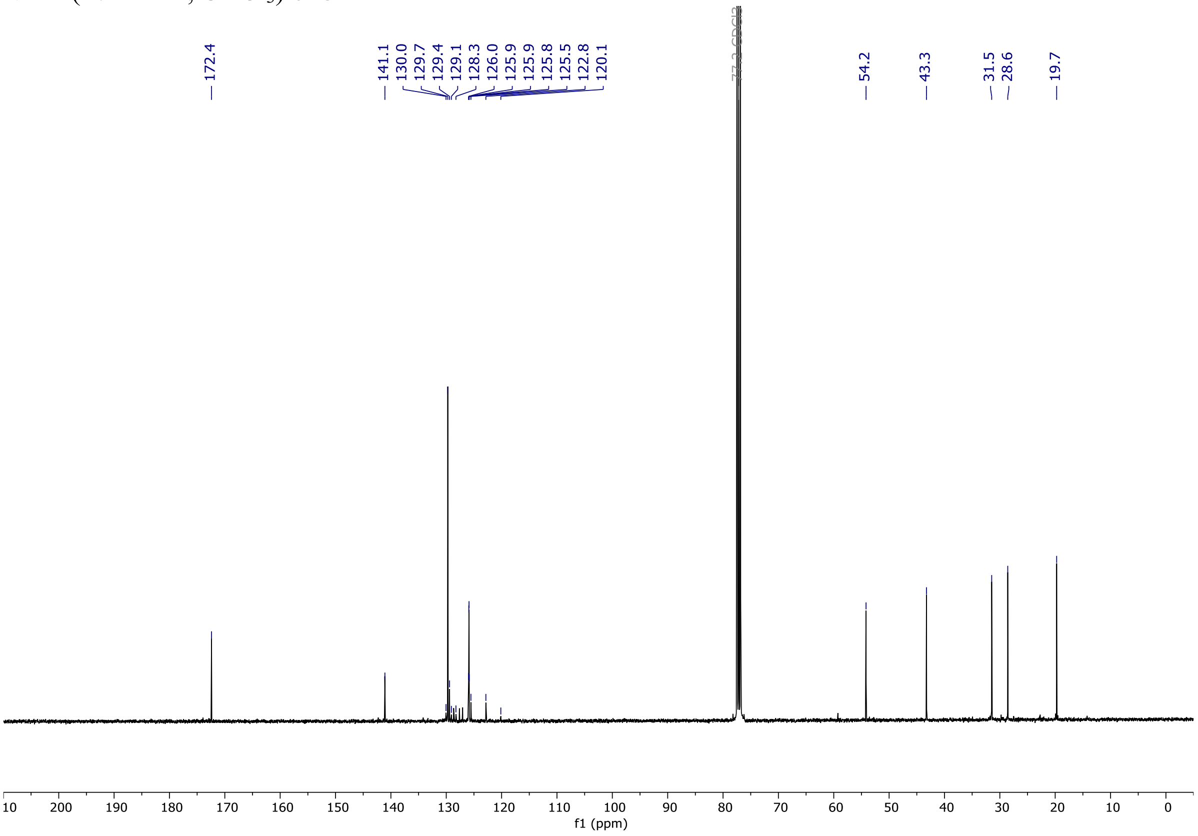

^{19}F NMR (282 MHz, $CDCl_3$) of **51**

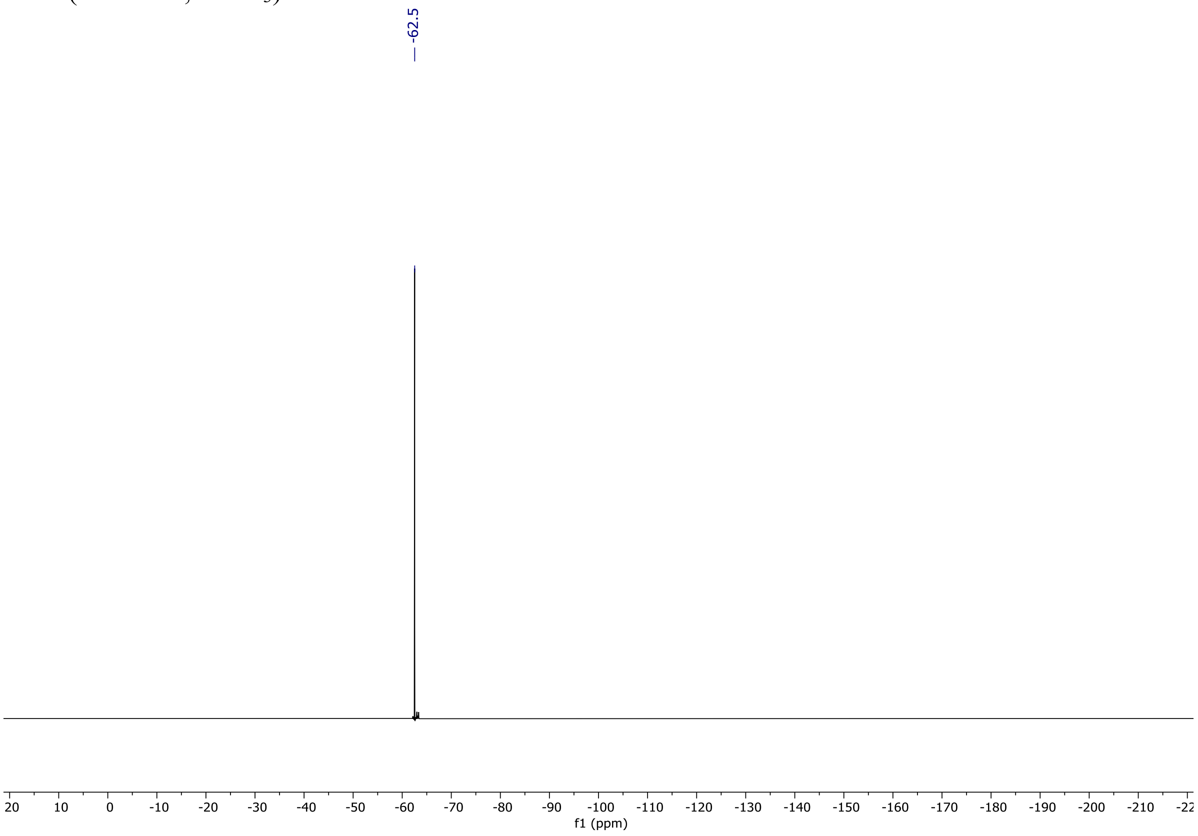

^{1}H NMR (400 MHz,CDCl$_3$) of **52**

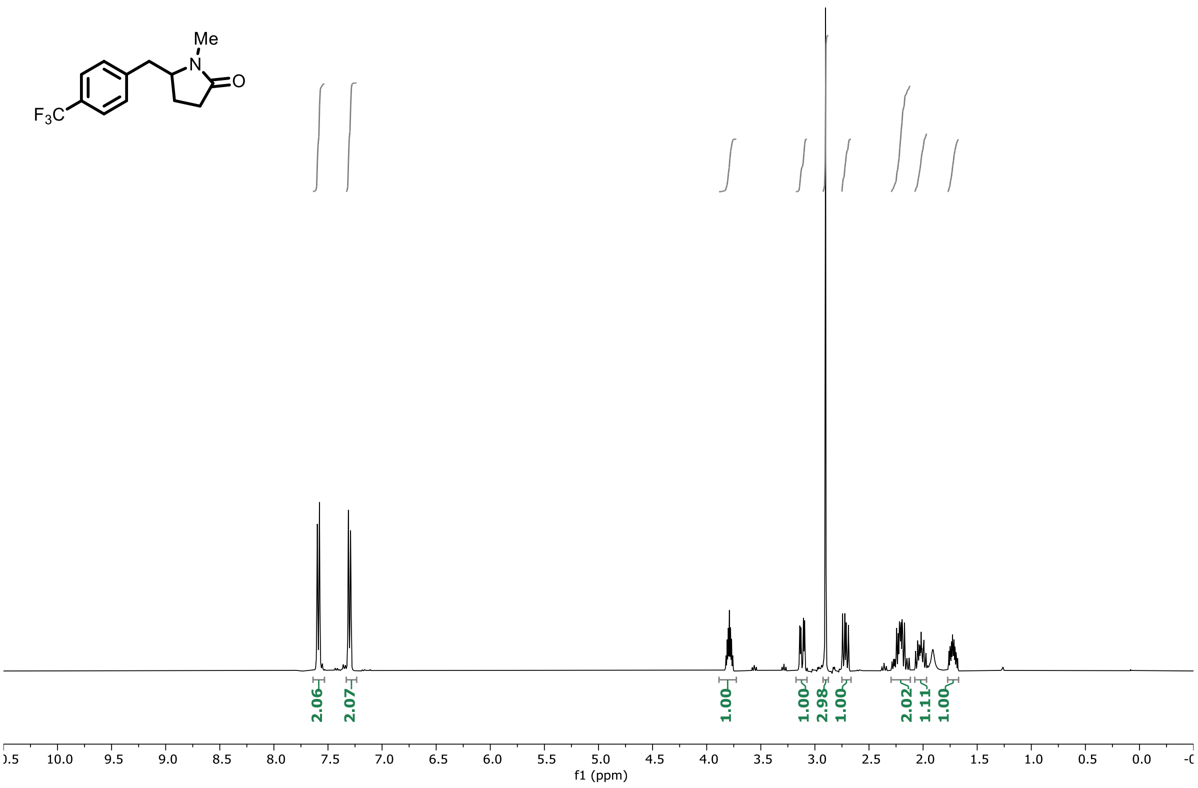

^{13}C NMR (101 MHz, CDCl$_3$) of **52**

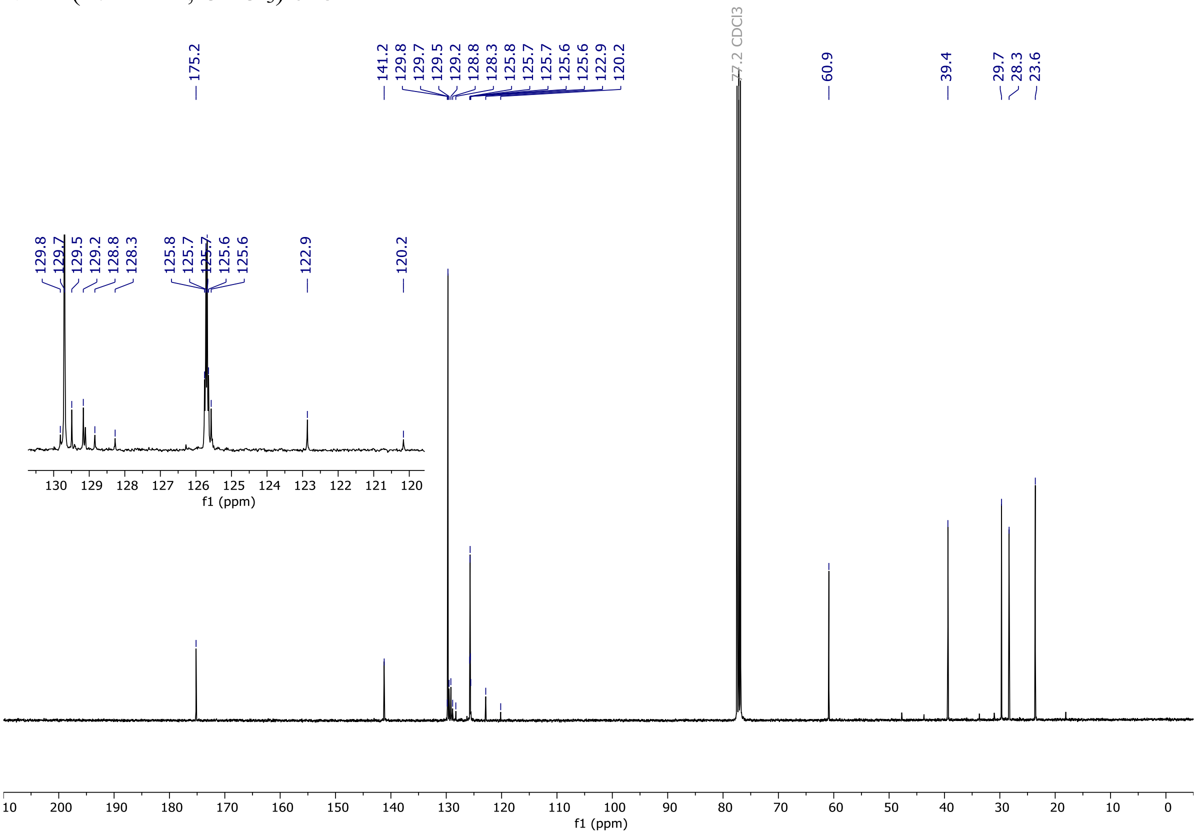

^{19}F NMR (282 MHz, $CDCl_3$) of **52**

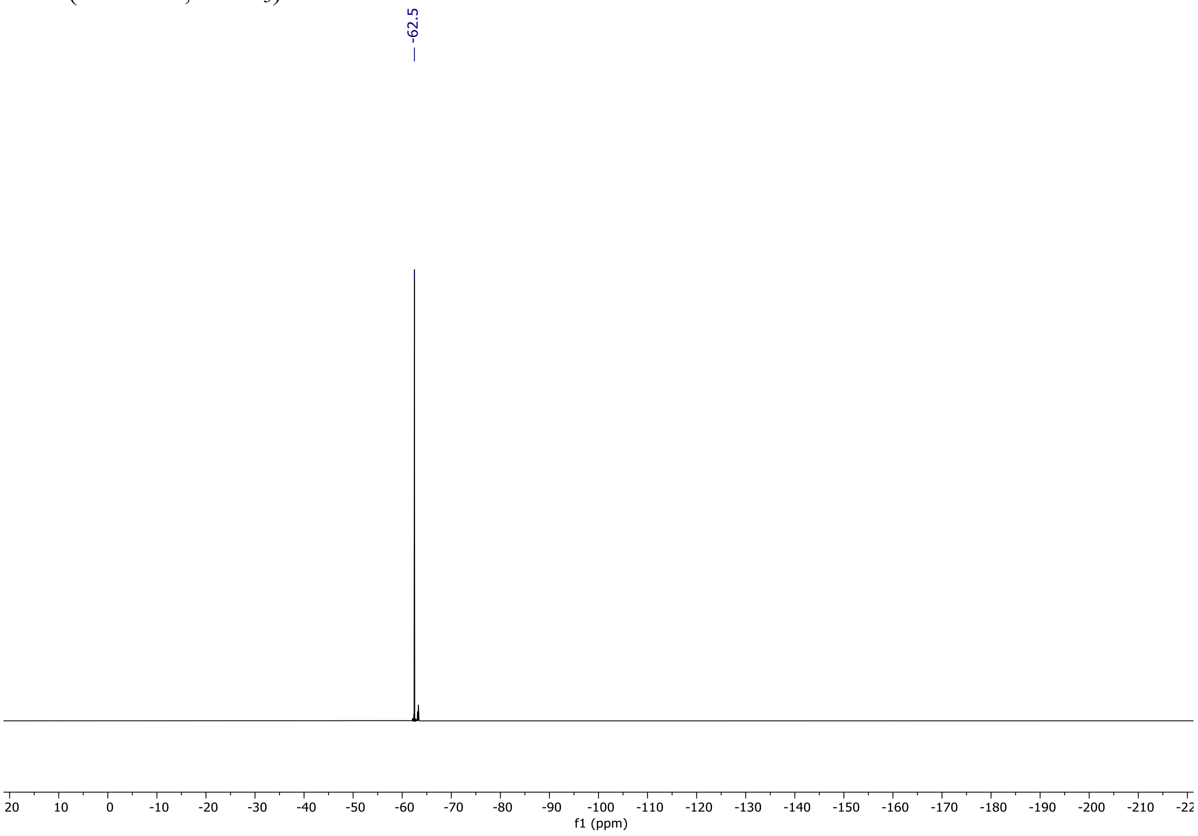

^{1}H NMR (400 MHz, $CDCl_3$) of **53**

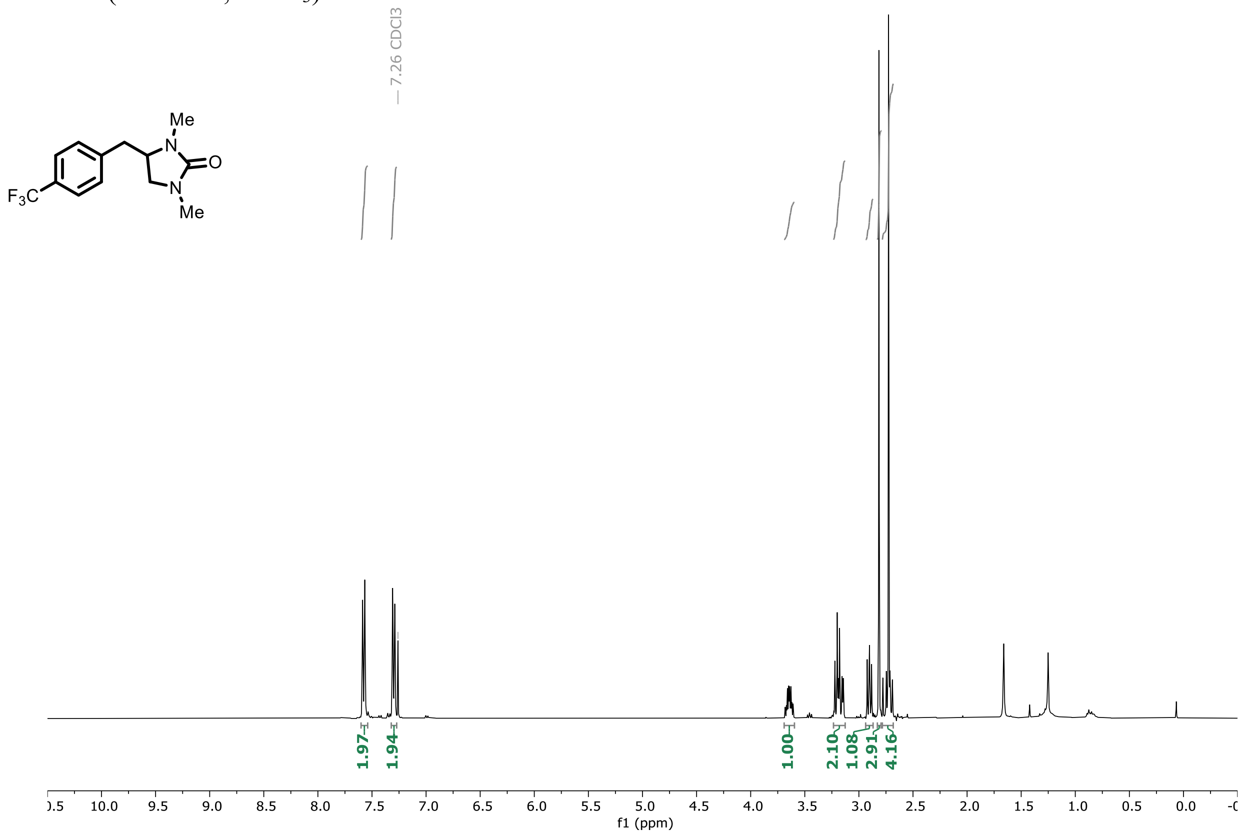

^{13}C NMR (101 MHz, $CDCl_3$) of **53**

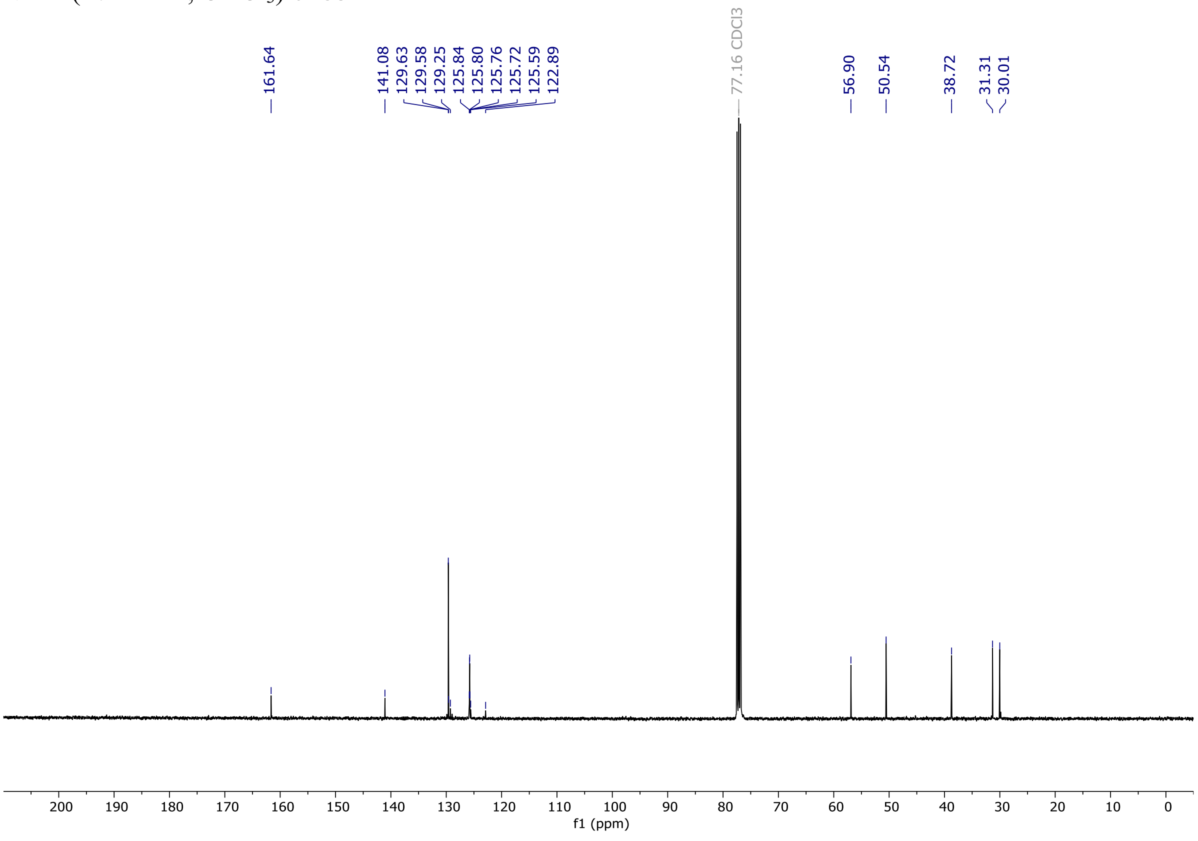

^{19}F NMR (282 MHz, $CDCl_3$) of **53**

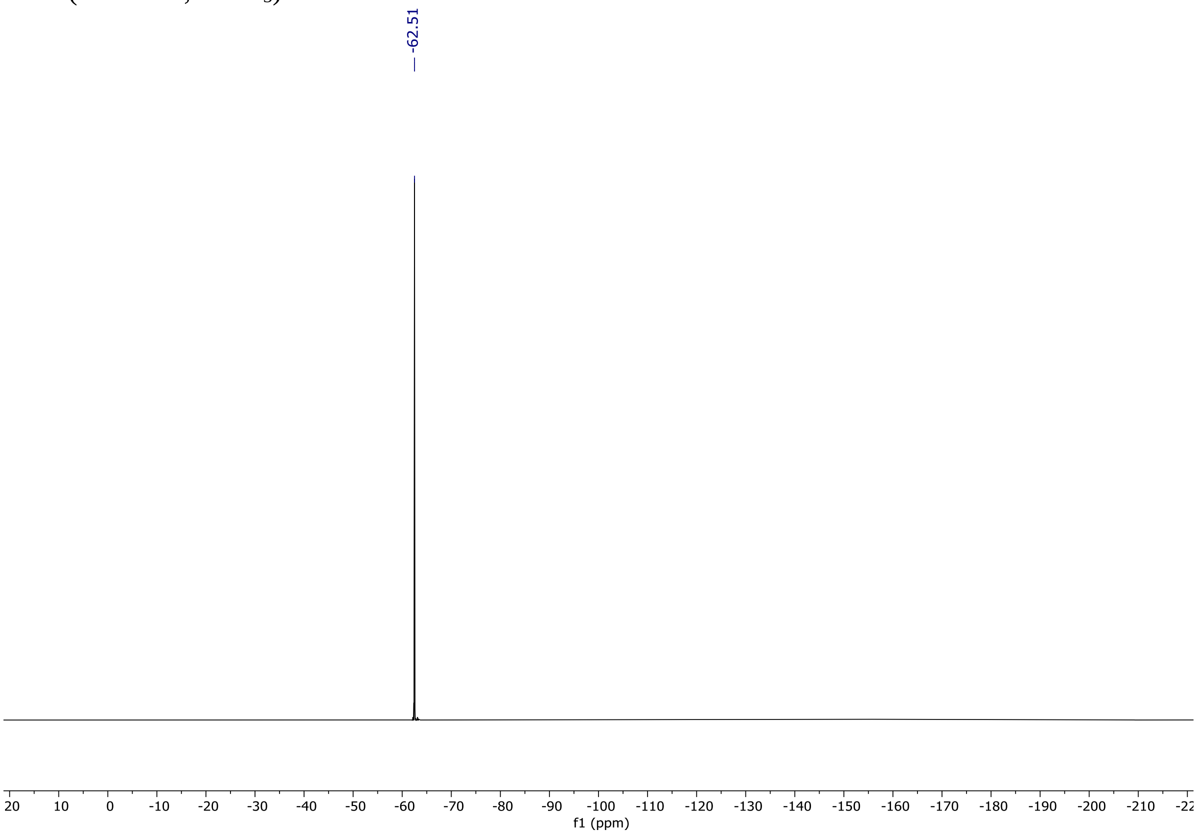

^{1}H NMR (400 MHz, $CDCl_3$) of **54**

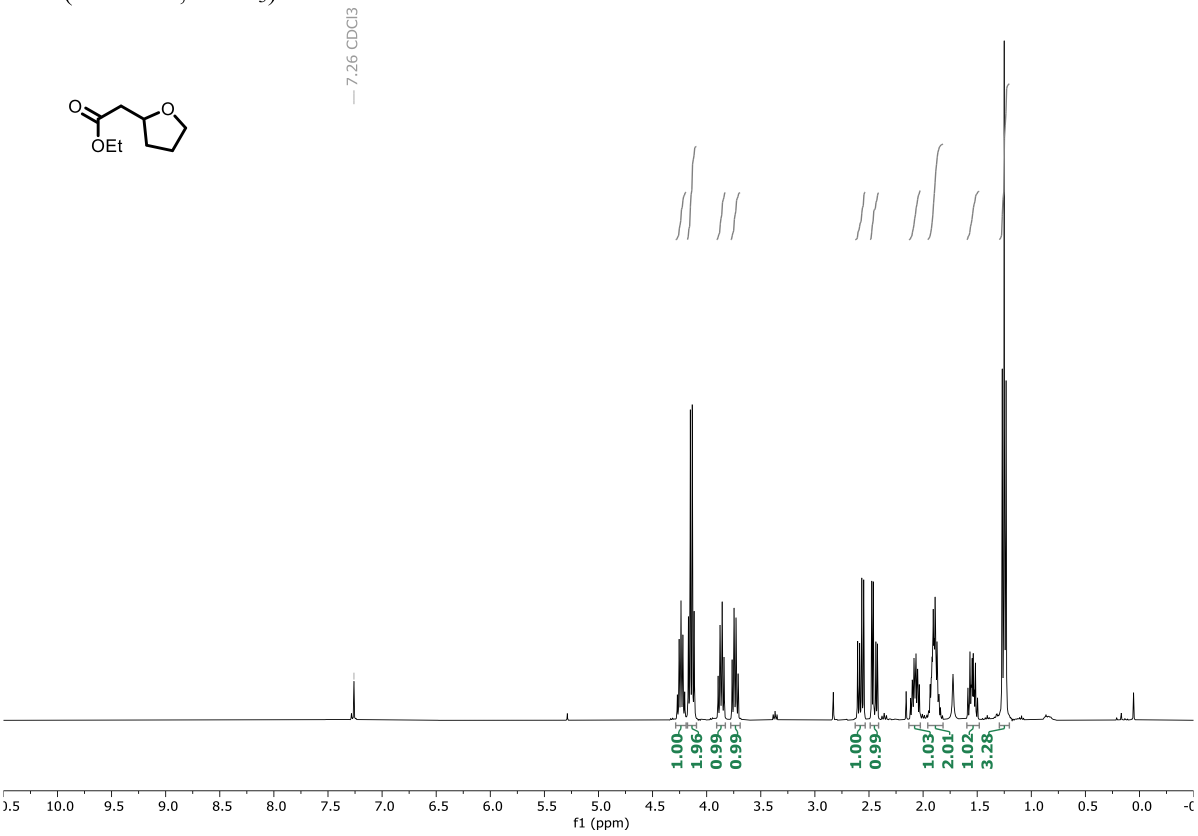

^{13}C NMR (101 MHz, $CDCl_3$) of **54**

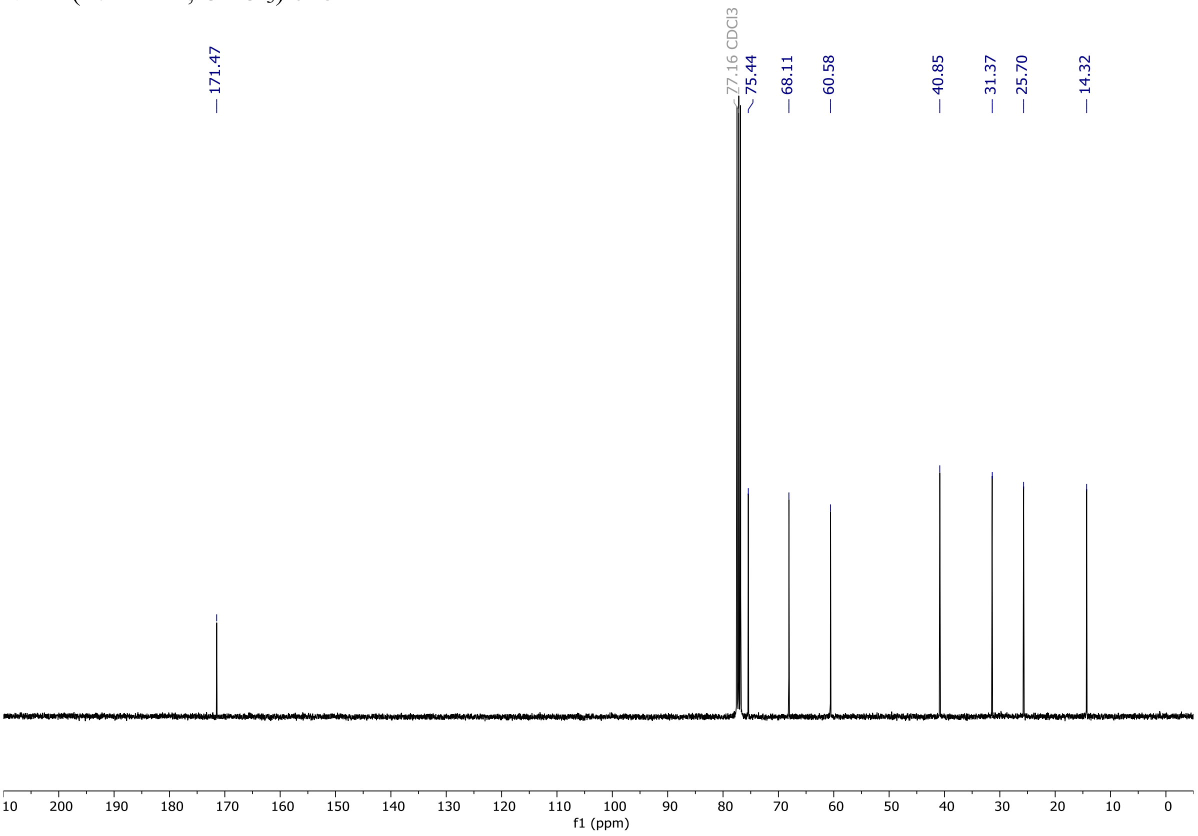

^{1}H NMR (400 MHz,CDCl$_3$) of **55**

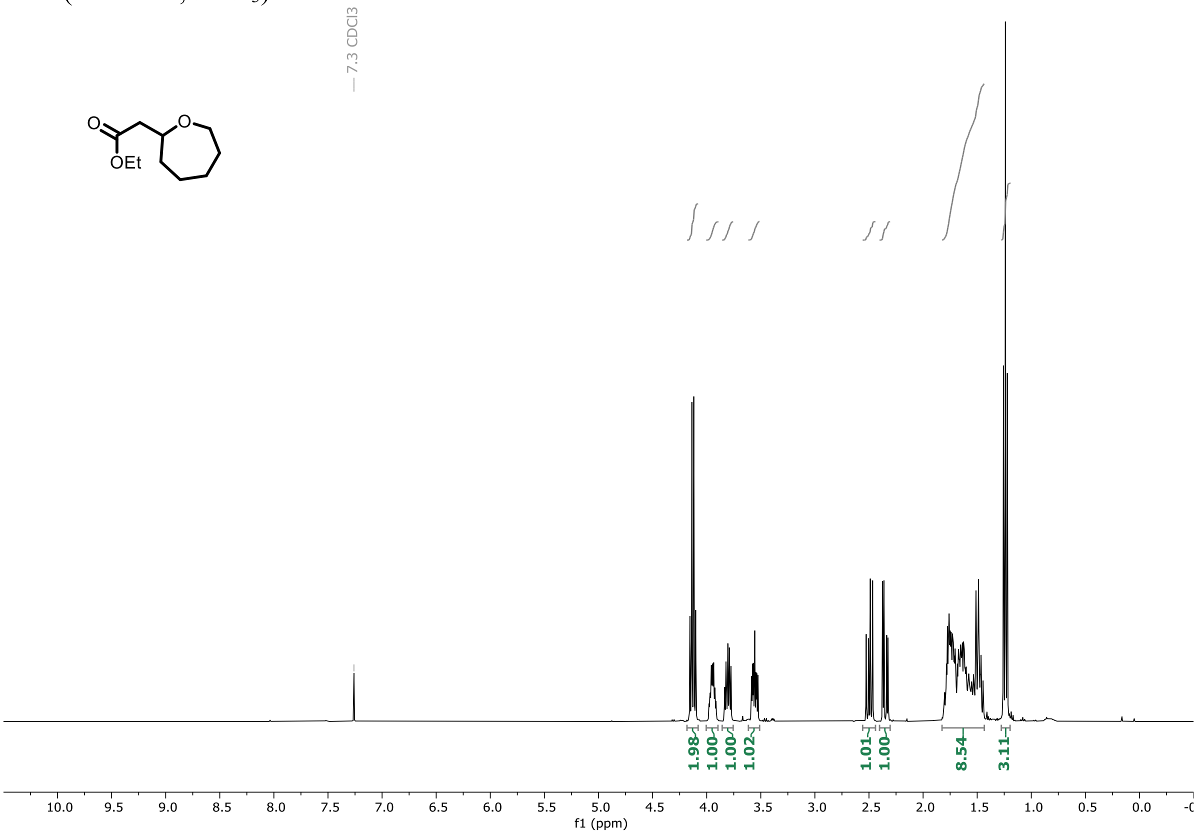

^{13}C NMR (101 MHz, CDCl$_3$) of **55**

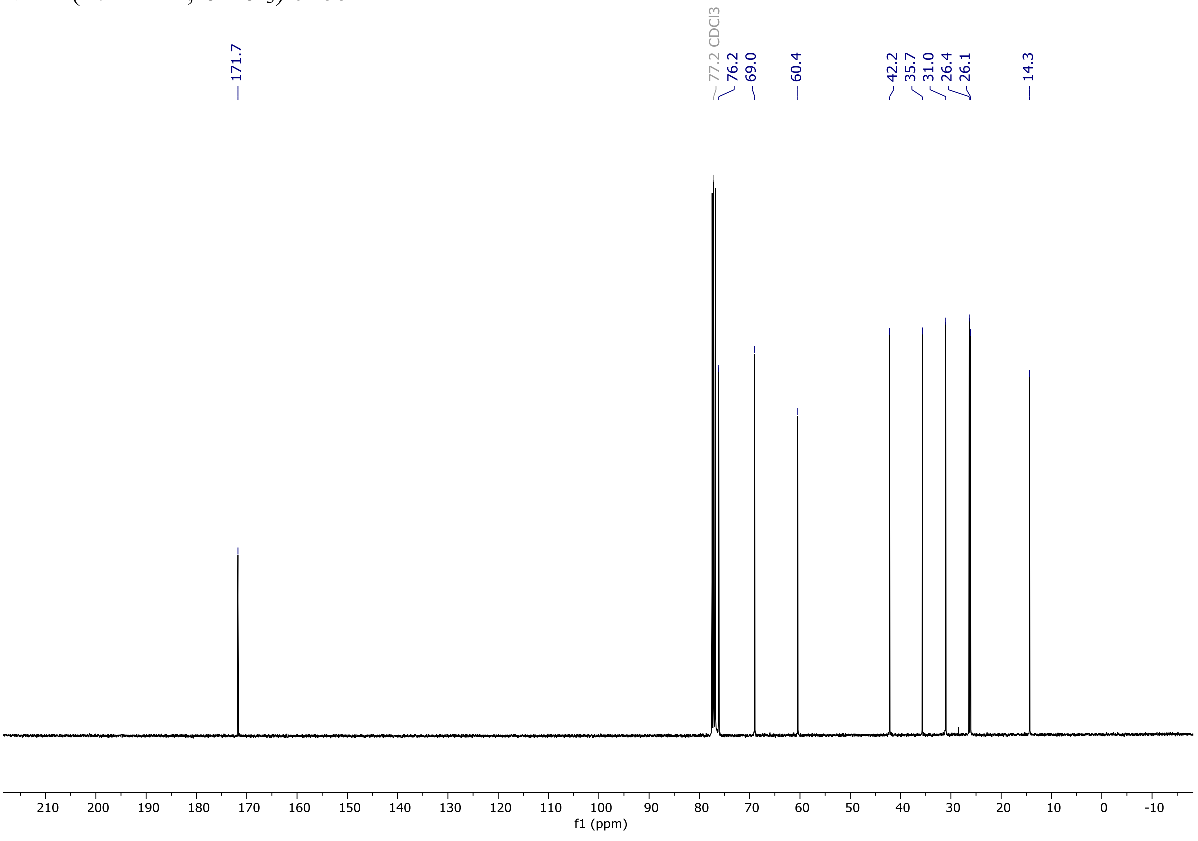

^{1}H NMR (400 MHz, $CDCl_3$) of **56**

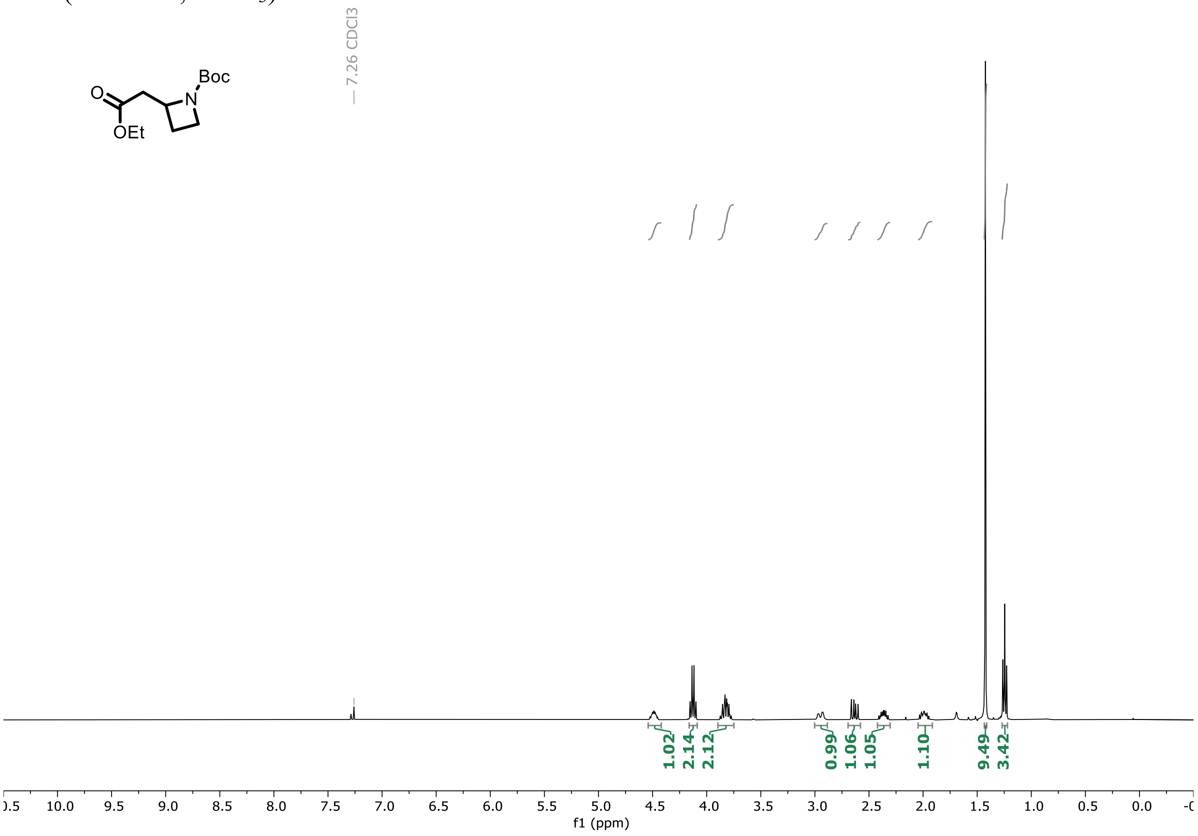

^{13}C NMR (101 MHz, $CDCl_3$) of **56**

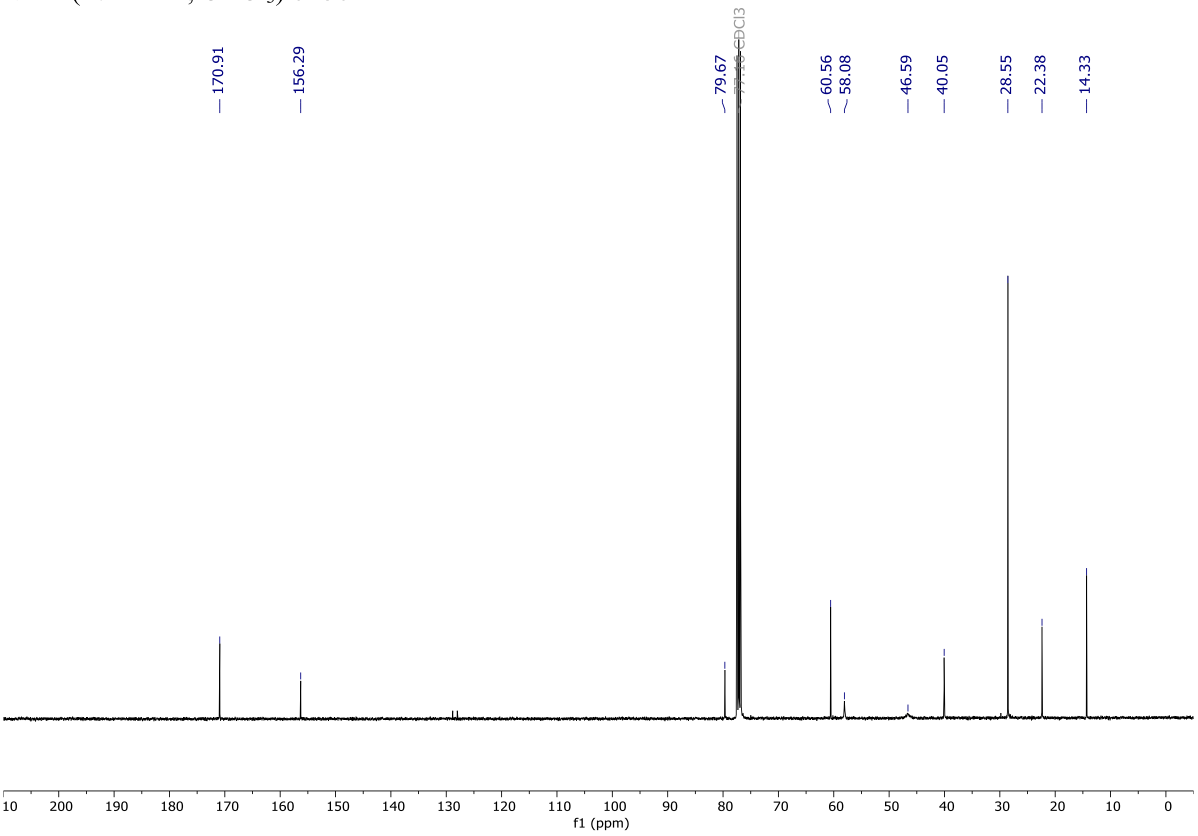

^{1}H NMR (400 MHz, $CDCl_3$) of **57**

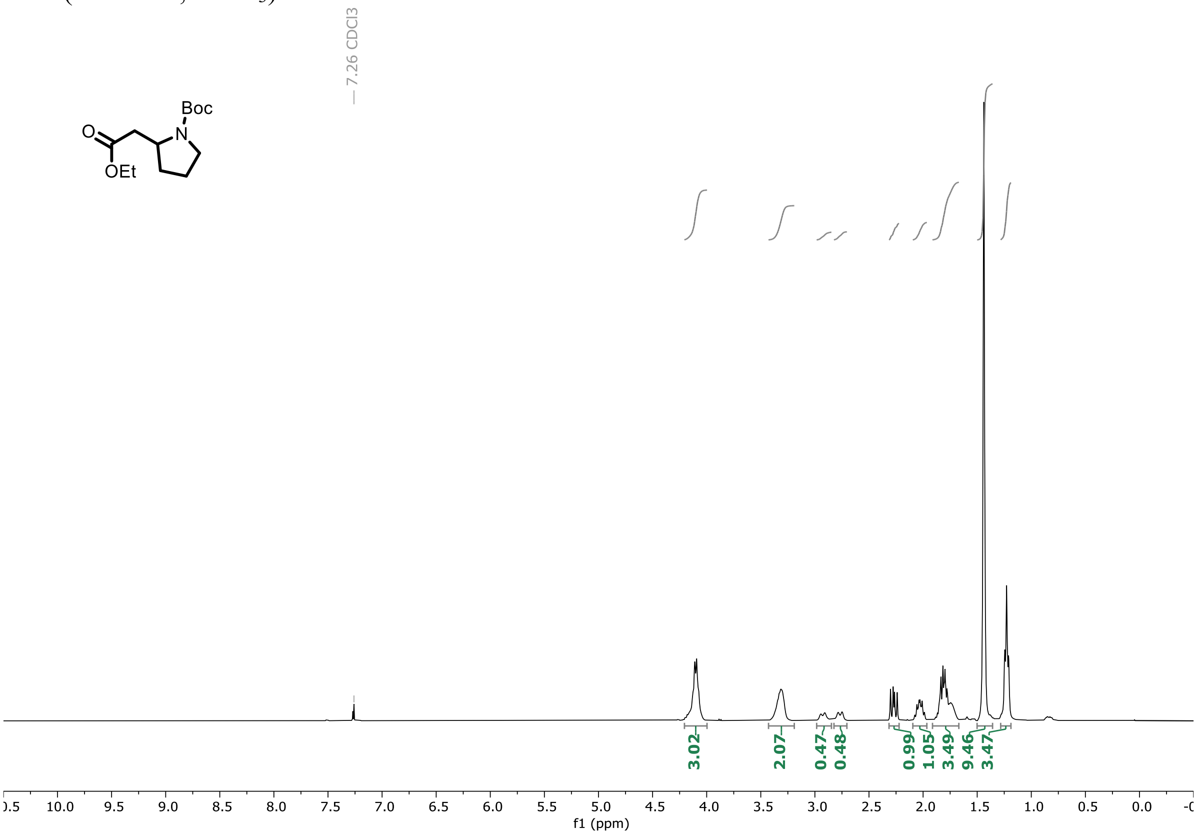

^{13}C NMR (101 MHz, $CDCl_3$) of **57**

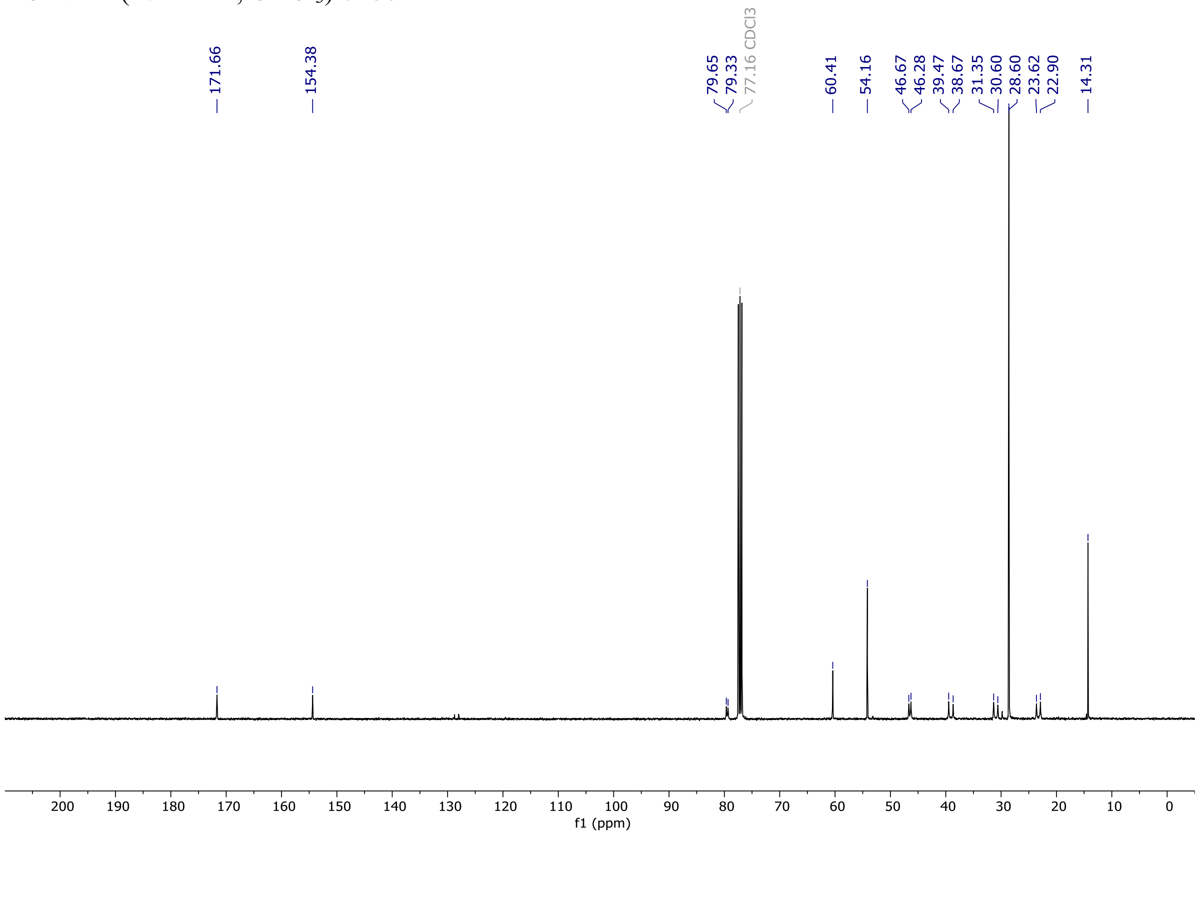

^{1}H NMR (400 MHz,$CDCl_3$) of **58**

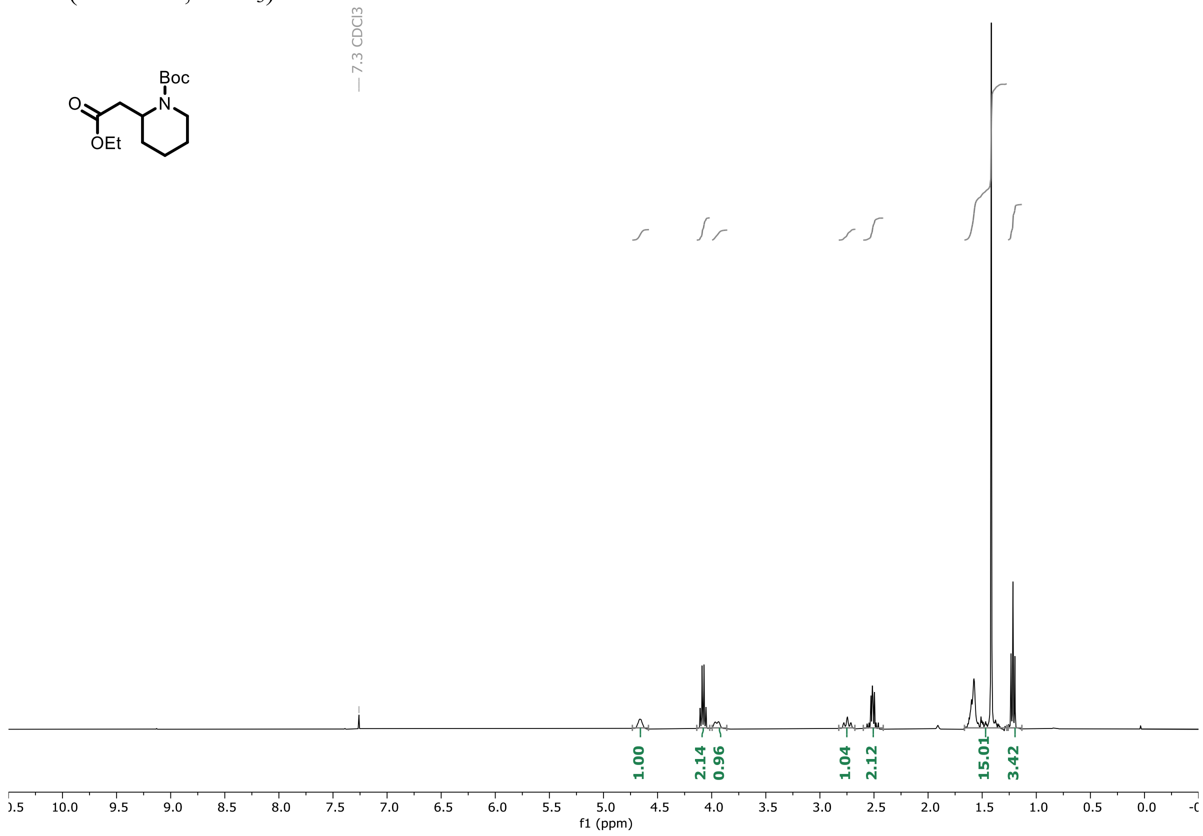

^{13}C NMR (101 MHz, $CDCl_3$) of **58**

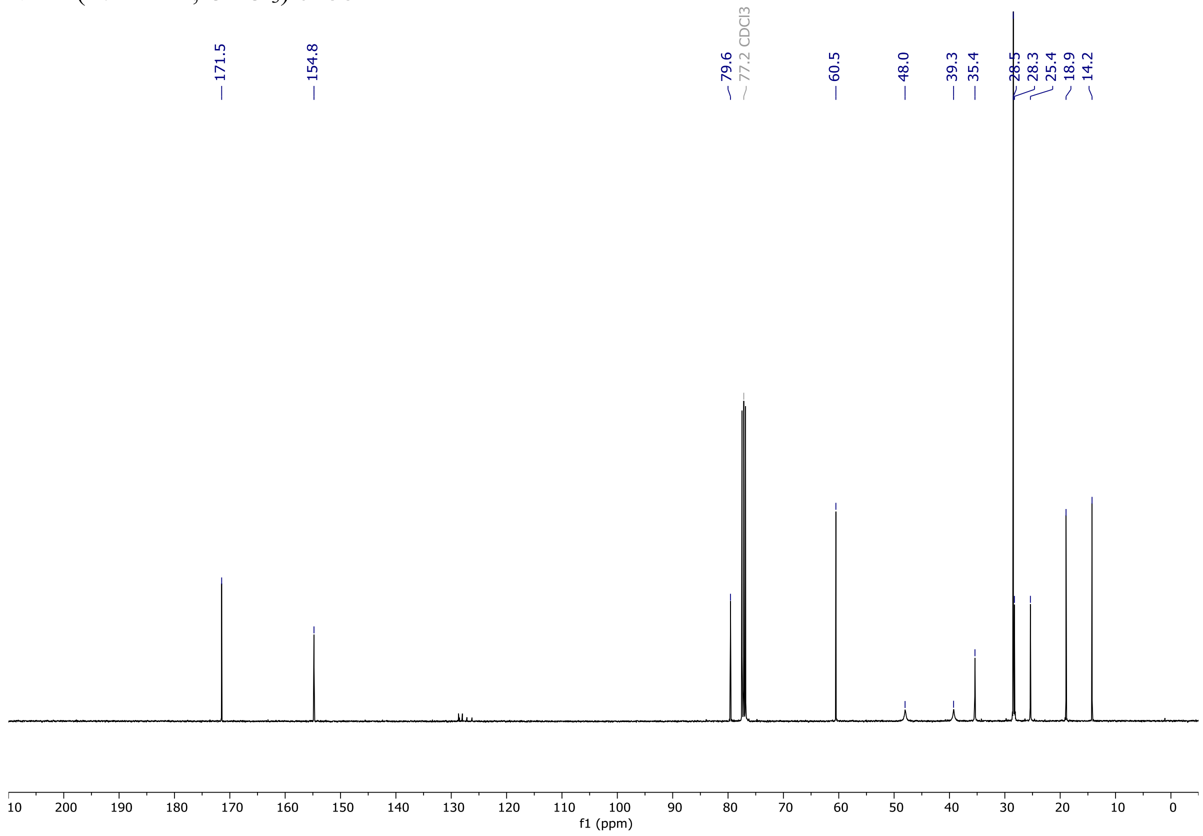

^{1}H NMR (400 MHz, $CDCl_3$) of **59**

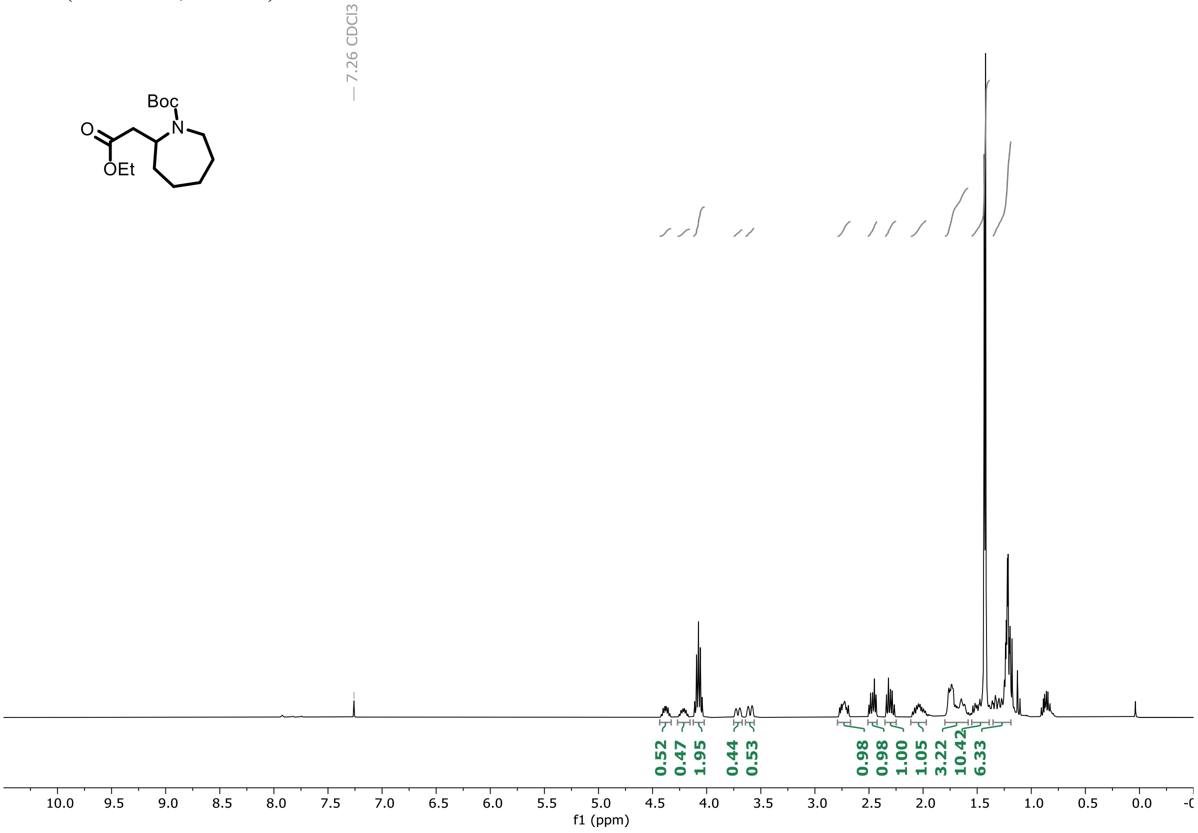

^{13}C NMR (101 MHz, $CDCl_3$) of **59**

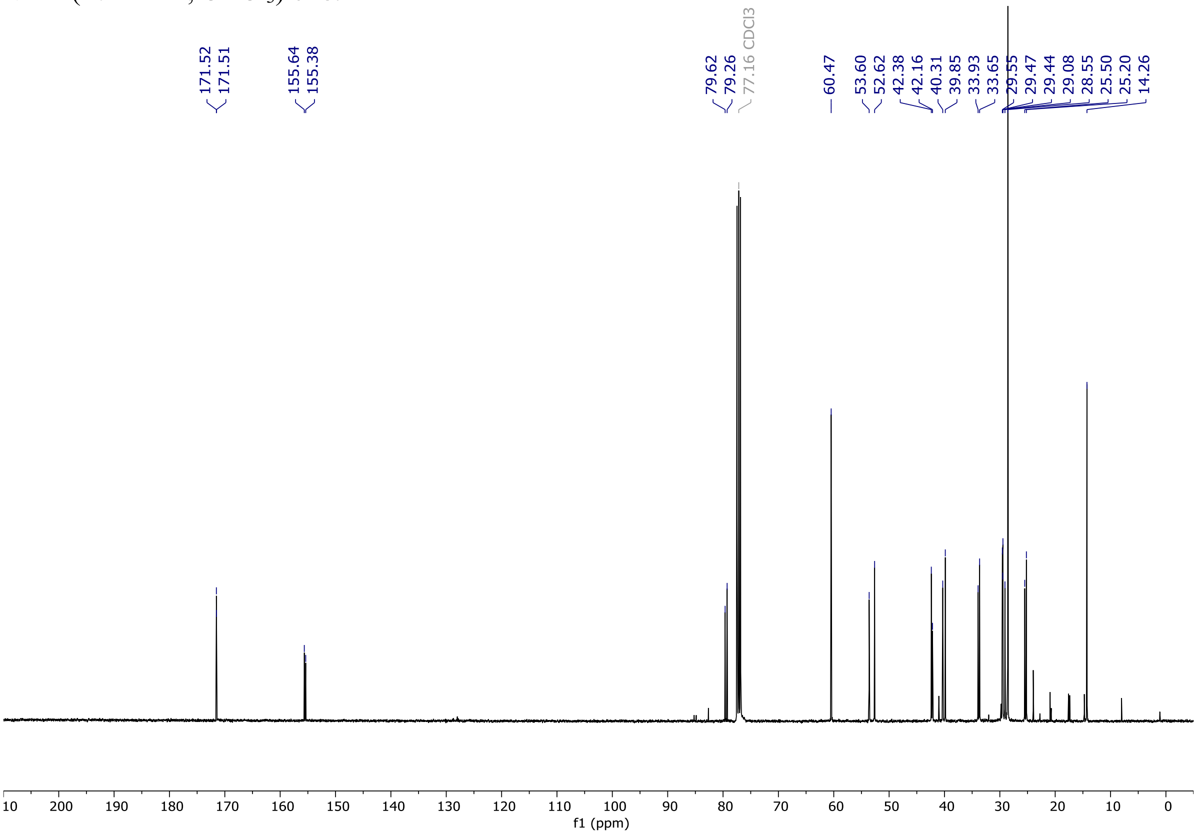

^{1}H NMR (400 MHz,CDCl$_3$) of **60**

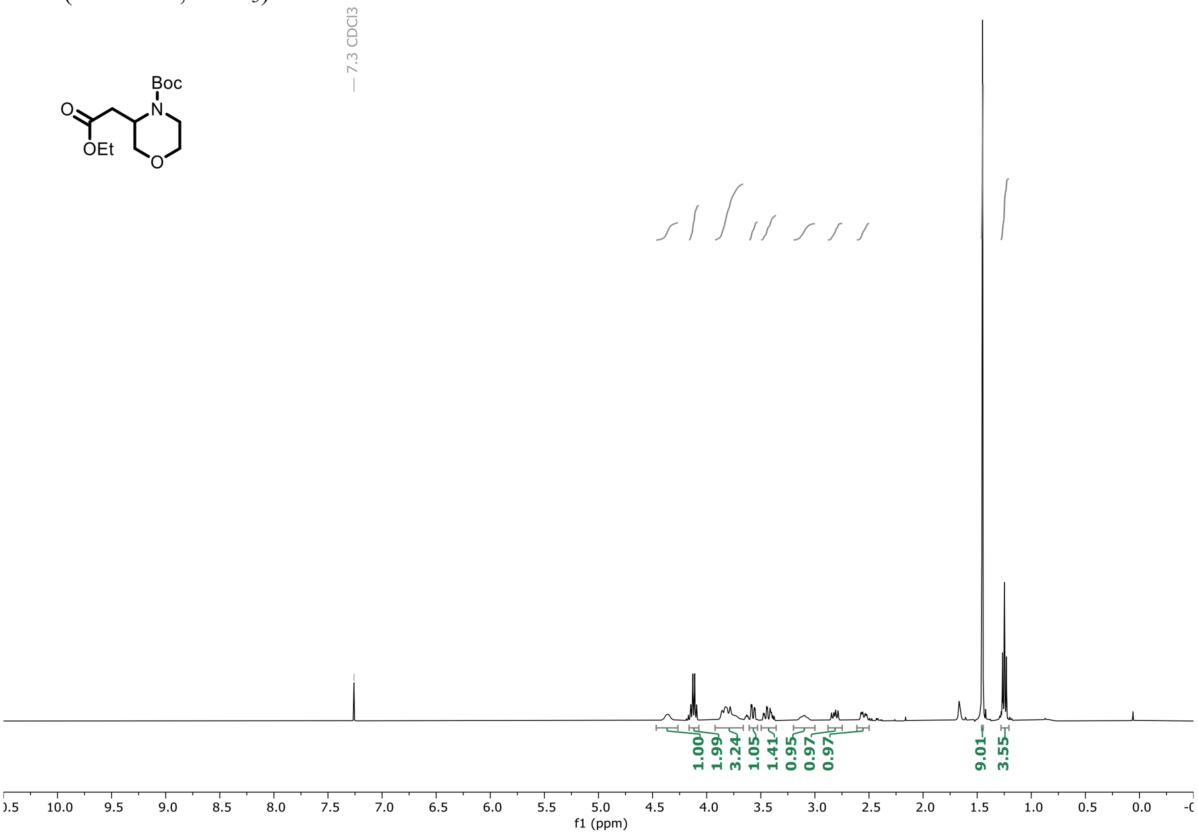

^{13}C NMR (101 MHz, CDCl$_3$) of **60**

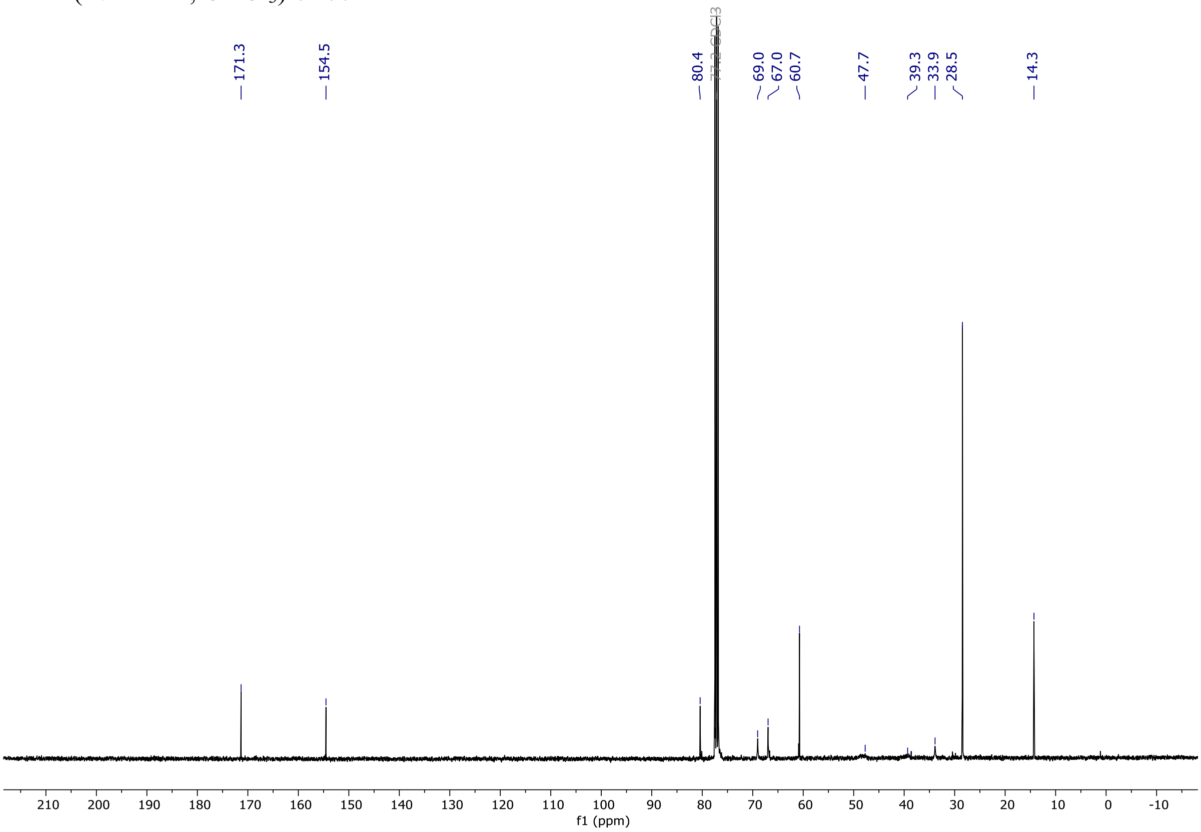

^{1}H NMR (400 MHz, $CDCl_3$) of **61**

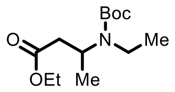

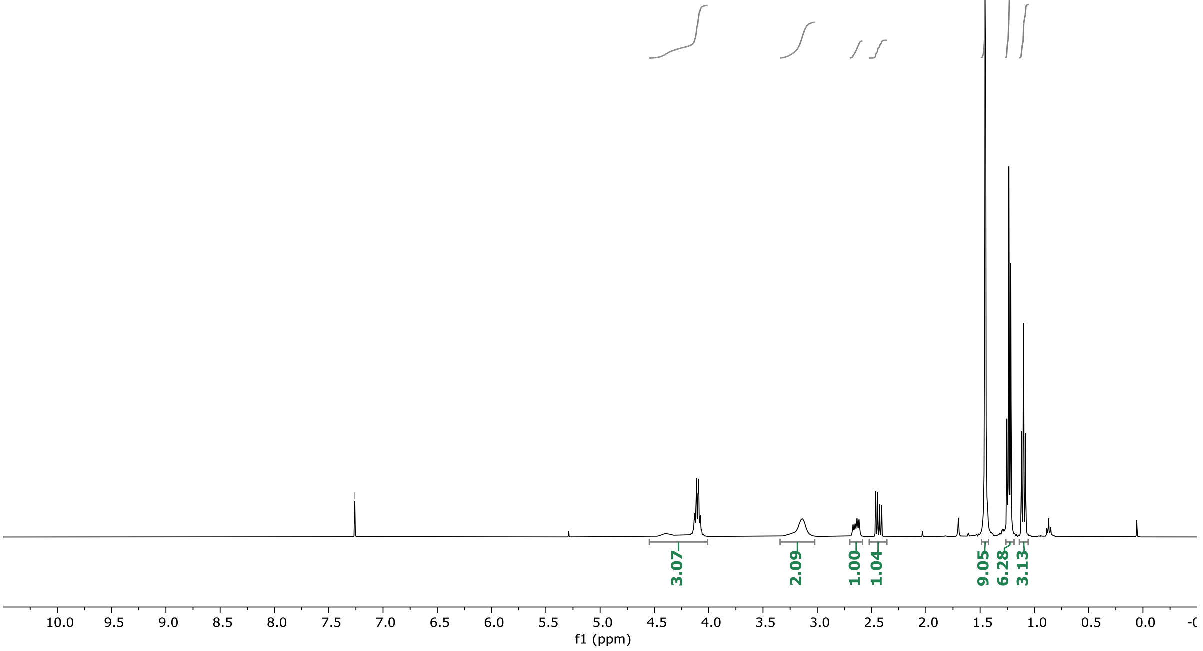

^{13}C NMR (101 MHz, $CDCl_3$) of **61**

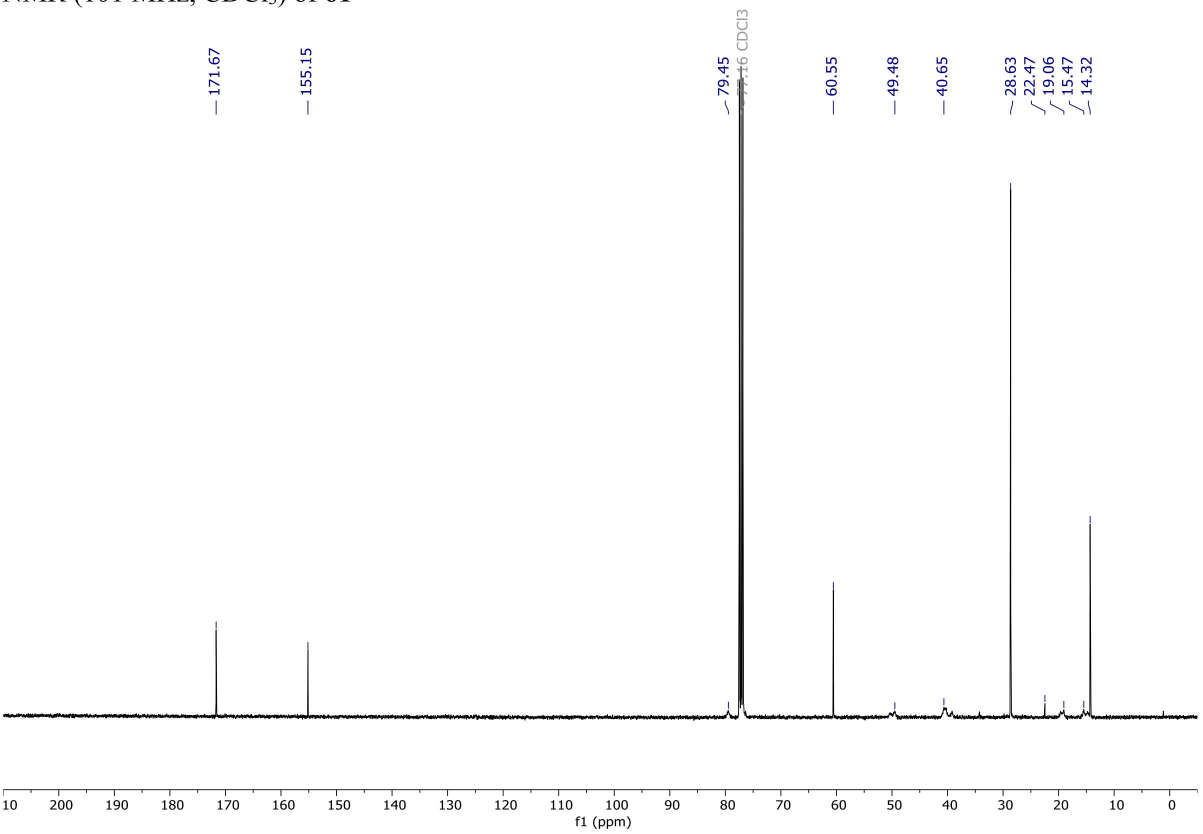

^{1}H NMR (400 MHz, $CDCl_3$) of **68**

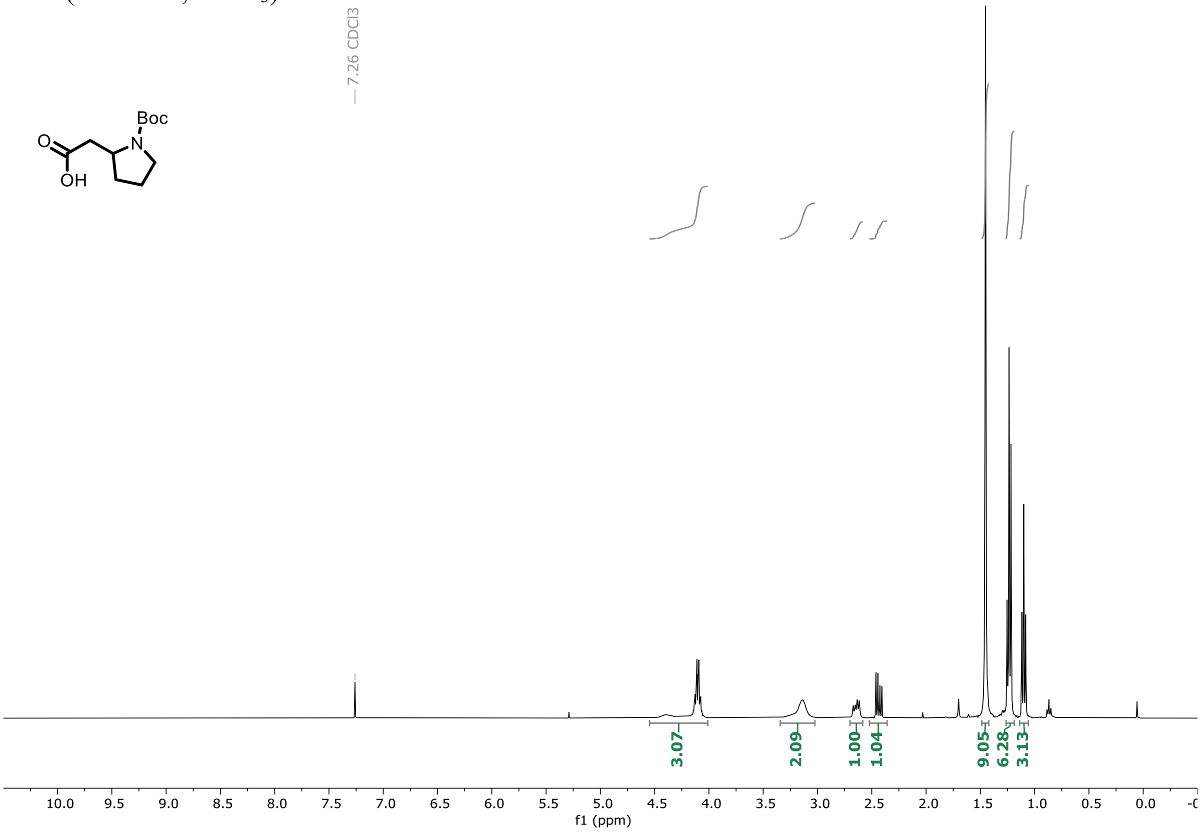

^{13}C NMR (101 MHz, $CDCl_3$) of **68**

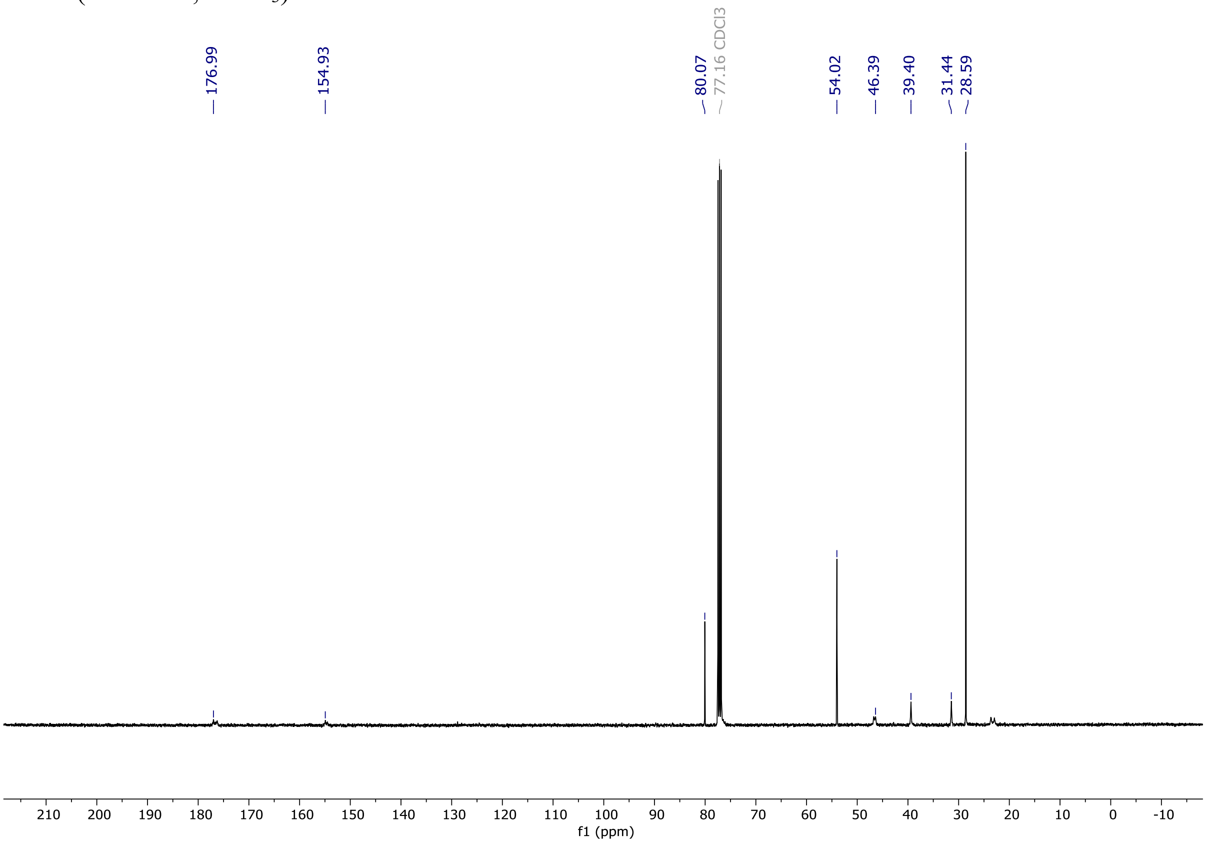

^{1}H NMR (400 MHz, $CDCl_3$) of **69**

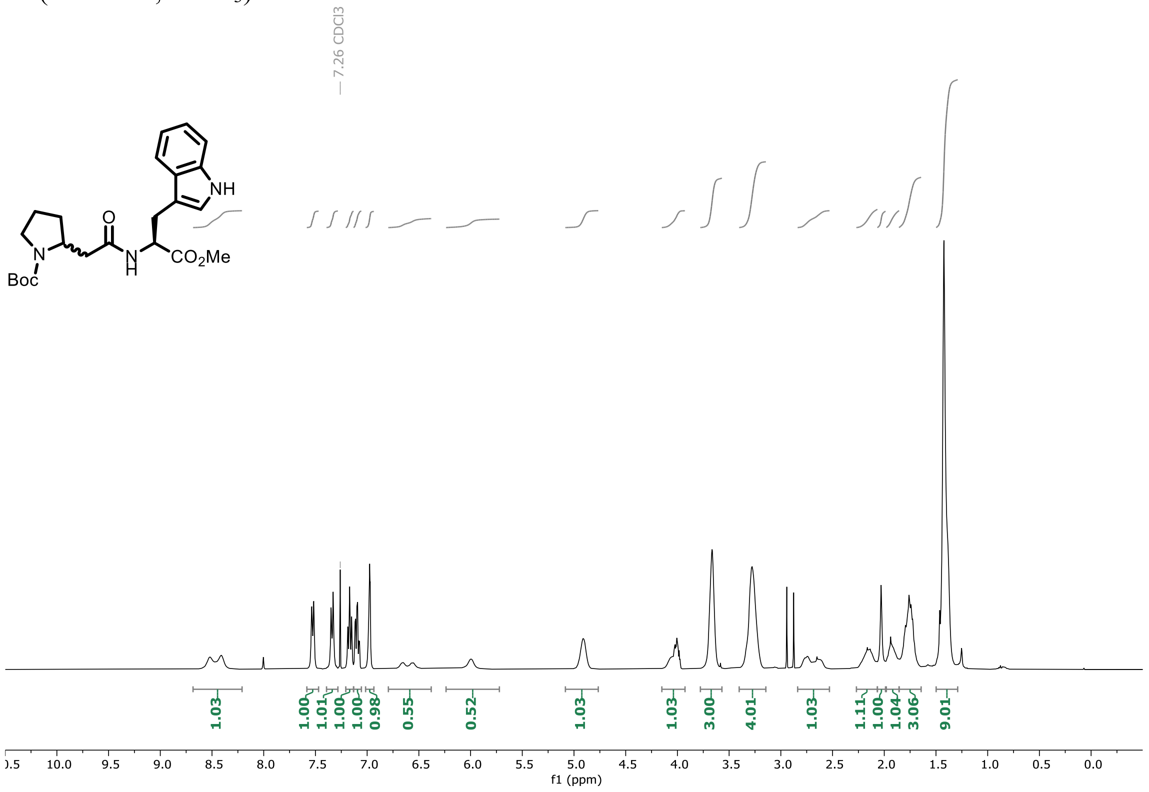

^{13}C NMR (101 MHz, $CDCl_3$) of **69**

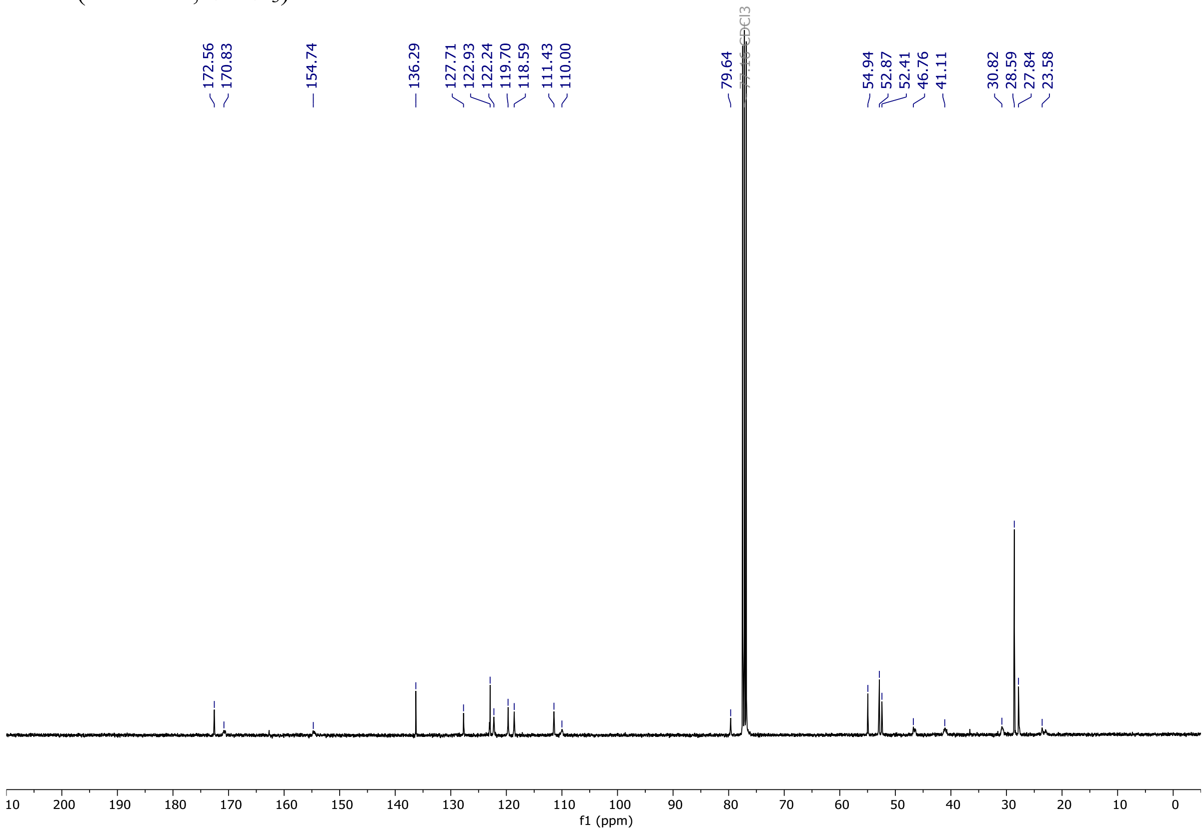

COSY (400 MHz, CDCl$_3$) of **69**

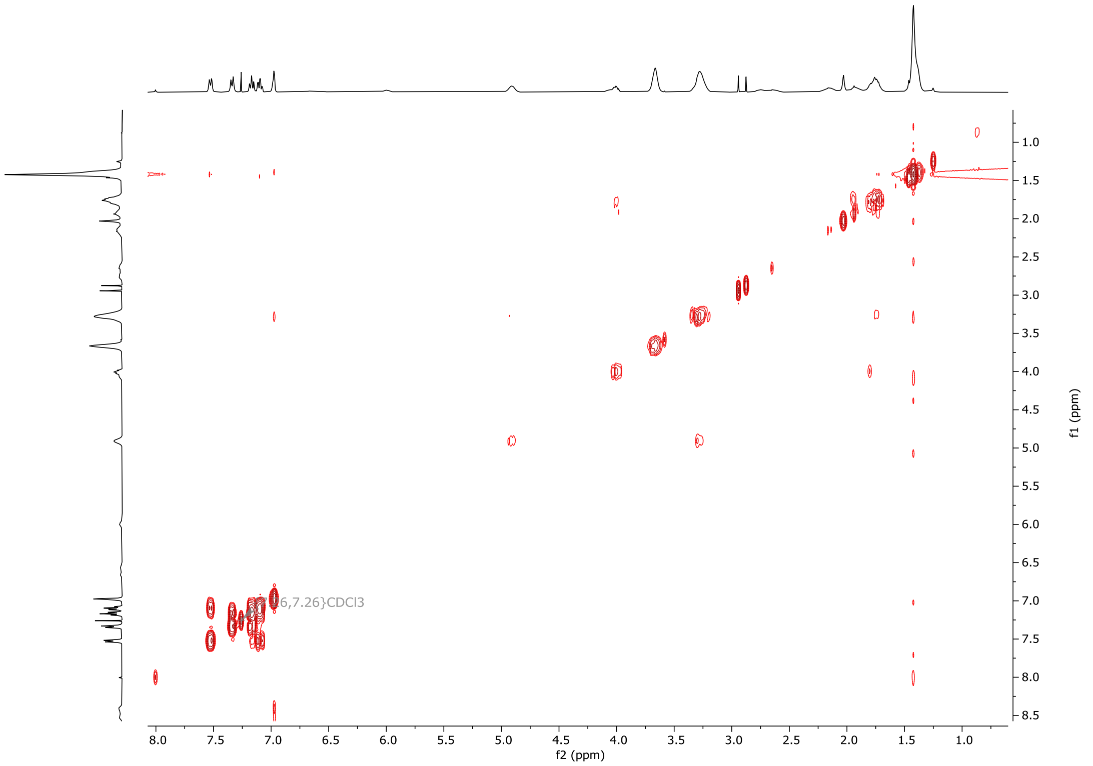

HSQC (CDCl$_3$) of **69**

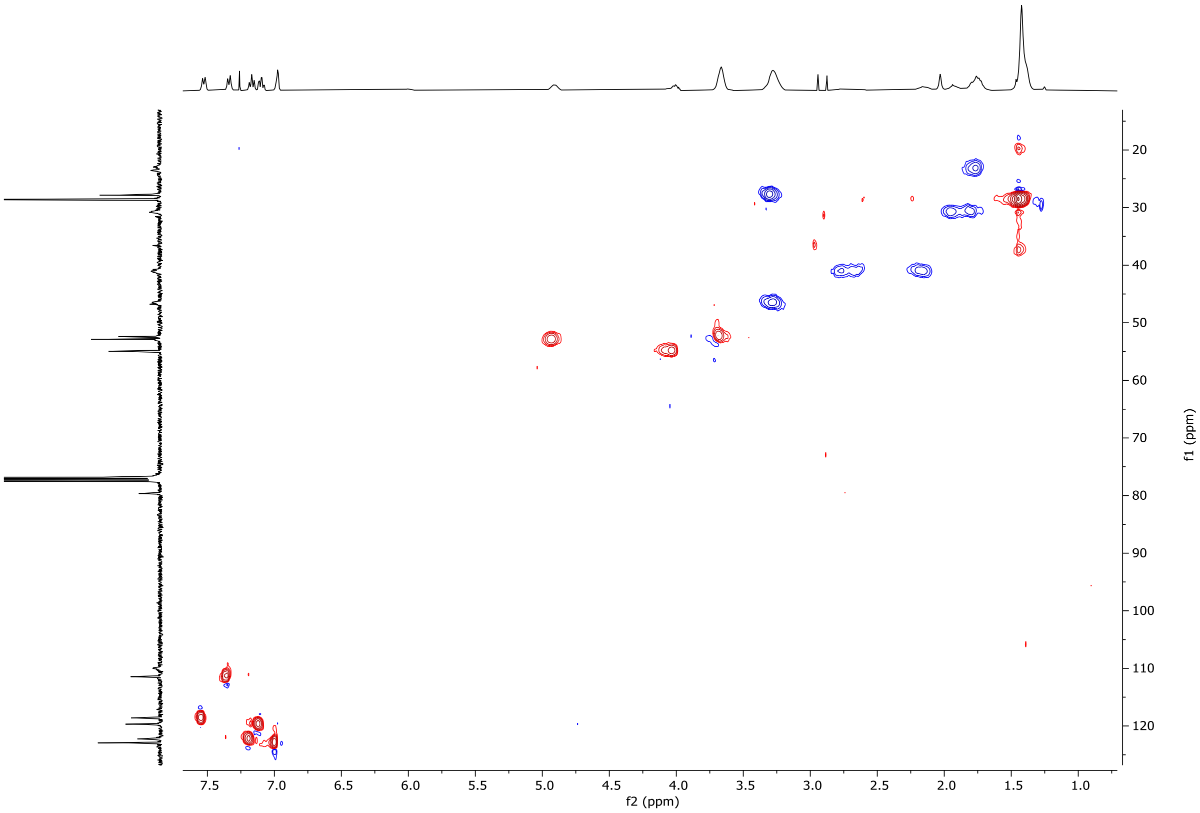

^{1}H NMR (400 MHz, $CDCl_3$) of **70**

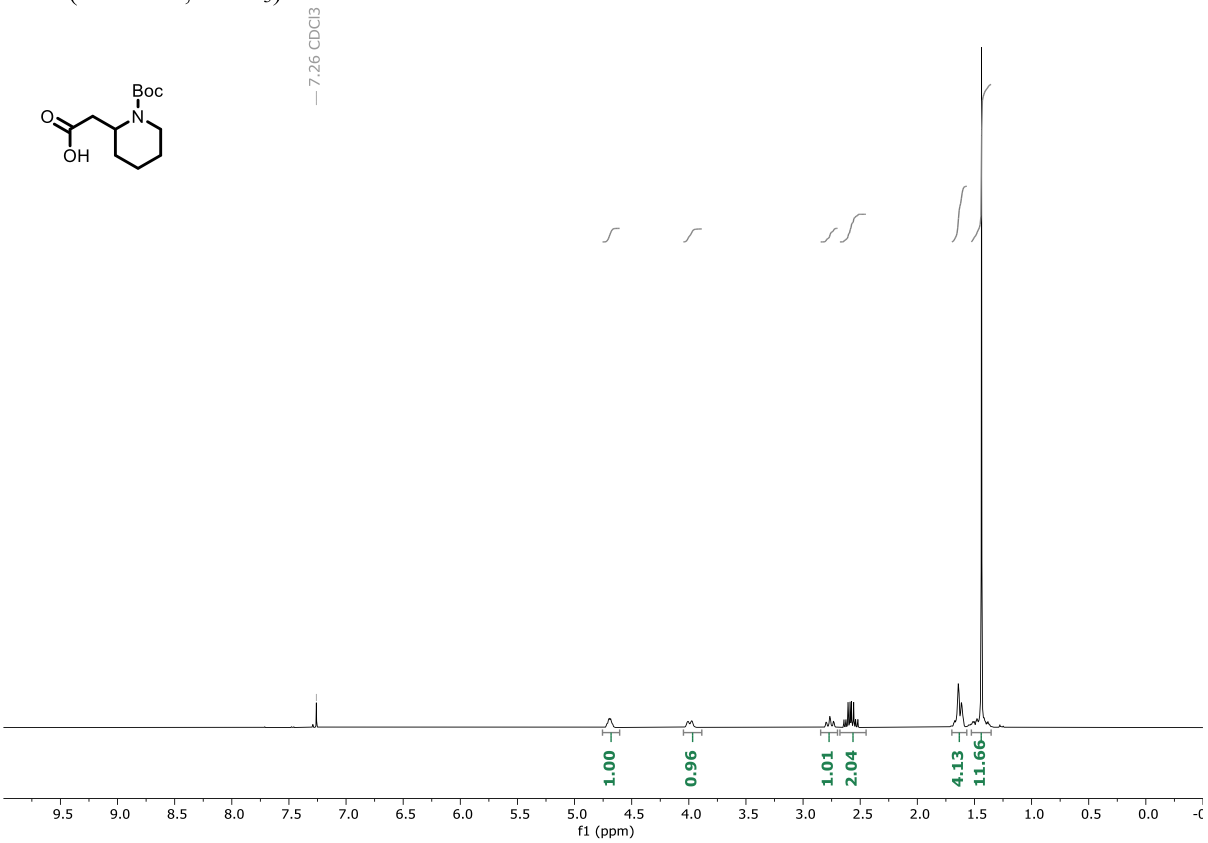

^{13}C NMR (101 MHz, $CDCl_3$) of **70**

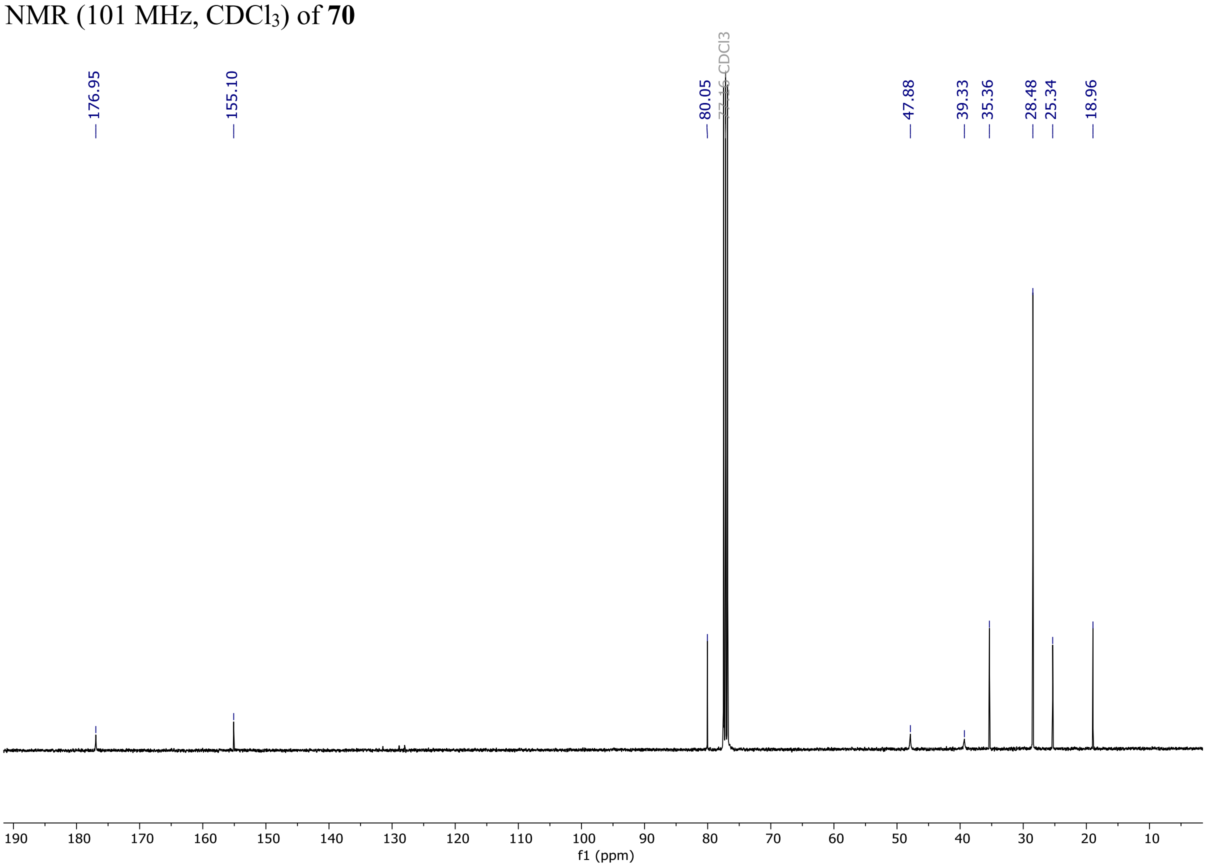

^{1}H NMR (400 MHz, $CDCl_3$) of **71**

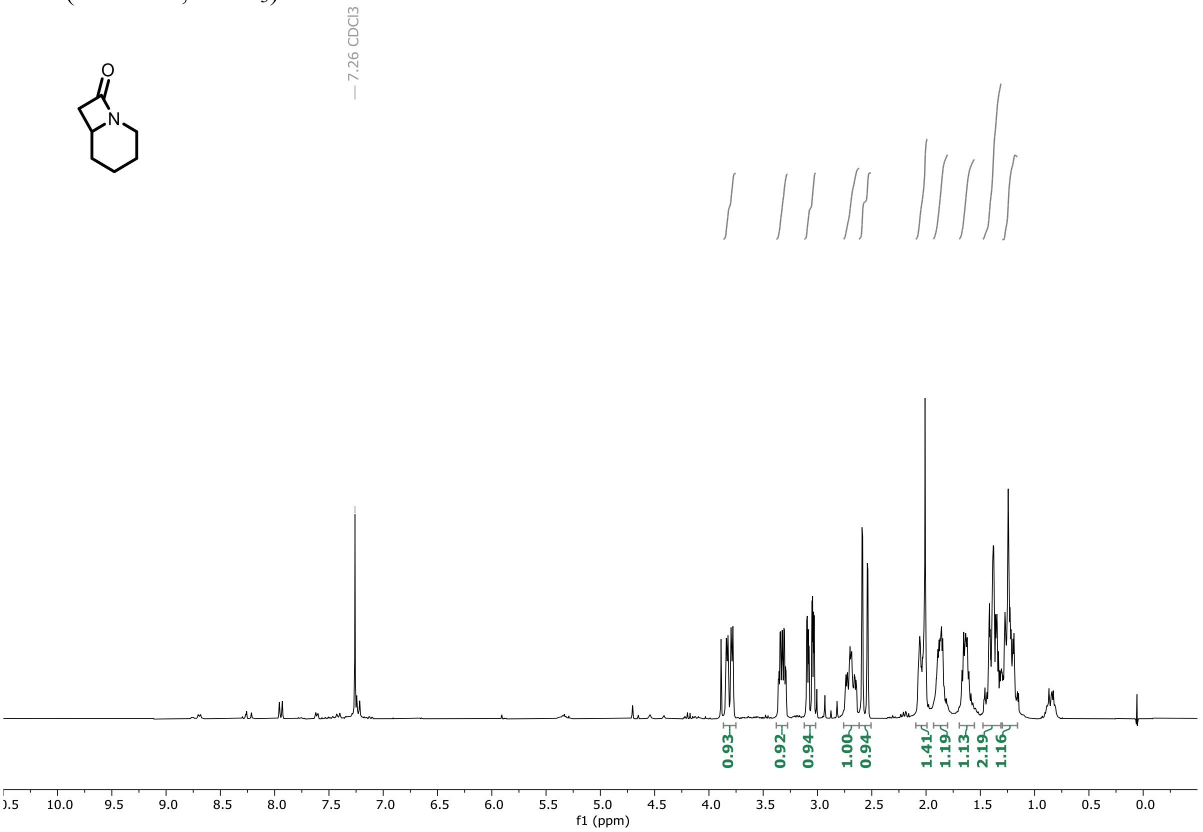

^{13}C NMR (101 MHz, $CDCl_3$) of **71**

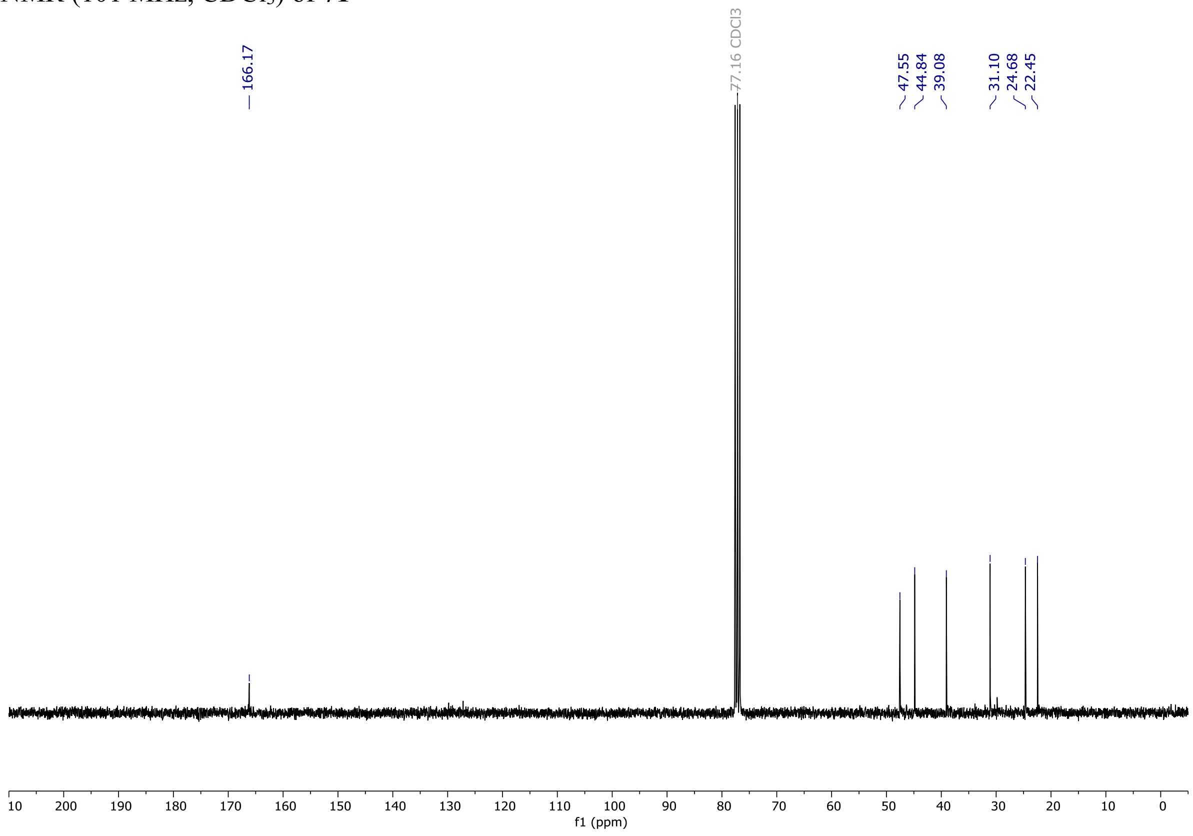

Supplement: Supplementary file 1 — Supporting Information [file ANIE-62-0-s001.pdf]
